# Photodissociation and Infrared Spectroscopy of U^+^(CO_2_)_*n*_, UO^+^(CO_2_)_*n*_, and UO^2+^(CO_2_)_*n*_ Cation-Molecular Complexes

**DOI:** 10.1021/acs.jpca.5c07786

**Published:** 2026-01-28

**Authors:** Joshua H. Marks, Richard B. Odonkor, Nathan J. Dynak, Michael A. Duncan

**Affiliations:** Department of Chemistry, University of Georgia, Athens, Georgia 30602, United States

## Abstract

Laser vaporization of uranium in a pulsed supersonic expansion of carbon dioxide is used to produce complexes of the form U^+^(CO_2_)_*n*_, UO^+^(CO_2_)_*n*_, and UO^2+^(CO_2_)_*n*_. These ions are selected in a reflectron time-of-flight mass spectrometer and studied with visible laser photodissociation and tunable infrared laser photodissociation spectroscopy in the region of the CO_2_ antisymmetric stretch. The dissociation patterns and spectroscopy of these ions indicate that CO_2_ ligands are intact molecules. Although reaction products that form oxide-carbonyl or oxalate species are predicted to be stable, there is no direct evidence in the frequency range studied for the formation of these species. There is no clear indication for the coordination numbers for singly charged uranium and its oxide complexes with CO_2_. However, there is strong support in the vibrational patterns for an eight-coordinate complex of the doubly charged UO^2+^ species, i.e., UO^2+^(CO_2_)_8_.

## Graphical abstract

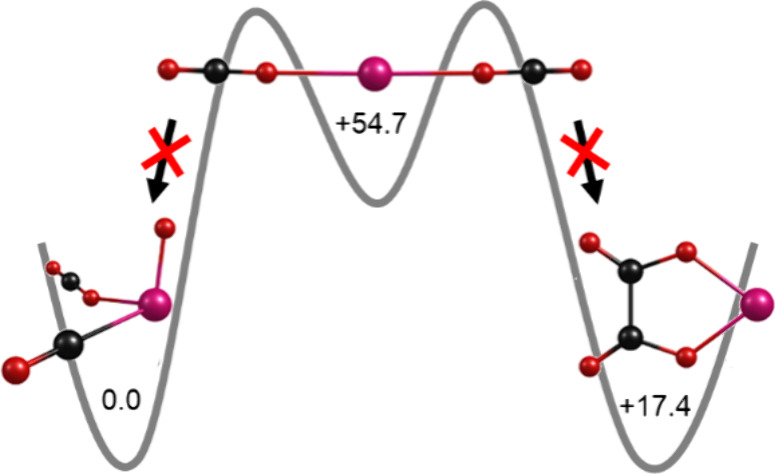

## Introduction

The electronic structure and bonding of small uranium molecules and ions provide fundamental insights into the role of *f* electrons in chemical bonding.^1–4^ Such insights are essential for applications in the disposal of nuclear waste, the development of new materials, and potential catalytic systems employing actinide elements.^1–9^ Gas phase experiments on uranium molecules and ions eliminate solvent and counterion effects, providing a direct connection to computational chemistry studies. Reaction studies in mass spectrometry,^10–15^ as well as both electronic and infrared spectroscopy,^16–37^ have been applied to these systems. Computational chemistry has employed various approaches to handle the issues of strong correlation, multireference behavior, relativistic effects and spin–orbit coupling.^38–49^ In the present study, we investigate uranium and its oxide ions in complexes with carbon dioxide molecules using infrared photodissociation spectroscopy complemented by computational chemistry. IR spectroscopy reveals the nature of the bonding and coordination, providing a comparison to transition metal cation-CO_2_ complexes studied previously.

Gas phase metal ion complexes with CO_2_ molecules have been studied previously for many transition metals in both positive and negative charge states.^50–81^ Electronic spectroscopy has employed metal ions with strong atomic transitions,^53–60^ whereas infrared spectroscopy experiments were motivated by the intense infrared absorption of CO_2_ and its well-known vibrational frequencies.^62–81^ It was also recognized in these studies that metal ions have the possibility to activate CO_2_. Reaction products such as carbonates, carbonyls or oxalate species have been suggested in previous infrared experiments on transition metal and lanthanide metal systems.^72,76–81^ In the case of V^+^(CO_2_)_*n*_ positive ions, infrared spectra documented the formation of the C_2_O_4_ oxalate species, facilitated by the solvation effect of multiple CO_2_ molecules.^72^ Other transition metal positive ions were relatively unreactive, presumably because of large activation barriers for breaking the CO_2_ bonds. Lanthanide oxide ions were found to form carbonate species.^75,76^ Anionic transition metal systems seemed to be more reactive.^78–81^ In the case of M^–^(CO_2_)_*n*_ anions (M = Ag, Au), metal–carbon bonding forms a bent OCO moiety.^78,79^ For Ni^–^(CO_2_)_*n*_ anions, the same bent configuration occurs in some cluster sizes, but others form the C_2_O_4_ oxalate structure.^81^ In all of these reactive systems, the number of interacting CO_2_ molecules is a critical variable, and reaction product formation was at least partially driven by solvation.

Similar infrared photodissociation experiments have been investigated for a number of uranium ion–molecule complexes to explore the role of *f* electrons in metal–ligand bonding.^28–37^ Complexes with large ligands have been studied with electrospray ionization sources and infrared multiphoton photodissociation (IR-MPD) spectroscopy using a free electron laser.^28–34^ In other cases, metal cation complexes with small-molecule ligands such as CO or N_2_ were produced in supersonic molecular beams with laser ablation. Higher resolution spectra were obtained in these studies, revealing details about coordination spheres and specific vibrational band shifts.^35–37^ U^+^(CO)_*n*_ and U^+^(N_2_)_*n*_ clusters were found to have filled coordination with eight ligands, with red-shifted vibrations comparable to those of corresponding transition metal ion complexes.^35,37^ Oxide carbonyls had lower coordination and blue-shifted vibrations, again similar to transition metal systems.^35^ In the present work, we investigate these kinds of issues for uranium and some of its oxide ions in complexes with CO_2_, looking for coordination numbers, vibrational frequency shifts and any evidence for possible intracluster reactions.

## Experimental Section

Uranium and uranium oxide cation-carbon dioxide complexes were produced in a pulsed supersonic expansion of pure CO_2_ with laser vaporization^82^ of a solid rod of depleted uranium. A Spectra Physics INDI Nd:YAG laser is used to produce 5–20 mJ/pulse at 355 nm for the vaporization. Mass separation and selection were carried out in a reflectron time-of-flight spectrometer described previously.^83,84^ After mass selection, photodissociation is conducted in the turning region of the reflectron, and mass analysis of the dissociation products is determined by the transit time through a second flight tube. The instrumentation and methods for photodissociation are similar to those employed in our previous studies of U^+^(CO)_*n*_, U^+^(O_2_)_*n*_ and U^+^(N_2_)_*n*_ complexes^35–38^ and that on U_*x*_O_*y*_^+^ clusters.^85^ Laser radiation for fixed-frequency photodissociation at 532 and 355 nm was produced by a Spectra Physics GCR-150 Nd:YAG laser. Tunable infrared light for photodissociation was produced with a Nd:YAG-pumped infrared OPO/OPA (LaserVision). Infrared radiation is passed through the turning region of the reflectron and retro-reflected with a concave gold folding mirror to improve the photodissociation yield.

Computational studies on uranium cation complexes were carried out with density functional theory and the u-B3LYP functional using the cc-pVTZ-PP basis set,^45^ which includes the Stuttgart/Köln fully relativistic 60 electron ECP for uranium,^43^ using the Gaussian16 program package.^86^ The relative energies for all proposed isomers of U^+^(CO_2_)_*n*_, UO^+^(CO_2_)_*n*_ and UO^2+^(CO_2_)_*n*_ were investigated for each of the different possible spin states. Vibrational frequency and natural bond orbital (NBO) analyses were conducted on all optimized structures. A scaling factor of 0.972 was determined by predicting the frequency of the CO_2_ asymmetric stretch at the same level of theory, and this was applied to all computed frequencies for comparison to the experimental spectra.

## Results and Discussion

Laser vaporization of a depleted uranium rod in an expansion of pure CO_2_ gas produces a variety of U^+^(CO_2_)_*n*_, UO^+^(CO_2_)_*n*_ and UO^2+^(CO_2_)_*n*_ complexes. Typical mass spectra are shown in Figure 1 using different ablation laser powers. The relative amounts of pure uranium versus oxide clusters varies with the expansion gas conditions and the ablation laser power. Similar variables affect the amounts of singly- versus doubly charged ions. As expected, higher ablation laser powers are required to produce the doubly charged ions. Unfortunately, we could not find conditions under which we produced usable amounts of UO_2_^+^(CO_2_)_*n*_, and so these clusters are not included in this study. As shown in Figure S1, these clusters can be produced, but the signals are not stable over time.

**Figure 1:**
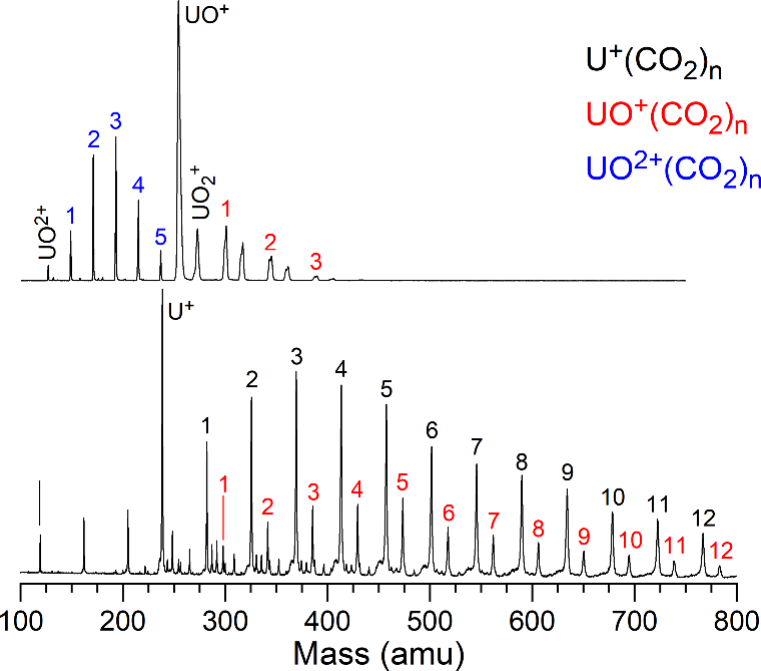
Mass spectrum obtained using a pure CO_2_ expansion and laser vaporization of a depleted uranium metal rod. The upper trace was obtained at higher vaporization laser power.

### Visible Laser Photodissociation

To investigate these complexes, we first employ visible and UV laser photodissociation. Figures 2 and 3 show the photodissociation mass spectra for U^+^(CO_2_)_*n*_ and UO^+^(CO_2_)_*n*_ ions, respectively at an excitation wavelength of 532 nm. These spectra are measured in a difference mode of operation, in which the mass-selected parent ion is measured in repeated cycles of fragmentation laser on-versus-off, taking the difference of these cycles. This produces a negative-going peak for the parent ion, showing its depletion, and positive peaks for the fragment ions. The integrated areas of depletion versus fragments do not match because of different instrument focusing on these masses. The laser pulse energy was adjusted to be about 1–5 mJ/pulse for these measurements. Higher pulse energies produced additional fragmentation assigned to multiphoton processes (see Figure S3). These spectra show that there is relatively efficient photodissociation at 532 nm for all cluster sizes, and that each eliminates a sequence of multiple CO_2_ units. There is no evidence for the elimination of CO, which might be expected from the oxide-carbonyl structures suggested by theory (see below). There is also no evidence for the loss of CO_3_ which might occur for clusters containing this carbonate moiety (see below).

**Figure 2:**
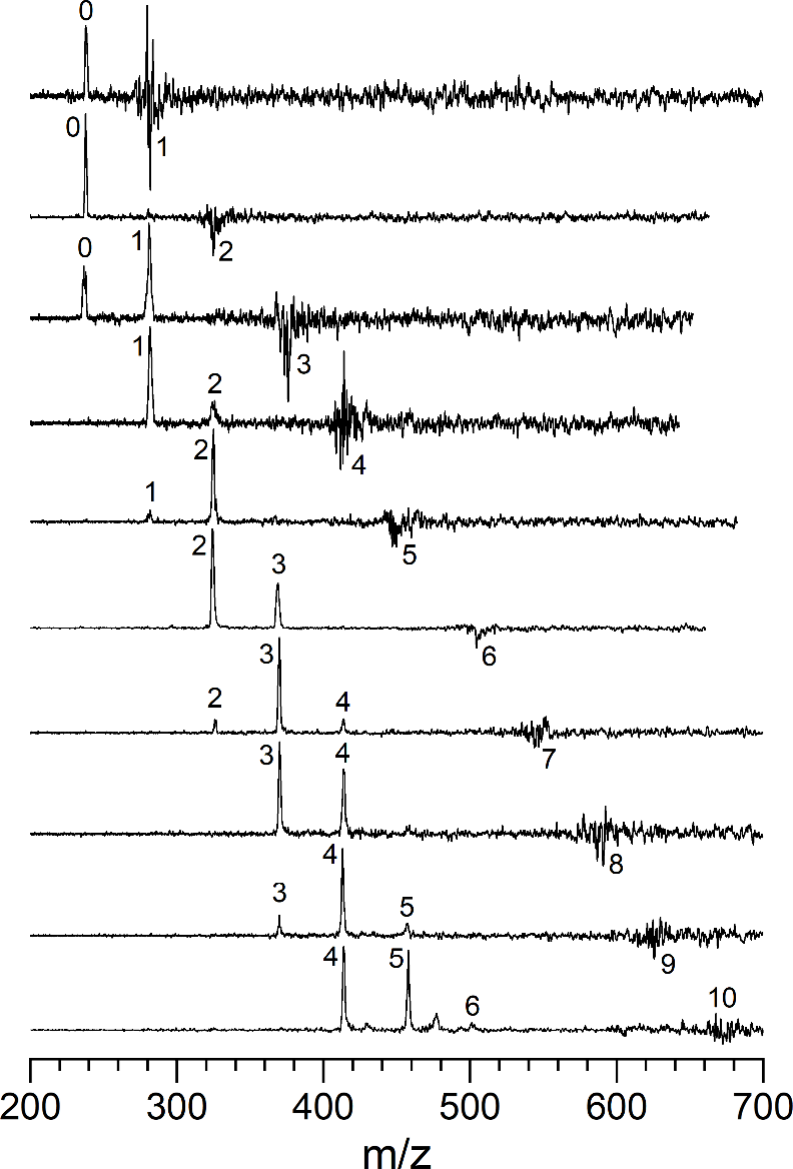
Photodissociation mass spectra of U^+^(CO_2_)_*n*_ ions at 532 nm.

**Figure 3:**
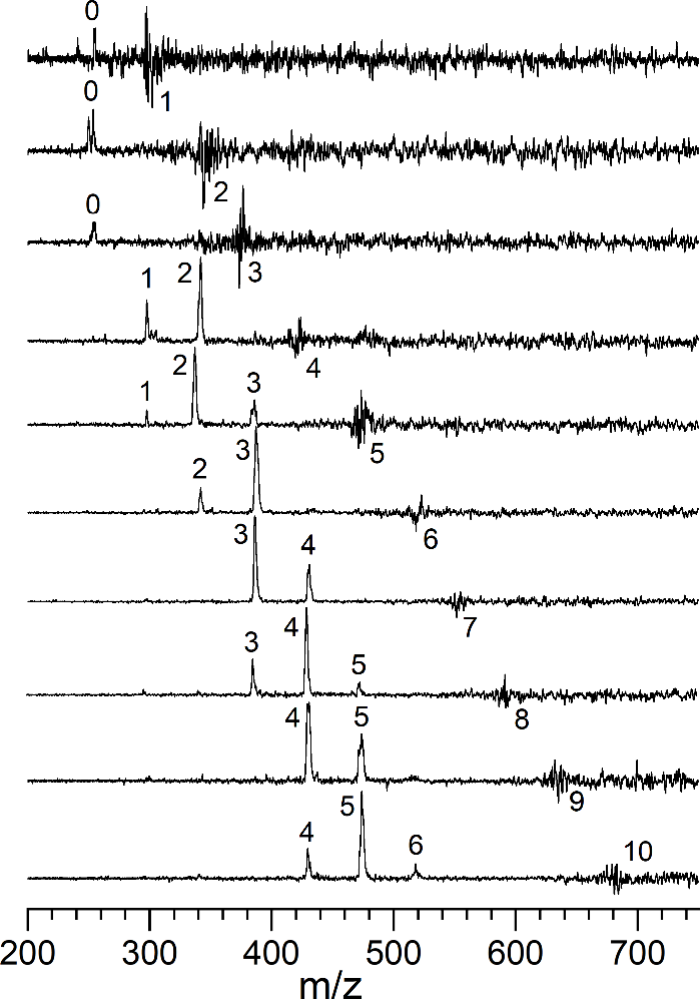
Photodissociation mass spectra of UO^+^(CO_2_)_*n*_ ions at 532 nm.

Although qualitative, these measurements provide some insights into the structures and energetics of these clusters. The elimination of only CO_2_ molecules for all cluster sizes establishes that these clusters are indeed U^+^(CO_2_)_*n*_ species (Figure 2) or UO^+^(CO_2_)_*n*_ species (Figure 3). Even though computational studies (see below) find that other isobaric structures (e.g., oxide-carbonyls or oxalates) are more stable, apparently these clusters do not rearrange efficiently to those structures. Activation of CO_2_ is known to be a high-energy process, and therefore this is not too surprising. The number of CO_2_ molecules eliminated for each cluster size is also informative, suggesting the approximate binding energies for the CO_2_ ligands. The larger clusters of the U^+^(CO_2_)_*n*_ species (*n* = 8–10) lose 5–6 ligands, whereas the smaller clusters (*n* = 3–5) lose roughly three ligands. At the 532 nm wavelength (18,796 cm^–1^ or 53.7 kcal/mol), the average bond energies would be 3100–3700 cm^–1^ for the larger clusters and about 6300 cm^–1^ for the smaller clusters. The number of ligands eliminated for the UO^+^(CO_2_)_*n*_ species are slightly less than this. These numbers are actually upper limits on the bond energies, as this analysis assumes that all the energy of photodissociation goes into ligand elimination. If the U^+^ (or UO^+^) fragments are produced in one or more excited electronic states, which seems entirely possible, then not all the energy is used for ligand elimination. However, bond energies in the 3000–6000 cm^–1^ region (8–17 kcal/mol) are not unreasonable for cation-quadrupole electrostatic bonding. The bond energy for the CO_2_ dimer is about 1.1 kcal/mol,^87^ and this should approximate the binding energy in the largest clusters which have only ligand–ligand interactions in their exterior regions. However, the cluster sizes here are probably not large enough for this limiting behavior. The clusters here should have either direct cation-ligand contact or contact between polarized ligands, and the binding energies for these should be greater than those for the neutral dimer. As shown below, some of these clusters photodissociate at infrared wavelengths near 2300 cm^–1^, suggesting that the bond energies may be even lower than suggested by the 532 nm experiments.

### Infrared Photodissociation Spectroscopy

Infrared photodissociation spectroscopy provides a more detailed way to investigate the structures of these complexes. For these measurements, we focus on the region near 2349 cm^–1^ where the isolated CO_2_ molecule has its IR-active antisymmetric stretch vibration.^88^ IR spectra in this region have been reported previously for several transition metal cation and anion complexes with CO_2_.^62–81^ Figures 4, 5, and 6 show the spectra obtained for the various U^+^, UO^+^ and UO^2+^ complexes. In some of the smallest clusters, argon tagging was employed to obtain these spectra, whereas other systems were measured in the mass channel corresponding to the elimination of one CO_2_ molecule from the cluster. As shown, all of these complexes absorb and photodissociate in this frequency region. As found in the data at 532 nm, infrared excitation leads to dissociation by the elimination of one or more CO_2_ units (see Figures S4 and S5). There is no evidence for the elimination of O or CO.

**Figure 4:**
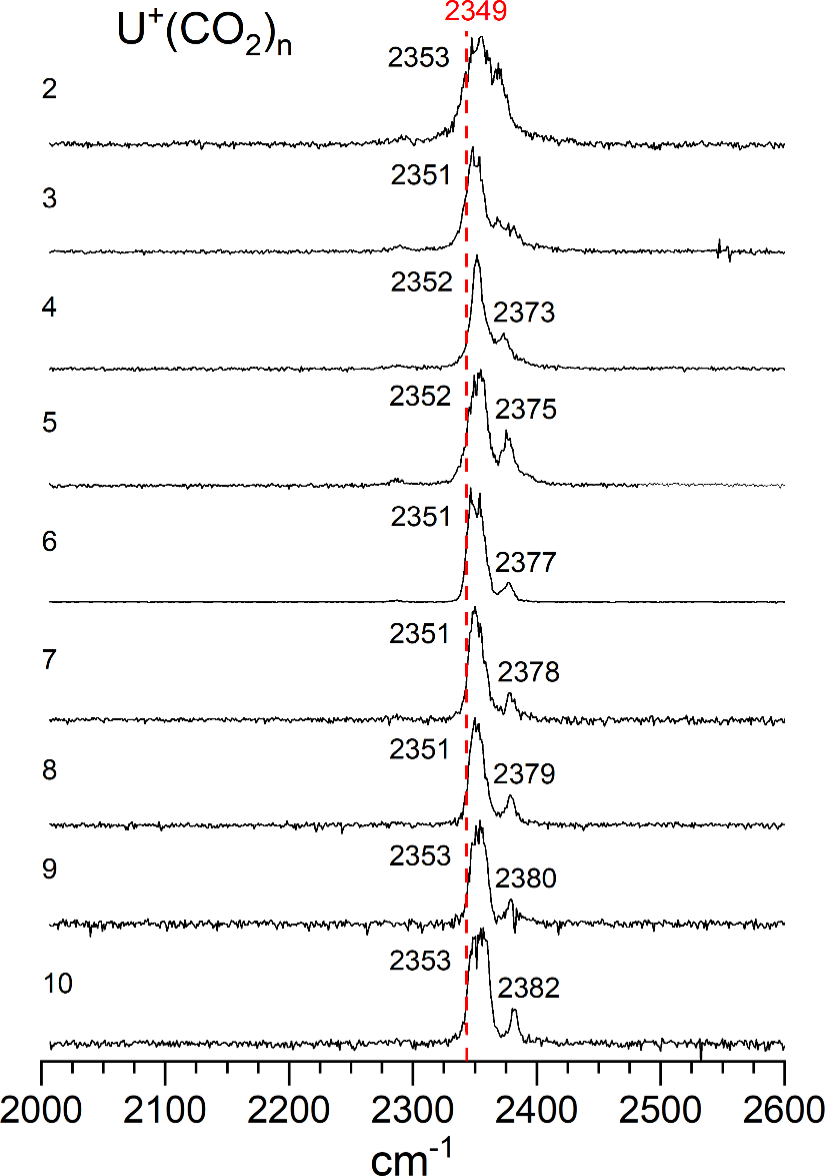
Infrared photodissociation spectra of U^+^(CO_2_)_*n*_ ions in the frequency region near the antisymmetric stretch of CO_2_.

**Figure 5:**
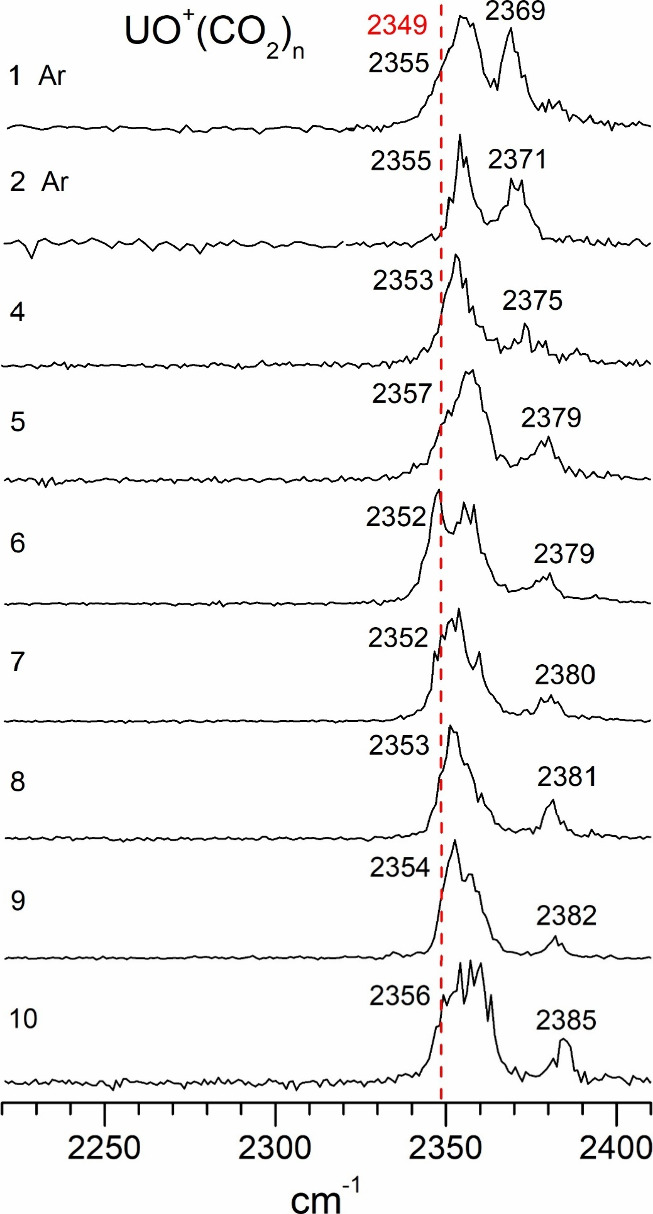
Infrared photodissociation spectra of UO^+^(CO_2_)_*n*_ ions in the frequency region near the antisymmetric stretch of CO_2_.

**Figure 6:**
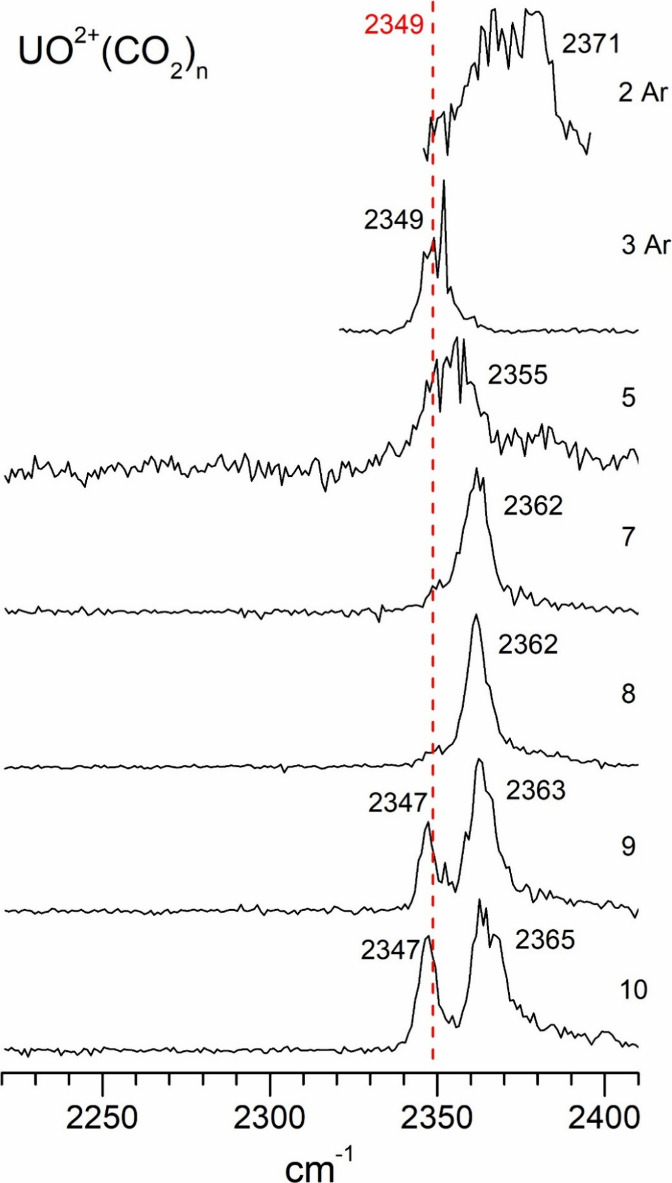
Infrared photodissociation spectra of UO^2+^(CO_2_)_*n*_ ions in the frequency region near the antisymmetric stretch of CO_2_.

All of the U^+^(CO_2_)_*n*_ clusters have a broad resonance in their photodissociation spectra near 2349 cm^–1^, and some have an additional shoulder on the high frequency side, but there are no additional bands at other frequencies. The frequency of the CO_2_ antisymmetric stretch vibration at 2349 cm^–1^ is indicated with a dashed red vertical line.^88^ As shown, all of these clusters have only a small shift to higher frequency for the CO_2_ vibration from the value for the isolated molecule. This is dramatically different from the behavior seen previously for transition metal cation-CO_2_ complexes. In those systems, for metal ions such as iron, nickel, or vanadium, the CO_2_ asymmetric stretch in the small clusters was shifted 30–50 cm^–1^ to higher frequency. This blue shift was interpreted to arise from an electrostatic interaction with a potential that is repulsive on the inner wall, driving the frequency higher. When the inner coordination was filled, a new band grew in with essentially no shift. This allowed determination of the coordination numbers for these ions. In the U^+^(CO_2_)_*n*_ system, the small vibrational band shift suggests that the interaction is weaker than it was in those transition metal systems. Likewise, no information can be obtained about the coordination number because no new bands emerge in the larger clusters. Vibrational bands for U^+^(CO)_*n*_ and U^+^(N_2_)_*n*_ clusters were red-shifted from those of the isolated molecules, just as those seen here.^35,37^

The UO^+^(CO_2_)_*n*_ clusters have spectra in this same frequency region, but each cluster size has two bands. The lower frequency band is more intense and falls slightly to the blue of the CO_2_ antisymmetric stretch (dashed red vertical line), whereas the higher frequency band is shifted 15–20 cm^–1^ higher than the lower frequency one. This behavior is again different from that of transition metal oxide ions. In those systems, oxide ions had a blue shift of the CO_2_ vibration that was even greater than the blue shifts for the metal cation complexes. Like the atomic metal ion complexes, the transition metal oxides had unshifted bands that emerged in the larger clusters, revealing the coordination numbers. The source of the two bands here could be two different environments for CO_2_, or it could be a combination with a low frequency vibration, but whatever the source, it seems to be relatively constant throughout all the cluster sizes. No new bands emerge in the larger clusters, and therefore there is no clear information about the coordination numbers.

The UO^2+^(CO_2_)_*n*_ clusters have also have spectra near the asymmetric stretch vibration, but some have single bands and some have doublets. The small clusters have single bands, but for cluster sizes above *n* = 8 there are two bands. The spectra for *n* = 2–5 are relatively noisy, probably because of the low signal levels and strong bonding, but the spectra for *n* = 7 and 8 have good signal levels, and a single peak that is shifted 13–14 cm^–1^ above the CO_2_ frequency. This behavior is more like that of the transition metal ions, where oxide vibrations were blue-shifted, although no doubly charged complexes were studied for those systems. Unlike the singly charged uranium and uranium-oxide ions, the doubly charged oxide ions have new bands that first appear at a cluster size of *n* = 9 and persist in the larger clusters. This band appears slightly red-shifted from the CO_2_ vibration. This signals the coordination number in the same way seem for various transition metal ion-CO_2_ complexes. The implication is that eight CO_2_ molecules fill the coordination around the UO^2+^ ion. A coordination number of eight was also seen previously for the U^+^(CO)_*n*_ and U^+^(N_2_)_*n*_ clusters,^35,37^ although in those systems there was not a clear indication from new vibrational bands.

The various shifts in these vibrations throughout these clusters and the multiplets produced could be caused by different cluster isomers or bonding arrangements, and therefore we turn to theory to try understand these patterns further.

### Computational Studies

To explore the structures and possible isomers of these various uranium-CO_2_ complexes, we use density functional theory, including the possible low energy spin states that might occur for U^+^, UO^+^ and UO^2+^. The complete results of these computations are presented in the Supporting Information file. Figures 7, 8, and 9 show selected examples of the lower energy structures and isomer types found for each of these cluster systems. Tables 1, 2, and 3 present the energetics for each of the isomeric structures identified for the U^+^(CO_2_)_*n*_, UO^+^(CO_2_)_*n*_, and UO^2+^(CO_2_)_*n*_ respectively.

**Table 1:** Relative Energies of Isomers for the Small U^+^(CO_2_)_*n*_ Clusters^a^

isomer	energy (hartree)	rel. energy (kcal/mol)	BDE (CO_2_) (kcal/mol)	BDE (CO) (kcal/mol)	BDE (oxalate)
1a^b^	–663.194605	+0.0		18.9	
1b	–663.112241	+51.7	13.3	–32.8	
2a^b^	–851.869871	+0.0	16.6	18.8	
2b^c^	–851.842109	+17.4	50.8	64.2	
2c	–851.782702	+54.7	13.5		
3a^b^	–1040.542000	+0.0	14.6	18.2	
3b^c^	–1040.521616	+12.8	19.2	29.0	70.0
3c	–1040.440537	+63.7	5.6	–45.4	
4a^b^	–1229.209333	+0.0	11.6	15.9	
4b^c^	–1229.197338	+7.5	16.8	28.8	73.3
4c	–1229.097086	+70.4	4.8	–54.5	
4d	–1229.095163	+71.6	3.6	–55.7	
5a^b^	–1417.871275	+0.0	8.2	14.2	
5b^c^	–1417.862848	+5.3	10.4	23.5	78.2
5c	–1417.743713	+80.0	–1.4	–68.2	
6a^b^	–1606.530093	+0.0	6.2	12.7	
6b^c^	–1606.527028	+1.9	9.6	21.2	82.9
6c	–1606.512616	+11.0	–4.7	1.8	
6d	–1606.400558	+81.3	3.6	–68.5	
7a^c^	–1795.187694	+0.0	7.4		91.8
7b^b^	–1795.185700	+1.3	4.2	14.5	
7c	–1795.167022	+13.0	3.5	13.2	
7d	–1795.083517	+65.4	21.4	–11.9	
8a^c^	–1983.845936	+0.0	5.9		92.6
8b^b^	–1983.839214	+4.2	2.9	12.9	
8c	–1983.820986	+15.7	3.2		

a According to theory, all of these are most stable in their quartet spin state.

b Oxide carbonyl structure.

c Oxalate structure.

**Table 2:** Relative Energies of Isomers for the Small UO^+^(CO_2_)_*n*_ Clusters^a^

isomer	2s + 1	energy (hartree)	rel. energy (kcal/mol)	BDE (CO_2_) (kcal/mol)	BDE (CO) (kcal/mol)	BDE (oxalate)
1a^b^	2	–738.557325	+0.0		15.7	
1b	4	–738.487666	+43.7	16.6		
1d^d^	4	–738.465316	+57.7			
2a^b^	2	–927.232105	+0.0	16.3		
2b^c^	2	–927.159853	+45.3			46.2
2c	4	–927.160732	+44.8	15.2		
2d^d^	4	–927.146047	+54.0	20.0		
3a^b^	2	–1115.904046	+0.0	14.5	13.1	
3b^c^	2	–1115.840204	+40.1	19.7		47.2
3c	4	–1115.831727	+45.4	13.9		
3d^d^	4	–1115.822217	+51.3	17.1		
4a^b^	2	–1304.570496	+0.0	11.0		
4b^c^	2	–1304.516733	+33.7	17.4		
4c	4	–1304.496418	+46.5	9.9		
4d^d^	4	–1304.489591	+50.8	11.6		
5a^b^	2	–1493.231085	+0.0	7.3	7.1	
5b^c^	2	–1493.185978	+28.3	12.8		48.3
5c	4	–1493.157574	+46.1	7.7		
5d^d^	4	–1493.154929	+47.8	10.3		

a The multiplicity is indicated as 2s + 1, because not all isomers have the same most-stable spin state. The most stable isomer for each cluster (1a, 2a, 3a, 4a, etc.) has the dioxide-carbonyl structure, whereas the second most stable isomers for *n* ≥ 2 have the oxalate structure.

b Dioxide carbonyl structure.

c Oxalate structure.

d Carbonate structure.

**Table 3:** Relative Energies of Isomers for the Small UO^2+^(CO_2_)_*n*_ Clusters

isomer	2s + 1	energy (hartree)	rel. E (kcal/mol)	BDE (CO_2_) (kcal/mol)	BDE (CO) (kcal/mol)
1a	3	–738.052286	+0.0	40.0	
1b^a^	1	–738.032576	+12.4		68.3
2a	3	–926.758963	+0.0	36.3	
2b^a^	1	–926.745858	+8.2	40.4	28.9
3a	3	–1115.454846	+0.0	29.5	
3b^a^	1	–1115.448309	+4.1	33.6	25.0
4a^a^	1	–1304.139366	+0.0	26.5	19.5
4b	3	–1304.13907	+0.2	22.2	
4c	3	–1304.138831	+0.3	22.0	
5a	3	–1492.818838	+0.0	18.9	
5b^a^	1	–1492.814834	+2.5	16.7	11.5
6a	3	–1681.488526	+0.0	13.1	
6b^a^	1	–1681.466828	+13.6		
7a	3	–1870.14912	+0.0	7.4	
7b^a^	1	–1870.131452	+11.1	9.9	8.2
8a	3	–2058.806164	+0.0	4.4	
8b	3	–2058.804757	+0.9	4.2	
8c^a^	1	–2058.78964	+10.4	5.8	3.1

a Dioxide carbonyl structure.

**Figure 7:**
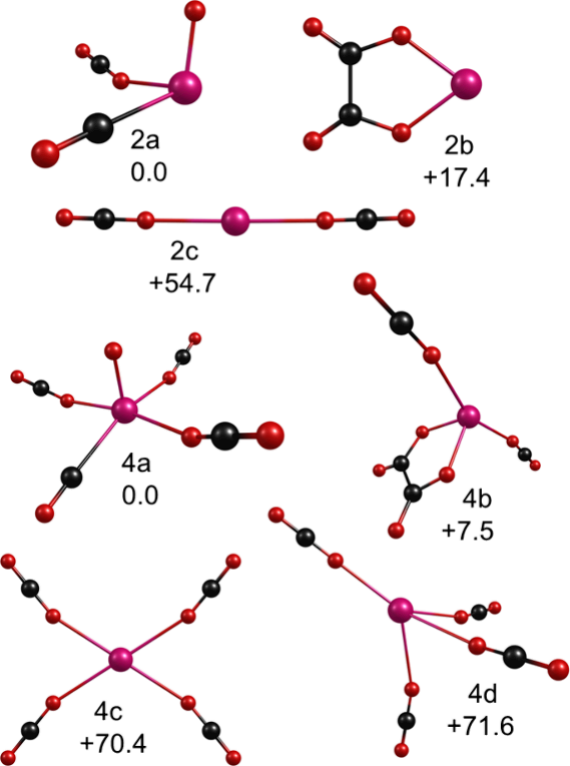
Structures determined by theory for selected cluster sizes of U^+^(CO_2_)_*n*_.

**Figure 8:**
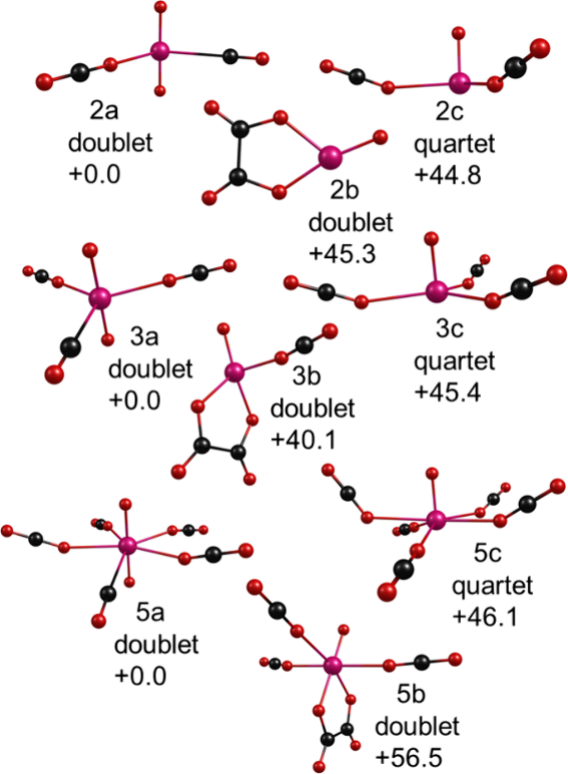
Structures determined by theory for selected cluster sizes of UO^+^(CO_2_)_*n*_.

**Figure 9:**
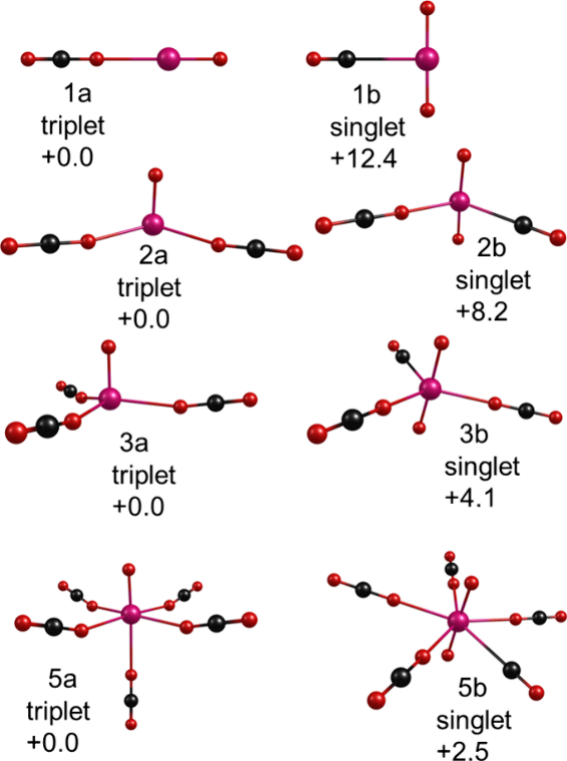
Structures determined by theory for selected cluster sizes of UO^2+^(CO_2_)_*n*_.

Figure 7 shows the isomers identified for the small U^+^(CO_2_)_*n*_ clusters, where *n* = 2 and 4. These two cluster sizes illustrate the kind of isomer types found by theory for the larger clusters. As shown, oxide-carbonyl structures are predicted to be more stable than metal ion-carbon dioxide structures. This continues to be true throughout all the cluster sizes studied. Beginning at clusters with two CO_2_ molecules, and also continuing for all larger clusters, C_2_O_4_ oxalate-containing isomers were found. In the small clusters, the oxide-carbonyl structures are more stable than those with oxalate, but oxalate structures gradually become more stable in the larger clusters. Beginning at *n* = 7, the oxalate structure is the most stable. The structures with unreacted CO_2_ molecules acting as the only kind of ligand are less stable for all cluster sizes.

Rearranged structures are also found for the UO^+^(CO_2_)_*n*_ complexes (Figure 8). Again, we have selected representative cluster sizes *n* = 2, 3, and 5 to illustrate the isomer types. In these systems, the dioxide-carbonyl structures UO_2_^+^(CO)­(CO_2_)_*n*‑1_ were found to be the most stable for all cluster sizes. These structures have a uranyl-like ion at their core, but one which is singly charged. Other isomers were found having the monoxide ion bound to an oxalate moiety - these have intermediate stability. The oxide-CO_2_ structures UO^+^(CO_2_)_*n*_ were found to be less stable for all cluster sizes. U­(CO_2_)_*n‑1*_(CO_3_)^+^ carbonate structures were also identified, but found to lie at much higher energies (see Supporting Information).

Figure 9 shows structures for the selected cluster sizes of *n* = 1, 2, 3, and 5 for the dication uranium oxide-CO_2_ complexes, UO^2+^(CO_2_)_*n*_. In this case, the dication monoxide-CO_2_ structures lie at the lowest energies for all cluster sizes except *n* = 4. Stable isomers are found for the UO_2_^2+^(CO)­(CO_2_)_*n*‑1_ structures, and these lie close in energy to the monoxide structures. At *n* = 4 the dioxide-carbonyl is the most stable, but monoxide-carbonyls become stable again at larger sizes. This is surprising because of the known stability of the uranyl dication in solution. The oxalate structures found for U^+^ and UO^+^ singly charged complexes are not stable minima for these dications. Those structures relaxed to dication-CO_2_ complexes. The same applies to carbonate structures.

### Comparison between Experiment and Theory

As noted above, the photodissociation experiments at 532 nm indicate that the only fragmentation process for all of these clusters is elimination of CO_2_. The computational data make it possible to evaluate this in the context of the bonding energies for CO versus CO_2_ versus oxalate ligands. The binding energies derived from theory are presented in Tables 1–3. It is important to mention that DFT binding energies for systems such as this are not likely to be highly accurate, and should only be used for approximate information and trends. The binding energies for CO_2_ versus CO ligands are predicted to be comparable throughout these data, in the range of approximately 12–20 kcal/mol. However, all of these binding energies are well below the energy of photons at the 532 nm wavelength. This suggests that if CO ligands were present their elimination would have been detected. The binding energies for oxalate ligands are much higher in the 50–60 kcal/mol range. These ligands would likely not be eliminated by excitation at 532 nm.

The infrared spectra measured for these clusters provide a more informative indication of structures. We have therefore selected representative examples from the spectra already presented above and now compare those to the spectra predicted by theory. The theory employed is harmonic, and therefore needs to be scaled to account for anharmonicity in these complexes. We calculated the asymmetric stretch vibrational frequency for an isolated CO_2_ molecule and then derived a factor of 0.972 needed to bring this into alignment with the known experimental value for this frequency (2349 cm^–1^).^88^ We then applied this to all the vibrations for the various structures identified by theory. This method may not be sufficient to account for anharmonicity in all of these systems, but without anharmonic theory it is the best initial approach. It should be noted that the infrared photon energies near the CO_2_ asymmetric stretch (2349 cm^–1^; 6.7 kcal/mol) are well below the computed binding energies of either the CO or CO_2_ ligands in the small clusters. However, infrared photodissociation without the need for argon tagging is detected for several of these species (see Figures S4 and S5). This is only possible if the ligand binding energies are greatly overestimated by DFT, or if the signals detected are the result of two-photon absorption. Either of these is possible. Although the spectra are measured with low laser pulse energies, resonant absorption to an excited vibrational state followed by sequential absorption of a second photon at the same frequency can be efficient, and laser power studies would not be able to detect this.

Figure 10 shows the infrared spectrum measured for the U^+^(CO_2_)_2_ complex compared to the spectra predicted by theory for the oxide-carbonyl-CO_2_ complex (isomer 2a; most stable), the oxalate complex (isomer 2b), and the complex with two intact CO_2_ molecules attached to U^+^ (isomer 2c; least stable). For each of these the quartet spin states lie at lower energy than the corresponding doublets or sextets (see Supporting Information). Isomer 2a should have a band near the CO_2_ asymmetric stretch, consistent with the experimental band at 2353 cm^–1^, but it should also have a carbonyl stretch at 2066 cm^–1^ which is not observed. The oxalate species should have no band near the CO_2_ asymmetric stretch, where we detect a strong feature, but should instead have a band near 1855 cm^–1^, which is outside the range of the experiment. Oxalate bands in this region were detected previously for V^+^(CO_2_)_*n*_ clusters.^72^ Isomer 2c with two CO_2_ ligands should have a band near 2363 cm^–1^ consistent with our experimental band. Considering these vibrational patterns together with the fragmentation pattern at 532 nm (loss of two CO_2_ molecules), the simplest explanation for these spectra is that we have isomer 2c. This suggests that there is a significant activation barrier to breaking into the CO_2_ bonds to make more stable structures like isomer 2a, which is understandable. It is not so obvious that the formation of oxalate would require a high activation barrier, and indeed we cannot rule out some formation of this isomer. Its spectrum is outside the range of the experiment, and if it were eliminated by fragmentation it would have the same mass loss as two CO_2_ units. However, the strong band at 2353 cm^–1^ and the lack of any band near 2066 cm^–1^ in the experiment suggests that most of these ions have the 2c unreacted structure. Arguments such as this apply to all of the U^+^(CO_2_)_*n*_ clusters studied here. As shown in the Supporting Information, oxide-carbonyl structures are predicted to be stable, but no carbonyl stretch vibrations are ever detected. Oxalate structures are predicted, and in the larger clusters these are found to be the most stable isomers, but these could only be confirmed by bands near 1850 cm^–1^, which is outside the range of the present experiments. Both unreacted clusters and the larger ones containing oxalate would have bands near 2350 cm^–1^, consistent with the experiment. Future experiments will extend the spectroscopy to the lower frequency range to determine if there is oxalate in any of these clusters.

**Figure 10:**
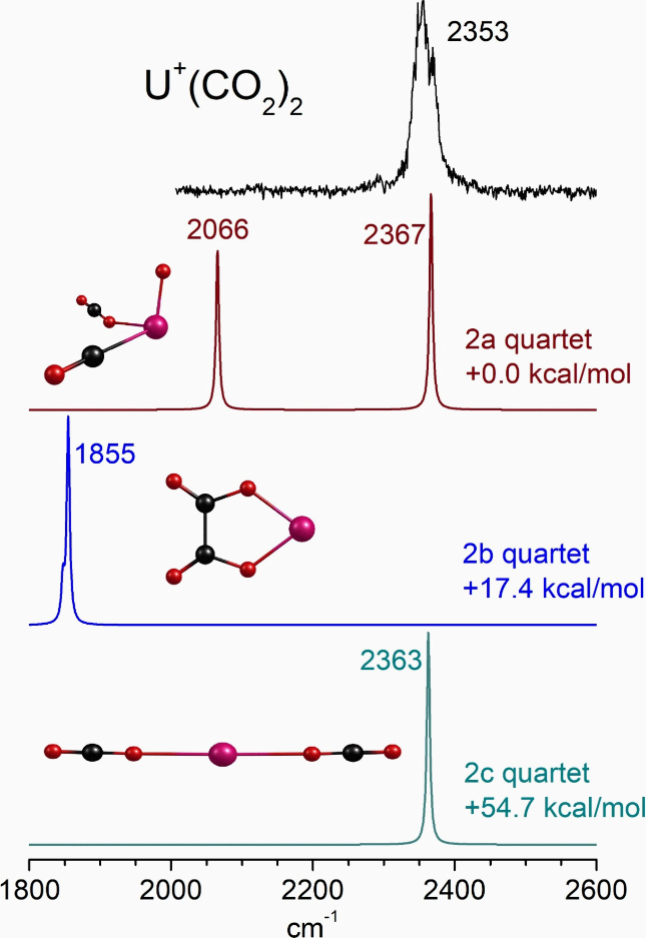
Infrared photodissociation spectroscopy of U^+^(CO_2_)_2_ compared to the spectra predicted by theory for different isomers of this ion.

Figure 11 shows the infrared spectrum of UO^+^(CO_2_)_4_, which is selected as an example of the singly charged oxide clusters. As shown in Figure 8, dioxide-carbonyl structures are predicted to be most stable for these clusters, and this is true here for isomer 4a. This isomer should have a carbonyl stretch vibration near 2200 cm^–1^, which is not detected. It should also have a UO_2_ oxygen stretch near 900 cm^–1^ that is outside the range of the experiment. Isomer 4b should have oxalate bands near 1800 cm^–1^ and low frequency oxygen stretches that are also outside the range of the experiment. Its computed stability is also much less than isomer 4a. All of these isomers should have CO_2_ ligands whose asymmetric stretch vibrations should be near that of isolated CO_2_ (2349 cm^–1^), as well as symmetric stretch vibrations near 1330 cm^–1^.^88^ In the asymmetric stretch region, the experiment has a strong band at 2353 cm^–1^ and a shoulder at 2375 cm^–1^. These features, like all of those for the UO^+^(CO_2_)_*n*_ clusters, are slightly blue-shifted from the isolated CO_2_ vibration. Theory predicts that all of these should be blue-shifted to higher frequencies than the CO_2_ vibration, but predicts a slightly higher blue shift than that observed. Theory also does not predict the pronounced doublet band structure detected throughout these clusters. As discussed above, it is conceivable that a small amount of isomer 4a is present and not detected because the carbonyl stretch is weak. It is also conceivable that the oxalate species is present and not detected because its low-frequency vibrations are out of the experimental range and its CO_2_ vibrations are not significantly different from those of the other isomers. A similar argument applies to carbonate species, although these are predicted to be much less stable. However, unreacted clusters with intact CO_2_ ligands throughout are also consistent with the infrared spectra and with the 532 nm fragmentation data. It is again clear that spectra at frequencies below 2000 cm^–1^ will be required to confirm the actual structures present for these clusters. Similar arguments apply for all of the singly charged oxide clusters.

**Figure 11:**
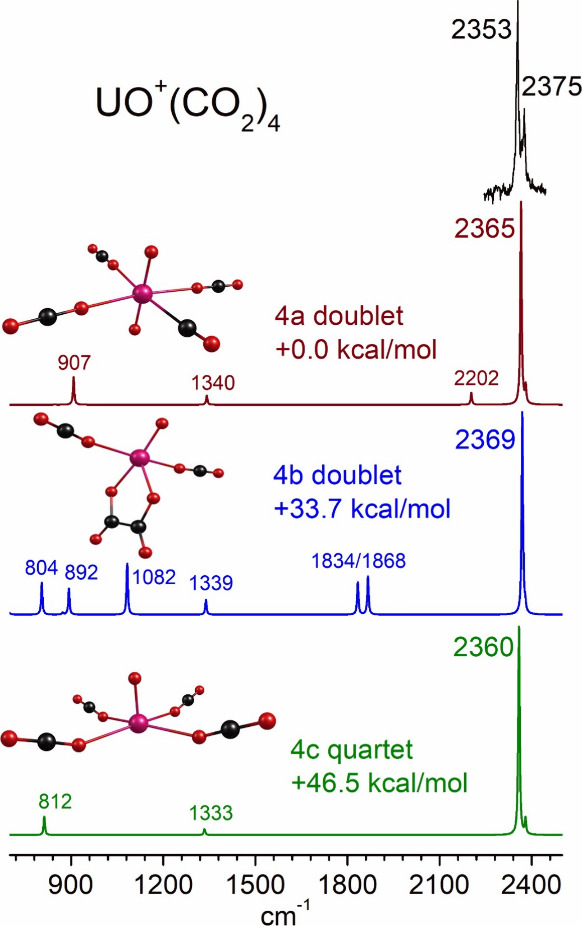
Infrared photodissociation spectroscopy of UO^+^(CO_2_)_4_ compared to the spectra predicted by theory for different isomers of this ion.

Figure 12 shows the infrared spectrum of UO^2+^(CO_2_)_5_ compared to the spectra predicted by theory for different isomers of this ion. As noted earlier, the dioxide structures are most stable for the singly charged ions, but surprisingly these are quite unstable for all the doubly charged ions. The dioxide carbonyls ions have a carbonyl stretch predicted near 2250 cm^–1^, but this vibration is predicted to have weak IR intensity. There is no evidence in the spectroscopy for a band here. However, the intensities of the mass peaks for the small doubly charged ions is much less than those for the corresponding singly charged ions and so the spectra are noisier. The spectra for the unreacted UO^2+^(CO_2_)_5_, and all the other small clusters like this, are predicted to have a single band in the region of the CO_2_ asymmetric stretch.

**Figure 12:**
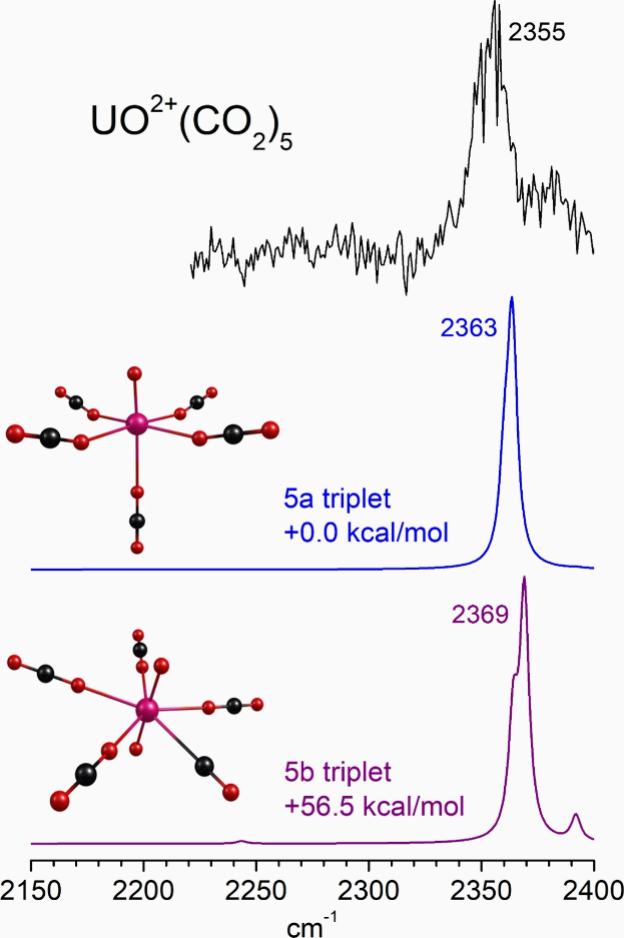
Infrared photodissociation spectroscopy of UO^2+^(CO_2_)_5_ compared to the spectra predicted by theory for different isomers of this ion.

As noted above, the larger UO^2+^(CO_2_)_*n*_ clusters have a second band that emerges at the cluster size of *n* = 9 and persists for *n* = 10. By analogy with systems studied previously,^63–72^ this seems to indicate the presence of second-sphere ligands, suggesting that the coordination around UO^2+^ is filled with eight molecules. Figure 13 shows the spectra for the *n* = 8 and 9 complexes compared to the prediction of theory for two isomers of the *n* = 8 species. Isomer 8a has only CO_2_ molecules clustering around the UO^2+^ ion, with seven of these attached to the metal center and one in the second sphere. The infrared spectrum predicted for this has a band near the frequency of the isolated CO_2_ molecule, suggesting a second-sphere ligand. Isomer 8b has a higher symmetry structure with three CO_2_ molecules opposite the oxide oxygen and five around the waist of the oxide with their linear structures tilted upward toward the oxide oxygen. This structure has an energy virtually the same as that of isomer 8a, but its infrared spectrum has a single band consistent with the experiment. The pattern of predicted and observed bands is therefore consistent with a full coordination for the *n* = 8 species, and one external molecule for the *n* = 9 species. The previously studied U^+^(CO)_8_ and U^+^(N_2_)_8_ clusters had a similar coordination of eight ligands, but their structures were square antiprisms.^35,37^ The structure suggested here for the *n* = 8 complex is somewhat less symmetric, but nevertheless fascinating and not seen previously to our knowledge.

**Figure 13:**
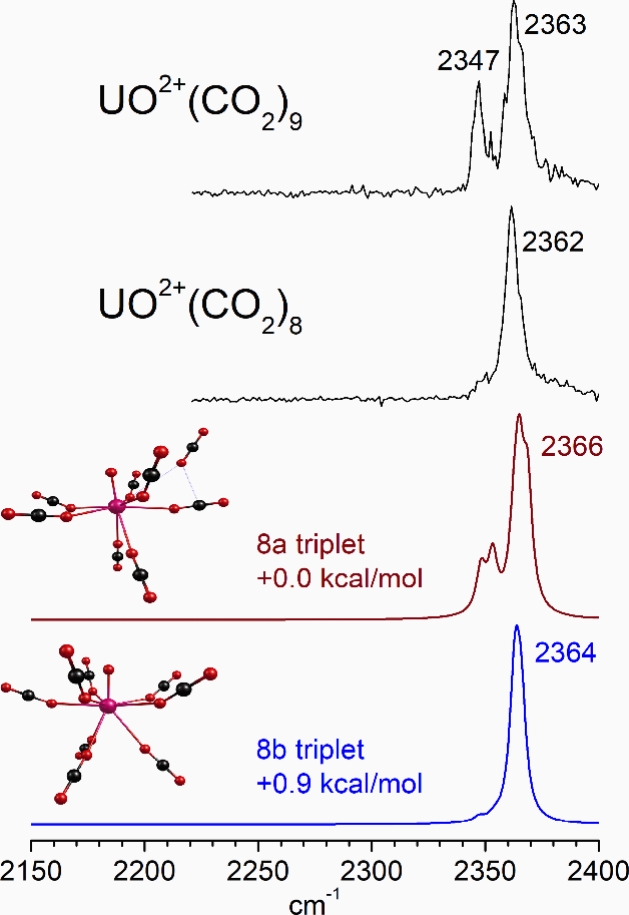
Infrared photodissociation spectroscopy of UO^2+^(CO_2_)_8_ and UO^2+^(CO_2_)_9_ compared to the spectra predicted by theory for different isomers of the UO^2+^(CO_2_)_8_ ion.

## Conclusions

CO_2_ complexes with U^+^, UO^+^ and UO^2+^ have been produced and studied with mass spectrometry and laser photodissociation measurements. Visible laser photodissociation at 532 nm causes elimination of one or more intact CO_2_ molecules for all clusters studied. There is no elimination of CO which could conceivably occur for some isomeric structures. Infrared laser photodissociation also leads to elimination of CO_2_. Infrared spectroscopy experiments produce resonances near the CO_2_ asymmetric stretch vibration. The U^+^(CO_2_)_*n*_ species all have resonances near the frequency of the CO_2_ stretch vibration at 2349 cm^–1^. The UO^+^(CO_2_)_*n*_ species all have resonances with a doublet structure shifted to the blue from the CO_2_ vibration. The small UO^2+^(CO_2_)_*n*_ species all have single-band resonances shifted to the blue from the CO_2_ vibration, which changes to a doublet feature for cluster sizes larger than *n* = 8.

Computational studies find that reacted structures, with activated CO_2_ forming different isomeric structures, are generally more stable than unreacted structures. Oxide-carbonyl and oxalate species are found for the U^+^(CO_2_)_*n*_ species, whereas dioxide-carbonyl and oxalate structures are found for the UO^+^(CO_2_)_*n*_ species. Reacted carbonate structures are found to be much less stable for these systems. UO^2+^(CO_2_)_*n*_ species are predicted to form unreacted cation-CO_2_ structures for all cluster sizes. Oxide-carbonyl and dioxide-carbonyl isomers are predicted to have a weak C–O stretch in the range of the experiment, but this vibration is not detected for any of these clusters. Oxalate clusters are predicted to be the most stable structures for U^+^(CO_2_)_*n*_ species after *n* = 7, but there is no distinctive IR signature for this moiety in the range of the experiment. Dioxide-carbonyl structures should have oxygen stretches near 900 cm^–1^, which are also outside the range of the present measurements. Both of these kinds of clusters have unreacted CO_2_ ligands near the same frequencies as those for other clusters. It is therefore not possible from the present data to rule out some contribution from oxalate or dioxide species. Future measurements in the frequency region below 2000 cm^–1^ will be required for further insight into these possible isomers. Reacted structures can be ruled out for several clusters predicted to contain carbonyl a ligand, where the C–O stretch is not detected. It is also conceivable that none of these clusters have reacted with CO_2_ as there are no distinctive bands to suggest reactions. If this is the case, it would be because there are large activation barriers to CO_2_ activation, which is completely understandable.

The computational studies here are useful, but they raise several questions. The vibrational bands for the U^+^(CO_2_)_*n*_ species fall near the position of the CO_2_ asymmetric stretch vibration, and theory agrees that these vibrations should have no large shifts. The vibrations for the UO^+^(CO_2_)_*n*_ and UO^2+^(CO_2_)_*n*_ clusters are predicted to have frequencies shifted to the blue from the CO_2_ vibration, consistent with the experiment. However, the pronounced doublet structure of the UO^+^(CO_2_)_*n*_ clusters is not captured by theory. On the other hand, the doublet structure that emerges in the UO^2+^(CO_2_)_*n*_ clusters after *n* = 8 is predicted by theory, confirming the coordination number of this ion.

Future studies will examine the frequency range below 2000 cm^–1^ for these clusters where uranium dioxide and oxalate vibrations are predicted. Additional studies will scan the dissociation thresholds of these clusters to measure bond energies, another way to test the theory. Additional computational work will investigate activation barriers for reaction in the small clusters studied here. Although CO_2_ is a relatively small ligand, its coordination behavior with these uranium ions continues to be quite interesting.

## Supplementary Material



## Notes

Details of the DFT computations done in support of the spectroscopy presented here, including the structures, energetics, and simulated vibrational spectra for each of the complexes considered (PDF)
