# Supplementary material for: Photodissociation and Infrared Spectroscopy of U+(CO2) n , UO+(CO2) n , and UO2+(CO2) n Cation-Molecular Complexes
Source: J Phys Chem A. 2026 Jan 28;130(6):1292–303. doi: 10.1021/acs.jpca.5c07786 (PMC12908122; doi:10.1021/acs.jpca.5c07786)
Supplement: Supplementary file 1 [file jp5c07786_si_001.pdf]

Supporting Information

Photodissociation and Infrared Spectroscopy of $U^+(CO_2)_n$, $UO^+(CO_2)_n$ and $UO^{2+}(CO_2)_n$ Cation-Molecular Complexes

Joshua H. Marks,[1] Richard B. Odonkor,[1] Nathan J. Dynak,[1] Michael A. Duncan[1]*

[1]Department of Chemistry, University of Georgia, Athens, Georgia 30602, U.S.A.

*Email: maduncan@uga.edu

Table of Contents

Computational details	S7
Figure S1: mass spectrum of $U^+(CO_2)_n$	S8
Figure S2: photodisociation mass spectra of $U^+(CO_2)_6$ at 355 nm wavelength	S9
Figure S3: photodissociation mass spectra of $U^+(CO_2)_6$ at 532nm wavelength	S10
Figure S4: Infrared photodissociation mass spectra of $UO^+(CO_2)_n$	S11
Figure S5: Infrared photodissociation mass spectra of $UO^{2+}(CO_2)_n$	S12
**(CO_2)**	
Table S1: energy	S13
Figure S6: structural parameters & unscaled vibrational frequencies	S14
**U^+**	
Table S2: relative energies	S15
**$U^+(CO_2)$**	
Table S3: relative energies	S16
Figures S7: predicted minimum energy structures	S17
Figures S8-17 : structural parameters & unscaled vibrational frequencies	S18 – S27
Figure S9: simulated spectra	S28
**$U^+(CO_2)_2$**	
Table S4: relative energies	S29
Figures S19 : predicted minimum energy structures	S30
Figures S20 – S28 : structural parameters & unscaled vibrational frequencies	S31 – S39
Figure S29: simulated spectra	S40
**$U^+(CO_2)_3$**	
Table S5: relative energies	S41
Figures S30: predicted minimum energy structures	S42
Tables S6 – S14: Cartesian coordinates & unscaled vibrational frequencies	S43 – S51
Figure S31 – : simulated spectra	S52
**$U^+(CO_2)_4$**	
Table S15: relative energies	S53
Figures S32: predicted minimum energy structures	S54
Tables S16 – S26 : Cartesian coordinates & unscaled vibrational frequencies	S55 – S65
Figures S33 – S34: simulated spectra	S66 – S67
**$U^+(CO_2)_5$**	
Table S27: relative energies	S68
Figures S35: predicted minimum energy structures	S69
Tables S28 – S35: Cartesian coordinates & unscaled vibrational frequencies	S70 – S77

Figures S36 – S37: simulated spectra S78 – S79

$U^+(CO_2)_6$
Table S36: relative energies S80
Figure S38: predicted minimum energy structures S81
Tables S37 – S46: Cartesian coordinates & unscaled vibrational frequencies S82 – S91
Figures S39 – S40: simulated spectra S92 – S93

$U^+(CO_2)_7$
Table S47: relative energies S94
Figure S41: predicted minimum energy structures S95
Tables S48 – S56 : Cartesian coordinates & unscaled vibrational frequencies S96 – S113
Figures S42 – S43 : simulated spectra S114 – S115

$U^+(CO_2)_8$
Table S57: relative energies S116
Figure S44: predicted minimum energy structures S117
Tables S58 – S66: Cartesian coordinates & unscaled vibrational frequencies S118 – S135
Figures S45 – S46 : simulated spectra S136 – S137

$U^+(CO_2)_9$
Table S67: relative energies S138
Figures S47: predicted minimum energy structures S139
Tables S68 – S75: Cartesian coordinates & unscaled vibrational frequencies S140 – S155
Figures S48 – S49: simulated spectra S156 – S157

UO^+
Table S76: relative energies S158
Figures S50 – S52: structural parameters & unscaled vibrational frequencies S159 – S161

$UO^+(CO_2)$
Table S77: relative energies S162
Figures S53: predicted minimum energy structures S163
Figures S54 – S60: structural parameters & unscaled vibrational frequencies S164 – S170
Figure S61 : simulated spectra S171

$UO^+(CO_2)_2$
Table S78: relative energies S172
Figure S62: predicted minimum energy structures S173
Figures S63 – S70 : structural parameters & unscaled vibrational frequencies S174 – S181
Figures S71 – S72 : simulated spectra S182 – S183

$UO^+(CO_2)_3$
Table S79: relative energies S184
Figure S73: predicted minimum energy structures S185

Figures S74 – S81: structural parameters & unscaled vibrational frequencies	S186 – S193
Figure S82: simulated spectra	S194

$UO^+(CO_2)_4$

Table S80: relative energies	S195
Figure S83: predicted minimum energy structures	S196
Tables S81 – S90 : Cartesian coordinates & unscaled vibrational frequencies	S197 – S206
Figures S84 – S85 : simulated spectra	S207 – S208

$UO^+(CO_2)_5$

Table S91: relative energies	S209
Figure S86: predicted minimum energy structures	S210
Tables S92 – S99: Cartesian coordinates & unscaled vibrational frequencies	S211 – S218
Figures S87 – S88 : simulated spectra	S219 – S220

$UO^+(CO_2)_6$

Table S100: relative energies	S221
Figure S89: predicted minimum energy structures	S222
Tables S101 – S107: Cartesian coordinates & unscaled vibrational frequencies	S223 – S229
Figures S90 – S91 : simulated spectra	S230 – S231

$UO^+(CO_2)_7$

Table S108: relative energies	S232
Figures S92: predicted minimum energy structures	S233
Tables S109 – S116: Cartesian coordinates & unscaled vibrational frequencies	S234 – S249
Figures S93 – S94 : simulated spectra	S250 – S251

$UO^+(CO_2)_8$

Table S117: relative energies	S252
Figure S95 : predicted minimum energy structures	S253
Tables S118 – S122: Cartesian coordinates & unscaled vibrational frequencies	S254 – S263
Figures S96 – S98: simulated spectra	S264 – S266

$UO^+(CO_2)_9$

Table S123: relative energies	S267
Figure S99: predicted minimum energy structures	S268
Tables S124 – S130 : Cartesian coordinates & unscaled vibrational frequencies	S269 – S282
Figures S100 – S102: simulated spectra	S283 – S285

UO^{2+}

Table S131: relative energies	S286
Figures S103 – S105: structural parameters & unscaled vibrational frequencies	S287 – S289

$UO^{2+}(CO_2)$

Table S132: relative energies	S290

Figure S106: predicted minimum energy structures S291
Figures S107 – S112: structural parameters & unscaled vibrational frequencies S292 – S297
Figures S113: simulated spectra S298

$UO^{2+}(CO_2)_2$
Table S133: relative energies S299
Figures S114: predicted minimum energy structures S300
Figure S115 – S118: structural parameters & unscaled vibrational frequencies S301 – S304
Figures S119: simulated spectra S305

$UO^{2+}(CO_2)_3$
Table S134: relative energies S306
Figure S120: predicted minimum energy structures S307
Tables S135 – S140: Cartesian coordinates & unscaled vibrational frequencies S308 – 313
Figure S121: simulated spectra S314

$UO^{2+}(CO_2)_4$
Table S141: relative energies S315
Figures S122: predicted minimum energy structures S316
Tables S142 – S149: Cartesian coordinates & unscaled vibrational frequencies S317 – S324
Figure S123 : simulated spectra S325

$UO^{2+}(CO_2)_5$
Table S150: relative energies S326
Figure S124: predicted minimum energy structures S327
Tables S151 – S153: Cartesian coordinates & unscaled vibrational frequencies S328 – S330
Figure S125: simulated spectra S331

$UO^{2+}(CO_2)_6$
Table S154: relative energies S332
Figures S126: predicted minimum energy structures S333
Tables S155 – S156: Cartesian coordinates & unscaled vibrational frequencies S334 – S335
Figures S127: simulated spectra S336

$UO^{2+}(CO_2)_7$
Table S157: relative energies S337
Figure S128: predicted minimum energy structures S338
Tables S158 – S161: Cartesian coordinates & unscaled vibrational frequencies S339 – S346
Figure S129: simulated spectra S347

$UO^{2+}(CO_2)_8$
Table S162: relative energies S348
Figure S130: predicted minimum energy structures S349
Tables S163 – S169: Cartesian coordinates & unscaled vibrational frequencies S350 – S363
Figures S131 – S132: simulated spectra S364 – S365

$UO^{2+}(CO_2)_9$
Table S170: relative energies S366
Figure S133 : predicted minimum energy structures S367
Tables S171 – S177 : Cartesian coordinates & unscaled vibrational frequencies S368 – S381
Figures S134 – S135: simulated spectra S382 – S383

Computational details;

All calculations were performed using density functional theory (DFT) with the B3LYP functional. The fully relativistic ECP60MDF Stuttgart/Cologne pseudopotential and corresponding correlation-consistent triple-ζ basis set (cc-pVTZ-pp) were used for uranium. Preliminary optimizations for many larger structures were performed using the cc-pVDZ-pp basis set before optimization with the triple-ζ basis set. Optimizations were performed with *the nosymm option, and wavefunction stability was verified using* the *stable=opt* keyword. The thresholds for energy and structure optimizations were set to "tight," and all calculations used a "superfine" integration grid. All optimized structures were confirmed to be true minima with no imaginary vibrational frequencies, and reported electronic energies are corrected for zero-point vibrational energy. Cartesian coordinates presented in this document are listed with atomic number (Z) and x, y, and z Cartesian coordinates. Listed vibrational frequencies (cm^{-1}) are unscaled with intensities (km/mol). A calculation of the vibrational frequency of CO_2 with B3LYP/cc pVTZ-pp provided a scaling factor of 0.972, with which all simulated spectra shown are scaled.

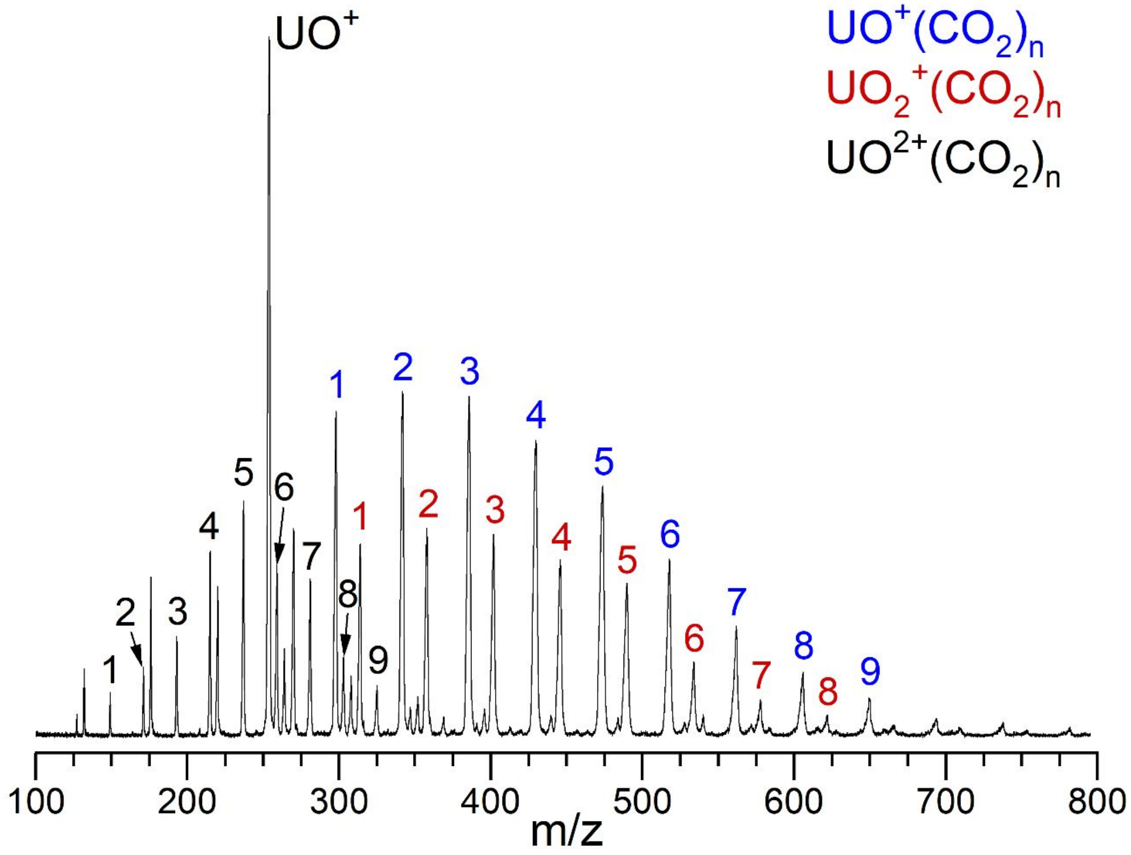

Figure S1. The mass spectrum of $U^+(CO_2)_n$ ions produced by laser vaporization of a uranium rod with an offset source configuration in a supersonic expansion of pure CO_2 gas.

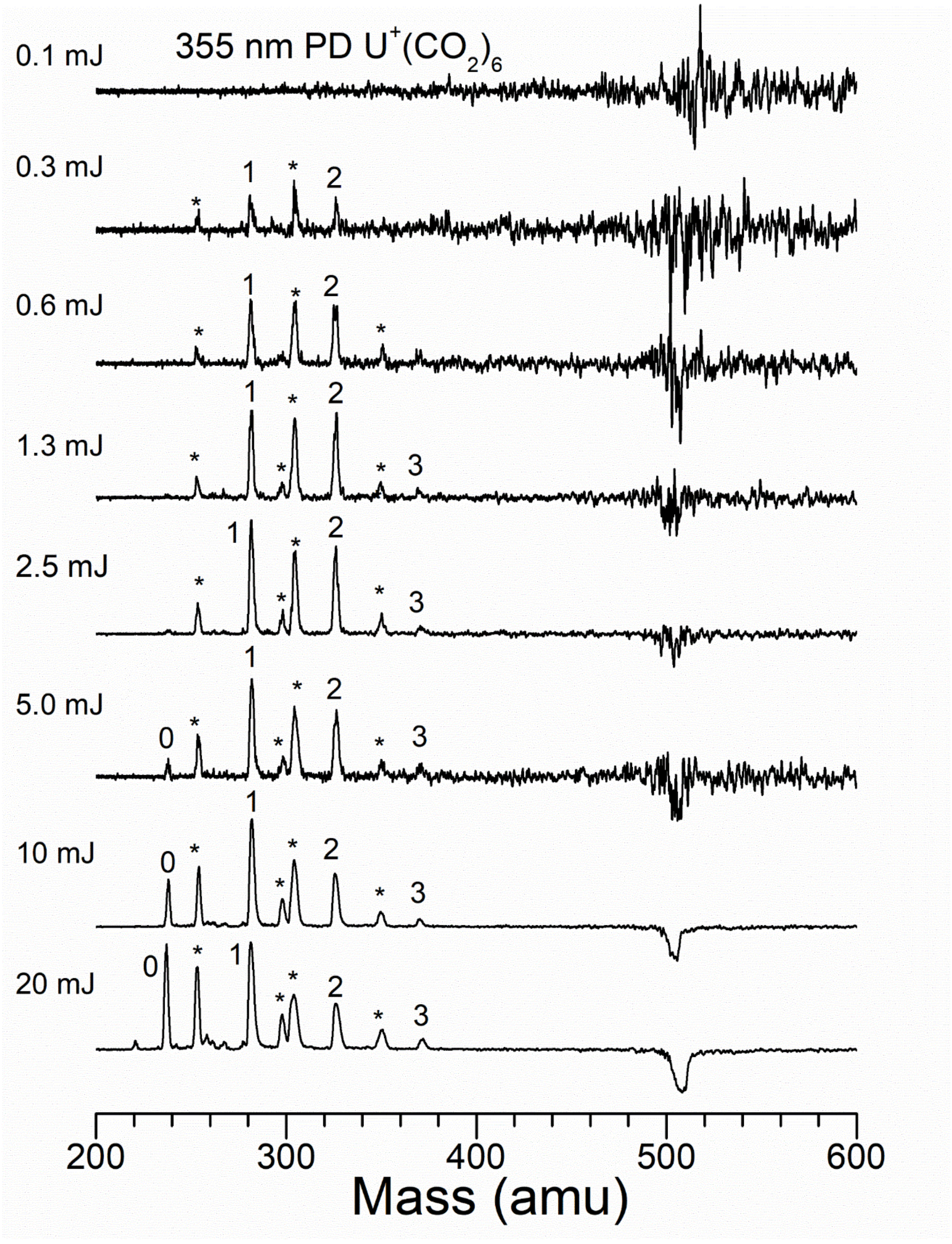

Figure S2. The photodisociation mass spectra of $U^+(CO_2)_6$ at 355 nm as a function of laser pulse energy. At 0.1 mJ energies, no fragmentation is observed; however, at energies ≥ 0.3 mJ, the complexes break apart step by step through CO_2 loss, producing fragment ions up to n = 3.

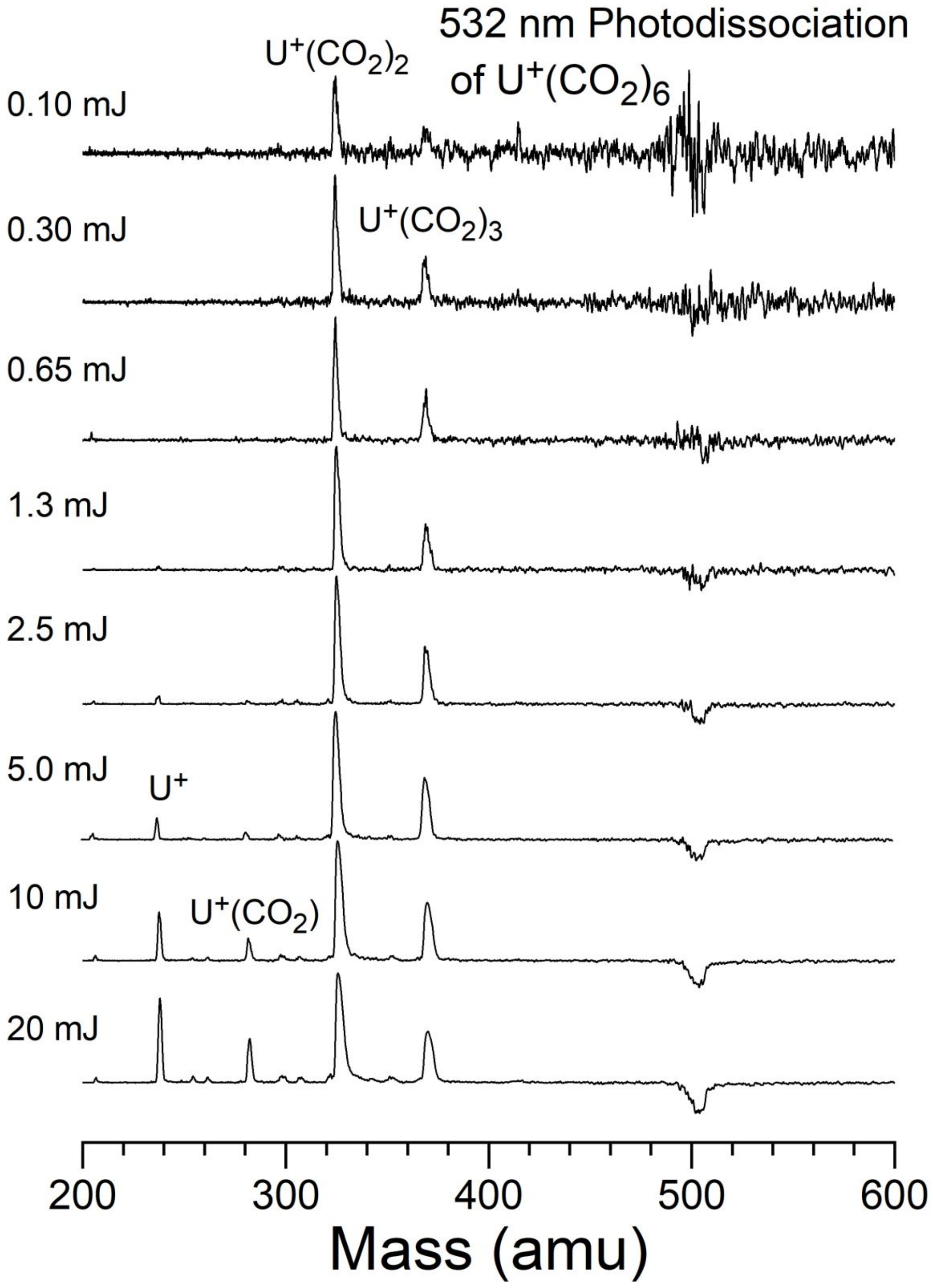

Figure S3. The photodissociation mass spectra of $U^+(CO_2)_6$ at 532nm wavelength as a function of laser pulse energy. The fragment peaks for $U^+(CO_2)_2$ and $U^+(CO_2)_3$ appear consistently across the fluence range, suggesting a single photon absorption. The U^+ peak is only observed at higher energies, indicating the need for multiphoton processes.

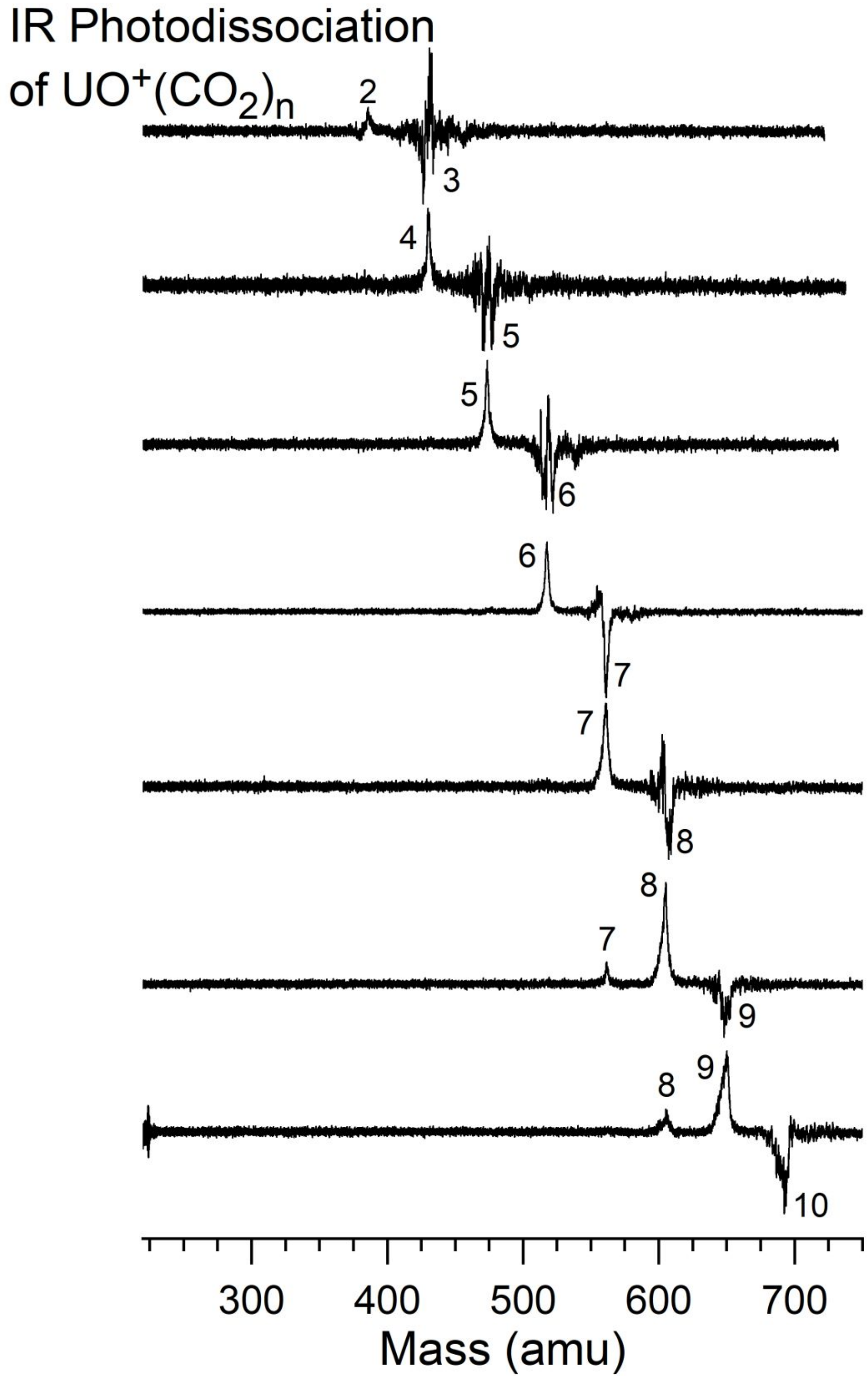

Figure S4. Infrared photodissociation mass spectra of $UO^+(CO_2)_n$. The spectra show sequential CO_2 loss with increasing cluster size, producing well-defined fragment ions throughout the series.

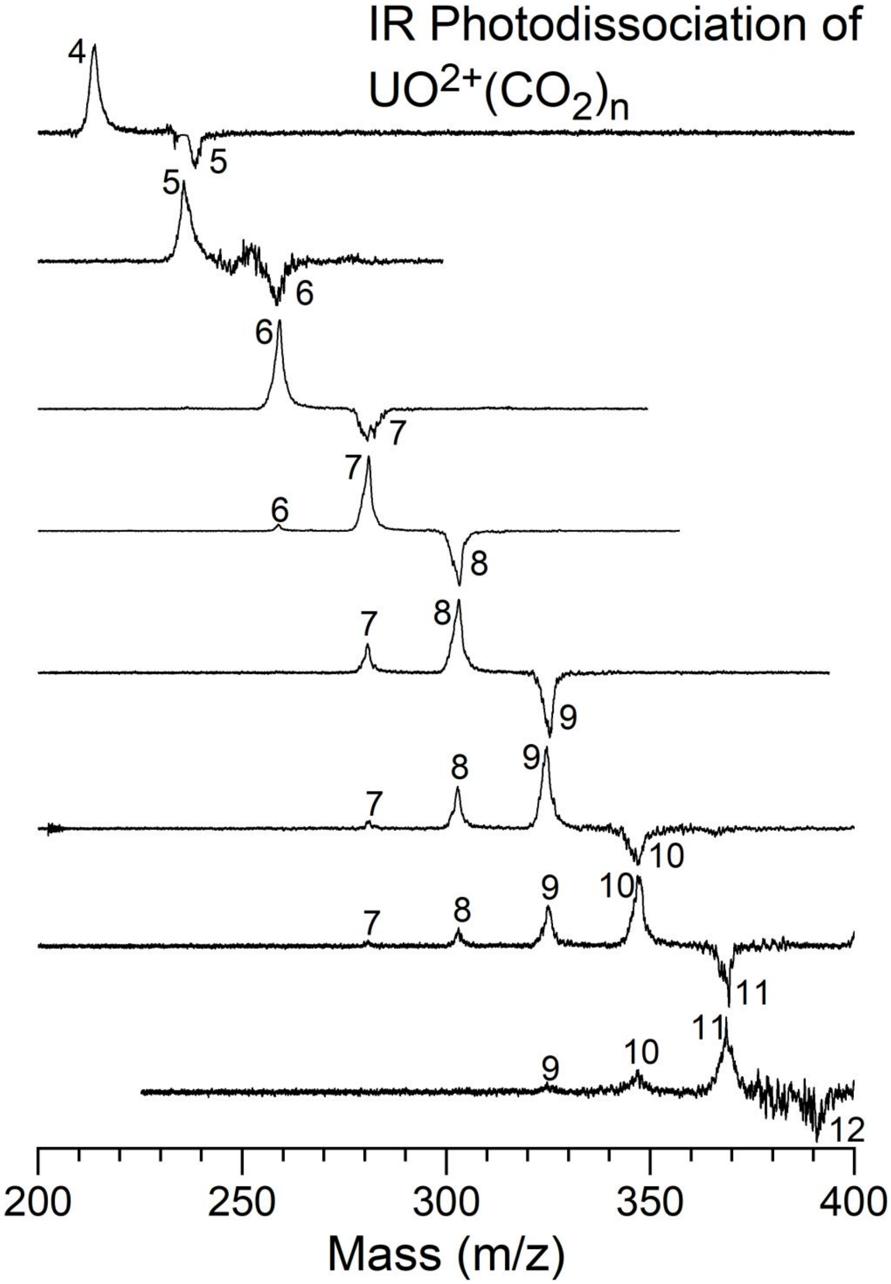

Figure S5. Infrared photodissociation mass spectra of $UO^{2+}(CO_2)_n$. The spectra show sequential CO_2 loss with increasing cluster size, producing well-defined fragment ions throughout the series.

Table S1. CO_2 electronic energy calculated at the B3LYP/cc-pVTZ(-pp) level of theory using Gaussian 16.

2s + 1	E (hartree)	Relative E (kcal/mol)
1	-188.648878	+0.0

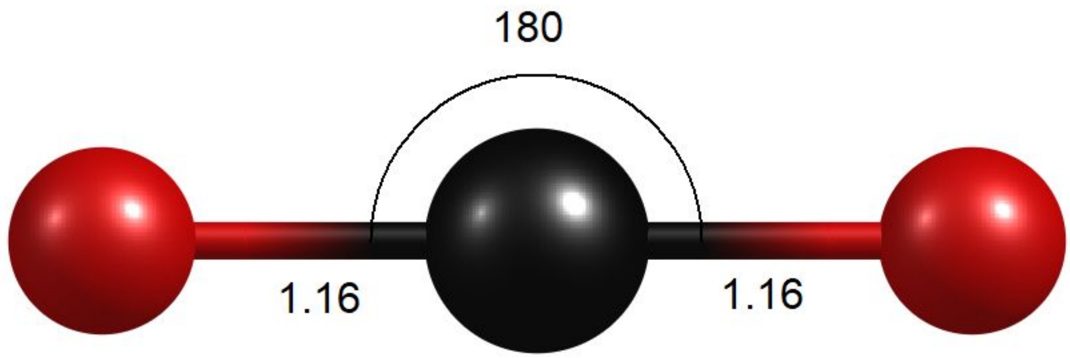

Figure S6. The optimized geometry of CO_2 followed by its predicted frequencies (cm^{-1}) and IR intensities (km/mol).

Frequency (cm^{-1})	Intensity (km/mol)
654.3235574	31.8657
654.3235574	31.8657
1335.900414	0
2353.760121	629.1408

Table S2. U^+ electronic energy calculated at the B3LYP/cc-pVTZ(-pp) level with Stuttgart/Koeln pseudopotential.

2s + 1	E (hartree)	Relative E (kcal/mol)
2	-474.4140245	+17.6
4	-474.4421108	+0.0
6	-474.4329448	+5.8

Table S3. $U^+(CO_2)$ electronic energy calculated at the B3LYP/cc-pVTZ(-pp) level with Stuttgart/Koeln pseudopotential.

Isomer	2s + 1	Energy (hartree)	Rel. E (kcal/mol)	BDE (CO_2) (kcal/mol)	BDE (CO) (kcal/mol)
1a	2	-663.173318	+13.4		21.0
1a	4	-663.194605	+0.0		18.9
1b	2	-663.085173	+68.7	14.0	
1b	4	-663.112241	+51.7	13.3	
1b	6	-663.101724	+58.3	12.5	
1c	2	-663.090934	+65.1	17.6	
1c	6	-663.103684	+57.1	13.7	
1d	2	-663.068959	+78.8		
1d	4	-663.082352	+70.4		
1d	6	-662.987231	+130.1		

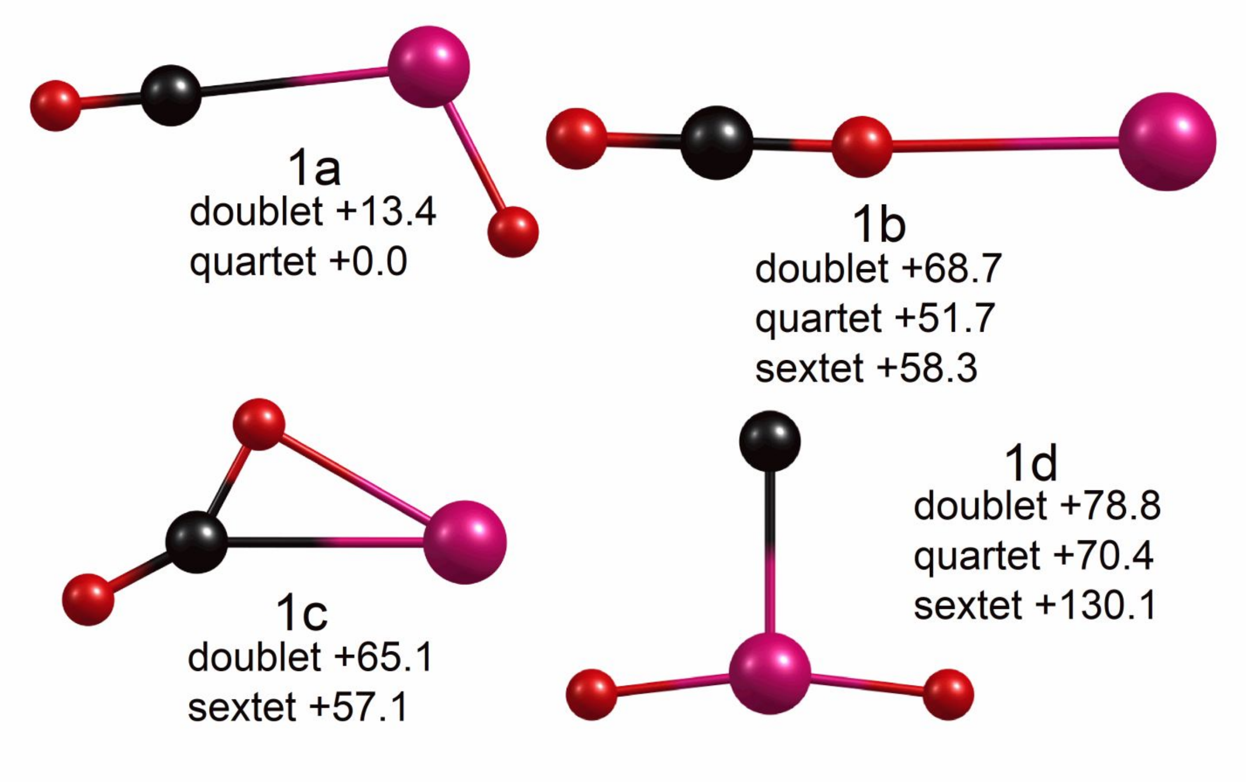

Figure S7. Predicted minimum energy structures of $U^+(CO_2)$ with energy of each spin state in kcal/mol. The lowest energy spin state of each isomer is shown.

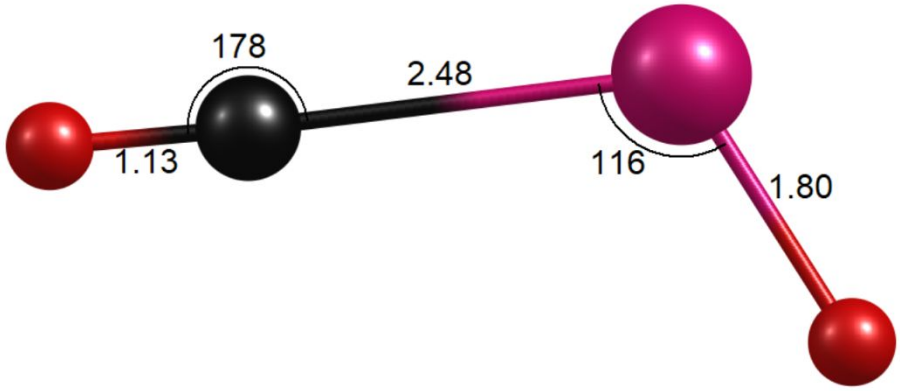

Figure S8. The optimized geometry of isomer 1a-doublet $U^+(CO_2)$ followed by its predicted frequencies (cm^{-1}) and IR intensities (km/mol).

Frequency (cm^{-1})	Intensity (km/mol)
77.8526	8.5376
219.1358	16.0665
219.4994	0.7558
265.9305	1.4767
911.0065	283.0622
2128.0909	727.6467

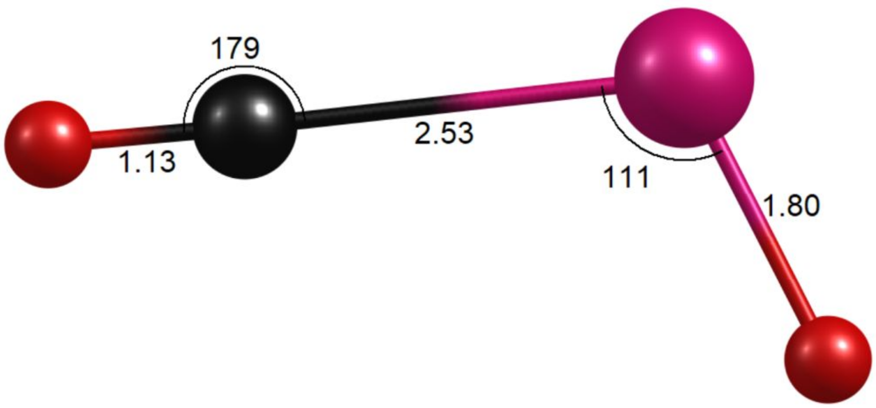

Figure S9. The optimized geometry of isomer 1a-quartet $U^+(CO_2)$ followed by its predicted frequencies (cm^{-1}) and IR intensities (km/mol).

Frequency (cm^{-1})	Intensity (km/mol)
75.45	7.4481
199.343	15.9684
213.399	0.0962
262.5551	0.4983
900.719	270.3143
2155.9052	662.6002

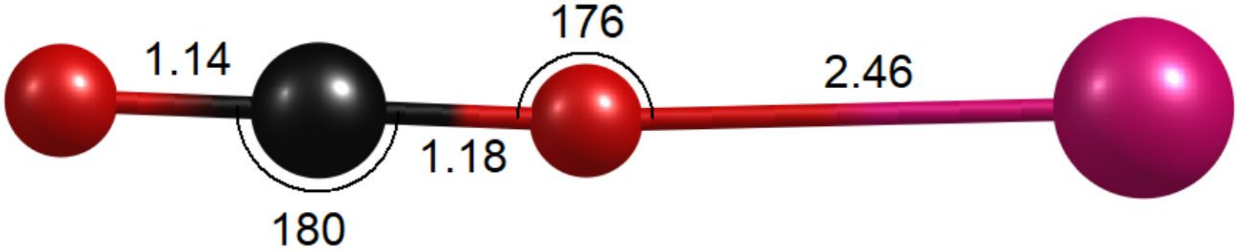

Figure S10. The optimized geometry of isomer 1b-doublet $U^+(CO_2)$ followed by its predicted frequencies (cm^{-1}) and IR intensities (km/mol).

Frequency (cm^{-1})	Intensity (km/mol)
53.1698	0.0694
165.9831	6.4979
625.6452	25.0431
625.8002	25.0318
1367.9828	73.7656
2437.7586	1114.3582

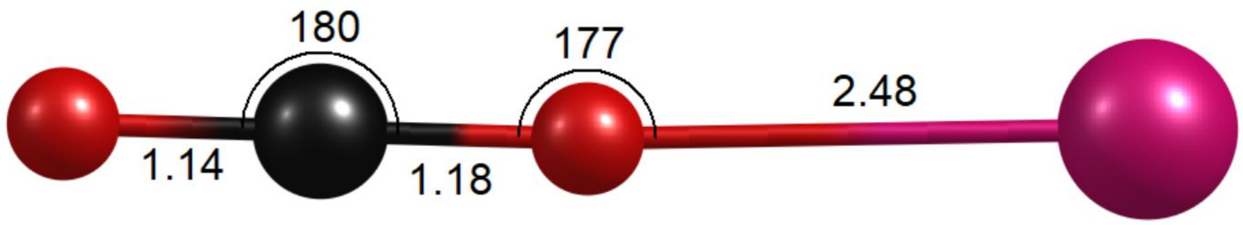

Figure S11. The optimized geometry of isomer 1b-quartet $U^+(CO_2)$ followed by its predicted frequencies (cm^{-1}) and IR intensities (km/mol).

Frequency (cm^{-1})	Intensity (km/mol)
53.587	0.086
159.4094	7.0211
626.53	24.7909
626.9961	24.8569
1366.4944	73.1142
2435.525	1102.1703

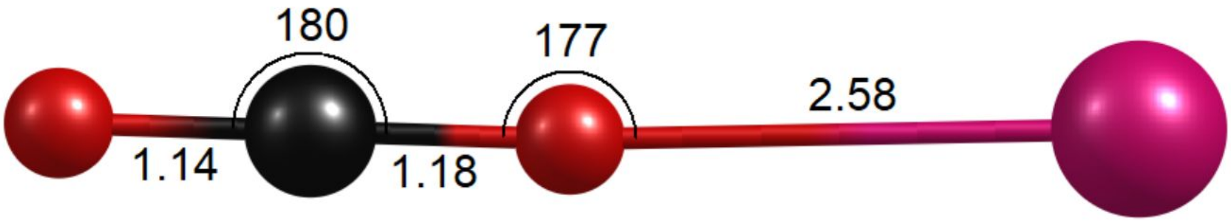

Figure S12. The optimized geometry of isomer 1b-sextet $U^{+}(CO_2)$ followed by its predicted frequencies (cm^{-1}) and IR intensities (km/mol).

Frequency (cm^{-1})	Intensity (km/mol)
59.1753	0.0051
137.8973	5.6114
641.8156	25.5042
641.8579	25.3927
1367.2801	41.1353
2431.8585	1085.0454

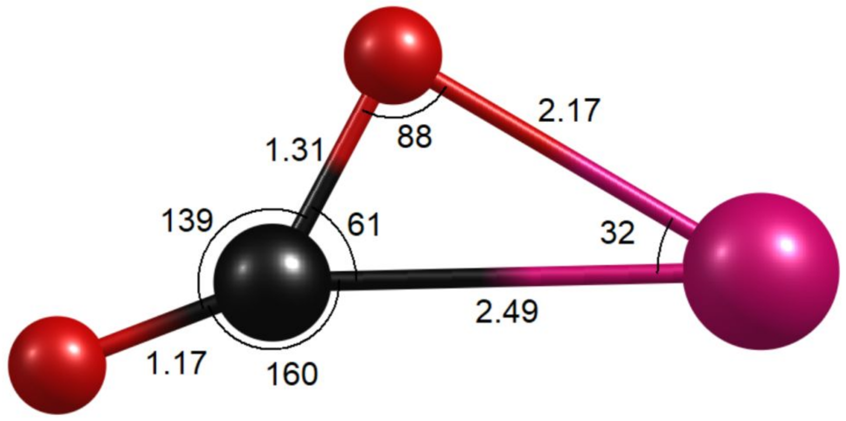

Figure S13. The optimized geometry of isomer 1c-doublet $U^+(CO_2)$ followed by its predicted frequencies (cm^{-1}) and IR intensities (km/mol).

Frequency (cm^{-1})	Intensity (km/mol)
177.3952	0.3973
302.3454	1.1365
352.3859	1.4431
717.417	93.2883
1022.566	56.5384
1909.0374	329.7311

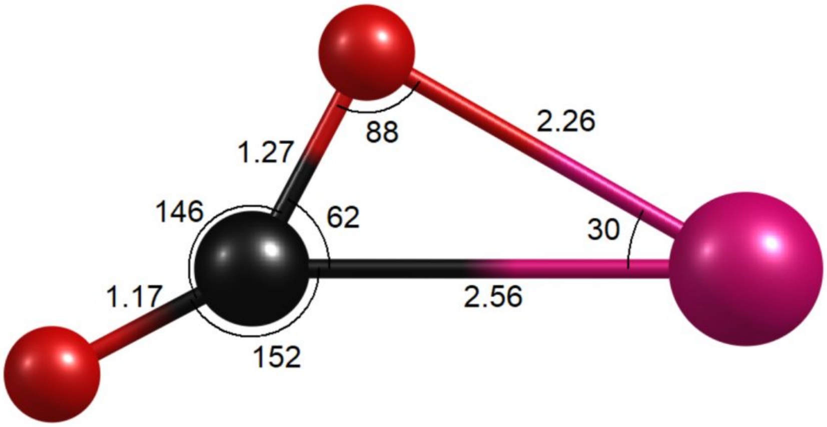

Figure S14. The optimized geometry of isomer 1c-sextet $U^+(CO_2)$ followed by its predicted frequencies (cm^{-1}) and IR intensities (km/mol).

Frequency (cm^{-1})	Intensity (km/mol)
172.0177	2.5409
332.822	2.652
386.9675	2.0444
683.9773	138.1744
1117.5749	53.1845
1980.1565	352.592

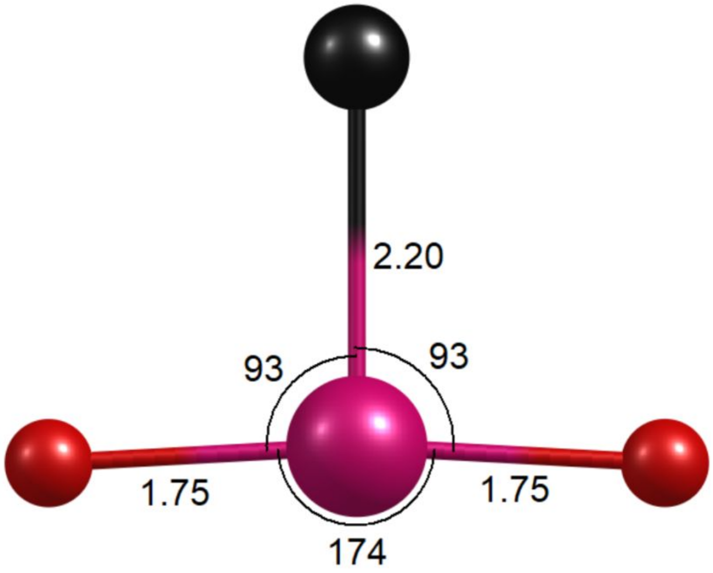

Figure S15. The optimized geometry of isomer 1d-doublet $U^+(CO_2)$ followed by its predicted frequencies (cm^{-1}) and IR intensities (km/mol).

Frequency (cm^{-1})	Intensity (km/mol)
116.5391	0.0214
166.1132	23.2992
171.8173	16.8518
449.219	0.0085
924.2669	37.2706
1009.6327	295.8941

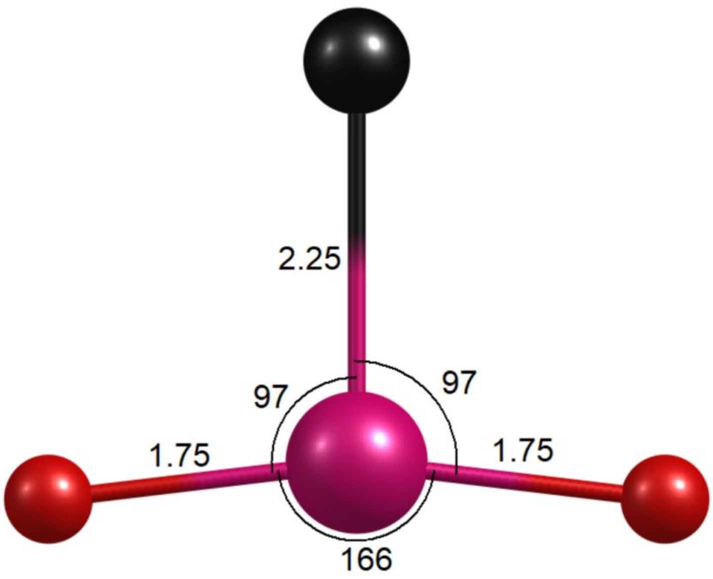

Figure S16. The optimized geometry of isomer 1d-quartet $U^+(CO_2)$ followed by its predicted frequencies (cm^{-1}) and IR intensities (km/mol).

Frequency (cm^{-1})	Intensity (km/mol)
154.1421	0.0074
160.9555	25.1148
202.3003	19.9824
505.7296	6.5362
934.0593	40.259
1010.4228	299.374

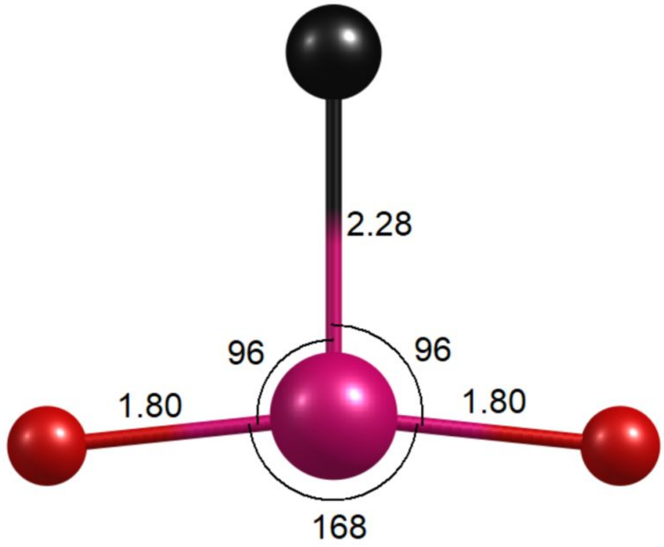

Figure S17. The optimized geometry of isomer 1d-sextet $U^+(CO_2)$ followed by its predicted frequencies (cm^{-1}) and IR intensities (km/mol).

Frequency (cm^{-1})	Intensity (km/mol)
78.6293	0.0725
144.2905	21.6926
192.8576	16.4258
532.2517	19.0014
630.2806	8.837
794.2378	18.5936

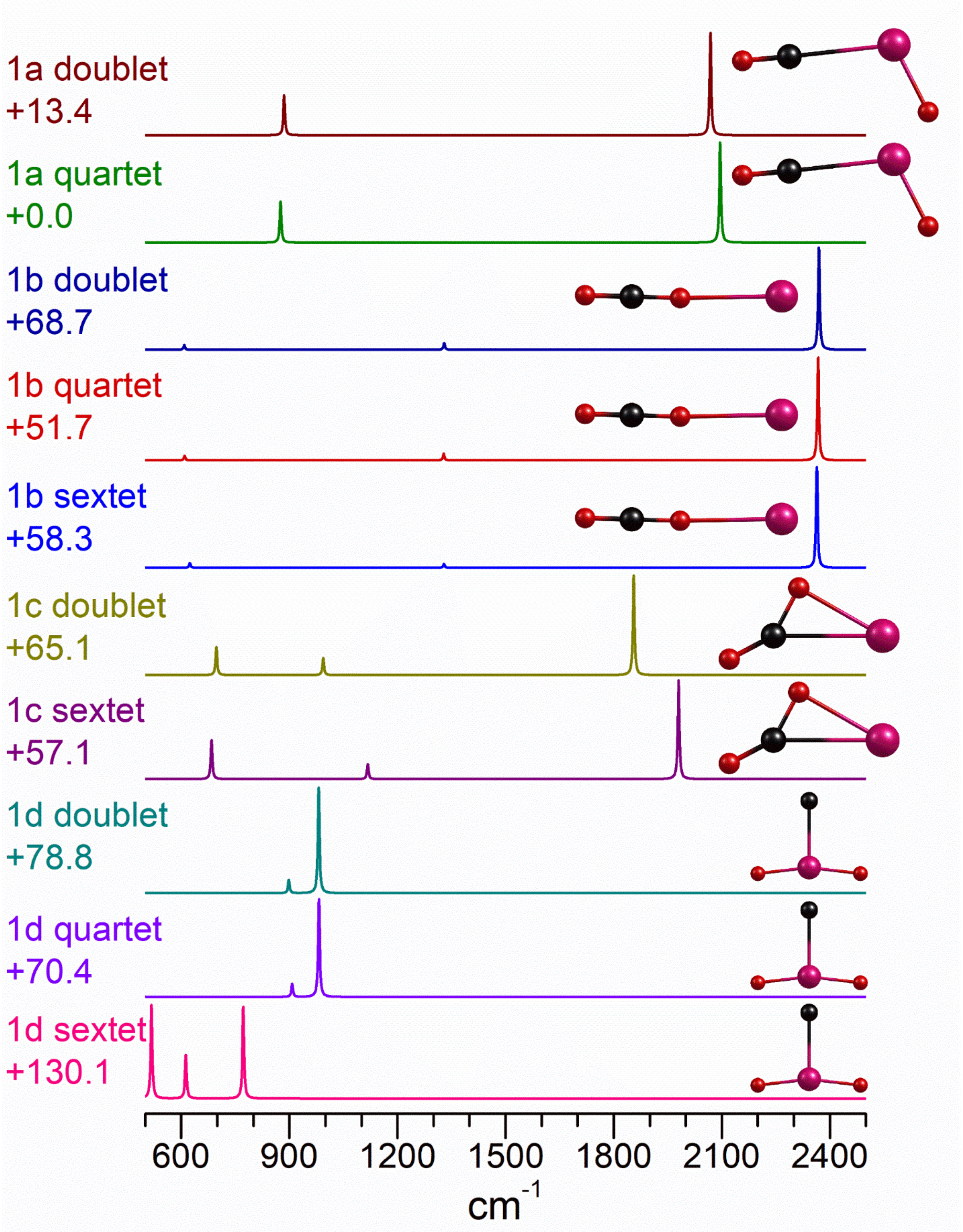

Figure S18. Simulated spectra for isomers 1a–1d in their doublet, quartet, and sextet spin states. Relative energies (kcal/mol) are shown next to each spectrum.

Table S4. $U^+(CO_2)_2$ electronic energy calculated at the B3LYP/cc-pVTZ(-pp) level with Stuttgart/Koeln pseudopotential.

Isomer	2s + 1	Energy (hartree)	Rel. E (kcal/mol)	BDE (CO_2) (kcal/mol)	BDE (CO) (kcal/mol)	BDE (oxalate) (kcal/mol)
2a	2	-851.849234	+12.9	17.0	18.7	
2a	4	-851.869871	+0.0	16.6	18.8	
2a	6	-851.738578	+82.4			
2b	2	-851.816555	+33.5	51.8		65.7
2b	4	-851.842109	+17.4	50.8		64.2
2b	6	-851.766904	+64.6	10.2		22.7
2c	2	-851.756811	+70.9	14.3		
2c	4	-851.782702	+54.7	13.5		
2c	6	-851.770647	+62.3	12.6		

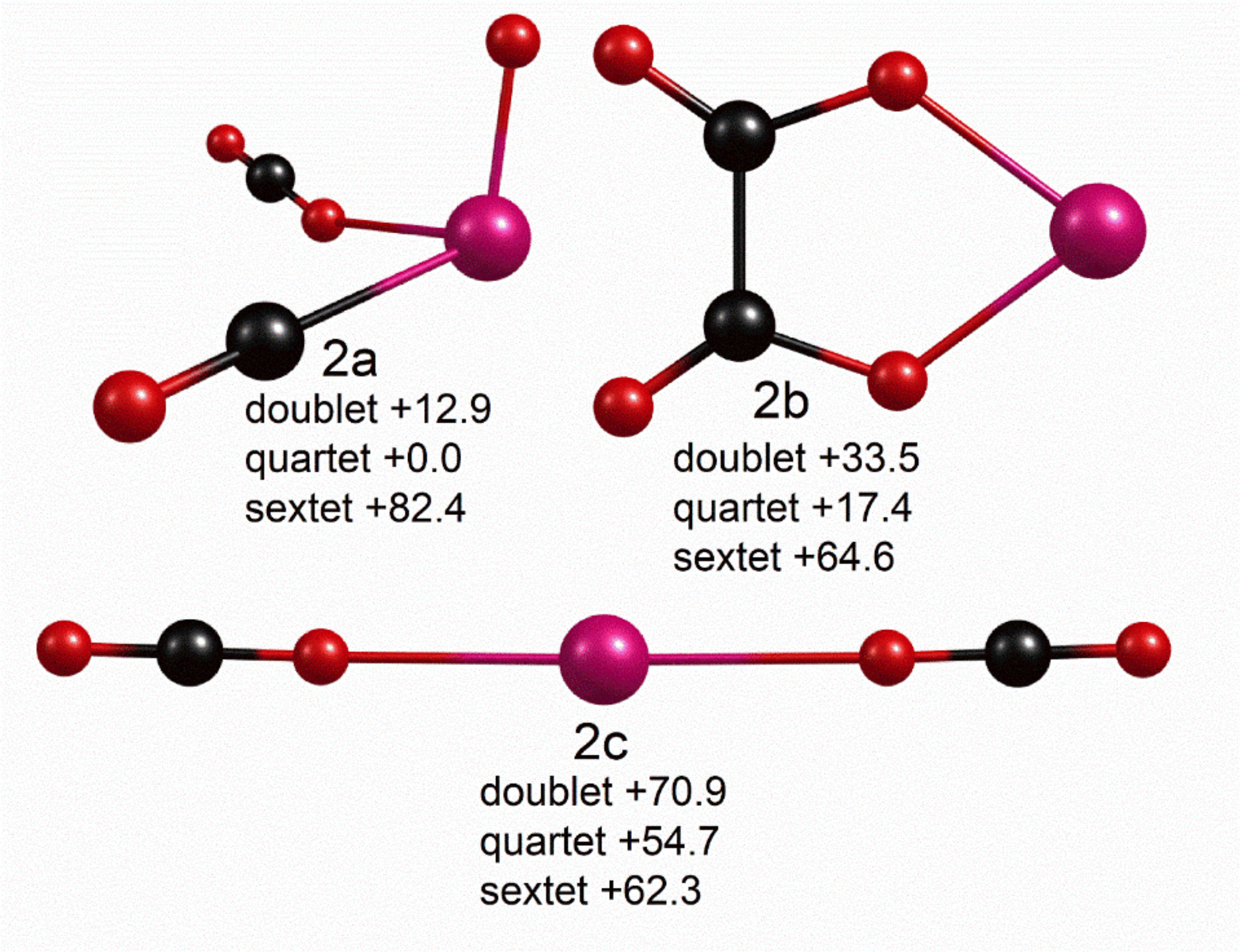

Figure S19. Predicted minimum energy structures of $U^+(CO_2)_2$ with energy of each spin state in kcal/mol. The lowest energy spin state of each isomer is shown.

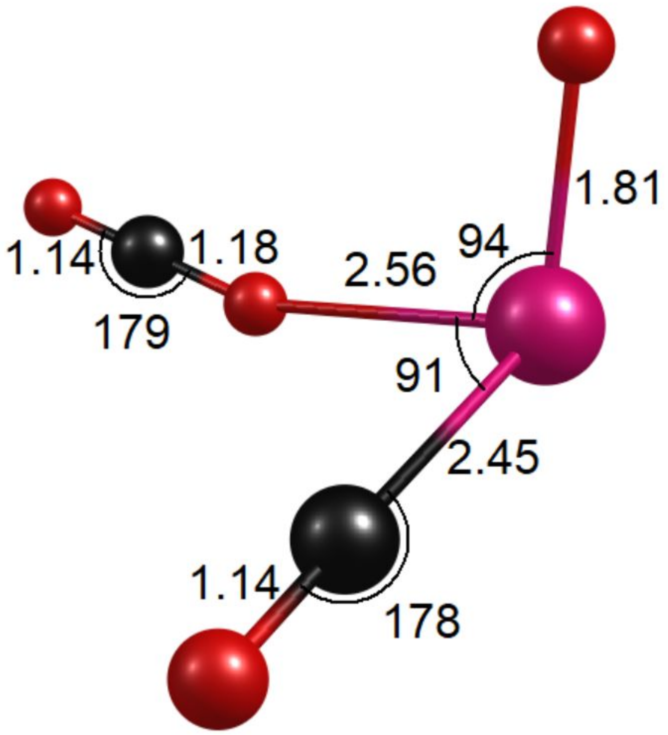

Figure S20. The optimized geometry of isomer 2a-doublet $U^+(CO_2)_2$ followed by its predicted frequencies (cm^{-1}) and IR intensities (km/mol).

Frequency (cm^{-1})	Intensity (km/mol)
23.7281	0.1868
42.0003	9.9483
71.4105	1.8572
86.2341	7.2903
118.1069	12.7338
167.6114	13.7339
233.0254	13.0593
234.5481	3.7277
270.6487	2.3938
647.0312	30.6794
649.1647	28.5862
898.4757	292.2158
1371.5245	58.607
2096.3826	808.7382
2436.8739	1024.5469

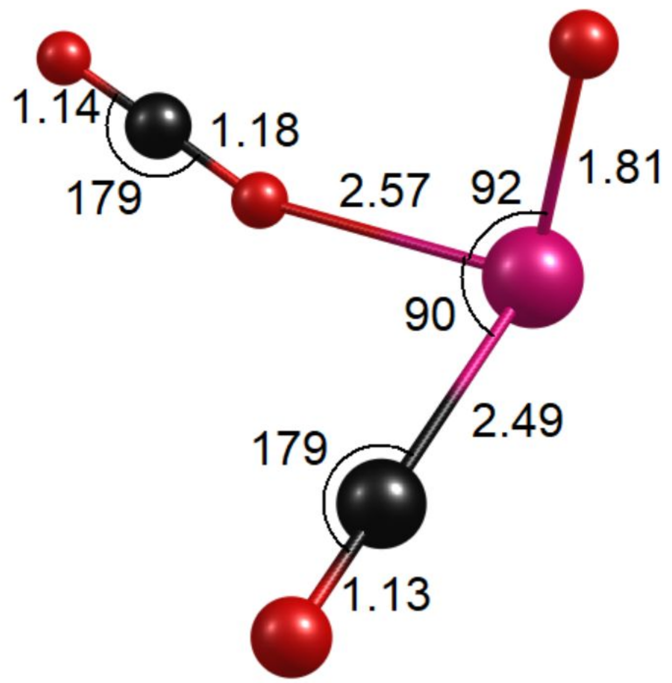

Figure S21. The optimized geometry of isomer 2a-quartet $U^+(CO_2)_2$ followed by its predicted frequencies (cm^{-1}) and IR intensities (km/mol).

Frequency (cm^{-1})	Intensity (km/mol)
23.3457	0.0686
39.7599	9.1841
70.8946	1.1715
86.7376	6.5443
124.9023	15.7436
169.6362	12.6014
216.6105	13.7923
225.5143	1.6249
269.0438	0.8072
650.1142	29.1027
651.4261	29.7508
887.1229	276.1247
1371.3883	62.3946
2125.0858	745.7381
2434.7019	1012.7189

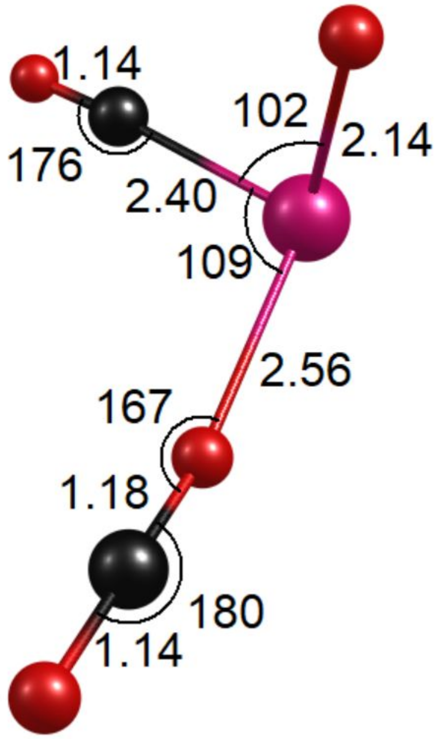

Figure S22. The optimized geometry of isomer 2a-sextet $U^{+}(CO_2)_2$ followed by its predicted frequencies (cm^{-1}) and IR intensities (km/mol).

Frequency (cm^{-1})	Intensity (km/mol)
21.1013	1.9542
35.9655	6.4447
56.0288	3.372
73.9479	1.0943
93.3685	2.7683
162.2964	10.4423
235.0139	16.0657
250.7945	10.0292
274.8091	7.279
561.5971	102.5534
642.3691	29.4715
648.6205	33.6001
1370.9506	57.3412
2020.4932	1325.3605
2437.098	1123.1621

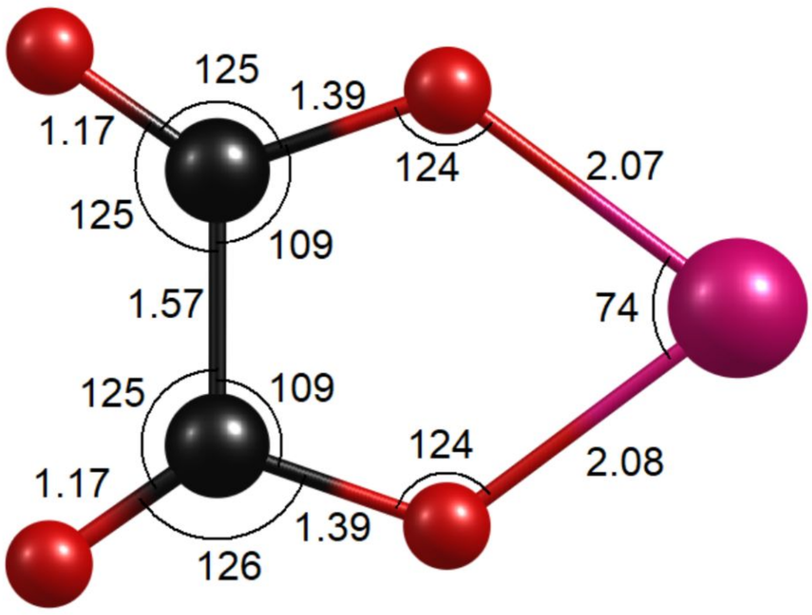

Figure S23. The optimized geometry of isomer 2b-doublet $U^+(CO_2)_2$ followed by its predicted frequencies (cm^{-1}) and IR intensities (km/mol).

Frequency (cm^{-1})	Intensity (km/mol)
83.4115	0.0001
135.0664	1.8311
314.6996	28.1685
337.8222	1.18
341.1129	17.4936
477.6396	42.3293
535.8017	2.338
575.9917	22.0904
736.1767	64.8494
808.005	0.0016
844.8773	340.3056
880.4251	63.5239
1096.9713	625.9134
1902.4151	124.4747
1910.5868	588.7953

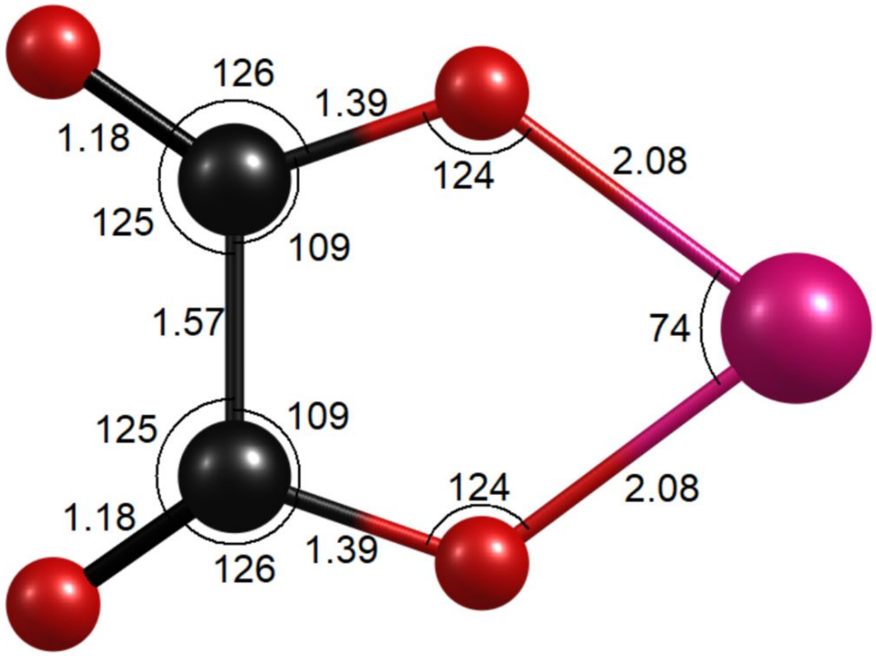

Figure S24. The optimized geometry of isomer 2b-quartet $U^+(CO_2)_2$ followed by its predicted frequencies (cm^{-1}) and IR intensities (km/mol).

Frequency (cm^{-1})	Intensity (km/mol)
82.8397	0
135.9238	1.9186
315.005	27.1296
337.2948	1.2967
340.4221	17.4323
477.3117	43.034
536.818	2.5498
576.0614	21.9036
740.4738	67.8065
808.6914	0
846.5889	319.0156
885.8088	61.625
1100.3778	615.8901
1900.6141	118.3403
1908.3804	597.8004

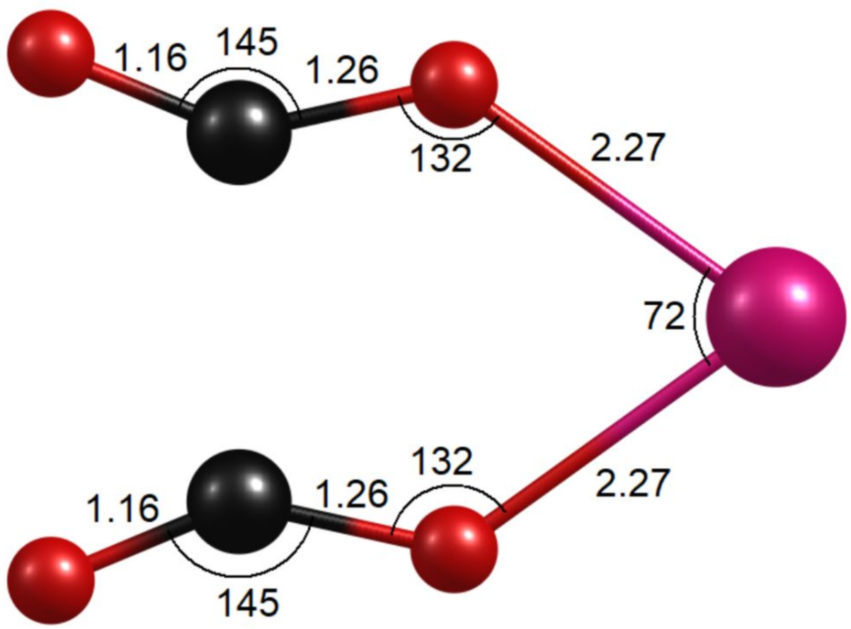

Figure S25. The optimized geometry of isomer 2b-sextet $U^+(CO_2)_2$ followed by its predicted frequencies (cm^{-1}) and IR intensities (km/mol).

Frequency (cm^{-1})	Intensity (km/mol)
74.3636	0.0061
101.5187	0.4025
182.0025	1.9176
210.3194	6.5745
234.8021	9.9304
317.6049	0.1235
358.2353	41.6428
457.7075	21.9319
635.0723	1.589
649.3119	194.9771
655.6379	29.5892
1094.1099	41.0079
1250.551	302.0656
1993.0379	137.0753
2064.3826	1176.5899

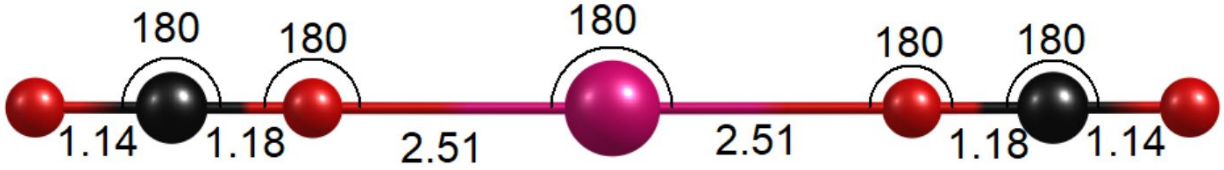

Figure S26. The optimized geometry of isomer 2c-doublet $U^+(CO_2)_2$ followed by its predicted frequencies (cm^{-1}) and IR intensities (km/mol).

Frequency (cm^{-1})	Intensity (km/mol)
15.8024	0.285
51.259	0.0178
52.7936	0.0001
78.2677	0.6296
79.8161	0.6367
157.9626	0
162.2177	16.5809
630.068	0.3077
630.1949	0.0293
630.3707	48.4825
630.4615	48.8235
1371.2403	127.9017
1374.0344	0.0003
2431.4146	2407.0423
2443.4416	0.0078

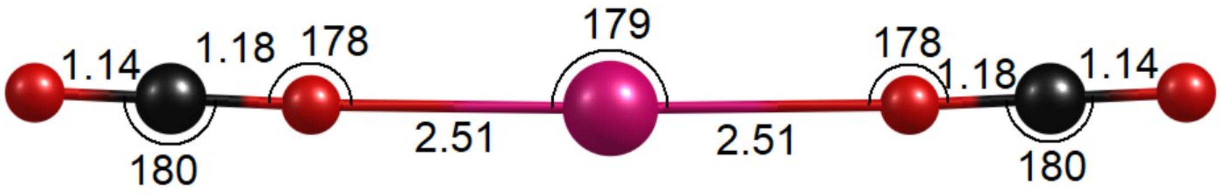

Figure S27. The optimized geometry of isomer 2c-quartet $U^+(CO_2)_2$ followed by its predicted frequencies (cm^{-1}) and IR intensities (km/mol).

Frequency (cm^{-1})	Intensity (km/mol)
15.2319	0.2771
50.159	0.0032
52.5541	0.0036
76.9294	0.6894
76.9921	0.6734
157.2645	0.0049
159.8943	16.8288
629.2755	6.3744
629.3376	3.1387
629.5557	44.5195
629.5871	41.7787
1370.6409	130.6759
1373.7915	0.1075
2430.7959	2388.2419
2443.0824	2.5812

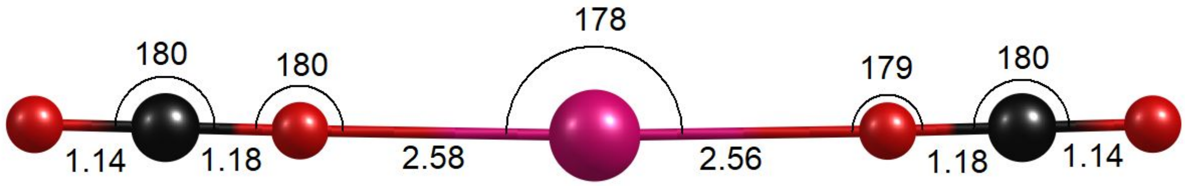

Figure S28. The optimized geometry of isomer 2c-sextet $U^+(CO_2)_2$ followed by its predicted frequencies (cm^{-1}) and IR intensities (km/mol).

Frequency (cm^{-1})	Intensity (km/mol)
3.7009	0.1176
49.3685	0.0009
50.1519	0.0037
69.9406	0.2283
70.5826	0.1623
135.4194	0.1119
149.8133	8.0684
639.853	23.7641
639.9455	32.9324
640.0934	22.4571
640.2401	14.7659
1374.3912	109.4414
1376.2436	0.1952
2433.3966	2609.9471
2440.8698	2.2489

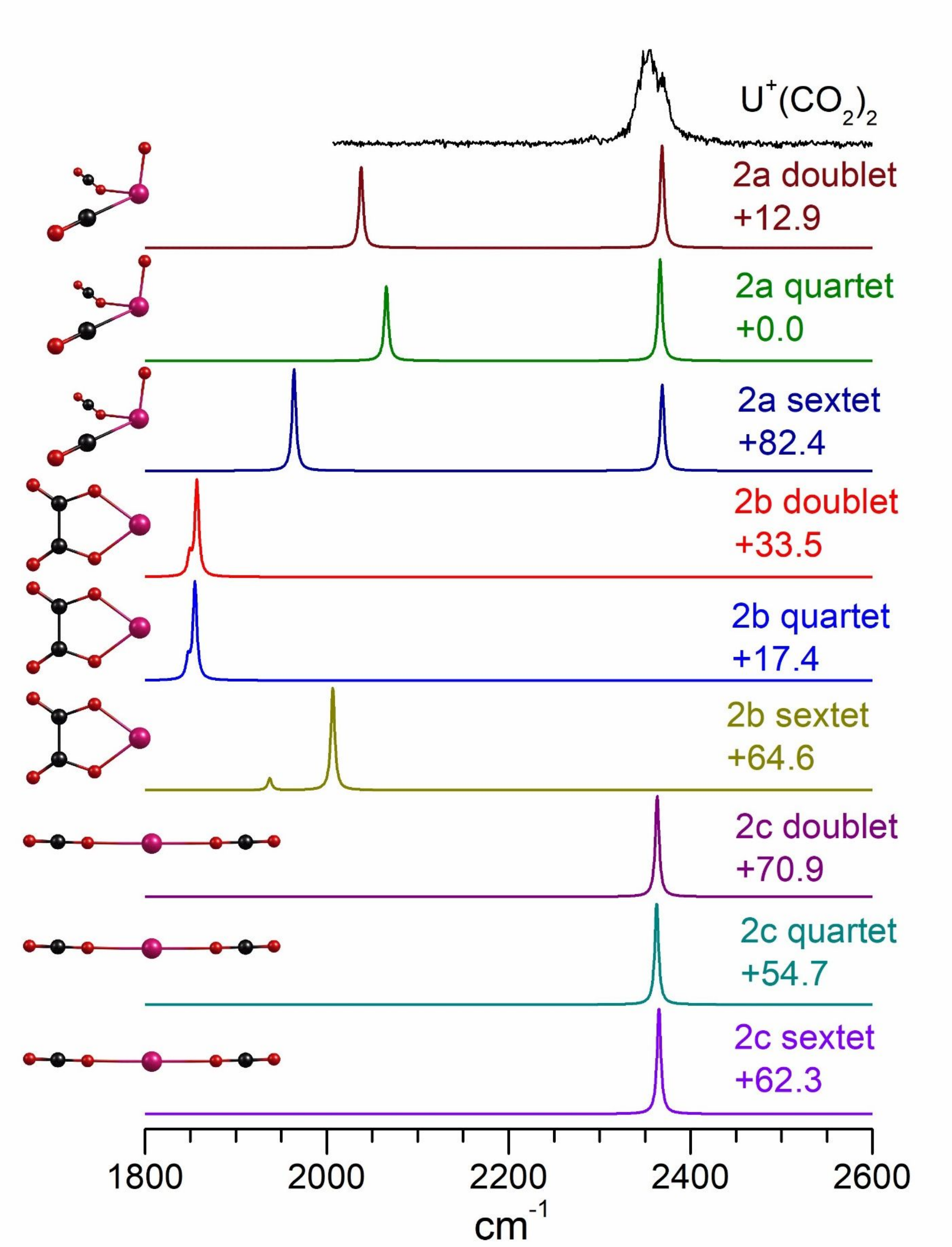

Figure S29. Experimental IR spectrum of $U^+(CO_2)_2$ compared with simulated spectra for isomers 2a, 2b and 2c. Relative energies (kcal/mol) are shown next to each spectrum.

Table S5. $U^+(CO_2)_3$ electronic energy calculated at the B3LYP/cc-pVTZ(-pp) level with Stuttgart/Koeln pseudopotential.

Isomer	2s + 1	Energy (hartree)	Rel. E (kcal/mol)	BDE (CO_2) (kcal/mol)	BDE (CO) (kcal/mol)	BDE (oxalate) (kcal/mol)
3a	2	-1040.525156	+10.6	17.0	22.0	
3a	4	-1040.542000	+0.0	14.6	18.2	
3a	6	-1040.410814	+82.3	14.7		
3b	2	-1040.496170	+28.8	19.3		71.1
3b	4	-1040.521616	+12.8	19.2		70.0
3b	6	-1040.439191	+64.5	14.7		24.9
3c	2	-1040.414586	+80.0	5.6		
3c	4	-1040.440537	+63.7	5.6		
3c	6	-1040.431470	+69.4	7.5		

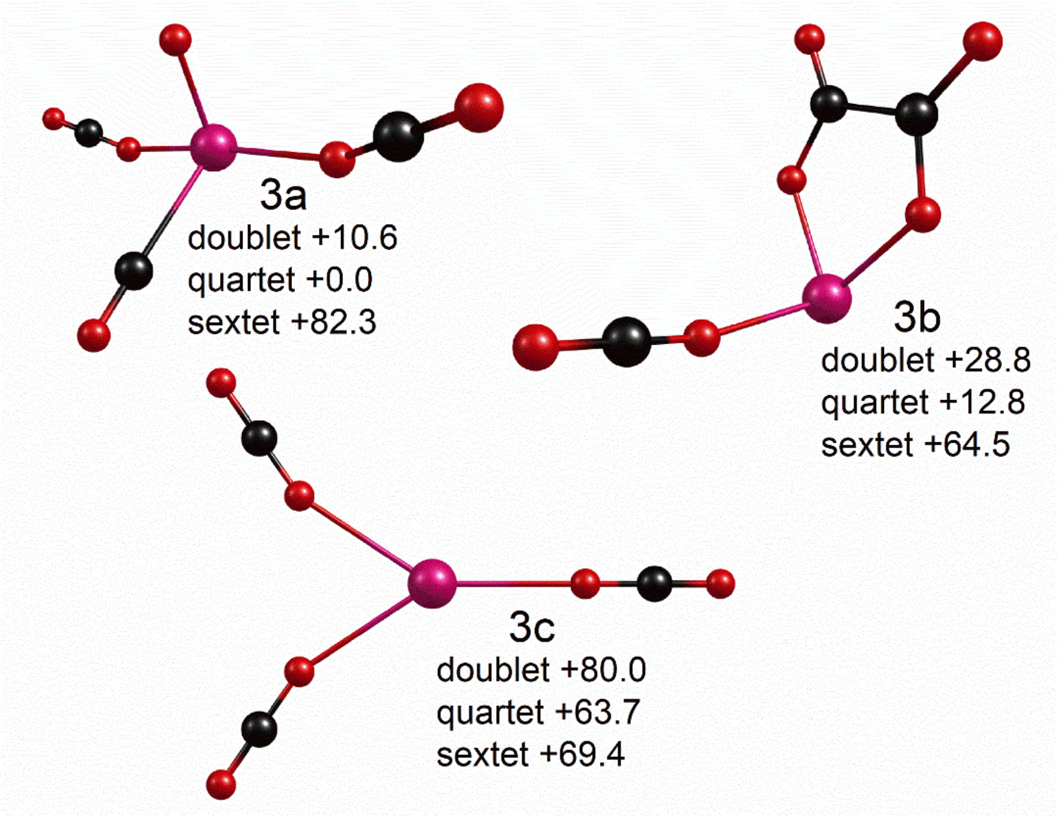

Figure S30. Predicted minimum energy structures of $U^{+}(CO_2)_3$ with energy of each spin state in kcal/mol. The lowest energy spin state of each isomer is shown.

Table S6. Cartesian coordinates for the optimized geometry of isomer 3a-doublet $U^+(CO_2)_3$ followed by its predicted frequencies (cm^{-1}) and IR intensities (km/mol).

Z	x	y	z
92	0.000081000	0.240651000	0.206114000
6	-0.000733000	-2.177634000	-0.147375000
8	-0.001125000	-3.305531000	-0.312477000
8	0.000133000	1.275365000	-1.282623000
6	3.708136000	0.164511000	-0.129035000
8	2.593897000	0.093067000	0.244102000
8	4.793926000	0.230836000	-0.479750000
8	-2.593739000	0.093319000	0.244301000
6	-3.707896000	0.165032000	-0.128977000
8	-4.793647000	0.231527000	-0.479821000

Frequency (cm^{-1})	Intensity (km/mol)	Frequency (cm^{-1})	Intensity (km/mol)
14.0705	0.239	245.4459	5.0136
15.0208	0.2704	273.3369	2.5204
27.4891	0.0688	651.301	4.692
45.4723	6.0201	651.4925	49.5445
70.3293	1.245	652.5944	3.5055
72.4878	0.1214	652.9339	58.2842
86.4785	0.1447	889.5951	270.9336
93.498	9.1478	1372.1295	111.04
142.1708	13.5386	1373.9292	5.1402
154.4174	0.3889	2060.9196	859.6375
163.6619	35.234	2429.0633	2048.7982
232.4142	7.0847	2440.055	106.7328

Table S7. Cartesian coordinates for the optimized geometry of isomer 3a-quartet $U^{+}(CO_2)_3$ followed by its predicted frequencies (cm^{-1}) and IR intensities (km/mol).

Z	x	y	z
92	-0.000018000	0.214340000	0.218163000
6	0.000090000	-2.222862000	-0.175931000
8	0.000152000	-3.347050000	-0.331571000
8	-0.000250000	1.201405000	-1.310895000
6	3.707232000	0.247866000	-0.142043000
8	2.605976000	0.123302000	0.254070000
8	4.781598000	0.364882000	-0.514869000
8	-2.606001000	0.123138000	0.254437000
6	-3.707143000	0.247740000	-0.141987000
8	-4.781406000	0.364853000	-0.515079000

Frequency (cm^{-1})	Intensity (km/mol)	Frequency (cm^{-1})	Intensity (km/mol)
14.0042	0.3288	234.3269	5.1689
14.659	0.1609	272.6624	1.1776
26.5451	0.0145	652.1959	7.3103
42.9905	6.0204	652.3682	46.9647
68.7521	0.8063	653.5405	2.3914
70.9216	0.0761	653.8688	59.1461
86.3999	0.0466	876.4582	278.3516
91.214	7	1372.0318	105.2341
143.7453	15.7181	1373.738	6.5743
154.2037	0.7987	2100.0046	784.8879
163.0865	32.6881	2428.3362	2004.9135
228.6007	12.1195	2439.5205	135.5504

Table S8. Cartesian coordinates for the optimized geometry of isomer 3a-sextet $U^+(CO_2)_3$ followed by its predicted frequencies (cm^{-1}) and IR intensities (km/mol).

Z	x	y	z
92	0.000016000	0.169899000	-0.129947000
6	-0.000077000	-2.186579000	-0.155112000
8	-0.000108000	-3.336853000	-0.141004000
8	0.000061000	0.914439000	-2.138650000
6	3.668573000	0.381754000	0.706653000
8	2.499401000	0.397014000	0.575588000
8	4.804482000	0.370989000	0.839663000
8	-2.499435000	0.396706000	0.575482000
6	-3.668601000	0.381209000	0.706560000
8	-4.804500000	0.371574000	0.839734000

Frequency (cm^{-1})	Intensity (km/mol)	Frequency (cm^{-1})	Intensity (km/mol)
14.4876	0.0416	268.177	3.2875
19.1863	0.103	283.8369	3.1708
28.9815	1.5194	534.7572	104.1951
44.5086	6.1035	643.3784	0.0198
63.436	7.9925	643.857	57.9171
67.6269	0.8808	650.1603	1.0912
75.2988	0.019	650.7886	65.897
85.2163	0.261	1370.7779	81.1946
89.1864	2.0736	1372.0976	2.2272
144.4884	1.8424	1992.9234	767.9282
154.7443	23.9676	2427.3747	2409.7561
264.3715	8.945	2439.8131	60.4562

Table S9. Cartesian coordinates for the optimized geometry of isomer 3b-doublet $U^+(CO_2)_3$ followed by its predicted frequencies (cm^{-1}) and IR intensities (km/mol).

Z	x	y	z
92	0.264124000	-0.865619000	-0.006338000
8	-1.025263000	0.210653000	-1.251308000
6	-2.045323000	1.014622000	-0.783184000
8	-2.787652000	1.639732000	-1.450570000
8	2.142835000	0.830813000	0.016676000
6	2.824540000	1.794567000	0.022098000
8	3.490549000	2.720325000	0.027735000
8	-1.061613000	0.146861000	1.245628000
6	-2.069730000	0.974780000	0.787633000
8	-2.828392000	1.568253000	1.464822000

Frequency (cm^{-1})	Intensity (km/mol)	Frequency (cm^{-1})	Intensity (km/mol)
23.4153	2.4665	569.4394	31.1087
26.5342	0.0278	644.7286	31.776
73.4917	0.2104	645.8538	30.9543
82.8428	0.083	752.7586	76.0516
100.9273	0.0316	813.8013	0.0172
140.5318	0.4344	847.971	315.2298
173.163	15.7839	905.2491	47.7178
310.488	29.7717	1119.054	675.5723
336.1469	3.4258	1371.3622	69.9212
336.258	16.6993	1888.6443	129.4658
479.3342	42.0786	1897.1055	643.8054
538.9172	2.0082	2439.1022	1033.6178

Table S10. Cartesian coordinates for the optimized geometry of isomer 3b-quartet $U^+(CO_2)_3$ followed by its predicted frequencies (cm^{-1}) and IR intensities (km/mol).

Z	x	y	z
92	0.268220000	-0.875950000	0.000032000
8	-1.039039000	0.177957000	-1.250824000
6	-2.046971000	0.998527000	-0.785994000
8	-2.795533000	1.612633000	-1.457128000
8	2.128233000	0.851756000	-0.000171000
6	2.788723000	1.830066000	-0.000228000
8	3.435086000	2.769739000	0.000019000
8	-1.038916000	0.178170000	1.250843000
6	-2.047445000	0.997985000	0.785973000
8	-2.795094000	1.613239000	1.457075000

Frequency (cm^{-1})	Intensity (km/mol)	Frequency (cm^{-1})	Intensity (km/mol)
23.4516	2.5724	568.935	30.2327
27.4583	0.0414	645.7515	32.1805
73.2984	0.239	646.6096	31.282
83.1869	0.0914	754.4783	79.0792
104.1763	0.0078	814.8217	0.0315
141.9846	0.4579	848.6882	298.7604
173.1464	16.1669	909.9019	48.5023
310.554	28.2373	1121.7796	658.7612
335.4942	17.5785	1371.8961	70.4689
336.0275	3.6356	1886.5538	120.7316
480.1246	42.8413	1894.6926	647.5061
539.4607	2.1459	2439.5439	1005.9267

Table S11. Cartesian coordinates for the optimized geometry of isomer 3b-sextet $U^+(CO_2)_3$ followed by its predicted frequencies (cm^{-1}) and IR intensities (km/mol).

Z	x	y	z
92	0.306999000	-0.347825000	0.004862000
8	-1.865313000	-1.144880000	-0.010245000
6	-2.966671000	-0.556831000	-0.013051000
8	-4.125270000	-0.676243000	-0.019729000
8	2.836284000	0.264125000	0.008393000
6	3.979557000	0.550396000	-0.009287000
8	5.087512000	0.827370000	-0.025954000
8	-1.133275000	1.418286000	0.007464000
6	-2.389591000	1.477196000	0.001049000
8	-3.297900000	2.208257000	0.000123000

Frequency (cm^{-1})	Intensity (km/mol)	Frequency (cm^{-1})	Intensity (km/mol)
13.0526	0.2799	456.522	19.2224
14.826	0.1294	631.8577	1.8116
67.3273	0.0453	644.1196	25.258
69.057	0.0011	645.6754	45.8872
73.3852	0.0402	647.296	178.7126
101.9545	0.0114	668.0724	134.0194
144.3481	8.7746	1109.3794	84.1689
180.0456	5.6009	1258.726	303.995
211.3813	5.5052	1370.8491	60.5028
229.157	17.873	1998.9949	104.27
314.6063	0.1939	2067.7786	1274.6946
349.0463	48.957	2434.3425	1144.7288

Table S12. Cartesian coordinates for the optimized geometry of isomer 3c-doublet $U^+(CO_2)_3$ followed by its predicted frequencies (cm^{-1}) and IR intensities (km/mol).

Z	x	y	z
92	-0.287441000	0.028494000	-0.076178000
8	-2.794236000	0.016132000	0.054813000
6	-3.969098000	0.005917000	0.138373000
8	-5.108388000	-0.004037000	0.218852000
8	1.854421000	-1.477043000	0.035392000
6	2.512559000	-2.450099000	0.087123000
8	3.150277000	-3.399499000	0.137282000
8	1.938605000	1.398308000	0.039959000
6	2.661025000	2.324518000	0.095445000
8	3.361531000	3.228210000	0.149041000

Frequency (cm^{-1})	Intensity (km/mol)	Frequency (cm^{-1})	Intensity (km/mol)
13.4631	0.2961	624.1796	21.3643
15.1479	0.1167	628.7953	0.5779
27.0946	0.0081	629.1481	42.2972
44.9139	0.2074	631.0115	11.5011
45.7634	0.0906	634.3589	18.1433
53.5299	0.0085	635.54	28.8395
60.0751	1.2039	1367.9332	41.8282
64.4468	1.4004	1370.4807	37.5078
108.0287	0.5724	1374.7132	35.352
112.4227	5.4997	2418.6551	1429.8381
138.9194	7.0503	2427.7725	2167.8796
160.5148	11.4532	2442.7169	50.5692

Table S13. Cartesian coordinates for the optimized geometry of isomer 3c-quartet $U^+(CO_2)_3$ followed by its predicted frequencies (cm^{-1}) and IR intensities (km/mol).

Z	x	y	z
92	0.286301000	0.000053000	-0.083303000
8	-1.901414000	1.432954000	0.038951000
6	-2.581657000	2.390634000	0.100534000
8	-3.239778000	3.325064000	0.160070000
8	-1.901245000	-1.433106000	0.038943000
6	-2.581322000	-2.390905000	0.100516000
8	-3.239284000	-3.325447000	0.160040000
8	2.789088000	0.000172000	0.047600000
6	3.962739000	0.000044000	0.150457000
8	5.100347000	-0.000070000	0.248750000

Frequency (cm^{-1})	Intensity (km/mol)	Frequency (cm^{-1})	Intensity (km/mol)
15.4053	0.0872	623.2666	20.1592
16.6284	0.1892	624.3076	17.5737
27.5248	0.021	626.9626	22.327
43.6692	0.0403	629.368	13.4144
44.1036	0.2577	631.2116	0.424
52.77	0.0067	632.8029	43.6913
64.3734	1.6906	1367.573	43.5001
74.3294	1.6002	1370.2439	37.9985
111.9126	0.2767	1375.7563	39.5108
112.5573	5.2619	2418.7503	1419.3662
140.3238	7.1929	2428.2794	2101.1147
162.0553	11.5988	2443.397	53.9484

Table S14. Cartesian coordinates for the optimized geometry of isomer 3c-sextet $U^+(CO_2)_3$ followed by its predicted frequencies (cm^{-1}) and IR intensities (km/mol).

Z	x	y	z
92	0.295497000	-0.000012000	0.068361000
8	-1.860509000	1.524520000	-0.040400000
6	-2.636080000	2.405242000	-0.081151000
8	-3.392624000	3.265424000	-0.120959000
8	-1.860539000	-1.524497000	-0.040406000
6	-2.636131000	-2.405201000	-0.081156000
8	-3.392701000	-3.265359000	-0.120959000
8	2.860164000	0.000034000	-0.059293000
6	4.034556000	0.000007000	-0.125040000
8	5.176238000	-0.000024000	-0.188618000

Frequency (cm^{-1})	Intensity (km/mol)	Frequency (cm^{-1})	Intensity (km/mol)
12.9483	0.011	627.3365	0.366
16.8703	0.1093	632.8976	44.7792
25.8346	0.0005	639.5861	23.7613
47.5203	0.1071	641.4893	7.6033
51.9756	0.0123	641.8551	37.0705
52.7928	0.3298	645.2192	16.9095
61.1632	0.5221	1364.0716	35.5946
73.3137	0.6989	1368.1112	11.9439
91.4473	0.1522	1375.8165	45.1003
111.9981	4.8375	2408.0765	1333.0545
128.8091	1.1151	2426.8959	2456.6433
150.7766	11.7744	2443.989	4.7422

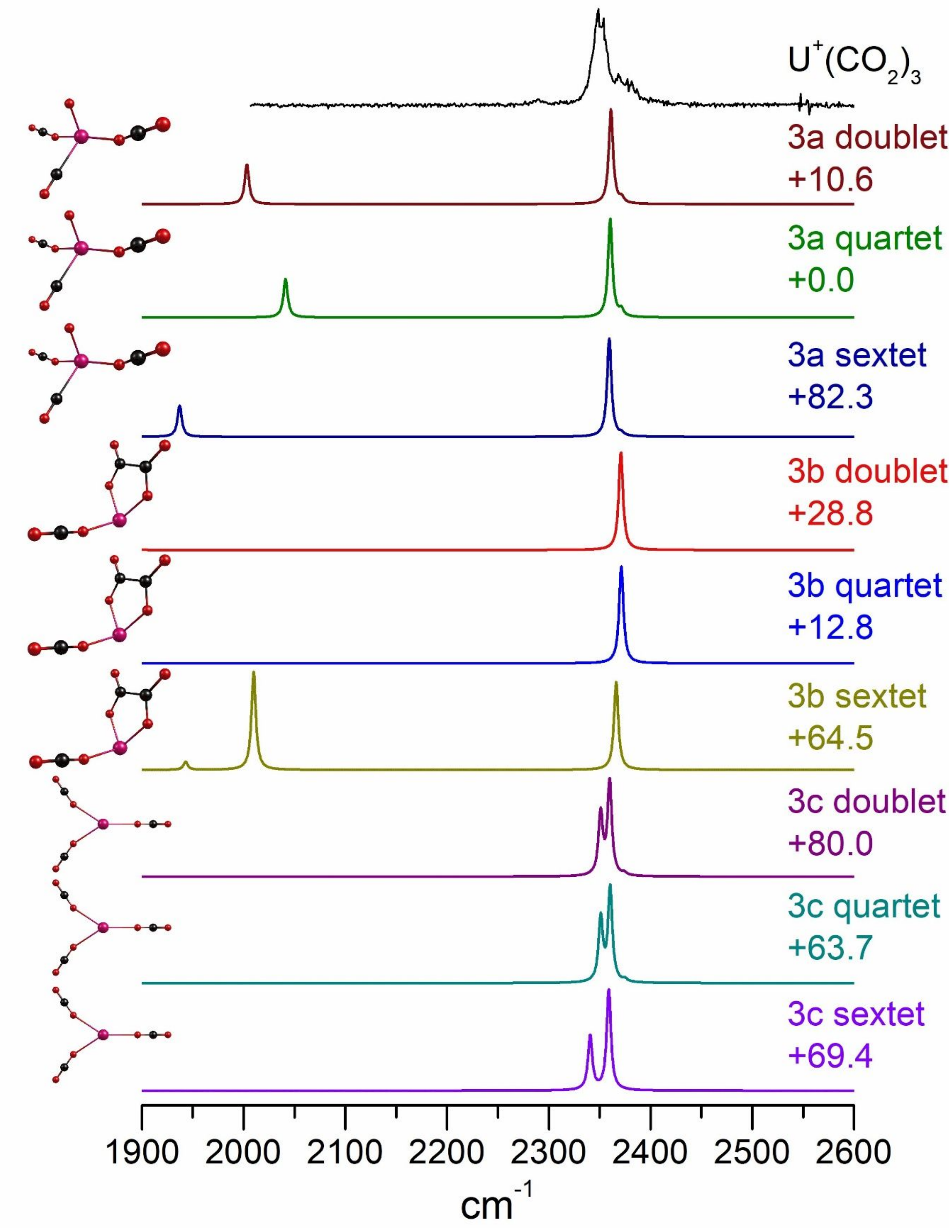

Figure S31. Figure S30. Experimental IR spectrum of $U^+(CO_2)_3$ compared with simulated spectra for isomers 3a, 3b and 3c. Relative energies (kcal/mol) are shown next to each spectrum.

Table S15. $U^+(CO_2)_4$ electronic energy calculated at the B3LYP/cc-pVTZ(-pp) level with Stuttgart/Koeln pseudopotential.

Isomer	2s + 1	Energy (hartree)	Rel. E (kcal/mol)	BDE (CO_2) (kcal/mol)	BDE (CO) (kcal/mol)	BDE (oxalate) (kcal/mol)
4a	2	-1229.192532	+10.5	11.6	18.2	
4a	4	-1229.209333	+0.0	11.6	15.9	
4a	6	-1229.077987	+82.4	11.5		
4b	2	-1229.172097	+23.4	17.0		73.8
4b	4	-1229.197338	+7.5	16.8		73.3
4b	6	-1229.076369	+83.4	-7.3		5.0
4c	2	-1229.072311	+86.0	5.6		
4c	4	-1229.097086	+70.4	4.8		
4c	6	-1229.091712	+73.8	7.1		
4d	2	-1229.065783	+90.1	1.5		
4d	4	-1229.095163	+71.6	3.6		
4d	6	-1229.088522	+75.8	5.1		

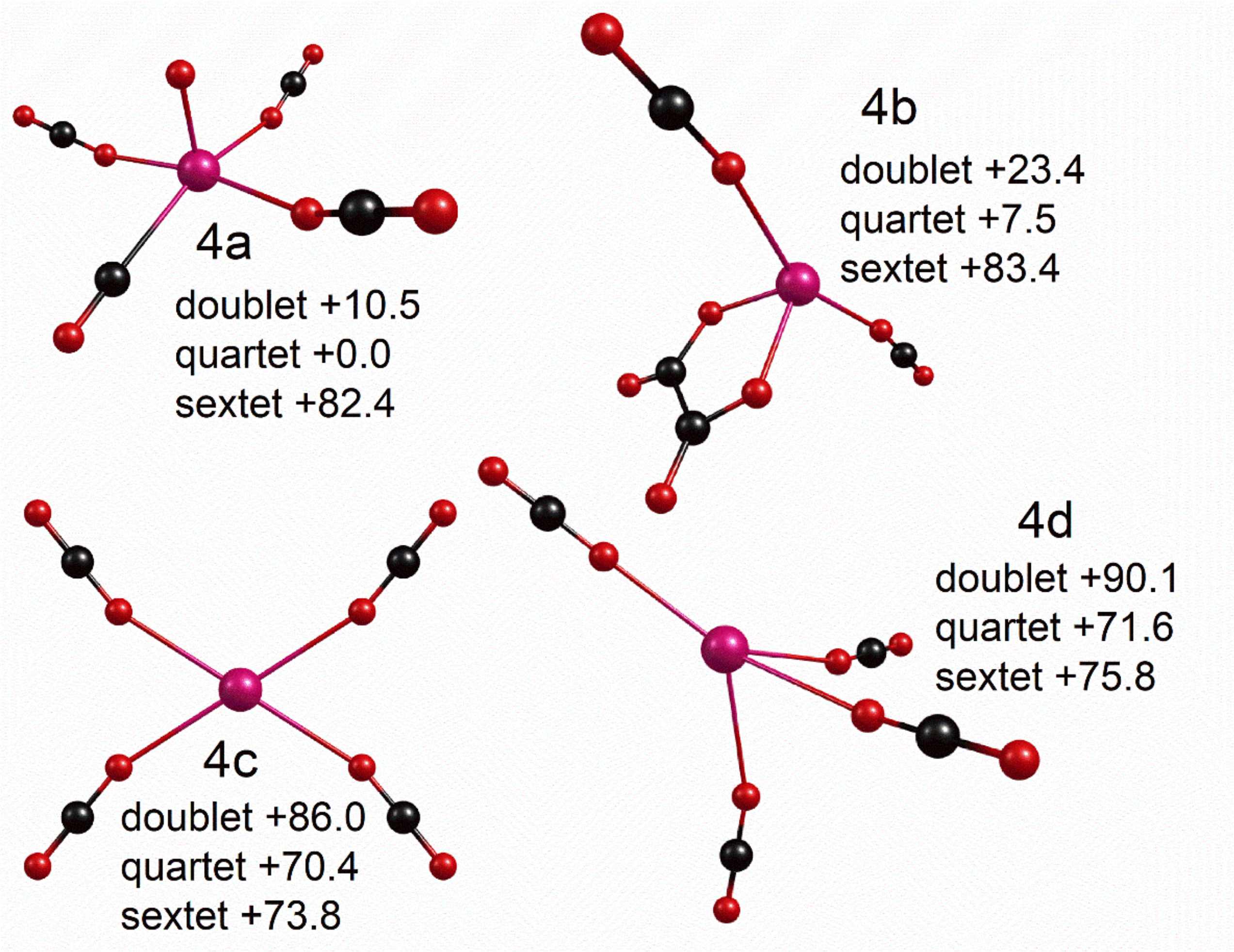

Figure S32. Predicted minimum energy structures of $U^+(CO_2)_4$ with energy of each spin state in kcal/mol. The lowest energy spin state of each isomer is shown.

Table S16. Cartesian coordinates for the optimized geometry of isomer 4a-doublet $U^+(CO_2)_4$ followed by its predicted frequencies (cm^{-1}) and IR intensities (km/mol).

Z	x	y	z
92	0.130676000	0.051649000	0.075255000
8	-2.231084000	1.176647000	0.028800000
6	-3.150725000	1.754996000	-0.419550000
8	-4.053211000	2.315900000	-0.844633000
8	-1.223035000	-2.208429000	0.596448000
6	-1.789448000	-3.223106000	0.422691000
8	-2.342673000	-4.213232000	0.265110000
8	2.700851000	-0.390915000	0.130283000
6	3.788733000	-0.513554000	-0.299257000
8	4.851413000	-0.636943000	-0.704209000
8	0.069262000	-0.307963000	-1.709030000
6	0.747115000	2.343479000	0.722255000
8	1.028945000	3.399606000	1.052199000

Frequency (cm^{-1})	Intensity (km/mol)	Frequency (cm^{-1})	Intensity (km/mol)
10.0291	0.2027	246.6533	2.7837
14.4723	0.4023	283.6258	2.2901
18.8935	0.0391	650.6323	14.427
20.2711	1.0423	651.5296	8.5187
29.3186	0.0012	652.2019	47.6229
43.1356	5.9812	653.1555	14.57
58.8404	0.8959	654.0625	50.4476
63.5812	0.9995	658.3044	36.8189
74.0111	0.1118	876.5416	275.3451
77.721	0.1431	1372.0715	44.8756
87.766	0.029	1373.388	79.1806
129.4117	12.7345	1375.2199	11.3809
137.8339	9.4645	2047.8947	1007.9347
144.5297	14.4209	2425.7374	1026.2061
150.4804	3.2667	2428.6754	1960.1183
160.5505	30.3538	2443.7623	269.6311
241.3882	6.9779		

Table S17. Cartesian coordinates for the optimized geometry of isomer 4a-quartet $U^+(CO_2)_4$ followed by its predicted frequencies (cm^{-1}) and IR intensities (km/mol).

Z	x	y	z
92	0.091807000	-0.108618000	-0.102997000
8	-2.393135000	-0.949151000	-0.055474000
6	-3.384149000	-1.355230000	0.428347000
8	-4.355881000	-1.749473000	0.887120000
8	-0.826464000	2.368873000	-0.614938000
6	-1.204007000	3.462890000	-0.413546000
8	-1.573077000	4.531306000	-0.229253000
8	2.697365000	0.160828000	-0.125762000
6	3.767250000	0.237858000	0.355677000
8	4.814138000	0.315632000	0.810914000
8	0.054340000	0.247379000	1.689882000
6	0.536724000	-2.489758000	-0.663128000
8	0.740077000	-3.568112000	-0.958533000

Frequency (cm^{-1})	Intensity (km/mol)	Frequency (cm^{-1})	Intensity (km/mol)
7.6005	0.0955	237.23	5.7505
14.0904	0.3198	279.2917	1.1891
18.5374	0.0187	651.4547	14.7853
21.4285	1.1274	652.3761	5.9058
28.5431	0.0537	652.9766	52.4504
41.0222	5.9699	653.9681	16.9428
57.9961	0.8723	654.99	47.2425
61.9097	0.7709	659.4296	35.5928
72.6783	0.0779	864.3253	276.5072
75.5831	0.1098	1372.5498	45.0101
86.9193	0.0288	1373.0403	75.372
131.392	12.89	1374.7465	11.9671
138.9843	7.339	2089.1112	931.5565
143.8818	19.2154	2425.6619	963.3457
150.7381	2.9357	2427.4431	1912.3692
159.7546	24.6117	2443.2858	348.7606
226.3875	10.2291		

Table S18. Cartesian coordinates for the optimized geometry of isomer 4a-sextet $U^+(CO_2)_4$ followed by its predicted frequencies (cm^{-1}) and IR intensities (km/mol).

Z	x	y	z
92	0.047619000	-0.237241000	0.051316000
8	-2.571884000	-0.220649000	-0.023116000
6	-3.696001000	-0.514578000	0.149937000
8	-4.794999000	-0.792659000	0.312648000
8	-0.317571000	2.393767000	-0.328797000
6	-0.483432000	3.547907000	-0.475098000
8	-0.644246000	4.672826000	-0.618879000
8	2.634058000	0.194920000	-0.049733000
6	3.792468000	0.096518000	0.122125000
8	4.922545000	0.008052000	0.282887000
8	0.059329000	-0.636898000	2.170394000
6	0.215186000	-2.410547000	-0.947874000
8	0.293984000	-3.430558000	-1.472354000

Frequency (cm^{-1})	Intensity (km/mol)	Frequency (cm^{-1})	Intensity (km/mol)
12.1341	0.5696	270.1259	3.4027
16.7962	0.0695	298.505	8.8049
21.0977	0.1394	543.4322	101.1804
21.7542	0.6295	640.9699	9.0027
33.182	0.3426	641.8154	41.0043
42.2709	4.4236	645.1403	26.8705
60.114	1.647	652.1033	9.5999
69.172	0.6388	652.9985	26.3724
72.8154	0.4922	654.0355	63.8499
75.7161	1.3464	1369.0266	23.7897
78.398	0.1587	1371.0417	58.2666
89.4738	0.5348	1372.5182	4.3993
102.9721	6.2215	1994.517	1047.4314
127.0035	4.4044	2423.9866	1108.151
143.7393	1.8517	2427.3173	2331.6018
152.0096	21.0276	2441.9942	236.3049
244.2669	4.264		

Table S19. Cartesian coordinates for the optimized geometry of isomer 4b-doublet $U^+(CO_2)_4$ followed by its predicted frequencies (cm^{-1}) and IR intensities (km/mol).

Z	x	y	z
92	-0.000017000	-0.567693000	0.004935000
8	2.519064000	-1.093692000	-0.004335000
6	3.697666000	-1.104252000	-0.007958000
8	4.839386000	-1.122116000	-0.011394000
8	0.000003000	1.111798000	-1.252109000
6	0.000062000	2.407210000	-0.788695000
8	0.000069000	3.376410000	-1.461275000
8	-2.519136000	-1.093507000	-0.004295000
6	-3.697739000	-1.103960000	-0.007888000
8	-4.839455000	-1.122093000	-0.011415000
8	0.000059000	1.123206000	1.256844000
6	0.000091000	2.413188000	0.783774000
8	0.000142000	3.389319000	1.446808000

Frequency (cm^{-1})	Intensity (km/mol)	Frequency (cm^{-1})	Intensity (km/mol)
13.3123	0.0184	563.3682	38.9743
17.3965	0.3639	647.0087	0.0015
23.0334	3.0444	647.1566	0.0209
25.7265	0.1515	647.3434	61.4733
69.8203	0.0001	647.6291	62.4837
76.7416	0.04	762.3156	83.4449
82.1204	0.184	817.8988	0.0005
84.4394	0.0891	849.9336	302.5866
93.5766	0.1505	927.0892	40.4508
136.6302	0.303	1135.7782	716.4676
152.7571	1.386	1371.3067	119.0453
170.8514	28.3982	1373.1274	1.3752
305.9195	31.8363	1878.6494	125.0809
333.1721	16.7684	1887.3508	695.5403
335.3105	5.5697	2432.0282	2190.8573
480.3849	41.7161	2442.0967	30.1682
540.8603	1.5172		

Table S20. Cartesian coordinates for the optimized geometry of isomer 4b-quartet $U^{+}(CO_2)_4$ followed by its predicted frequencies (cm^{-1}) and IR intensities (km/mol).

Z	x	y	z
92	0.000013000	0.574750000	0.000177000
8	2.531140000	1.092986000	-0.000057000
6	3.709696000	1.088282000	-0.000299000
8	4.851606000	1.091693000	-0.000534000
8	-0.000032000	-1.117448000	1.256168000
6	-0.000053000	-2.408222000	0.786466000
8	-0.000074000	-3.382487000	1.452791000
8	-2.531099000	1.093066000	-0.000069000
6	-3.709656000	1.088369000	-0.000306000
8	-4.851565000	1.091786000	-0.000533000
8	-0.000019000	-1.117235000	-1.256114000
6	-0.000045000	-2.408087000	-0.786628000
8	-0.000059000	-3.382241000	-1.4531150

Frequency (cm^{-1})	Intensity (km/mol)	Frequency (cm^{-1})	Intensity (km/mol)
13.4905	0.0174	562.6497	37.2613
17.5118	0.3567	648.0161	0.0127
22.7855	3.3237	648.3747	0.0003
31.362	0	648.6069	64.3755
69.7445	0	648.6848	62.203
77.2283	0.0166	763.0293	84.7352
82.8256	0.2994	819.2293	0
84.3765	0.0152	850.3742	283.2151
108.9824	0	932.3348	41.2797
137.7126	0.5136	1138.5342	693.7423
152.7976	1.8183	1371.8489	122.7552
170.5363	28.7382	1373.7278	1.3693
306.1772	30.1745	1876.585	120.9529
331.437	17.1066	1885.0971	696.9772
335.2211	5.6864	2433.0536	2117.8054
481.1644	42.0143	2442.7977	28.7466
541.3998	1.705		

Table S21. Cartesian coordinates for the optimized geometry of isomer 4b-sextet $U^+(CO_2)_4$ followed by its predicted frequencies (cm^{-1}) and IR intensities (km/mol).

Z	x	y	z
92	-0.000043000	-0.620486000	-0.063838000
8	2.554843000	-1.042890000	0.015574000
6	3.725037000	-0.954098000	0.117935000
8	4.860687000	-0.877226000	0.215341000
8	0.000121000	1.150979000	-1.288827000
6	0.000138000	2.338240000	-0.668063000
8	0.000298000	3.400625000	-1.439051000
8	-2.554970000	-1.042688000	0.015579000
6	-3.725160000	-0.953841000	0.117939000
8	-4.860809000	-0.876890000	0.215290000
8	0.000078000	1.015170000	1.210233000
6	0.000034000	2.371720000	0.781217000
8	0.000212000	3.306992000	1.528232000

Frequency (cm^{-1})	Intensity (km/mol)	Frequency (cm^{-1})	Intensity (km/mol)
12.4298	0.0032	566.7312	24.8849
16.2788	0.3493	612.8036	50.8672
19.5816	2.4977	634.1786	10.4262
36.3846	0.5985	648.7101	0.1609
71.2108	0.1695	649.0078	0.5762
79.6304	0.2202	649.2134	63.7629
80.7298	0.1076	649.2558	62.1779
83.9033	0.0602	799.506	325.1695
122.4703	1.9898	930.7542	94.35
151.7997	1.5123	1233.272	31.321
159.4455	1.8534	1263.3432	153.5405
165.4716	30.363	1371.4818	112.23
262.7877	12.677	1373.2405	0.9054
290.3132	3.2049	1756.7634	469.6803
307.5977	9.1217	2431.8945	2108.4954
324.8076	19.2781	2441.0974	23.9834
529.8752	2.0376		

Table S22. Cartesian coordinates for the optimized geometry of isomer 4c-doublet $U^+(CO_2)_4$ followed by its predicted frequencies (cm^{-1}) and IR intensities (km/mol).

Z	x	y	z
92	0.000003000	0.000001000	0.000004000
8	-2.190694000	1.430903000	0.007676000
6	-2.968006000	2.310965000	0.001162000
8	-3.719291000	3.176366000	-0.004189000
8	2.190670000	1.430915000	0.007720000
6	2.967961000	2.310996000	0.001249000
8	3.719216000	3.176423000	-0.004065000
8	-2.190669000	-1.430928000	-0.007709000
6	-2.967966000	-2.311003000	-0.001170000
8	-3.719240000	-3.176413000	0.004206000
8	2.190693000	-1.430904000	-0.007750000
6	2.968004000	-2.310966000	-0.001253000
8	3.719284000	-3.176372000	0.004077000

Frequency (cm^{-1})	Intensity (km/mol)	Frequency (cm^{-1})	Intensity (km/mol)
7.862	0	621.7623	0
16.7901	0.0005	623.3606	6.6981
18.7588	0.0669	624.686	75.0111
26.0378	0	625.8558	0
26.8748	0.0104	627.4766	0.7378
39.189	0	627.8451	0
42.2677	0	627.8989	0.0001
45.2452	0	629.1392	81.4162
62.4042	0.0009	1369.3419	52.5158
62.9717	2.1133	1369.8088	0
77.2763	3.1802	1371.3397	26.3609
110.2622	0	1372.526	0
115.7627	0	2417.466	0
119.2196	6.9481	2420.4937	2666.6094
122.4387	0.0485	2429.8721	2080.0353
132.1893	0	2443.1656	0
143.8335	15.1949		

Table S23. Cartesian coordinates for the optimized geometry of isomer 4c-sextet $U^+(CO_2)_4$ followed by its predicted frequencies (cm^{-1}) and IR intensities (km/mol).

Z	x	y	z
92	-0.000006000	0.000021000	-0.000003000
8	-2.160856000	-1.516007000	0.001859000
6	-2.998334000	-2.337633000	0.000247000
8	-3.814518000	-3.144054000	-0.001222000
8	-2.160884000	1.515993000	0.001878000
6	-2.998402000	2.337579000	0.000275000
8	-3.814618000	3.143967000	-0.001186000
8	2.160821000	-1.516037000	-0.001861000
6	2.998355000	-2.337606000	-0.000241000
8	3.814601000	-3.143965000	0.001236000
8	2.160873000	1.515995000	-0.001877000
6	2.998404000	2.337568000	-0.000269000
8	3.814637000	3.143938000	0.001199000

Frequency (cm^{-1})	Intensity (km/mol)	Frequency (cm^{-1})	Intensity (km/mol)
11.8742	0.0014	618.5262	0
15.2545	0.0023	619.5107	0.0031
20.6732	0.0915	621.966	0
25.9547	0	623.1815	97.2646
26.255	0	639.7942	0
42.9584	0	641.8505	68.9413
54.0235	0	642.7367	19.7658
56.3528	0.0016	644.6201	0
56.9121	0.2229	1362.8259	23.0443
75.8383	0.0405	1363.106	0.0001
81.1908	2.3417	1364.894	5.2755
92.4683	0	1365.8629	0
99.9296	0.0138	2399.2834	0
111.221	0	2418.3612	2588.8058
119.6282	4.7122	2426.8874	2496.101
126.4863	0	2445.8488	0
138.3759	8.5202		

Table S24. Cartesian coordinates for the optimized geometry of isomer 4d-doublet $U^+(CO_2)_4$ followed by its predicted frequencies (cm^{-1}) and IR intensities (km/mol).

Z	x	y	z
92	-0.503092000	0.089542000	-0.000033000
8	1.448592000	-0.950204000	-1.550811000
6	2.037085000	-1.509005000	-2.396620000
8	2.609350000	-2.056255000	-3.229358000
8	1.620986000	1.714374000	-0.000279000
6	2.268088000	2.693161000	-0.000426000
8	2.895162000	3.653593000	-0.000576000
8	1.448574000	-0.949674000	1.551145000
6	2.037056000	-1.508181000	2.397155000
8	2.609314000	-2.055139000	3.230091000
8	-3.055446000	-0.024318000	0.000036000
6	-4.230874000	-0.051876000	0.000030000
8	-5.374488000	-0.080187000	0.000029000

Frequency (cm^{-1})	Intensity (km/mol)	Frequency (cm^{-1})	Intensity (km/mol)
10.1044	0.0002	615.4328	20.422
16.0068	0.0485	618.7064	19.5077
21.3253	0.0102	624.6588	17.5083
23.312	0.0072	629.6562	22.7095
26.5781	0.0211	632.5135	17.2274
44.0695	0.1921	640.5372	16.3446
44.6919	0.2547	642.346	30.9089
50.2984	0.0326	642.4318	0.6388
50.5845	0.0829	1358.9788	2.5563
60.24	1.869	1359.867	3.6356
74.785	0.1167	1368.5497	16.1585
90.217	0.4811	1374.3509	33.1723
93.614	3.3883	2395.4377	1802.155
96.1735	0.1189	2416.1829	1395.3857
103.4861	1.6597	2427.9756	2314.0456
123.1097	2.819	2444.5229	1.0538
154.6579	10.0088		

Table S25. Cartesian coordinates for the optimized geometry of isomer 4d-quartet $U^+(CO_2)_4$ followed by its predicted frequencies (cm^{-1}) and IR intensities (km/mol).

Z	x	y	z
92	-0.504984000	-0.005176000	0.001660000
8	1.613932000	0.669294000	-1.559341000
6	2.092410000	1.094830000	-2.542938000
8	2.552666000	1.512266000	-3.507110000
8	1.616600000	1.021377000	1.353278000
6	2.093056000	1.668581000	2.208636000
8	2.551484000	2.303215000	3.046966000
8	1.621377000	-1.682670000	0.202353000
6	2.110179000	-2.742318000	0.328753000
8	2.580129000	-3.781233000	0.452714000
8	-2.999381000	-0.001843000	0.002185000
6	-4.176489000	0.000699000	-0.001529000
8	-5.318861000	0.002769000	-0.004825000

Frequency (cm^{-1})	Intensity (km/mol)	Frequency (cm^{-1})	Intensity (km/mol)
13.3829	0.0011	615.4124	12.9967
14.0739	0.0323	616.9968	17.6571
23.1862	0.0114	621.5433	0.3695
23.3699	0.001	627.0953	15.1756
28.6399	0	629.6158	18.6524
37.3934	0.7191	636.6451	0.0482
38.076	0.272	638.149	43.6732
43.9424	0.0338	639.4716	27.4707
44.2055	2.022	1367.5458	29.7316
63.3152	1.8672	1367.6506	25.4875
83.3037	3.5716	1370.1528	12.5182
88.0113	4.8428	1378.495	43.6248
91.6155	2.1285	2414.7841	1342.9123
92.3517	1.0605	2415.0387	1336.0351
108.6656	0.215	2428.9037	1980.9197
132.372	8.3125	2444.3287	95.4045
168.0432	10.8777		

Table S26. Cartesian coordinates for the optimized geometry of isomer 4d-sextet $U^{+}(CO_2)_4$ followed by its predicted frequencies (cm^{-1}) and IR intensities (km/mol).

Z	x	y	z
92	-0.513841000	-0.000028000	0.046472000
8	1.499859000	0.001233000	-1.801224000
6	2.085890000	0.001958000	-2.815698000
8	2.657339000	0.002668000	-3.812759000
8	1.560375000	1.538603000	0.866065000
6	2.160387000	2.428903000	1.336937000
8	2.744347000	3.301719000	1.800653000
8	1.560372000	-1.539811000	0.863933000
6	2.160247000	-2.430841000	1.333595000
8	2.744072000	-3.304375000	1.796128000
8	-3.076882000	0.000147000	-0.037761000
6	-4.252050000	0.000095000	-0.049775000
8	-5.396168000	0.000049000	-0.063253000

Frequency (cm^{-1})	Intensity (km/mol)	Frequency (cm^{-1})	Intensity (km/mol)
13.0842	0.0251	629.8049	19.9084
16.0295	0.0353	631.2754	3.4269
19.3335	0.0005	633.1317	29.295
22.5259	0.0046	636.7551	23.2808
26.2266	0.0211	637.1469	22.6974
41.7998	0.3929	643.9816	18.2754
43.3124	0.3368	644.3588	17.3519
46.366	0.0083	647.2954	26.3457
48.8047	0.0003	1364.1988	6.6001
63.2813	1.1807	1367.5543	14.6737
77.1795	0.0388	1369.2174	7.1258
86.558	0.2174	1376.8067	41.1139
92.9027	1.7324	2396.2633	1625.8699
96.8713	3.6589	2413.7772	1435.8605
97.3508	0.2472	2427.7328	2335.4052
120.0049	1.7934	2445.5568	0.3797
152.4943	10.8968		

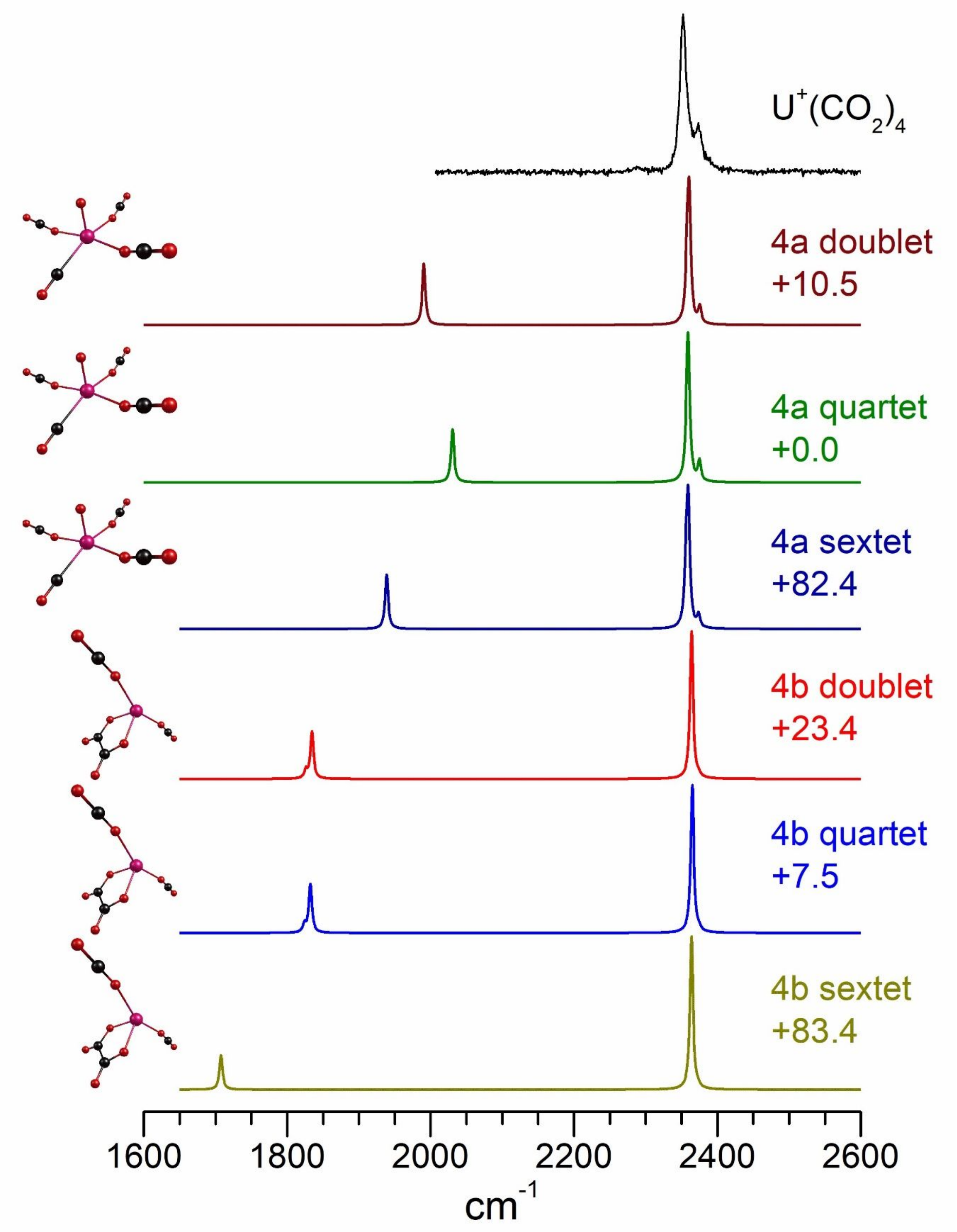

Figure S33. Experimental IR spectrum of $U^{+}(CO_2)_4$ compared with simulated spectra for isomers 4a and 4b. Relative energies (kcal/mol) are shown next to each spectrum.

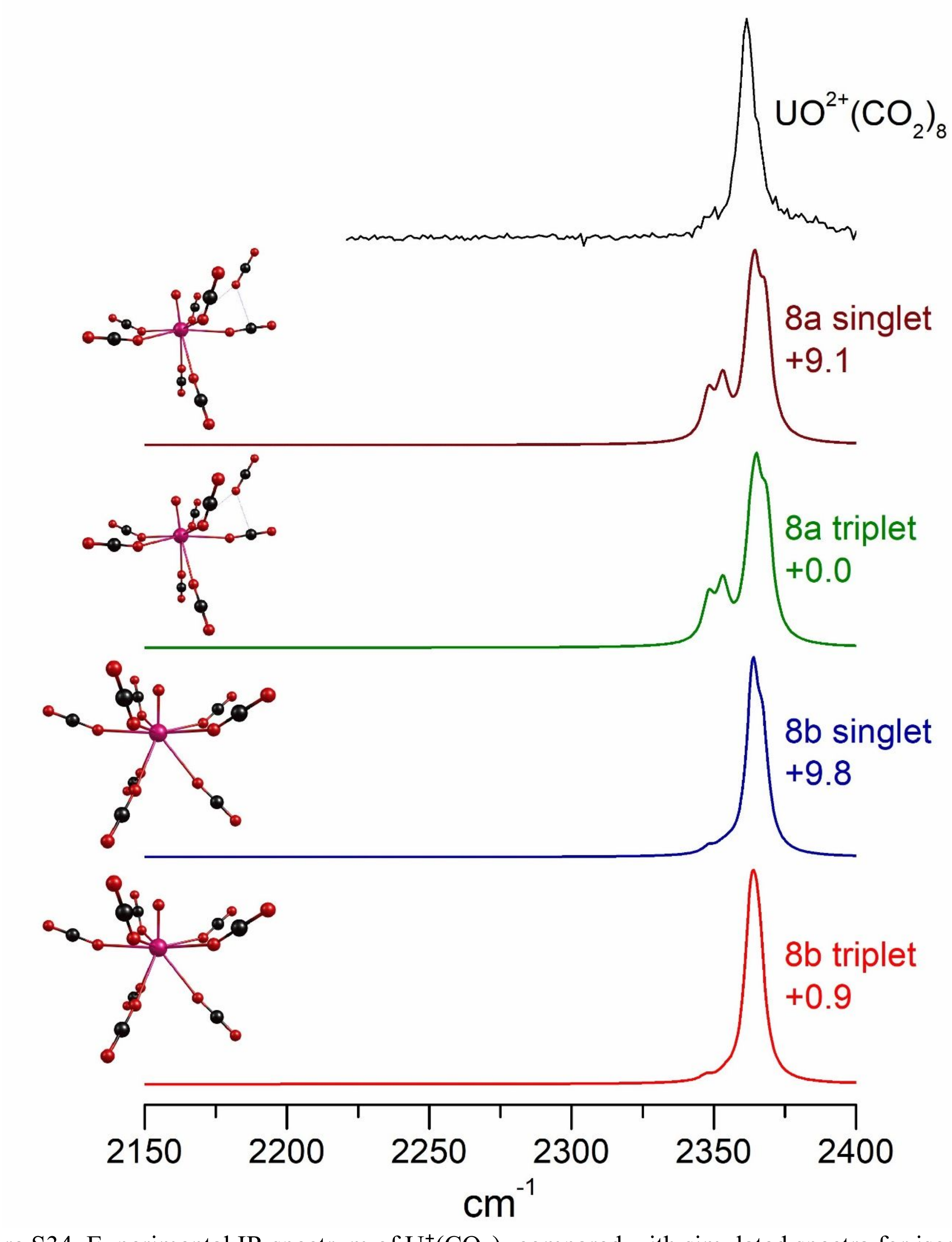

Figure S34. Experimental IR spectrum of $U^{+}(CO_2)_4$ compared with simulated spectra for isomers 4c and 4d. Relative energies (kcal/mol) are shown next to each spectrum.

Table S27. $U^+(CO_2)_5$ electronic energy calculated at the B3LYP/cc-pVTZ(-pp) level with Stuttgart/Koeln pseudopotential.

Isomer	2s + 1	Energy (hartree)	Rel. E (kcal/mol)	BDE (CO_2) (kcal/mol)	BDE (CO) (kcal/mol)	BDE (oxalate) (kcal/mol)
5a	2	-1417.855204	+10.1	8.7	17.1	
5a	4	-1417.871275	+0.0	8.2	14.2	
5a	6	-1417.748392	+77.1	13.5		
5b	2	-1417.838864	+20.3	11.2		79.4
5b	4	-1417.862848	+5.3	10.4		78.2
5b	6	-1417.742379	+80.9	10.8		8.3
5c	2	-1417.739946	+82.4	11.8		
5c	4	-1417.743713	+80.0	-1.4		
5c	6	-1417.771447	+62.6	19.4		
5d	6	-1417.746542	+78.3	5.7		

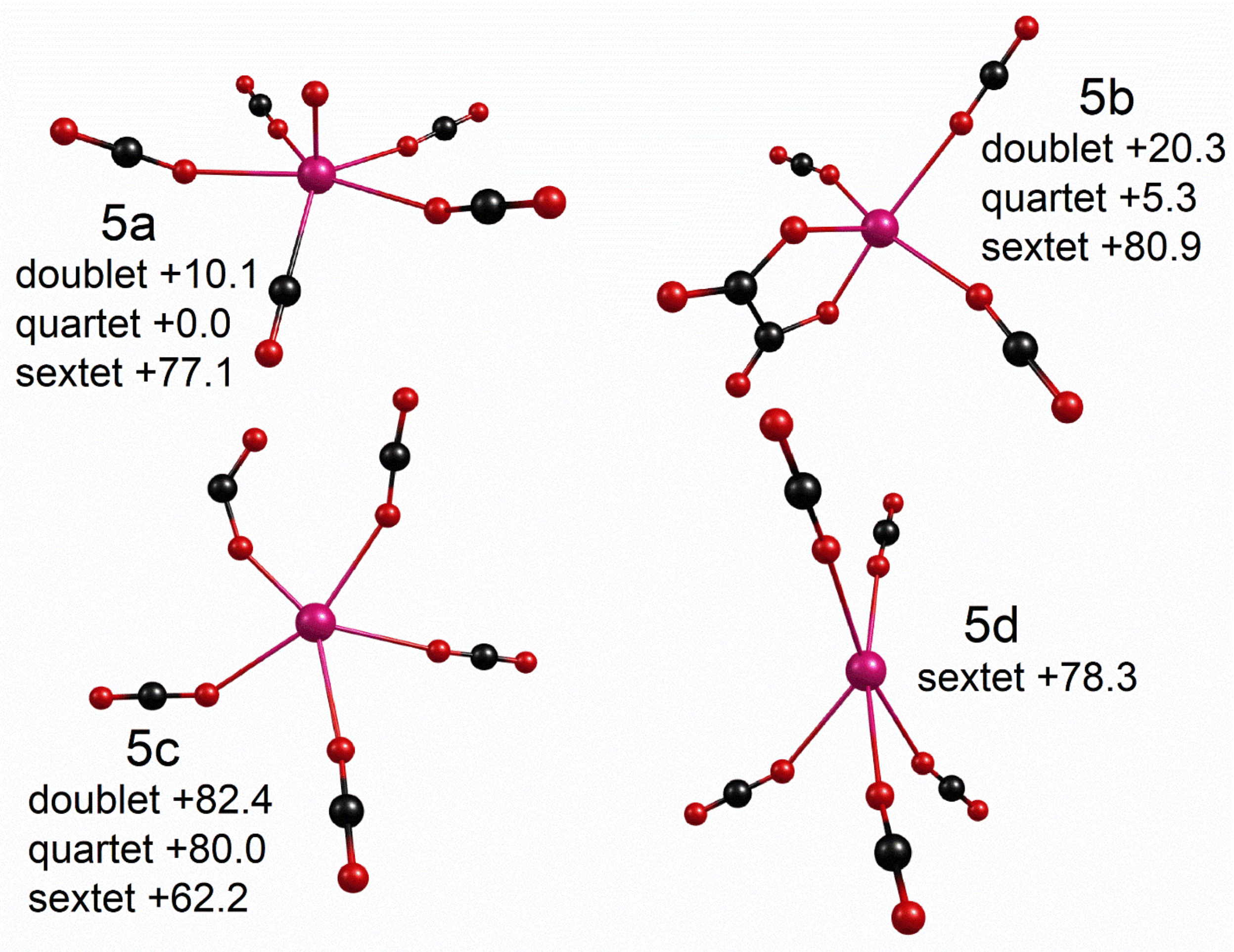

Figure S35. Predicted minimum energy structures of $U^+(CO_2)_5$ with energy of each spin state in kcal/mol. The lowest energy spin state of each isomer is shown.

Table S28. Cartesian coordinates for the optimized geometry of isomer 5a-doublet $U^+(CO_2)_5$ followed by its predicted frequencies (cm^{-1}) and IR intensities (km/mol).

Z	x	y	z
92	0.000026000	0.154794000	-0.060965000
8	2.517145000	0.987658000	0.023878000
6	3.352494000	1.614749000	0.559971000
8	4.178571000	2.221594000	1.071873000
8	1.601243000	-2.004184000	-0.309172000
6	2.388334000	-2.863204000	-0.173221000
8	3.156266000	-3.705018000	-0.047021000
8	-2.516938000	0.988094000	0.024361000
6	-3.351992000	1.615311000	0.560769000
8	-4.177785000	2.222283000	1.072977000
8	0.000149000	0.264536000	1.762025000
6	0.000093000	2.226988000	-1.400102000
8	0.000141000	3.154514000	-2.069823000
8	-1.601687000	-2.003786000	-0.309546000
6	-2.389072000	-2.862618000	-0.174120000
8	-3.157290000	-3.704247000	-0.048425000

Frequency (cm^{-1})	Intensity (km/mol)	Frequency (cm^{-1})	Intensity (km/mol)
3.9699	0.0017	245.7868	1.2089
15.0664	0.6452	269.5564	2.1816
18.8264	0.0434	275.0203	4.1184
22.2355	0.1313	650.3907	4.8359
22.8109	0.3043	651.4778	33.6752
27.404	0.0869	652.6811	7.2496
31.4589	0.1349	653.5406	1.4388
41.4865	3.5875	654.7831	70.9458
53.3764	1.3072	655.7467	35.2721
61.9511	1.3026	657.5556	54.8016
70.0584	1.4737	658.5272	9.9562
75.7311	0.0956	868.5125	290.3497
90.4215	0.0194	1373.3557	33.2618
91.7041	0.089	1373.7961	39.843
104.0266	0.389	1374.9833	36.6689
114.8866	0.0548	1376.4066	19.2428
122.1514	0.5468	2030.1169	1075.3806
135.7848	23.8047	2421.6365	1.2983
149.4829	1.4949	2425.8219	1685.7096
151.6494	52.9489	2429.4241	2136.9921
162.5474	0.9437	2449.2404	339.8802

Table S29. Cartesian coordinates for the optimized geometry of isomer 5a-sextet $U^+(CO_2)_5$ followed by its predicted frequencies (cm^{-1}) and IR intensities (km/mol).

Z	x	y	z
92	-0.021948000	0.003363000	-0.148006000
8	1.858592000	1.847321000	-0.046789000
6	2.690438000	2.674168000	-0.056748000
8	3.503240000	3.482320000	-0.064308000
8	-1.780113000	-1.960306000	-0.123523000
6	-2.565072000	-2.832026000	-0.134155000
8	-3.332140000	-3.683604000	-0.144651000
8	-1.899857000	1.852280000	-0.133987000
6	-2.736752000	2.674272000	-0.146115000
8	-3.554769000	3.477014000	-0.158357000
8	1.938684000	-1.754022000	-0.035627000
6	2.807631000	-2.541718000	-0.046569000
8	3.656962000	-3.311413000	-0.054741000
8	0.097417000	-0.007994000	-2.133986000
6	-0.168134000	0.017303000	2.128438000
8	-0.256692000	0.025726000	3.289402000

Frequency (cm^{-1})	Intensity (km/mol)	Frequency (cm^{-1})	Intensity (km/mol)
11.9153	0.0003	277.495	15.6386
13.2882	0.0439	301.55	0.6161
19.0808	0.2842	544.5033	51.0858
19.4808	0.0514	641.0806	0.832
22.2383	0.1332	642.1321	22.9173
26.5344	0.3025	643.0918	0.7038
50.7835	0.0029	644.7347	83.5159
52.2403	0.2402	653.0951	3.7425
57.7598	0.691	655.8995	59.4907
67.2963	1.4502	656.0574	59.8506
68.4459	2.5018	657.9841	2.369
73.5005	1.0777	1375.356	39.0498
84.6854	0.3491	1375.5699	51.2751
92.7849	5.8497	1376.4001	17.186
96.3093	0.611	1377.4598	5.9687
112.8225	2.922	1886.5716	503.0921
126.5997	0.0305	2426.7922	0.248
131.2297	0.5185	2430.4795	2214.7582
140.7747	21.5199	2430.6205	2235.9861
141.4494	22.6402	2451.447	10.9281
276.0667	16.325		

Table S30. Cartesian coordinates for the optimized geometry of isomer 5b-doublet $U^+(CO_2)_5$ followed by its predicted frequencies (cm^{-1}) and IR intensities (km/mol).

Z	x	y	z
92	0.066191000	-0.358670000	-0.281380000
8	2.400164000	-1.322333000	-0.826756000
6	3.553103000	-1.535970000	-0.943358000
8	4.668370000	-1.750834000	-1.061208000
8	0.606620000	1.695573000	-0.352144000
6	0.904707000	2.428327000	0.760244000
8	1.199046000	3.574456000	0.774311000
8	-2.083105000	0.622311000	-1.427828000
6	-2.689821000	1.514421000	-1.898088000
8	-3.292174000	2.370433000	-2.358938000
8	0.432919000	0.257870000	1.703109000
6	0.801818000	1.532707000	2.046262000
8	1.013559000	1.912811000	3.145359000
8	-1.985969000	-1.773331000	0.623355000
6	-2.782651000	-2.253810000	1.342877000
8	-3.560992000	-2.726510000	2.035655000

Frequency (cm^{-1})	Intensity (km/mol)	Frequency (cm^{-1})	Intensity (km/mol)
10.0347	0.6343	545.1887	2.5924
14.1712	1.2732	557.759	46.0071
17.5091	0.7801	648.1748	18.3557
23.1387	2.6746	650.5023	26.282
25.187	0.6906	650.7676	8.8951
28.9767	0.105	651.2963	45.8351
62.2397	0.1828	654.0545	32.351
65.3483	0.0406	654.8488	53.5077
71.5144	0.1808	770.135	87.0608
76.8577	0.0253	822.6805	0.1305
80.7299	0.5547	853.4814	281.4084
90.4736	0.2052	954.6503	40.0639
105.9233	1.8974	1156.7921	695.5259
135.5032	4.454	1370.1375	54.699
143.2753	2.3719	1371.6691	74.2305
157.5142	2.4232	1373.9544	28.5119
168.32	29.932	1864.5164	157.1438
301.503	32.4944	1876.1399	704.3846
325.2001	19.234	2424.2198	1214.6064
335.1949	7.1675	2432.0713	1901.937
483.3629	39.8858	2444.4613	26.8711

Table S31. Cartesian coordinates for the optimized geometry of isomer 5b-sextet $U^+(CO_2)_5$ followed by its predicted frequencies (cm^{-1}) and IR intensities (km/mol).

Z	x	y	z
92	-0.209813000	-0.000083000	-0.311987000
8	-0.360461000	-2.591483000	-0.552348000
6	-0.076956000	-3.733621000	-0.543606000
8	0.187961000	-4.845924000	-0.538895000
8	1.908838000	0.000425000	-0.849706000
6	2.793183000	0.000680000	0.149400000
8	4.062077000	0.000987000	-0.193923000
8	-0.361690000	2.591231000	-0.552517000
6	-0.078648000	3.733484000	-0.543805000
8	0.185756000	4.845909000	-0.538917000
8	0.898695000	0.000242000	1.447900000
6	2.316761000	0.000604000	1.517119000
8	2.934171000	0.000786000	2.545446000
8	-2.845041000	-0.000576000	0.401738000
6	-3.921732000	-0.000767000	0.873206000
8	-4.971910000	-0.000926000	1.329840000

Frequency (cm^{-1})	Intensity (km/mol)	Frequency (cm^{-1})	Intensity (km/mol)
9.6088	1.2035	520.5406	12.4483
10.0238	0.6226	565.6579	30.0981
15.5079	0.4264	610.6088	48.968
17.414	0.096	635.4168	9.6255
18.3225	2.4027	648.5833	0.104
33.8343	0.4239	649.1924	54.3522
62.9945	0.0592	650.9078	0.4258
71.1386	0.1715	651.0844	37.779
71.5859	0.0367	653.8871	57.362
79.8266	0.1126	655.3238	36.5012
81.7213	0.3247	801.9442	327.342
82.6106	0.0578	938.1758	108.049
120.0493	7.9483	1239.4243	5.4595
128.6575	1.5671	1269.0999	157.2444
150.1936	1.0938	1370.2051	38.5188
160.3557	30.7131	1371.6357	90.8646
163.9142	0	1373.302	0.0311
257.666	15.931	1745.2779	495.8746
285.5045	2.5594	2425.0518	1023.6324
319.4578	8.7324	2430.1775	2069.1163
320.524	18.6173	2443.0731	133.3387

Table S32. Cartesian coordinates for the optimized geometry of isomer 5c-doublet $U^+(CO_2)_5$ followed by its predicted frequencies (cm^{-1}) and IR intensities (km/mol).

Z	x	y	z
92	-0.084133000	0.062862000	0.001538000
8	2.439348000	-0.527679000	0.159154000
6	3.585798000	-0.275532000	0.270933000
8	4.710653000	-0.105881000	0.382244000
8	-2.093949000	1.748473000	-0.221747000
6	-2.339925000	2.892948000	-0.344658000
8	-2.598963000	4.000755000	-0.465747000
8	0.348381000	-2.573829000	-0.248190000
6	0.690929000	-3.680591000	-0.435051000
8	1.019076000	-4.763649000	-0.617842000
8	-2.462478000	-1.165985000	0.254371000
6	-3.556623000	-1.547021000	0.441836000
8	-4.623262000	-1.924407000	0.625377000
8	0.888843000	1.988762000	0.017273000
6	1.947102000	2.771952000	0.034886000
8	3.094423000	2.479205000	0.121459000

Frequency (cm^{-1})	Intensity (km/mol)	Frequency (cm^{-1})	Intensity (km/mol)
4.5237	0.0248	174.2083	5.7045
10.0997	1.637	379.6946	81.6826
19.1574	0.0834	617.691	54.0519
21.9244	0.5305	645.4765	7.9121
22.6851	0.0654	645.7817	11.1787
27.073	0.6008	647.0338	12.2118
54.9753	0.2126	648.0683	8.4284
56.1947	0.0757	648.7099	73.0985
61.4055	0.0281	649.4731	42.1303
64.6707	0.0006	654.0959	36.5171
74.2222	0.5006	714.9269	5.3989
86.7884	0.8814	1110.2874	484.2497
88.0244	2.2021	1363.9265	44.4179
94.591	0.3607	1366.6573	41.6082
102.7717	0.1989	1372.1622	26.7901
107.5103	1.6724	1373.4119	16.783
121.3908	9.0323	1815.1433	423.5905
134.2958	9.1492	2418.3003	173.3365
141.2437	7.1988	2421.5368	1671.6466
147.1069	0.8706	2425.5773	2155.5547
162.2417	3.0906	2445.7526	168.9563

Table S33. Cartesian coordinates for the optimized geometry of isomer 5c-quartet $U^{+}(CO_2)_5$ followed by its predicted frequencies (cm^{-1}) and IR intensities (km/mol).

Z	x	y	z
92	-0.066137000	-0.078827000	0.007312000
8	2.402986000	0.593443000	-0.381441000
6	3.539115000	0.371105000	-0.606368000
8	4.649808000	0.228123000	-0.834872000
8	-2.076587000	-1.738585000	0.357857000
6	-2.381906000	-2.848880000	0.600113000
8	-2.696908000	-3.923367000	0.836212000
8	0.245986000	2.527044000	0.524077000
6	0.533458000	3.601087000	0.900571000
8	0.809092000	4.650896000	1.269007000
8	-2.420927000	1.115688000	-0.538023000
6	-3.466557000	1.499073000	-0.908054000
8	-4.486300000	1.877607000	-1.270431000
8	0.962815000	-1.972043000	0.114400000
6	2.045917000	-2.712556000	0.033242000
8	3.168087000	-2.384664000	-0.1755050

Frequency (cm^{-1})	Intensity (km/mol)	Frequency (cm^{-1})	Intensity (km/mol)
7.3639	0.5394	168.8853	6.9758
14.2851	0.8586	383.3777	84.4773
19.9091	0.3693	617.9502	54.9474
21.9942	0.5491	642.3865	9.1998
23.5955	0.2334	644.9907	36.7424
25.8082	0.3181	645.965	6.5433
55.0185	0.1408	647.4248	15.576
56.1502	0.1716	648.5891	53.9082
61.5128	0.0456	649.3014	33.9373
67.5408	0.0339	650.9363	32.8923
75.0126	0.2594	713.0046	4.8562
84.8424	1.813	1115.1935	489.4592
87.1579	1.9961	1363.0637	45.7816
89.4627	0.1534	1367.1937	38.7727
102.9404	0.7679	1370.921	26.2727
103.9547	1.2024	1372.1057	13.2856
122.9062	8.7178	1813.4974	441.5751
126.9088	5.8154	2418.0589	430.7373
137.7308	8.8447	2421.1079	1567.7277
148.4508	0.8295	2425.2837	2099.7511
158.3684	3.251	2445.2945	151.8263

Table S34. Cartesian coordinates for the optimized geometry of isomer 5c-sextet $U^+(CO_2)_5$ followed by its predicted frequencies (cm^{-1}) and IR intensities (km/mol).

Z	x	y	z
92	-0.074490000	0.078057000	-0.022150000
8	0.331100000	-2.492484000	-0.685548000
6	0.640177000	-3.529439000	-1.140716000
8	0.937300000	-4.542884000	-1.585924000
8	2.407252000	-0.545450000	0.423601000
6	3.525633000	-0.289106000	0.696152000
8	4.621266000	-0.111150000	0.967693000
8	-2.355624000	-1.181205000	0.714769000
6	-3.334838000	-1.589257000	1.217142000
8	-4.289724000	-1.990159000	1.708462000
8	-2.128467000	1.644043000	-0.520155000
6	-2.498967000	2.720602000	-0.816154000
8	-2.876033000	3.761242000	-1.106756000
8	0.904040000	2.013450000	-0.033089000
6	1.962771000	2.776157000	0.083969000
8	3.084437000	2.480223000	0.341381000

Frequency (cm^{-1})	Intensity (km/mol)	Frequency (cm^{-1})	Intensity (km/mol)
11.956	0.0669	166.1655	9.0902
14.7729	1.5928	381.7751	87.9723
19.2588	0.2936	619.7331	56.0644
20.3799	0.444	643.4355	8.517
23.5641	0.3552	645.0453	5.1588
24.3664	0.3109	646.0823	36.7146
52.857	0.2544	647.9086	32.0532
54.0057	0.1002	649.0169	32.0893
59.4583	0.2817	650.9646	41.0327
66.5455	0.0262	653.2811	32.9293
71.836	0.1187	716.3426	4.135
79.6257	0.9432	1142.3074	518.7092
85.6062	1.6222	1364.4996	52.9855
85.8721	1.4235	1368.8934	42.1576
92.3114	0.6584	1371.7743	31.8636
103.5028	1.297	1372.9698	13.2018
120.5955	10.0618	1813.5652	462.0253
122.6387	5.7885	2418.7973	509.8288
132.1304	6.2236	2421.4282	1566.7123
147.6172	1.6503	2426.3187	2012.1726
157.5214	4.2651	2445.0763	107.0696

Table S35. Cartesian coordinates for the optimized geometry of isomer 5d-sextet $U^+(CO_2)_5$ followed by its predicted frequencies (cm^{-1}) and IR intensities (km/mol).

Z	x	y	z
92	-0.226343000	0.000001000	0.115783000
8	1.873597000	1.553963000	0.780667000
6	2.589128000	2.371617000	1.218349000
8	3.283390000	3.177631000	1.652730000
8	1.873609000	-1.553900000	0.780780000
6	2.589134000	-2.371523000	1.218530000
8	3.283390000	-3.177506000	1.652977000
8	-2.405282000	-1.494530000	-0.000825000
6	-3.242882000	-2.314543000	-0.026379000
8	-4.058334000	-3.122511000	-0.052232000
8	1.775185000	-0.000075000	-1.805403000
6	2.258440000	-0.000101000	-2.871273000
8	2.727798000	-0.000128000	-3.921803000
8	-2.405279000	1.494536000	-0.000826000
6	-3.242879000	2.314548000	-0.026369000
8	-4.058334000	3.122514000	-0.052211000

Frequency (cm^{-1})	Intensity (km/mol)	Frequency (cm^{-1})	Intensity (km/mol)
8.0455	0.0024	134.9979	7.8794
13.8243	0.0002	612.2143	0.18
18.2361	0.0032	616.6204	32.1583
22.2932	0.0031	619.0544	10.9007
22.4785	0.0028	622.0219	54.4991
25.1737	0.0027	628.3308	15.3952
26.5954	0.0205	636.8601	29.8768
38.7455	0.023	639.1988	7.005
43.7161	0.407	641.4899	40.3738
47.3436	0.1828	642.2777	13.0951
49.0842	0.5271	644.9317	16.8955
59.8824	0.0024	1361.2513	6.1553
66.4559	1.724	1363.2679	5.0294
79.3605	0.582	1364.0453	0.5015
84.2535	2.0742	1364.7753	7.6874
93.9493	0.003	1366.4224	1.0866
99.5627	0.0554	2400.875	76.158
99.5909	0.7349	2413.6883	1377.068
104.4955	0.0469	2416.7219	2553.7028
113.9955	2.8913	2426.6462	2399.9833
117.6849	0.6803	2446.6947	8.3348

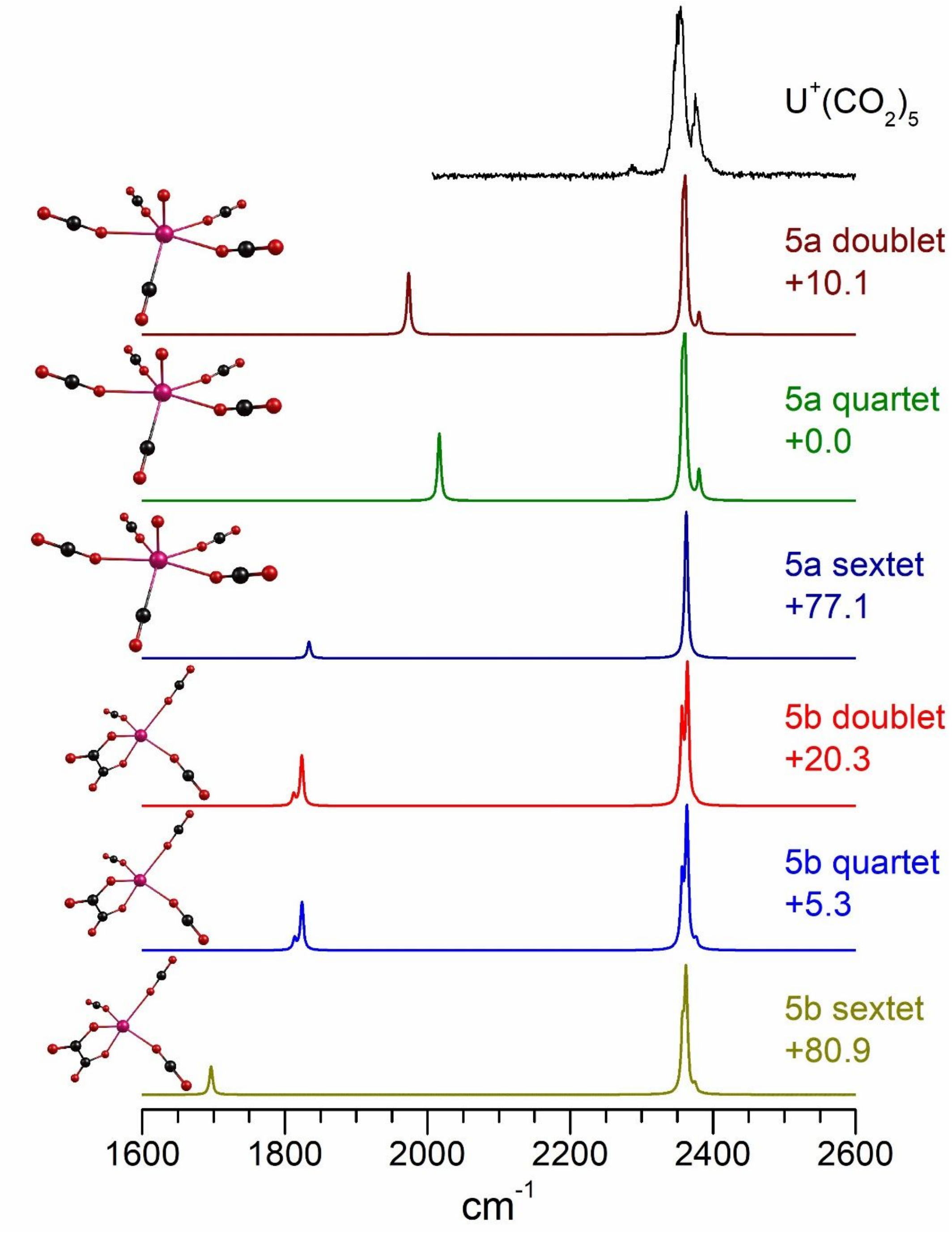

Figure S36. Experimental IR spectrum of $U^{+}(CO_2)_5$ compared with simulated spectra for isomers 5a and 5b. Relative energies (kcal/mol) are shown next to each spectrum.

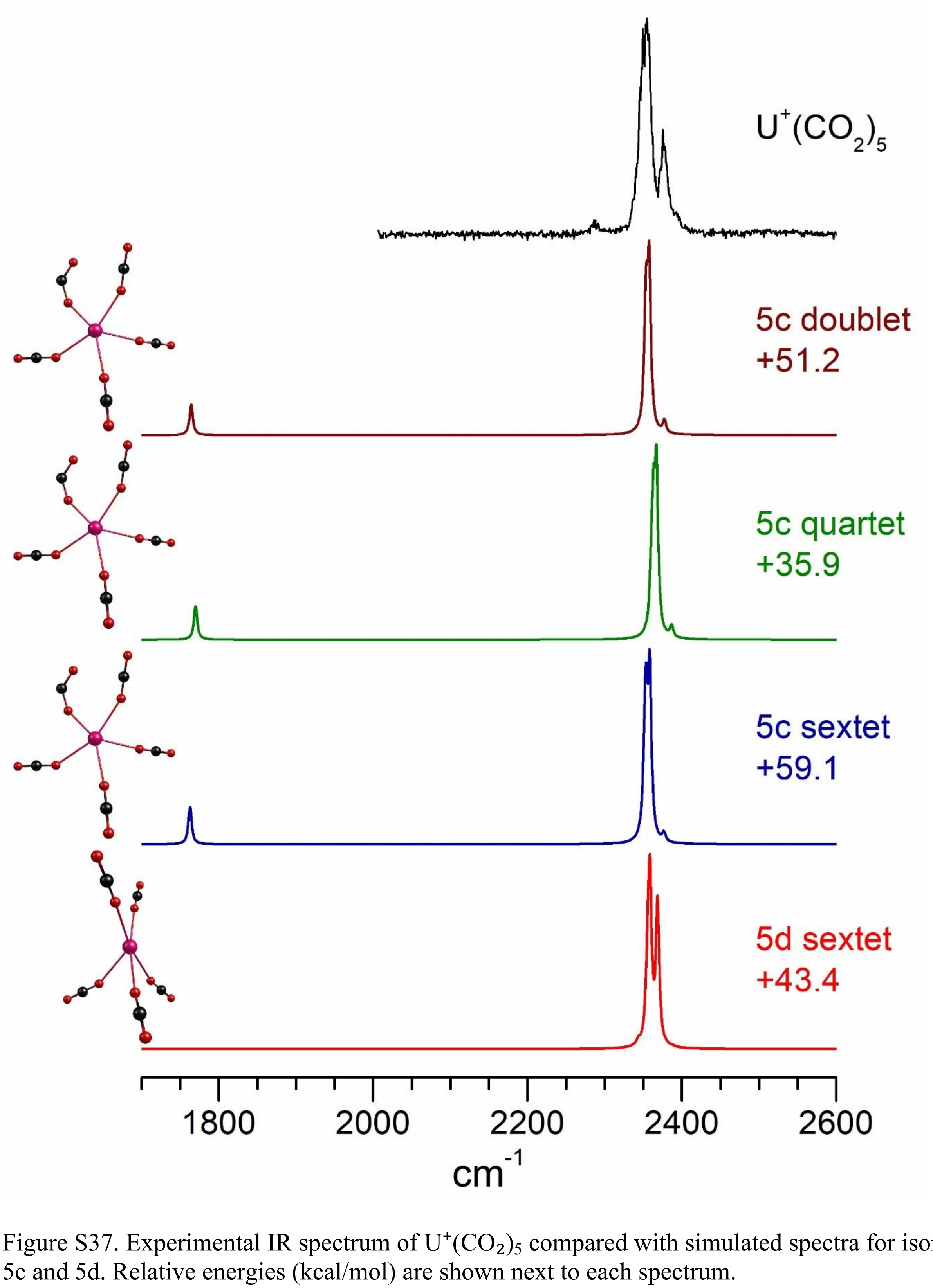

Figure S37. Experimental IR spectrum of $U^{+}(CO_2)_5$ compared with simulated spectra for isomers 5c and 5d. Relative energies (kcal/mol) are shown next to each spectrum.

Table S36. $U^+(CO_2)_6$ electronic energy calculated at the B3LYP/cc-pVTZ(-pp) level with Stuttgart/Koeln pseudopotential.

Isomer	2s + 1	Energy (hartree)	Rel. E (kcal/mol)	BDE (CO_2) (kcal/mol)	BDE (CO) (kcal/mol)	BDE(oxalate) (kcal/mol)
6a	2	-1606.511211	+11.8	4.5	13.9	
6a	4	-1606.530093	+0.0	6.2	12.7	
6a	6	-1606.408329	+76.4	6.9		
6b	2	-1606.502120	+17.6	9.0	-22.6	82.9
6b	4	-1606.527028	+1.9	9.6	21.2	82.9
6b	6	-1606.405866	+78.0	9.2		10.3
6c	2	-1606.491723	+24.1	-7.8	1.7	
6c	4	-1606.512616	+11.0	-4.7	1.8	
6d	2	-1606.417083	+70.9	29.5	-45.1	
6d	4	-1606.400558	+81.3	3.6	-68.5	
6d	6	-1606.427682	+64.3	24.0		
6e	6	-1606.400953	+81.0	3.5		
6f	6	-1606.400354	+81.4	1.9		

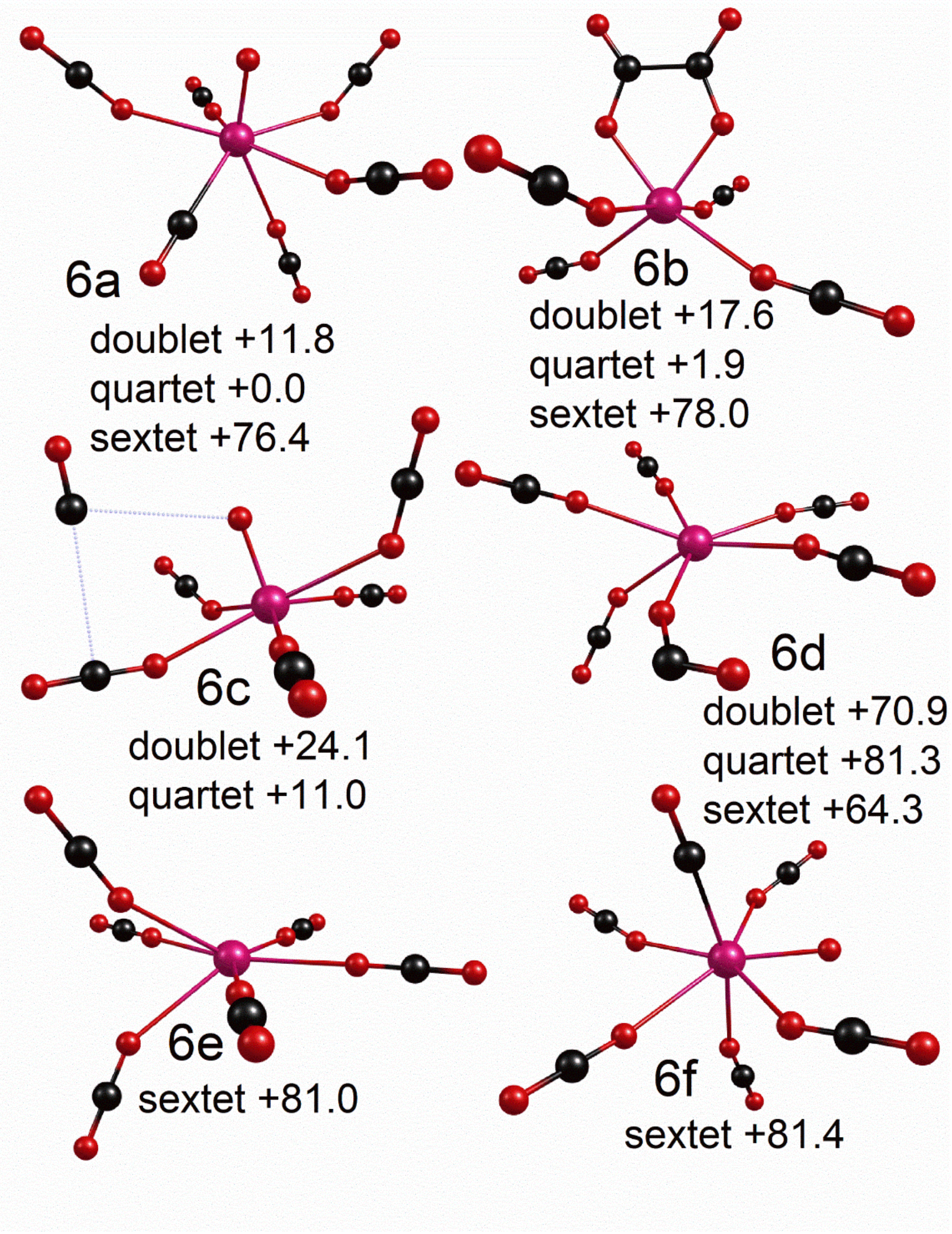

Figure S38. Predicted minimum energy structures of $U^+(CO_2)_6$ with energy of each spin state in kcal/mol. The lowest energy spin state of each isomer is shown.

.

Table S37. Cartesian coordinates for the optimized geometry of isomer 6a-sextet $U^+(CO_2)_6$ followed by its predicted frequencies (cm^{-1}) and IR intensities (km/mol).

Z	x	y	z
92	-0.000267000	-0.000831000	-0.101981000
8	-2.484699000	0.998067000	-0.090672000
6	-3.570513000	1.435567000	-0.117413000
8	-4.634906000	1.864416000	-0.142272000
8	0.181850000	2.670662000	-0.101974000
6	0.262248000	3.837986000	-0.146827000
8	0.341051000	4.982314000	-0.188930000
8	1.421564000	-2.269629000	-0.091798000
6	2.047260000	-3.258219000	-0.139867000
8	2.660606000	-4.227364000	-0.184991000
8	2.597049000	0.650805000	-0.086798000
6	3.732207000	0.937545000	-0.104494000
8	4.844973000	1.218526000	-0.120600000
8	-1.717482000	-2.055078000	-0.085848000
6	-2.470025000	-2.951554000	-0.119284000
8	-3.207757000	-3.830339000	-0.150198000
8	-0.000394000	-0.005529000	-2.095392000
6	0.000087000	0.004952000	2.181657000
8	0.000266000	0.007996000	3.346930000

Frequency	Intensity	Frequency	Intensity	Frequency	Intensity
4.7876	0	100.1209	0.0002	648.9087	128.8647
4.8213	0	100.2029	0.0017	650.4115	0.0003
19.7668	0.0246	110.946	0.0007	654.7521	76.6154
19.7966	0.0219	111.0109	0.0021	654.7644	76.3167
19.8589	0.2389	115.5267	0.8564	659.5228	0
20.4084	0.0003	116.281	0.8227	659.5387	0
25.0962	0.3186	123.0389	0.2748	1377.5579	0.06
25.1632	0.3194	136.6895	28.4757	1377.569	0.0333
27.3243	0	136.7215	28.4256	1378.1166	49.5965
27.3591	0	275.363	16.3771	1378.1467	49.8698
60.9766	1.6627	275.5412	16.4387	1379.661	0.1009
61.0934	1.6613	303.2951	0.5866	1880.7927	542.8847
73.4947	1.8472	539.6185	52.2627	2423.1557	0.3135
73.6761	1.9014	644.5725	0.0017	2423.1694	0.0197
73.8388	0.0002	644.5821	0.0034	2433.3031	2648.7584
73.9181	0.0486	646.7686	0.2188	2433.3361	2657.2187
94.8838	0.8701	646.7874	0.2748	2458.1403	14.828

Table S38. Cartesian coordinates for the optimized geometry of isomer 6b-doublet $U^{+}(CO_2)_6$ followed by its predicted frequencies (cm^{-1}) and IR intensities (km/mol).

Z	x	y	z
92	-0.000034000	-0.000397000	-0.298451000
8	1.648533000	1.623966000	-1.661789000
6	2.389764000	2.490856000	-1.944052000
8	3.111493000	3.333724000	-2.227566000
8	2.306898000	-1.255596000	-0.415615000
6	3.128711000	-1.999511000	-0.022149000
8	3.936296000	-2.720873000	0.347093000
8	-0.254171000	-1.233881000	1.431368000
6	-0.147908000	-0.767531000	2.705959000
8	-0.256015000	-1.417931000	3.691062000
8	0.254282000	1.238538000	1.427448000
6	0.147774000	0.776292000	2.703506000
8	0.256317000	1.429728000	3.686548000
8	-2.307213000	1.253949000	-0.419270000
6	-3.129190000	1.998903000	-0.028114000
8	-3.936694000	2.721620000	0.338647000
8	-1.648252000	-1.629232000	-1.656954000
6	-2.389346000	-2.497064000	-1.936671000
8	-3.110935000	-3.340904000	-2.217643000

Frequency	Intensity	Frequency	Intensity	Frequency	Intensity
10.1654	0.0304	129.8157	9.6932	654.9367	42.092
10.5248	2.5961	131.8236	3.6454	655.4156	96.1519
16.3547	0.7764	149.5851	2.9057	773.3268	94.751
18.9009	0.2816	155.0511	1.7444	827.007	0.1861
22.0117	0.0678	160.442	28.4569	854.8005	258.4161
23.9412	3.9924	296.1996	36.0957	978.7205	37.9953
24.0674	0.2532	314.4647	23.0577	1171.751	682.4086
24.7087	0.0124	334.3015	9.1025	1370.4734	64.1936
58.2915	0.0014	484.3254	42.1946	1370.6816	10.9228
65.8305	0.0063	547.554	2.4653	1371.1358	72.4625
66.3769	0.6913	549.3727	54.2773	1372.7679	0.0042
69.1497	0.0023	648.1668	2.3748	1852.6971	118.9289
74.717	0.0747	649.4087	31.2563	1863.5148	771.4121
80.561	0.0177	650.3496	49.9176	2423.3227	334.618
82.6623	0.398	650.7337	6.2936	2423.6713	1998.8961
89.5512	1.0772	653.6311	7.1355	2429.7974	1949.5969
111.3984	0.7772	654.5036	8.8867	2447.2899	25.6658

Table S39. Cartesian coordinates for the optimized geometry of isomer 6b-quartet $U^+(CO_2)_6$ followed by its predicted frequencies (cm^{-1}) and IR intensities (km/mol).

Z	x	y	z
92	-0.000014000	0.000044000	0.311281000
8	-1.681194000	1.621256000	1.652185000
6	-2.434594000	2.486349000	1.906054000
8	-3.168750000	3.327109000	2.162419000
8	-2.270276000	-1.328387000	0.420924000
6	-3.076549000	-2.086488000	0.022503000
8	-3.869030000	-2.821977000	-0.351404000
8	0.309684000	-1.225370000	-1.420295000
6	0.183891000	-0.764641000	-2.694198000
8	0.324496000	-1.409252000	-3.679370000
8	-0.309636000	1.225031000	-1.420607000
6	-0.183790000	0.763990000	-2.694394000
8	-0.324353000	1.408361000	-3.679729000
8	2.270264000	1.328475000	0.420696000
6	3.076569000	2.086466000	0.022132000
8	3.869080000	2.821853000	-0.351915000
8	1.681142000	-1.620849000	1.652595000
6	2.434536000	-2.485891000	1.906656000
8	3.168687000	-3.326598000	2.163210000

Frequency	Intensity	Frequency	Intensity	Frequency	Intensity
5.8011	4.9088	130.7597	10.0809	655.4054	55.9384
10.9836	0.0428	132.2883	3.8321	655.8413	103.8774
12.8662	0.282	147.1908	0.1131	770.966	126.2643
19.2473	0.324	154.4022	1.9026	827.9841	0.1924
20.6424	0.0842	157.7596	34.6292	855.8185	247.5014
22.385	0.0329	296.0622	35.6979	981.4106	29.3294
24.003	4.2101	308.1757	38.8007	1174.7309	681.7454
25.1871	0.0199	334.3921	8.9894	1371.3951	77.5304
57.9962	0.0016	484.5426	43.612	1371.6486	11.1967
65.5889	1.2659	545.8252	6.2867	1371.9639	73.1526
66.8252	0.002	548.6949	50.749	1373.6711	0.066
69.1587	0.0092	650.0499	0.8291	1850.7351	140.2438
75.7136	0.0287	651.0353	36.2042	1862.3297	757.7986
78.6662	2.1021	652.3339	27.9778	2423.8105	310.3274
80.6905	0.0339	652.5868	5.9694	2424.1155	2091.7555
82.5406	2.0067	654.2114	4.8059	2428.3888	1964.5467
110.8081	0.5941	655.3404	4.7327	2447.4625	19.0394

Table S40. Cartesian coordinates for the optimized geometry of isomer 6b-sextet $U^+(CO_2)_6$ followed by its predicted frequencies (cm^{-1}) and IR intensities (km/mol).

Z	x	y	z
92	-0.004050000	-0.033984000	-0.370370000
8	1.905121000	1.241300000	-1.816379000
6	2.801019000	1.950434000	-2.087647000
8	3.674920000	2.639560000	-2.360635000
8	1.903695000	-1.807023000	-0.078792000
6	2.570941000	-2.568339000	0.519389000
8	3.228031000	-3.313664000	1.087127000
8	-0.790584000	-0.772936000	1.448144000
6	-0.525370000	-0.229456000	2.714347000
8	-0.995148000	-0.647425000	3.740786000
8	0.798485000	1.178226000	1.280409000
6	0.387613000	0.879869000	2.513895000
8	0.858830000	1.638699000	3.478706000
8	-1.903069000	1.755634000	-0.554425000
6	-2.677028000	2.563971000	-0.193858000
8	-3.435369000	3.352456000	0.141863000
8	-1.744765000	-1.686284000	-1.592768000
6	-2.595891000	-2.488203000	-1.708610000
8	-3.424541000	-3.268937000	-1.832922000

Frequency	Intensity	Frequency	Intensity	Frequency	Intensity
6.1706	1.3751	135.5573	7.706	653.5733	42.6648
10.4272	0.1124	139.2797	7.2917	654.6928	25.2245
11.2958	2.2751	150.0631	0.7529	655.5366	43.8677
14.9972	0.4849	158.3355	18.8704	655.7826	80.4127
17.6407	0.6024	164.2855	7.3448	825.7713	245.0273
18.7802	0.6882	254.4064	16.2206	958.084	141.98
19.825	1.9494	279.3399	2.5078	1206.6855	13.5512
27.8634	0.4597	308.3382	24.7741	1266.3736	159.8388
58.9499	0.1567	309.3324	11.895	1370.4361	46.4887
61.7398	0.0006	515.9396	20.4652	1370.84	29.2675
70.4052	0.3858	566.3848	34.2565	1371.3206	60.0353
75.4554	0.1054	601.6966	30.1816	1373.0481	4.7122
77.9421	0.0526	640.1383	11.3924	1726.6979	493.3932
82.1157	0.1474	648.6997	4.3396	2424.0774	635.2527
85.6157	0.1775	649.6009	46.8892	2424.4575	1675.8859
88.1312	0.9563	651.808	1.7159	2428.6795	1919.4138
128.7517	6.2043	652.8314	4.1648	2445.4878	9.3484

Table S41. Cartesian coordinates for the optimized geometry of isomer 6c-doublet $U^+(CO_2)_6$ followed by its predicted frequencies (cm^{-1}) and IR intensities (km/mol).

Z	x	y	z
92	-0.234173000	0.043773000	-0.236985000
8	0.952808000	2.361628000	-0.878035000
6	1.431045000	3.396360000	-1.148483000
8	1.899816000	4.409646000	-1.413586000
8	-1.903323000	2.020806000	0.484856000
6	-2.227905000	2.526332000	1.493914000
8	-2.557924000	3.033420000	2.467809000
8	-0.122235000	-2.624399000	-0.449341000
6	0.027518000	-3.673085000	0.054031000
8	0.172784000	-4.705007000	0.533377000
8	-2.745648000	-0.888393000	-0.434186000
6	-3.849441000	-1.234833000	-0.619466000
8	-4.930424000	-1.575319000	-0.799268000
8	2.220338000	-0.514129000	-1.090137000
6	3.361476000	-0.783738000	-1.090066000
8	4.476802000	-1.048731000	-1.121386000
8	0.123422000	-0.072344000	1.569274000
6	3.400052000	-0.639165000	2.126054000
8	3.499510000	-0.594467000	3.243968000

Frequency	Intensity	Frequency	Intensity	Frequency	Intensity
6.2607	0.0934	78.723	0.3747	651.7169	47.5697
6.9359	0.0035	91.1113	0.2616	652.5317	48.2032
9.27	0.0294	97.9538	1.1733	654.5329	48.0864
16.7845	0.0995	100.2477	0.6176	655.3696	1.8442
20.4088	0.0426	108.3454	1.7106	656.8442	23.1662
22.9276	0.0058	112.8667	0.5854	836.9961	388.7812
25.9913	0.136	122.6133	3.9615	1367.5898	23.5372
26.6965	0.0437	134.4605	11.1644	1372.4639	18.5182
28.6889	0.8938	139.9307	22.7145	1374.8367	25.7661
30.6619	2.6902	144.5837	4.445	1376.3878	28.8475
37.2306	0.101	149.3454	25.4089	1377.1467	4.5853
47.4436	2.3187	167.424	0.6993	2235.9488	71.0214
60.7937	0.406	639.882	23.9417	2418.1552	657.6085
63.5881	0.3041	645.9256	1.5564	2420.0508	126.7438
72.1732	0.2329	646.3064	2.8002	2427.3443	1884.1066
74.1489	0.0111	647.4486	39.5587	2430.6371	2357.2963
77.8421	0.2268	647.8668	42.6285	2452.1088	45.338

Table S42. Cartesian coordinates for the optimized geometry of isomer 6c-quartet $U^+(CO_2)_6$ followed by its predicted frequencies (cm^{-1}) and IR intensities (km/mol).

Z	x	y	z
92	0.237884000	0.072546000	0.302454000
8	-1.196651000	2.279473000	0.869001000
6	-1.793816000	3.274384000	1.030310000
8	-2.378727000	4.248551000	1.190519000
8	1.682126000	2.176996000	-0.551953000
6	1.869201000	2.578373000	-1.640308000
8	2.066695000	2.989593000	-2.691903000
8	0.406504000	-2.603406000	0.436194000
6	0.341914000	-3.587833000	-0.198798000
8	0.281181000	-4.559601000	-0.804900000
8	2.844985000	-0.583834000	0.526475000
6	3.984659000	-0.817243000	0.664845000
8	5.100822000	-1.046729000	0.800432000
8	-2.159168000	-0.731413000	1.147719000
6	-3.264548000	-1.122157000	1.129320000
8	-4.345281000	-1.505564000	1.143295000
8	-0.093610000	-0.109297000	-1.518474000
6	-3.263276000	-0.953768000	-2.082496000
8	-3.350147000	-0.917869000	-3.201784000

Frequency	Intensity	Frequency	Intensity	Frequency	Intensity
6.1721	0.1554	79.6504	0.6935	654.6644	47.2444
7.4299	0.0032	92.8898	0.1491	656.2665	68.5261
10.2033	0.0421	96.8265	0.8728	656.3697	35.1865
17.0936	0.1088	98.5726	0.4774	658.5625	5.5782
20.1735	0.09	109.1808	1.2759	660.7041	23.9784
23.1568	0.003	113.2001	0.325	821.9963	347.4193
25.9993	0.2383	123.5171	3.9235	1365.6672	23.8539
26.7073	0.1315	137.5462	9.8961	1371.3317	21.6533
30.1428	2.5135	142.2386	33.3183	1374.8807	25.433
31.3484	1.9431	151.9884	21.1896	1376.7553	33.2885
39.5263	0.1936	153.9556	9.3004	1377.3967	3.5314
48.9049	2.95	179.6604	0.9418	2235.9783	67.2371
60.9149	0.4149	641.9314	27.2024	2416.9716	637.4629
64.0381	0.1925	649.1741	0.6069	2419.5068	229.8304
71.4019	0.1271	650.6767	34.9413	2426.823	1661.6722
74.2273	0.0016	650.8631	9.2885	2430.7715	2301.428
77.099	0.3558	651.8541	43.061	2452.0343	69.9655

Table S43. Cartesian coordinates for the optimized geometry of isomer 6d-quartet $U^{+}(CO_2)_6$ followed by its predicted frequencies (cm^{-1}) and IR intensities (km/mol).

Z	x	y	z
92	-0.116869000	0.071844000	-0.006921000
8	1.837312000	1.886328000	-0.527449000
6	2.778876000	2.508565000	-0.844515000
8	3.696821000	3.122594000	-1.155977000
8	-1.220186000	2.491277000	0.156720000
6	-1.858864000	3.457874000	0.341997000
8	-2.476184000	4.406767000	0.524154000
8	1.940856000	-1.160606000	-1.304080000
6	2.303746000	-2.088357000	-1.923457000
8	2.662159000	-2.987618000	-2.539628000
8	-2.683425000	-0.060372000	-0.342976000
6	-3.710120000	-0.625413000	-0.468248000
8	-4.738398000	-1.104433000	-0.612389000
8	2.067938000	-0.428547000	1.555567000
6	2.507607000	-0.936811000	2.516898000
8	2.939570000	-1.423504000	3.462169000
8	-0.564823000	-2.010680000	0.405458000
6	-1.404497000	-3.008940000	0.524884000
8	-2.580208000	-3.037604000	0.347347000

Frequency	Intensity	Frequency	Intensity	Frequency	Intensity
8.0894	0.2933	96.1312	0.3853	646.8917	35.4005
16.727	1.4661	100.8085	0.7008	649.0798	22.0139
18.7256	0.5444	107.8338	2.29	651.0937	39.7539
19.6599	0.2788	110.1446	0.0253	653.5142	36.4286
20.7914	0.0269	121.5049	6.153	714.0334	5.4408
21.866	0.1628	127.5892	4.5672	1133.3956	476.6942
24.3259	0.435	132.0703	7.5793	1364.2531	39.5698
27.4154	0.1166	140.3886	1.2212	1367.8259	20.2881
44.266	0.4302	143.9703	10.7963	1369.2235	18.7947
48.8661	0.1091	165.8615	2.936	1371.86	20.2743
58.2834	0.4044	374.6005	80.9305	1372.9416	11.1365
64.8432	0.6488	615.1153	55.9461	1805.6525	462.9749
69.393	0.8849	636.1546	17.1329	2415.0281	881.5697
78.4644	1.1239	639.2677	15.6529	2417.5725	542.2793
83.7201	0.0475	639.41	11.3635	2421.4125	1669.037
87.6924	1.2361	643.8834	27.5496	2425.6741	2266.6728
92.1473	2.8616	645.9126	17.5712	2447.418	74.7498

Table S44. Cartesian coordinates for the optimized geometry of isomer 6d-sextet $U^+(CO_2)_6$ followed by its predicted frequencies (cm^{-1}) and IR intensities (km/mol).

Z	x	y	z
92	-0.124066000	-0.274938000	-0.004528000
8	0.284666000	1.931192000	1.612482000
6	0.503466000	2.580383000	2.563228000
8	0.713980000	3.212976000	3.498324000
8	0.843286000	-2.224467000	-0.132423000
6	1.900579000	-2.990193000	-0.198168000
8	3.053344000	-2.697683000	-0.200389000
8	-2.514766000	1.067925000	-0.104327000
6	-3.633670000	1.415530000	-0.153540000
8	-4.727472000	1.757913000	-0.202343000
8	-2.140862000	-1.961854000	0.095765000
6	-2.358700000	-3.118137000	0.113189000
8	-2.594051000	-4.237946000	0.132468000
8	0.259000000	2.072659000	-1.421171000
6	0.495113000	2.806659000	-2.303475000
8	0.723081000	3.523006000	-3.171716000
8	2.441067000	0.313417000	-0.005486000
6	3.587131000	0.041910000	-0.027607000
8	4.715048000	-0.147469000	-0.044336000

Frequency	Intensity	Frequency	Intensity	Frequency	Intensity
8.779	0.9954	90.6555	1.4359	650.4544	51.7733
14.2017	0.8575	91.9011	1.8527	652.6542	28.1341
18.1715	0.3865	95.5201	0.8762	653.0827	41.2618
19.9034	0.2384	97.8703	0.4479	654.4623	38.349
21.2576	0.0071	115.9195	10.5112	720.6854	4.3358
23.0289	0.0466	128.2565	14.1652	1156.1138	499.0296
24.6151	0.0942	129.0545	1.3111	1365.3395	37.2998
25.9492	0.8804	133.0558	1.0683	1366.4523	41.9934
44.9624	0.2322	156.1026	0.9006	1369.5262	19.3087
46.7912	0.4858	171.9739	7.2229	1370.7653	11.2076
54.5212	0.0892	371.126	92.5573	1372.8366	16.2907
57.7426	0.0474	617.5422	55.3533	1810.8246	455.5224
66.6429	0.1704	637.0578	22.4377	2416.0508	133.9955
73.0556	0.1775	639.2953	8.5465	2416.9962	1287.897
80.5033	0.4907	644.3234	16.0515	2420.6852	1599.4649
86.4955	1.1476	648.0652	0.8576	2423.9977	2118.7694
87.9196	2.2276	649.5169	24.1952	2446.9808	131.1971

Table S45. Cartesian coordinates for the optimized geometry of isomer 6e-sextet $U^+(CO_2)_6$ followed by its predicted frequencies (cm^{-1}) and IR intensities (km/mol).

Z	x	y	z
92	0.241773000	-0.000029000	0.020641000
8	2.406382000	1.514518000	0.033130000
6	3.322274000	2.245679000	0.035537000
8	4.216631000	2.966836000	0.038173000
8	2.406140000	-1.514910000	0.033142000
6	3.321925000	-2.246206000	0.035551000
8	4.216175000	-2.967495000	0.038190000
8	-2.124055000	0.000171000	1.402338000
6	-2.835058000	0.000211000	2.331957000
8	-3.528464000	0.000250000	3.249242000
8	-0.753172000	-2.534283000	0.057123000
6	-1.150719000	-3.635531000	0.066357000
8	-1.537387000	-4.720323000	0.075454000
8	-0.752671000	2.534409000	0.057147000
6	-1.150060000	3.635715000	0.066411000
8	-1.536571000	4.720562000	0.075539000
8	-2.049645000	0.000215000	-1.553177000
6	-2.522059000	0.000254000	-2.624352000
8	-2.983477000	0.000293000	-3.677276000

Frequency	Intensity	Frequency	Intensity	Frequency	Intensity
13.3264	0.0499	86.4419	0.001	638.9451	49.2774
14.9059	0.0632	89.5718	5.3358	641.2007	12.4584
15.7141	0.0328	91.6139	0.2882	650.3702	21.3335
20.0903	0.2319	95.2687	0.2614	651.0592	28.5488
22.0303	0.0395	98.8008	1.5368	1344.8843	16.7482
22.558	0.0421	99.7023	0.9187	1346.0654	7.2863
23.1714	0.0288	113.9932	1.1116	1356.0943	0.127
24.5584	0.1049	116.6856	2.1283	1360.3208	0.6708
25.5607	0.0005	132.9704	4.7919	1366.8513	9.2758
28.1565	0.083	583.2059	0.0557	1369.3372	3.6307
35.4771	0.6226	583.9054	54.4722	2401.9658	402.6226
47.4411	0.0048	597.8453	0.0589	2408.5841	1111.0798
55.7114	0.0404	603.0617	39.6479	2410.1599	705.1238
57.1329	0.0263	629.52	16.7403	2413.2035	3292.4872
65.7778	0.4087	632.3325	13.6197	2424.4803	2370.6823
75.2968	0.0371	633.0611	12.7345	2447.9963	6.0336
81.4598	0.1812	637.517	0.0125		

Table S46. Cartesian coordinates for the optimized geometry of isomer 6f-sextet $U^{+}(CO_2)_6$ followed by its predicted frequencies (cm^{-1}) and IR intensities (km/mol).

Z	x	y	z
92	0.075915000	0.179798000	0.160154000
8	1.768513000	-1.927935000	-0.285289000
6	2.590349000	-2.762353000	-0.235487000
8	3.393486000	-3.580796000	-0.192174000
8	2.535151000	1.169113000	-0.098922000
6	3.281341000	2.066325000	0.030624000
8	4.023019000	2.931691000	0.152604000
8	-2.453584000	1.053145000	0.444719000
6	-3.143095000	1.963773000	0.716443000
8	-3.832660000	2.840908000	0.980842000
8	-0.549662000	-0.136554000	-2.508046000
6	-0.824474000	0.051672000	-3.632235000
8	-1.092847000	0.229871000	-4.733680000
8	-1.652100000	-1.942585000	0.163756000
6	-2.467606000	-2.723549000	0.474945000
8	-3.265688000	-3.493305000	0.773859000
8	-0.080285000	2.351505000	-0.069172000
6	0.354430000	-0.172907000	2.507129000
8	0.490425000	-0.379952000	3.633669000

Frequency	Intensity	Frequency	Intensity	Frequency	Intensity
11.1351	0.1149	100.1186	0.4905	1367.523	22.7164
14.5266	0.3682	110.7892	3.0027	1369.37	28.1383
16.4384	0.0861	120.758	8.3388	1370.6303	18.4929
17.7854	0.053	127.1032	19.9952	1372.5707	21.2388
20.3467	0.048	148.432	3.3627	1373.2523	10.9956
21.9596	0.0915	161.465	2.5688	1972.2533	1111.9306
22.4661	2.0856	258.2873	3.675	2416.7993	312.1492
23.9985	0.066	279.137	3.7197	2420.1901	1296.8195
31.4665	0.4945	304.6499	10.4238	2422.0574	1816.5743
38.4104	4.2649	515.5642	123.5448	2423.5948	2224.1447
46.6344	3.094	644.7182	16.4461	2447.3869	90.7947
57.0309	0.2221	647.9344	2.7394		
62.4793	0.5488	648.7622	32.8827		
65.9705	0.2074	651.1352	19.5342		
68.0226	0.6256	651.9252	27.4981		
73.6256	0.1687	653.9705	10.8231		
74.5051	0.9455	656.0896	24.23		
79.3199	3.2498	656.8716	77.1635		
91.6286	1.5569	657.7225	46.4285		
99.6227	0.9089	658.7221	16.6045		

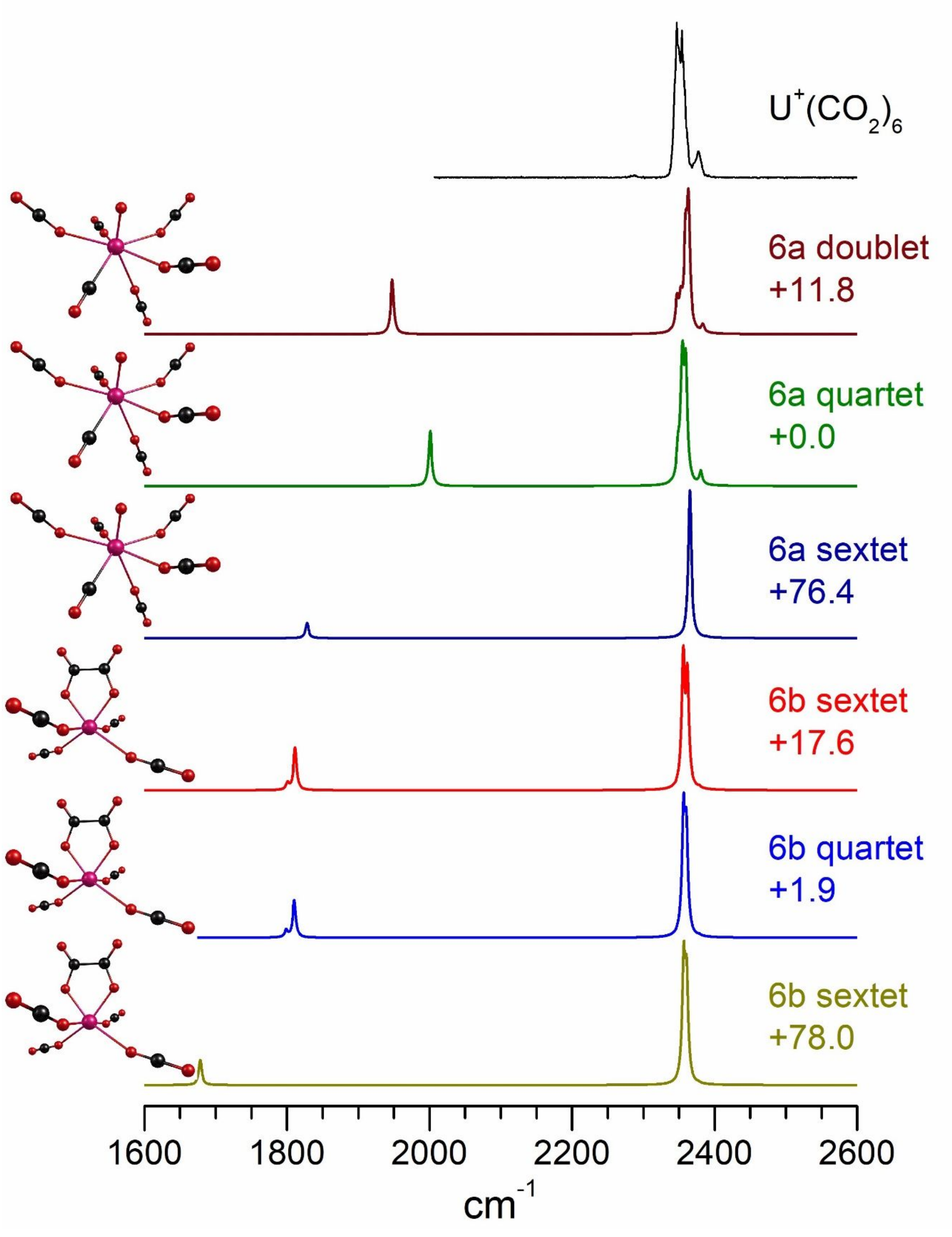

Figure S39. Experimental IR spectrum of $U^{+}(CO_2)_6$ compared with simulated spectra for isomers 6a and 6b. Relative energies (kcal/mol) are shown next to each spectrum.

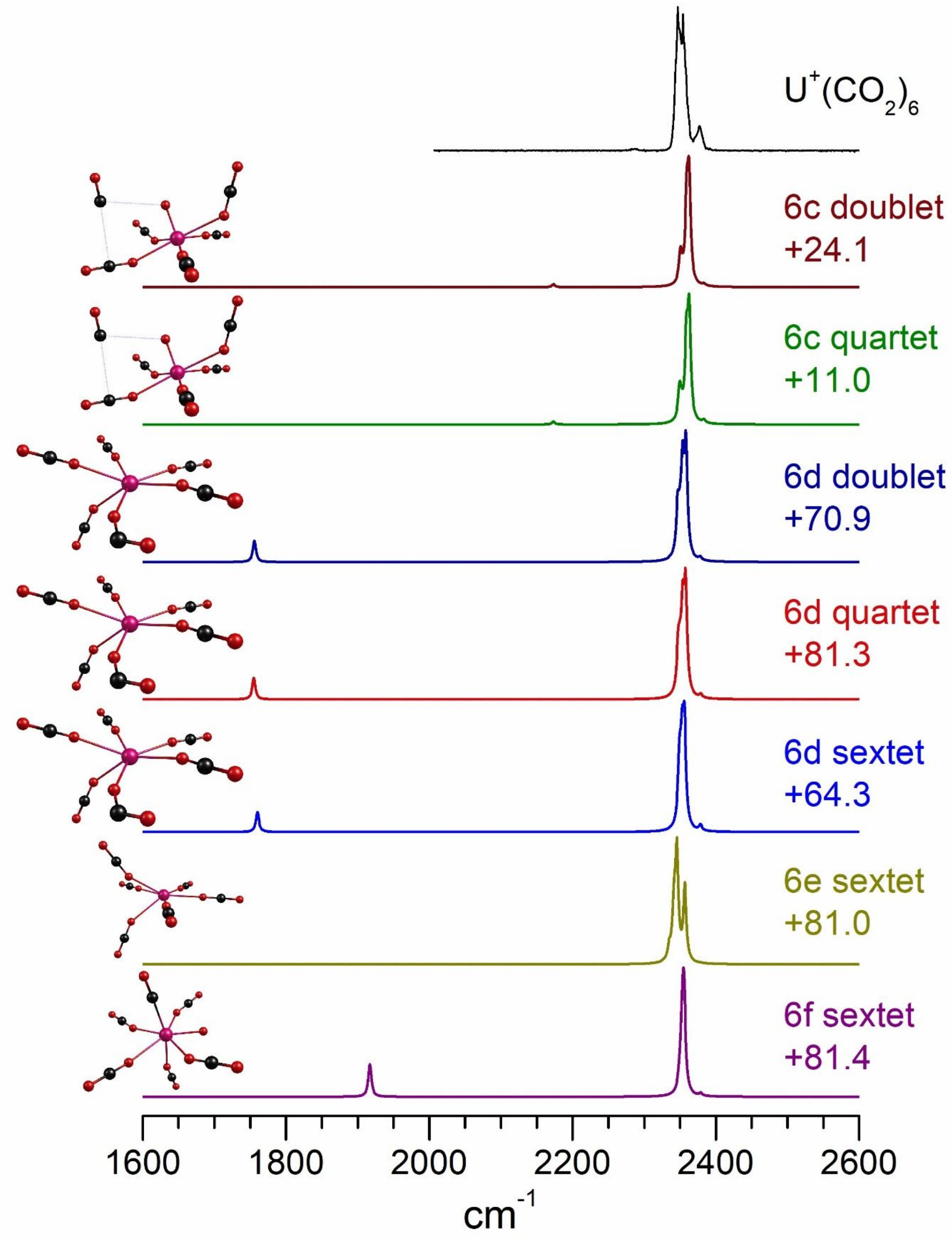

Figure S40. Experimental IR spectrum of $U^+(CO_2)_6$ compared with simulated spectra for isomers 6c–6f.. Relative energies (kcal/mol) are shown next to each spectrum.

Table S47. $U^+(CO_2)_7$ electronic energy calculated at the B3LYP/cc-pVTZ(-pp) level with Stuttgart/Koeln pseudopotential.

Isomer	2s + 1	Energy (hartree)	Rel. E (kcal/mol)	BDE (kcal/mol)	BDE (CO) (kcal/mol)	BDE (oxalate) (kcal/mol)
7a	2	-1795.162414	+15.9	7.2		78.3
7a	4	-1795.187694	+0.0	7.4		91.8
7a	6	-1795.066777	+75.9	7.6		-1.5
7b	2	-1795.163971	+14.9	2.4	12.1	
7b	4	-1795.185700	+1.3	4.2	14.5	
7b	6	-1795.056157	+82.5	-0.7		
7c	2	-1795.145671	+26.4	3.2	13.1	
7c	4	-1795.167022	+13.0	3.5	13.2	
7d	2	-1795.055834	+82.7	-6.4		
7d	4	-1795.083517	+65.4	21.4		
7d	6	-1795.083299	+65.5	4.2		
7e	6	-1795.058123	+81.3	0.6		

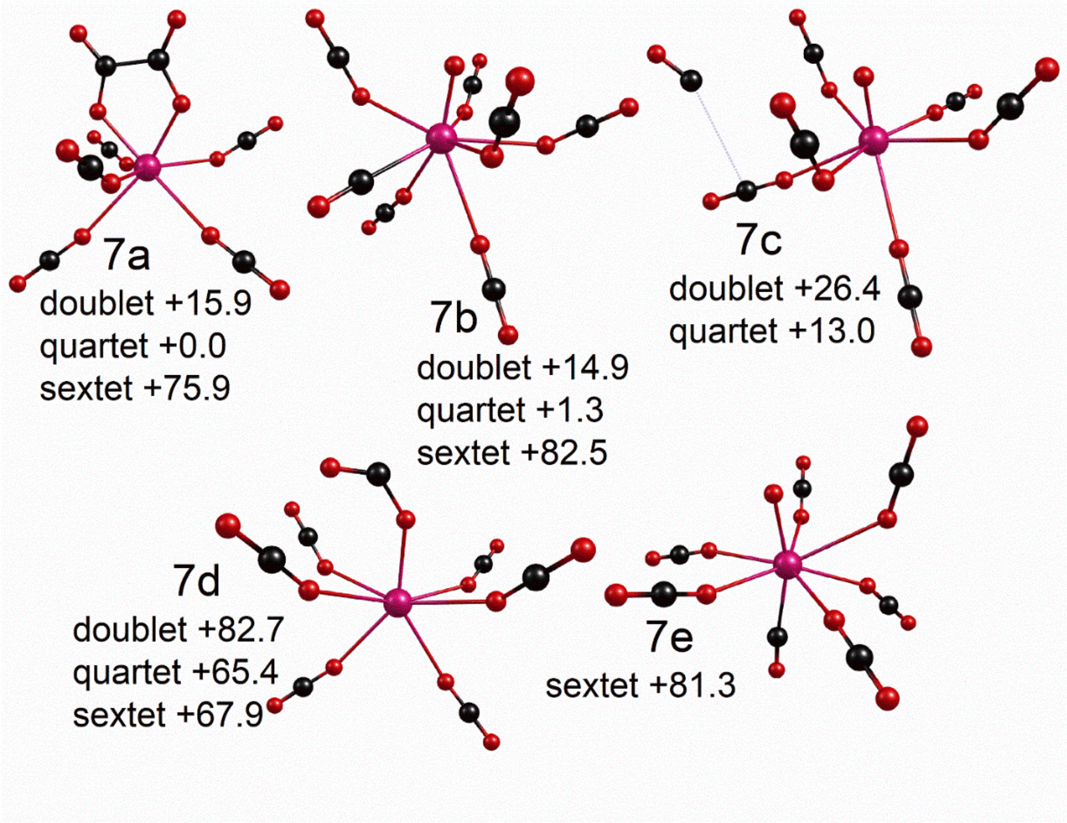

Figure S41. Predicted minimum energy structures of $U^+(CO_2)_7$ with energy of each spin state in kcal/mol. The lowest energy spin state of each isomer is shown.

Table S48. Cartesian coordinates for the optimized geometry of isomer 7a-doublet $U^+(CO_2)_7$ followed by its predicted frequencies (cm^{-1}) and IR intensities (km/mol).

Z	x	y	z
92	-0.057951000	-0.127862000	-0.167131000
8	-2.123947000	0.798439000	1.213609000
6	-2.619388000	1.366093000	2.115267000
8	-3.118058000	1.915538000	2.987361000
8	0.849351000	-0.455444000	1.762823000
6	1.498040000	0.514070000	2.455491000
8	1.987985000	0.386821000	3.529566000
8	1.982822000	-0.132886000	-1.864811000
6	2.989803000	0.254485000	-2.329761000
8	3.969668000	0.623166000	-2.793889000
8	0.942141000	-2.632678000	-0.319207000
6	1.540084000	-3.449758000	0.276821000
8	2.120604000	-4.259362000	0.842933000
8	-2.262990000	-1.737799000	-0.580795000
6	-3.189236000	-2.445561000	-0.709340000
8	-4.094851000	-3.138302000	-0.837348000
8	-1.166851000	1.513780000	-2.057031000
6	-1.379002000	2.541092000	-2.584284000
8	-1.592010000	3.539135000	-3.106573000
8	0.866423000	1.715099000	0.463431000
6	1.517981000	1.859919000	1.643022000
8	2.037440000	2.854647000	2.031521000

Frequency	Intensity	Frequency	Intensity	Frequency	Intensity
6.2618	0.9454	87.6693	0.5939	650.7365	18.4172
10.5671	0.0399	91.2648	1.5613	651.7735	25.1497
15.5511	1.9528	99.3877	0.1022	653.3648	13.7421
18.6269	1.8783	105.1404	2.4822	654.1368	21.1724
19.2797	0.1484	112.7779	3.1376	654.853	34.3559
21.0418	0.632	124.5461	8.6996	655.4334	16.6643
21.819	1.719	143.467	2.3508	655.9197	65.0875
24.1877	0.0437	147.7243	17.2538	658.1551	73.4298
27.5536	1.6788	151.8101	7.3953	658.5143	32.5087
28.956	0.1728	155.8927	8.7081	776.123	86.6914
51.0976	0.0539	293.3108	40.6466	831.4707	0.0951
56.2617	0.1921	306.6229	22.1819	857.5603	234.955
59.8087	0.6515	333.9988	9.4977	1001.7179	42.5807
65.8168	0.0465	485.1453	41.3052	1187.3278	676.0167
68.7514	0.0647	542.5682	55.632	1368.0938	33.4685
70.3142	0.2034	549.5716	2.4819	1369.51	20.772
82.0301	0.3829	649.5678	4.0326	1370.7054	20.4244

1372.0504	61.539	1854.815	770.6219	2423.2826	2061.4656
1373.3132	5.7323	2418.5429	376.8754	2429.0804	1987.3305
1843.4812	105.6257	2421.0144	673.4152	2449.2053	32.2658

Table S49. Cartesian coordinates for the optimized geometry of isomer 7a-quartet $U^+(CO_2)_7$ followed by its predicted frequencies (cm^{-1}) and IR intensities (km/mol).

Z	x	y	z
92	0.003729000	0.000140000	-0.221812000
8	-1.790794000	-1.534425000	-1.496615000
6	-2.588246000	-2.395765000	-1.450178000
8	-3.367136000	-3.235331000	-1.422402000
8	-0.706883000	-1.264989000	1.378613000
6	-1.207035000	-0.788246000	2.544565000
8	-1.591950000	-1.445778000	3.455520000
8	2.270794000	-0.000230000	1.180204000
6	2.867147000	-0.000450000	2.192344000
8	3.463929000	-0.000665000	3.169858000
8	1.655482000	-1.904279000	-1.332593000
6	2.309662000	-2.833204000	-1.624726000
8	2.950141000	-3.739222000	-1.915036000
8	1.655771000	1.904978000	-1.331647000
6	2.310313000	2.833619000	-1.623864000
8	2.951138000	3.739371000	-1.914246000
8	-1.790540000	1.536878000	-1.494496000
6	-2.587718000	2.398379000	-1.446306000
8	-3.366344000	3.238131000	-1.416836000
8	-0.707092000	1.263207000	1.380168000
6	-1.207148000	0.784943000	2.545538000
8	-1.592130000	1.441291000	3.457319000

Frequency	Intensity	Frequency	Intensity	Frequency	Intensity
5.6674	0.6971	86.4306	0.299	651.3254	14.2207
13.2864	0.0736	91.3689	0.8076	653.4606	40.7069
15.7317	2.0516	100.4969	0.1659	654.5386	19.3235
18.3677	0.3015	107.8998	1.0325	655.0679	14.7329
20.6456	1.8464	110.7407	4.6896	655.6261	39.129
20.8021	0.3116	117.6983	9.692	656.5384	36.7667
21.1708	1.4961	145.341	7.2651	656.7715	2.1354
22.9851	0	148.0417	3.2037	658.1726	55.5982
27.304	0.7272	154.025	20.5859	658.8003	79.3627
28.8432	1.3978	157.7884	5.6258	775.31	87.8806
49.2769	0.0547	295.6262	41.8971	832.2022	0.1252
55.8305	0.1091	307.283	21.4765	857.2878	230.77
59.1805	0.726	334.1948	8.0285	1004.303	45.7209
63.8478	0.0002	485.5826	41.7931	1188.5114	666.7613
69.5797	0.0006	544.6699	52.6252	1370.3743	29.0599
70.3854	0.2543	549.7882	1.4573	1371.5493	49.3282
80.4476	0.1791	650.3764	3.4781	1371.974	26.5864

1372.0354	33.3961	1854.3469	770.4588	2425.6553	2173.5107
1373.8167	10.0102	2419.7183	72.1273	2428.6966	1870.2163
1843.1311	102.6487	2421.6545	851.1496	2450.0818	57.7229

Table S50. Cartesian coordinates for the optimized geometry of isomer 7a-sextet $U^+(CO_2)_7$ followed by its predicted frequencies (cm^{-1}) and IR intensities (km/mol).

Z	x	y	z
92	-0.009151000	-0.089030000	-0.260723000
8	2.286344000	0.267210000	1.047963000
6	2.785621000	0.576617000	2.065641000
8	3.295258000	0.875273000	3.047267000
8	-0.650677000	1.423040000	1.096726000
6	-1.046979000	1.201501000	2.416789000
8	-1.384624000	2.070017000	3.181522000
8	-1.885817000	-1.798367000	-1.122894000
6	-2.739416000	-2.597081000	-1.017121000
8	-3.572443000	-3.379013000	-0.926433000
8	-1.857172000	1.268238000	-1.669151000
6	-2.601283000	2.172457000	-1.568988000
8	-3.332076000	3.051254000	-1.493094000
8	1.531615000	1.595279000	-1.772078000
6	2.164850000	2.461054000	-2.246654000
8	2.785165000	3.304359000	-2.715310000
8	1.672976000	-2.161601000	-0.962680000
6	2.379428000	-3.095453000	-1.029408000
8	3.070232000	-4.008647000	-1.099633000
8	-0.560774000	-1.027647000	1.657587000
6	-0.960146000	-0.230169000	2.648286000
8	-1.279328000	-0.822250000	3.777110000

Frequency	Intensity	Frequency	Intensity	Frequency	Intensity
6.2394	0.6263	84.4257	0.3476	563.4329	25.9433
12.886	0.0632	87.6244	0.5138	597.4581	27.2433
15.5076	1.7755	96.1101	0.265	643.1104	12.1742
18.5589	0.484	97.3843	0.0874	650.4567	0.9108
19.4145	2.2459	110.233	4.5778	651.6003	17.6727
21.204	0.8186	114.7526	5.2207	652.6249	27.9831
22.429	0.0758	133.2944	5.7396	652.9258	4.707
26.6693	0.2021	148.9715	21.4148	654.5371	42.1357
27.2654	1.2946	156.1683	14.7018	655.5912	17.4064
30.0815	0.3158	158.5705	0.3505	656.0455	70.4333
51.653	0.1329	172.8917	1.3945	657.0043	27.8355
56.8304	0.0328	254.3749	15.4118	657.6537	43.6023
63.1334	0.0409	283.2081	7.1739	658.9683	57.2647
67.9743	0.1854	299.9964	16.5919	835.1744	210.6487
70.7884	0.1394	305.7443	14.0263	970.4417	169.6689
80.3228	0.3526	510.5539	28.6871	1182.8869	32.2721

1270.622	160.1563	1372.33	42.9293	2419.9516	664.4391
1368.2464	35.1797	1373.2716	6.206	2424.9345	1897.6149
1368.9231	28.6637	1720.9858	475.6445	2427.5089	2028.9084
1371.5568	21.7183	2418.9875	337.6678	2447.5355	49.1508

Table S51. Cartesian coordinates for the optimized geometry of isomer 7b-doublet $U^{+}(CO_2)_7$ followed by its predicted frequencies (cm^{-1}) and IR intensities (km/mol).

Z	x	y	z
92	0.008285000	-0.132976000	-0.071252000
8	2.450942000	1.016652000	0.095082000
6	3.605196000	1.215171000	0.108400000
8	4.735015000	1.416167000	0.123603000
8	-0.059106000	1.513963000	2.162704000
6	-0.044317000	1.181953000	3.288637000
8	-0.030448000	0.880711000	4.395620000
8	-1.984800000	-1.994181000	-0.606030000
6	-2.223626000	-3.040430000	-0.133152000
8	-2.468237000	-4.068956000	0.314651000
8	-2.497795000	0.875740000	0.069406000
6	-3.664895000	0.970336000	0.038125000
8	-4.807845000	1.070682000	0.010485000
8	2.074273000	-1.848320000	-0.718162000
6	2.433080000	-2.893705000	-0.326759000
8	2.794406000	-3.919482000	0.041239000
8	0.063518000	-1.308286000	1.356227000
6	-0.058544000	-0.294877000	-2.512044000
8	-0.090310000	-0.281477000	-3.656034000
6	-0.113119000	3.759856000	-1.136098000
8	-0.162526000	4.840755000	-1.526137000
8	-0.062693000	2.661527000	-0.738588000

Frequency	Intensity	Frequency	Intensity	Frequency	Intensity
4.109	0.3891	73.3216	0.0771	652.1282	2.9218
11.2629	0.028	77.8262	0.3982	653.1386	1.2416
14.6774	0.7146	81.4951	1.3574	653.9678	46.627
16.5485	0.0382	83.7482	0.73	654.5301	8.9582
19.7094	0.0712	89.2367	1.2176	655.5609	22.5335
21.9619	0.1161	96.5234	3.8674	656.8646	4.5853
22.2944	0.0827	98.2915	3.7898	658.2344	48.7606
22.6879	0.7354	114.9054	5.7059	660.3248	83.2812
24.2701	0.2409	122.8283	25.5415	661.2678	41.1161
28.3194	0.0523	129.5101	5.02	662.8569	31.7281
33.6304	0.8766	134.6978	27.0318	665.2583	42.3022
35.5743	4.6854	160.0839	7.5787	666.6334	1.8365
37.4523	0.7488	189.0806	11.8791	832.7469	358.0501
44.9995	0.112	209.8856	11.4711	1365.4341	23.868
59.1179	4.7156	239.7649	8.2441	1367.2572	15.4886
61.5851	0.9997	246.6406	3.8327	1368.5095	26.0703
66.1054	0.7173	276.1871	8.2175	1375.1172	9.8414

1376.7386	42.4103	2412.1583	36.1862	2422.371	2471.9182
1377.5961	7.7594	2414.1009	787.2693	2429.1047	2201.2251
2023.9928	997.6996	2417.8729	185.7389	2451.4088	101.0994

Table S52. Cartesian coordinates for the optimized geometry of isomer 7b-quartet $U^{+}(CO_2)_7$ followed by its predicted frequencies (cm^{-1}) and IR intensities (km/mol).

Z	x	y	z
92	0.001769000	0.073641000	-0.259924000
8	-1.583626000	1.248321000	1.667199000
6	-2.190746000	2.183292000	2.027061000
8	-2.789672000	3.093201000	2.390077000
8	1.590579000	1.245339000	1.666436000
6	2.187376000	2.192965000	2.010456000
8	2.776521000	3.115083000	2.358231000
8	2.538715000	0.085173000	-1.225449000
6	3.094694000	0.696341000	-2.059034000
8	3.655538000	1.282795000	-2.869737000
8	1.630588000	-1.845063000	1.134577000
6	2.398965000	-2.635619000	1.524387000
8	3.153045000	-3.413055000	1.911253000
8	-2.530277000	0.113774000	-1.236431000
6	-3.075972000	0.733545000	-2.070463000
8	-3.627051000	1.328459000	-2.881692000
8	0.012201000	1.744654000	-1.046179000
6	-0.003305000	-1.772472000	-1.897963000
8	-0.006265000	-2.654398000	-2.621892000
6	-2.422880000	-2.637507000	1.488867000
8	-3.176578000	-3.423932000	1.857974000
8	-1.655156000	-1.837635000	1.117272000

Frequency	Intensity	Frequency	Intensity	Frequency	Intensity
12.882	0.1904	77.4446	0.1218	655.255	4.9715
17.1032	0.2237	77.9446	0.0765	655.4458	3.6125
17.6715	0.0046	79.2254	0.0044	657.2283	4.5297
18.7934	0.4096	86.0098	0.1389	657.6457	96.7369
20.6419	0.5417	90.7886	4.4166	658.4329	7.4972
21.3161	0.2052	95.2086	0.5173	659.9971	7.3866
21.5665	0.0092	100.5162	0.6663	660.4138	24.3742
25.8947	0.3921	124.6995	19.3585	661.1447	47.8916
26.853	1.0193	125.8851	2.8807	661.9935	6.1686
29.562	0.3243	141.2848	47.059	662.3167	108.1219
38.2672	2.5842	150.8395	1.0179	664.6179	6.6692
49.1067	0.0131	162.0111	2.0848	835.2658	322.794
54.7694	0.0966	191.4776	7.8825	1368.2519	18.9075
55.1138	0.8673	238.266	5.8584	1368.7219	30.8883
69.6043	1.3447	271.9861	1.9031	1373.1514	8.633
69.7709	1.9955	303.7031	0.5611	1373.9121	38.933
71.2043	0.1439	654.3811	0.0845	1374.5493	18.4818

1375.3077	7.7844	2416.2571	0.2283	2424.7937	2046.3281
2053.9071	992.904	2418.5293	1404.7578	2450.8753	220.1078
2413.7223	22.1736	2424.286	2166.0018		

Table S53. Cartesian coordinates for the optimized geometry of isomer 7c-doublet $U^+(CO_2)_7$ followed by its predicted frequencies (cm^{-1}) and IR intensities (km/mol).

Z	x	y	z
92	0.116839000	-0.084286000	0.182131000
8	2.035479000	0.571669000	-1.743105000
6	1.803504000	0.467611000	-2.889443000
8	1.604842000	0.376474000	-4.016193000
8	2.017937000	-2.001605000	0.169449000
6	2.845877000	-2.829146000	0.147998000
8	3.657054000	-3.641597000	0.127957000
8	-2.032677000	0.503291000	1.633756000
6	-3.136359000	0.659777000	1.994946000
8	-4.207651000	0.819360000	2.373902000
8	-1.029317000	-2.413927000	0.870649000
6	-1.732125000	-3.192575000	0.344244000
8	-2.419427000	-3.965871000	-0.151414000
8	-0.249895000	2.582358000	0.015301000
6	-0.608325000	3.575734000	-0.492375000
8	-0.955752000	4.553830000	-0.982510000
8	-0.739746000	-0.356025000	-1.445222000
6	-4.163633000	0.093671000	-1.048296000
8	-4.607648000	-0.013915000	-2.074367000
6	3.397515000	1.776246000	1.667267000
8	4.281137000	2.313383000	2.172624000
8	2.497177000	1.228377000	1.161405000

Frequency	Intensity	Frequency	Intensity	Frequency	Intensity
4.6929	0.0037	66.1748	0.039	640.1201	24.0891
6.5303	0.0757	69.6239	0.5521	644.6737	13.1327
11.4023	0.0913	72.5158	0.456	645.8627	10.868
15.3055	0.1234	73.5792	1.2202	647.3368	22.0568
18.6247	0.0452	79.3493	0.177	648.5857	31.8735
19.9886	0.095	83.4138	1.3117	649.3905	44.9209
21.0246	0.6584	90.3034	0.2199	650.4622	46.3671
22.0524	0.5821	94.732	0.2652	653.6674	50.1809
22.7528	0.1371	97.9849	1.824	655.4023	42.2899
24.4674	0.6413	107.8173	1.1302	657.3491	30.5019
28.0282	0.3314	118.5788	5.0167	658.0044	16.5005
31.4509	1.4159	127.2749	1.9139	664.5475	24.8778
35.9362	0.188	134.7617	8.8587	818.2914	377.1953
44.5083	0.7347	136.3454	23.9596	1362.6296	17.1521
49.9744	1.9583	142.6225	24.2915	1367.1603	17.3138
55.6002	0.2703	164.9749	2.7472	1370.199	4.1723
62.0963	0.6554	192.6538	7.74	1373.2331	14.3069

1374.3201	20.3723	2409.9369	748.0023	2424.9249	1704.7813
1376.1053	9.6488	2416.5622	520.5471	2429.355	2374.0239
2236.1717	65.27	2417.452	521.3814	2451.9485	47.2711

Table S54. Cartesian coordinates for the optimized geometry of isomer 7c-quartet $U^{+}(CO_2)_7$ followed by its predicted frequencies (cm^{-1}) and IR intensities (km/mol).

Z	x	y	z
92	0.356569000	-0.101608000	0.161016000
8	-0.480332000	2.279611000	-1.048510000
6	-0.916506000	2.256554000	-2.138536000
8	-1.348069000	2.269187000	-3.201913000
8	2.765544000	0.221471000	-1.018687000
6	3.157535000	0.073713000	-2.115423000
8	3.561069000	-0.062953000	-3.180204000
8	-1.285110000	-2.165378000	0.730195000
6	-1.934521000	-2.753085000	-0.052732000
8	-2.580569000	-3.344570000	-0.792856000
8	1.883122000	-2.201388000	0.890989000
6	2.490383000	-3.147093000	1.220347000
8	3.087443000	-4.072203000	1.545007000
8	-1.838684000	0.881600000	1.407049000
6	-2.868960000	1.207828000	1.857001000
8	-3.873063000	1.534208000	2.308359000
8	-0.164531000	-0.584939000	-1.573743000
6	-4.751110000	-0.359265000	-0.508160000
8	-5.679944000	-0.495017000	-1.124375000
6	1.991249000	3.261383000	1.640934000
8	2.458348000	4.229362000	2.050579000
8	1.518178000	2.274475000	1.228857000

Frequency	Intensity	Frequency	Intensity	Frequency	Intensity
5.0795	0.1129	63.6311	0.1528	646.6455	28.4581
8.0339	0.3275	67.729	1.0063	648.0788	22.893
10.1434	0.0827	71.0519	0.0858	649.8088	14.1162
14.8037	0.0089	78.4503	0.5357	650.3285	20.1534
16.7143	0.5432	79.2193	1.4389	651.4251	6.6433
18.7267	0.0023	84.1874	1.5095	653.249	48.207
21.2715	0.1032	91.8737	0.6234	654.4117	84.4498
23.2543	0.3799	95.871	0.3643	656.1122	27.6075
25.2395	0.0874	96.6896	0.1597	658.6195	62.5963
26.9101	0.7135	101.7501	1.1054	659.4686	1.6743
29.0799	0.9654	110.8589	0.7389	660.6379	46.6401
31.574	2.4834	114.2346	0.1784	666.3303	26.3273
44.026	0.3112	139.4642	33.6813	804.475	309.4895
44.785	0.1	145.5085	41.2917	1362.4657	14.0723
46.8214	1.5332	167.741	1.2694	1363.9558	20.5748
48.5365	2.2588	180.1272	3.6012	1367.5613	24.9586
56.643	0.2978	201.8874	6.802	1372.4764	8.7572

1376.2494	22.2852	2408.9168	312.6258	2423.5228	1389.626
1376.7695	8.7935	2413.9258	447.7787	2428.8854	1968.4814
2238.6346	71.7132	2417.3179	1510.3587	2450.6025	44.4968

Table S55. Cartesian coordinates for the optimized geometry of isomer 7d-doublet $U^+(CO_2)_7$ followed by its predicted frequencies (cm^{-1}) and IR intensities (km/mol).

Z	x	y	z
92	0.147613000	0.117296000	0.053566000
8	-2.126975000	0.161071000	1.458365000
6	-3.011487000	-0.395925000	1.994267000
8	-3.870832000	-0.907731000	2.555264000
8	-0.436585000	-2.009857000	-0.045300000
6	-1.451949000	-2.825135000	-0.016864000
8	-2.621866000	-2.582631000	-0.002650000
8	2.174083000	-0.858132000	-1.505767000
6	2.517582000	-1.617860000	-2.329761000
8	2.853337000	-2.350164000	-3.148236000
8	-2.113428000	0.248401000	-1.451318000
6	-2.934149000	-0.318900000	-2.069974000
8	-3.729893000	-0.846273000	-2.706756000
8	2.237335000	1.832798000	0.026414000
6	3.227769000	2.458909000	0.015607000
8	4.193151000	3.079712000	0.005496000
8	-0.882840000	2.590863000	0.026627000
6	-1.544154000	3.558234000	0.016083000
8	-2.180937000	4.513056000	0.006055000
8	2.197162000	-0.973995000	1.429959000
6	2.622796000	-1.779559000	2.167741000
8	3.040931000	-2.555841000	2.903013000

Frequency	Intensity	Frequency	Intensity	Frequency	Intensity
6.7211	0.298	81.3051	0.3844	627.2117	8.9022
16.9349	0.2776	84.2005	0.6594	627.6314	66.8204
17.4501	0.4743	87.2761	0.2352	630.8901	27.0501
18.6006	0.232	90.0151	2.3457	638.6693	11.0495
20.2627	0.0754	93.3996	1.0456	641.1678	10.0208
20.5528	0.2801	107.6077	1.1149	643.4318	5.7373
22.4438	0.3998	109.1337	0.4308	644.3553	53.7029
26.6567	0.0258	111.7693	2.2043	644.8501	44.5215
32.9393	0.0105	114.1543	2.884	646.341	48.3502
36.6246	0.0198	120.4212	1.1546	648.4276	27.0583
44.7746	2.2237	123.8398	3.5884	650.5252	40.2308
48.6318	0.9974	126.5523	0.4178	706.7465	2.6962
57.0709	1.7285	129.2348	1.265	1154.8178	270.1123
57.8736	1.2369	141.5799	16.506	1362.3597	10.0981
68.6437	1.2562	150.8025	0.3572	1363.1934	10.0908
70.4216	0.4861	365.8459	67.9458	1366.7133	11.9049
76.1291	1.2119	624.4142	1.8987	1368.2088	10.2469

1371.9425	11.5797	2410.5347	458.8189	2422.7895	2159.118
1372.9607	11.2909	2415.3324	1397.7627	2426.3484	2424.9121
1774.6637	452.9627	2418.2649	59.1113	2448.389	14.0897

Table S56. Cartesian coordinates for the optimized geometry of isomer 7d-quartet $U^+(CO_2)_7$ followed by its predicted frequencies (cm^{-1}) and IR intensities (km/mol).

Z	x	y	z
92	0.148600000	0.123457000	-0.034860000
8	-2.140353000	0.211978000	1.430312000
6	-2.965808000	-0.382909000	2.016844000
8	-3.768550000	-0.932099000	2.625155000
8	-0.418661000	-2.018085000	0.044620000
6	-1.443215000	-2.822013000	-0.024886000
8	-2.611118000	-2.564928000	-0.024167000
8	2.201466000	-1.058255000	-1.359603000
6	2.571727000	-1.964864000	-2.004182000
8	2.938484000	-2.840779000	-2.649568000
8	-2.087592000	0.152848000	-1.501752000
6	-2.969867000	-0.400030000	-2.045696000
8	-3.826876000	-0.907362000	-2.614147000
8	2.240138000	1.842273000	-0.188750000
6	3.219110000	2.467928000	-0.340807000
8	4.173249000	3.088153000	-0.489862000
8	-0.927392000	2.594273000	0.017037000
6	-1.600537000	3.550878000	0.087465000
8	-2.249132000	4.495077000	0.156013000
8	2.181440000	-0.734935000	1.567618000
6	2.607095000	-1.398154000	2.434953000
8	3.022116000	-2.036040000	3.295212000

Frequency	Intensity	Frequency	Intensity	Frequency	Intensity
6.9497	0.266	78.1721	0.1146	626.6842	31.6575
16.4047	0.1115	83.6079	0.8139	630.324	61.2383
17.0019	0.5532	90.0332	1.5749	632.0747	13.2434
18.0128	0.3805	90.8869	0.4576	640.2745	9.8197
19.5719	0.0594	94.9335	0.636	643.309	12.4166
20.3508	0.2377	106.0482	0.991	644.398	27.0198
22.5035	0.4323	111.0346	3.4941	646.3149	38.033
26.9015	0.0186	112.3079	1.236	646.9012	36.0646
33.4689	0.0268	119.523	1.7999	649.2985	47.4077
38.2892	0.0251	120.1027	1.0045	649.8601	34.2378
47.3565	1.431	125.5277	2.906	653.0339	32.8336
50.6504	2.1944	130.0637	3.3069	706.0926	1.745
57.5537	1.2444	139.4006	2.629	1135.6247	222.0781
58.1662	0.9051	141.7855	13.7813	1362.2835	10.4959
68.4026	0.0339	151.4062	0.7304	1363.0537	10.6376
69.8818	2.4999	361.2352	59.6619	1367.0317	12.6674
75.1343	0.4656	625.9692	4.2487	1368.404	9.7647

1373.5271	14.823	2410.0385	655.9155	2422.758	2079.5151
1374.3472	16.056	2415.1895	1156.4909	2427.2838	2347.181
1768.1618	427.1916	2418.8255	225.3671	2448.3875	13.2456

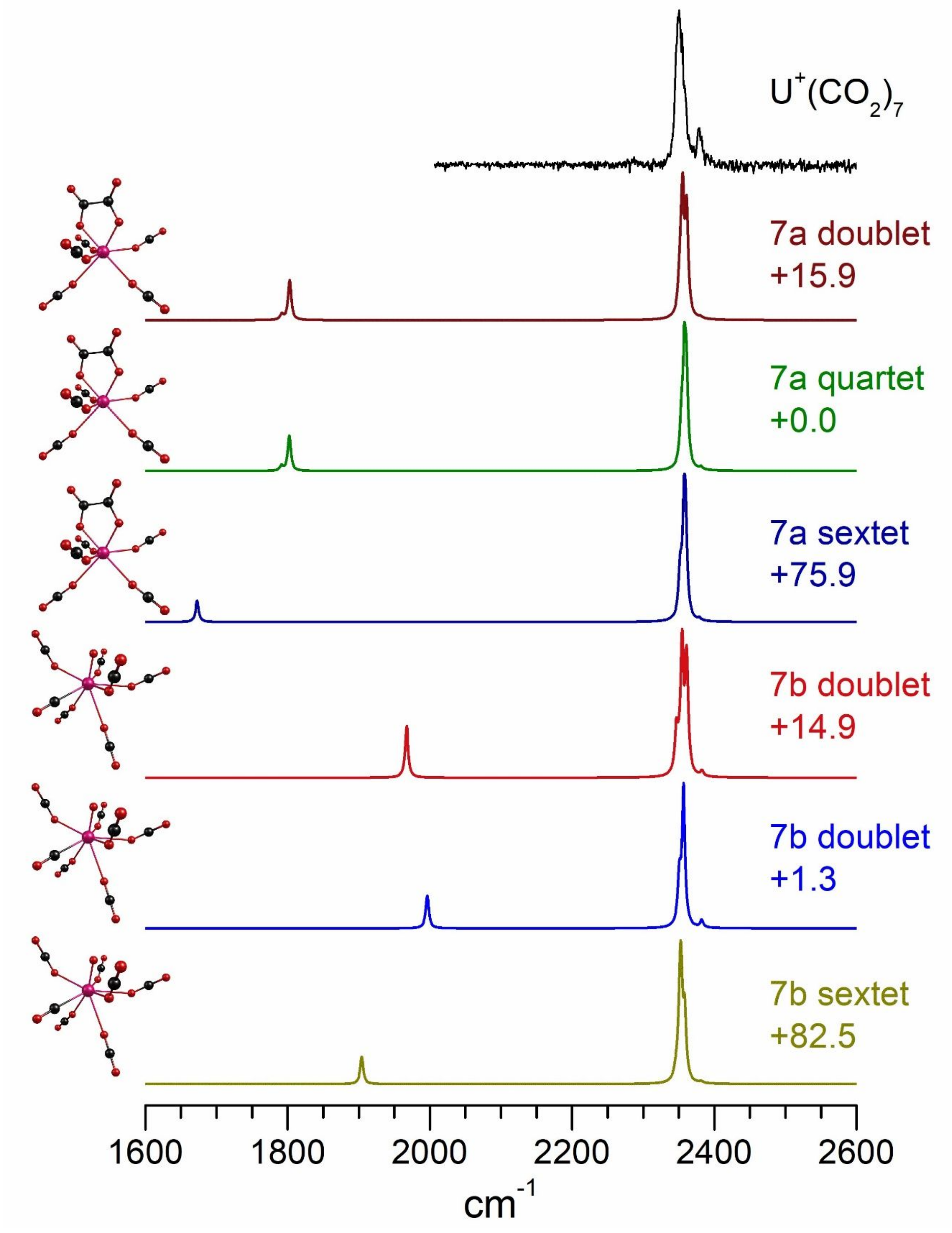

Figure S42. Experimental IR spectrum of $U^+(CO_2)_7$ compared with simulated spectra for isomers 7a and 7b. Relative energies (kcal/mol) are shown next to each spectrum.

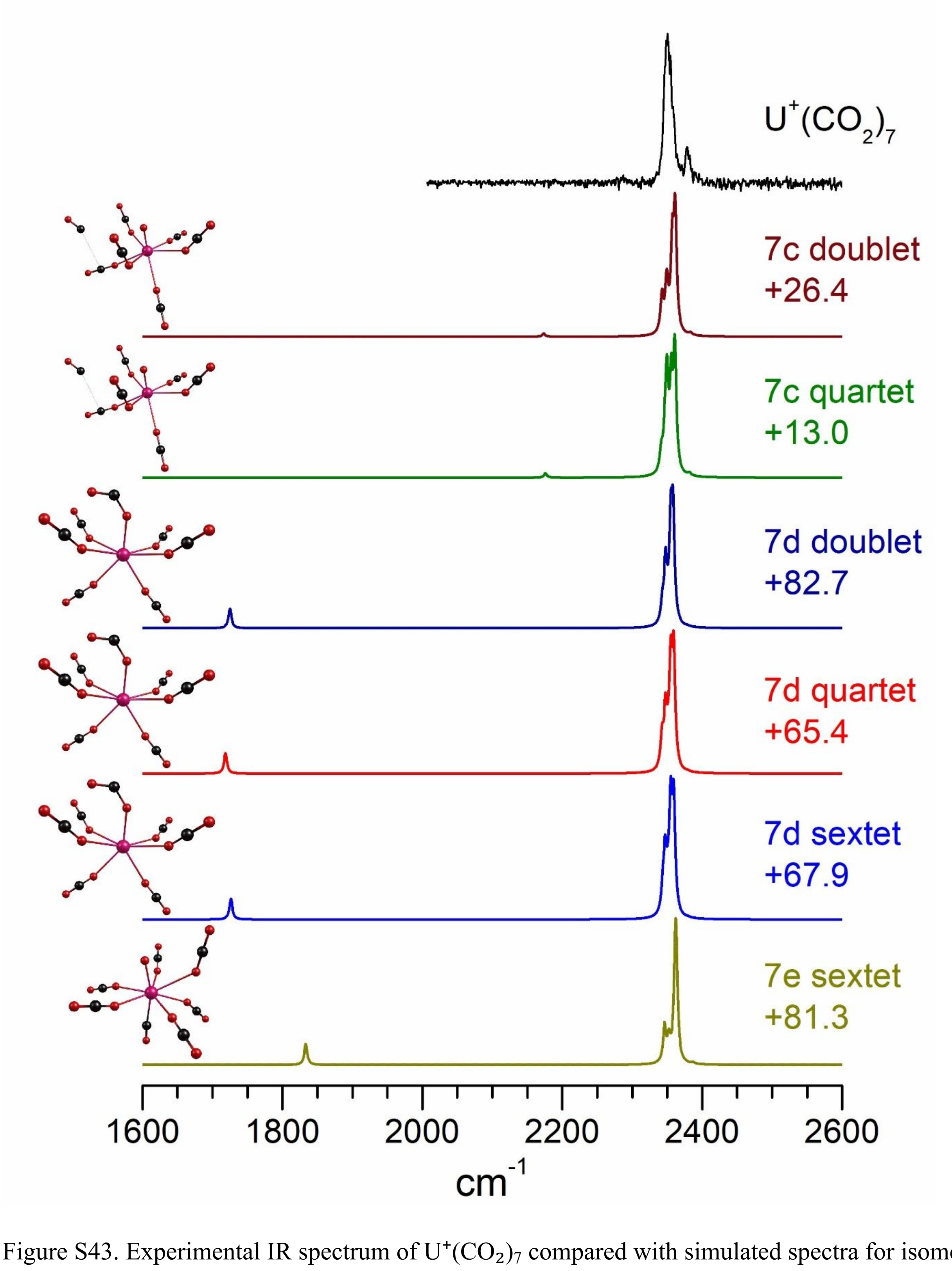

Figure S43. Experimental IR spectrum of $U^{+}(CO_2)_7$ compared with simulated spectra for isomers 7c–7e. Relative energies (kcal/mol) are shown next to each spectrum.

Table S57. $U^+(CO_2)_8$ electronic energy calculated at the B3LYP/cc-pVTZ(-pp) level with Stuttgart/Koeln pseudopotential.

Isomer	2s + 1	Energy (hartree)	Rel. E (kcal/mol)	BDE (CO_2) (kcal/mol)	BDE (CO) (kcal/mol)	BDE (oxalate) (kcal/mol)
8a	2	-1983.795118	+31.9	-10.1		50.4
8a	4	-1983.845936	+0.0	5.9		92.6
8a	6	-1983.757704	+55.4	26.4		20.2
8b	2	-1983.824028	+13.7	7.0	17.2	
8b	4	-1983.839214	+4.2	2.9	12.9	
8c	2	-1983.799181	+29.3	2.9		
8c	4	-1983.820986	+15.7	3.2		
8d	2	-1983.74911	+60.8	27.9		
8d	4	-1983.729999	+72.8	-1.5		
8d	6	-1983.736437	+68.7	2.7		
8e	6	-1983.711334	+84.5	2.7		

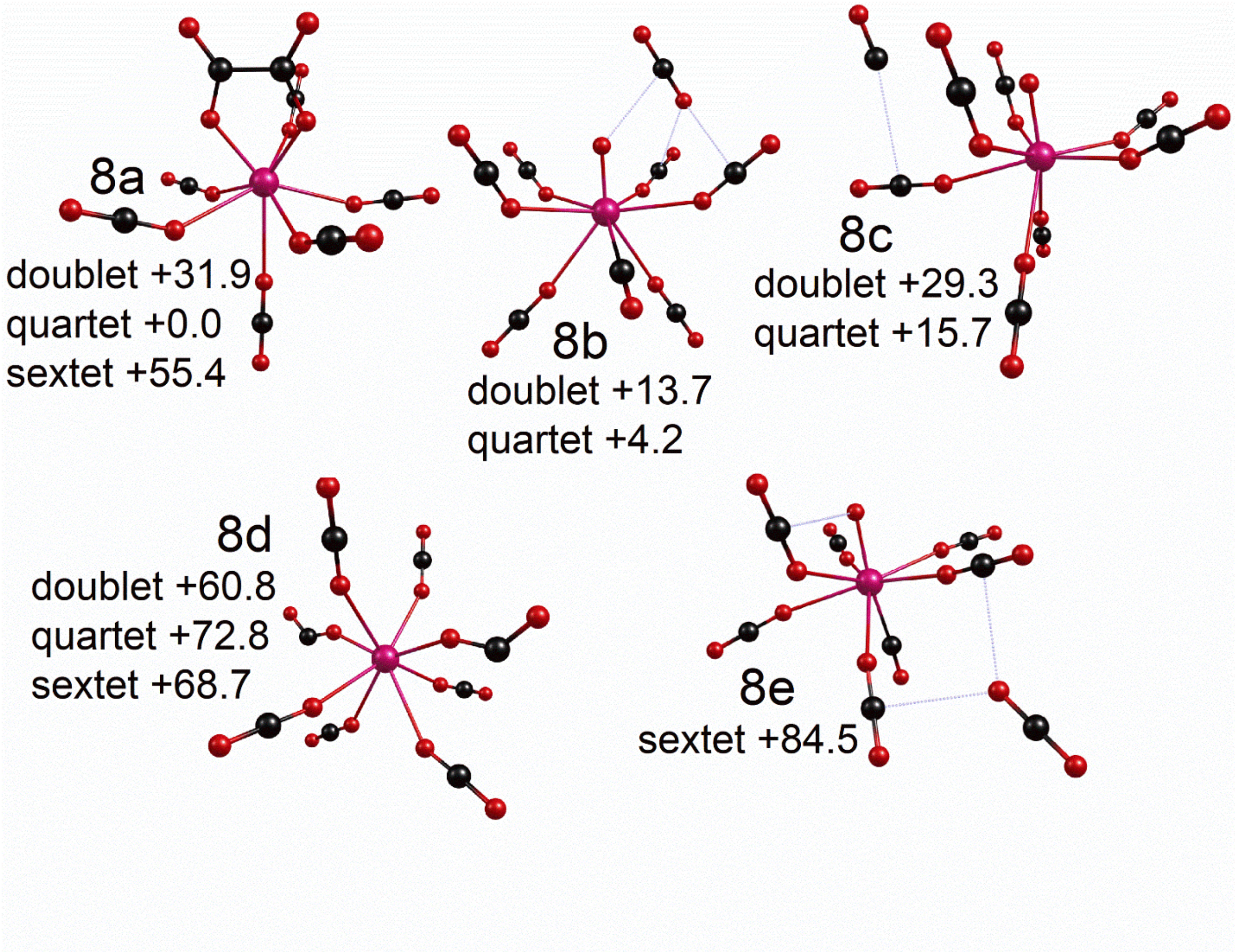

Figure S44. Predicted minimum energy structures of $U^{+}(CO_2)_8$ with energy of each spin state in kcal/mol. The lowest energy spin state of each isomer is shown.

Table S58. Cartesian coordinates for the optimized geometry of isomer 8a-doublet $U^+(CO_2)_8$ followed by its predicted frequencies (cm^{-1}) and IR intensities (km/mol).

Z	x	y	z
92	0.033804000	-0.019634000	-0.100681000
8	1.851958000	-0.094113000	-2.000328000
6	2.393928000	-0.704510000	-2.846677000
8	2.929836000	-1.283953000	-3.675032000
8	-1.916976000	-1.215174000	1.156186000
6	-2.547206000	-2.199202000	1.303208000
8	-3.166663000	-3.142002000	1.488011000
8	-0.816966000	1.389383000	2.064419000
6	-0.546520000	1.847795000	3.110810000
8	-0.297917000	2.302108000	4.133019000
8	0.941615000	-1.873520000	0.377838000
6	2.061513000	-2.019569000	1.142780000
8	2.573603000	-3.045194000	1.433666000
8	1.733422000	0.356325000	1.118709000
6	2.582660000	-0.611857000	1.586623000
8	3.555743000	-0.416025000	2.228673000
8	0.638963000	2.462595000	-0.914228000
6	1.360192000	3.377366000	-1.064246000
8	2.053716000	4.275746000	-1.216570000
8	-2.223373000	1.583591000	-0.510662000
6	-3.279029000	1.919258000	-0.893179000
8	-4.311358000	2.256425000	-1.263097000
8	-1.090474000	-0.912945000	-1.731178000
6	-1.983353000	-1.750865000	-2.186038000
8	-2.875513000	-2.311268000	-1.646554000

Frequency	Intensity	Frequency	Intensity	Frequency	Intensity
10.7027	0.1242	64.7573	1.3865	162.4018	2.7196
14.6877	0.5495	77.3775	0.3338	173.8416	11.6494
18.7878	0.3671	79.8395	0.5314	177.95	2.9418
20.7782	0.3101	84.4018	2.3734	211.954	6.2898
22.4736	1.3061	94.5346	0.9608	301.9608	23.7115
24.0657	0.3771	97.402	1.6971	308.063	33.084
24.7275	0.2586	105.9371	2.2081	335.768	11.8674
25.3508	0.4862	111.6812	1.6082	356.1133	63.0331
27.4204	0.405	112.4079	2.3638	481.3242	46.5234
30.1678	0.5227	115.3993	2.267	550.8498	4.3397
32.1042	3.1046	128.7346	2.8544	564.2441	71.1277
52.5058	0.5623	136.209	3.4025	634.3444	32.3417
57.8824	1.1224	146.2442	11.0218	650.8314	10.6038
60.0588	1.296	155.1578	23.8551	653.2706	8.8081

653.3617	5.6408	822.131	0.4695	1377.4886	22.6375
655.0542	34.078	854.5226	321.2809	1833.2445	680.0928
655.5446	26.6056	948.4146	59.497	1866.8128	120.9183
657.284	24.8613	1040.4061	84.6709	1876.7397	657.6952
658.6633	65.3915	1155.9742	730.6309	2421.3705	147.9293
659.3015	20.9556	1368.7286	46.3736	2423.1024	1235.7902
660.0782	67.276	1372.696	67.2213	2426.7584	1533.2623
676.9624	50.5378	1373.2366	15.8795	2430.8155	1779.763
763.2768	70.7187	1374.7436	48.0103	2450.9693	156.5668

Table S59. Cartesian coordinates for the optimized geometry of isomer 8a-quartet $U^{+}(CO_2)_8$ followed by its predicted frequencies (cm^{-1}) and IR intensities (km/mol).

Z	x	y	z
92	-0.085340000	0.000441000	-0.090208000
8	1.257331000	1.267662000	1.060634000
6	2.246366000	0.786722000	1.847057000
8	3.021359000	1.442190000	2.466590000
8	-1.669615000	-1.575113000	1.457682000
6	-1.591257000	-2.415344000	2.274741000
8	-1.536064000	-3.237942000	3.070960000
8	-2.720576000	-0.011418000	-1.112066000
6	-3.856554000	-0.017895000	-1.396220000
8	-4.969763000	-0.024210000	-1.678597000
8	-1.687656000	1.542517000	1.472859000
6	-1.618488000	2.374039000	2.299598000
8	-1.572302000	3.187946000	3.105267000
8	-0.275411000	2.233970000	-1.698465000
6	-0.013283000	3.310062000	-2.083842000
8	0.235770000	4.362315000	-2.467345000
8	1.943068000	0.024152000	-1.858012000
6	3.082245000	0.032947000	-2.141858000
8	4.189931000	0.041613000	-2.435144000
8	-0.245991000	-2.217604000	-1.722681000
6	0.033131000	-3.286840000	-2.115183000
8	0.298744000	-4.332421000	-2.505741000
8	1.271977000	-1.267037000	1.043344000
6	2.255275000	-0.785489000	1.836496000
8	3.037531000	-1.440342000	2.447513000

Frequency	Intensity	Frequency	Intensity	Frequency	Intensity
7.4388	0.2074	62.7065	0.0705	149.1798	21.2008
16.4909	0.2605	63.104	0.1781	158.1113	1.2944
17.5756	0.6885	72.9898	0.5922	167.3147	1.319
17.6354	0.2732	76.2044	0.0712	291.6073	45.2436
21.2389	1.9661	79.3629	1.3157	296.733	25.3429
21.5789	0.091	83.3437	0.7854	333.7049	9.2759
22.3766	0.7879	85.774	0.0222	485.4039	41.8643
24.69	0.0717	93.8017	1.8303	537.3363	53.9007
26.7358	0.9866	97.7618	1.8276	549.981	2.4552
27.0656	0.01	97.8442	1.0319	650.769	16.3354
29.5408	1.9493	109.4476	2.348	654.1337	18.2497
43.2924	0.007	116.5947	4.6642	654.2776	7.6688
48.3594	0.0222	123.3348	10.8883	655.3985	8.4776
55.3961	0.7678	140.1746	12.7988	656.7547	0.1858

657.4699	0.6328
657.6684	29.3765
658.3631	28.8576
658.7449	99.9877
660.5029	97.6479
660.7473	14.3552
661.3344	43.9292
775.7183	86.5684
835.0219	0.0823
858.7073	210.8972
1020.1355	49.0502
1200.398	660.7775
1367.9259	27.0912
1369.0351	33.4066
1371.7649	3.3488
1371.8683	37.7394
1373.6953	42.2252
1374.9194	9.7384
1835.8707	95.6867
1847.816	759.5944
2416.215	215.3154
2417.8355	89.7205
2421.3977	1182.7347
2425.1203	2334.8849
2428.1398	1905.5857
2452.0697	34.6526

Table S60. Cartesian coordinates for the optimized geometry of isomer 8a-sextet $U^+(CO_2)_8$ followed by its predicted frequencies (cm^{-1}) and IR intensities (km/mol).

Z	x	y	z
92	0.041304000	-0.065389000	-0.020219000
8	1.561371000	-1.795948000	-1.402613000
6	1.473641000	-2.929082000	-1.706323000
8	1.411769000	-4.030340000	-2.013035000
8	-1.786795000	0.150323000	1.851078000
6	-2.670825000	-0.330840000	2.461099000
8	-3.523658000	-0.766522000	3.086554000
8	-0.307146000	2.590920000	0.515284000
6	-0.266358000	3.712310000	0.856081000
8	-0.229289000	4.809402000	1.187523000
8	1.042483000	-0.988836000	1.906779000
6	2.184998000	-0.884138000	2.394699000
8	2.874195000	-1.230391000	3.276067000
8	2.324143000	0.713231000	0.242199000
6	3.182383000	0.432195000	1.093553000
8	4.285172000	0.615803000	1.443373000
8	0.754420000	1.104600000	-2.327456000
6	1.506171000	1.492812000	-3.140725000
8	2.230844000	1.875124000	-3.942038000
8	-2.184950000	0.865199000	-1.241523000
6	-3.311287000	0.744491000	-1.555163000
8	-4.406370000	0.666442000	-1.881528000
8	-1.080174000	-1.991105000	-0.485633000
6	-2.315424000	-2.358312000	-0.374311000
8	-3.278487000	-1.745505000	-0.004191000

Frequency	Intensity	Frequency	Intensity	Frequency	Intensity
13.9847	0.0505	66.5993	3.5141	145.8479	9.1882
17.4777	0.3132	72.9988	1.8676	161.2467	9.5892
20.2097	0.238	79.1718	1.0661	174.7412	3.1355
20.7482	0.6273	82.8299	0.6748	184.3833	6.7846
23.9842	0.7834	92.1932	0.8414	191.9292	5.5428
24.5627	0.121	92.3729	0.7581	192.7267	7.6966
26.8568	0.7666	100.7041	1.3071	214.6706	25.1416
29.6936	0.2745	102.7489	1.1735	314.9878	27.3241
29.8895	0.8655	105.5729	2.2167	318.8814	56.4686
35.7773	0.9739	108.3511	1.5168	359.0275	68.0502
54.036	0.0787	119.9048	9.7597	453.38	23.9177
56.4997	0.6262	123.3815	3.365	632.2776	36.048
59.7109	1.1468	133.325	5.7363	639.8427	33.2692
63.1139	3.4711	140.2134	2.4334	647.3465	13.5899

648.3959	108.658	677.6443	35.3892	1375.9107	23.0109
652.3287	22.3081	714.0809	7.0149	1756.5079	510.9211
652.6316	10.7352	1151.7978	135.4033	1985.1071	52.2894
653.9677	30.1629	1207.4281	357.5117	2047.7615	1362.5258
656.1587	19.6873	1286.5169	264.4044	2418.9275	369.6752
656.64	72.308	1365.2319	34.2763	2420.1479	560.8383
658.7035	42.7373	1368.5414	16.2221	2424.7007	1524.9121
660.0414	74.9257	1369.7614	69.4698	2427.5912	2031.7204
663.3398	187.0075	1374.2754	26.803	2450.4974	323.6422

Table S61. Cartesian coordinates for the optimized geometry of isomer 8b-quartet $U^+(CO_2)_8$ followed by its predicted frequencies (cm^{-1}) and IR intensities (km/mol).

Z	x	y	z
92	-0.236160000	0.004739000	0.230433000
8	1.623457000	-1.724919000	1.125543000
6	2.614733000	-1.770761000	1.750728000
8	3.578645000	-1.839160000	2.368835000
8	1.324315000	-0.737462000	-1.896953000
6	2.329545000	-0.834165000	-2.490630000
8	3.301066000	-0.939387000	-3.092780000
8	-1.823210000	2.022363000	1.144129000
6	-1.553003000	2.978420000	1.769630000
8	-1.311110000	3.918592000	2.380488000
8	-0.832923000	1.575092000	-1.954290000
6	-0.608893000	2.544535000	-2.572437000
8	-0.398157000	3.491430000	-3.186849000
8	3.863601000	0.313632000	-0.118990000
6	4.028523000	1.415020000	0.229452000
8	4.210641000	2.503199000	0.566799000
8	1.024819000	1.302754000	0.620261000
6	-1.260259000	-0.954633000	2.266216000
8	-1.756553000	-1.422937000	3.180062000
6	-1.060343000	-3.822636000	-0.872369000
8	-1.300070000	-4.932036000	-1.060238000
8	-0.817488000	-2.694634000	-0.684790000
6	-4.130283000	-0.201493000	-0.763709000
8	-5.269179000	-0.134405000	-0.909274000
8	-2.972031000	-0.272337000	-0.619588000

Frequency	Intensity	Frequency	Intensity	Frequency	Intensity
6.0012	0.0502	52.2686	0.1325	101.8689	0.2094
11.3631	0.1321	54.5891	0.3285	106.2064	0.8087
16.9547	0.0761	55.1504	0.3202	122.5239	3.836
17.9586	0.0715	59.1203	0.374	126.8939	16.0843
18.7241	0.3295	64.4202	0.607	145.2457	30.9846
20.6624	0.2593	69.9773	1.2103	149.2744	9.5905
21.2313	0.1067	71.4309	1.7769	156.3283	2.9791
21.5159	0.1223	75.6738	0.8629	199.9248	13.6312
25.2015	0.1514	76.0324	1.0728	236.2842	6.5887
29.5881	0.4713	80.1993	0.5459	273.1753	1.8935
30.797	0.0514	86.1464	0.4025	302.9387	0.4032
34.4756	0.2189	87.2945	1.3492	648.8101	24.1621
40.0397	0.893	91.9119	3.3099	652.4552	8.8322
42.9335	1.6275	95.7392	1.5414	654.9542	30.2266

655.3445	28.0019	664.7226	3.9027	1376.0387	18.3503
655.9639	35.6714	675.527	26.5495	2057.197	1007.8551
657.7306	33.6938	828.01	338.4867	2412.6151	163.7634
658.4052	36.4032	1366.9051	23.0369	2414.367	236.6764
658.687	28.8961	1370.5365	4.3086	2417.8714	257.2863
660.0744	26.7908	1371.8263	25.2754	2420.0083	1441.2662
660.6942	15.2739	1373.6069	12.9298	2424.1382	1970.9733
662.0556	20.2566	1374.4664	19.0609	2427.5583	2126.8625
662.7609	113.3404	1374.7809	22.0011	2451.7635	220.864

Table S62. Cartesian coordinates for the optimized geometry of isomer 8c-quartet $U^{+}(CO_2)_8$ followed by its predicted frequencies (cm^{-1}) and IR intensities (km/mol).

Z	x	y	z
92	0.325193000	-0.001912000	-0.032798000
8	-0.805989000	2.535268000	-0.364243000
6	-1.447196000	2.714874000	-1.331833000
8	-2.084284000	2.930662000	-2.261903000
8	2.342747000	1.574934000	-0.910832000
6	2.924562000	2.261740000	-1.659982000
8	3.502906000	2.937388000	-2.386642000
8	-0.833285000	-2.532128000	-0.329895000
6	-1.477423000	-2.717185000	-1.294484000
8	-2.117722000	-2.938182000	-2.221139000
8	2.324729000	-1.608237000	-0.898337000
6	2.889911000	-2.306347000	-1.649828000
8	3.452094000	-2.993074000	-2.378754000
8	-1.936143000	0.017966000	1.453971000
6	-3.031641000	0.026261000	1.865942000
8	-4.098698000	0.034504000	2.291600000
8	-0.486028000	-0.009621000	-1.731576000
6	-4.384834000	0.012980000	-1.165389000
8	-5.048380000	0.010882000	-2.071512000
6	0.998782000	2.729985000	2.918533000
8	1.205069000	3.551155000	3.698767000
8	0.793611000	1.892490000	2.129806000
6	0.979746000	-2.705499000	2.946974000
8	1.179861000	-3.519951000	3.735798000
8	0.780865000	-1.874672000	2.149624000

Frequency	Intensity	Frequency	Intensity	Frequency	Intensity
4.0907	0.0929	46.6461	2.8095	91.2381	1.7056
10.619	0.092	47.8279	0.1721	91.6336	0.1931
10.8917	0.0045	48.2062	0.2383	98.6172	0.2929
13.3747	0.1846	52.7171	0.276	111.2803	2.6674
14.7367	0.0076	56.3482	3.8123	116.7834	1.5195
17.288	0.0377	59.1554	0.0503	121.8215	1.9354
18.7134	0.5994	61.8305	0.0788	131.6283	39.8843
19.4158	0.2786	65.1792	0.6301	133.3904	9.0339
20.1821	0.3606	66.7477	0.0567	141.3772	20.1924
22.276	0.0002	67.1169	1.6276	177.2271	1.5687
23.1454	0.0195	70.8822	0.024	220.0116	14.8362
27.1235	0.1093	77.1774	3.0287	640.3608	22.297
31.2514	0.0619	84.5566	0.1568	641.5162	28.6202
35.1008	0.0143	86.5357	0.21	642.0613	65.6813

650.5692	19.3063	666.6982	9.6628	1373.7749	1.3455
651.3154	0.0368	667.6978	38.2289	2236.7853	67.7448
653.0573	9.6028	792.5163	305.5087	2405.5428	2.2109
653.1592	7.7981	1360.2999	2.1407	2408.8127	1086.6209
655.0853	35.9422	1360.6571	26.0109	2416.432	159.8019
655.387	74.1165	1370.6757	3.8177	2418.315	1247.9936
655.7721	102.6033	1370.7705	6.605	2423.243	1699.9882
660.3444	19.3573	1372.5827	10.1563	2427.6688	2363.6706
661.9716	31.7988	1373.1948	22.6187	2452.41	21.7894

Table S63. Cartesian coordinates for the optimized geometry of isomer 8d-doublet $U^+(CO_2)_8$ followed by its predicted frequencies (cm^{-1}) and IR intensities (km/mol).

Z	x	y	z
92	0.000000000	0.036056000	-0.000001000
8	-1.500233000	-2.192808000	-0.407570000
6	-2.558036000	-2.650303000	-0.634958000
8	-3.567943000	-3.147016000	-0.856565000
8	1.500264000	-2.192784000	0.407592000
6	2.558072000	-2.650262000	0.634987000
8	3.567982000	-3.146964000	0.856604000
8	2.099315000	0.705571000	0.300293000
6	3.372029000	0.515383000	0.521427000
8	4.276855000	1.268364000	0.641033000
8	0.608843000	-0.990775000	-2.393595000
6	1.010543000	-1.267877000	-3.460130000
8	1.399610000	-1.541633000	-4.502899000
8	0.178653000	1.995332000	-1.815474000
6	-0.088310000	3.005867000	-2.347245000
8	-0.339131000	3.993289000	-2.872936000
8	-2.099325000	0.705539000	-0.300301000
6	-3.372035000	0.515330000	-0.521435000
8	-4.276871000	1.268300000	-0.641052000
8	-0.178681000	1.995349000	1.815452000
6	0.088270000	3.005892000	2.347212000
8	0.339078000	3.993318000	2.872903000
8	-0.608830000	-0.990757000	2.393605000
6	-1.010527000	-1.267852000	3.460143000
8	-1.399589000	-1.541600000	4.502916000

Frequency	Intensity	Frequency	Intensity	Frequency	Intensity
5.1132	0.0436	42.2631	0.3046	117.0426	0.7467
12.9464	0.0019	50.7461	0.0107	119.1938	0.0529
13.4262	2.7698	55.9068	3.9628	128.1584	16.2173
14.6686	3.8599	64.5792	0.0683	136.2963	45.8376
16.5657	0.2577	73.8509	0.3524	136.3827	14.0796
18.8775	0.3868	80.5683	0.2054	142.282	0.6159
21.0548	6.5385	85.4046	2.4731	154.5706	0.1393
22.139	0.239	90.5701	0.2446	168.8323	0.2224
25.8074	0.1124	95.3944	0.1489	175.0141	0.8171
26.0809	0.3827	102.7364	0.1396	282.0861	8.6556
27.3768	0.465	103.2545	4.9406	290.5313	160.1147
27.4273	0.7894	104.0012	2.3499	623.308	2.7436
37.107	0.9026	104.5463	1.4607	626.5426	120.6592
37.3081	0.0624	110.5061	0.1669	648.4262	2.1579

648.8384	5.8983	725.834	67.1116	1378.703	7.1698
649.4272	0.9888	730.0133	37.9708	1843.4369	1125.4435
649.5989	12.8805	1126.3209	927.126	1850.5269	76.0014
652.1824	89.6333	1147.6753	0.895	2413.3643	1335.7436
653.4487	77.5756	1363.3616	54.4121	2415.7356	115.2104
654.5054	84.4368	1364.7528	4.6077	2424.2359	271.0321
655.5858	0	1376.5265	6.142	2429.742	1922.0969
655.9605	17.5696	1376.5507	41.2324	2432.6777	2319.6685
659.1689	1.535	1377.2687	66.8911	2455.563	22.6458

Table S64. Cartesian coordinates for the optimized geometry of isomer 8d-quartet $U^{+}(CO_2)_8$ followed by its predicted frequencies (cm^{-1}) and IR intensities (km/mol).

Z	x	y	z
92	0.000000000	0.000000000	0.213569000
8	-1.588247000	-0.096670000	2.442114000
6	-2.699866000	-0.129469000	2.815626000
8	-3.781125000	-0.161524000	3.197256000
8	1.588247000	0.096670000	2.442114000
6	2.699866000	0.129469000	2.815626000
8	3.781125000	0.161524000	3.197256000
8	2.126539000	0.008160000	-0.525056000
6	2.749124000	0.012544000	-1.663893000
8	2.316791000	0.001228000	-2.780246000
8	0.124974000	-2.468625000	1.200198000
6	0.213390000	-3.636002000	1.282181000
8	0.299239000	-4.775276000	1.369981000
8	0.000944000	-1.746236000	-1.743676000
6	0.000046000	-1.918215000	-2.908612000
8	0.000000000	-2.128036000	-4.032572000
8	-2.126539000	-0.008160000	-0.525056000
6	-2.749124000	-0.012544000	-1.663893000
8	-2.316791000	-0.001228000	-2.780246000
8	-0.000944000	1.746236000	-1.743676000
6	-0.000046000	1.918215000	-2.908612000
8	0.000000000	2.128036000	-4.032572000
8	-0.124974000	2.468625000	1.200198000
6	-0.213390000	3.636002000	1.282181000
8	-0.299239000	4.775276000	1.369981000

Frequency	Intensity	Frequency	Intensity	Frequency	Intensity
12.4567	0.0052	67.2703	0.8824	135.9404	0.0832
12.9931	0.7008	75.5507	0.8016	138.2608	0.1506
15.2584	0.0245	76.376	1.3745	139.4882	0.8331
19.0853	0.2599	78.9678	5.3722	147.923	0.9859
20.3477	1.2264	82.2503	0.0759	151.9579	11.5487
23.4292	0.4899	89.974	3.3426	157.0871	13.9616
26.0803	0.002	91.348	4.2495	158.202	9.387
26.6487	0.075	96.0321	0.1319	201.2307	10.774
34.3314	0.0028	99.071	0.8609	205.5129	0.0058
39.1072	0.4007	100.2661	0.0012	353.301	158.7484
51.813	0.0041	110.8958	13.4816	369.5772	27.1582
52.2414	5.0964	119.7858	2.4389	628.2872	0.0016
54.595	2.1198	121.9694	0.8412	633.0505	59.8736
59.8266	2.8958	133.2878	8.9166	644.7652	56.2746

649.7483	7.1692	708.818	12.3337	1375.5392	0.5155
651.3207	3.9646	711.7042	4.3616	1758.7696	74.4074
651.3968	55.3	1178.7735	275.481	1773.3231	804.2666
653.1938	35.4429	1199.3632	390.1298	2418.0456	72.1133
654.625	28.5342	1366.8425	5.0729	2419.8589	1502.8247
657.624	17.8939	1367.9271	76.4893	2419.9327	54.7918
658.9625	14.1903	1370.292	39.4186	2430.1696	1910.7062
659.3439	80.2778	1370.9801	4.765	2430.5081	2163.1194
659.4199	54.8236	1374.4546	59.1923	2454.8041	32.4466

Table S65. Cartesian coordinates for the optimized geometry of isomer 8d-sextet $U^+(CO_2)_8$ followed by its predicted frequencies (cm^{-1}) and IR intensities (km/mol).

Z	x	y	z
92	0.124001000	-0.012471000	-0.007830000
8	-1.994798000	1.008939000	1.302091000
6	-2.932064000	0.882266000	1.998141000
8	-3.840642000	0.805689000	2.694167000
8	-2.327292000	-0.476308000	-1.257170000
6	-3.215524000	-1.219276000	-1.445677000
8	-4.081621000	-1.938099000	-1.672252000
8	2.346745000	-0.624398000	1.428263000
6	2.932686000	-1.160169000	2.289778000
8	3.506184000	-1.670983000	3.144297000
8	1.600163000	-2.120061000	-1.037290000
6	1.487695000	-3.271650000	-1.226750000
8	1.381001000	-4.396508000	-1.433292000
8	1.050224000	2.452172000	0.891291000
6	1.283518000	3.431547000	1.487925000
8	1.511433000	4.387481000	2.085074000
8	-0.681653000	-1.905318000	0.876622000
6	-1.739933000	-2.413422000	1.424661000
8	-2.864807000	-2.006228000	1.459510000
6	-1.615471000	2.600207000	-2.215024000
8	-2.291979000	3.073492000	-3.014437000
8	-0.916370000	2.121949000	-1.407157000
6	3.290155000	0.832460000	-2.077223000
8	4.154892000	0.833845000	-2.834176000
8	2.404209000	0.836277000	-1.312376000

Frequency	Intensity	Frequency	Intensity	Frequency	Intensity
11.5785	0.1793	53.6402	1.231	108.1019	4.1869
13.08	0.3196	54.4678	1.333	110.3599	1.7606
15.4357	0.8449	58.3279	0.1228	118.9713	1.2621
16.6868	0.3198	66.3607	0.6425	122.1133	1.7762
18.5977	0.1556	67.1546	1.4372	125.7668	3.0166
19.1618	0.1798	72.5073	2.7576	132.6487	8.7929
21.5149	0.1086	75.1078	0.8977	140.0812	2.9577
22.1036	0.0417	80.1561	1.4081	141.9212	0.8069
23.0669	0.5111	86.1113	0.8552	151.4676	0.9277
27.0997	0.0449	86.3083	0.2827	351.8626	76.6331
32.4771	0.0997	91.5966	2.3258	621.3875	13.1577
35.3026	0.0202	93.8442	0.3316	628.7824	13.4573
42.3041	0.9195	102.6147	0.3807	631.0013	2.3278
50.9066	1.15	106.095	1.8144	633.0598	9.8378

633.8034	18.6409	655.7131	6.6807	1372.1085	8.0344
634.7678	70.8154	717.3677	7.5786	1772.7844	442.5542
637.424	39.5927	1197.0802	365.1042	2409.5266	240.6105
638.672	38.1986	1362.6369	9.5288	2411.7605	416.3759
649.0023	11.4874	1363.7922	8.3937	2415.4473	1536.2444
649.8375	12.4596	1364.1893	11.4758	2417.0595	796.6206
651.7393	57.0494	1366.1461	7.1289	2421.2578	2140.7818
652.5416	57.1982	1367.7257	6.4107	2426.4329	2301.2106
654.2482	52.2792	1371.2392	9.136	2449.3865	4.1596

Table S66. Cartesian coordinates for the optimized geometry of isomer 8e-sextet $U^+(CO_2)_8$ followed by its predicted frequencies (cm^{-1}) and IR intensities (km/mol).

Z	x	y	z
92	0.608942000	0.105112000	-0.086747000
8	2.007855000	-2.035421000	0.742473000
6	2.465754000	-2.996718000	1.228744000
8	2.917658000	-3.939458000	1.703573000
8	0.132622000	-2.332580000	-1.698583000
6	0.425017000	-2.523676000	-2.815702000
8	0.705766000	-2.723530000	-3.913108000
8	0.548225000	2.795823000	-0.138399000
6	0.524352000	3.959742000	-0.025242000
8	0.500628000	5.103102000	0.084001000
8	-1.727962000	0.918865000	-1.128888000
6	-2.684573000	1.226992000	-1.727328000
8	-3.617172000	1.537192000	-2.321620000
8	3.071706000	0.939423000	0.583854000
6	4.174847000	1.327561000	0.620758000
8	5.257445000	1.708896000	0.666130000
8	1.372778000	0.310598000	-1.952770000
6	0.156482000	0.455403000	2.136151000
8	-0.143541000	0.539078000	3.259104000
6	-2.387369000	-2.067402000	1.191602000
8	-3.221758000	-2.548372000	1.820659000
8	-1.534226000	-1.592374000	0.549573000
6	-5.560731000	0.208425000	0.832431000
8	-6.646880000	0.300736000	1.209334000
8	-4.461311000	0.116493000	0.451201000

Frequency	Intensity	Frequency	Intensity	Frequency	Intensity
3.7044	0.0067	38.1483	0.6854	106.8102	0.3942
6.777	0.0133	41.2977	0.2442	108.9485	0.1788
10.3253	0.0509	47.6908	0.3348	112.9487	0.87
14.6605	0.0185	55.8717	0.8168	120.7413	19.2018
17.3904	0.0698	56.6884	0.7212	125.2671	1.1706
17.7591	0.0203	58.8001	1.5686	133.2979	13.0922
20.3846	0.0393	69.4971	4.3698	137.8406	14.7298
23.249	0.0664	75.6195	1.6956	193.1235	5.7219
24.7818	0.0627	78.4953	1.576	284.938	14.1882
26.3574	0.0884	84.4379	0.7063	296.0337	1.4042
27.3668	0.1674	87.2084	0.1171	312.5573	9.6962
29.5239	0.2685	96.8924	2.3494	505.197	67.2906
32.3972	0.1488	98.8538	0.4806	642.9503	5.2779
37.0062	0.8361	104.7883	1.7793	643.7557	9.5204

644.6326	11.2231	666.7657	27.5922	1380.1779	12.1429
646.1677	72.957	668.7849	38.8695	1885.8747	603.8088
651.7515	2.78	671.1287	29.3045	2412.1985	1512.4289
653.1662	23.7282	1368.3623	15.7231	2417.2494	820.1213
655.2013	54.0091	1370.9308	6.1769	2420.5029	227.2189
656.6779	76.7915	1376.2455	8.3845	2423.6566	191.5486
658.477	10.3429	1376.9443	17.2286	2430.8916	2180.053
660.3177	2.4701	1377.8738	23.5767	2433.3018	2074.8486
663.9334	42.2638	1379.0643	16.8874	2457.4225	33.7582

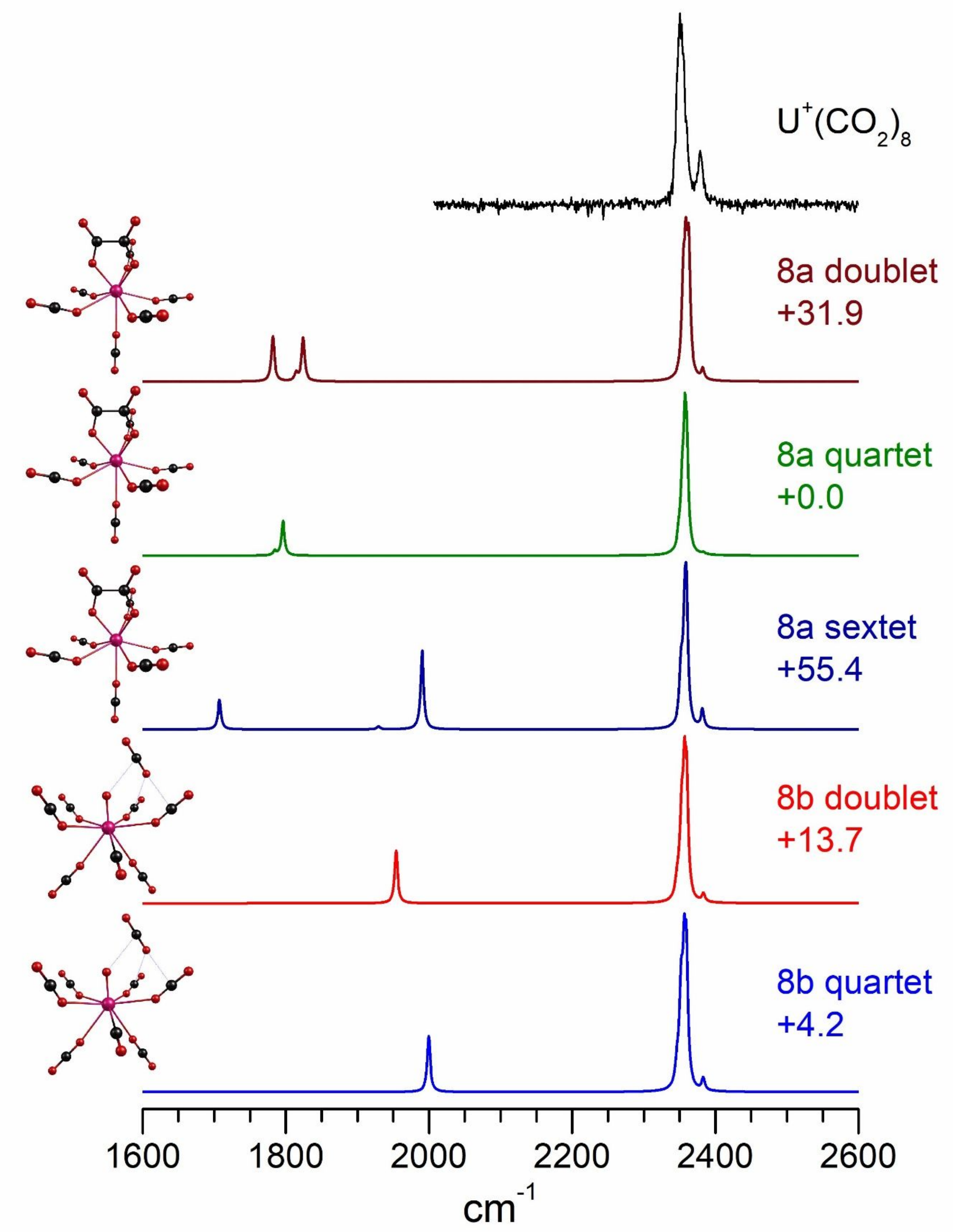

Figure S45. Experimental IR spectrum of $U^{+}(CO_2)_8$ compared with simulated spectra for isomers 8a and 8b. Relative energies (kcal/mol) are shown next to each spectrum.

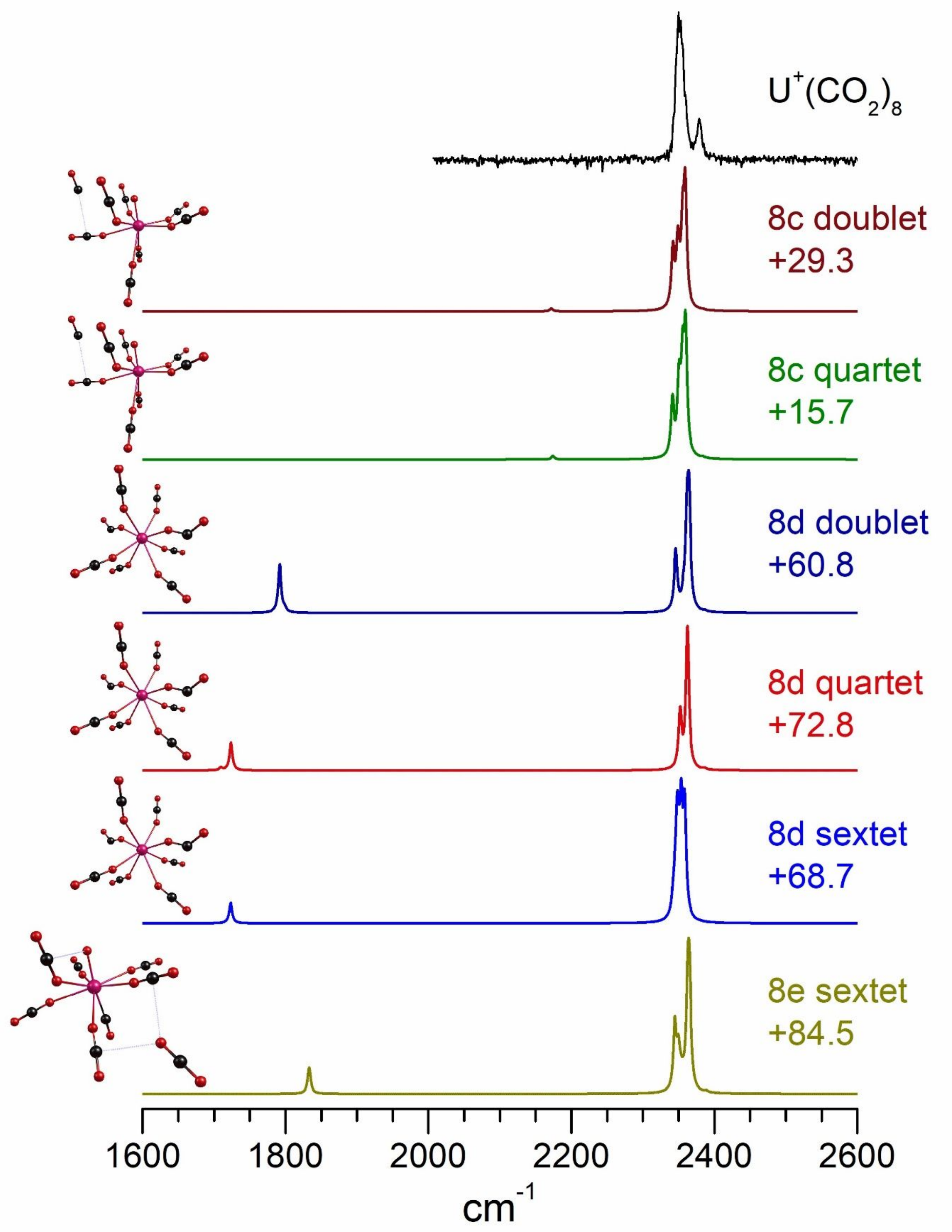

Figure S46. Experimental IR spectrum of $U^{+}(CO_2)_8$ compared with simulated spectra for isomers 8c–8e in their doublet, quartet, and sextet spin states. Relative energies (kcal/mol) are shown next to each spectrum.

Table S67. $U^+(CO_2)_9$ electronic energy calculated at the B3LYP/cc-pVTZ(-pp) level with Stuttgart/Koeln pseudopotential.

Isomer	2s + 1	Energy (hartree)	Rel. E (kcal/mol)	BDE (CO_2) (kcal/mol)	BDE (CO) (kcal/mol)	BDE (oxalate) (kcal/mol)
9a	2	-2172.476094	+15.5	20.1		76.9
9a	4	-2172.500873	+0.0	3.8		75.1
9a	6	-2172.380422	+75.6	-16.4		-0.4
9b	2	-2172.477155	+14.9	2.7		
9c	2	-2172.451044	+31.3	1.9	-43.3	
9c	4	-2172.473904	+16.9	2.5	3.3	
9d	2	-2172.405666	+59.7	4.8		
9d	4	-2172.40659	+59.2	17.4		
9d	6	-2172.40706	+58.9	13.6		
9e	6	-2172.383731	+73.5	-14.3		
9f	6	-2172.368758	+82.9	5.4		

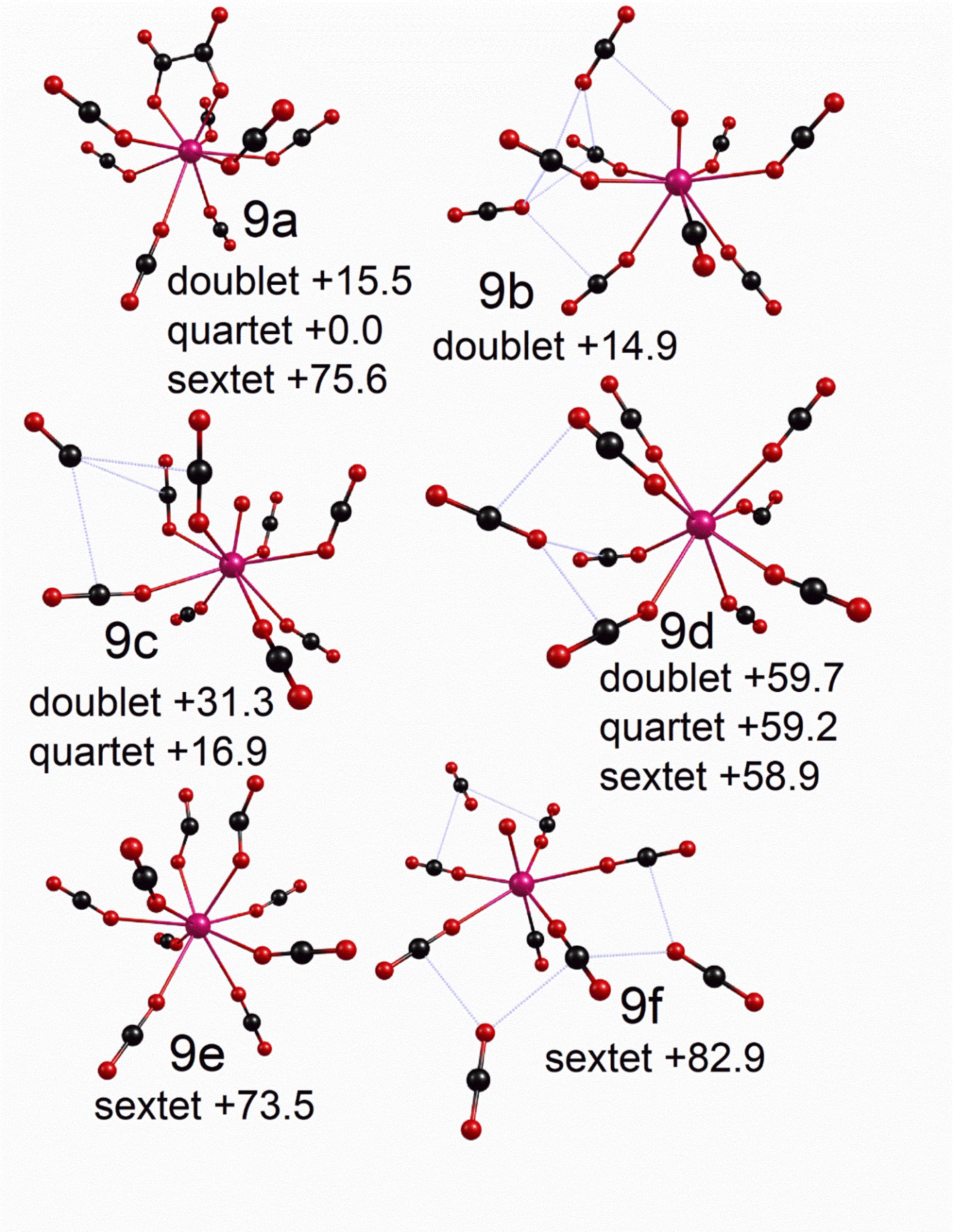

Figure S47. Predicted minimum energy structures of $U^+(CO_2)_9$ with energy of each spin state in kcal/mol. The lowest energy spin state of each isomer is shown.

Table S68. Cartesian coordinates for the optimized geometry of isomer 9a-quartet $U^{+}(CO_2)_9$ followed by its predicted frequencies (cm^{-1}) and IR intensities (km/mol).

Z	x	y	z
92	0.000351000	0.172625000	-0.056459000
8	-0.002915000	-1.386852000	1.494162000
6	-0.009624000	-2.705816000	1.215786000
8	-0.011902000	-3.582293000	2.022734000
8	-1.648686000	0.302552000	-2.273635000
6	-2.074126000	-0.455946000	-3.062151000
8	-2.499766000	-1.180553000	-3.842567000
8	-1.575092000	2.501460000	-0.114623000
6	-2.391850000	3.335084000	-0.201500000
8	-3.189399000	4.157600000	-0.286072000
8	-2.644264000	-0.236970000	0.598167000
6	-3.389244000	-0.914242000	1.201758000
8	-4.130874000	-1.563564000	1.787368000
8	0.014649000	1.313435000	2.479700000
6	0.014690000	0.863834000	3.564651000
8	0.014752000	0.447325000	4.633123000
8	2.645457000	-0.263903000	0.581169000
6	3.386750000	-0.951757000	1.177347000
8	4.124497000	-1.611335000	1.756336000
8	1.633312000	0.289639000	-2.285436000
6	2.045372000	-0.471302000	-3.078722000
8	2.458185000	-1.198237000	-3.863822000
8	-0.011426000	-1.802602000	-1.020170000
6	-0.014332000	-2.962531000	-0.333159000
8	-0.019669000	-4.052084000	-0.813623000
8	3.241010000	4.114764000	-0.288192000
6	2.429858000	3.305352000	-0.206782000
8	1.599979000	2.484419000	-0.123263000

Frequency	Intensity	Frequency	Intensity	Frequency	Intensity
7.0075	0.0129	28.3191	0.2849	82.6799	2.2238
13.0727	1.2124	31.2641	1.1848	86.1845	0.2725
16.2603	1.7419	31.3419	3.6868	89. 646	0.011
18.8485	0.3006	38.1916	0.0809	94.5782	3.286
18.9341	0.0357	41.0015	0.0446	99.7011	1.6022
20.1108	0.9748	53.4142	0.4131	101.206	0.872
20.7864	0.0306	55.3088	0.1107	101.7196	0.1499
22.945	0.0104	57.9367	0.0335	105.4676	12.5139
24.1584	0.0484	72.405	0.3402	115.7801	0.4474
26.1751	0.0007	73.0643	0.0045	135.2945	31.7692
27.7767	2.632	82.5306	0.4307	135.741	0.7786

148.6493	4.3927	658.0796	29.6439	1367.248	4.7573
152.3674	10.5	658.8487	58.5966	1367.9152	36.7779
155.3001	0.1411	659.4267	25.6852	1370.1794	29.2971
180.895	1.3809	660.0611	83.6808	1370.8668	29.1932
284.5896	33.8245	660.6366	19.8157	1375.6422	20.3924
287.8246	39.7546	661.2306	90.9976	1376.605	15.6847
333.4163	9.0198	663.4077	54.1527	1826.9389	87.6705
484.7972	44.1268	664.4121	13.4906	1840.0331	661.1047
527.8538	41.0644	664.694	49.1944	2411.3438	4.0682
553.4014	4.7777	778.4664	80.8632	2413.2836	37.2631
653.7171	1.0936	838.5346	0.0582	2418.3624	0.039
654.8134	2.4804	862.21	160.2019	2421.3215	2107.7328
655.209	10.3455	1040.6063	52.2311	2424.4884	1938.5637
656.2	7.122	1215.6656	604.9113	2427.7197	2107.4517
657.226	7.6921	1365.5961	23.4504	2453.513	5.1154

Table S69. Cartesian coordinates for the optimized geometry of isomer 9a-sextet $U^+(CO_2)_9$ followed by its predicted frequencies (cm^{-1}) and IR intensities (km/mol).

Z	x	y	z
92	0.222934000	-0.001615000	0.031665000
8	-1.692559000	0.012254000	1.041831000
6	-2.927169000	0.020688000	0.409913000
8	-3.988177000	0.026529000	0.988568000
8	-0.297811000	2.637727000	-0.522018000
6	-1.119613000	3.333052000	-0.990213000
8	-1.907993000	4.029459000	-1.447168000
8	2.535755000	1.591719000	-0.035981000
6	3.356479000	2.424406000	0.015129000
8	4.165631000	3.238669000	0.064065000
8	0.476938000	1.589632000	2.275138000
6	-0.279997000	2.013500000	3.066263000
8	-1.001258000	2.439489000	3.849569000
8	0.456568000	-1.603450000	2.269464000
6	-0.302470000	-2.021058000	3.061853000
8	-1.026080000	-2.441086000	3.846231000
8	-0.342348000	-2.630652000	-0.527367000
6	-1.175847000	-3.311001000	-0.996910000
8	-1.975836000	-3.993129000	-1.455219000
8	1.178515000	-0.001923000	-2.565249000
6	0.658150000	0.004099000	-3.617550000
8	0.171452000	0.009868000	-4.656308000
8	-1.438019000	0.013547000	-1.487071000
6	-2.686542000	0.020858000	-1.021135000
8	-3.635167000	0.028432000	-1.934702000
8	4.116260000	-3.302313000	0.037705000
6	3.318451000	-2.476540000	-0.004161000
8	2.509306000	-1.632200000	-0.048031000

Frequency	Intensity	Frequency	Intensity	Frequency	Intensity
3.7639	0.0003	28.5084	2.3373	86.6881	0.1086
13.8084	0.9128	30.4084	1.3829	88.5676	0.0486
17.6792	1.1991	32.9277	1.6123	88.8116	0.5543
18.4359	0.0547	41.79	0.9146	93.866	3.0154
18.6202	0.1862	49.4695	0.1845	100.8013	0.8198
18.8919	1.5089	55.7186	0.1134	102.9507	2.6227
21.539	0.0466	61.2268	0.1212	107.9508	9.2995
23.7684	0.5592	70.2268	0.0397	115.44	0.1673
24.3882	0.0782	75.9312	0.1854	128.1574	8.1391
26.4891	0.2881	81.7644	0.0495	134.287	27.7332
27.7406	0.694	82.0437	1.7289	138.3614	0.6637

151.5076	4.954	655.7086	0.0034	1365.8562	21.7216
156.3639	9.0446	656.1164	5.8247	1366.3904	3.395
162.31	0.0052	657.0842	0.5857	1367.0167	29.9414
184.3743	1.2074	658.5722	35.0834	1368.0503	24.2611
244.3211	15.5861	658.6931	96.6628	1369.009	33.205
277.8952	4.8414	659.6051	49.2446	1374.7829	17.7434
289.5716	36.8219	660.5573	102.9745	1375.6265	14.3093
295.0182	5.9476	660.97	57.0317	1707.459	431.1992
493.554	36.4395	663.0825	87.1638	2409.6149	1.8524
565.6295	23.4708	663.7915	3.5317	2412.8254	16.2942
591.4902	9.3933	665.383	19.4987	2417.4725	6.3569
644.7461	17.8693	846.4527	158.8027	2419.7255	1953.1683
653.7028	3.0304	990.4411	195.6408	2424.0599	2029.0185
654.2615	1.9771	1181.2617	48.4048	2426.0098	2038.6065
655.3283	5.5777	1284.6498	168.3536	2450.9038	1.9633

Table S70. Cartesian coordinates for the optimized geometry of isomer 9b-doublet $U^{+}(CO_2)_9$ followed by its predicted frequencies (cm^{-1}) and IR intensities (km/mol).

Z	x	y	z
92	-0.711798000	0.032395000	0.230339000
8	-1.852880000	-0.500701000	-2.214452000
6	-2.218980000	-1.355541000	-2.927071000
8	-2.581124000	-2.183468000	-3.635419000
8	1.263286000	-0.362776000	-1.630371000
6	2.208277000	-0.897929000	-2.067564000
8	3.131599000	-1.409137000	-2.520029000
8	2.125178000	-2.922795000	0.243600000
6	1.452420000	-3.859558000	0.421979000
8	0.804717000	-4.798383000	0.594654000
8	1.619280000	0.000360000	1.513129000
6	2.516950000	-0.514682000	2.060883000
8	3.402694000	-0.997114000	2.608306000
8	-3.358263000	-0.323026000	0.697093000
6	-3.975413000	-1.171448000	1.223194000
8	-4.596838000	-1.989712000	1.733006000
8	-0.754717000	-1.800441000	0.421021000
6	-1.128354000	1.241326000	2.319519000
8	-1.316193000	1.826790000	3.287812000
6	1.736161000	3.173102000	-0.280163000
8	2.607310000	3.925085000	-0.269887000
8	0.847502000	2.414377000	-0.293837000
6	5.322837000	1.011064000	-0.400420000
8	6.461318000	1.157857000	-0.504765000
8	4.168578000	0.863447000	-0.295224000
6	-2.994574000	3.098651000	-0.962042000
8	-3.711529000	3.974937000	-1.163356000
8	-2.263729000	2.208421000	-0.761421000

Frequency	Intensity	Frequency	Intensity	Frequency	Intensity
6.9518	0.0557	32.6919	0.0704	62.6012	0.8881
12.3975	0.0813	35.5649	1.6101	66.6023	0.1328
13.4486	0.1443	36.08	0.1241	73.5067	1.5344
15.9304	0.0388	38.7959	0.0201	76.8805	0.3787
18.0189	0.1917	41.5637	0.3906	77.9362	1.1958
20.29	0.1907	47.7752	0.7363	79.3093	1.7313
20.8434	0.0364	52.2574	0.2484	85.9429	0.2318
22.74	0.0996	54.4873	0.2421	90.6712	0.5739
23.3915	0.4187	55.7656	0.1323	93.2828	1.7274
28.6061	0.513	57.3868	0.6481	94.712	2.8664
29.2119	0.2999	59.3677	0.3262	97.0546	1.7654

109.2924	0.9074	654.44	19.1086	1370.7279	4.1518
114.8115	0.1543	655.8403	34.3097	1372.9087	22.2908
123.35	6.0414	656.6214	91.9007	1373.6194	9.6494
130.1954	15.766	657.777	19.4973	1375.8228	23.9155
145.2073	19.0203	658.8884	34.2471	1376.3381	11.8339
150.0494	24.4937	660.0972	26.915	1378.5329	22.478
158.2256	4.093	661.0604	16.0627	2010.7759	1087.343
194.0489	14.9238	662.2154	82.4945	2411.6487	750.6686
256.2349	0.6111	663.6465	13.6296	2414.8271	124.5971
275.1041	4.0592	669.5623	30.8777	2416.1714	962.2531
307.0345	1.5338	671.2563	34.747	2417.5256	315.7496
647.9611	20.7194	675.1813	30.5774	2422.9509	1076.9156
650.4535	4.3723	842.6152	326.3973	2423.7198	1663.7539
652.6185	20.3766	1368.5647	25.5695	2430.8733	2002.5073
653.7816	23.9376	1369.3335	6.8631	2454.2202	346.6067

Table S71. Cartesian coordinates for the optimized geometry of isomer 9c-doublet $U^{+}(CO_2)_9$ followed by its predicted frequencies (cm^{-1}) and IR intensities (km/mol).

Z	x	y	z
92	-0.350555000	-0.187649000	-0.006513000
8	-0.754226000	-2.090829000	-1.879333000
6	-0.562189000	-2.836934000	-2.760899000
8	-0.382093000	-3.572163000	-3.625368000
8	-0.297994000	1.105497000	-2.470318000
6	0.741111000	1.434615000	-2.905710000
8	1.740204000	1.774369000	-3.358470000
8	0.832887000	0.366433000	2.446899000
6	1.981126000	0.602593000	2.497484000
8	3.102108000	0.838153000	2.579378000
8	2.263880000	3.086677000	0.006997000
6	3.297117000	2.677413000	-0.346229000
8	4.325420000	2.285545000	-0.694302000
8	-0.034687000	-2.560225000	1.198154000
6	0.485778000	-3.507053000	1.650968000
8	0.967536000	-4.443205000	2.109121000
8	1.476686000	0.000636000	-0.414423000
6	3.218939000	-2.804599000	0.069123000
8	4.214483000	-2.607442000	-0.412590000
6	-2.805145000	-0.063336000	3.225285000
8	-3.433992000	-0.058885000	4.189894000
8	-2.171993000	-0.070794000	2.243161000
6	-0.632677000	3.571471000	0.644715000
8	-0.471045000	4.698900000	0.795053000
8	-0.820503000	2.425035000	0.496108000
8	-2.941802000	0.354544000	-1.156298000
8	-4.922980000	0.861406000	-2.252050000
6	-3.938075000	0.611576000	-1.710352000

Frequency	Intensity	Frequency	Intensity	Frequency	Intensity
8.4678	0.0117	25.7785	0.0324	59.4317	0.1723
8.9955	0.0179	31.0264	0.0254	61.9465	0.1312
10.1854	0.051	32.241	0.0218	65.3124	0.2759
12.9417	0.0817	34.9631	0.115	67.8751	0.5744
14.2662	0.1922	35.5452	0.1821	70.5887	0.2827
16.6167	0.095	39.72	0.3804	73.7968	0.6934
16.9894	0.0183	47.9223	2.0146	80.5385	2.4883
19.3462	0.0668	49.7316	2.6215	83.1741	2.2368
20.319	0.2789	51.9127	0.3183	87.2933	1.0701
22.5356	0.057	54.7544	1.3751	89.7267	0.3362
23.3539	0.32	57.4085	1.8464	91.2861	0.6381

93.1253	0.328	647.1968	40.2238	1368.0853	4.8848
96.302	0.3856	649.3679	4.5828	1369.0061	1.8779
100.0706	0.248	650.1578	4.6762	1369.5154	6.7341
118.2496	2.0863	651.7881	42.5123	1369.7764	2.949
121.7495	0.7795	652.9014	75.7532	1371.0769	5.9681
128.284	8.2892	654.4148	64.2907	1372.2797	3.2256
132.8785	11.109	656.9833	40.7934	2234.2807	58.8471
136.3506	23.5207	658.5967	81.2789	2405.6928	39.9055
154.0884	17.4638	660.0226	31.0232	2407.3829	267.8241
174.8066	4.7107	663.2894	21.7476	2415.6431	211.035
210.5623	12.0966	664.7941	26.2847	2417.5085	2415.2879
638.3439	22.3066	672.4089	18.2379	2419.2555	65.3434
641.193	27.8429	790.1811	323.6223	2422.4956	1671.3067
644.7636	12.2667	1361.4836	10.0187	2425.7055	2689.3115
646.5716	32.171	1362.7956	14.3058	2452.4324	26.5619

Table S72. Cartesian coordinates for the optimized geometry of isomer 9d-quartet $U^+(CO_2)_9$ followed by its predicted frequencies (cm^{-1}) and IR intensities (km/mol).

Z	x	y	z
92	-0.426978000	0.006262000	-0.049492000
8	-1.536918000	-2.248415000	0.995212000
6	-2.277558000	-2.712735000	1.779695000
8	-2.986438000	-3.215109000	2.528659000
8	0.872321000	-2.216254000	-0.969634000
6	1.788622000	-2.599943000	-1.591898000
8	2.678490000	-2.993034000	-2.200554000
8	0.957797000	0.763475000	-1.655478000
6	1.977299000	1.483546000	-1.993525000
8	2.700793000	2.170263000	-1.333056000
8	-1.973190000	-1.100730000	-1.920969000
6	-2.579758000	-1.371546000	-2.887241000
8	-3.175054000	-1.641221000	-3.829101000
8	-1.887505000	1.914702000	-1.289619000
6	-2.606554000	2.836717000	-1.192554000
8	-3.307676000	3.740487000	-1.113864000
8	-1.970737000	0.676363000	1.397261000
6	-2.816347000	0.453444000	2.369027000
8	-3.515163000	1.178250000	2.989982000
8	0.862497000	2.004572000	1.074995000
6	1.567258000	2.944520000	1.123932000
8	2.229510000	3.872275000	1.229318000
8	1.146955000	-0.952187000	1.869974000
6	2.064046000	-1.124278000	2.580145000
8	2.950960000	-1.297348000	3.285204000
6	4.740927000	-0.235108000	-0.232442000
8	5.766186000	0.183761000	-0.547471000
8	3.703970000	-0.667823000	0.091946000

Frequency	Intensity	Frequency	Intensity	Frequency	Intensity
8.5912	0.0185	30.9667	0.4355	79.6622	0.9425
14.0541	0.1167	32.0862	0.6597	84.4972	0.0522
16.2144	2.2273	33.7528	2.2682	87.2602	4.7623
17.0204	1.0063	41.1445	0.2572	93.7206	0.6478
19.22	1.2662	43.1631	0.9633	96.5462	1.7736
20.9437	0.6917	45.865	0.1577	99.25	5.3673
22.318	0.8446	60.0644	0.2852	102.4763	0.5293
24.2951	0.2144	60.7267	0.3227	105.9744	0.6507
25.0483	2.2659	64.7378	1.0578	111.084	0.8148
25.8628	0.2571	70.168	1.2355	112.9508	2.3154
29.5488	2.192	78.5747	0.4399	117.9452	5.0205

119.3771	3.3217	651.2581	14.1307	1369.2282	6.5604
127.6587	1.3992	652.512	57.0069	1370.8794	32.1579
134.5773	21.8931	653.4241	42.6519	1372.857	33.614
138.9231	9.0934	654.5588	52.4734	1373.3375	18.8895
146.2677	21.793	656.6607	45.486	1377.2751	57.2847
149.0025	3.8988	657.1889	23.069	1378.2754	3.2491
159.7119	0.445	659.1789	34.9955	1777.1475	525.3128
160.769	5.4719	659.5709	4.4842	1847.39	566.0409
170.128	0.6566	663.5281	58.566	2413.5804	1133.3586
288.2117	82.5271	672.0805	26.4938	2416.2227	351.2896
343.6512	113.5396	718.0913	5.6167	2419.9235	451.4907
617.9219	41.4799	728.0544	51.6063	2421.9917	495.1024
625.8157	65.6247	1132.1795	553.3945	2427.3839	1689.9523
647.0787	23.9071	1196.1812	280.9129	2432.9721	2299.2461
649.8016	4.1805	1364.4934	34.5102	2455.2432	76.1988

Table S73. Cartesian coordinates for the optimized geometry of isomer 9d-quartet $U^{+}(CO_2)_9$ followed by its predicted frequencies (cm^{-1}) and IR intensities (km/mol).

Z	x	y	z
92	-0.427456000	0.002340000	-0.038178000
8	-1.559895000	-2.260060000	0.975740000
6	-2.307407000	-2.733428000	1.747721000
8	-3.024977000	-3.238621000	2.486427000
8	0.874740000	-2.202611000	-0.997996000
6	1.787574000	-2.584177000	-1.626507000
8	2.674034000	-2.974920000	-2.241667000
8	0.925479000	0.799869000	-1.651138000
6	1.946581000	1.517530000	-1.988749000
8	2.692424000	2.175468000	-1.323645000
8	-1.976318000	-1.098837000	-1.918149000
6	-2.577828000	-1.332527000	-2.897149000
8	-3.168431000	-1.566558000	-3.851510000
8	-1.918523000	1.897913000	-1.281558000
6	-2.605519000	2.845455000	-1.204197000
8	-3.276341000	3.773569000	-1.143602000
8	-1.949105000	0.686195000	1.422347000
6	-2.806735000	0.487518000	2.385324000
8	-3.451157000	1.228767000	3.043984000
8	0.902974000	1.957733000	1.118365000
6	1.604988000	2.899258000	1.173158000
8	2.266427000	3.827057000	1.284261000
8	1.146380000	-1.003940000	1.859498000
6	2.069307000	-1.179588000	2.561136000
8	2.962033000	-1.356872000	3.257874000
6	4.717844000	-0.216895000	-0.262880000
8	5.737242000	0.214780000	-0.579417000
8	3.687155000	-0.663203000	0.063340000

Frequency	Intensity	Frequency	Intensity	Frequency	Intensity
9.089	0.0179	30.6151	0.9308	79.9401	0.289
14.7145	0.1848	32.0474	0.1448	85.1317	0.1434
16.0865	1.7127	34.6669	2.923	87.7814	5.2937
16.9838	1.4357	40.7054	0.2753	91.7765	0.481
18.6978	1.6757	43.3436	1.0824	96.4684	2.8214
20.5297	1.0817	45.4302	0.0921	98.0416	5.0173
21.9273	0.3963	60.0516	0.1294	102.6565	0.4726
23.9973	0.9539	60.5564	0.2051	105.7623	1.1078
24.6319	0.9318	64.4044	0.7075	111.1217	0.6207
25.845	0.2268	70.5131	2.1089	114.3999	1.928
29.9546	1.3073	79.2585	1.2003	118.2905	6.8963

123.962	1.5278	651.54	10.1633	1369.1305	6.487
127.4512	0.694	652.7544	61.2064	1370.9179	31.5487
135.0476	19.6195	653.3541	45.7908	1373.0485	24.2844
138.5821	10.3972	654.8426	54.3241	1373.8733	26.1885
147.4646	15.915	656.605	38.3107	1377.0221	57.0812
149.6957	6.7089	657.0799	22.611	1378.0557	3.2156
159.0017	2.7724	659.1244	35.2265	1776.2057	528.2227
169.4319	0.9896	659.6802	5.9167	1850.8614	628.0412
175.5984	4.0259	663.2307	60.9172	2413.829	855.0433
288.5578	78.4371	672.185	26.3159	2417.1612	570.9377
343.6284	111.7901	717.7827	5.544	2419.8142	486.0531
618.4048	41.9873	722.139	60.6997	2422.1744	471.7814
630.1907	56.0475	1136.3123	519.6707	2427.4622	1719.7572
647.3318	23.6808	1197.0639	276.8462	2432.8628	2291.2129
650.1189	1.8675	1366.1288	35.8469	2455.1138	82.2401

Table S74. Cartesian coordinates for the optimized geometry of isomer 9e-sextet $U^+(CO_2)_9$ followed by its predicted frequencies (cm^{-1}) and IR intensities (km/mol).

Z	x	y	z
92	-0.082107000	-0.097290000	-0.021323000
8	2.296100000	-1.101805000	0.990069000
6	3.322727000	-1.228123000	1.538697000
8	4.328610000	-1.360875000	2.080733000
8	1.133418000	-1.672641000	-1.907352000
6	1.862127000	-2.029594000	-2.751396000
8	2.569976000	-2.386210000	-3.586328000
8	-1.994006000	1.898038000	0.153473000
6	-2.514118000	2.897886000	0.471457000
8	-3.027900000	3.879966000	0.778417000
8	-0.852726000	0.804804000	-2.529137000
6	-0.701983000	1.439884000	-3.501156000
8	-0.565983000	2.056491000	-4.462666000
8	0.783878000	1.572159000	1.569304000
6	1.644710000	2.453005000	1.451112000
8	2.214635000	3.320275000	2.002721000
8	1.640855000	1.349599000	-1.027781000
6	2.308284000	2.281527000	-0.563462000
8	3.135145000	3.083674000	-0.795264000
6	-1.532919000	-0.077354000	3.548148000
8	-1.689337000	0.107365000	4.673289000
8	-1.379294000	-0.279998000	2.405185000
6	-3.643240000	-1.362972000	-1.031561000
8	-4.711720000	-1.632449000	-1.369928000
8	-2.556389000	-1.094501000	-0.691323000
8	-0.233575000	-2.758960000	0.707263000
8	-0.451015000	-5.022102000	1.176477000
6	-0.340837000	-3.899588000	0.942247000

Frequency	Intensity	Frequency	Intensity	Frequency	Intensity
8.6149	0.0681	24.8014	0.0459	75.8355	0.6362
12.551	0.2948	26.0675	0.0686	76.6142	0.0095
15.0758	0.3175	26.3456	0.087	79.7723	0.3632
16.341	0.4887	28.4616	0.1614	83.7289	0.7289
18.404	0.0828	37.149	0.0459	86.7271	0.0698
20.3297	0.5867	38.6155	0.1011	89.6033	0.1918
21.4561	0.0159	46.1825	0.1031	90.0444	0.0973
22.2221	0.2792	58.455	0.1356	94.2771	0.2585
23.1723	0.4544	68.5586	0.1263	96.1125	0.5375
24.0118	0.0894	73.6937	0.0244	96.7878	1.093

99.0389	0.1408	629.2275	78.9233	1342.5638	24.7646
107.3782	0.1845	634.5268	0.8884	1348.9019	11.3153
108.4672	1.3328	634.6541	9.1335	1353.0732	7.3094
110.1965	0.8067	635.9781	25.0735	1354.1086	7.8184
118.4739	0.3642	637.0772	44.3894	1358.3632	0.7442
122.0394	0.224	637.6149	35.8714	1358.8938	4.6455
131.6222	0.209	640.3595	26.6077	1360.7053	1.9881
176.2607	1.301	641.4627	8.2202	1982.3624	37.485
179.9036	16.8649	642.251	40.625	2038.9736	1547.567
197.7191	6.1036	644.595	39.6581	2407.4165	174.6102
290.787	33.9453	646.8671	19.5131	2409.4634	261.3122
308.2365	0.337	648.9355	88.2586	2410.8644	369.4849
448.983	16.5637	654.3411	142.7514	2412.2837	1818.5243
605.5825	35.2912	681.573	11.2444	2416.2298	2737.3903
614.7902	28.4272	1166.8803	118.5849	2419.528	2891.9231
628.3715	24.5092	1295.4772	201.5441	2448.0074	34.7032

Table S75. Cartesian coordinates for the optimized geometry of isomer 9f-sextet $U^+(CO_2)_9$ followed by its predicted frequencies (cm^{-1}) and IR intensities (km/mol).

Z	x	y	z
92	0.317039000	-0.000048000	-0.194608000
8	2.392002000	-1.573180000	0.386419000
6	3.329294000	-2.247753000	0.571695000
8	4.239725000	-2.922629000	0.758405000
8	-0.464385000	-2.542072000	-0.423538000
6	-0.880481000	-3.624576000	-0.575328000
8	-1.274555000	-4.692986000	-0.724665000
8	-0.464194000	2.542024000	-0.423628000
6	-0.880197000	3.624545000	-0.575529000
8	-1.274163000	4.692979000	-0.724984000
8	-2.257245000	-0.000023000	-0.859663000
6	-3.415485000	0.000176000	-1.018197000
8	-4.551862000	0.000372000	-1.185701000
8	2.391775000	1.573456000	0.386390000
6	3.328875000	2.248176000	0.572113000
8	4.239099000	2.923206000	0.759261000
8	0.841668000	-0.000048000	-2.118964000
6	-0.282529000	-0.000023000	2.006121000
8	-0.599183000	0.000001000	3.129222000
6	-4.634936000	-3.393087000	0.558709000
8	-5.556177000	-3.984845000	0.920832000
8	-3.702387000	-2.793217000	0.193494000
6	-4.634459000	3.393046000	0.558580000
8	-5.555989000	3.984415000	0.920599000
8	-3.701627000	2.793560000	0.193451000
6	6.443060000	-0.000306000	-0.190212000
8	7.584318000	-0.001198000	-0.355476000
8	5.287373000	0.000588000	-0.024429000

Frequency	Intensity	Frequency	Intensity	Frequency	Intensity
3.0555	0.0434	32.3423	0.0443	65.6782	0.1256
3.3809	0.0033	34.5057	0.0524	66.2557	2.7384
6.054	0.0244	35.9771	0.4467	73.992	0.1134
7.3717	0.0114	36.9984	0.2778	74.4334	0.0526
8.0486	0.0007	41.1304	0.6057	78.8888	0.012
16.2152	0.0206	41.9399	0.0103	79.1018	0.1343
18.814	0.0685	42.2664	0.3643	87.3051	0.21
22.2688	0.0427	46.265	0.1596	90.9869	0.0037
23.8878	0.1846	54.9795	0.2026	96.9747	0.5269
28.2311	0.1747	60.3342	0.7381	97.3759	1.0568
29.9613	0.0237	63.9726	2.5049	108.3768	0.24

110.1895	0.0526	647.5908	38.5168	1370.8175	6.6735
117.483	0.7176	647.9838	33.0209	1379.5249	0.057
118.775	1.1696	649.0127	86.4167	1379.7755	17.5754
119.7136	0.0755	649.9392	51.9627	1380.1667	38.9646
121.4228	1.5576	652.4942	83.6195	1380.7928	22.7023
129.2445	0.3528	655.3589	2.8617	1382.2381	6.7815
139.8616	29.0326	655.9326	0.119	1872.46	520.3061
142.4461	34.1831	667.8423	5.146	2414.3929	731.5012
276.4027	16.2611	668.4045	2.5957	2416.1301	2594.4777
276.7098	18.8097	668.6368	98.3969	2419.8173	474.7466
305.7843	0.4059	671.0326	23.1236	2424.2596	44.0369
537.2255	52.6881	671.4447	43.2114	2424.3073	4.8481
643.6198	5.9414	672.053	18.823	2434.4946	2182.9581
644.7462	10.3075	1370.7267	9.7295	2436.1063	1774.5699
644.9617	18.5174	1370.7567	3.7489	2460.1008	10.052

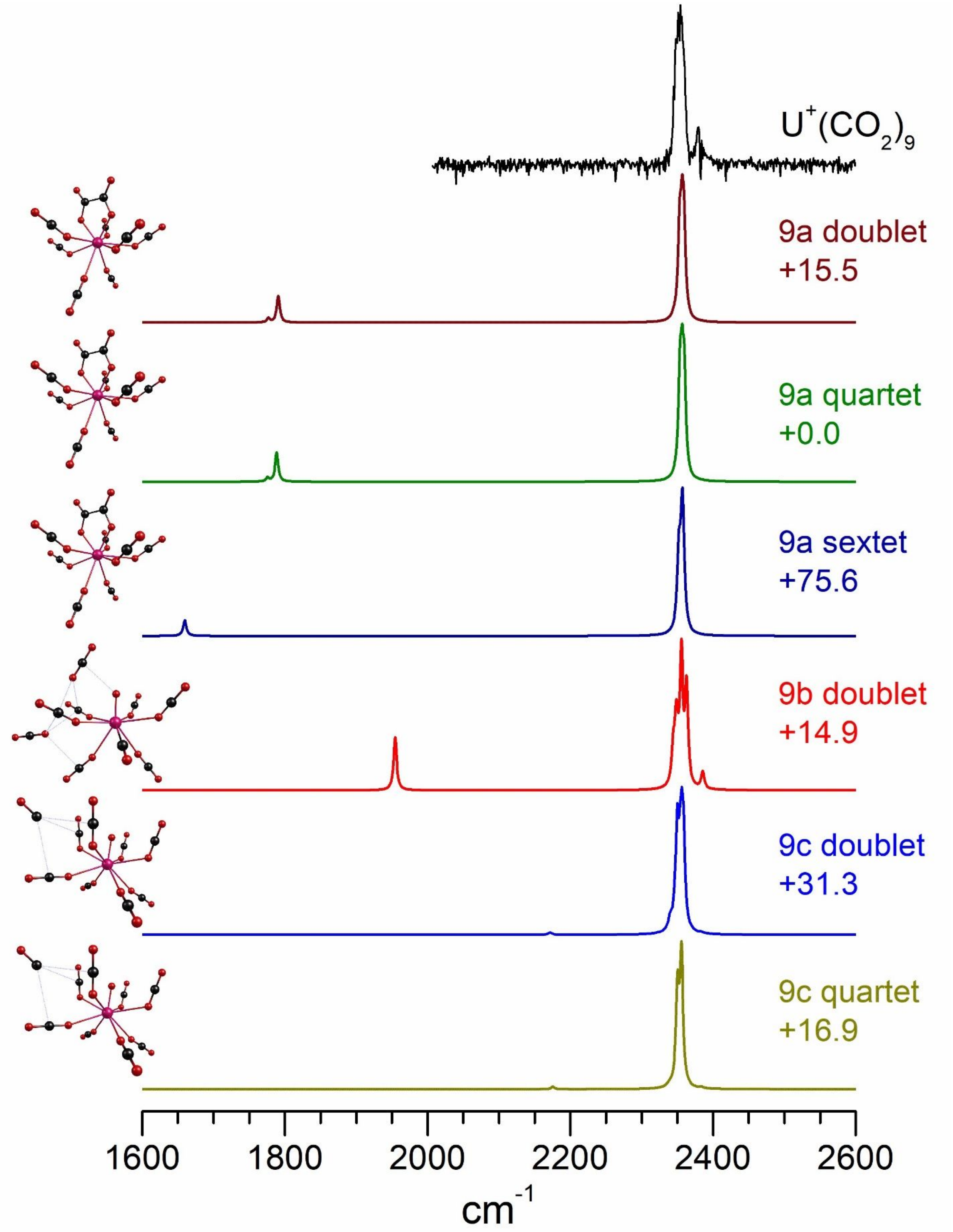

Figure S48. Experimental IR spectrum of $U^{+}(CO_2)_9$ compared with simulated spectra for isomers 9a, 9b and 9c . Relative energies (kcal/mol) are shown next to each spectrum.

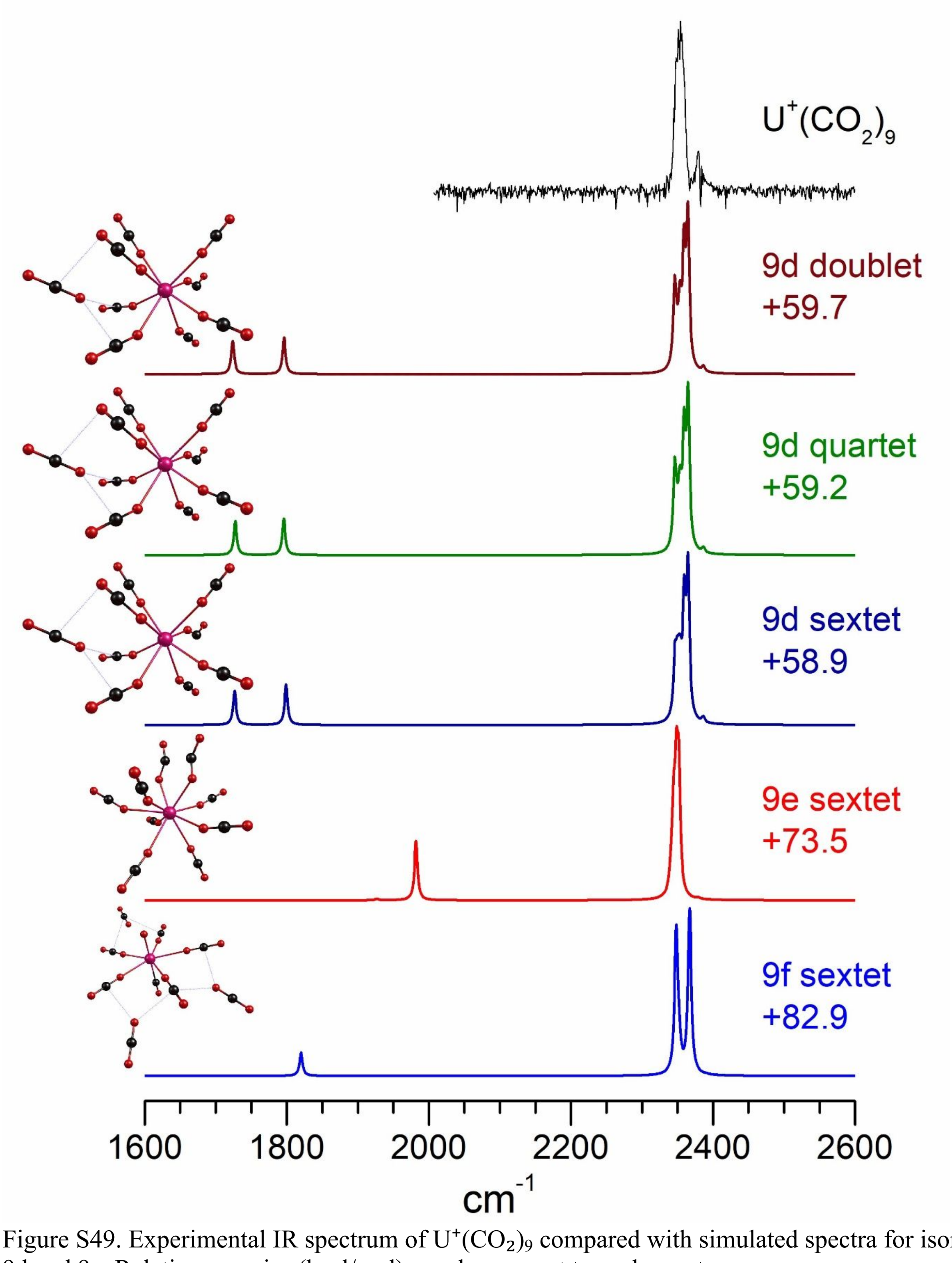

Figure S49. Experimental IR spectrum of $U^{+}(CO_2)_9$ compared with simulated spectra for isomers 9d and 9e. Relative energies (kcal/mol) are shown next to each spectrum.

Table S76. UO^+ electronic energy calculated at the B3LYP/cc-pVTZ(-pp) level with Stuttgart/Koeln pseudopotential.

2s + 1	E (hartree)	Relative E (kcal/mol)
2	-549.787704	+15.4
4	-549.812283	+0.0
6	-549.689731	+76.9

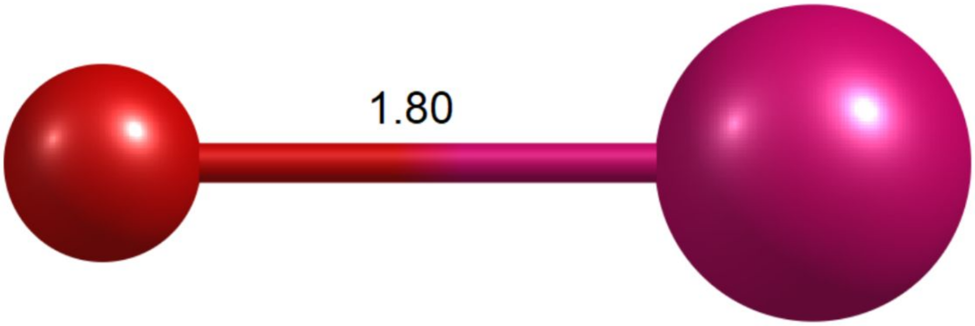

Figure S50.The optimized geometry of UO^+ doublet followed by its predicted frequencies(cm^{-1}) and IR intensities (km/mol).

Frequency (cm^{-1})	Intensity (km/mol)
843.8214	241.6122

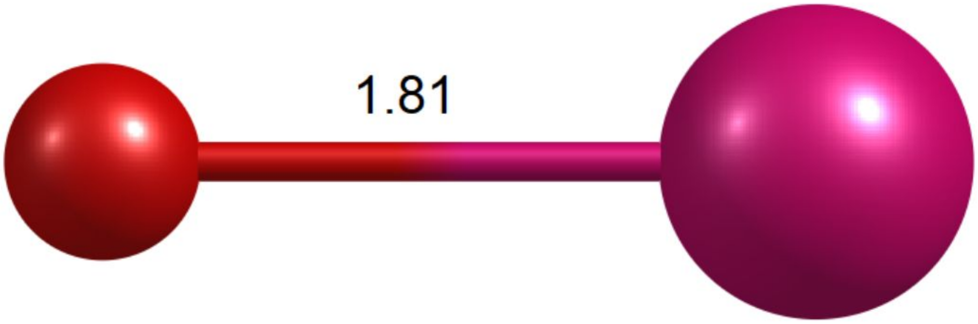

Figure S51.The optimized geometry of UO^+ quartet followed by its predicted frequencies (cm^{-1}) and IR intensities (km/mol).

Frequency (cm^{-1})	Intensity (km/mol)
896.6371	212.3595

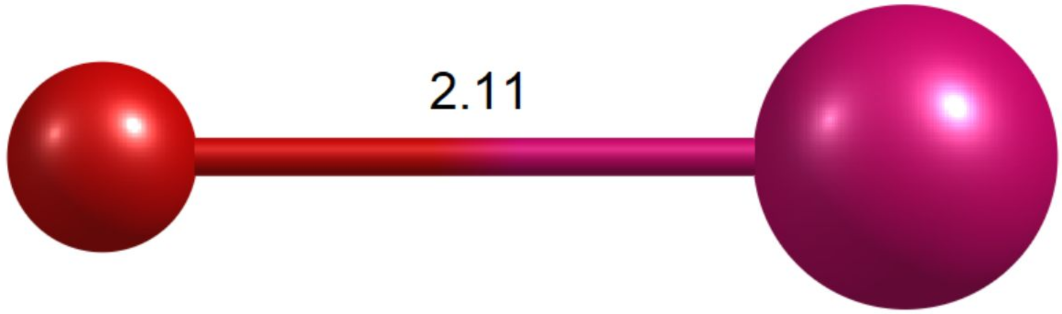

Figure S52.The optimized geometry of UO^+ quartet followed by its predicted frequencies (cm^{-1}) and IR intensities (km/mol).

Frequency (cm^{-1})	Intensity (km/mol)
603.5545	89.5883

Table S77. $UO^+(CO_2)$ electronic energy calculated at the B3LYP/cc-pVTZ(-pp) level with Stuttgart/Koeln pseudopotential.

Isomer	2s + 1	Energy (hartree)	Rel. E (kcal/mol)	BDE (CO_2) (kcal/mol)	BDE (CO) (kcal/mol)
1a	2	-738.557325	+0.0		15.7
1a	4	-738.457303	+62.8		14.9
1b	2	-738.467235	+56.5	19.2	
1b	4	-738.487666	+43.7	16.6	
1c	2	-738.469903	+54.9	20.9	
1d	2	-738.440802	+73.1		
1d	4	-738.465316	+57.7		

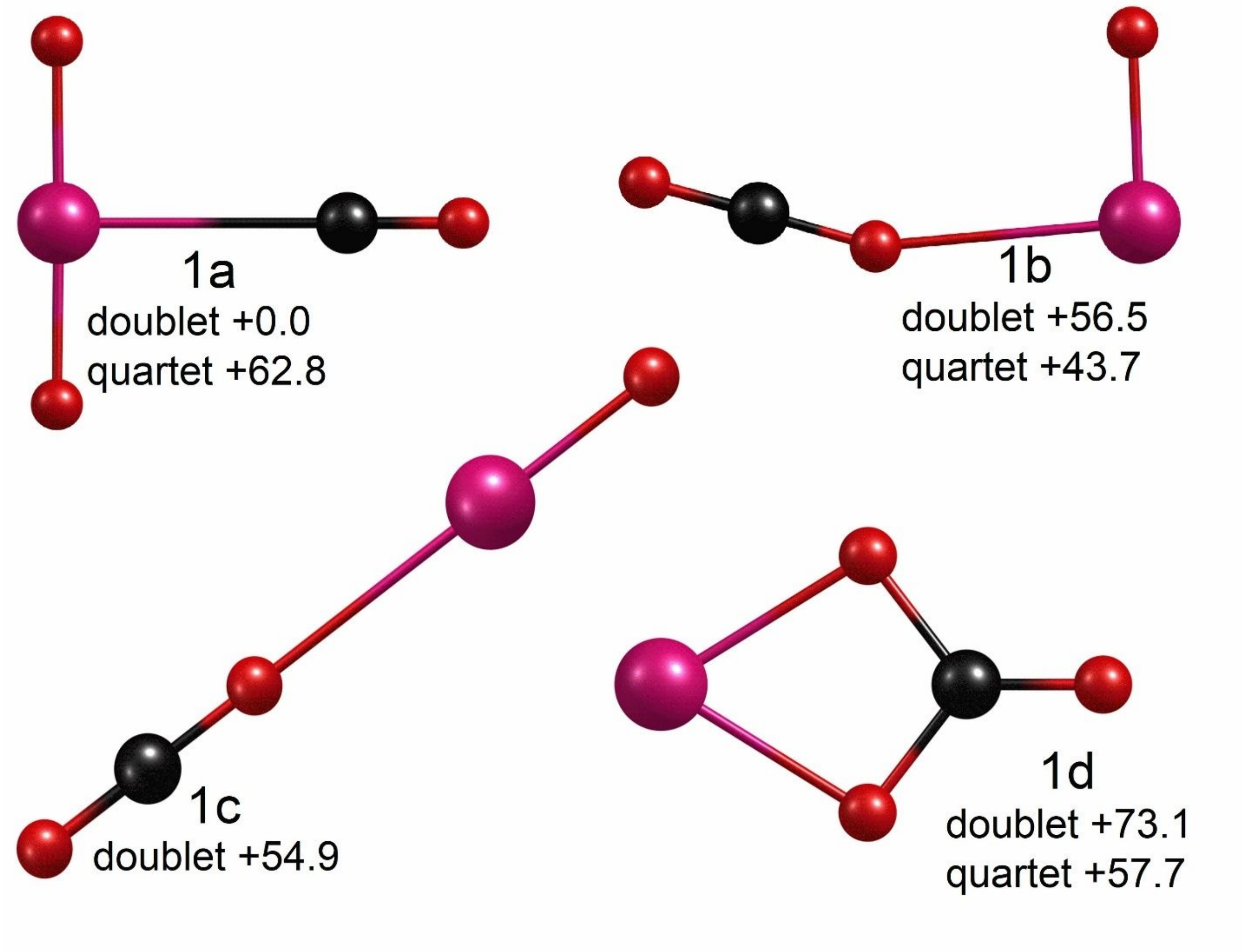

Figure S53. Predicted minimum energy structures of $UO^+(CO_2)$ with energy of each spin state in kcal/mol. The lowest energy spin state of each isomer is shown.

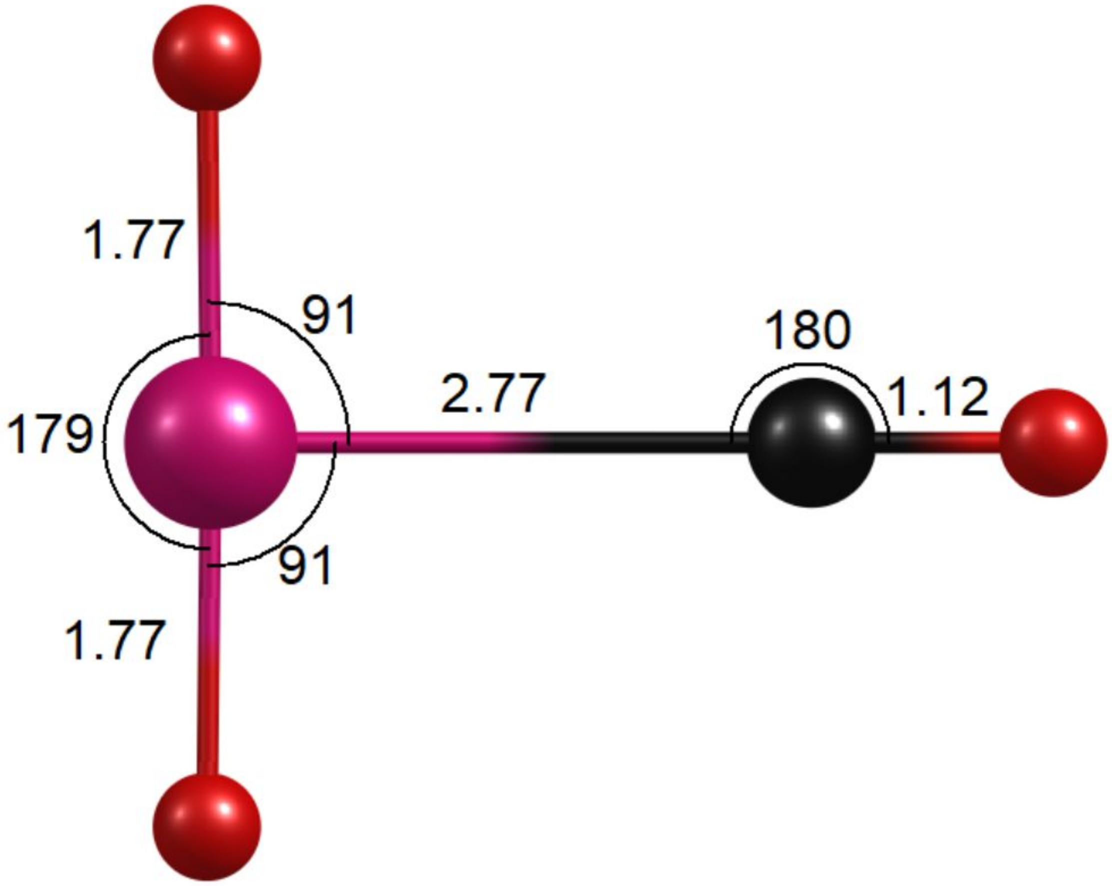

Figure S54. The optimized geometry of isomer 1a-doublet $UO^+(CO_2)$ followed by its predicted frequencies (cm^{-1}) and IR intensities (km/mol).

Frequency (cm^{-1})	Intensity (km/mol)
62.1797	0.0672
143.3614	32.7346
154.338	8.4206
185.4006	24.3486
192.9059	0.009
246.5055	1.7985
910.4966	4.3167
973.7201	383.3245
2295.8099	70.7549

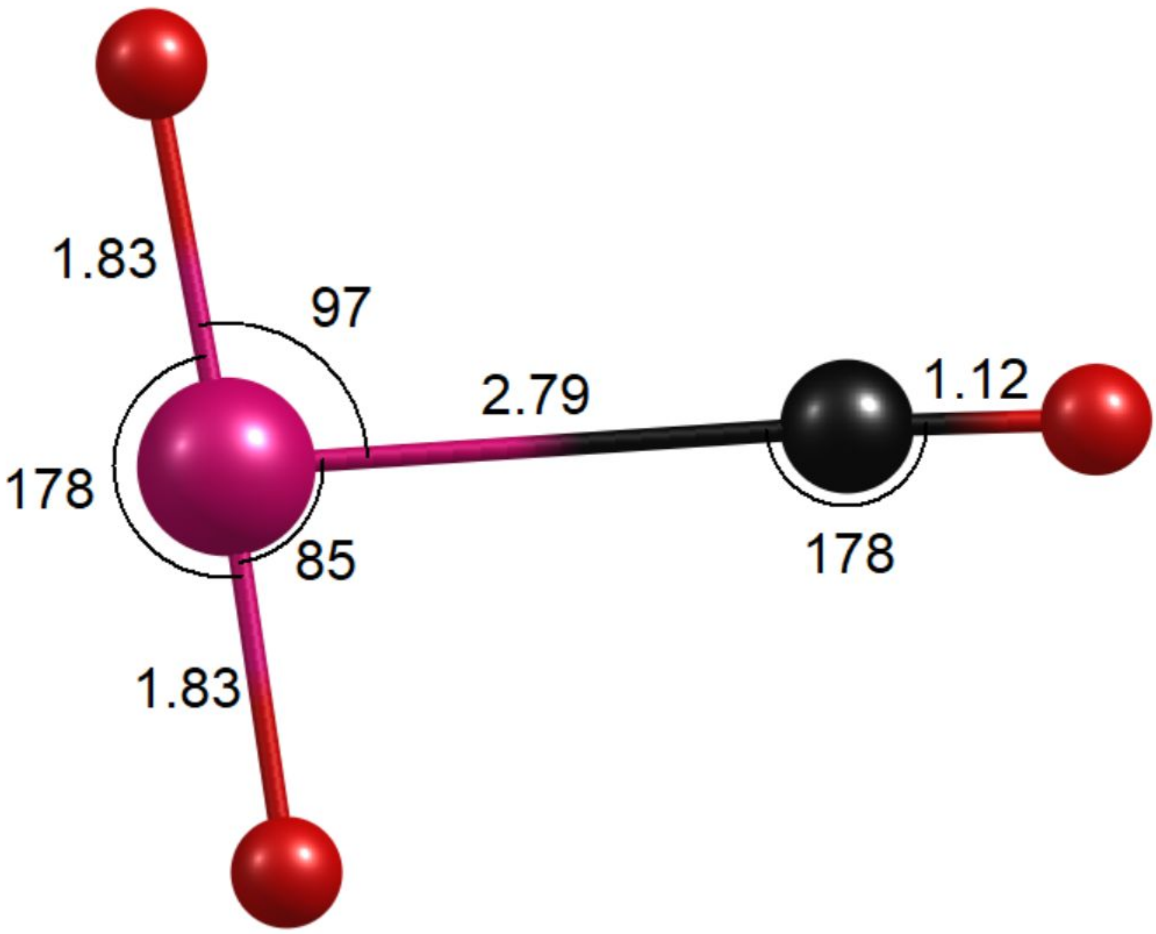

Figure S55. The optimized geometry of isomer 1a-quartet $UO^+(CO_2)$ followed by its predicted frequencies (cm^{-1}) and IR intensities (km/mol).

Frequency (cm^{-1})	Intensity (km/mol)
52.0804	0.108
131.2721	25.6763
149.0018	10.4661
169.887	11.9547
190.7049	0.0063
243.4726	3.8309
663.1576	0.0047
756.2663	3.356
2291.4004	82.0573

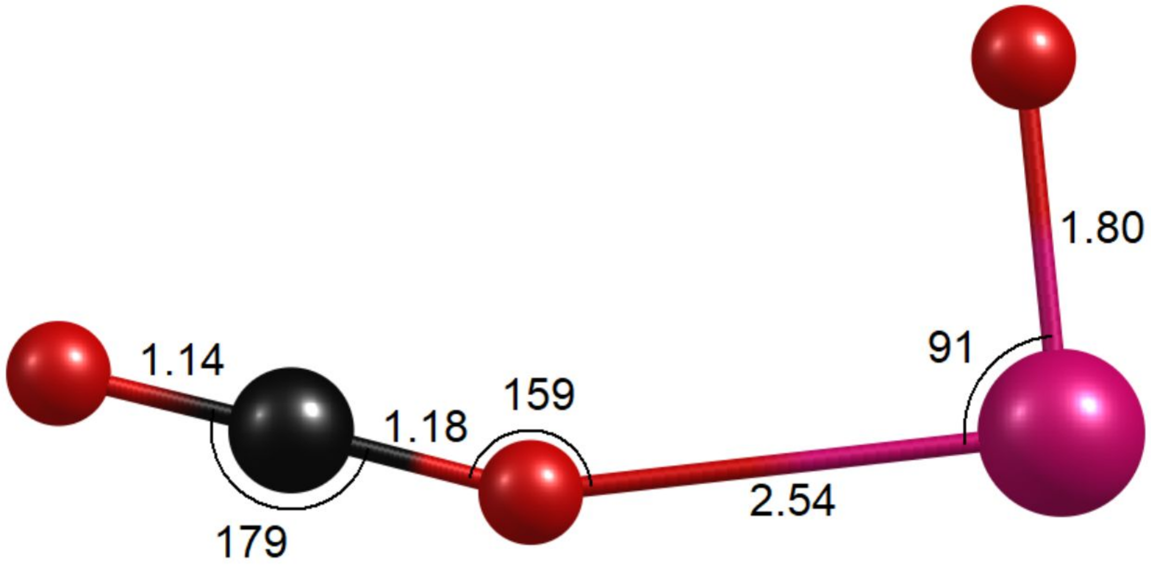

Figure S56. The optimized geometry of isomer 1b-doublet $UO^+(CO_2)$ followed by its predicted frequencies (cm^{-1}) and IR intensities (km/mol).

Frequency (cm^{-1})	Intensity (km/mol)
39.0687	9.077
66.4343	1.4451
107.8866	13.6913
163.4801	12.7494
645.1379	29.5351
645.6072	28.909
861.3558	327.1824
1369.2044	65.1124
2435.0287	1065.4108

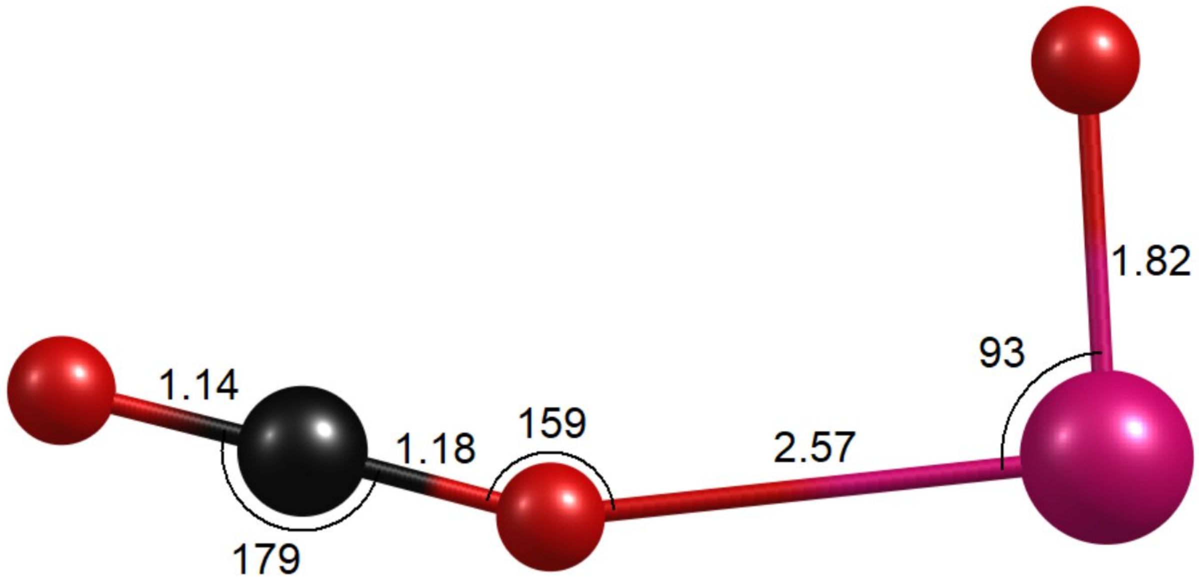

Figure S57. The optimized geometry of isomer 1b-quartet $UO^+(CO_2)$ followed by its predicted frequencies (cm^{-1}) and IR intensities (km/mol).

Frequency (cm^{-1})	Intensity (km/mol)
37.173	8.7599
73.9343	2.4986
119.1824	14.0189
165.7262	11.3312
647.8078	30.6897
650.3547	32.2212
868.2139	261.9927
1371.0083	55.1699
2436.0786	1012.7694

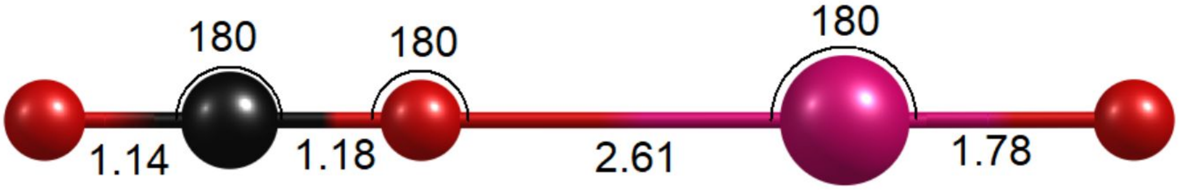

Figure S58. The optimized geometry of isomer 1c-doublet $UO^+(CO_2)$ followed by its predicted frequencies (cm^{-1}) and IR intensities (km/mol).

Frequency (cm^{-1})	Intensity (km/mol)
29.0999	11.3484
65.5881	0.9836
66.2129	0.9616
145.6783	12.1169
646.7875	28.6386
646.8331	28.841
934.551	212.1521
1367.9911	81.6048
2434.584	990.3148

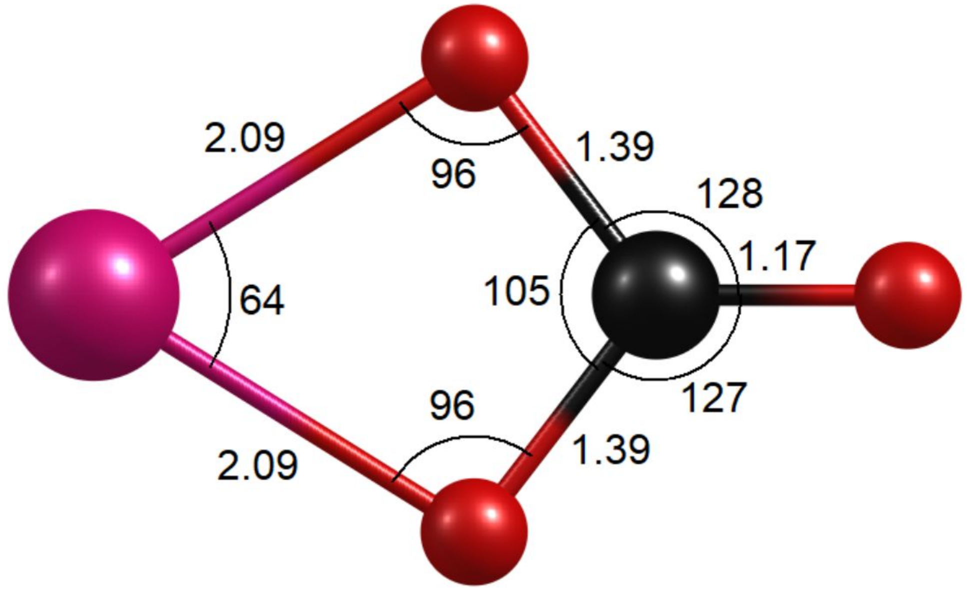

Figure S59. The optimized geometry of isomer 1d-doublet $UO^+(CO_2)$ followed by its predicted frequencies (cm^{-1}) and IR intensities (km/mol).

Frequency (cm^{-1})	Intensity (km/mol)
126.1994	2.0375
305.6291	0.4527
376.3566	41.6649
595.0851	6.2331
771.6554	22.324
773.4886	153.9715
901.0532	182.9347
956.3483	187.9166
1939.3044	649.7228

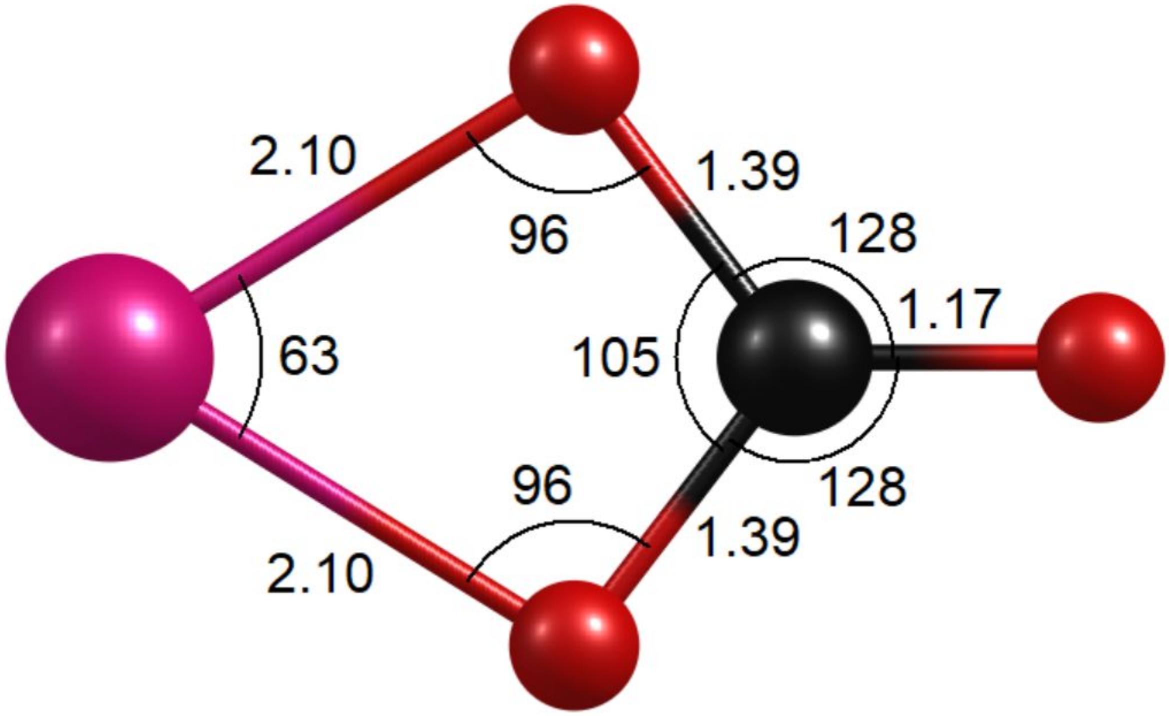

Figure S60. The optimized geometry of isomer 1d-quartet $UO^+(CO_2)$ followed by its predicted frequencies (cm^{-1}) and IR intensities (km/mol).

Frequency (cm^{-1})	Intensity (km/mol)
126.9024	2.0899
299.1575	0.7247
381.0037	39.6174
595.2454	5.5499
771.6178	146.105
776.9571	27.7753
903.5766	181.3351
953.8676	186.671
1936.6531	645.4257

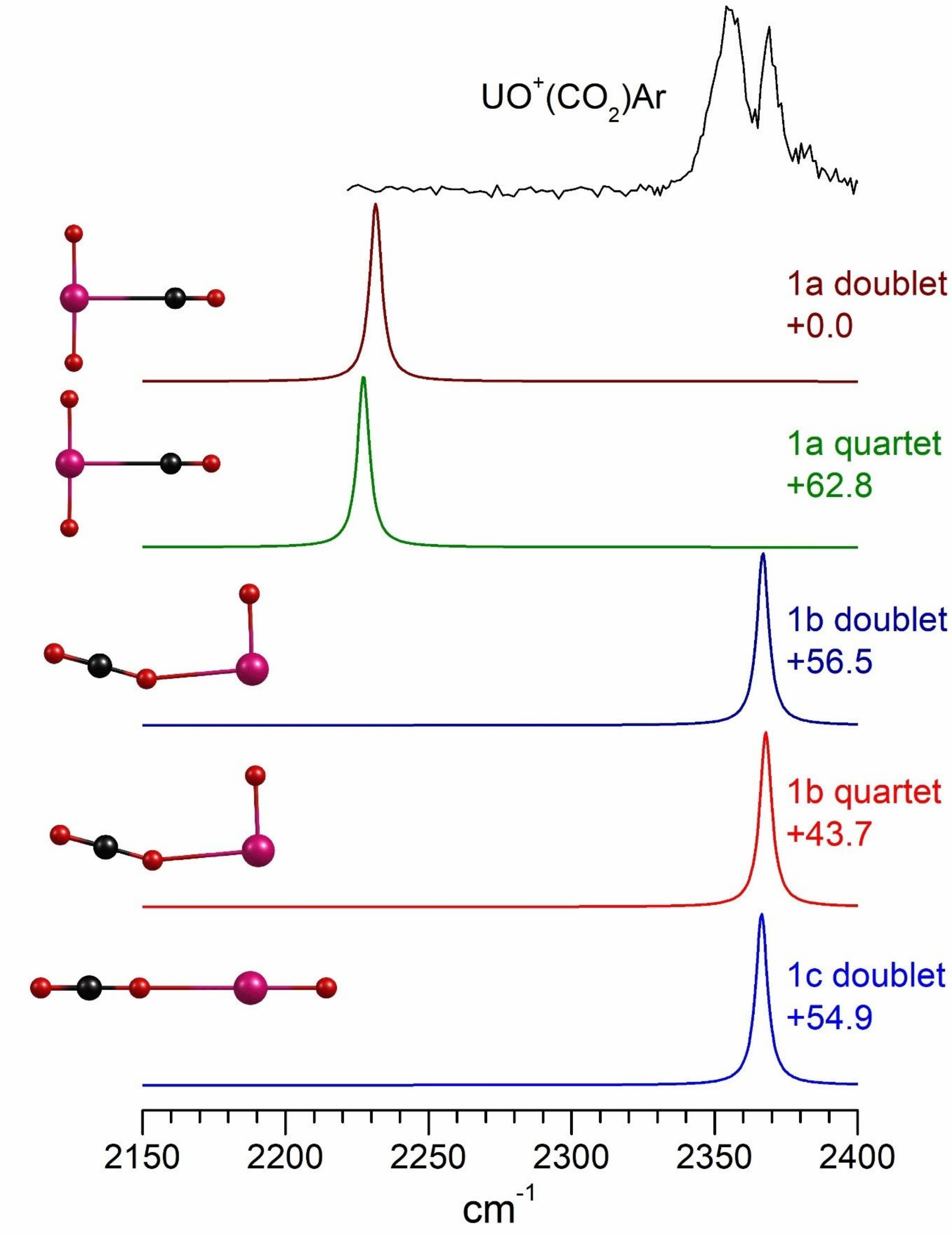

Figure S61. Experimental IR spectrum of $UO^{+}(CO_2)Ar$ with simulated spectra for isomers 1a,1b, and 1c. Relative energies (kcal/mol) are shown next to each spectrum.

Table S78. $UO^+(CO_2)_2$ electronic energy calculated at the B3LYP/cc-pVTZ(-pp) level with Stuttgart/Koeln pseudopotential.

Isomer	2s + 1	Energy (hartree)	Rel. E (kcal/mol)	BDE (CO_2) (kcal/mol)	BDE (CO) (kcal/mol)	BDE (oxalate) (kcal/mol)
2a	2	-927.232105	+0.0	16.3	14.3	
2a	4	-927.131987	+62.8	16.2	13.8	
2b	2	-927.159853	+45.3			46.2
2b	4	-927.123175	+68.4			8.2
2c	2	-927.137818	+59.2	13.6		
2c	4	-927.160732	+44.8	15.2		
2d	2	-927.121719	+69.3	20.1		
2d	4	-927.146047	+54.0	20.0		

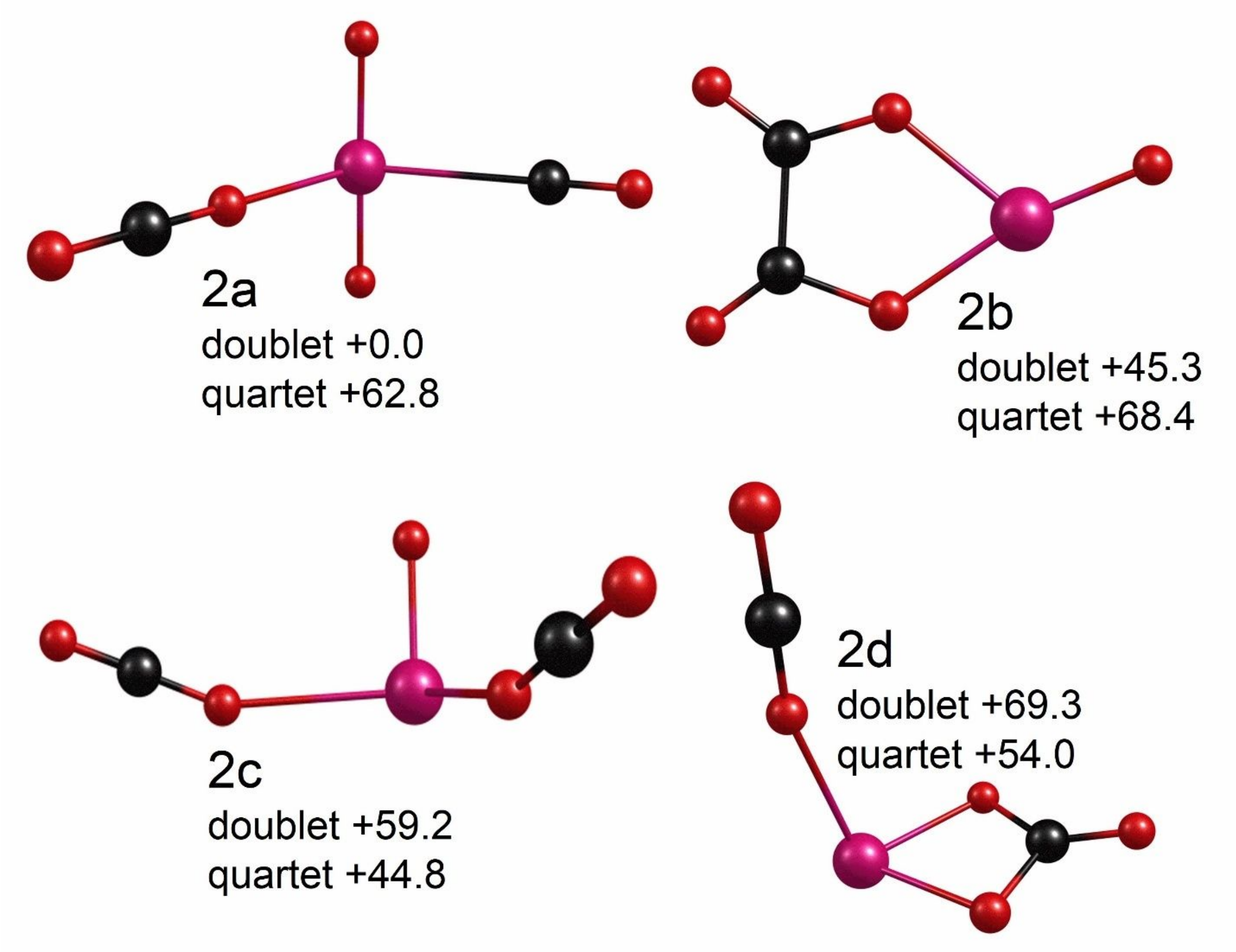

Figure S62. Predicted minimum energy structures of $UO^+(CO_2)_2$ with energy of each spin state in kcal/mol. The lowest energy spin state of each isomer is shown.

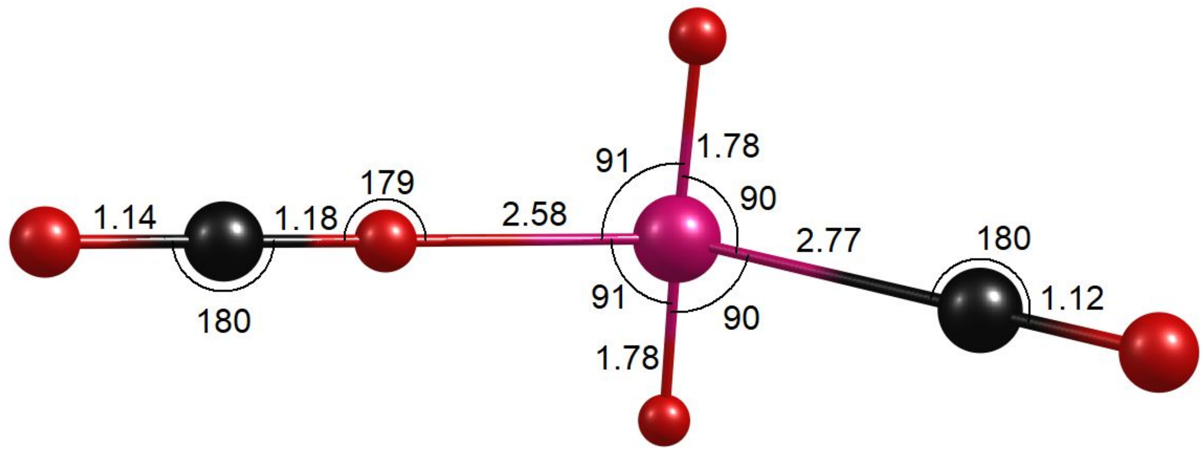

Figure S63. The optimized geometry of isomer 2a-doublet $UO^+(CO_2)_2$ followed by its predicted frequencies (cm^{-1}) and IR intensities (km/mol).

Frequency (cm^{-1})	Intensity (km/mol)
19.1045	0.1072
28.0308	0.1569
59.124	0.0362
68.9134	0.0148
121.0515	0
145.7831	7.4013
152.9148	4.3144
168.018	32.7837
188.8861	28.8253
198.2191	11.5094
246.7187	1.8186
644.5749	29.901
651.6222	35.1081
894.658	5.0555
957.2371	388.7839
1380.0164	59.9141
2287.5402	101.9036
2442.9609	1000.9138

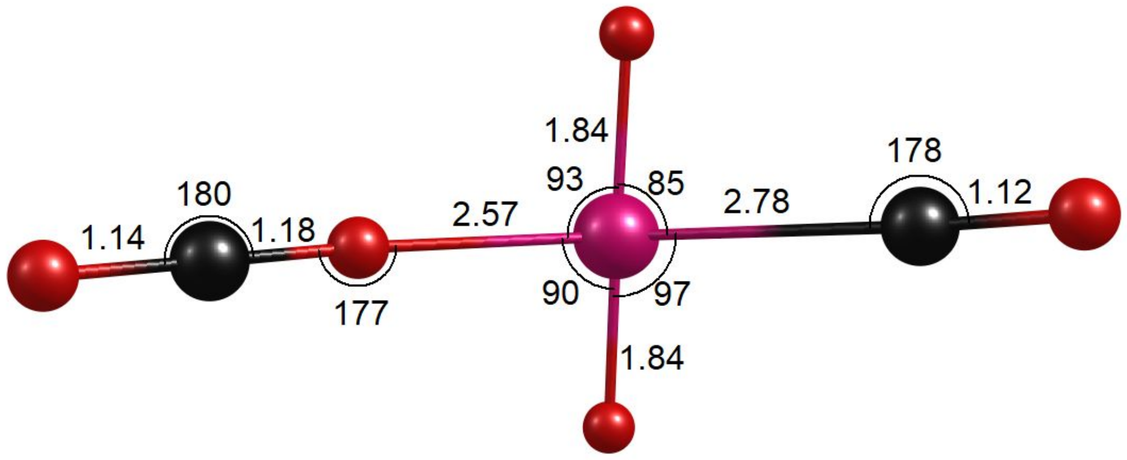

Figure S64. The optimized geometry of isomer 2a-quartet $UO^+(CO_2)_2$ followed by its predicted frequencies (cm^{-1}) and IR intensities (km/mol).

Frequency (cm^{-1})	Intensity (km/mol)
13.2284	0.0671
27.7014	0.1273
49.4328	0.0575
67.235	0.0141
115.2012	0.1942
141.3527	10.2274
148.2108	4.627
160.0414	17.6289
173.2357	30.6678
192.1471	1.9956
244.1048	4.0041
644.6262	29.2359
652.7583	33.3074
652.9549	1.427
741.1139	4.6303
1378.7695	61.648
2282.0047	119.9517
2441.5993	999.8408

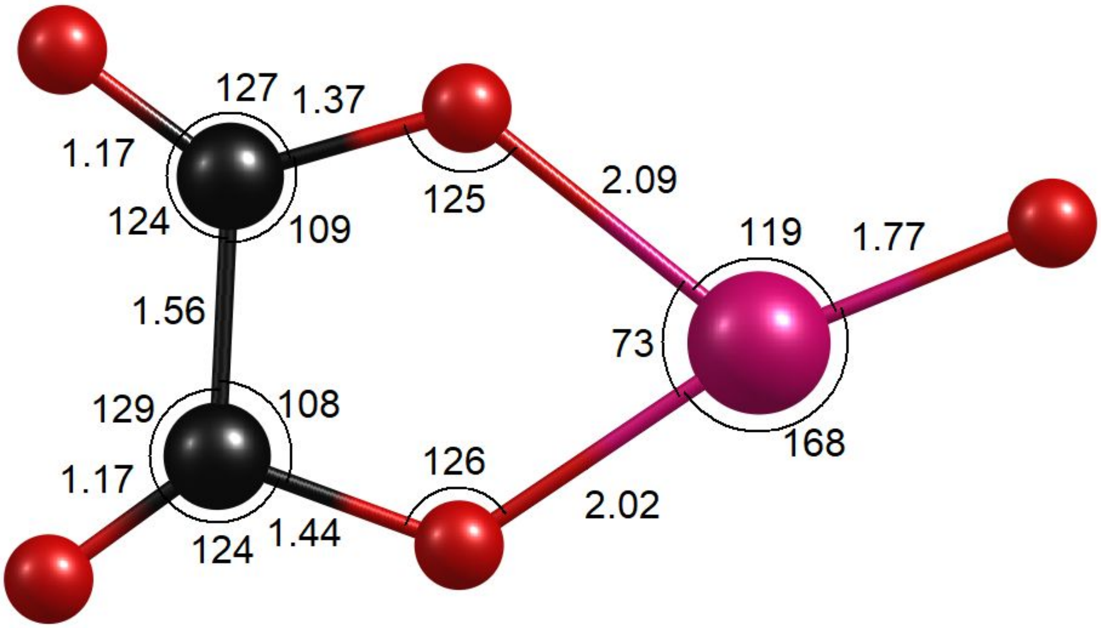

Figure S65. The optimized geometry of isomer 2b-doublet $UO^{+}(CO_2)_2$ followed by its predicted frequencies (cm^{-1}) and IR intensities (km/mol).

Frequency (cm^{-1})	Intensity (km/mol)
72.0616	10.1121
77.8898	20.1023
97.5689	3.1125
150.0105	5.081
307.0781	16.8617
316.271	16.8501
343.784	0.5956
469.718	44.6935
523.0133	9.0114
584.3585	4.124
653.785	147.1033
790.567	0.1002
815.2483	286.1758
876.55	91.6496
941.3491	296.7599
1072.4064	450.6267
1907.6779	311.2966
1953.1453	325.8002

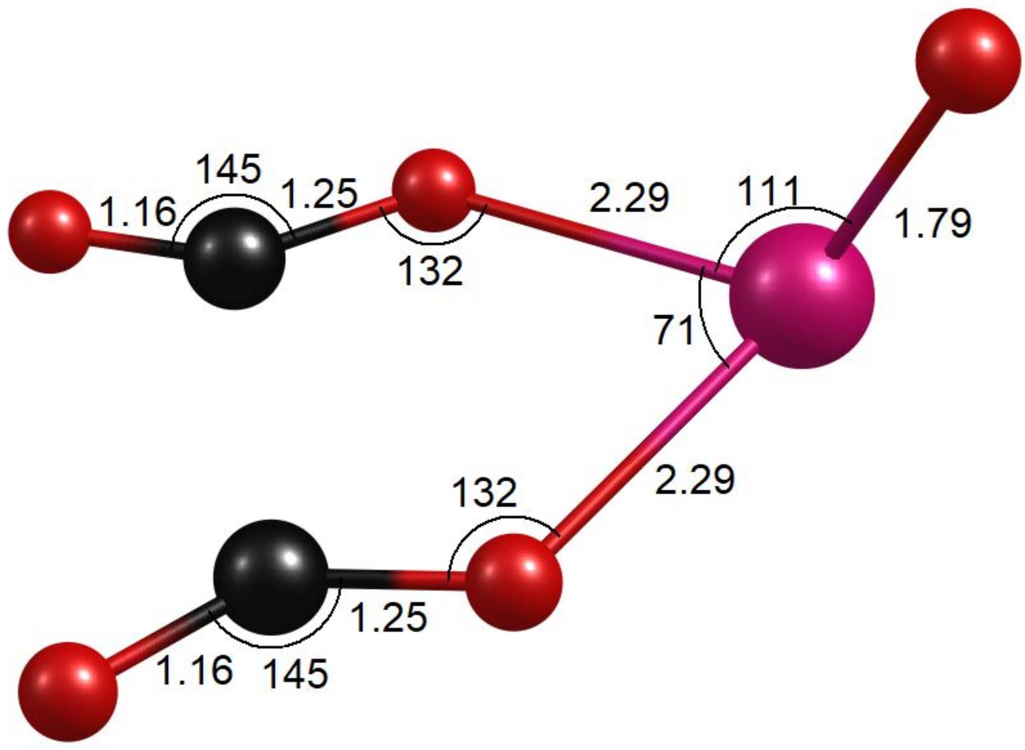

Figure S66. The optimized geometry of isomer 2b-quartet $UO^+(CO_2)_2$ followed by its predicted frequencies (cm^{-1}) and IR intensities (km/mol).

Frequency (cm^{-1})	Intensity (km/mol)
51.0791	10.8388
53.1755	16.5154
96.5987	3.6602
115.5603	6.5332
174.9013	2.5843
204.1643	5.4918
232.2196	15.6627
310.3887	0.4384
346.9738	49.1728
449.704	27.3568
626.975	5.842
641.595	59.6145
647.287	323.6776
929.3942	213.2617
1095.5982	80.384
1241.3925	222.943
2013.6058	65.2147
2075.8791	1058.8333

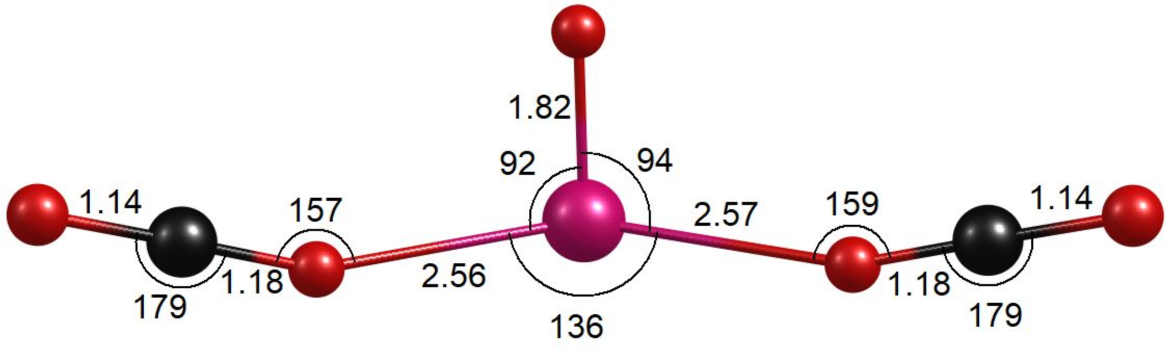

Figure S67. The optimized geometry of isomer 2c-doublet $UO^+(CO_2)_2$ followed by its predicted frequencies (cm^{-1}) and IR intensities (km/mol).

Frequency (cm^{-1})	Intensity (km/mol)
11.1198	0.033
26.4977	8.249
36.5436	5.459
66.4477	0.2291
71.6742	0.786
97.3313	5.4484
130.2809	15.1027
161.6322	3.8319
170.039	18.6854
647.2808	26.1914
648.6033	25.1423
649.3551	13.1739
649.9856	52.2095
861.5298	320.2735
1371.6033	86.0964
1372.9542	21.1794
2428.6156	1745.3652
2439.3037	403.6375

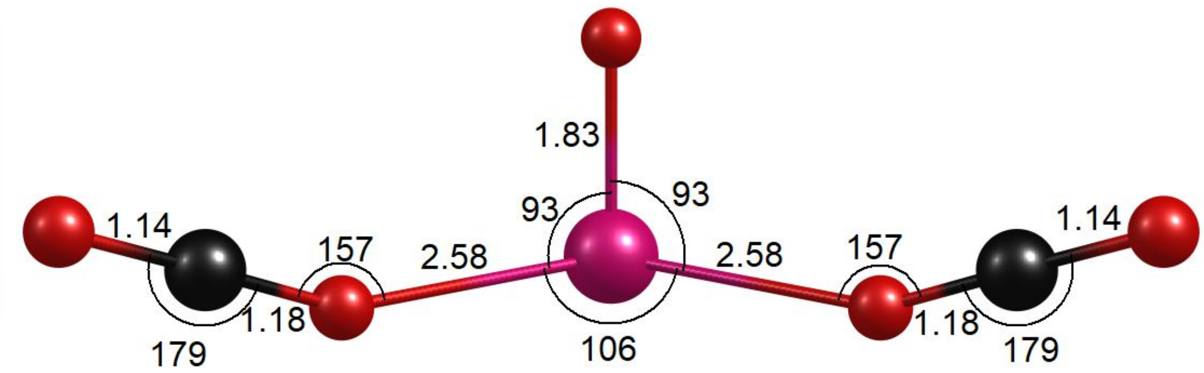

Figure S68. The optimized geometry of isomer 2c-quartet $UO^{+}(CO_2)_2$ followed by its predicted frequencies (cm^{-1}) and IR intensities (km/mol).

Frequency (cm^{-1})	Intensity (km/mol)
13.7794	0.0329
29.9261	8.037
33.1332	6.7447
66.0671	0.228
72.1832	0.341
109.6082	13.765
118.9214	16.7779
164.2976	5.3261
166.4829	13.8828
649.8116	13.5385
650.6611	45.2708
651.7075	13.2718
653.1708	48.2012
858.2898	313.5526
1372.0082	66.0973
1373.2593	36.1879
2425.1425	1386.8352
2441.1196	708.3839

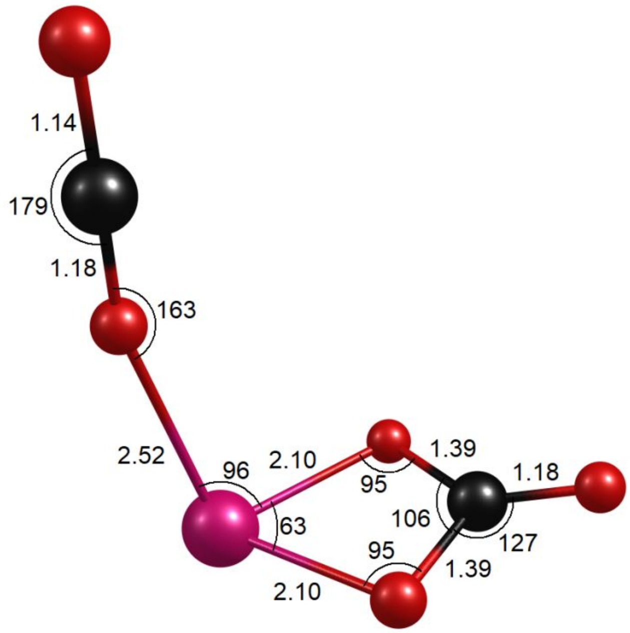

Figure S69. The optimized geometry of isomer 2d-doublet $UO^+(CO_2)_2$ followed by its predicted frequencies (cm^{-1}) and IR intensities (km/mol).

Frequency (cm^{-1})	Intensity (km/mol)
25.5326	5.2844
38.8556	0.2041
83.4243	0.4124
87.6114	0.2829
137.2051	2.6667
179.5825	18.0365
313.3794	1.2735
373.4429	45.382
607.9518	2.9848
647.1219	30.5305
647.8422	31.6848
768.1296	161.809
777.0739	20.7962
910.0281	175.1502
972.8662	190.5629
1370.046	86.4104
1920.8176	685.3228
2439.1782	988.0322

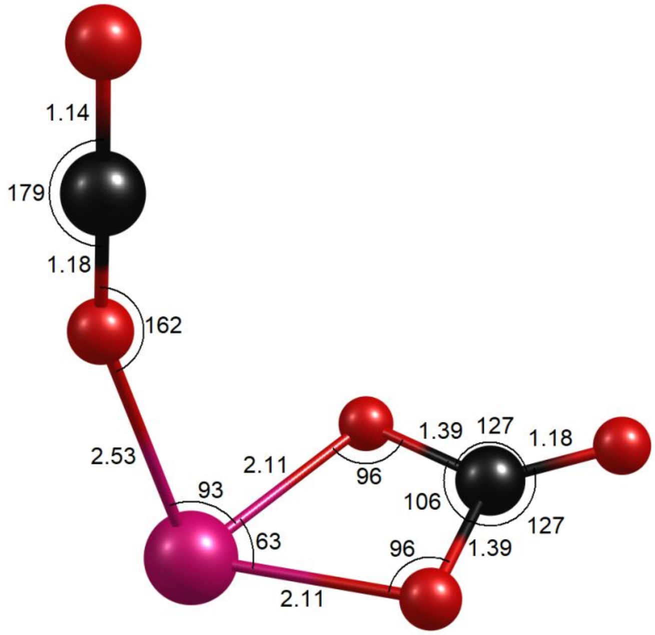

Figure S70. The optimized geometry of isomer 2d-quartet $UO^{+}(CO_2)_2$ followed by its predicted frequencies (cm^{-1}) and IR intensities (km/mol).

Frequency (cm^{-1})	Intensity (km/mol)
26.6889	5.2765
42.9041	0.2804
85.9556	0.3277
93.8826	0.2448
140.2772	2.827
179.9795	16.9734
307.4488	1.7201
376.1412	44.8789
608.8101	2.5905
646.8912	31.3889
647.706	31.6103
766.9211	146.723
783.2081	24.9489
912.4667	173.7497
975.2883	194.0329
1370.5182	78.7633
1917.2881	669.3053
2438.6559	994.0782

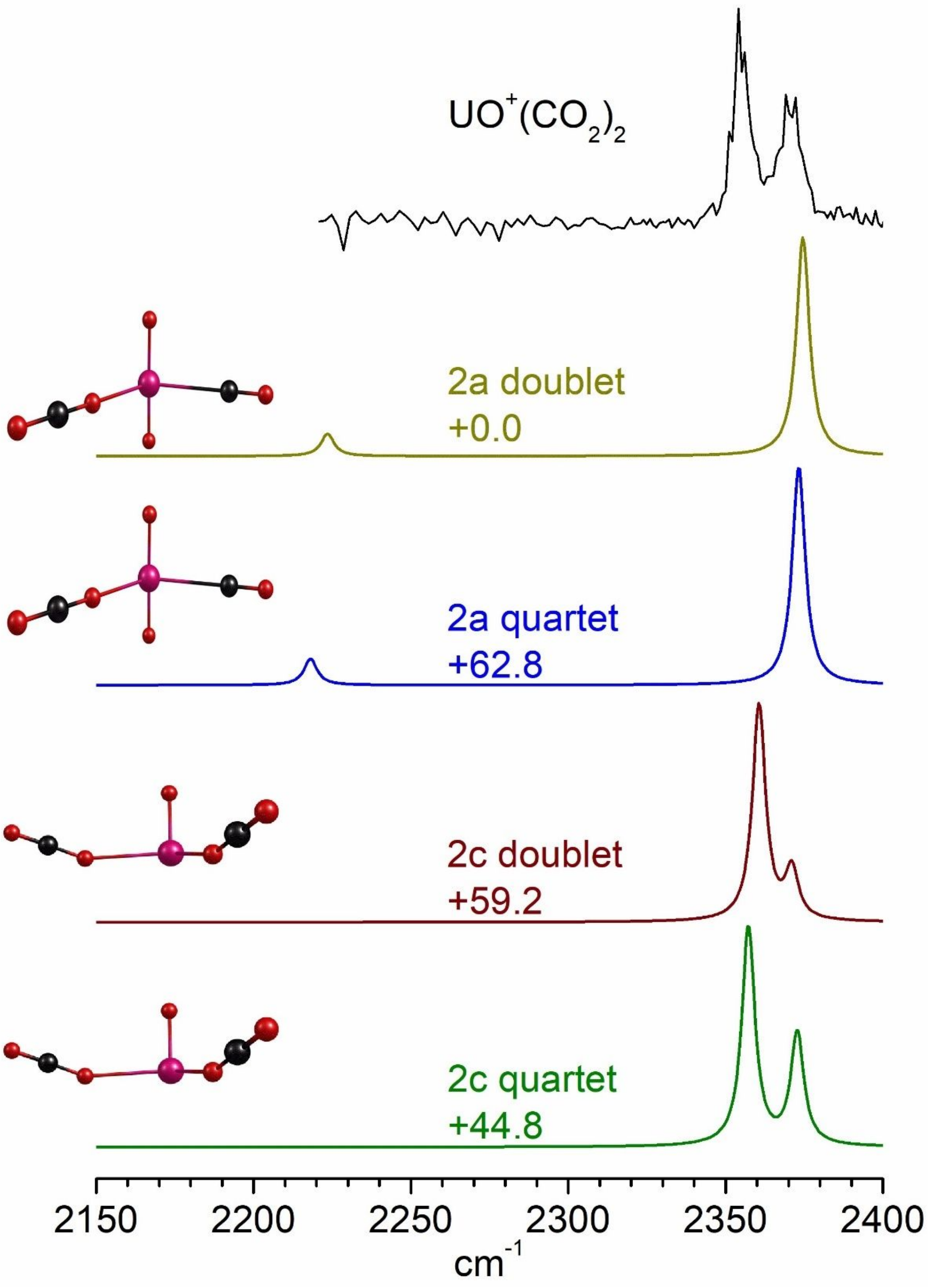

Figure S71. Experimental IR spectrum of $UO^+(CO_2)_2$ compared with simulated spectra for isomers 2a and 2c. Relative energies (kcal/mol) are shown next to each spectrum.

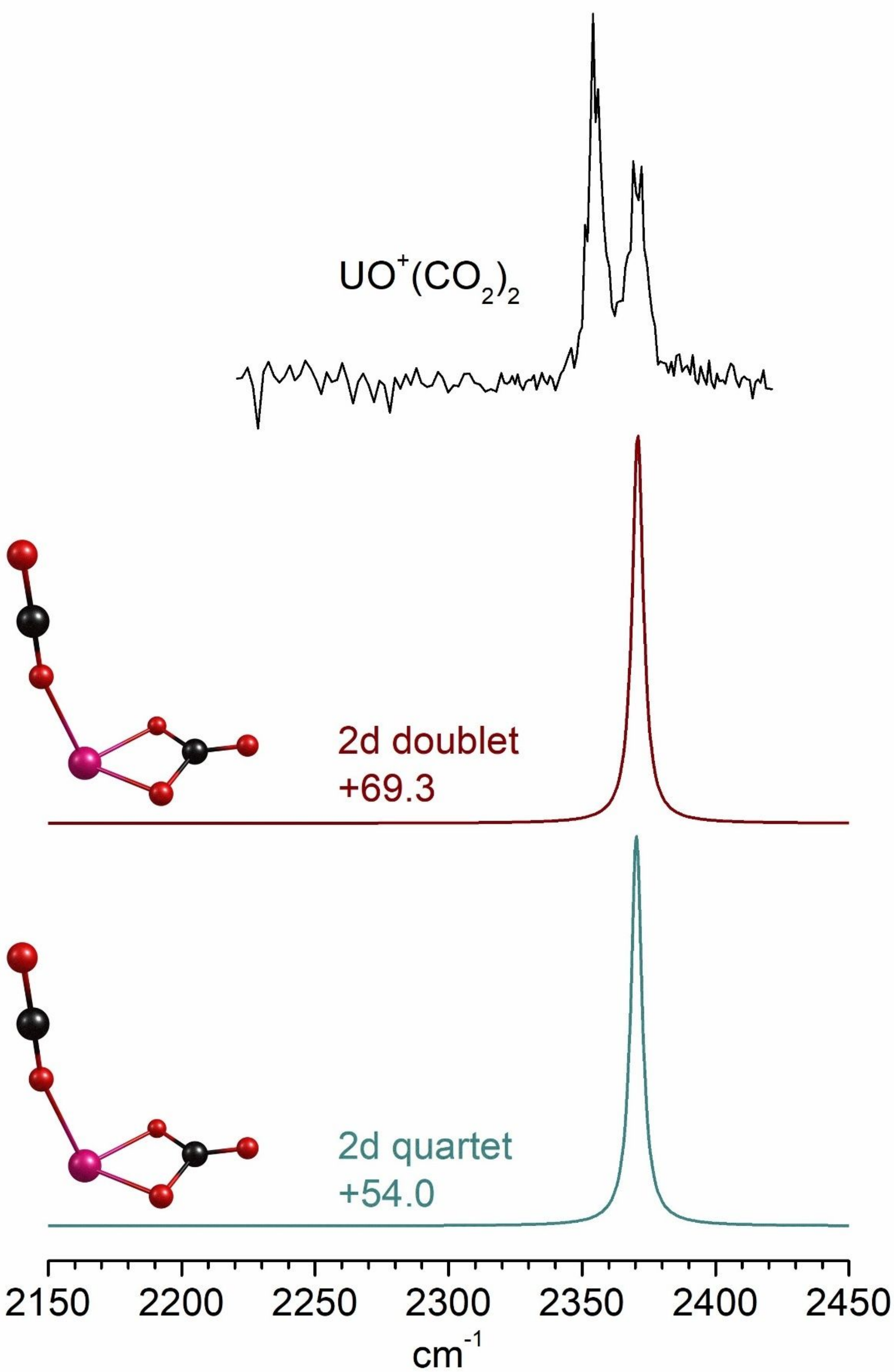

Figure S72. Experimental IR spectrum of $UO^{+}(CO_2)_2$ compared with simulated spectra for isomer 2d. Relative energies (kcal/mol) are shown next to each spectrum.

Table S79. $UO^+(CO_2)_3$ electronic energy calculated at the B3LYP/cc-pVTZ(-pp) level with Stuttgart/Koeln pseudopotential.

Isomer	2s + 1	Energy (hartree)	Rel. E (kcal/mol)	BDE (CO_2) (kcal/mol)	BDE (CO) (kcal/mol)	BDE (oxalate)
3a	2	-1115.904046	+0.0	14.5	13.1	
3a	4	-1115.803788	+62.9	14.4		
3b	2	-1115.840204	+40.1	19.7		47.2
3b	4	-1115.799176	+65.8	17.0		8.6
3c	2	-1115.811279	+58.2	15.4		
3c	4	-1115.831727	+45.4	13.9		
3d	2	-1115.798151	+66.5	17.3		
3d	4	-1115.822217	+51.3	17.1		

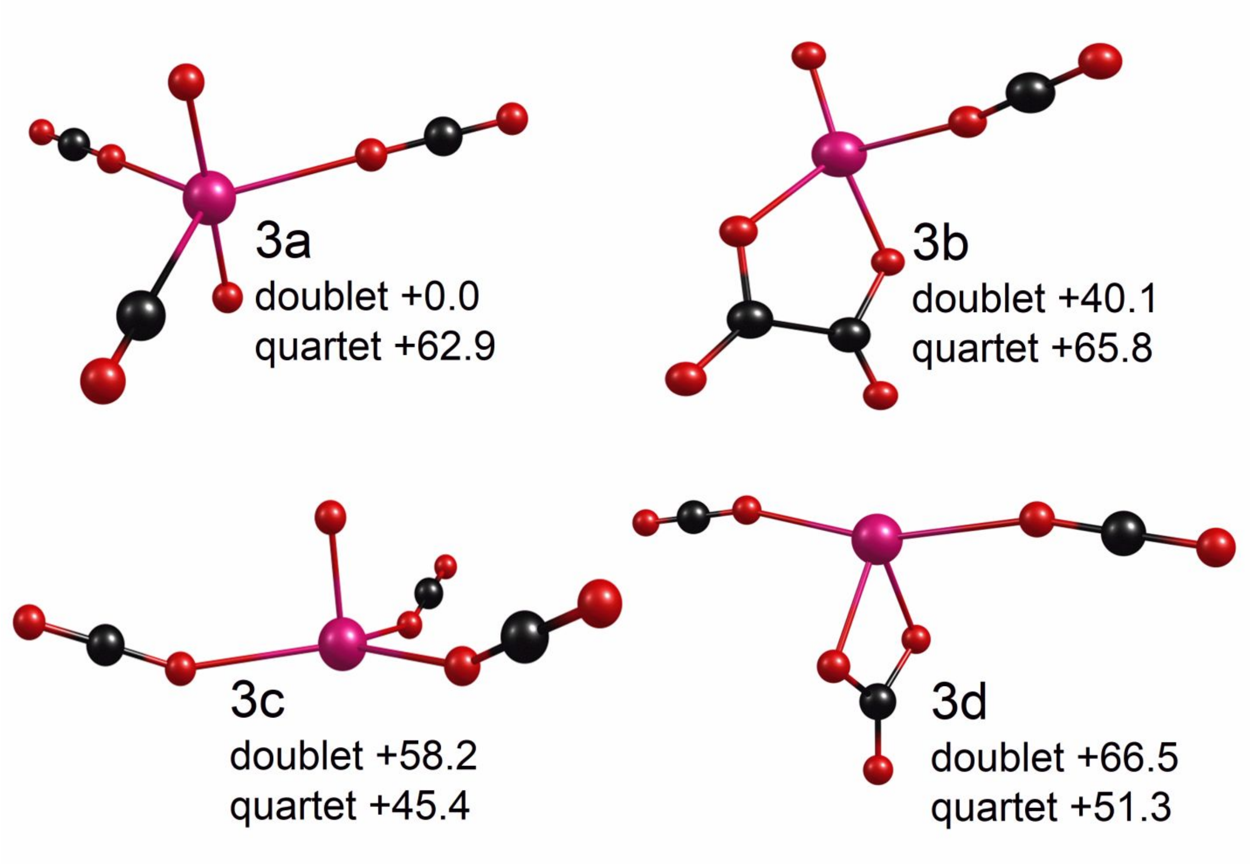

Figure S73. Predicted minimum energy structures of $UO^+(CO_2)_3$ with energy of each spin state in kcal/mol. The lowest energy spin state of each isomer is shown.

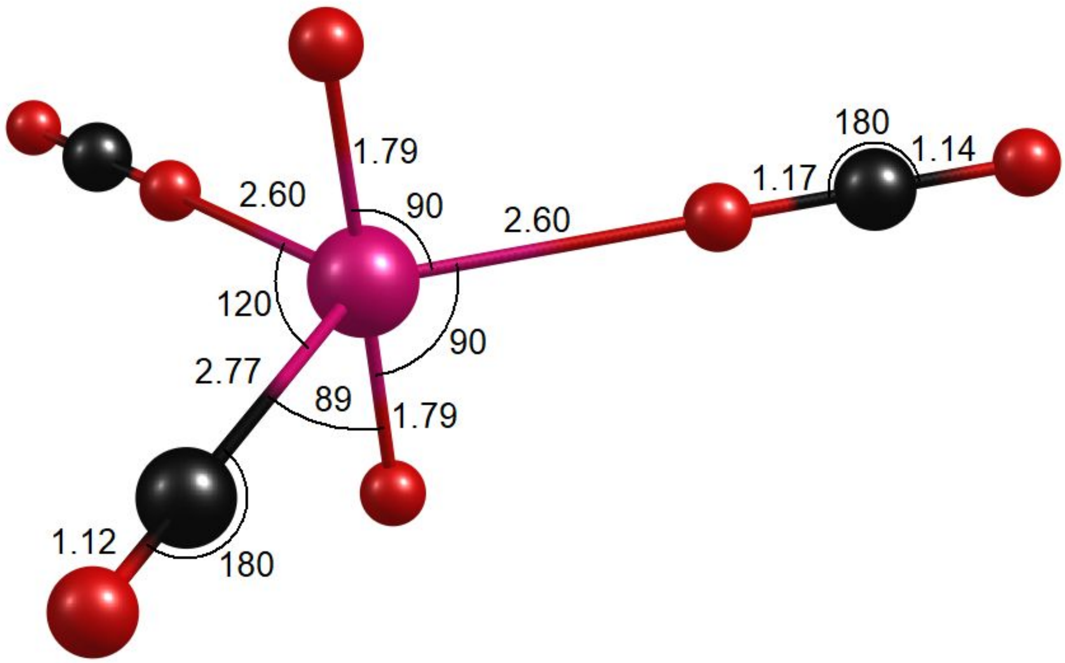

Figure S74. The optimized geometry of isomer 3a-doublet $UO^+(CO_2)_3$ followed by its predicted frequencies (cm^{-1}) and IR intensities (km/mol).

Frequency (cm^{-1})	Intensity (km/mol)	Frequency (cm^{-1})	Intensity (km/mol)
15.1113	0.1386	201.6111	13.1958
17.5986	0.3785	248.7884	1.8714
21.6775	0.1578	646.0597	0
27.162	0	646.5656	58.1984
55.1183	0.0154	652.4983	17.2624
64.1398	0.0158	653.5311	51.6682
68.6792	0.0039	881.1922	0.5555
112.1429	0.0021	943.7481	391.3944
129.3717	0	1380.7129	77.4364
142.118	1.7758	1381.9431	25.9523
146.0617	1.3066	2279.195	138.8376
152.0253	4.0269	2436.8564	1549.5628
183.5234	47.1895	2446.9781	515.5129
191.0971	34.3961		

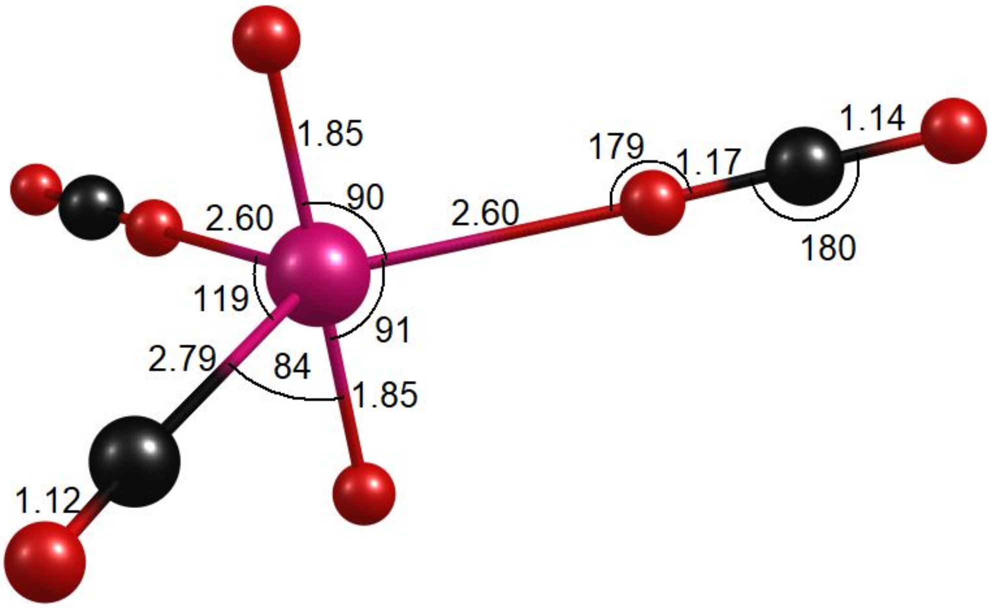

Figure S75. The optimized geometry of isomer 3a-quartet $UO^+(CO_2)_3$ followed by its predicted frequencies (cm^{-1}) and IR intensities (km/mol).

Frequency (cm^{-1})	Intensity (km/mol)	Frequency (cm^{-1})	Intensity (km/mol)
12.0127	0.1373	193.5901	7.0763
15.824	0.0956	246.1805	4.1188
17.1278	0.3448	644.67	3.4773
26.411	0.0001	645.9852	0.5058
47.8344	0.0263	646.716	54.3385
62.3926	0.0009	653.7051	15.2331
65.2525	0.0003	654.6436	52.145
106.2902	0.1087	729.0237	0.6511
123.5123	0.0568	1379.8506	82.5501
140.2494	1.3988	1381.0958	25.4334
144.604	0.6445	2273.1827	167.8421
147.5928	5.0347	2435.9112	1593.3491
172.3168	21.8382	2445.8962	486.7856
174.1887	45.6774		

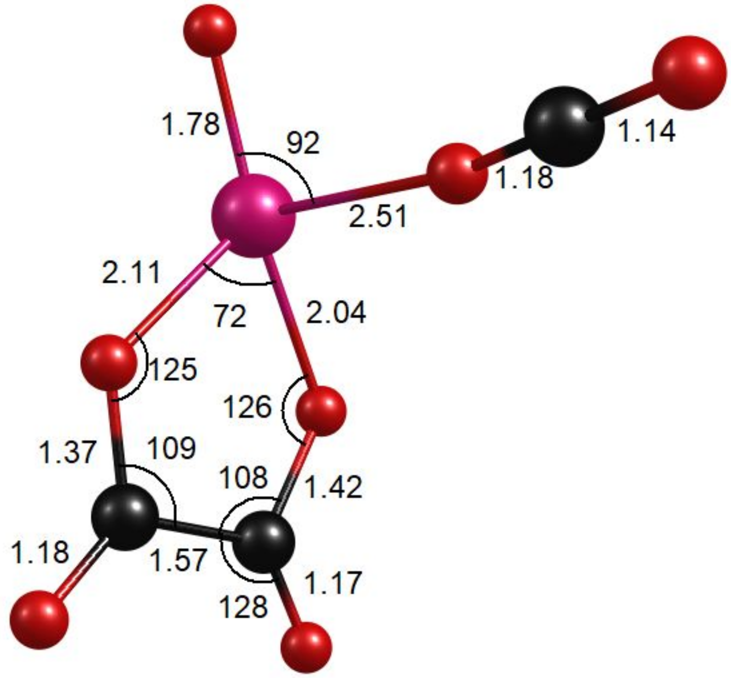

Figure S76. The optimized geometry of isomer 3b-doublet $UO^{+}(CO_2)_3$ followed by its predicted frequencies (cm^{-1}) and IR intensities (km/mol).

Frequency (cm^{-1})	Intensity (km/mol)	Frequency (cm^{-1})	Intensity (km/mol)
22.4778	1.2214	584.6422	7.3953
27.9221	0.2355	642.5148	25.006
64.1127	3.9515	645.1208	32.493
71.9244	4.8345	688.9674	144.2848
87.5023	13.8864	801.3981	0.1419
110.0518	0.6707	822.3663	315.7744
131.6072	7.2824	881.2215	42.9262
152.7414	1.0272	926.5537	289.15
184.3961	27.4924	1091.11	497.5045
306.659	17.707	1377.5191	84.0265
313.9264	17.9796	1896.9219	325.1931
340.8112	1.0384	1933.9396	370.5946
473.1145	47.6239	2444.5707	1038.8461
533.0105	3.2907		

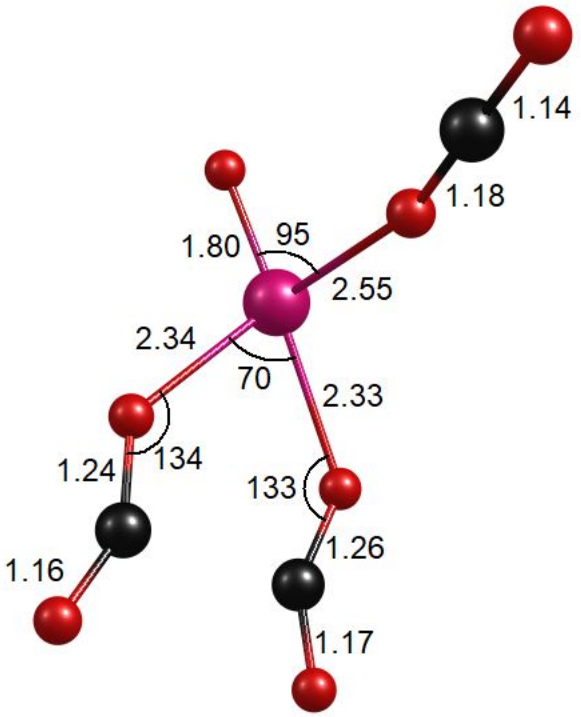

Figure S77. The optimized geometry of isomer 3b-quartet $UO^+(CO_2)_3$ followed by its predicted frequencies (cm^{-1}) and IR intensities (km/mol).

Frequency (cm^{-1})	Intensity (km/mol)	Frequency (cm^{-1})	Intensity (km/mol)
10.8316	4.0012	450.6338	27.2334
22.9823	0.0841	619.3385	184.9749
36.1228	11.3964	628.8905	0.3803
58.7354	10.1342	645.7793	14.4204
74.2863	3.9459	648.9915	31.2672
81.3453	0.9973	655.6612	216.3817
101.9735	1.6393	906.2226	271.2628
117.9263	11.5288	1103.5081	85.7787
158.6245	8.7541	1246.6684	215.5518
173.4185	16.312	1374.3288	72.4157
188.7666	2.1663	2005.3972	158.5133
222.6105	18.591	2083.8	1081.6485
305.1325	0.5453	2440.6928	1020.8122
338.8142	41.7538		

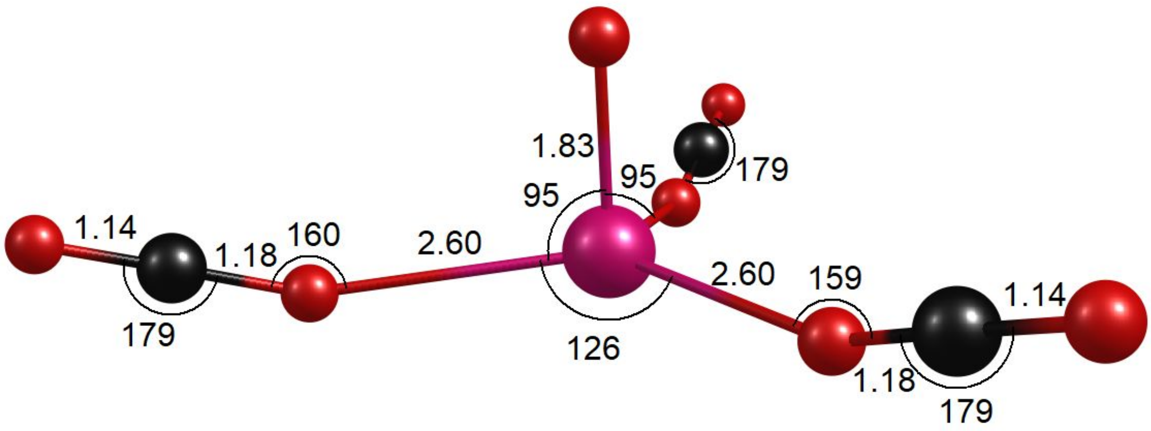

Figure S78. The optimized geometry of isomer 3c-doublet $UO^+(CO_2)_3$ followed by its predicted frequencies (cm^{-1}) and IR intensities (km/mol).

Frequency (cm^{-1})	Intensity (km/mol)	Frequency (cm^{-1})	Intensity (km/mol)
12.282	0.0832	646.4806	7.2854
13.3097	0.1668	646.9736	8.557
16.0066	0.2698	647.8062	64.5071
29.3262	6.5696	648.0555	14.9156
32.6823	4.613	649.6815	35.0116
61.7043	0.0002	650.6228	46.6468
66.412	0.0403	858.7815	357.8119
67.361	0.1683	1372.9679	62.4903
84.4977	0.1145	1373.2496	54.3146
109.4658	13.4628	1374.616	8.3254
124.7277	13.0583	2426.6482	1435.152
150.1844	0.2704	2429.3938	1712.0552
155.0818	10.8328	2444.3581	133.6734
160.1301	16.904		

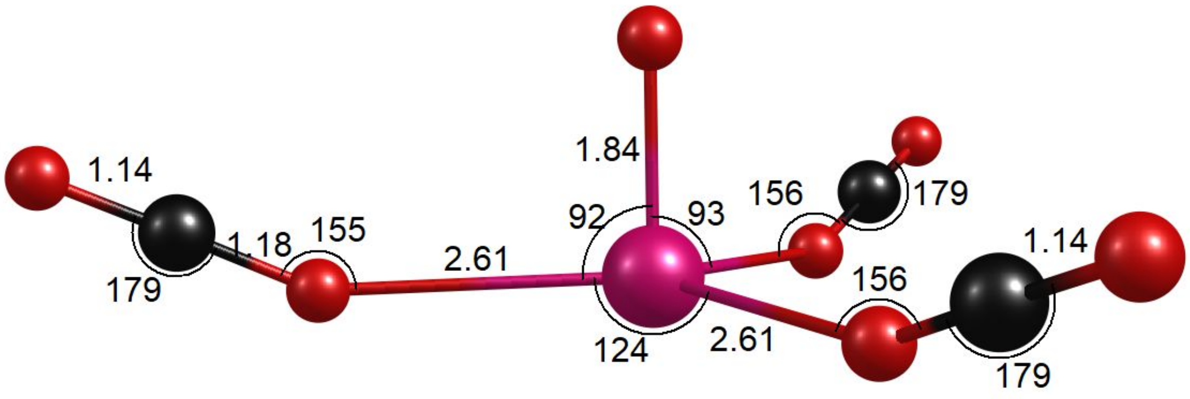

Figure S79. The optimized geometry of isomer 3c-quartet $UO^+(CO_2)_3$ followed by its predicted frequencies (cm^{-1}) and IR intensities (km/mol).

Frequency (cm^{-1})	Intensity (km/mol)	Frequency (cm^{-1})	Intensity (km/mol)
7.3801	0.6153	651.024	11.9289
13.3371	0.0715	651.141	9.2263
15.4912	0.1981	651.9045	62.7064
27.0749	7.9993	651.9659	16.5752
31.3532	5.7608	653.6081	32.3931
61.491	0.0663	654.5506	48.6773
62.7223	0.002	845.8332	324.561
68.521	0.0708	1372.2638	57.6364
85.8312	0.0244	1372.8206	56.0063
115.2678	17.6404	1374.3308	14.4328
131.4223	17.4126	2421.9965	1424.1942
154.394	0.1469	2428.495	1532.2542
160.4471	13.5178	2444.1127	235.309
163.4287	16.0781		

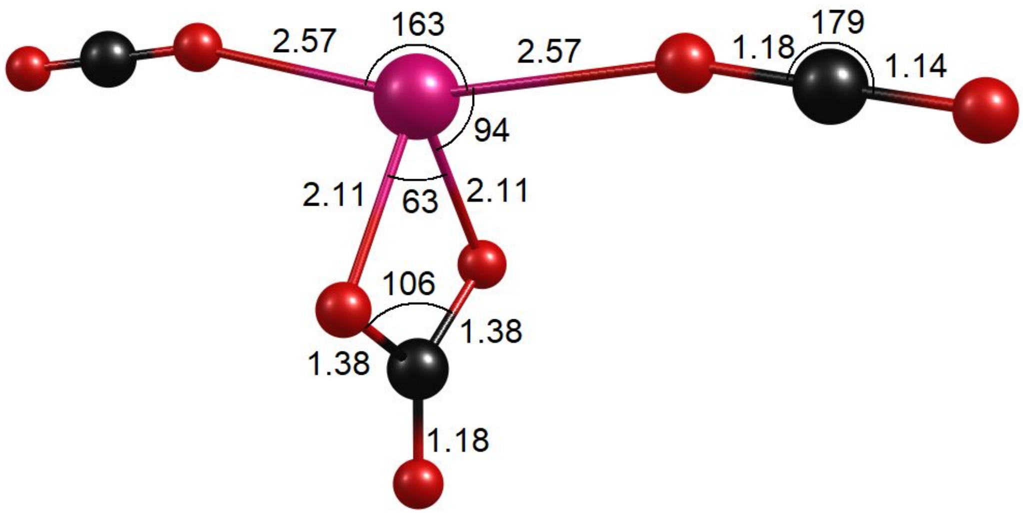

Figure S80. The optimized geometry of isomer 3d-doublet $UO^{+}(CO_2)_3$ followed by its predicted frequencies (cm^{-1}) and IR intensities (km/mol).

Frequency (cm^{-1})	Intensity (km/mol)	Frequency (cm^{-1})	Intensity (km/mol)
10.9621	0.0238	648.723	8.1746
15.0992	0.3151	648.8832	38.3329
27.6027	5.6593	649.7173	25.7756
53.7793	0.0001	649.9608	48.987
78.3851	0.2741	764.4095	167.0439
80.6357	0.0609	782.6162	21.2148
81.8501	0.8816	917.0214	171.2286
101.5566	0.0074	986.6711	193.6291
139.4158	0.0909	1370.6974	143.3207
159.1997	0.6508	1372.809	0.028
173.192	37.0321	1906.145	722.0885
317.1619	1.515	2431.2699	2139.7202
368.9354	48.7053	2442.117	1.3537
617.1374	1.6554		

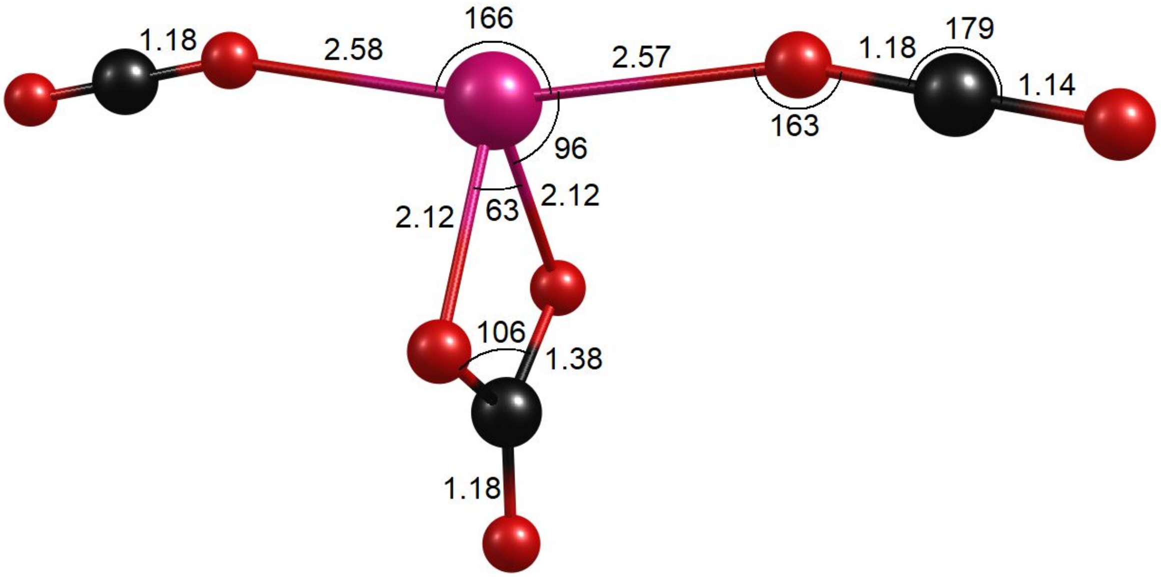

Figure S81. The optimized geometry of isomer 3d-quartet $UO^+(CO_2)_3$ followed by its predicted frequencies (cm^{-1}) and IR intensities (km/mol).

Frequency (cm^{-1})	Intensity (km/mol)	Frequency (cm^{-1})	Intensity (km/mol)
12.4299	0.0001	646.5628	29.2044
15.2408	0.3649	648.8433	25.6609
26.5106	6.1977	650.3953	34.0928
46.945	0.0246	651.26	35.6618
79.4915	0.2747	761.9666	150.148
83.2637	0.685	788.1254	21.6932
84.2463	0.0719	919.4123	169.5815
92.6963	0.001	989.4889	195.6836
139.2942	0.2455	1370.9889	133.7796
159.2487	0.7508	1373.0622	0.473
172.7693	36.8225	1902.663	694.8307
313.8497	1.9737	2431.7917	2117.5368
372.484	50.0208	2442.8047	9.0709
618.4655	1.2161		

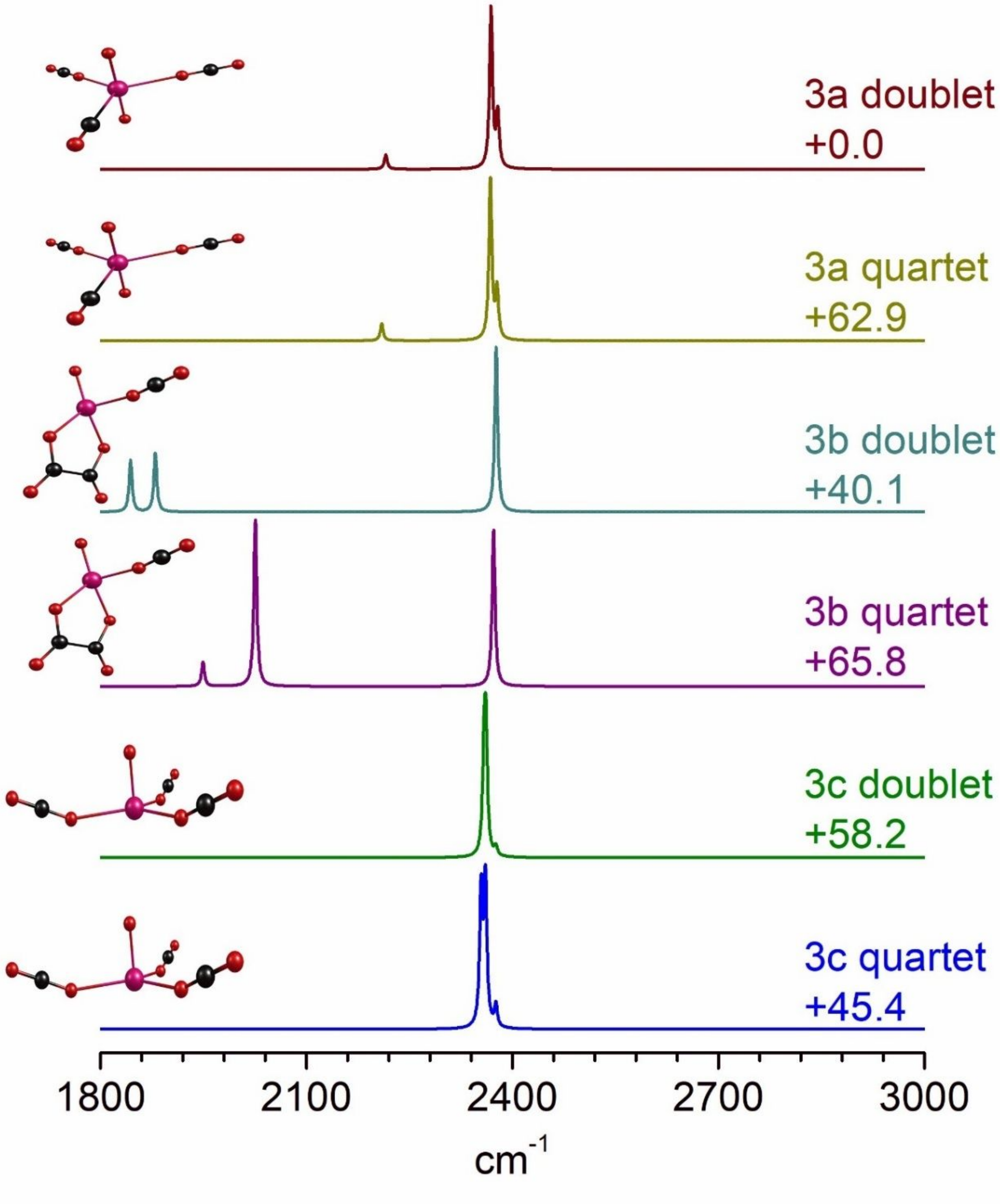

Figure S82. Simulated spectra of $UO^{+}(CO_2)_3$ for isomers 3a, 3b and 3c. Relative energies (kcal/mol) are shown next to each spectrum.

Table S80. $UO^+(CO_2)_4$ electronic energy calculated at the B3LYP/cc-pVTZ(-pp) level with Stuttgart/Koeln pseudopotential.

Isomer	2s + 1	Energy (hartree)	Rel. E (kcal/mol)	BDE (CO_2) (kcal/mol)	BDE (CO) (kcal/mol)	BDE (oxalate) (kcal/mol)
4a	2	-1304.570496	+0.0	11.0	10.8	
4a	4	-1304.470600	+62.7	11.3		
4b	2	-1304.516733	+33.7	17.4		50.9
4b	4	-1304.473222	+61.0	15.8		9.2
4c	2	-1304.475669	+59.5	9.7		
4c	4	-1304.496418	+46.5	9.9		
4d	2	-1304.466139	+65.5	12.0		
4d	4	-1304.489591	+50.8	11.6		
4e	2	-1304.389441	+113.6	-102.6		
4e	4	-1304.388560	+114.2	-40.2		

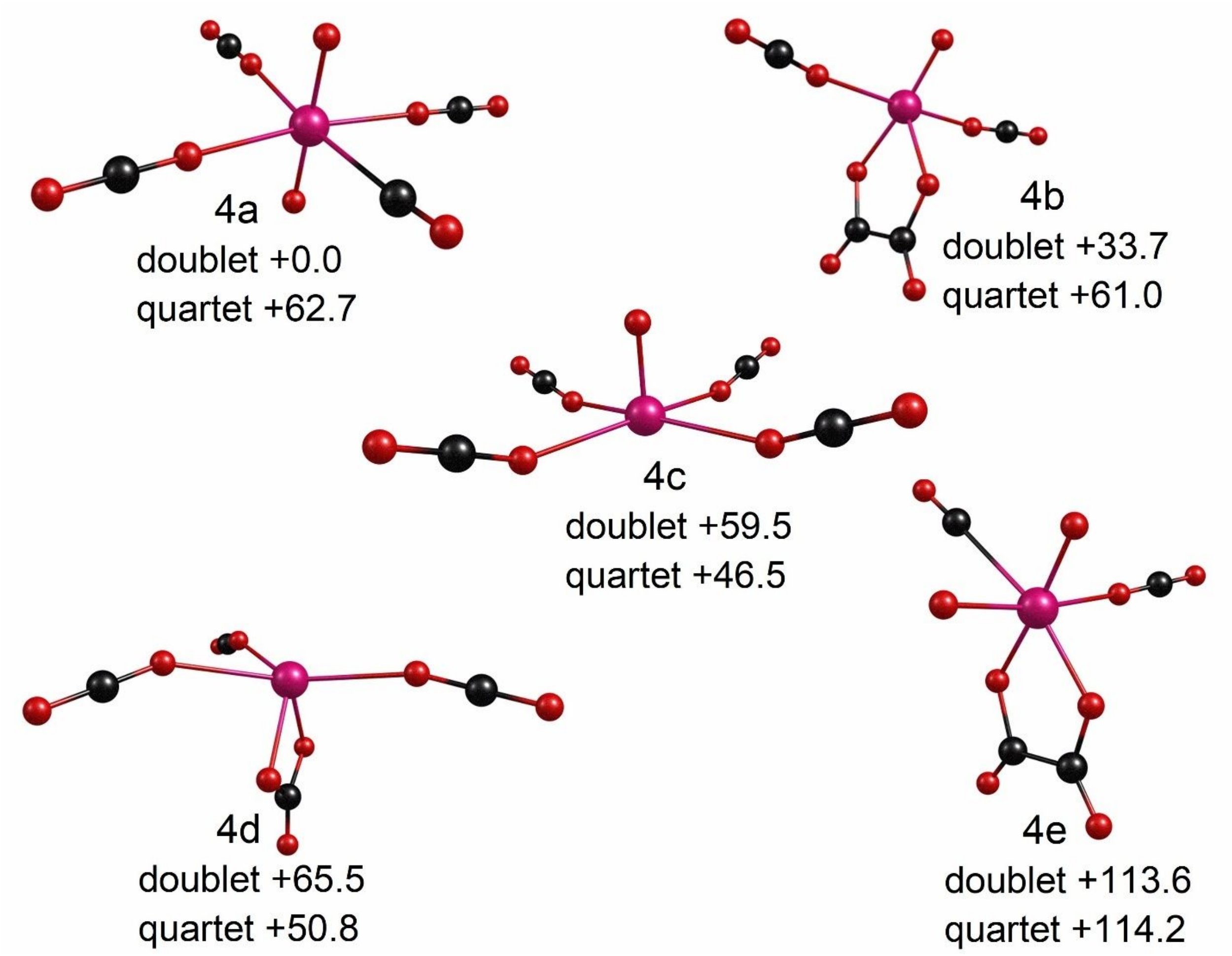

Figure S83. Predicted minimum energy structures of $UO^+(CO_2)_4$ with energy of each spin state in kcal/mol. The lowest energy spin state of each isomer is shown.

Table S81. Cartesian coordinates for the optimized geometry of isomer 4a-doublet $UO^{+}(CO_2)_4$ followed by its predicted frequencies (cm^{-1}) and IR intensities (km/mol).

Z	x	y	z
92	0.000024000	-0.050810000	0.000011000
8	2.604750000	-0.487421000	-0.000014000
6	3.767259000	-0.646704000	0.000017000
8	4.902029000	-0.801721000	0.000046000
8	-0.000164000	2.587901000	0.000094000
6	-0.000244000	3.761243000	-0.000017000
8	-0.000324000	4.906519000	-0.000121000
8	-2.604670000	-0.487615000	0.000015000
6	-3.767165000	-0.646991000	0.000025000
8	-4.901923000	-0.802100000	0.000036000
8	0.000012000	-0.059885000	-1.792086000
6	0.000042000	-2.824715000	-0.000106000
8	0.000050000	-3.943432000	-0.000149000
8	0.000040000	-0.060061000	1.792108000

Frequency	Intensity	Frequency	Intensity	Frequency	Intensity
10.8189	0.0942	130.4744	0.0062	654.6093	18.5343
14.8739	0.0681	130.6022	0	656.8842	61.9807
15.7289	0.0696	135.841	3.2274	657.2927	17.5833
17.7946	0.3081	136.3805	5.6626	871.1637	3.855
24.7619	0	148.2626	3.3843	934.2784	390.2663
27.3046	0.0013	188.3998	35.7981	1380.1685	86.7028
49.2659	0.0405	194.9804	42.3871	1380.7611	32.604
56.7661	0.0498	201.1389	28.1817	1382.0167	14.0998
61.26	0.0094	244.1274	1.8417	2266.9456	168.7012
66.6376	0.0326	648.3497	0.2468	2432.89	866.206
91.5863	0.0198	648.894	0	2433.5888	2039.8089
118.8707	0.0019	649.5953	83.9439	2449.0895	239.348

Table S82. Cartesian coordinates for the optimized geometry of isomer 4a-quartet $UO^+(CO_2)_4$ followed by its predicted frequencies (cm^{-1}) and IR intensities (km/mol).

Z	x	y	z
92	0.000033000	-0.102432000	0.018153000
8	2.616622000	-0.380139000	0.046235000
6	3.784876000	-0.480044000	0.092910000
8	4.925110000	-0.577433000	0.137618000
8	-0.000084000	2.534249000	-0.154656000
6	-0.000130000	3.707521000	-0.154108000
8	-0.000176000	4.852936000	-0.156499000
8	-2.616563000	-0.380125000	0.046267000
6	-3.784814000	-0.480087000	0.092934000
8	-4.925043000	-0.577531000	0.137635000
8	0.000013000	-0.277113000	-1.831172000
6	-0.000119000	-2.887291000	-0.111061000
8	-0.000180000	-3.999137000	-0.238037000
8	0.000065000	0.087191000	1.863342000

Frequency	Intensity	Frequency	Intensity	Frequency	Intensity
10.662	0.0602	127.0424	0.0823	649.41	74.546
13.7104	0.033	131.8074	0.1477	655.0563	10.4789
14.255	0.0728	133.6375	3.4276	656.5972	61.8828
17.5781	0.2974	136.7972	3.3823	657.8368	23.7942
22.6657	0.0005	143.8291	1.3587	718.9797	3.1491
26.882	0.0025	174.1117	29.8047	1379.919	87.2969
45.5618	0.0177	176.9484	46.4596	1380.1425	37.8882
55.6858	0.0183	193.897	9.8901	1381.5565	7.8724
61.9928	0.0146	247.8082	4.8588	2259.1887	212.9383
66.7965	0.0011	639.1036	0.0755	2431.9812	807.4094
91.6113	0.1298	647.9279	9.652	2433.9301	2068.9613
113.7909	0.0951	648.1367	0.075	2448.8454	264.9937

Table S83. Cartesian coordinates for the optimized geometry of isomer 4b-doublet $UO^{+}(CO_2)_4$ followed by its predicted frequencies (cm^{-1}) and IR intensities (km/mol).

Z	x	y	z
92	0.000005000	-0.552797000	-0.068741000
8	2.544404000	-0.610912000	-0.199089000
6	3.703136000	-0.760755000	-0.049742000
8	4.827639000	-0.902008000	0.087381000
8	-0.000001000	1.159041000	-1.198399000
6	-0.000014000	2.475344000	-0.690869000
8	-0.000014000	3.438157000	-1.358917000
8	-2.544394000	-0.610936000	-0.199092000
6	-3.703126000	-0.760789000	-0.049749000
8	-4.827627000	-0.902052000	0.087371000
8	-0.000019000	1.095471000	1.261114000
6	-0.000027000	2.401386000	0.873757000
8	-0.000040000	3.349563000	1.574442000
8	0.000014000	-2.175553000	0.673160000

Frequency	Intensity	Frequency	Intensity	Frequency	Intensity
16.7423	0.3392	160.7039	1.8406	713.9604	135.9272
19.4236	0.3284	189.005	55.7117	807.1463	0.0409
24.9016	1.4435	307.8085	17.4793	827.6387	330.2678
31.9692	0.2707	314.7127	20.4901	897.6948	19.7842
60.5676	2.1264	339.9894	0.9042	918.4997	272.7692
68.8614	5.2733	475.1448	50.6399	1113.8659	528.8561
71.7345	0.2543	538.51	1.9645	1377.5018	150.2728
98.4207	1.4396	582.3853	15.0142	1379.9109	3.9363
100.3603	10.8555	645.694	1.8239	1886.4944	333.1221
121.784	0.0372	645.8982	48.0873	1920.478	397.2376
150.3294	5.3881	648.6626	0.2172	2437.6885	2083.3141
160.6063	0.1245	649.0797	63.9221	2447.5853	76.2992

Table S84. Cartesian coordinates for the optimized geometry of isomer 4b-quartet $UO^+(CO_2)_4$ followed by its predicted frequencies (cm^{-1}) and IR intensities (km/mol).

Z	x	y	z
92	0.000076000	-0.443329000	0.185267000
8	2.433551000	-0.933839000	-0.505277000
6	3.516198000	-1.394842000	-0.547476000
8	4.569146000	-1.835164000	-0.597631000
8	-0.000064000	1.359203000	-1.317788000
6	-0.000311000	2.600987000	-1.146402000
8	-0.000452000	3.634158000	-1.689982000
8	-2.433238000	-0.934426000	-0.505392000
6	-3.515739000	-1.395774000	-0.547591000
8	-4.568549000	-1.836426000	-0.597744000
8	-0.000370000	1.597317000	1.348908000
6	-0.000543000	2.785536000	0.982829000
8	-0.000773000	3.905247000	1.305585000
8	0.000170000	-1.804715000	1.372735000

Frequency	Intensity	Frequency	Intensity	Frequency	Intensity
13.7862	0.3336	158.1895	6.2898	651.4452	6.1949
16.4155	0.8192	168.959	4.3192	652.1133	61.8851
17.9646	0.0785	171.9305	34.0709	658.8153	214.5476
25.0935	0.0583	191.5829	1.0805	894.4712	259.0161
47.4457	5.1949	218.6049	19.762	1117.0341	103.9894
60.6088	5.4163	307.4121	0.4032	1257.6458	228.8405
67.8978	0.019	335.7071	47.3219	1374.7111	111.1643
79.359	0.3645	452.9706	26.7951	1376.4465	19.6135
81.8962	3.4071	629.2443	188.6986	2000.3718	146.0571
103.3566	0.7682	633.7597	0.1815	2074.6135	1156.3444
106.6011	8.7563	647.7862	20.8143	2434.3668	1799.5808
141.6566	17.7537	647.9613	4.3357	2443.7403	323.7401

Table S85. Cartesian coordinates for the optimized geometry of isomer 4c-doublet $UO^+(CO_2)_4$ followed by its predicted frequencies (cm^{-1}) and IR intensities (km/mol).

Z	x	y	z
92	0.000026000	-0.163700000	-0.108813000
8	0.000006000	-0.352926000	1.715849000
8	-1.682876000	1.876507000	-0.253803000
6	-2.448246000	2.733515000	-0.015922000
8	-3.196536000	3.572031000	0.209259000
8	-3.923454000	-3.016717000	0.252004000
6	-3.063322000	-2.326249000	-0.056510000
8	-2.184325000	-1.619921000	-0.385376000
8	1.682768000	1.876626000	-0.253824000
6	2.448050000	2.733740000	-0.016041000
8	3.196251000	3.572359000	0.209051000
8	2.184393000	-1.619900000	-0.385312000
6	3.063407000	-2.326189000	-0.056405000
8	3.923554000	-3.016618000	0.252156000

Frequency	Intensity	Frequency	Intensity	Frequency	Intensity
6.5281	0.0009	81.9549	0.1793	650.5398	54.9383
13.7248	0.0996	115.1689	12.1449	651.6077	43.0914
16.2758	0.0555	121.9941	6.2681	652.9381	10.4856
16.4144	0.2378	130.2884	10.1993	850.0335	366.2238
22.5384	0.0291	139.5769	10.9605	1372.6179	40.5572
29.2065	3.4883	144.1665	5.9289	1372.96	44.0991
29.8199	4.5281	149.1461	15.1507	1374.1334	16.1963
50.6825	0.0345	645.799	6.9795	1375.2113	10.548
65.0471	0.0037	647.2035	9.2078	2423.5494	22.6329
65.1528	0.3497	647.3219	17.9889	2426.9826	2251.1054
72.1324	0.0108	648.9721	1.514	2427.307	1893.1174
77.4995	0.0575	649.7404	77.5902	2448.3315	181.0026

Table S86. Cartesian coordinates for the optimized geometry of isomer 4c-quartet $UO^+(CO_2)_4$ followed by its predicted frequencies (cm^{-1}) and IR intensities (km/mol).

Z	x	y	z
92	-0.000782000	0.128327000	0.204772000
8	-0.003068000	0.321510000	-1.633879000
8	2.125387000	1.701761000	0.392298000
6	2.940174000	2.412913000	-0.066249000
8	3.739492000	3.109269000	-0.499783000
8	3.215512000	-3.538350000	-0.401707000
6	2.470951000	-2.728387000	-0.080266000
8	1.710342000	-1.901455000	0.258158000
8	-2.132683000	1.693772000	0.395155000
6	-2.950312000	2.401451000	-0.063733000
8	-3.752399000	3.094413000	-0.497573000
8	-1.700076000	-1.911270000	0.258263000
6	-2.456418000	-2.741154000	-0.082531000
8	-3.196806000	-3.554031000	-0.406224000

Frequency	Intensity	Frequency	Intensity	Frequency	Intensity
6.2487	0.0034	83.2859	0.0306	654.3978	51.3227
12.6894	0.0866	123.2761	14.4875	655.88	44.6505
13.3226	0.1833	127.4934	6.5836	657.4588	13.597
16.1815	0.2073	134.499	16.0934	835.9465	331.3139
22.2533	0.0128	142.3279	14.151	1372.0389	39.2655
25.958	4.0246	149.826	6.5061	1372.5523	48.6355
29.0703	5.2783	152.3966	11.0589	1374.1801	17.6102
52.0092	0.0571	649.5831	12.7328	1375.23	14.1266
62.0517	0.6323	650.7162	7.4956	2423.2131	119.4243
64.6324	0.1398	651.4698	27.5945	2426.1856	1975.2188
73.2071	0.0004	653.4708	4.3636	2427.0829	1815.8035
78.5335	0.0685	653.6239	65.4346	2448.4098	280.3062

Table S87. Cartesian coordinates for the optimized geometry of isomer 4d-doublet $UO^{+}(CO_2)_4$ followed by its predicted frequencies (cm^{-1}) and IR intensities (km/mol).

Z	x	y	z
92	0.181246000	-0.057447000	-0.342602000
6	0.370569000	0.383484000	2.262578000
8	0.308442000	1.334923000	1.272849000
8	0.463630000	0.572377000	3.425841000
8	0.305522000	-0.841521000	1.630923000
6	-2.599661000	-2.519585000	-0.137542000
8	-1.847345000	-1.706899000	-0.536307000
8	-3.336548000	-3.309879000	0.236706000
8	5.018566000	-0.136902000	-0.559288000
6	3.883738000	-0.147819000	-0.684545000
8	2.713833000	-0.161098000	-0.826663000
8	-3.191393000	3.322225000	-0.404050000
6	-2.531324000	2.418982000	-0.640732000
8	-1.861528000	1.486119000	-0.899909000

Frequency	Intensity	Frequency	Intensity	Frequency	Intensity
9.8562	1.4864	143.6166	1.0774	655.6547	54.6926
11.5839	3.5169	152.3741	5.1037	758.4318	162.0943
17.1092	0.7222	161.0076	1.872	789.7826	20.6337
24.2035	2.8951	172.3739	35.5174	928.3762	154.3691
27.9223	2.966	311.0396	4.9242	1009.5887	200.5988
47.3309	0.0266	368.2562	51.5905	1368.8004	53.6437
60.9234	0.1813	629.0521	0.5104	1370.4512	78.5843
76.2677	0.5492	647.7942	17.3757	1373.1437	30.4669
80.7853	0.3091	650.2337	24.5438	1891.8957	728.277
81.4167	0.2604	650.9234	34.8402	2424.1975	1170.731
92.6112	0.1035	651.9812	35.8268	2431.4583	1896.6084
97.3291	3.421	654.0284	13.682	2444.6984	29.3665

Table S88. Cartesian coordinates for the optimized geometry of isomer 4d-doublet $UO^{+}(CO_2)_4$ followed by its predicted frequencies (cm^{-1}) and IR intensities (km/mol).

Z	x	y	z
92	0.159633000	0.178570000	-0.321939000
6	0.457745000	-0.759504000	2.145374000
8	0.338002000	0.564993000	1.760047000
8	0.601606000	-1.153569000	3.251115000
8	0.379545000	-1.508469000	1.002548000
6	-2.236104000	-2.514942000	-0.868250000
8	-1.745368000	-1.464622000	-1.076092000
8	-2.726355000	-3.531284000	-0.683829000
8	4.991295000	0.378998000	-0.525149000
6	3.857069000	0.397575000	-0.655764000
8	2.688132000	0.421022000	-0.803696000
8	-3.743786000	2.981579000	0.514310000
6	-2.888368000	2.332427000	0.121223000
8	-2.011611000	1.666125000	-0.293895000

Frequency	Intensity	Frequency	Intensity	Frequency	Intensity
10.0113	0.8979	141.3506	3.3733	656.3577	48.5388
13.5793	1.9988	150.5318	3.7454	755.1308	141.5989
19.1134	2.5805	168.279	14.2542	792.048	20.5827
24.1253	1.5996	174.4491	24.4223	924.3297	160.1159
27.6681	4.4456	313.3902	3.1908	1016.5077	216.2719
44.691	0.2167	367.5244	53.7005	1366.9011	51.235
61.8341	0.2917	630.7473	1.7399	1371.3981	90.0835
76.5481	0.5983	648.7492	20.3409	1373.2328	20.5232
80.5975	0.0936	651.2628	27.8573	1889.6247	704.2876
84.135	0.4407	651.6852	23.4617	2423.652	1112.0367
90.9385	0.0621	653.0827	32.7011	2431.5296	1882.5561
102.1124	3.1806	655.2994	34.8214	2444.1788	26.4826

Table S89. Cartesian coordinates for the optimized geometry of isomer 4e-doublet $UO^+(CO_2)_4$ followed by its predicted frequencies (cm^{-1}) and IR intensities (km/mol).

Z	x	y	z
92	0.309255000	-0.309759000	-0.345391000
8	0.768107000	2.118316000	0.410418000
6	1.015126000	3.271350000	0.423949000
8	1.253114000	4.387149000	0.447723000
8	-1.624516000	0.302748000	-0.986649000
6	-2.744511000	0.221196000	-0.237879000
8	-3.843218000	0.529989000	-0.529367000
8	0.265142000	-2.304246000	-0.759508000
6	2.454816000	-1.222957000	1.217346000
8	3.306676000	-1.674134000	1.780017000
8	-0.927977000	-0.594286000	1.179582000
6	-2.378537000	-0.356062000	1.172922000
8	-3.048156000	-0.580822000	2.094936000
8	1.534230000	-0.057632000	-1.597405000

Frequency	Intensity	Frequency	Intensity	Frequency	Intensity
21.0314	0.3731	181.4418	6.899	646.6859	39.6083
25.5284	0.0276	194.2822	14.1461	650.2726	32.0462
33.3179	0.4897	226.8645	0.5102	788.5579	14.6865
35.1563	0.5337	231.131	3.2018	791.926	201.3373
61.5564	0.9088	283.9711	17.2702	881.8736	12.7656
73.5448	0.5967	315.6211	20.0053	921.1466	218.3834
85.7448	1.3431	340.733	2.5652	1091.2375	445.086
109.7125	2.9729	460.1869	33.1194	1372.7419	83.2647
119.4513	8.5399	484.5806	84.4807	1887.1971	401.2339
137.6415	3.124	499.3732	58.3617	1971.4205	331.2087
157.8255	8.9329	562.7561	28.6425	2299.8318	53.5057
172.7815	7.7734	623.2422	191.6865	2437.4949	1046.5149

Table S90. Cartesian coordinates for the optimized geometry of isomer 4e-quartet $UO^{+}(CO_2)_4$ followed by its predicted frequencies (cm^{-1}) and IR intensities (km/mol).

Z	x	y	z
92	-0.037376000	-0.130301000	-0.372310000
8	1.135244000	2.004693000	0.673297000
6	1.603114000	3.068025000	0.861189000
8	2.059040000	4.098867000	1.051677000
8	-2.440270000	0.461338000	0.279823000
6	-3.616312000	0.504797000	0.244609000
8	-4.757148000	0.554729000	0.221451000
8	-0.583349000	-1.910531000	-1.252124000
6	2.459051000	-1.093400000	-1.063948000
8	3.410545000	-1.563531000	-1.410779000
8	0.191954000	-1.142458000	1.438491000
6	0.226015000	-1.713553000	2.648867000
8	1.049016000	-2.358228000	3.171547000
8	-0.139106000	0.779181000	-1.909853000

Frequency	Intensity	Frequency	Intensity	Frequency	Intensity
14.5744	0.1075	117.4943	0.9427	650.3242	26.6228
18.2695	0.1843	140.8357	6.7178	651.9004	26.7458
20.0896	0.1892	143.9909	9.1807	655.5351	38.4521
22.5287	0.5632	163.4586	7.822	670.4704	33.1997
27.0685	0.5205	167.9774	8.4783	871.4995	50.3666
38.9695	2.165	192.7683	28.8112	892.1008	392.7072
58.5717	4.0557	194.8221	21.766	1373.8592	73.3702
59.2638	0.0978	237.8999	1.8104	1377.4376	65.4911
67.1859	1.0635	240.0703	1.1117	1896.685	596.3711
83.9221	0.2001	303.0112	13.8215	2296.1297	65.732
99.4565	3.5044	511.0825	48.7733	2431.8048	1333.5601
112.1441	2.0881	646.5164	21.7852	2443.5516	763.3381

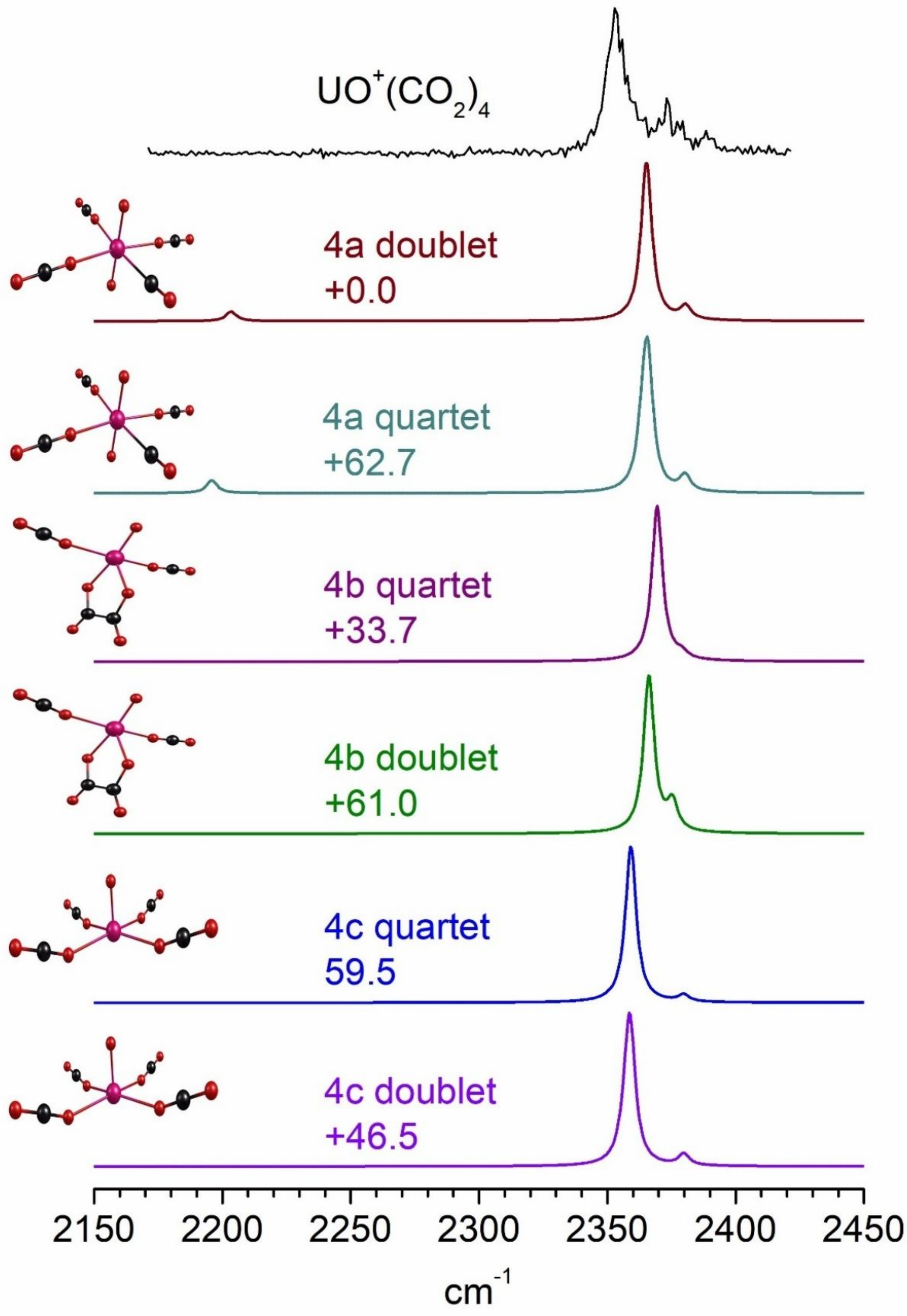

Figure S84. Experimental IR spectrum of $UO^{+}(CO_2)_4$ compared with simulated spectra for isomers 4a, 4b, and 4c. Relative energies (kcal/mol) are shown next to each spectrum.

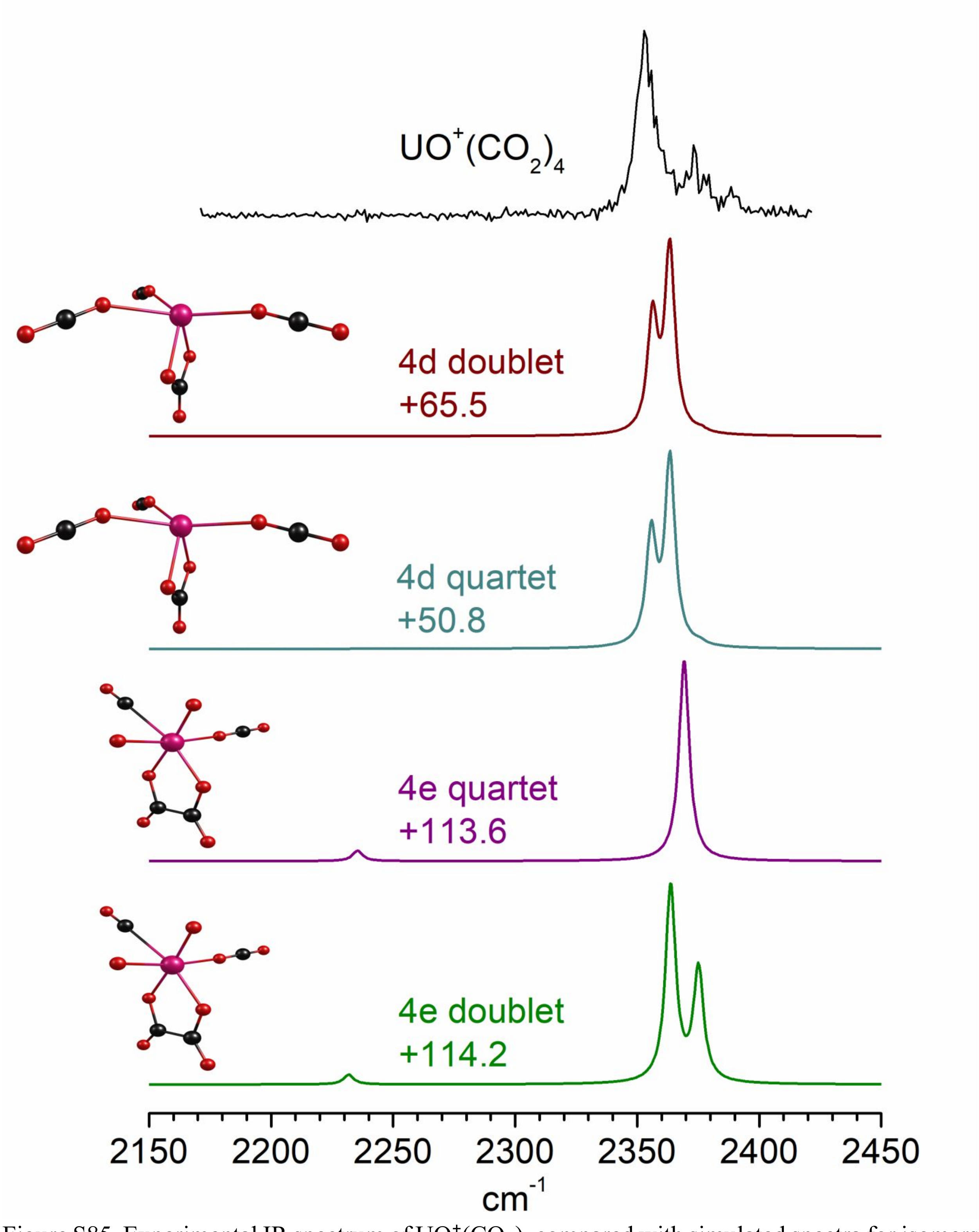

Figure S85. Experimental IR spectrum of $UO^{+}(CO_2)_4$ compared with simulated spectra for isomers 4d and 4e. Relative energies (kcal/mol) are shown next to each spectrum.

Table S91. $UO^+(CO_2)_5$ electronic energy calculated at the B3LYP/cc-pVTZ(-pp) level with Stuttgart/Koeln pseudopotential.

Isomer	2s + 1	Energy (hartree)	Rel. E (kcal/mol)	BDE (CO_2) (kcal/mol)	BDE (CO) (kcal/mol)	BDE (oxalate) (kcal/mol)
5a	2	-1493.231085	+0.0	7.3	7.1	
5a	4	-1493.131165	+62.7	7.3		
5b	2	-1493.185978	+28.3	12.8		48.3
5b	4	-1493.141057	+56.5	11.9		7.3
5c	2	-1493.136778	+59.2	7.7		
5c	4	-1493.157574	+46.1	7.7		
5d	4	-1493.154929	+47.8	10.3		
5e	2	-1493.053908	+112.2	9.8		
5e	4	-1493.050145	+113.5	8.0		

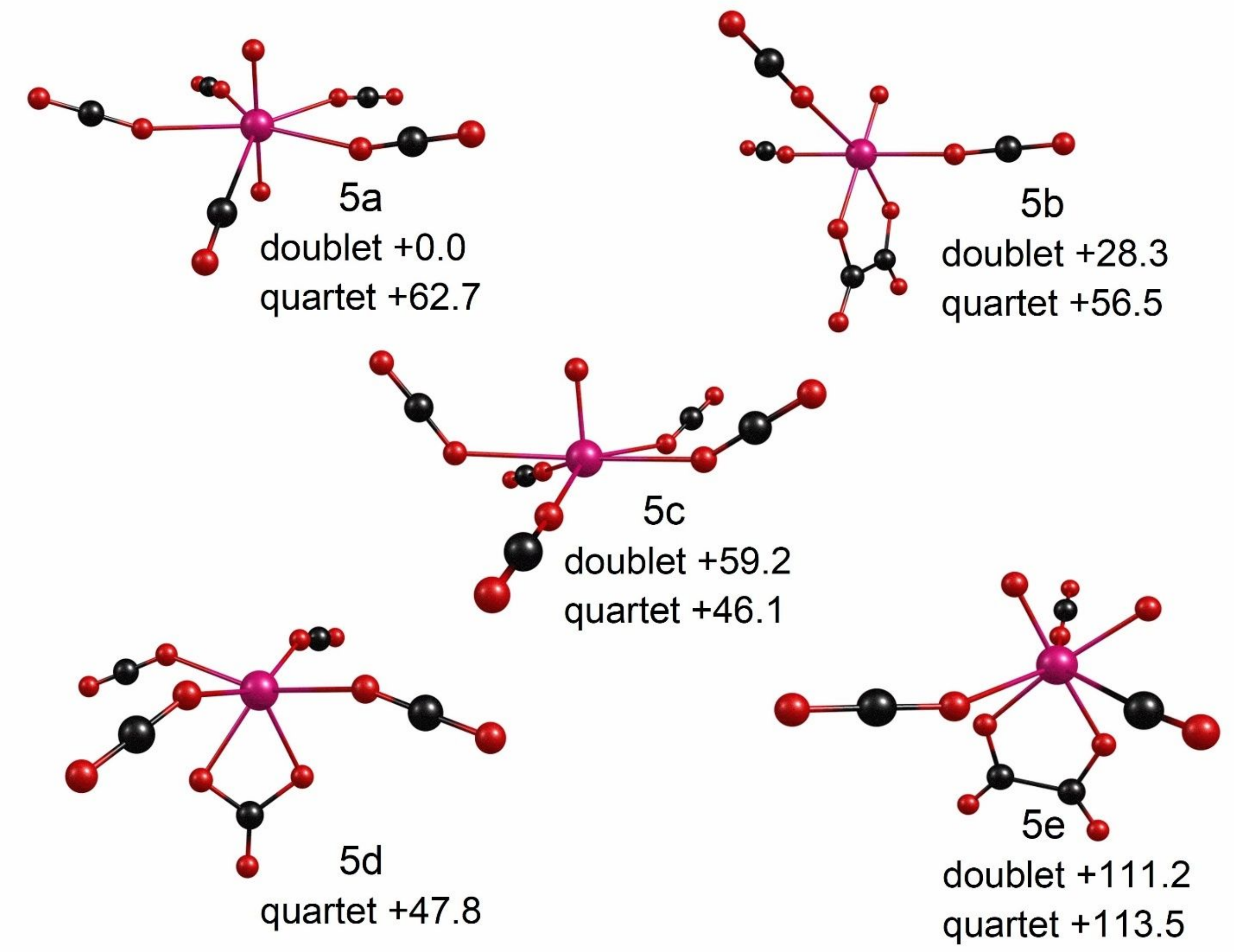

Figure S86. Predicted minimum energy structures of $UO^+(CO_2)_5$ with energy of each spin state in kcal/mol. The lowest energy spin state of each isomer is shown.

Table S92. Cartesian coordinates for the optimized geometry of isomer 5a-doublet $UO^+(CO_2)_5$ followed by its predicted frequencies (cm^{-1}) and IR intensities (km/mol).

Z	x	y	z
92	-0.000024000	-0.144225000	0.000010000
8	-2.528822000	-1.039642000	0.092105000
6	-3.605287000	-1.353366000	0.432723000
8	-4.661400000	-1.660395000	0.757866000
8	-1.622062000	1.996240000	-0.184515000
6	-2.232591000	2.845602000	-0.714260000
8	-2.832919000	3.680540000	-1.221483000
8	2.528816000	-1.039569000	-0.092130000
6	3.605220000	-1.353344000	-0.432895000
8	4.661275000	-1.660423000	-0.758180000
8	-0.010806000	-0.152643000	1.796057000
6	0.000062000	-2.918860000	0.000107000
8	0.000081000	-4.038286000	0.000149000
8	1.622113000	1.996168000	0.184481000
6	2.232734000	2.845419000	0.714300000
8	2.833153000	3.680247000	1.221593000
8	0.010745000	-0.152739000	-1.796037000

Frequency	Intensity	Frequency	Intensity	Frequency	Intensity
8.0785	0.0043	108.1103	0.2262	656.2023	64.5364
9.4133	0.0596	116.3714	1.3689	656.3794	82.474
20.2019	0.2381	126.3977	1.9574	659.3917	46.2414
21.9564	0.045	136.0569	0.9702	661.9273	5.3426
22.7412	0.0008	142.0275	4.8454	864.0437	3.2262
27.4014	0.0047	145.3036	6.8949	926.5681	386.1481
30.1626	0.0743	151.0906	0.9034	1376.9251	22.1919
34.0024	0.0138	198.7388	60.2641	1377.6659	46.655
46.894	0.3509	202.1326	47.7395	1379.6854	62.7222
47.0341	0.067	236.3688	2.9253	1380.6882	0.0042
70.133	0.075	240.9202	1.9108	2260.8845	161.4846
74.8768	0.0018	652.6268	0.1533	2423.3415	412.8789
83.1994	0.4963	652.6824	24.0768	2427.9508	1055.6075
86.89	0.0091	654.2459	5.9134	2432.1207	2416.9086
98.5278	0.3138	654.544	1.4241	2451.327	202.3216

Table S93. Cartesian coordinates for the optimized geometry of isomer 5a-quartet $UO^{+}(CO_2)_5$ followed by its predicted frequencies (cm^{-1}) and IR intensities (km/mol).

Z	x	y	z
92	-0.005445000	-0.157013000	0.017889000
8	-2.544825000	-0.992172000	0.214509000
6	-3.549016000	-1.287697000	0.743171000
8	-4.535966000	-1.577870000	1.249451000
8	-1.594721000	1.999542000	-0.204554000
6	-2.178971000	2.787431000	-0.847111000
8	-2.754964000	3.564642000	-1.462921000
8	2.514480000	-1.052585000	-0.057052000
6	3.628727000	-1.398961000	-0.163616000
8	4.719688000	-1.737468000	-0.265170000
8	0.007234000	-0.188537000	1.876413000
6	-0.075179000	-2.897583000	-0.492456000
8	-0.108494000	-3.975455000	-0.794275000
8	1.578373000	2.006544000	0.169511000
6	2.188864000	2.905928000	0.607753000
8	2.789118000	3.788455000	1.027490000
8	-0.018125000	-0.111283000	-1.844937000

Frequency	Intensity	Frequency	Intensity	Frequency	Intensity
5.9107	0.01	105.9967	0.0676	654.361	18.0823
11.0648	0.059	116.7373	1.205	656.611	58.8093
20.2557	0.1796	121.292	1.6191	657.6782	52.6417
22.153	0.0372	133.0486	1.5183	659.5611	50.1384
23.593	0.0155	139.5382	2.5129	661.483	3.1606
27.2292	0.0045	148.5019	5.9564	712.9024	3.0101
28.9564	0.0918	150.9381	5.8029	1375.7931	27.1095
33.5925	0.0039	182.134	30.8485	1377.1288	49.0405
45.0613	0.0612	185.6779	53.0354	1378.7524	29.0968
48.0333	0.0853	225.9985	0.5023	1381.4165	25.7222
70.5159	0.1009	248.6301	5.7585	2252.8351	220.8338
74.568	0.0147	633.1172	0.3805	2422.6511	501.3305
84.9989	0.2045	650.4908	11.1922	2427.7259	1045.8747
86.9117	0.067	653.4676	12.4842	2432.5472	2225.9924
99.0553	0.3903	653.8617	23.9969	2451.0227	259.6882

Table S94. Cartesian coordinates for the optimized geometry of isomer 5b-doublet $UO^+(CO_2)_5$ followed by its predicted frequencies (cm^{-1}) and IR intensities (km/mol).

Z	x	y	z
92	-0.000053000	-0.170027000	-0.325064000
8	2.561035000	-0.152810000	-0.332758000
6	3.675771000	-0.028670000	-0.690145000
8	4.761358000	0.087709000	-1.025839000
8	0.001066000	1.941199000	-0.619436000
6	0.001438000	2.844274000	0.392163000
8	0.002049000	4.021837000	0.294835000
8	-2.561117000	-0.150305000	-0.333072000
6	-3.675758000	-0.025067000	-0.690371000
8	-4.761255000	0.092380000	-1.025982000
8	0.000235000	0.709282000	1.528672000
6	0.000933000	2.095422000	1.767677000
8	0.001092000	2.575998000	2.838652000
8	-0.001382000	-2.530679000	0.864353000
6	-0.001952000	-3.593945000	1.365747000
8	-0.002506000	-4.628014000	1.854366000
8	-0.000293000	-0.980297000	-1.914351000

Frequency	Intensity	Frequency	Intensity	Frequency	Intensity
14.6846	0.5993	150.2919	4.2224	655.2455	40.1863
16.0595	0.0432	161.4349	1.5916	726.7257	136.3929
17.8641	0.095	163.8058	0.0024	811.6107	0.0311
19.7293	0.3284	187.2145	54.0201	828.727	373.3264
24.2862	1.6922	307.1011	14.7129	903.5898	119.8804
29.8289	0.8102	307.9059	29.7036	914.9305	136.0532
56.5755	1.5174	336.1372	4.2846	1131.4367	563.8318
63.9294	1.0414	475.609	49.4127	1376.9089	133.9893
73.1047	1.4691	540.4693	1.8894	1377.3493	57.3174
73.5339	0.0213	577.4299	26.8626	1379.6308	11.1219
83.8156	0.3439	646.9342	15.3737	1874.7054	353.588
97.7938	0.0129	647.0818	2.6111	1910.9609	417.8271
121.9242	2.6416	648.8732	58.0522	2432.4009	1007.2374
132.994	0.2576	651.1814	0.0353	2436.6132	1926.0104
148.2632	29.9288	652.1189	57.0056	2450.1854	228.9404

Table S95. Cartesian coordinates for the optimized geometry of isomer 5b-quartet $UO^+(CO_2)_5$ followed by its predicted frequencies (cm^{-1}) and IR intensities (km/mol).

Z	x	y	z
92	-0.248608000	-0.000106000	-0.239956000
8	-0.068170000	-2.595408000	-0.358410000
6	-0.081307000	-3.652305000	-0.874392000
8	-0.092320000	-4.686298000	-1.362781000
8	2.091997000	0.000254000	-0.735877000
6	3.098852000	0.000408000	-0.019440000
8	4.263890000	0.000561000	0.046270000
8	-0.068739000	2.595217000	-0.358792000
6	-0.081945000	3.652117000	-0.874762000
8	-0.093029000	4.686115000	-1.363141000
8	1.014530000	0.000164000	1.740815000
6	2.251889000	0.000350000	1.952886000
8	3.060078000	0.000496000	2.798020000
8	-2.522859000	0.000037000	1.118036000
6	-3.645272000	0.000041000	1.466513000
8	-4.735658000	0.000047000	1.813450000
8	-1.147387000	-0.000422000	-1.816203000

Frequency	Intensity	Frequency	Intensity	Frequency	Intensity
9.3825	0.1159	143.5472	18.2336	653.797	0.2513
13.3229	0.1779	144.9632	34.9346	655.0278	75.105
16.6512	0.2149	155.1162	6.4856	657.4792	35.7359
17.9589	0.0811	161.7282	5.843	670.3465	160.0278
19.6879	0.3153	165.7005	23.5208	882.6133	250.3859
27.6255	0.1284	195.7117	2.4479	1128.1675	116.4924
42.3981	5.5631	209.8401	21.357	1268.1057	221.6125
57.6232	3.1097	305.2622	2.31	1373.9883	98.126
60.1896	0.1107	327.0394	51.5677	1375.0671	34.906
65.0114	0.1137	446.2448	26.7817	1376.7167	30.5147
75.7104	0.0816	620.2951	220.634	1990.0005	239.0141
88.7037	0.4152	634.1582	0.8135	2079.5887	1147.0617
91.0153	0.0178	650.9029	5.3849	2429.2367	945.5097
105.267	0.0193	651.9706	10.7385	2430.8643	1810.2388
130.5567	6.5937	652.3824	47.8733	2445.8663	404.147

Table S96. Cartesian coordinates for the optimized geometry of isomer 5c-quartet $UO^{+}(CO_2)_5$ followed by its predicted frequencies (cm^{-1}) and IR intensities (km/mol).

Z	x	y	z
92	0.000758000	0.003464000	0.140556000
8	-0.008886000	0.059872000	-1.715597000
8	-1.577501000	2.170804000	0.237221000
6	-2.199803000	3.002468000	-0.307462000
8	-2.811753000	3.821762000	-0.826990000
8	3.031964000	3.735929000	-0.644507000
6	2.365457000	2.917513000	-0.195291000
8	1.687180000	2.085580000	0.276781000
8	2.461434000	-0.964375000	0.666255000
6	3.502126000	-1.459375000	0.877499000
8	4.521805000	-1.942883000	1.085527000
8	-0.080159000	-2.645337000	-0.316980000
6	-0.105906000	-3.237324000	-1.331893000
8	-0.132563000	-3.837851000	-2.307785000
8	-2.500429000	-0.825705000	0.712555000
8	-4.600400000	-1.673882000	1.211671000
6	-3.561087000	-1.254943000	0.964422000

Frequency	Intensity	Frequency	Intensity	Frequency	Intensity
1.4393	0.0328	96.2949	0.7416	655.6925	77.4688
10.6006	0.0063	98.6501	0.428	656.2676	51.8059
17.8215	0.161	109.9671	0.88	658.0739	2.1886
20.2249	0.3179	112.3665	0.4047	660.76	23.9191
23.4557	0.002	133.4322	4.8134	827.1507	333.9989
26.5861	0.1514	142.2875	27.2322	1365.248	24.6035
27.3604	0.2884	143.4365	41.5379	1372.7966	16.5784
30.9538	5.3586	145.6317	1.6021	1373.8526	31.086
34.2441	2.4369	179.8925	0.3368	1376.5441	33.9934
40.6327	0.3741	649.1084	0.1408	1377.1504	2.899
64.0395	0.0237	650.7037	3.8999	2416.7452	696.2715
65.6096	0.0534	650.8308	38.6897	2419.6987	57.7234
74.8126	0.1113	651.5731	26.1689	2426.6684	1805.3532
76.8296	0.3522	653.47	15.5642	2430.5375	2328.5912
86.283	0.1315	654.3865	45.4594	2452.1771	98.2493

Table S97. Cartesian coordinates for the optimized geometry of isomer 5d-quartet $UO^{+}(CO_2)_5$ followed by its predicted frequencies (cm^{-1}) and IR intensities (km/mol).

Z	x	y	z
92	0.006909000	-0.148141000	0.271721000
6	-0.037864000	0.403218000	-2.342630000
8	-0.066251000	1.320913000	-1.334149000
8	0.018529000	-0.844201000	-1.748986000
8	-0.057381000	0.617769000	-3.507744000
6	3.086194000	-2.332990000	0.376085000
8	2.182909000	-1.627042000	0.637248000
8	3.967501000	-3.021206000	0.132572000
8	-3.855426000	-3.109957000	-0.029815000
6	-3.007304000	-2.400663000	0.265235000
8	-2.138803000	-1.673237000	0.580406000
8	-3.255540000	3.299465000	0.429932000
6	-2.624885000	2.384160000	0.702455000
8	-1.986346000	1.442521000	1.001373000
8	3.138367000	3.403382000	0.198865000
6	2.565045000	2.467287000	0.522044000
8	1.987102000	1.504462000	0.873114000

Frequency	Intensity	Frequency	Intensity	Frequency	Intensity
1.3832	0.2687	102.8766	3.2844	657.1913	32.7581
9.8213	2.2019	138.1494	2.0138	657.7517	59.3788
14.6054	1.0566	142.5958	9.6654	750.6116	132.7212
21.0109	2.7361	160.2801	12.6487	796.9056	19.5963
22.9066	4.8991	164.4559	10.8835	930.8169	152.7609
27.1731	0.3755	167.4036	17.7601	1034.9102	246.8718
27.3459	0.0567	309.4591	4.2754	1368.1556	28.7795
31.667	0.9684	365.3405	53.9762	1368.6324	66.0933
54.6537	0.0039	638.1544	1.1532	1371.7747	52.9725
60.6394	0.5377	648.5341	7.6032	1372.8972	13.1457
75.1087	1.069	650.3419	35.532	1879.0017	710.8731
81.8906	0.2658	653.2297	23.823	2420.871	61.9804
91.1561	0.0547	654.9658	41.6943	2424.2775	2035.7154
92.1149	0.1863	655.1552	41.305	2429.6696	1809.1099
95.0626	1.319	656.1186	7.2963	2447.4801	45.873

Table S98. Cartesian coordinates for the optimized geometry of isomer 5e-doublet $UO^{+}(CO_2)_5$ followed by its predicted frequencies (cm^{-1}) and IR intensities (km/mol).

Z	x	y	z
92	-0.070894000	-0.469545000	0.077131000
8	2.470212000	-0.080924000	-0.825287000
6	3.219315000	0.013408000	-1.725931000
8	3.959059000	0.107063000	-2.594565000
8	-0.027029000	1.371128000	-0.998468000
6	-0.223428000	2.581781000	-0.419344000
8	-0.217861000	3.633517000	-0.952380000
8	-2.526449000	-0.318624000	-0.570612000
6	-3.595960000	-0.707627000	-0.870343000
8	-4.639031000	-1.070350000	-1.162418000
8	-0.421948000	0.998560000	1.410850000
6	-0.469762000	2.400097000	1.115272000
8	-0.664067000	3.218616000	1.924448000
8	-0.714128000	-1.950741000	1.296703000
6	2.014976000	-0.961265000	1.916863000
8	2.778220000	-1.223050000	2.689607000
8	0.109449000	-1.780226000	-1.117276000

Frequency	Intensity	Frequency	Intensity	Frequency	Intensity
18.5	0.3191	171.5343	5.4832	652.0662	14.5454
19.8483	0.0296	178.2997	1.1268	659.2324	29.0855
24.8783	0.2105	186.9025	14.8426	672.0277	220.1307
32.128	0.6764	207.2715	24.3012	788.5375	230.0634
37.1144	0.3331	232.7305	0.6738	796.99	0.108
42.4742	0.4463	275.3297	8.1922	881.6744	89.1036
61.5293	0.8314	285.3459	4.5011	889.7278	117.1847
64.4201	1.3732	308.9103	14.7511	1084.7903	361.0749
77.4618	1.0339	335.1781	6.4102	1372.3284	54.7163
95.1087	5.1113	436.9027	30.7274	1379.182	66.7605
104.3814	10.2017	466.8571	51.6515	1878.8882	332.6665
122.7672	0.4664	530.0252	3.5752	1939.9935	372.2224
136.8734	8.4206	573.1018	3.9388	2289.7926	63.0502
153.8196	8.5608	646.2408	27.185	2429.3717	1328.8863
155.6669	3.2954	649.8639	14.1573	2442.8024	773.5782

Table S99. Cartesian coordinates for the optimized geometry of isomer 5e-quartet $UO^+(CO_2)_5$ followed by its predicted frequencies (cm^{-1}) and IR intensities (km/mol).

Z	x	y	z
92	-0.029081000	-0.311517000	-0.300538000
8	2.291270000	1.021747000	-0.071310000
6	3.228615000	1.699504000	-0.274544000
8	4.142741000	2.362096000	-0.464482000
8	-0.505606000	2.291484000	0.216862000
6	-0.984787000	3.338956000	0.437119000
8	-1.453151000	4.362780000	0.652403000
8	-2.595671000	0.195321000	-0.166706000
6	-3.633849000	-0.315327000	-0.382588000
8	-4.653042000	-0.793171000	-0.584409000
8	-0.015675000	-0.712686000	1.751684000
6	-0.063259000	-0.866655000	3.079475000
8	0.645013000	-1.448631000	3.806161000
8	-0.849330000	-2.193013000	-0.628665000
6	2.075242000	-2.078366000	-0.563182000
8	2.836194000	-2.881135000	-0.722540000
8	0.025224000	0.044065000	-2.055018000

Frequency	Intensity	Frequency	Intensity	Frequency	Intensity
10.6541	0.0455	115.6961	0.2997	653.4573	55.357
15.1723	0.4734	118.8104	1.3312	654.2788	35.3694
20.7188	0.2891	134.4592	7.8689	658.3683	45.5787
24.5674	0.0472	138.1947	4.657	660.8136	27.9307
25.0125	0.2035	149.3315	15.4297	678.6874	32.7573
26.4382	0.5507	168.4285	7.6697	882.4659	70.2416
34.0096	0.4834	187.5339	2.0077	903.5718	412.1185
37.383	1.6224	188.3517	45.5321	1370.0973	55.7523
53.935	0.3773	197.3263	16.9806	1376.6526	40.1361
60.6646	0.4061	233.2969	2.3147	1379.3254	46.6535
74.2757	0.1348	283.6959	2.7695	1892.1169	587.7556
83.6289	0.2098	309.4632	18.1453	2286.2038	79.1802
90.3745	2.4363	503.0976	54.378	2424.3989	468.4304
104.9973	2.9042	650.7517	0.4172	2429.0555	1829.1933
108.0701	0.8031	652.3314	5.6916	2446.2149	693.0843

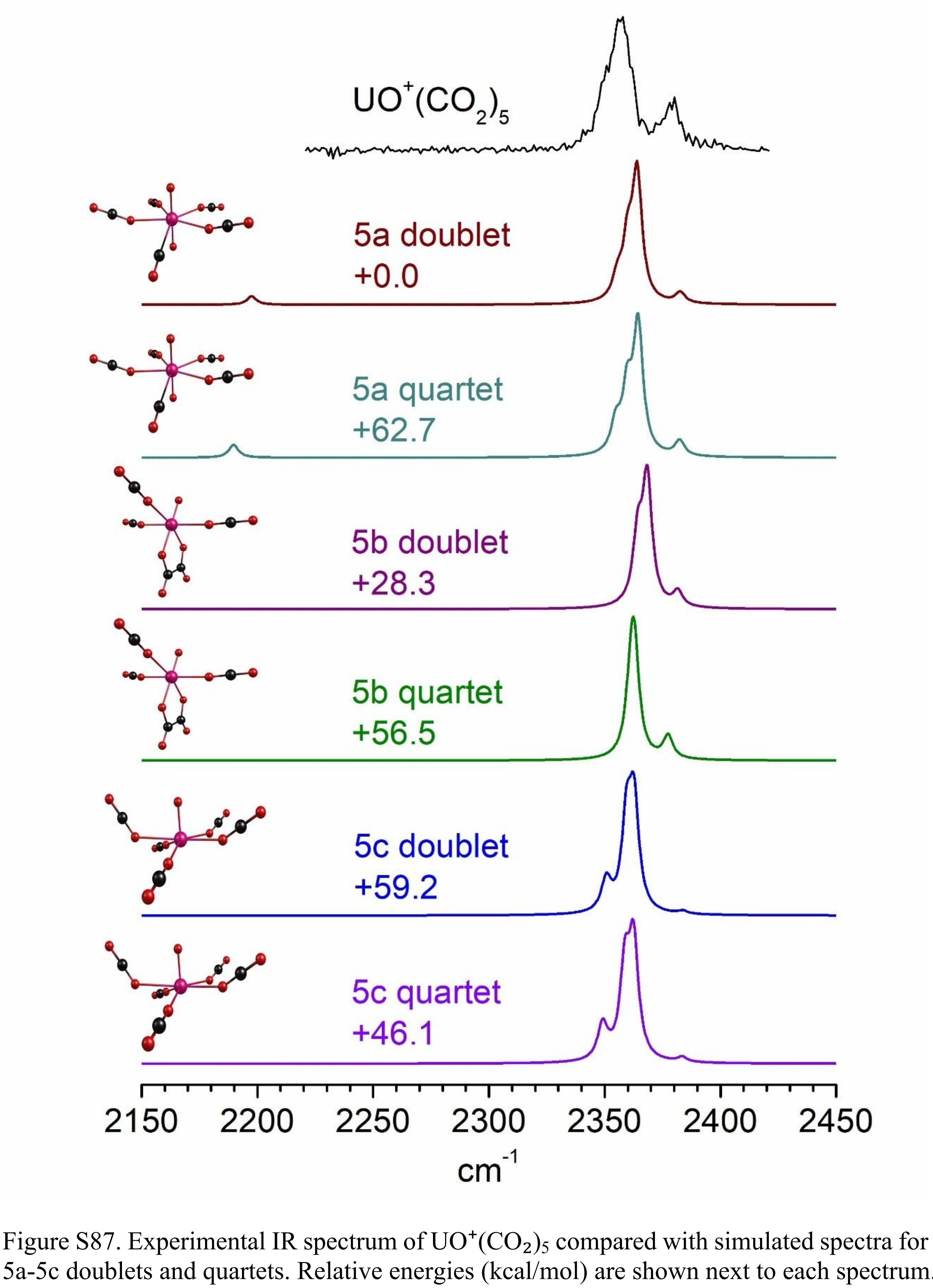

Figure S87. Experimental IR spectrum of $UO^+(CO_2)_5$ compared with simulated spectra for isomer 5a-5c doublets and quartets. Relative energies (kcal/mol) are shown next to each spectrum.

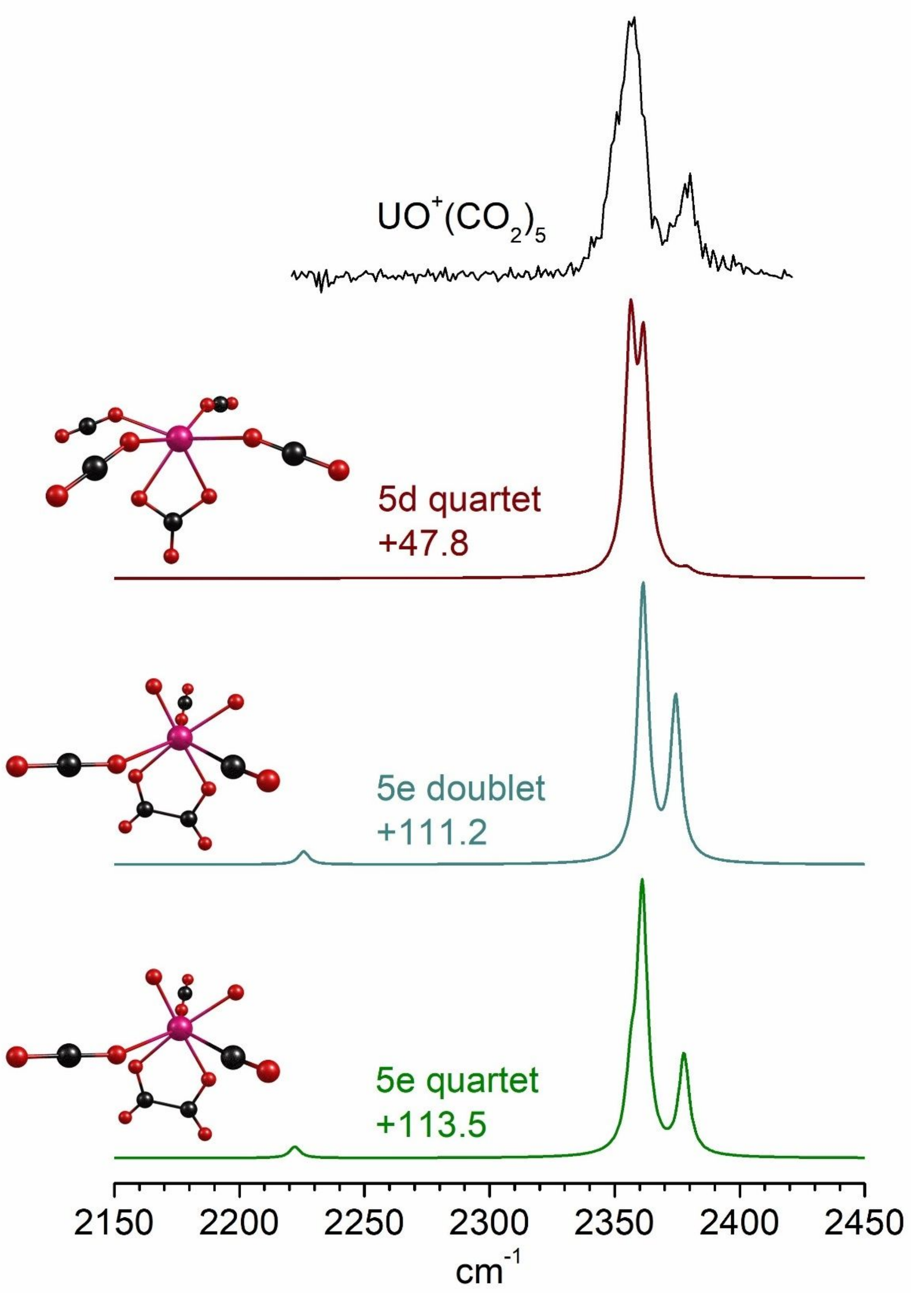

Figure S88. Experimental IR spectrum of $UO^{+}(CO_2)_5$ compared with simulated spectra for 5d and 5e isomers. Relative energies (kcal/mol) are shown next to each spectrum.

Table S100. $UO^+(CO_2)_6$ electronic energy calculated at the B3LYP/cc-pVTZ(-pp) level with Stuttgart/Koeln pseudopotential.

Isomer	2s + 1	Energy (hartree)	Rel. E (kcal/mol)	BDE (CO_2) (kcal/mol)	BDE (CO) (kcal/mol)	BDE (oxalate) (kcal/mol)
6a	2	-1681.882434	+0.0	1.6		
6b	2	-1681.847181	+22.1	7.7		46.3
6b	4	-1681.803754	+49.4	8.7		6.0
6c	2	-1681.791834	+56.9			
6d	2	-1681.790883	+57.4	3.3		
6d	4	-1681.812450	+43.9	3.8		
6e	2	-1681.707849	+109.6	3.2		

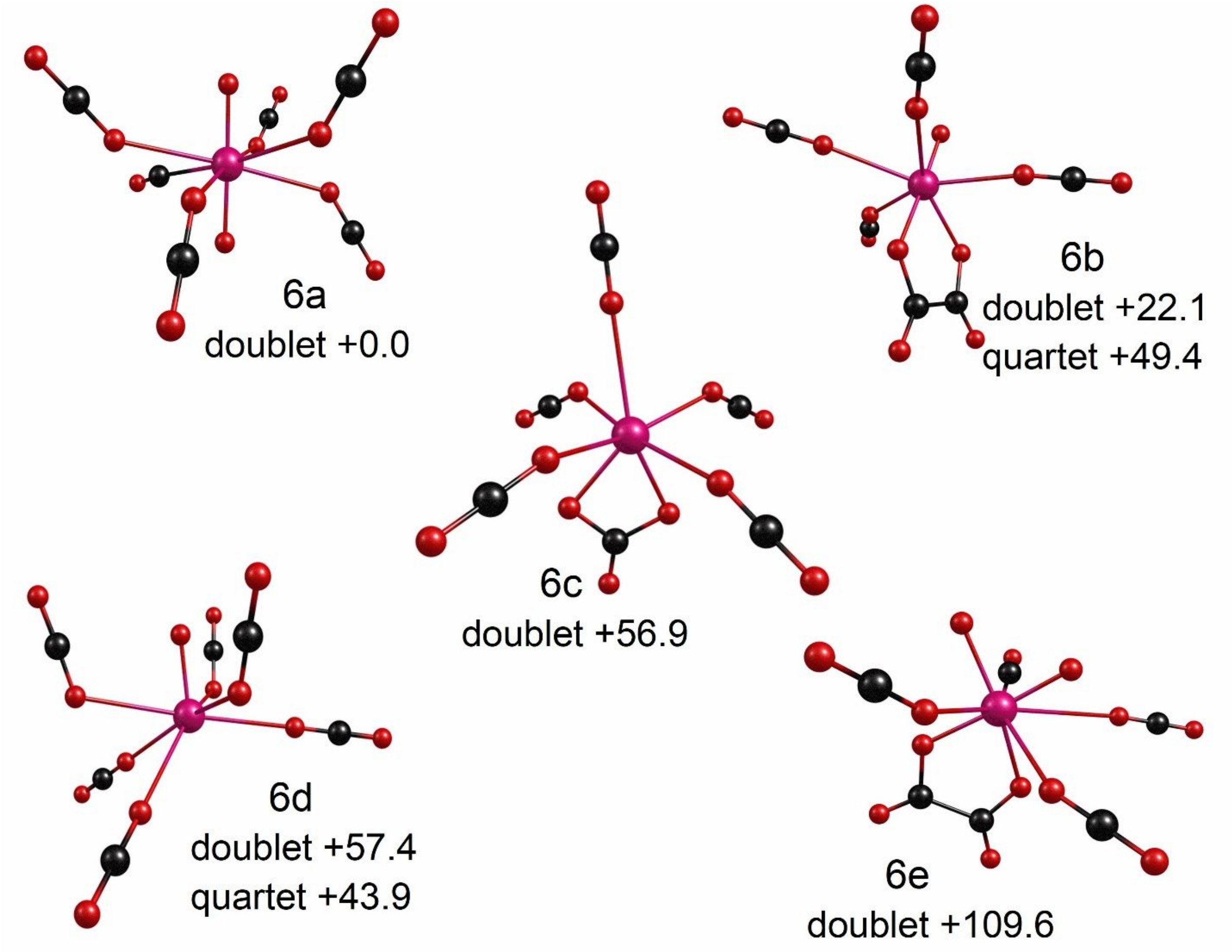

Figure S89. Predicted minimum energy structures of $UO^+(CO_2)_6$ with energy of each spin state in kcal/mol. The lowest energy spin state of each isomer is shown.

Table S101. Cartesian coordinates for the optimized geometry of isomer 6a-doublet $UO^+(CO_2)_6$ followed by its predicted frequencies (cm^{-1}) and IR intensities (km/mol).

Z	x	y	z
92	-0.000007000	-0.150231000	-0.092672000
8	-2.413029000	-1.487403000	0.093176000
6	-3.227230000	-1.916866000	0.818558000
8	-4.032483000	-2.340403000	1.518131000
8	-2.286126000	1.381132000	-0.392691000
6	-2.838758000	1.953987000	-1.253261000
8	-3.391134000	2.520083000	-2.085041000
8	2.413131000	-1.487291000	0.093137000
6	3.227325000	-1.916719000	0.818547000
8	4.032571000	-2.340219000	1.518152000
8	0.000038000	-0.560105000	1.661819000
6	0.000098000	-2.794779000	-1.075470000
8	0.000157000	-3.809004000	-1.549139000
8	2.285995000	1.381276000	-0.392673000
6	2.838688000	1.954099000	-1.253225000
8	3.391122000	2.520167000	-2.084986000
8	-0.000033000	0.203507000	-1.859855000
8	-0.000105000	2.361465000	1.119424000
8	-0.000054000	3.302366000	3.239847000
6	-0.000080000	2.829731000	2.193428000

Frequency	Intensity	Frequency	Intensity	Frequency	Intensity
11.1704	0.0199	112.8256	0.6286	661.9307	17.7361
11.3479	0.0525	123.4554	3.4409	662.0052	70.0805
18.5558	0.006	136.9526	1.3266	664.6142	27.0074
22.693	0.0179	142.5552	0.223	664.659	67.6965
25.7621	0.0019	143.6275	0.5392	665.3837	29.7335
26.3092	0.0189	145.3086	25.792	856.0638	0.3109
26.9725	0.0086	151.654	12.997	914.3233	391.0555
30.3256	0.015	168.1761	1.5436	1371.2488	44.7746
34.5853	0.0016	181.0711	3.4688	1371.6809	7.7169
35.3592	0.0057	217.9119	41.5824	1373.2358	25.9342
44.0773	0.0346	217.9353	49.0199	1373.988	42.2151
47.4128	0.0002	245.508	3.928	1375.0889	5.2644
59.4193	0.1317	260.4525	0.2854	2266.1437	110.6649
64.0936	0.0615	656.5484	0.0863	2417.0764	1899.0113
74.4458	1.3738	658.0045	40.5419	2417.3291	0.3719
84.7596	0.0006	659.3026	29.8952	2423.1429	972.3984
86.0972	1.361	660.1417	5.2154	2427.1321	1622.3173
96.468	0.0598	660.3886	0.6062	2446.7431	179.7772

Table S102. Cartesian coordinates for the optimized geometry of isomer 6b-doublet $UO^{+}(CO_2)_6$ followed by its predicted frequencies (cm^{-1}) and IR intensities (km/mol).

Z	x	y	z
92	-0.000004000	0.084172000	-0.332162000
8	-2.538535000	-0.503330000	-0.588633000
6	-3.386654000	-1.177365000	-1.045711000
8	-4.224246000	-1.822406000	-1.482842000
8	0.000082000	-2.066432000	-0.151096000
6	0.000144000	-2.720783000	1.026330000
8	0.000217000	-3.892683000	1.197583000
8	2.538561000	-0.503110000	-0.588661000
6	3.386750000	-1.177035000	-1.045772000
8	4.224411000	-1.821967000	-1.482932000
8	0.000048000	-0.390727000	1.670595000
6	0.000107000	-1.689261000	2.205247000
8	0.000126000	-1.922763000	3.356534000
8	-1.531508000	2.171022000	0.314744000
6	-2.327894000	2.995548000	0.562720000
8	-3.103029000	3.803372000	0.805696000
8	-0.000029000	0.586062000	-2.050849000
8	1.531268000	2.171180000	0.314787000
8	3.102655000	3.803655000	0.805754000
6	2.327587000	2.995768000	0.562769000

Frequency	Intensity	Frequency	Intensity	Frequency	Intensity
7.3667	0.0695	133.1882	7.3961	657.1424	57.9409
16.7686	0.9565	150.0457	20.5692	658.3169	1.1189
17.912	0.7166	154.4744	0.8541	734.2877	146.998
21.2158	0.1551	161.5201	8.9017	815.3461	0.0068
21.9092	0.2229	179.1616	21.0694	826.9372	366.3614
26.28	1.3698	184.0487	35.9922	903.6113	192.2414
26.9099	0.0339	296.9418	27.9476	924.6879	56.9831
31.8937	0.5254	309.7631	21.6843	1149.139	530.5939
52.4969	0.0058	336.8368	2.8822	1374.2969	60.9943
60.6032	2.9394	475.9941	52.7023	1375.2991	46.0196
66.9766	1.1685	542.3623	2.0301	1380.4127	42.9721
69.3568	0.2522	574.9518	27.5346	1382.4018	36.6874
87.2443	2.0723	649.332	1.1455	1863.0963	364.6896
89.28	1.2173	650.5217	5.3724	1906.809	412.6224
97.8579	0.035	651.1131	6.3792	2426.0356	25.8236
110.4013	0.2519	651.884	3.6554	2430.7158	1709.2241
120.4867	0.2798	652.9602	92.5828	2433.6922	2144.433
132.843	6.1271	655.221	54.4646	2454.134	236.3611

Table S103. Cartesian coordinates for the optimized geometry of isomer 6b-quartet $UO^{+}(CO_2)_6$ followed by its predicted frequencies (cm^{-1}) and IR intensities (km/mol).

Z	x	y	z
92	-0.000057000	0.138824000	-0.313992000
8	-2.524524000	-0.603719000	-0.606006000
6	-3.339567000	-1.098412000	-1.292377000
8	-4.145908000	-1.577431000	-1.949286000
8	0.000736000	-2.272556000	-0.111507000
6	0.001005000	-3.018685000	0.870430000
8	0.001374000	-4.114167000	1.277663000
8	2.524876000	-0.602070000	-0.606173000
6	3.340187000	-1.096235000	-1.292604000
8	4.146789000	-1.574733000	-1.949573000
8	0.000223000	-0.514996000	1.951701000
6	0.000596000	-1.640298000	2.505654000
8	0.000789000	-2.171062000	3.549012000
8	-1.568912000	2.157793000	0.444697000
6	-2.359305000	3.000243000	0.647241000
8	-3.129544000	3.824555000	0.848219000
8	-0.000237000	0.505509000	-2.095220000
8	1.567535000	2.158785000	0.444682000
8	3.127191000	3.826404000	0.848437000
6	2.357435000	3.001669000	0.647345000

Frequency	Intensity	Frequency	Intensity	Frequency	Intensity
7.1377	0.0096	126.7057	1.2475	654.7662	98.8352
15.3852	0.2817	136.5613	22.0297	656.0583	36.1888
19.426	0.156	140.339	1.0412	658.8567	54.6743
21.0485	0.2088	153.5408	62.1391	659.8417	9.05
21.6728	0.0615	154.4656	9.8297	674.5963	145.5515
22.4254	0.2917	164.784	3.7019	878.4742	252.868
26.7238	0.0303	166.4737	0.1307	1138.5947	136.0346
30.2612	0.0591	194.5762	6.943	1275.2074	211.862
43.7242	4.4908	206.7534	17.9665	1373.3637	47.8624
48.323	0.0219	305.8673	4.5032	1374.1466	39.4062
58.1004	2.1862	320.7317	46.1173	1379.1433	35.1564
69.7385	0.0176	447.2476	27.1279	1380.7112	34.226
82.1784	0.2147	622.1145	209.4935	1984.4745	242.0949
82.7312	0.1887	637.6339	1.0051	2074.311	1123.8241
92.8743	0.0228	650.1415	0.5262	2424.4033	29.2784
95.8272	0.3185	651.5909	16.5786	2428.3597	1677.6842
108.7051	1.3923	651.7571	24.975	2432.5427	1994.3438
114.2314	0.9764	654.354	0.5957	2452.479	353.654

Table S104. Cartesian coordinates for the optimized geometry of isomer 6c-doublet $UO^{+}(CO_2)_6$ followed by its predicted frequencies (cm^{-1}) and IR intensities (km/mol).

Z	x	y	z
92	0.072734000	0.076687000	-0.125997000
6	0.979206000	-1.139920000	2.083951000
8	1.374189000	-1.674704000	3.067259000
8	0.778265000	-1.724522000	0.862875000
8	0.634747000	0.183436000	1.963107000
6	-2.658497000	0.396592000	2.291873000
8	-2.256099000	0.341657000	1.188301000
8	-3.071683000	0.454566000	3.358393000
8	-2.014017000	-4.107640000	-0.817910000
6	-1.764699000	-2.996082000	-0.935027000
8	-1.522850000	-1.853590000	-1.071711000
8	3.067868000	3.541934000	1.186060000
6	2.382462000	2.873272000	0.558542000
8	1.681009000	2.198195000	-0.101176000
8	-3.403240000	2.665320000	-2.831985000
6	-2.600980000	2.106124000	-2.231690000
8	-1.777649000	1.536548000	-1.621059000
8	2.148937000	-0.530387000	-1.644186000
6	3.069574000	-1.236600000	-1.835532000
8	3.968776000	-1.915256000	-2.038084000

Frequency	Intensity	Frequency	Intensity	Frequency	Intensity
6.7894	0.4848	96.439	3.394	657.6959	28.9268
10.0221	2.8145	103.8662	1.8906	658.6338	64.1096
14.4937	0.4661	104.4447	4.7786	659.4578	51.2719
16.8726	0.9497	150.0807	12.9573	748.5647	157.1707
19.3029	2.1834	155.1605	12.5067	801.8843	20.0847
21.892	3.6446	160.7272	7.6055	941.5636	132.6353
25.6742	0.152	164.7102	14.5224	1037.4621	241.8725
27.8598	0.5137	174.1697	0.4912	1366.4114	35.0909
28.5417	2.7325	305.5859	5.3853	1367.1308	52.5277
35.2428	0.4881	358.3226	58.9441	1367.8319	18.1222
54.0808	0.0058	642.3415	0.2318	1369.6648	38.466
55.4418	0.0812	649.4472	10.3065	1370.8734	8.632
64.8161	0.4553	651.1309	8.4802	1867.1759	730.757
69.878	1.3635	652.9039	66.7304	2417.9221	464.3038
79.0769	0.2281	654.6073	17.2409	2419.9368	994.8457
86.3616	0.0462	655.0983	4.6941	2422.146	1730.7329
91.5083	0.1789	655.5752	9.4784	2426.701	1763.1977
92.5045	0.0688	656.6621	40.7027	2447.7206	17.2468

Table S105. Cartesian coordinates for the optimized geometry of isomer 6d-doublet $UO^+(CO_2)_6$ followed by its predicted frequencies (cm^{-1}) and IR intensities (km/mol).

Z	x	y	z
92	-0.136752000	-0.076990000	0.052759000
8	-0.468477000	-0.321305000	-1.760140000
8	1.788551000	-1.842212000	0.770209000
6	2.613110000	-2.590694000	1.131894000
8	3.420199000	-3.325982000	1.487288000
8	-2.094040000	-3.890906000	-1.482178000
6	-1.682423000	-3.200053000	-0.665015000
8	-1.267446000	-2.511501000	0.191757000
8	-2.622359000	0.225778000	1.015918000
6	-3.691811000	0.304283000	1.487137000
8	-4.738782000	0.383251000	1.950839000
8	-0.865789000	2.483452000	-0.382957000
6	-1.240606000	3.168590000	-1.258878000
8	-1.606761000	3.852401000	-2.103882000
8	2.292744000	0.505927000	-1.223834000
8	2.545839000	0.305062000	-3.522892000
6	2.406415000	0.399536000	-2.387602000
6	2.327639000	2.354242000	2.081825000
8	1.636353000	1.624358000	1.484228000
8	3.003375000	3.070132000	2.676894000

Frequency	Intensity	Frequency	Intensity	Frequency	Intensity
5.8867	0.1989	81.8159	0.8788	654.3485	60.4255
10.5827	0.1039	93.3606	0.5461	656.1394	49.6517
16.1601	0.2562	95.8882	0.3568	656.725	19.7583
18.5685	0.1018	96.8524	0.9618	658.4556	24.6578
19.5496	0.0015	106.3876	0.8869	664.462	25.8215
21.5764	0.4686	113.9175	0.6678	819.6418	363.8085
23.0961	0.5159	136.6413	31.1907	1363.0203	16.4915
24.024	0.6252	141.0953	33.1708	1364.595	22.6481
25.3163	1.1127	156.168	0.9854	1369.0895	23.4487
28.1664	1.5386	174.6836	3.5475	1370.8048	4.8804
40.3571	0.2988	196.7719	6.4274	1375.9723	22.4248
41.8922	1.6895	644.7357	0.5537	1376.3588	6.5957
45.7821	1.037	645.6999	8.5851	2409.8036	471.9889
57.0416	0.0532	646.347	20.6144	2416.2159	289.5976
63.5371	0.119	646.5141	14.9815	2417.4661	1611.7907
69.9049	0.8734	648.4653	71.2908	2423.6778	1507.8609
74.9895	0.5043	650.3479	32.9269	2429.023	2104.3879
78.0462	1.28	653.3462	25.4434	2450.8987	20.1653

Table S106. Cartesian coordinates for the optimized geometry of isomer 6d-quartet $UO^+(CO_2)_6$ followed by its predicted frequencies (cm^{-1}) and IR intensities (km/mol).

Z	x	y	z
92	0.113631000	0.039272000	-0.106238000
8	0.501867000	0.211556000	1.720232000
8	2.564248000	-0.132356000	-1.216765000
6	3.623669000	-0.188969000	-1.713228000
8	4.660776000	-0.245238000	-2.201660000
8	2.008364000	3.633487000	1.791463000
6	1.608384000	3.065124000	0.879937000
8	1.206441000	2.507489000	-0.073617000
8	-1.788284000	1.820115000	-0.969286000
6	-2.598755000	2.578982000	-1.340527000
8	-3.392159000	3.324139000	-1.706761000
8	-2.332146000	-0.280860000	1.222188000
6	-2.374103000	-0.188970000	2.392352000
8	-2.450071000	-0.105844000	3.534284000
8	1.050031000	-2.447691000	0.386674000
8	1.846095000	-3.341388000	2.375623000
6	1.450928000	-2.887707000	1.399769000
6	-2.356144000	-2.615705000	-1.766530000
8	-1.635963000	-1.815380000	-1.308733000
8	-3.061436000	-3.401725000	-2.220736000

Frequency	Intensity	Frequency	Intensity	Frequency	Intensity
5.7452	0.1358	85.1634	1.9814	655.413	72.585
11.9301	0.0803	93.2307	0.7103	657.2821	56.1917
17.2042	0.0736	95.961	0.2774	660.4815	2.4973
18.6129	0.2575	97.5202	0.7052	662.0699	42.3108
19.5295	0.0293	102.4264	0.7046	665.8983	30.7786
21.9371	0.2624	113.7228	0.3824	803.7557	309.0242
23.882	0.419	139.5316	34.823	1362.4344	12.5843
25.4488	0.0652	145.8616	40.2296	1363.2412	23.5507
26.9566	0.9086	174.9521	1.9618	1364.8451	28.8012
29.695	3.0804	188.333	3.6682	1373.2219	10.5399
43.038	0.5359	202.1189	7.0586	1375.8894	21.5662
44.9669	0.6324	647.1706	32.7204	1376.4463	11.2066
48.7671	3.0393	649.714	7.1199	2409.1815	268.9389
55.4666	0.0695	650.0971	13.7315	2414.3877	130.3134
63.7899	0.0808	651.2852	12.1648	2416.7763	2083.1976
72.4904	1.0342	653.1099	7.8104	2423.5848	1332.561
77.2351	0.8613	653.9621	36.2732	2428.4998	1924.8685
83.4316	0.6333	654.529	71.0067	2450.1469	16.8835

Table S107. Cartesian coordinates for the optimized geometry of isomer 6e-doublet $UO^+(CO_2)_6$ followed by its predicted frequencies (cm^{-1}) and IR intensities (km/mol).

Z	x	y	z
92	0.283085000	-0.092565000	0.452406000
8	-3.540066000	0.431966000	0.543008000
6	-4.589667000	0.939004000	0.482697000
8	-5.625383000	1.442379000	0.422113000
8	-0.838916000	-0.154599000	-1.360410000
6	-0.612106000	-1.046582000	-2.356069000
8	-1.170285000	-1.111742000	-3.393126000
8	2.860842000	0.535552000	0.310362000
6	3.965256000	0.462998000	0.704814000
8	5.045618000	0.402290000	1.077613000
8	0.973549000	-1.652659000	-0.626080000
6	0.537400000	-2.021170000	-1.935781000
8	0.997443000	-2.908274000	-2.540692000
8	1.039156000	-0.798168000	2.198714000
6	-1.672126000	-1.776200000	1.296759000
8	-2.436542000	-2.470589000	1.720250000
8	-0.448831000	1.146888000	1.500696000
8	0.331996000	4.227741000	-1.782972000
8	0.885288000	2.153216000	-0.905858000
6	0.598779000	3.202617000	-1.347466000

Frequency	Intensity	Frequency	Intensity	Frequency	Intensity
11.0093	0.0143	152.4041	2.8532	658.4451	48.5349
14.7992	0.0949	166.9425	0.1514	666.5374	21.3726
19.1129	0.1416	169.6472	0.2167	668.9531	24.7677
23.2609	0.1893	179.0844	0.6992	679.3727	202.676
25.5219	0.4707	191.5928	8.8357	791.887	236.7794
28.2043	0.0613	212.505	21.7887	799.2372	0.8811
34.8252	0.8581	255.0476	4.4638	884.4794	96.803
39.0107	0.8431	277.6085	12.5201	891.4785	116.5813
41.8566	1.0529	286.3271	4.0878	1088.3785	370.0129
46.2633	0.2687	309.595	14.6019	1372.0633	7.6846
53.9302	0.075	336.1347	5.3547	1374.2939	55.8603
65.3599	0.7293	444.3319	31.309	1377.6532	43.0561
68.5621	0.5191	468.8501	50.3099	1877.2252	331.3986
75.431	1.8287	534.0418	2.4987	1935.7725	378.9574
94.2063	0.5178	575.3095	5.1438	2301.2627	56.3118
105.5395	14.6733	650.9927	6.2952	2417.9725	766.8627
117.9665	0.0831	653.0374	30.6897	2425.7897	1502.7431
141.7884	29.3507	654.8999	13.6635	2442.4915	694.4063

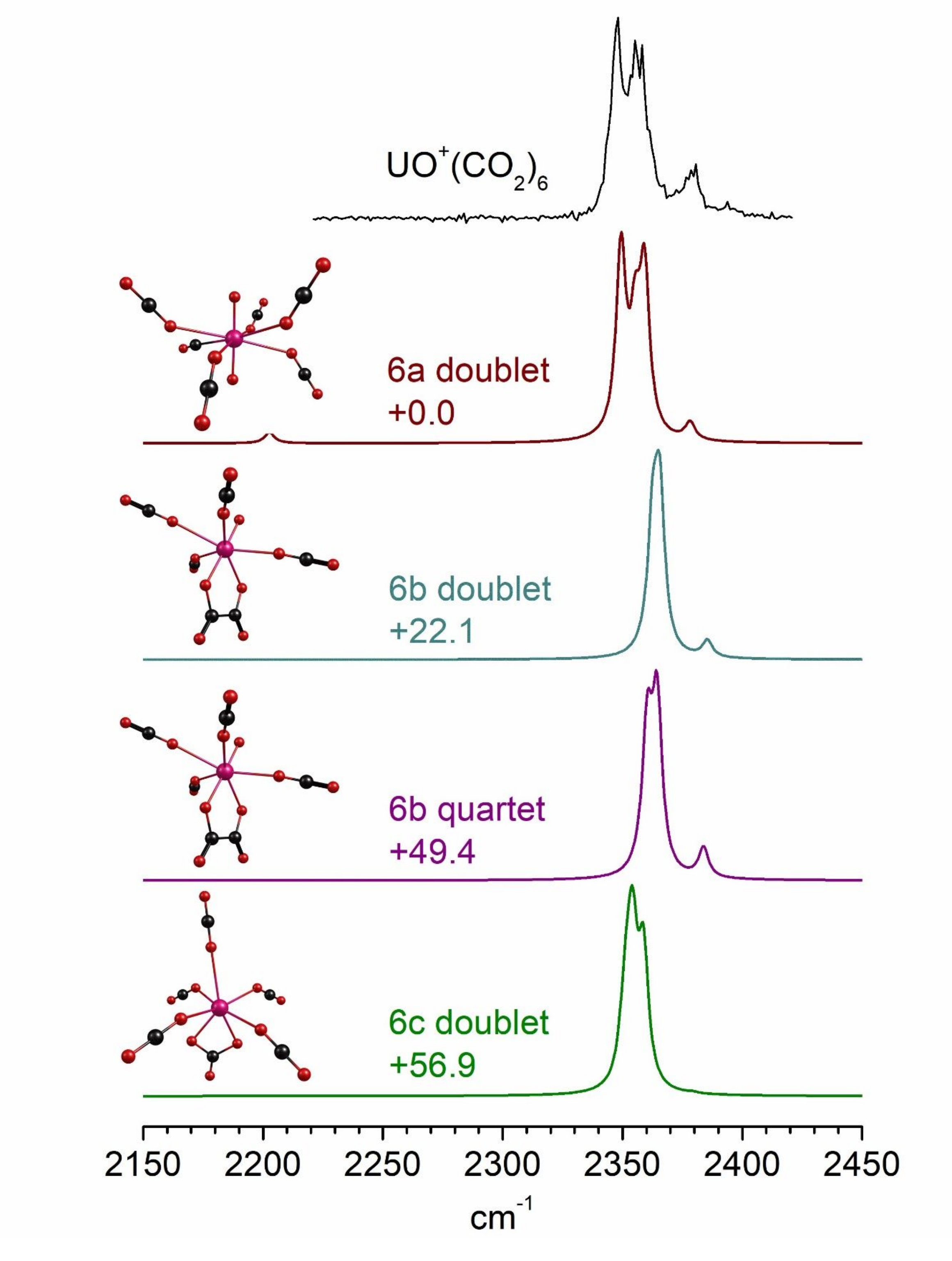

Figure S90. Experimental IR spectrum of $UO^+(CO_2)_6$ compared with simulated spectra for isomers 6a, 6b and 6c. Relative energies (kcal/mol) are shown next to each spectrum.

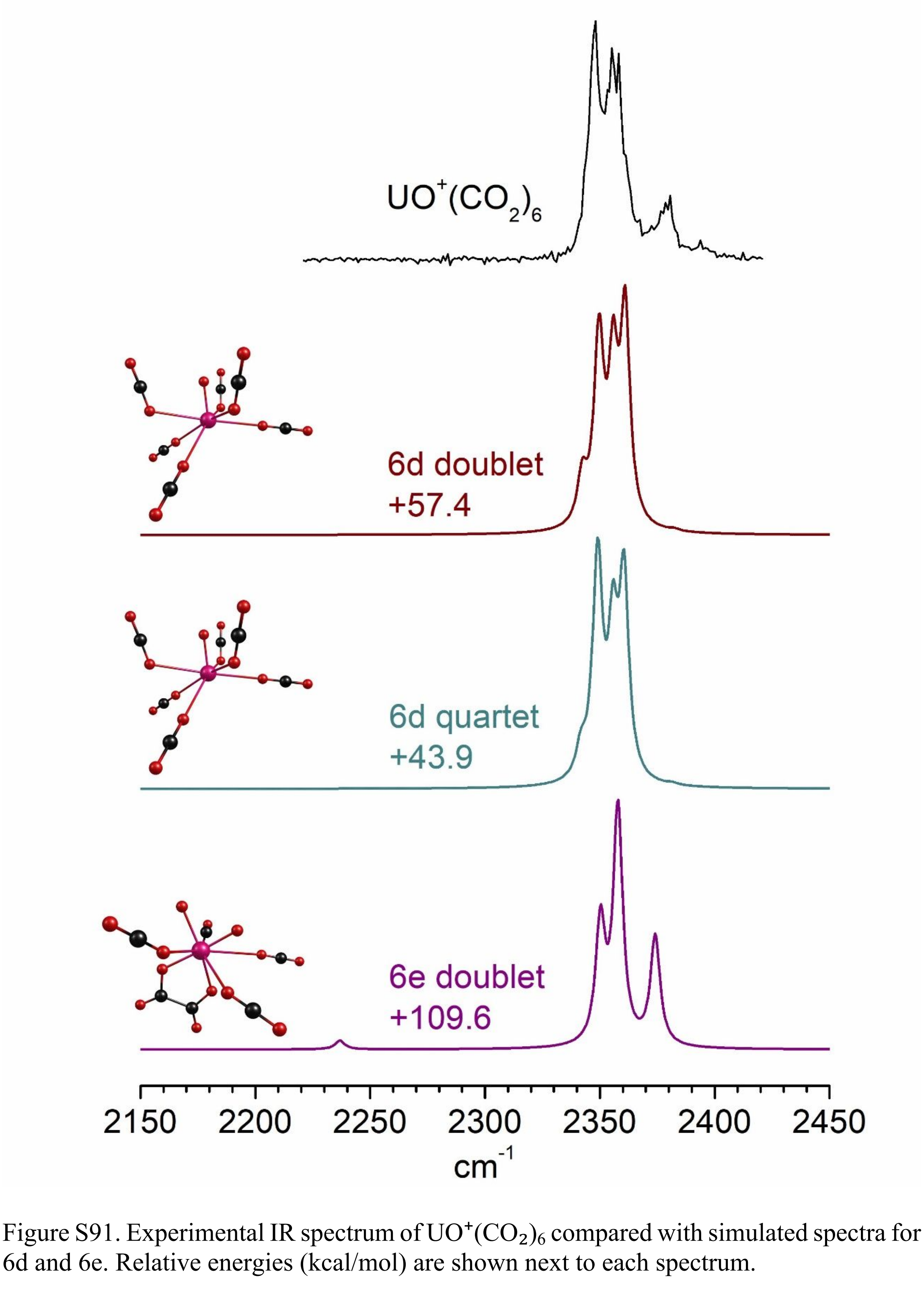

Figure S91. Experimental IR spectrum of $UO^{+}(CO_2)_6$ compared with simulated spectra for isomers 6d and 6e. Relative energies (kcal/mol) are shown next to each spectrum.

Table S108. $UO^+(CO_2)_7$ electronic energy calculated at the B3LYP/cc-pVTZ(-pp) level with Stuttgart/Koeln pseudopotential.

Isomer	2s + 1	Energy (hartree)	Rel. E (kcal/mol)	BDE (CO_2) (kcal/mol)	BDE (CO) (kcal/mol)	BDE (oxalate) (kcal/mol)
7a	2	-1870.534952	+0.0	2.3	2.3	
7a	4	-1870.434969	+62.7		2.3	
7b	2	-1870.501218	+21.2	3.2		41.8
7b	4	-1870.457312	+48.7	2.9		1.2
7c	2	-1870.451041	+52.7			
7c	4	-1870.475781	+37.1			
7d	2	-1870.44445	+56.8	2.9		
7d	4	-1870.46641	+43.0	3.2		
7e	2	-1870.360609	+109.4	2.4		

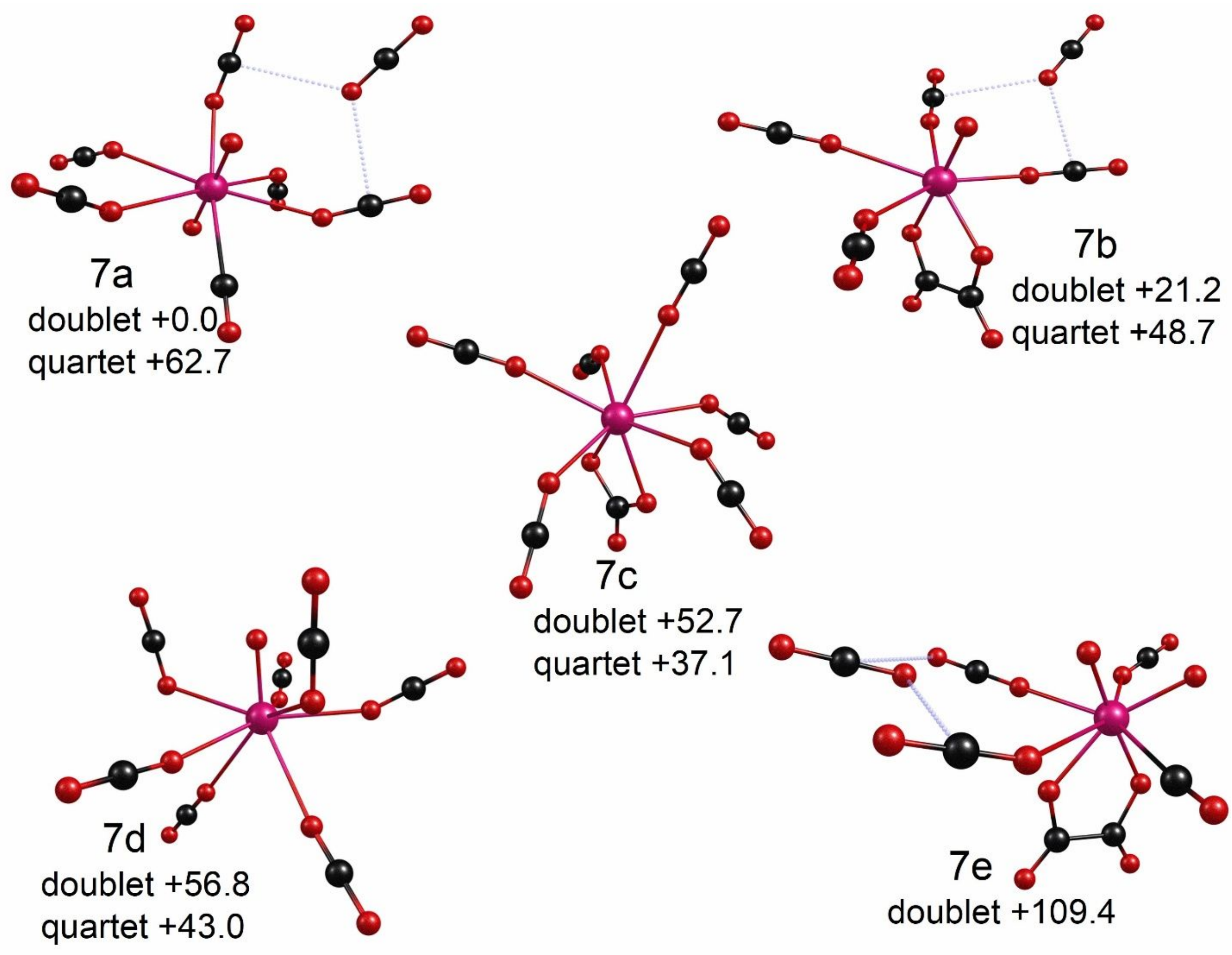

Figure S92. Predicted minimum energy structures of $UO^+(CO_2)_7$ with energy of each spin state in kcal/mol. The lowest energy spin state of each isomer is shown.

Table S109. Cartesian coordinates for the optimized geometry of isomer 7a-doublet $UO^+(CO_2)_7$ followed by its predicted frequencies (cm^{-1}) and IR intensities (km/mol).

Z	x	y	z
92	-0.521464000	-0.188695000	-0.189990000
8	1.501108000	-1.926735000	-0.841885000
6	2.517186000	-2.381313000	-0.477707000
8	3.512382000	-2.840697000	-0.136102000
8	1.555555000	0.807111000	-1.847491000
6	1.592802000	1.150068000	-2.966792000
8	1.644876000	1.490446000	-4.062867000
8	-2.254924000	1.870956000	0.360332000
6	-2.989225000	2.606042000	-0.181931000
8	-3.711347000	3.333340000	-0.698660000
8	-1.258937000	0.077191000	-1.813332000
6	-1.358614000	-2.828056000	-0.682796000
8	-1.716883000	-3.855104000	-0.947144000
8	0.648046000	2.233146000	0.517030000
6	1.381574000	2.616330000	1.345140000
8	2.098025000	3.010451000	2.152102000
8	0.128195000	-0.529089000	1.455372000
8	5.420544000	0.241092000	2.361480000
8	3.566523000	0.264115000	0.965569000
6	4.498870000	0.252435000	1.667788000
8	-3.954386000	-1.319917000	3.067911000
8	-2.859142000	-0.910447000	1.064698000
6	-3.406330000	-1.116663000	2.080131000

Frequency	Intensity	Frequency	Intensity	Frequency	Intensity
10.1593	0.035	59.0212	0.2736	216.915	42.9596
11.2827	0.004	62.5854	0.2555	222.9722	45.7107
12.4523	0.0264	71.9939	0.83	245.272	3.4812
20.7342	0.0024	79.3679	1.165	258.4507	0.2067
21.9292	0.0262	85.8461	0.3799	655.8962	4.5768
23.843	0.0833	90.2542	0.3906	657.4003	51.2616
26.1799	0.0027	97.528	0.0983	658.311	29.0422
28.1883	0.0307	114.2248	0.3948	659.6481	9.6956
29.854	0.071	125.069	3.5783	659.8549	3.6403
30.9474	0.0807	136.0157	2.2591	661.204	39.8487
34.3836	0.1701	138.8911	0.4849	662.7916	25.0104
35.1746	0.2192	144.2644	4.485	664.1131	51.0452
39.5357	0.0424	145.8048	23.2711	665.1958	47.2186
44.8554	0.1027	152.6655	11.4317	665.6188	9.1954
48.3458	0.2165	169.0067	1.871	669.4253	62.1133
53.2957	0.0582	183.7449	4.4393	672.2731	26.9647

855.939	0.7056	1373.9062	21.9281	2417.5102	756.973
914.354	403.1227	1374.9324	40.207	2419.1212	113.8042
1369.8796	6.7964	1375.9042	5.614	2422.8217	953.3547
1370.8249	24.6126	2265.5613	119.8871	2427.274	1639.1179
1372.6115	22.0818	2411.5359	1768.5866	2446.8612	219.0349

Table S110. Cartesian coordinates for the optimized geometry of isomer 7a-quartet $UO^+(CO_2)_7$ followed by its predicted frequencies (cm^{-1}) and IR intensities (km/mol).

Z	x	y	z
92	-0.530909000	0.184376000	0.174041000
8	1.461562000	1.926925000	0.866426000
6	2.462596000	2.415082000	0.502823000
8	3.442097000	2.907238000	0.162317000
8	1.480954000	-0.870503000	1.835233000
6	1.516948000	-1.234656000	2.947894000
8	1.567959000	-1.595728000	4.037262000
8	-2.243775000	-1.891947000	-0.373986000
6	-2.988377000	-2.615246000	0.170047000
8	-3.720399000	-3.331027000	0.688977000
8	-1.336578000	-0.088412000	1.838527000
6	-1.471961000	2.735624000	0.857776000
8	-1.890574000	3.699811000	1.244432000
8	0.655860000	-2.221401000	-0.594668000
6	1.398785000	-2.528647000	-1.445547000
8	2.126135000	-2.848893000	-2.275491000
8	0.196259000	0.525841000	-1.510818000
8	5.565483000	-0.226770000	-2.134214000
8	3.638888000	-0.218009000	-0.840123000
6	4.607632000	-0.222150000	-1.491162000
8	-3.734884000	1.404068000	-3.248460000
8	-2.798712000	0.922187000	-1.181520000
6	-3.265385000	1.165061000	-2.228983000

Frequency	Intensity	Frequency	Intensity	Frequency	Intensity
8.4595	0.0056	57.4271	0.085	206.2537	26.3564
10.3278	0.0399	63.3088	0.1876	212.7072	32.1154
13.6096	0.0385	71.977	0.5823	245.4748	0.1332
20.4973	0.0069	79.7942	1.5859	262.1541	6.4628
21.9221	0.0248	86.9897	0.3768	627.7239	1.9939
23.9701	0.1135	91.386	0.5407	656.3433	14.3279
25.7378	0.0003	97.1245	0.295	657.7763	33.0146
27.3997	0.0231	108.5426	0.4133	658.8845	15.4283
29.6083	0.0351	121.9311	1.4924	659.1272	16.168
30.8767	0.1869	132.7995	6.1676	659.4722	20.6692
32.9949	0.1814	133.3977	2.4827	660.7343	32.0791
34.878	0.0582	141.6796	3.5424	662.1991	17.0896
37.8081	0.0749	146.1711	27.2837	663.8734	72.0818
42.4666	0.0583	150.2993	13.8569	664.7211	33.6943
47.1797	0.173	168.1322	1.3435	665.5601	21.9471
53.1168	0.0947	177.9522	2.8372	669.3279	56.6278

672.1758	28.4857	1373.1855	25.0235	2416.8292	703.36
701.2276	0.5632	1374.0456	40.6412	2419.0924	83.6599
1369.8887	6.6071	1375.1274	5.0167	2422.1181	1121.223
1370.9533	30.2821	2255.4346	173.5475	2426.4171	1557.6726
1372.2299	16.5145	2411.5121	1836.8879	2446.0128	204.6953

Table S111. Cartesian coordinates for the optimized geometry of isomer 7b-doublet $UO^+(CO_2)_7$ followed by its predicted frequencies (cm^{-1}) and IR intensities (km/mol).

Z	x	y	z
92	0.412737000	0.049509000	-0.236112000
8	-1.632924000	-1.508069000	0.056176000
6	-2.444433000	-2.287648000	-0.278923000
8	-3.234165000	-3.054479000	-0.591816000
8	1.360358000	-1.886798000	-0.307168000
6	1.895611000	-2.500066000	0.765503000
8	2.423583000	-3.559891000	0.798748000
8	2.833435000	0.577090000	-1.120950000
6	3.717494000	0.279819000	-1.836493000
8	4.587420000	0.006188000	-2.527753000
8	1.074183000	-0.409507000	1.658949000
6	1.728895000	-1.593432000	2.031986000
8	2.080700000	-1.829314000	3.128024000
8	-1.599437000	1.388065000	0.889319000
6	-2.600517000	1.866819000	1.266738000
8	-3.569596000	2.347316000	1.645330000
8	-0.202562000	0.517805000	-1.851517000
8	1.159644000	2.553888000	0.316305000
8	2.155144000	4.599030000	0.765023000
6	1.665414000	3.587129000	0.543254000
8	-6.613803000	0.153216000	-1.072838000
6	-5.516411000	0.063443000	-0.733246000
8	-4.402996000	-0.025945000	-0.389658000

Frequency	Intensity	Frequency	Intensity	Frequency	Intensity
4.4354	0.1452	80.6684	0.062	476.2694	53.9747
7.8137	0.0183	89.0539	2.0235	542.4242	2.1096
16.9342	0.6679	96.9586	1.0074	574.7841	28.7382
18.5965	0.3466	102.4921	0.3718	642.965	33.702
20.0439	0.9131	111.273	0.3278	648.9615	32.9658
23.046	0.8092	125.8323	0.7375	650.7459	16.7428
26.1623	0.8613	134.2094	8.9224	651.9314	4.3519
31.8311	0.3635	135.3591	5.2376	653.0481	47.6002
34.2552	0.3203	149.6354	18.5946	654.629	40.504
37.3843	0.298	158.8573	3.1638	655.9593	35.7627
45.4861	0.2427	161.917	8.2259	659.0141	25.7655
53.1107	0.0992	180.3439	12.431	667.9181	37.2021
58.4524	0.284	184.9162	50.0408	671.5464	28.9292
63.8822	3.8365	296.5745	27.7403	735.6374	147.0847
66.537	1.0452	308.3662	20.4576	815.5545	0.1358
71.2977	0.0372	336.4835	3.6929	827.7753	380.426

897.0048	182.6487	1379.9051	48.6462	2417.1021	1169.5531
925.9168	57.895	1380.9155	47.8694	2425.9753	128.7852
1149.9998	551.0793	1383.3033	32.3173	2430.7386	1409.7572
1369.9271	9.2629	1862.4675	377.614	2436.0318	1946.4658
1374.1318	51.4322	1905.3752	428.9505	2455.0863	293.0939

Table S112. Cartesian coordinates for the optimized geometry of isomer 7b-quartet $UO^+(CO_2)_7$ followed by its predicted frequencies (cm^{-1}) and IR intensities (km/mol).

Z	x	y	z
92	-0.079560000	0.016962000	-0.291411000
8	-0.735495000	-2.533816000	-0.661935000
6	-0.765243000	-3.394312000	-1.460916000
8	-0.802369000	-4.244694000	-2.227342000
8	1.988737000	-1.143538000	-0.637675000
6	2.972536000	-1.338867000	0.087722000
8	4.041259000	-1.809211000	0.167730000
8	1.666675000	1.914194000	-0.972355000
6	2.125863000	2.356179000	-1.959695000
8	2.583903000	2.800844000	-2.910659000
8	1.271147000	0.005586000	1.693357000
6	2.425773000	-0.420034000	1.904876000
8	3.272440000	-0.491096000	2.711868000
8	-1.520906000	-1.035749000	1.884527000
6	-1.854992000	-1.422957000	2.937731000
8	-2.187895000	-1.803397000	3.968538000
8	-0.578816000	0.235390000	-2.034486000
8	-0.706968000	2.345597000	0.921102000
8	-0.712824000	4.263803000	2.223815000
6	-0.706541000	3.314298000	1.580948000
8	-4.300658000	0.921758000	-1.997118000
6	-3.568367000	0.721342000	-1.136717000
8	-2.835062000	0.517527000	-0.243599000

Frequency	Intensity	Frequency	Intensity	Frequency	Intensity
10.3726	0.0908	79.4373	2.7305	205.7372	3.2962
14.3712	0.2451	85.0236	1.9775	213.2618	32.2165
17.2041	0.3218	88.827	0.9298	313.8243	7.3934
18.9231	0.0427	95.0242	0.2285	319.7599	45.1188
20.899	0.2217	96.3232	0.283	455.0675	27.812
21.409	0.0775	103.0395	1.8527	640.4814	165.0454
24.7856	0.272	113.1746	1.8541	644.77	2.565
25.9598	0.1548	125.3211	0.8036	653.206	11.1802
28.4992	0.3436	132.4947	28.2658	654.0187	21.1571
28.848	0.6841	136.9356	6.6777	656.0727	4.4707
36.3077	3.2057	150.8252	56.0808	656.3168	9.3093
48.7968	1.477	158.8542	8.7395	657.5436	21.6791
49.361	0.0655	178.1544	1.4138	658.7195	88.7751
51.2381	0.5237	178.5789	2.7712	660.4012	37.7515
74.0364	0.2962	195.658	2.7851	662.521	27.8178

662.8124	50.1633	1367.3984	24.2337	2054.7076	1231.4021
664.8875	40.3111	1369.5985	39.031	2415.3504	860.3648
671.8272	151.8722	1372.4204	45.5241	2421.7015	143.6436
867.1272	241.4784	1377.7152	23.8287	2424.7042	2030.3426
1149.2374	139.6966	1379.3095	27.2047	2430.0624	1629.3631
1279.7012	220.612	1982.7454	123.7458	2450.0171	164.378

Table S113. Cartesian coordinates for the optimized geometry of isomer 7c-doublet $UO^+(CO_2)_7$ followed by its predicted frequencies (cm^{-1}) and IR intensities (km/mol).

Z	x	y	z
92	-0.139788000	0.007516000	-0.101783000
6	0.523208000	1.373506000	2.134864000
8	-0.403684000	0.365521000	2.025089000
8	1.062332000	1.563328000	0.901475000
8	0.797310000	1.960789000	3.131943000
6	3.138768000	-1.312816000	1.098563000
8	2.146726000	-1.319626000	0.470642000
8	4.113360000	-1.322587000	1.702160000
8	3.280062000	2.753133000	-1.668625000
6	2.471333000	1.943885000	-1.736589000
8	1.648520000	1.110346000	-1.834077000
8	-5.087565000	-0.505187000	-0.706995000
6	-3.942634000	-0.454172000	-0.664249000
8	-2.771413000	-0.406450000	-0.625781000
8	0.927278000	-3.622238000	-3.528476000
6	0.651194000	-2.795632000	-2.780789000
8	0.365458000	-1.951257000	-2.020507000
8	-1.397194000	2.283059000	-0.745239000
6	-1.416565000	3.429178000	-0.483509000
8	-1.450367000	4.548832000	-0.246357000
8	-1.136839000	-2.910776000	3.338377000
6	-0.978460000	-2.603750000	2.245964000
8	-0.821561000	-2.318463000	1.116180000

Frequency	Intensity	Frequency	Intensity	Frequency	Intensity
7.0821	0.5431	71.6612	0.232	359.0433	58.1701
14.2897	0.2579	77.1365	0.4272	648.9255	0.7973
16.9013	0.6534	79.4566	0.4165	649.5428	20.1179
20.0745	0.8911	87.524	0.3248	650.4762	16.2255
21.9471	0.2151	93.4981	0.2002	653.6753	12.3015
23.5831	0.6023	97.6155	1.1344	654.1816	11.037
25.7147	0.4112	103.5496	3.1092	654.7292	26.8076
26.147	1.3535	105.3469	0.4091	656.3378	44.4867
27.0325	0.3063	113.8972	2.5212	657.0101	29.9825
31.1995	2.9347	122.6345	14.8573	658.2185	99.3539
35.6788	6.8547	141.5525	15.0976	659.0639	54.1388
37.5043	0.6159	152.244	17.5496	661.43	23.9657
53.219	0.1639	160.6907	5.0439	661.5347	38.349
57.1222	0.0333	167.5649	2.6889	662.908	5.2748
65.0704	0.4244	173.7954	0.656	747.3522	144.4636
67.6237	0.3159	304.3448	8.0667	809.7939	21.8628

950.5608	124.8321	1369.7839	28.7479	2415.0843	414.1518
1063.7941	250.0613	1371.4881	16.5734	2420.7335	1065.8706
1363.9401	38.3252	1373.1611	17.1059	2423.0637	2111.2443
1364.368	7.8356	1856.4517	705.3815	2427.5038	2062.7712
1368.3719	36.0022	2412.9442	29.1352	2451.2095	19.5291

Table S114. Cartesian coordinates for the optimized geometry of isomer 7c-quartet $UO^+(CO_2)_7$ followed by its predicted frequencies (cm^{-1}) and IR intensities (km/mol).

Z	x	y	z
92	-0.103549000	0.072115000	-0.080833000
6	1.537717000	0.894399000	1.911179000
8	0.203067000	0.594521000	2.016108000
8	1.902113000	0.685436000	0.617288000
8	2.239209000	1.268170000	2.796495000
6	2.087836000	-2.764037000	0.882816000
8	1.136291000	-2.292986000	0.380153000
8	3.010633000	-3.242729000	1.364990000
8	3.902013000	0.619612000	-2.372477000
6	2.793400000	0.358496000	-2.247366000
8	1.654562000	0.084391000	-2.147405000
8	-4.696102000	2.096628000	-0.635889000
6	-3.672363000	1.582002000	-0.582245000
8	-2.627390000	1.052569000	-0.530910000
8	-1.698491000	-3.750366000	-3.075214000
6	-1.382189000	-2.850159000	-2.436589000
8	-1.062075000	-1.929122000	-1.787296000
8	-0.066261000	2.691721000	-0.631533000
6	0.505319000	3.691676000	-0.395680000
8	1.048418000	4.676766000	-0.182164000
8	-1.987950000	-1.571770000	3.790383000
6	-1.867851000	-1.471952000	2.655510000
8	-1.768629000	-1.392485000	1.486332000

Frequency	Intensity	Frequency	Intensity	Frequency	Intensity
8.5572	0.7556	70.7435	0.0919	358.2046	60.8081
13.7596	0.5459	76.6744	0.6455	649.2546	11.6222
16.0037	0.4014	79.3263	0.507	652.0228	10.2865
19.3683	0.7937	86.9257	0.5137	654.0594	1.6547
20.9219	0.1007	93.3812	0.2502	654.8912	6.6397
23.3558	0.1093	96.0276	0.4653	655.0311	18.7996
23.7841	0.5181	100.8792	1.4876	656.3826	4.2404
25.6706	0.3403	104.9323	2.5182	657.4667	45.2663
27.1218	0.9505	112.5576	1.341	658.1518	46.3749
30.9163	3.6311	120.9773	16.4337	658.6283	118.1624
35.2589	0.707	145.3622	26.6015	659.8671	47.4529
37.5547	7.3114	153.7614	8.6127	661.4094	29.8123
50.3963	0.0417	163.3543	6.074	661.7743	46.0393
57.606	0.1782	168.5277	2.4695	663.2058	3.0801
61.3608	0.2611	175.6289	1.0835	746.8608	138.8553
65.8209	0.2495	306.089	8.3448	812.2335	22.8023

952.0396	119.9652	1369.5994	32.783	2415.4866	533.0458
1063.9391	254.1605	1373.5037	24.2535	2420.7187	874.6122
1363.5011	34.6687	1374.1987	17.6129	2423.8791	2194.07
1364.415	13.6946	1853.154	683.92	2427.0359	1970.7019
1368.8858	36.1663	2413.8689	21.8326	2450.8519	9.4697

Table S115. Cartesian coordinates for the optimized geometry of isomer 7d-quartet $UO^{+}(CO_2)_7$ followed by its predicted frequencies (cm^{-1}) and IR intensities (km/mol).

Z	x	y	z
92	0.000117000	0.125847000	-0.045392000
8	0.001923000	0.795314000	-1.803590000
8	1.592655000	2.151528000	0.781130000
6	2.267986000	3.099949000	0.653274000
8	2.932653000	4.029449000	0.539510000
8	3.558095000	-1.889056000	3.205118000
6	2.733541000	-1.493918000	2.505542000
8	1.892415000	-1.092316000	1.800524000
8	2.512416000	-0.483820000	-1.110341000
6	2.716525000	-0.191090000	-2.229930000
8	2.956285000	0.077644000	-3.319352000
8	-2.510840000	-0.481524000	-1.114402000
6	-2.712860000	-0.188443000	-2.234282000
8	-2.950623000	0.080661000	-3.324047000
8	-1.895409000	-1.093398000	1.796934000
8	-3.562943000	-1.890885000	3.198901000
6	-2.737462000	-1.495377000	2.500626000
6	-2.269043000	3.099582000	0.654624000
8	-1.593334000	2.151150000	0.780293000
8	-2.934070000	4.029085000	0.542956000
8	-0.000002000	-2.540047000	-0.601102000
6	0.000157000	-3.626336000	-1.036356000
8	0.000304000	-4.694301000	-1.460652000

Frequency	Intensity	Frequency	Intensity	Frequency	Intensity
3.7907	0.1255	59.686	0.0754	178.8379	1.6464
12.8414	0.084	63.5433	0.9786	221.1637	15.1467
13.342	0.2039	66.5445	1.5865	642.1332	21.4486
17.2969	0.0357	67.6031	0.0423	642.6596	62.1894
17.5729	0.0354	70.4868	0.0001	647.6853	17.939
19.4269	0.3717	73.578	1.5926	650.4939	3.3775
20.0243	0.3672	82.0543	1.487	651.0884	11.6444
21.5304	0.9587	86.042	0.3176	652.9977	19.9756
22.7287	0.0003	90.7886	0.467	653.0254	12.5433
23.9271	0.1241	90.8001	1.2888	654.2709	68.0341
28.1538	0.0526	97.7196	0.3351	655.3815	52.4169
35.2604	0.0037	109.0699	0.4599	655.9058	86.1223
40.2169	1.757	123.3378	1.6008	660.3867	20.2384
46.0199	0.483	131.9894	42.4588	662.0012	32.7633
47.1917	0.0798	132.7735	21.0098	666.7987	7.9019
55.614	4.0007	138.6572	8.5556	667.8898	36.47

794.3141	301.2446	1372.3975	10.2692	2416.4093	150.4044
1359.8776	2.9241	1372.9052	22.7403	2418.1767	1153.0652
1360.251	29.1007	1373.3744	1.2641	2423.1734	1736.8685
1370.6497	3.9645	2406.4455	4.8079	2427.6742	2440.2715
1370.7298	6.5352	2409.8654	1165.1075	2452.5314	26.033

Table S116. Cartesian coordinates for the optimized geometry of isomer 7e-doublet $UO^+(CO_2)_7$ followed by its predicted frequencies (cm^{-1}) and IR intensities (km/mol).

Z	x	y	z
92	-0.742348000	-0.149292000	-0.437044000
8	2.446341000	-2.098803000	-0.824779000
6	3.589070000	-1.878345000	-0.768182000
8	4.720257000	-1.653751000	-0.711050000
8	0.360781000	-0.734086000	1.293332000
6	-0.211748000	-1.173904000	2.439226000
8	0.337011000	-1.476485000	3.439048000
8	-2.400182000	1.952549000	-0.295624000
6	-3.394730000	2.470409000	-0.646271000
8	-4.360318000	2.988121000	-0.978456000
8	-2.073301000	-0.801405000	0.935064000
6	-1.765160000	-1.230874000	2.260713000
8	-2.575569000	-1.557641000	3.036452000
8	-2.038407000	-0.407724000	-1.981100000
6	-0.410969000	-2.767187000	-1.078752000
8	-0.313545000	-3.827198000	-1.414545000
8	0.401150000	0.249804000	-1.741200000
8	1.793578000	3.775777000	1.088959000
8	0.287058000	2.096561000	0.555956000
6	1.061244000	2.937142000	0.820367000
8	5.779715000	1.891307000	0.162118000
8	3.526318000	1.339820000	0.094913000
6	4.660446000	1.616109000	0.128782000

Frequency	Intensity	Frequency	Intensity	Frequency	Intensity
5.0031	0.0483	68.8267	0.3017	309.6326	15.1554
12.3379	0.002	74.515	1.8147	336.5818	4.7622
19.0942	0.0897	92.2493	0.324	445.2969	31.6918
20.7033	0.0734	94.8836	1.1385	469.4745	49.1675
22.1967	0.2235	105.531	14.7643	535.3431	2.2152
26.6846	0.1756	126.1454	0.6806	575.9194	5.1323
29.7205	0.3707	140.4718	28.6794	646.7821	42.4502
32.7704	0.0917	148.4698	1.9952	651.7875	3.407
35.6407	0.8824	165.1578	0.0948	653.9693	30.0003
39.9136	1.7157	171.0335	0.26	657.9821	34.1877
41.7248	0.843	179.6959	0.9756	665.3806	2.454
44.5045	0.0591	192.4621	7.9594	666.8598	61.1078
48.7205	0.3268	211.5101	22.0775	669.6604	41.1504
53.8109	0.4737	255.2217	4.0802	671.5053	5.7511
60.18	1.0064	279.9397	15.9509	682.6821	202.4562
66.6266	0.8671	287.923	1.4272	792.9148	235.6582

799.9983	1.0651	1373.8116	20.7284	2301.0966	59.132
885.3288	109.3646	1373.9368	35.5835	2413.1365	193.8206
893.1642	107.054	1381.311	44.0349	2419.5715	1811.4629
1091.0212	372.6066	1875.3965	331.0991	2426.7838	789.7571
1369.8666	6.6427	1934.1908	389.2588	2444.6884	879.2991

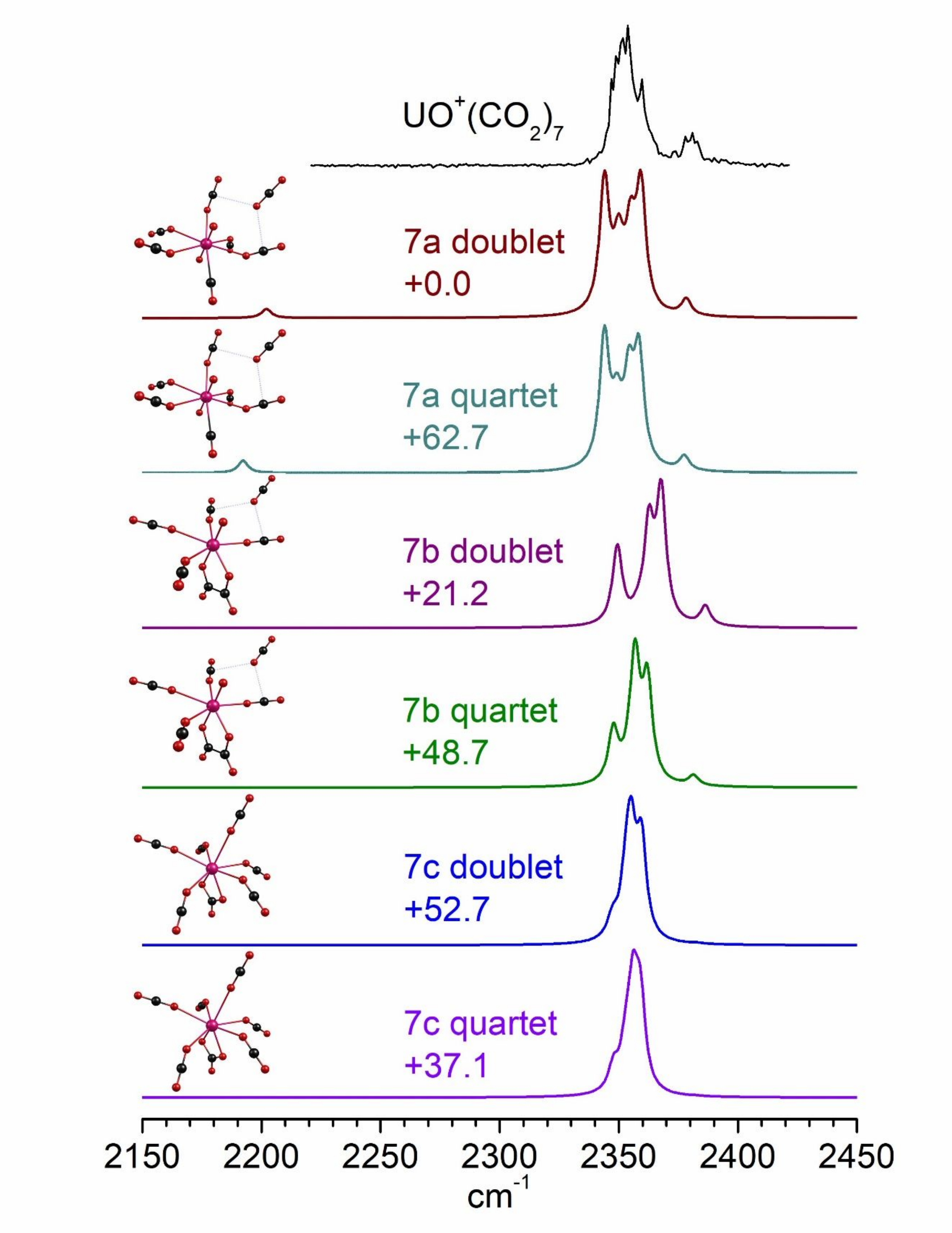

Figure S93. Experimental IR spectrum of $UO^+(CO_2)_7$ compared with simulated spectra for isomers 7a, 7b and 7c doublets and quartets. Relative energies (kcal/mol) are shown next to each spectrum.

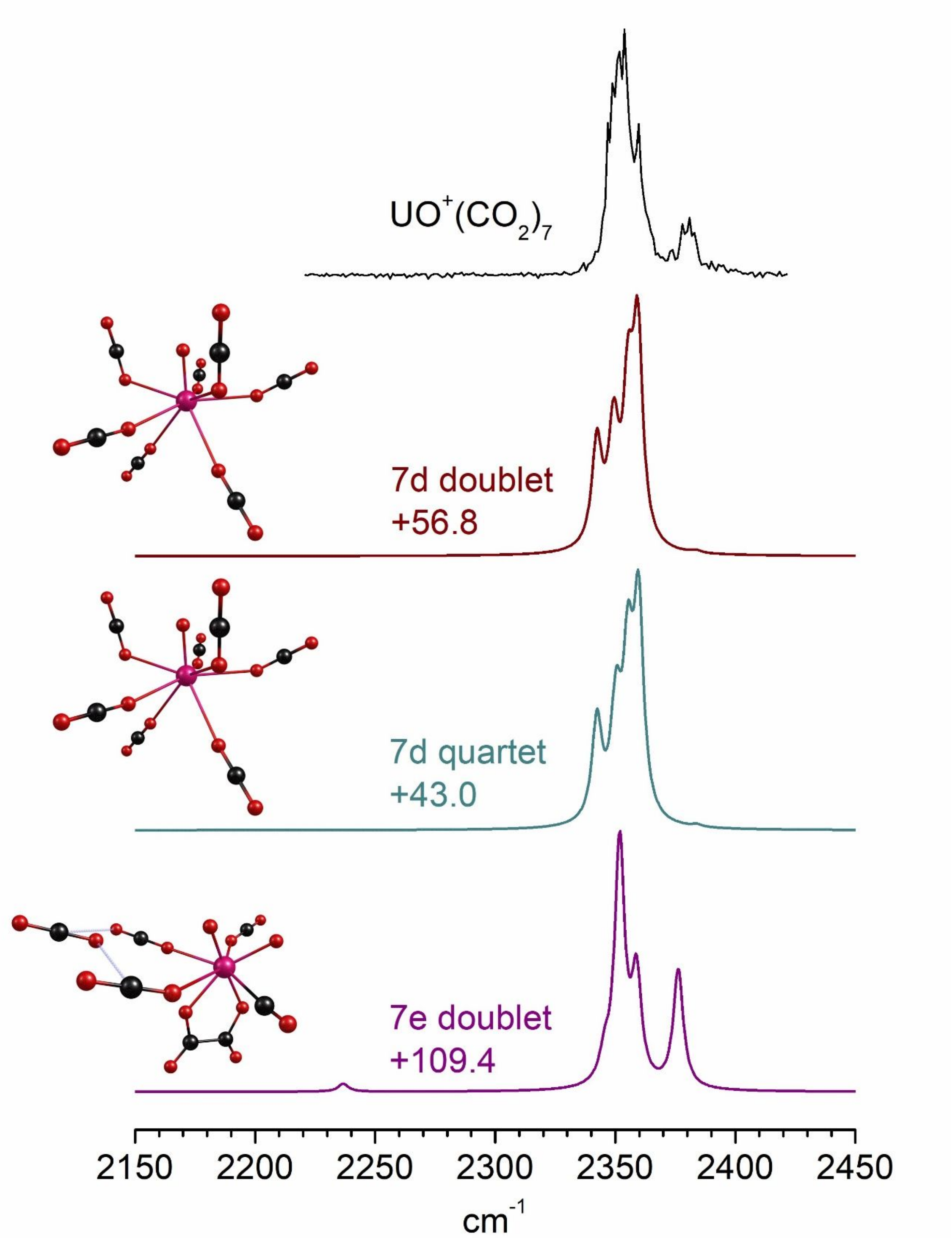

Figure S94. Experimental IR spectrum of $UO^{+}(CO_2)_7$ compared with simulated spectra for isomers 7d and 7e. Relative energies (kcal/mol) are shown next to each spectrum.

Table S117. $UO^+(CO_2)_8$ electronic energy calculated at the B3LYP/cc-pVTZ(-pp) level with Stuttgart/Koeln pseudopotential.

Isomer	2s + 1	Energy (hartree)	Rel. E (kcal/mol)	BDE (CO_2) (kcal/mol)	BDE (CO) (kcal/mol)	BDE (oxalate) (kcal/mol)
8a	2	-2059.188068	+0.0	2.7	2.3	
8b	2	-2059.154574	+21.0	2.8		41.4
8c	2	-2059.098673	+56.1	3.4		
8c	4	-2059.120830	+42.2	3.5		
8d	2	-2059.098171	+56.4	3.0		
8d	4	-2059.120294	+42.5	3.1		
8e	2	-2059.097609	+56.8	2.7		
8e	4	-2059.119623	+42.9	2.7		
8f	2	-2059.097197	+57.0	2.4		
8f	4	-2059.119646	+42.9	2.7		

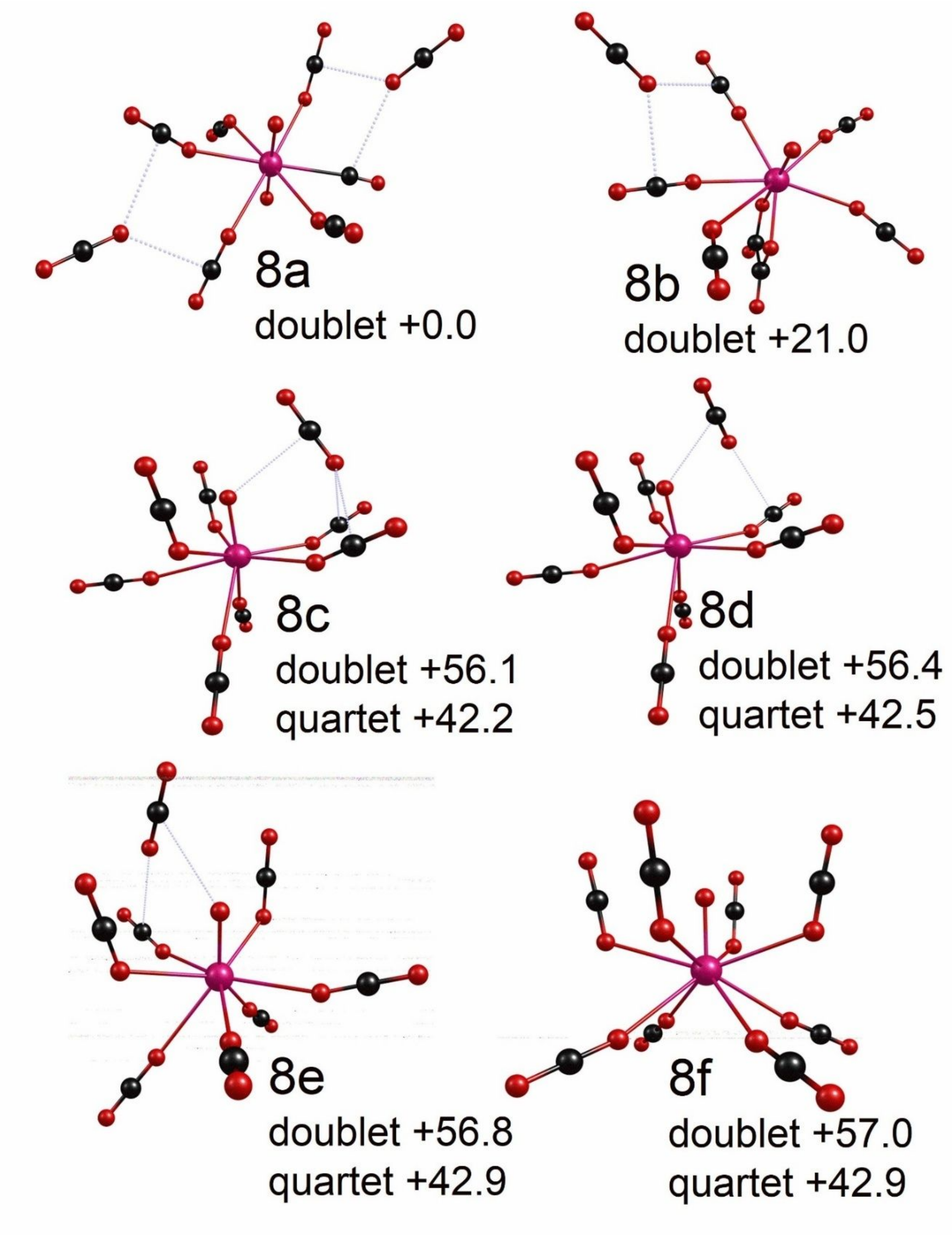

Figure S95. Predicted minimum energy structures of $UO^+(CO_2)_8$ with energy of each spin state in kcal/mol. The lowest energy spin state of each isomer is shown.

Table S118. Cartesian coordinates for the optimized geometry of isomer 8a-doublet $UO^+(CO_2)_8$ followed by its predicted frequencies (cm^{-1}) and IR intensities (km/mol).

Z	x	y	z
92	0.185743000	0.117482000	-0.331558000
8	4.666966000	-0.649014000	0.748826000
6	5.593822000	-1.057893000	1.327461000
8	6.509406000	-1.465701000	1.899282000
8	-0.175583000	2.874069000	-0.178284000
6	-0.487489000	3.727731000	-0.917569000
8	-0.792553000	4.575580000	-1.629515000
8	-2.127836000	-1.324886000	-0.657003000
6	-3.039877000	-1.461037000	-1.378703000
8	-3.933843000	-1.610323000	-2.083653000
8	-0.248712000	0.564271000	-2.023519000
6	2.484006000	-0.783007000	-1.710277000
8	3.333501000	-1.104449000	-2.364764000
8	-2.028408000	1.022167000	1.045617000
6	-2.585227000	1.015877000	2.074970000
8	-3.134703000	1.024155000	3.083606000
8	0.631943000	-0.351118000	1.350379000
8	4.008374000	2.383874000	1.553243000
8	2.440871000	1.657978000	0.005838000
6	3.232668000	2.013546000	0.792032000
8	0.693880000	-4.590077000	0.638251000
8	0.446905000	-2.642496000	-0.597356000
6	0.571846000	-3.622104000	0.032508000
6	-6.097011000	-0.578644000	1.039794000
8	-4.961309000	-0.457803000	0.799556000
8	-7.219495000	-0.698124000	1.277255000

Frequency	Intensity	Frequency	Intensity	Frequency	Intensity
4.3485	0.0194	37.6969	0.1913	91.823	0.8654
7.4185	0.0199	39.7898	0.5256	100.0211	0.2231
8.259	0.0878	41.539	0.0363	118.871	1.0328
11.4553	0.0293	46.8578	0.0612	123.8269	3.4087
18.3111	0.0048	49.0127	0.0884	141.3864	2.4766
19.4854	0.0374	51.2806	0.1795	145.8627	2.0293
24.4138	0.0219	52.253	0.2512	146.5415	22.3928
24.5707	0.0019	62.5931	0.093	151.8012	4.3137
27.6459	0.0398	66.0946	0.0515	152.2235	8.9758
29.358	0.0311	74.0608	0.2608	164.9376	1.5847
31.2107	0.0433	78.5748	0.1539	181.2888	1.7538
33.8271	0.0213	80.2173	0.7738	215.4542	52.1775
37.3029	0.2709	85.8091	0.4212	217.8982	46.4406

249.896	5.7427	665.5343	16.3498	1375.1006	42.6169
260.2953	0.3836	668.7099	19.2183	1375.423	6.4936
651.7016	17.1196	668.7927	60.5748	1377.3259	18.0937
653.6402	49.6851	670.9614	26.8662	2265.5532	111.95
656.8445	53.2506	671.6725	47.279	2414.9328	612.5795
659.2855	16.285	855.4309	0.529	2416.4023	1668.5549
660.1005	10.8745	913.2499	393.723	2418.0002	1664.9962
662.6335	29.3164	1371.0914	6.3035	2420.3461	139.683
663.3433	20.9144	1371.322	6.314	2424.637	713.9692
663.7475	46.4614	1371.9204	27.2462	2428.0968	1171.1739
664.7864	26.204	1373.3226	19.3083	2448.7807	288.7179

Table S119. Cartesian coordinates for the optimized geometry of isomer 8b-doublet $UO^+(CO_2)_8$ followed by its predicted frequencies (cm^{-1}) and IR intensities (km/mol).

Z	x	y	z
92	0.493121000	-0.210499000	-0.129439000
8	2.900472000	-0.020023000	-1.211409000
6	3.696991000	0.670761000	-1.729992000
8	4.483439000	1.329329000	-2.238339000
8	1.287203000	1.644736000	0.633973000
6	1.830689000	1.782168000	1.857344000
8	2.277449000	2.769154000	2.337770000
8	-1.660622000	0.690576000	0.942401000
6	-2.586138000	1.402679000	1.063019000
8	-3.493361000	2.084202000	1.209904000
8	1.226653000	-0.553942000	1.767905000
6	1.801677000	0.407773000	2.607279000
8	2.190893000	0.182901000	3.693741000
8	-1.248905000	-2.203147000	0.275342000
6	-2.147588000	-2.949027000	0.368434000
8	-3.014669000	-3.693038000	0.462643000
8	-0.162623000	-0.043825000	-1.787018000
8	-4.289567000	-0.786520000	-0.100082000
8	-6.524679000	-0.918869000	-0.710808000
6	-5.415026000	-0.853025000	-0.406634000
8	2.897491000	-4.458225000	-1.084417000
6	2.249083000	-3.533006000	-0.891556000
8	1.581148000	-2.589720000	-0.696424000
6	-1.343745000	4.110734000	-1.512975000
8	-1.294132000	5.114964000	-2.078130000
8	-1.391532000	3.092892000	-0.944689000

Frequency	Intensity	Frequency	Intensity	Frequency	Intensity
4.5761	0.1771	44.5231	0.195	127.4259	3.6615
7.193	0.0055	47.8961	0.2685	136.5405	6.2357
8.5809	0.0656	56.5871	0.1301	138.0152	8.5949
14.9898	0.1767	58.12	0.2308	149.3562	17.4219
17.0993	0.9451	64.2349	3.0189	158.0579	7.7091
19.7879	0.0788	69.9076	0.578	161.0657	3.5366
20.3757	0.5497	76.2059	1.095	179.7395	8.6076
23.8238	0.8548	78.5256	0.2769	185.7193	52.3726
26.4479	0.7256	83.5228	0.4028	294.5341	28.4509
33.1634	0.0544	97.4061	0.8932	308.2369	22.2521
34.926	0.2063	101.712	0.3813	336.7331	3.2321
36.178	0.572	104.9419	2.9011	477.3228	51.6002
39.115	1.2043	112.5198	0.0947	544.2727	2.528

574.7447	28.9027	669.6063	22.1636	1380.8345	35.2148
642.6955	46.7807	671.5564	32.9459	1381.1565	60.7428
648.9064	23.2647	739.9102	137.6349	1383.7303	29.6938
650.2093	13.3412	816.6368	0.1253	1859.9102	358.9377
651.1837	24.1405	830.9771	369.8521	1902.0865	439.7435
652.9402	13.0414	898.066	191.2616	2415.0953	889.2806
653.8203	67.4784	930.007	55.5388	2420.2939	1060.6527
656.0436	34.4818	1154.6411	558.0138	2426.0909	390.8809
659.9542	31.8295	1370.1619	8.5908	2430.5092	1352.1947
666.4791	21.4087	1373.1091	5.62	2437.3521	1733.7839
668.1663	44.8927	1374.3279	46.9885	2455.6158	266.9224

Table S120. Cartesian coordinates for the optimized geometry of isomer 8c-doublet $UO^{+}(CO_2)_8$ followed by its predicted frequencies (cm^{-1}) and IR intensities (km/mol).

Z	x	y	z
92	-0.208624000	-0.000014000	0.071018000
8	1.020507000	-0.000159000	-1.350060000
8	-1.983656000	-1.885056000	1.337377000
6	-2.574807000	-2.731738000	1.884824000
8	-3.159501000	-3.559175000	2.431942000
8	4.307371000	-0.000101000	-2.087603000
6	4.009800000	-0.000064000	-0.973660000
8	3.735761000	-0.000024000	0.161550000
8	1.337339000	-1.592668000	1.580009000
6	2.336367000	-2.150396000	1.833227000
8	3.302922000	-2.710530000	2.097343000
8	-2.526583000	0.000180000	-1.346998000
6	-3.423762000	0.000146000	-2.098344000
8	-4.305457000	0.000112000	-2.835350000
8	-1.983435000	1.885320000	1.337279000
8	-3.159221000	3.559637000	2.431605000
6	-2.574556000	2.732102000	1.884605000
6	0.209281000	2.738547000	-2.102774000
8	-0.450081000	2.505740000	-1.159335000
8	0.839070000	3.000939000	-3.025842000
8	-0.450596000	-2.505718000	-1.159328000
6	0.208767000	-2.738648000	-2.102736000
8	0.838548000	-3.001160000	-3.025775000
6	2.336491000	2.150163000	1.833356000
8	3.303032000	2.710290000	2.097541000
8	1.337476000	1.592444000	1.580071000

Frequency	Intensity	Frequency	Intensity	Frequency	Intensity
11.3129	0.0098	39.3281	0.0003	86.2315	0.4165
12.4584	0.0345	45.964	0.2594	89.4487	0.3167
15.1831	0.0176	48.8587	0.995	89.7585	2.0084
16.7273	0.1419	49.9364	1.6315	95.2713	0.4039
16.7493	0.1233	53.9176	1.8097	95.8933	1.1066
20.5559	0.0382	56.3796	0.781	107.0148	0.6465
20.6632	0.3543	56.6129	1.0354	112.5274	0.9384
23.5859	0.0063	62.4853	0.1093	125.4554	14.1381
23.5874	0.0068	63.1032	0.3335	134.0704	22.2958
26.8452	0.224	69.8616	0.1217	140.053	14.292
30.0937	0.2083	71.9628	2.6439	152.0157	8.8681
31.4317	0.1145	76.5987	2.2951	181.1193	8.2132
37.6947	0.1451	84.7726	0.7406	216.3113	11.4631

642.4477	3.1911	658.0561	12.9533	1370.5167	4.9949
643.4455	22.1353	659.3585	46.1416	1371.1933	19.1411
644.5666	18.1213	665.2141	1.4009	1372.6377	4.0271
644.6069	13.3252	666.7206	37.1753	2405.9167	227.0089
645.8554	52.3196	675.9156	28.7408	2406.8544	7.2506
645.9692	64.1754	793.4416	329.2807	2415.5269	205.4832
648.9022	14.6508	1361.2716	3.2729	2417.414	1731.6155
650.6927	19.533	1361.5703	26.244	2418.7259	1012.338
651.5235	64.2024	1367.5328	1.1155	2421.8898	1568.3043
651.7991	103.9093	1367.5704	2.6304	2428.1779	2495.2166
655.0495	64.9156	1369.7682	4.3705	2452.4254	42.1258

Table S121. Cartesian coordinates for the optimized geometry of isomer 8d-doublet $UO^+(CO_2)_8$ followed by its predicted frequencies (cm^{-1}) and IR intensities (km/mol).

Z	x	y	z
92	-0.198983000	-0.038172000	0.090021000
8	1.073642000	-0.525214000	-1.199242000
8	-1.980149000	1.082931000	-1.624504000
6	-2.582815000	1.502762000	-2.535851000
8	-3.175910000	1.915455000	-3.429337000
8	-4.867078000	-1.841462000	1.502069000
6	-3.857241000	-1.385062000	1.189355000
8	-2.832263000	-0.915544000	0.881060000
8	-1.247354000	2.364664000	1.330997000
6	-1.555021000	3.338061000	1.899908000
8	-1.863719000	4.291688000	2.466725000
8	0.937648000	2.379567000	-0.720950000
6	1.765396000	2.405574000	-1.552445000
8	2.571283000	2.464557000	-2.368079000
8	1.622301000	0.592148000	1.945790000
8	3.681796000	1.122544000	2.871376000
6	2.669969000	0.857767000	2.399025000
6	4.083903000	-0.492261000	-1.078904000
8	3.913837000	0.275273000	-0.217487000
8	4.278977000	-1.254098000	-1.923811000
8	-1.306578000	-2.167189000	-1.366006000
6	-0.679867000	-2.613130000	-2.253962000
8	-0.098279000	-3.074972000	-3.128843000
6	0.621798000	-3.273623000	1.961271000
8	1.005450000	-4.286017000	2.344774000
8	0.225104000	-2.240417000	1.578925000

Frequency	Intensity	Frequency	Intensity	Frequency	Intensity
9.9915	0.0946	39.3327	1.1938	86.2931	0.0367
12.6798	0.0993	42.1283	0.1788	90.2431	0.6466
13.976	0.1481	44.9928	0.251	91.8191	3.6575
16.1059	0.1451	46.1723	0.8797	96.0608	0.1314
17.5872	0.0556	51.9658	0.2612	98.169	0.5978
19.3813	0.1465	55.4576	2.8482	101.5739	0.8375
20.1683	0.2285	61.5693	0.0585	112.4186	0.8219
21.2191	0.0839	65.1517	0.8026	120.4401	1.1649
22.6474	0.2412	67.3687	0.6386	131.4957	28.8509
23.9743	0.1815	70.0995	0.454	134.8889	8.7911
26.3912	0.1868	71.113	0.7606	143.9843	18.7433
28.6541	0.0068	74.8094	1.2374	172.4476	3.2904
32.8948	0.2156	79.685	2.0852	221.1815	14.7221

642.3402	18.9184	658.3296	22.389	1371.5452	12.3636
643.3398	3.0786	659.8898	33.0779	1371.7371	6.7702
644.092	22.7797	663.3163	21.4517	1372.6984	6.938
644.4805	6.3076	666.5396	26.1029	2406.2621	17.1254
646.1661	37.9805	674.1159	25.6462	2409.8926	734.5733
647.6287	31.8757	797.0921	346.3924	2415.2621	499.5392
648.7919	23.3458	1360.5502	14.0882	2416.797	875.2092
650.7083	77.0718	1363.3122	16.4522	2417.3174	1022.9504
651.7295	42.493	1367.4011	1.6734	2425.4549	1850.0651
652.8509	88.1093	1368.0996	2.165	2427.8399	2360.1759
653.3009	85.7222	1371.3743	2.5385	2452.9132	22.858

Table S122. Cartesian coordinates for the optimized geometry of isomer 8d-quartet $UO^+(CO_2)_8$ followed by its predicted frequencies (cm^{-1}) and IR intensities (km/mol).

Z	x	y	z
92	0.199421000	-0.040984000	0.080415000
8	-1.105202000	-0.529550000	-1.190496000
8	-0.867496000	2.385303000	-0.757836000
6	-1.699011000	2.421155000	-1.585651000
8	-2.507213000	2.491891000	-2.397692000
8	-3.656862000	1.205884000	2.876573000
6	-2.648884000	0.923520000	2.406216000
8	-1.605310000	0.640385000	1.954383000
8	1.322889000	2.348547000	1.352079000
6	1.639731000	3.329179000	1.903005000
8	1.955409000	4.292195000	2.449482000
8	2.006196000	1.029852000	-1.653023000
6	2.631575000	1.424671000	-2.559965000
8	3.248344000	1.813657000	-3.448145000
8	2.820202000	-0.979767000	0.940182000
8	4.819802000	-1.963956000	1.584441000
6	3.827355000	-1.477554000	1.262046000
6	0.626506000	-2.616097000	-2.248794000
8	1.273259000	-2.175841000	-1.371915000
8	0.028025000	-3.075400000	-3.113257000
8	-0.290300000	-2.228579000	1.596941000
6	-0.748650000	-3.247247000	1.948042000
8	-1.192403000	-4.246345000	2.300118000
6	-4.085604000	-0.404521000	-1.057237000
8	-3.897067000	0.377139000	-0.212323000
8	-4.302876000	-1.178923000	-1.885033000

Frequency	Intensity	Frequency	Intensity	Frequency	Intensity
9.4593	0.1149	38.9147	1.2471	85.8777	0.9666
12.2033	0.1645	43.2857	0.3079	88.72	2.3488
13.5695	0.1418	45.3228	0.1778	89.1079	0.3782
15.932	0.1803	46.9396	1.1178	95.549	0.604
17.3324	0.056	51.9952	0.2225	97.4487	0.6434
18.9528	0.2449	56.2908	2.7046	101.0815	1.7054
19.8542	0.2534	60.6862	0.3274	111.551	0.8648
20.498	0.1617	63.9851	0.9171	121.3982	0.048
22.6032	0.2044	66.3022	0.0966	132.7338	21.6165
23.7745	0.1964	69.1693	0.9295	134.4106	21.9704
26.6763	0.2018	70.2726	0.8403	146.4762	21.1868
28.9173	0.0163	73.0931	1.3445	175.37	2.5431
32.7168	0.2842	79.0931	2.9248	221.0508	17.0691

642.0022	38.1288	660.6734	26.3994	1372.9451	16.1291
646.3135	18.4211	662.5645	21.9374	1373.1118	9.5067
647.2383	10.0712	664.0047	28.7627	1374.0658	9.983
649.4376	29.5669	667.4074	25.8884	2406.1795	23.3379
650.8031	8.9303	674.4123	26.8464	2410.2824	696.5085
651.3407	52.019	788.8093	307.5874	2415.2616	613.3545
651.6488	26.1518	1359.8502	14.139	2417.2237	441.4263
652.8995	16.9458	1363.0346	16.6036	2418.3444	1213.9537
654.7605	90.1434	1370.7795	4.8396	2425.5091	1866.2854
654.8748	87.0678	1371.0024	7.3905	2428.2623	2318.4872
656.2803	59.2737	1371.0805	1.5752	2453.1254	19.1929

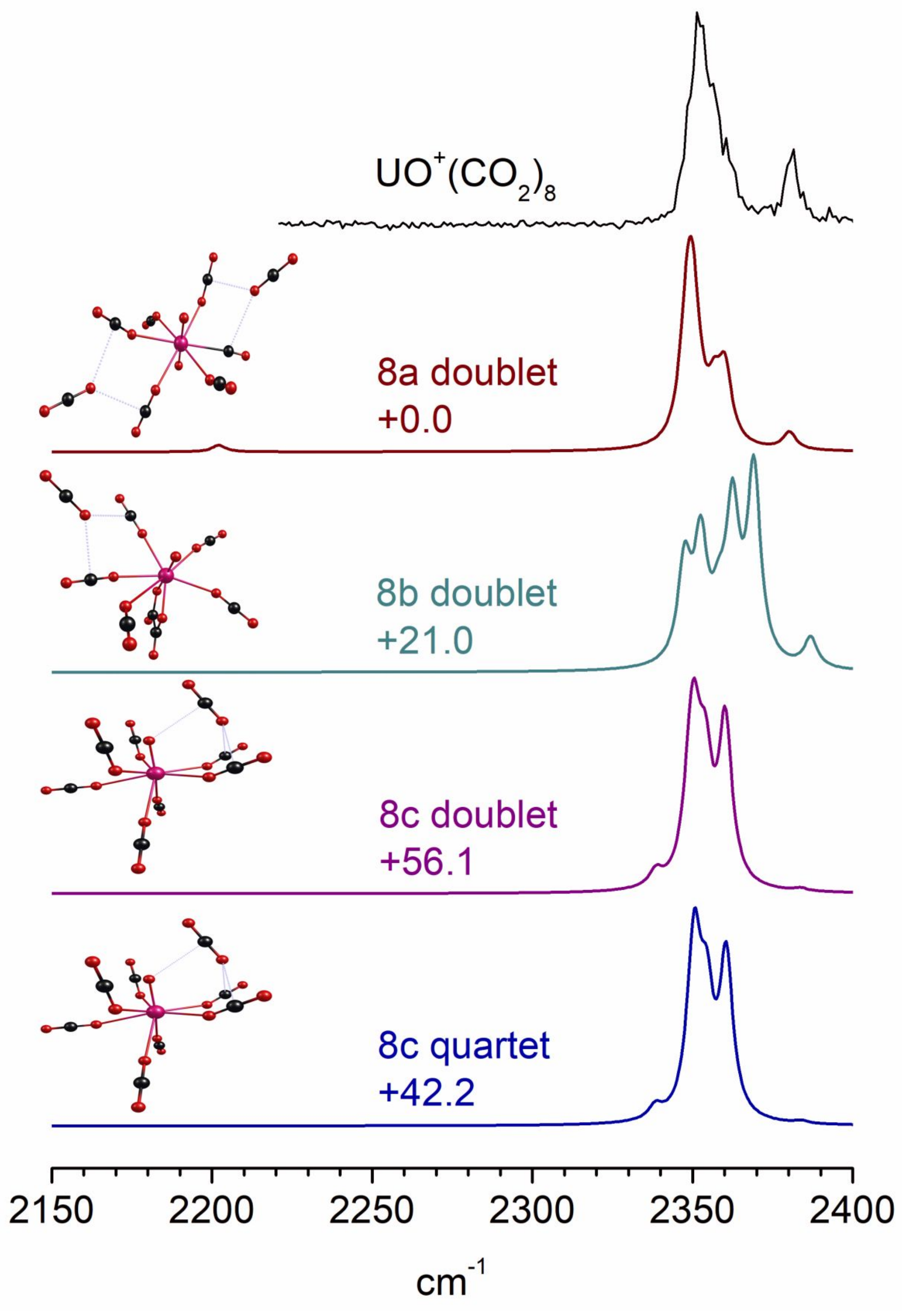

Figure S96. Experimental IR spectrum of $UO^{+}(CO_2)_8$ compared with simulated spectra for isomers 8a, 8b and 8c. Relative energies (kcal/mol) are shown next to each spectrum.

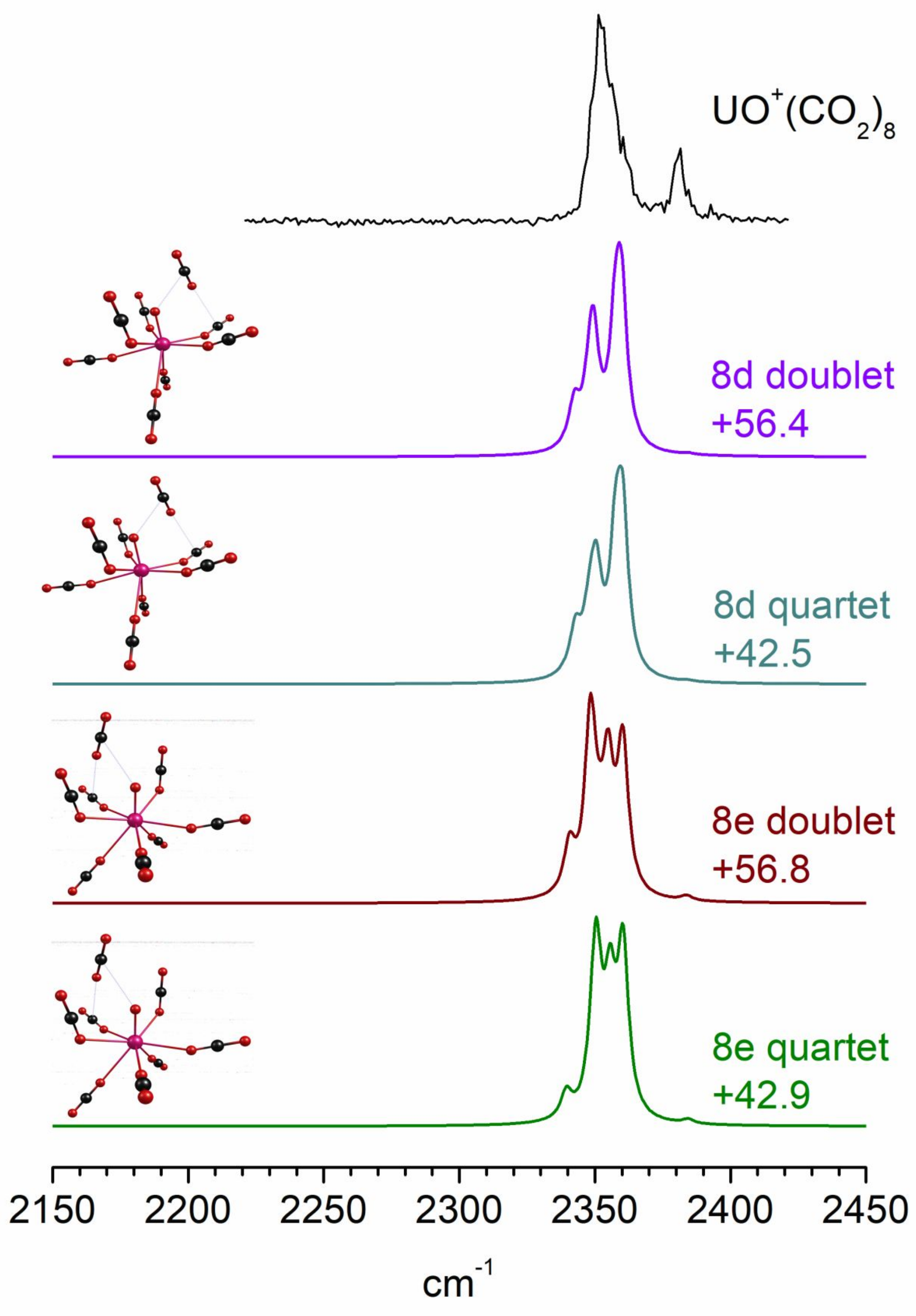

Figure S97. Experimental IR spectrum of $UO^{+}(CO_2)_8$ compared with simulated spectra for isomers 8d and 8e. Relative energies (kcal/mol) are shown next to each spectrum.

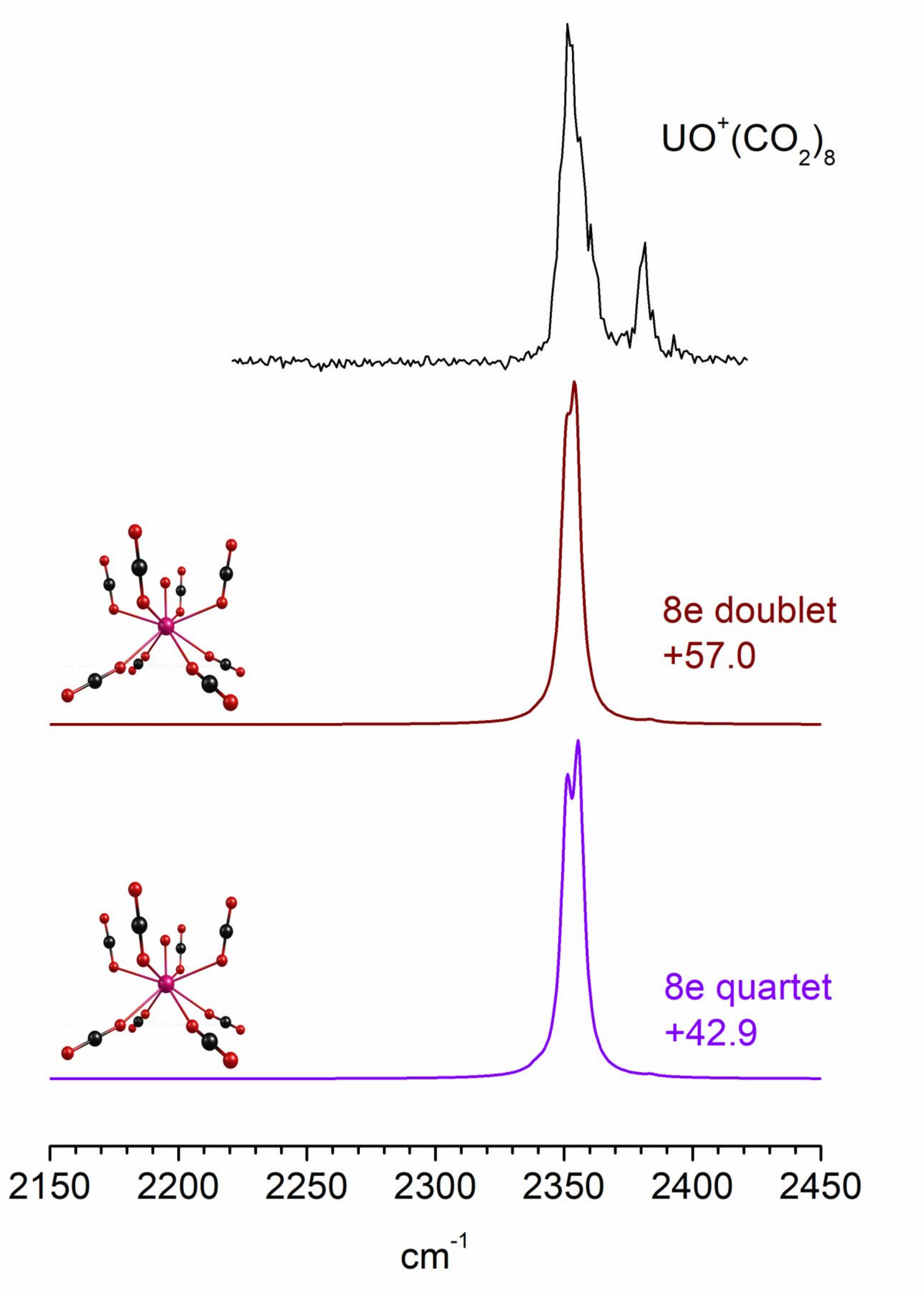

Figure S98. Experimental IR spectrum of $UO^{+}(CO_2)_8$ compared with simulated spectra for isomer 8e. Relative energies (kcal/mol) are shown next to each spectrum.

Table S123. $UO^+(CO_2)_9$ electronic energy calculated at the B3LYP/cc-pVTZ(-pp) level with Stuttgart/Koeln pseudopotential.

Isomer	2s + 1	Energy (hartree)	Rel. E (kcal/mol)	BDE (CO_2) (kcal/mol)	BDE (oxalate) (kcal/mol)
9a	2	-2247.842802	+0.0	3.7	
9b	2	-2247.807154	+22.4	2.3	40.8
9c	2	-2247.751814	+57.1	3.6	
9c	4	-2247.771628	+44.7	1.9	
9d	2	-2247.750568	+57.9	2.2	
9d	4	-2247.772058	+44.4	1.8	
9e	2	-2247.748547	+59.1	0.6	
9e	4	-2247.774021	+43.2	2.7	
9f	4	-2247.773001	+43.8	2.8	
9g	2	-2247.668698	+109.3		

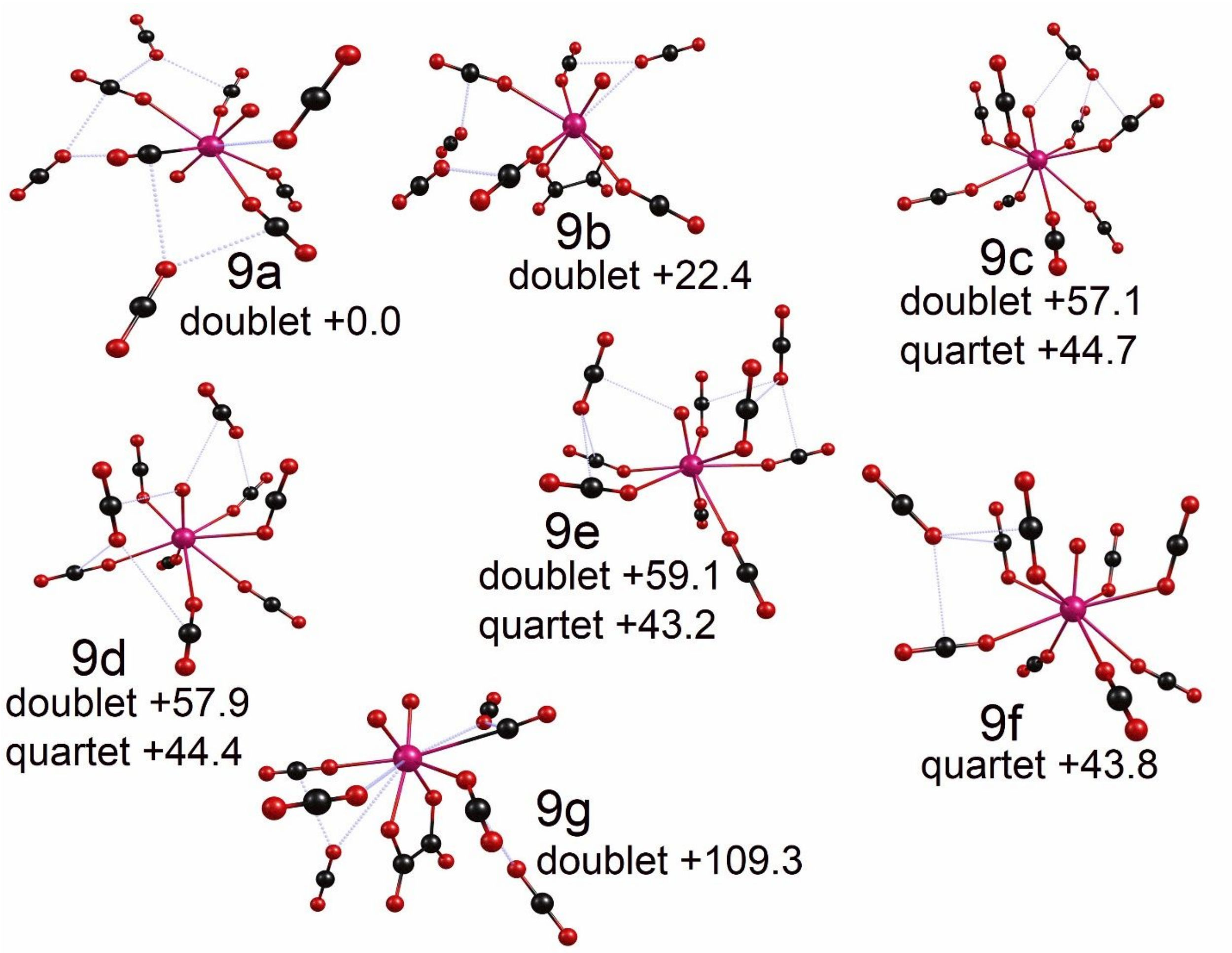

Figure S99. Predicted minimum energy structures of $UO^+(CO_2)_9$ with energy of each spin state in kcal/mol. The lowest energy spin state of each isomer is shown.

Table S124. Cartesian coordinates for the optimized geometry of isomer 9a-doublet $UO^{+}(CO_2)_9$ followed by its predicted frequencies (cm^{-1}) and IR intensities (km/mol).

Z	x	y	z
92	0.068586000	-0.497474000	-0.209241000
8	-2.518709000	-1.079715000	-0.359120000
6	-3.608747000	-1.381697000	-0.055699000
8	-4.678177000	-1.693614000	0.217250000
8	-0.226292000	-2.636594000	-1.832438000
6	-0.415336000	-3.158209000	-2.864463000
8	-0.599577000	-3.680595000	-3.869228000
8	1.777295000	1.269403000	0.803609000
6	2.486277000	2.196592000	0.890112000
8	3.185338000	3.100782000	0.992468000
8	-0.024710000	0.493029000	-1.707006000
6	-1.342236000	1.426366000	1.201348000
8	-1.896799000	2.201557000	1.788509000
8	2.544704000	-1.376598000	-0.768012000
6	3.537842000	-1.964129000	-0.567732000
8	4.509051000	-2.548540000	-0.389461000
8	0.178223000	-1.499866000	1.282884000
8	-6.358118000	2.752398000	-0.678308000
8	-4.457457000	1.459331000	-0.362007000
6	-5.413702000	2.107720000	-0.520633000
8	0.349865000	3.834031000	-0.385206000
8	-0.026924000	5.512222000	-1.942664000
6	0.162171000	4.677908000	-1.167745000
6	6.116727000	0.701584000	0.716806000
8	4.990252000	0.484358000	0.500365000
8	7.229400000	0.916492000	0.930532000
8	-1.632653000	-2.515419000	4.461537000
6	-2.145960000	-1.908971000	3.623750000
8	-2.666232000	-1.294587000	2.780761000

Frequency	Intensity	Frequency	Intensity	Frequency	Intensity
2.2153	0.0185	25.5381	0.0397	49.5832	0.1723
3.688	0.0034	28.1591	0.095	50.1173	0.0677
6.9045	0.0141	31.2446	0.069	51.7607	0.0072
9.0551	0.0057	31.741	0.0772	55.6202	0.1141
10.1684	0.0558	32.1435	0.1351	56.6603	0.1687
10.4281	0.013	34.0814	0.3621	60.4077	0.4223
15.2291	0.0832	37.4585	0.1262	70.924	0.4179
17.198	0.0489	40.8502	0.3686	71.6653	0.5073
20.2926	0.0038	42.5468	0.1484	73.6868	0.9021
20.9923	0.2578	47.0901	0.2674	75.9048	0.0753

84.1705	0.2147	648.0577	47.7238	1370.7626	6.9219
93.8004	0.3032	653.4333	29.3376	1371.9047	5.1314
97.6953	0.4131	654.4653	36.3538	1372.2993	4.4707
103.2557	0.096	656.7257	9.5419	1372.3628	3.8067
110.634	0.9893	657.127	38.038	1377.3587	28.3945
116.5737	0.5574	658.1465	37.3714	1379.1106	36.45
127.189	1.7531	661.1875	21.8041	1381.5708	53.0138
134.5542	1.383	664.5255	36.7354	1382.633	2.9197
143.9171	1.303	667.1313	20.2769	2261.7854	145.7267
145.1941	3.9822	668.3798	6.1601	2414.4305	157.2641
151.2371	7.0481	668.6663	77.7824	2415.8257	2174.1855
156.7802	3.6847	670.2591	37.9645	2416.933	1441.0173
202.4078	58.4883	671.4784	28.0912	2421.2574	587.4759
206.6126	57.324	671.737	9.3447	2423.9278	428.4051
236.5545	2.8346	672.3511	59.0115	2428.9105	733.5902
243.7372	2.6231	859.3114	4.7976	2433.8947	1625.0819
646.1895	40.0054	920.4847	409.3571	2452.235	101.1432

Table S125. Cartesian coordinates for the optimized geometry of isomer 9b-doublet $UO^+(CO_2)_9$ followed by its predicted frequencies (cm^{-1}) and IR intensities (km/mol).

Z	x	y	z
92	0.621610000	0.402834000	-0.311785000
8	0.820997000	-2.150451000	-0.722570000
6	1.400185000	-3.163700000	-0.853487000
8	1.930632000	-4.169225000	-0.983924000
8	1.773739000	-0.167294000	1.425942000
6	1.233310000	-0.470307000	2.619155000
8	1.804891000	-0.785142000	3.608983000
8	1.448665000	2.698817000	0.681237000
6	2.309404000	3.365125000	1.122247000
8	3.137853000	4.028235000	1.552232000
8	-0.682906000	0.073931000	1.258923000
6	-0.326346000	-0.354288000	2.536885000
8	-1.101946000	-0.588802000	3.391454000
8	-3.804376000	1.138170000	-0.522551000
6	-4.722645000	0.782931000	0.103652000
8	-5.628531000	0.427468000	0.722931000
8	1.641677000	0.741267000	-1.742991000
8	-0.969228000	2.424851000	-0.967377000
8	-2.577990000	4.067035000	-1.262573000
6	-1.788842000	3.249498000	-1.113714000
8	-3.325236000	-1.223577000	-2.803571000
6	-2.348548000	-0.851343000	-2.333477000
8	-1.338579000	-0.472384000	-1.875866000
6	-3.984920000	-2.873455000	1.017547000
8	-3.378454000	-2.152930000	0.327986000
8	-4.580676000	-3.589118000	1.698352000
6	5.041921000	-1.709761000	-0.918240000
8	3.876232000	-1.701116000	-0.873626000
8	6.194575000	-1.713353000	-0.962890000

Frequency	Intensity	Frequency	Intensity	Frequency	Intensity
3.2403	0.2348	27.8097	0.6177	58.332	0.1559
5.4881	0.136	32.6627	0.197	59.4907	1.4114
8.5152	0.142	34.9671	0.1915	63.4821	0.7466
12.7345	0.0876	37.1165	0.0908	77.1833	1.4034
15.6741	0.3818	38.8296	1.7567	81.1897	0.0238
19.0624	0.3381	41.2968	0.3652	82.0304	0.7013
20.5641	0.4897	47.0326	0.372	84.9535	0.4785
23.0017	1.3585	48.9231	1.0815	96.3243	0.6106
23.9005	0.011	49.8258	0.8073	101.9103	0.6562
24.8067	0.2902	55.2692	0.4265	108.8447	2.1662

114.4779	0.0405	648.3776	64.7659	1160.3674	518.8815
132.1247	0.1365	651.1074	14.1943	1370.7691	4.7273
135.8983	6.0835	652.1841	23.1867	1372.1018	6.3205
138.4853	6.2883	653.584	54.1164	1373.2013	6.5301
149.5259	19.1682	655.7063	24.9148	1376.2783	50.3754
154.8458	2.4289	658.2112	27.9911	1379.2037	52.8958
159.8871	15.3672	665.2772	34.2169	1382.2993	33.9728
176.4791	13.9256	666.6064	25.3276	1384.3703	34.2172
183.852	41.7694	667.7865	45.2221	1857.9964	336.6925
293.4987	31.044	669.6438	12.9454	1893.5882	399.6159
308.2546	23.4313	670.4603	66.8342	2412.7379	165.5148
336.6248	2.4604	672.3553	12.7872	2417.5472	2061.8791
477.2097	49.736	746.8686	112.7355	2420.3939	536.2798
547.2223	3.8185	818.5589	0.0324	2427.5038	202.1317
574.3149	29.2168	834.8621	338.1455	2432.9889	1338.3085
644.5027	18.1397	903.2131	216.0761	2436.3438	1804.7869
646.9009	29.4982	938.5096	54.7326	2456.3937	225.2558

Table S126. Cartesian coordinates for the optimized geometry of isomer 9c-doublet $UO^{+}(CO_2)_9$ followed by its predicted frequencies (cm^{-1}) and IR intensities (km/mol).

Z	x	y	z
92	0.324348000	0.402239000	0.000015000
8	-0.163173000	-1.414594000	0.000321000
8	-0.949991000	-0.080367000	-2.461960000
6	-1.453668000	-1.129965000	-2.607764000
8	-1.961091000	-2.144886000	-2.786795000
8	-4.368282000	2.106840000	-0.000164000
6	-3.248603000	1.849808000	-0.000007000
8	-2.103628000	1.607874000	0.000156000
8	0.447412000	2.516336000	-1.937498000
6	0.541452000	3.307338000	-2.792633000
8	0.639512000	4.093213000	-3.628586000
8	2.425450000	-0.076904000	-1.599500000
6	3.158933000	-0.792159000	-2.168319000
8	3.884512000	-1.473779000	-2.739856000
8	0.448169000	2.516445000	1.937198000
8	0.640146000	4.093275000	3.628344000
6	0.542148000	3.307423000	2.792363000
6	3.159283000	-0.792138000	2.167794000
8	2.425931000	-0.076764000	1.598954000
8	3.884738000	-1.473871000	2.739354000
8	-0.949697000	-0.079811000	2.462323000
6	-1.453475000	-1.129341000	2.608274000
8	-1.961011000	-2.144180000	2.787449000
6	-4.309023000	-2.136178000	0.000108000
8	-3.776114000	-1.098233000	-0.000122000
8	-4.837936000	-3.161415000	0.000337000
8	0.962287000	-4.547724000	0.000207000
6	1.749329000	-3.704672000	0.000009000
8	2.567985000	-2.872290000	-0.000198000

Frequency	Intensity	Frequency	Intensity	Frequency	Intensity
8.1094	0.1081	24.7936	0.0558	53.9552	0.4128
11.1034	0.0076	31.137	0.0838	57.1238	0.0008
13.3161	0.0001	31.1996	0.1698	60.0468	1.1578
14.9323	0.0334	33.0099	0	60.9972	1.1017
15.2719	0.064	34.3384	0.048	62.5849	0.1137
17.6352	0.137	39.9795	0.5477	63.8956	0.3194
19.1141	0.0843	44.6107	1.1192	70.7306	0.1256
22.4238	0.401	44.6752	0.0028	75.4099	2.5845
24.0001	0.1538	50.9469	1.7728	75.6124	0.9238
24.1835	0.0043	51.8773	0.4369	80.3486	3.4224

87.5811	0.6101	643.9782	28.2855	1362.0677	20.8553
88.491	1.9283	645.6725	51.9022	1367.3067	0.618
93.1377	0.2731	645.8512	47.5233	1367.3865	2.4003
95.852	0.3566	647.9093	24.8533	1369.7558	3.6489
99.045	0.1738	651.6385	93.2178	1370.8968	5.6616
99.4832	1.0895	651.8466	3.0094	1371.5571	18.7666
108.1823	0.6452	653.3604	68.31	1372.1074	5.3083
116.0626	0.848	655.6733	61.7755	1374.4326	8.0966
126.182	17.6491	657.401	4.6646	2404.9021	228.3367
134.2044	14.7396	658.6045	54.698	2404.9944	2.8632
141.1567	15.4994	664.0754	16.7744	2414.4095	227.0872
153.5051	10.4999	666.0186	69.2245	2415.7384	157.9651
184.9867	11.9791	666.8488	0.3993	2417.4898	2681.6635
216.2802	11.6999	671.0117	24.4263	2421.0987	718.7712
641.2746	16.6474	676.1307	31.1585	2422.0998	1565.0902
641.5721	30.2727	791.9033	333.0507	2429.1779	2269.203
643.1839	31.368	1361.8139	1.3533	2452.8567	78.4552

Table S127. Cartesian coordinates for the optimized geometry of isomer 9c-quartet $UO^+(CO_2)_9$ followed by its predicted frequencies (cm^{-1}) and IR intensities (km/mol).

Z	x	y	z
92	0.247438000	-0.002886000	0.004531000
8	-1.257712000	-0.888367000	0.741463000
8	-3.840560000	0.368311000	-0.309401000
6	-4.154711000	-0.483462000	0.423979000
8	-4.499046000	-1.317381000	1.143271000
8	-3.206841000	3.360914000	0.802700000
6	-2.233567000	2.808999000	0.543731000
8	-1.225738000	2.271543000	0.285412000
8	-1.217441000	0.035299000	-2.297230000
6	-2.229184000	-0.195302000	-2.840225000
8	-3.206672000	-0.422043000	-3.398772000
8	0.296307000	-2.877384000	-0.624858000
6	-0.624651000	-3.471602000	-0.204709000
8	-1.509124000	-4.092063000	0.185174000
8	1.897278000	-0.939526000	-2.143625000
8	2.875118000	-2.450670000	-3.607433000
6	2.385982000	-1.704897000	-2.879893000
6	3.047709000	-2.029605000	1.777333000
8	2.345862000	-1.317922000	1.169508000
8	3.742487000	-2.725293000	2.372575000
8	0.234757000	0.258874000	2.909103000
6	-0.682043000	-0.250815000	3.436380000
8	-1.562871000	-0.732853000	3.993768000
6	2.397554000	2.642294000	2.085069000
8	2.889330000	3.276255000	2.910519000
8	1.904614000	2.002759000	1.239924000
6	1.479619000	2.998889000	-2.614559000
8	0.942946000	2.222126000	-1.928015000
8	2.011740000	3.764735000	-3.291525000

Frequency	Intensity	Frequency	Intensity	Frequency	Intensity
7.1086	0.0323	22.9588	0.1054	56.6167	0.0209
8.1347	0.0527	29.8222	0.9167	57.0578	0.1126
11.6994	0.1851	31.4593	0.0031	62.8178	1.5408
13.461	0.3393	35.1095	0.0646	64.5745	4.5555
15.693	0.0677	38.134	0.1098	66.4077	0.5953
16.2168	0.3156	40.994	0.6855	68.099	1.3592
19.0669	0.0383	43.4492	0.42	69.3514	2.0458
19.2639	0.0605	48.0433	0.084	71.7487	0.0818
20.9139	0.0797	53.0513	0.5658	75.7308	2.3986
21.534	0.0082	53.9777	0.3645	84.6029	1.3223

88.9258	3.2091	651.5946	49.2236	1369.4475	3.692
91.5348	0.7296	652.0027	20.4839	1369.5923	5.8627
97.4184	0.5733	653.1247	32.1042	1369.8211	4.9642
98.694	2.4427	655.2452	4.0326	1372.755	14.786
110.2632	1.4383	655.2822	26.0649	1373.2698	8.9084
113.9247	23.3334	657.6459	20.5138	1374.1264	25.1091
117.1029	6.7973	658.7428	69.9098	1374.6278	0.9381
118.6302	1.0648	660.461	15.1349	2402.3075	38.0263
130.2351	6.2378	661.5701	19.0172	2404.0829	0.2874
131.302	13.8712	662.1339	107.1509	2413.4492	91.1688
147.4245	2.5389	664.8395	23.7563	2415.3668	230.6221
199.0175	16.8996	668.5974	7.8163	2418.1023	2828.9172
226.0029	22.3416	671.5297	26.4848	2419.5778	315.4225
636.6953	49.7889	677.2679	34.4756	2423.2783	1793.8428
637.8166	65.7297	782.508	295.6805	2428.8683	2345.1622
646.163	70.2385	1360.5568	5.0971	2454.0542	31.5022
648.1254	23.8709	1361.0484	16.9351		

Table S128. Cartesian coordinates for the optimized geometry of isomer 9d-quartet $UO^+(CO_2)_9$ followed by its predicted frequencies (cm^{-1}) and IR intensities (km/mol).

Z	x	y	z
92	-0.334059000	-0.012057000	0.000290000
8	1.225014000	-1.093281000	0.000069000
8	-2.211446000	-0.798761000	-1.914168000
6	-2.904184000	-1.461243000	-2.584852000
8	-3.589952000	-2.106630000	-3.244607000
8	-3.585894000	-2.099179000	3.254991000
6	-2.900853000	-1.456636000	2.591715000
8	-2.208853000	-0.797060000	1.917426000
8	-1.284900000	2.225090000	-1.613703000
6	-1.582006000	2.956284000	-2.474627000
8	-1.879128000	3.678880000	-3.320617000
8	0.700305000	0.105918000	-2.612009000
6	1.720417000	-0.439611000	-2.808892000
8	2.718348000	-0.959281000	-3.039200000
8	1.413746000	2.107135000	-0.001779000
8	3.365144000	3.360043000	-0.002970000
6	2.407548000	2.725142000	-0.002362000
6	4.275921000	-0.864894000	-0.000184000
8	4.055225000	0.279144000	0.000808000
8	4.519264000	-1.994045000	-0.001179000
8	-1.071935000	-2.812413000	0.001276000
6	-0.172533000	-3.568395000	0.001409000
8	0.675162000	-4.342026000	0.001565000
6	1.722990000	-0.436265000	2.808358000
8	2.720773000	-0.956099000	3.038948000
8	0.703023000	0.109415000	2.611172000
6	-1.580975000	2.963536000	2.468207000
8	-1.877940000	3.691515000	3.309628000
8	-1.284021000	2.226854000	1.611934000

Frequency	Intensity	Frequency	Intensity	Frequency	Intensity
7.6132	0.1053	25.2607	0.3556	57.8093	0.3055
10.8924	0.0004	27.0602	0.0687	61.1701	1.6856
13.5692	0.2496	29.8311	0.0801	63.9542	1.3511
14.6842	0.0631	35.4248	0.0183	64.6947	0.8583
15.8502	0.412	38.3849	0.2398	65.7287	1.1544
15.985	0.0008	39.2421	0.0425	70.2947	0.1476
18.0183	0.0712	43.4523	0.2549	72.5135	1.4762
18.8956	0.0338	43.5293	0.2882	74.6466	0.0066
19.995	0.0948	46.5826	0.4835	81.5174	0.7466
23.6575	0.1447	53.3204	0.1697	81.8711	5.5281

88.1854	0.4312	645.4279	22.2697	1362.1556	2.3769
90.2637	2.2596	651.4726	4.1573	1362.552	26.1327
93.0957	3.7827	653.4684	37.3538	1371.5862	2.9751
96.7254	1.2192	654.416	6.0549	1371.8042	11.3543
97.1776	0.0461	655.1692	111.3504	1372.5274	2.4449
102.0539	1.297	656.3137	5.3937	1373.1855	8.4637
112.034	8.2911	657.8601	69.0946	1373.5165	8.0488
118.6493	2.3406	659.443	40.9441	1375.2674	10.7324
120.0735	0.1694	659.7669	73.3418	2404.2116	19.4375
131.0201	35.6199	660.008	52.9069	2405.5072	9.7785
133.7996	13.7952	662.2246	15.6316	2408.5125	441.5766
168.0795	0.3277	665.0765	1.8168	2415.1249	100.7965
216.0719	15.9196	666.4796	44.0086	2418.5956	224.5345
224.2533	21.1309	669.9674	29.8566	2419.3701	2746.649
637.8836	49.1356	672.7305	27.9509	2423.8855	1726.6763
643.3768	8.7557	777.1012	268.4661	2427.5298	2368.8527
644.2262	80.6875	1360.1615	9.9854	2453.9696	24.8079

Table S129. Cartesian coordinates for the optimized geometry of isomer 9f-quartet $UO^+(CO_2)_9$ followed by its predicted frequencies (cm^{-1}) and IR intensities (km/mol).

Z	x	y	z
92	-0.434321000	0.040643000	-0.093185000
8	0.298381000	0.070953000	1.657725000
8	-1.268292000	2.381280000	-1.443479000
6	-1.459517000	3.439478000	-1.901499000
8	-1.649677000	4.478135000	-2.358736000
8	6.352861000	0.125606000	0.650447000
6	5.259310000	0.115526000	0.285532000
8	4.152176000	0.105228000	-0.086330000
8	1.407374000	2.155819000	-0.026188000
6	2.071361000	2.287427000	0.932891000
8	2.739132000	2.452204000	1.852052000
8	-1.850583000	-1.968234000	1.319514000
6	-1.585533000	-2.230125000	2.431463000
8	-1.344903000	-2.508310000	3.519740000
8	-0.975036000	-2.259552000	-1.669498000
8	-0.951023000	-4.411285000	-2.535239000
6	-0.961044000	-3.344787000	-2.102414000
6	2.147944000	-2.133602000	1.073925000
8	1.461607000	-2.111199000	0.123460000
8	2.840052000	-2.188441000	1.989549000
8	-2.100564000	1.722776000	1.458746000
6	-1.826162000	1.894244000	2.587100000
8	-1.588481000	2.085043000	3.694108000
6	2.448453000	0.006329000	-2.735502000
8	3.423063000	0.064938000	-3.343128000
8	1.448417000	-0.053620000	-2.132538000
6	-4.359377000	-0.231540000	-0.734871000
8	-3.195255000	-0.132223000	-0.733784000
8	-5.506134000	-0.328724000	-0.742261000

Frequency	Intensity	Frequency	Intensity	Frequency	Intensity
7.4921	0.0377	26.1571	0.1098	50.5761	0.4269
9.6723	0.0032	29.6865	0.0863	51.9468	2.4999
13.7694	0.0594	29.9261	0.1145	59.2529	0.3689
15.5547	0.2258	33.5585	0.1964	64.6563	0.3201
16.4644	0.6338	36.1974	0.2324	70.4861	0.4702
16.917	0.1786	38.3969	0.0367	74.1274	1.3346
17.7116	0.0593	40.904	0.347	76.0858	0.4469
18.2696	0.1979	46.1192	0.2213	78.4282	3.1998
21.2129	0.0159	47.2087	0.447	79.1657	0.1552
24.9119	0.1726	48.3226	2.3005	80.5127	1.2438

86.283	4.7994	649.434	21.5835	1362.3966	15.9753
87.9669	1.4168	649.8254	31.8305	1363.6707	9.5243
93.486	1.9639	651.7731	40.8942	1364.8138	21.9713
97.339	0.696	654.2696	3.2156	1370.0327	7.0081
103.1487	0.1194	655.9576	10.7968	1372.3171	2.3197
105.7015	1.5562	657.6574	73.6293	1372.4209	8.2138
107.1583	0.7635	657.7589	77.7574	1372.9569	8.8704
113.7483	0.3705	659.1501	72.9897	1374.3374	6.4581
122.9683	31.8369	659.9008	4.2207	2401.1892	11.3267
127.4533	33.8586	660.2486	0.6116	2405.6928	22.3461
152.8977	1.3432	665.1521	73.2046	2408.3598	28.0083
166.6118	0.8704	667.4369	69.653	2414.5161	617.4741
212.8931	17.1033	668.6353	12.9599	2417.5192	623.9816
225.0484	18.4898	671.1427	3.082	2418.3232	3056.1485
643.3895	18.0937	671.7611	27.4831	2422.9557	1814.1584
644.5548	36.4327	776.7283	264.4944	2425.0943	1536.8482
645.2621	64.9347	1362.0222	3.6268	2452.7168	75.6814

Table S130. Cartesian coordinates for the optimized geometry of isomer 9g-doublet $UO^+(CO_2)_9$ followed by its predicted frequencies (cm^{-1}) and IR intensities (km/mol).

Z	x	y	z
92	-0.049514000	-0.268493000	-0.926134000
8	2.141614000	-1.552174000	-0.079695000
6	3.153597000	-1.529436000	0.513560000
8	4.138876000	-1.537405000	1.097746000
8	0.881805000	0.965933000	0.553482000
6	0.334067000	1.207102000	1.764267000
8	0.780840000	1.884065000	2.624247000
8	-1.890587000	3.402097000	1.059515000
6	-2.243056000	4.180535000	1.856811000
8	-2.591972000	4.948642000	2.641200000
8	-1.276333000	-0.235428000	0.677360000
6	-1.023362000	0.447228000	1.894204000
8	-1.746627000	0.412096000	2.814468000
8	-1.413338000	-0.971594000	-2.247509000
6	-0.285164000	-3.039733000	-0.453014000
8	-0.411893000	-4.143296000	-0.328625000
8	0.962831000	-0.182057000	-2.389149000
8	-1.985960000	4.013422000	-2.145274000
8	-1.030916000	1.985716000	-1.558289000
6	-1.516169000	3.013448000	-1.849246000
8	5.899350000	1.887060000	-1.978955000
8	4.098619000	0.642773000	-1.208965000
6	5.005146000	1.266781000	-1.594722000
8	-3.351986000	-2.522307000	-0.479014000
8	-5.565055000	-3.147216000	-0.789305000
6	-4.463811000	-2.837501000	-0.635281000
6	1.349308000	-1.742635000	4.102908000
8	1.494632000	-1.930335000	5.229837000
8	1.202587000	-1.556669000	2.957849000

Frequency	Intensity	Frequency	Intensity	Frequency	Intensity
6.5712	0.0173	32.7221	0.8414	53.6041	0.6351
7.7753	0.0735	34.7076	0.3308	59.0672	0.4476
8.9032	0.0555	35.0024	0.1219	63.7189	0.0817
11.1822	0.0414	36.9969	0.162	70.5536	0.3851
13.2955	0.2576	38.0768	0.8553	73.8868	0.0544
15.9904	0.0697	39.8727	0.2072	78.2231	0.4875
19.6365	0.0053	40.9318	2.0062	79.3725	0.6997
20.8072	0.0177	44.797	0.5683	88.6805	0.959
24.7941	0.2181	47.143	0.7023	97.4343	3.5784
28.0632	0.0221	50.4561	0.9335	107.1517	1.3447

115.842	12.122	576.9558	12.1064	905.5708	46.3035
127.3159	0.2523	637.6571	32.4528	1117.8088	409.9098
142.958	7.7685	644.17	44.8408	1369.3365	9.374
155.2227	4.043	647.0528	28.9704	1370.6169	9.3272
158.6741	1.4965	658.9724	17.4422	1371.8109	7.5483
162.3971	15.4466	667.0858	20.7406	1372.5209	5.4092
189.1225	1.4033	667.3483	11.9458	1376.6921	44.5954
194.8585	16.9074	667.6552	24.9106	1388.8754	58.5521
206.5394	17.114	668.1564	39.9286	1868.6576	313.8936
234.2436	1.2872	669.5097	22.5032	1924.3063	352.9675
279.5953	14.6413	669.8505	1.4515	2285.9287	66.0064
291.5288	2.6406	669.9611	14.7707	2413.9887	1119.6074
311.5937	17.0318	670.538	72.1035	2414.7902	1230.2429
337.0006	6.655	703.5248	181.3962	2416.5523	1055.9678
446.0443	38.4056	800.1425	262.1772	2422.0278	371.5319
473.2983	74.7794	802.0201	0.2875	2432.7917	981.718
541.5831	1.5548	883.2879	165.3206	2450.0513	742.5239

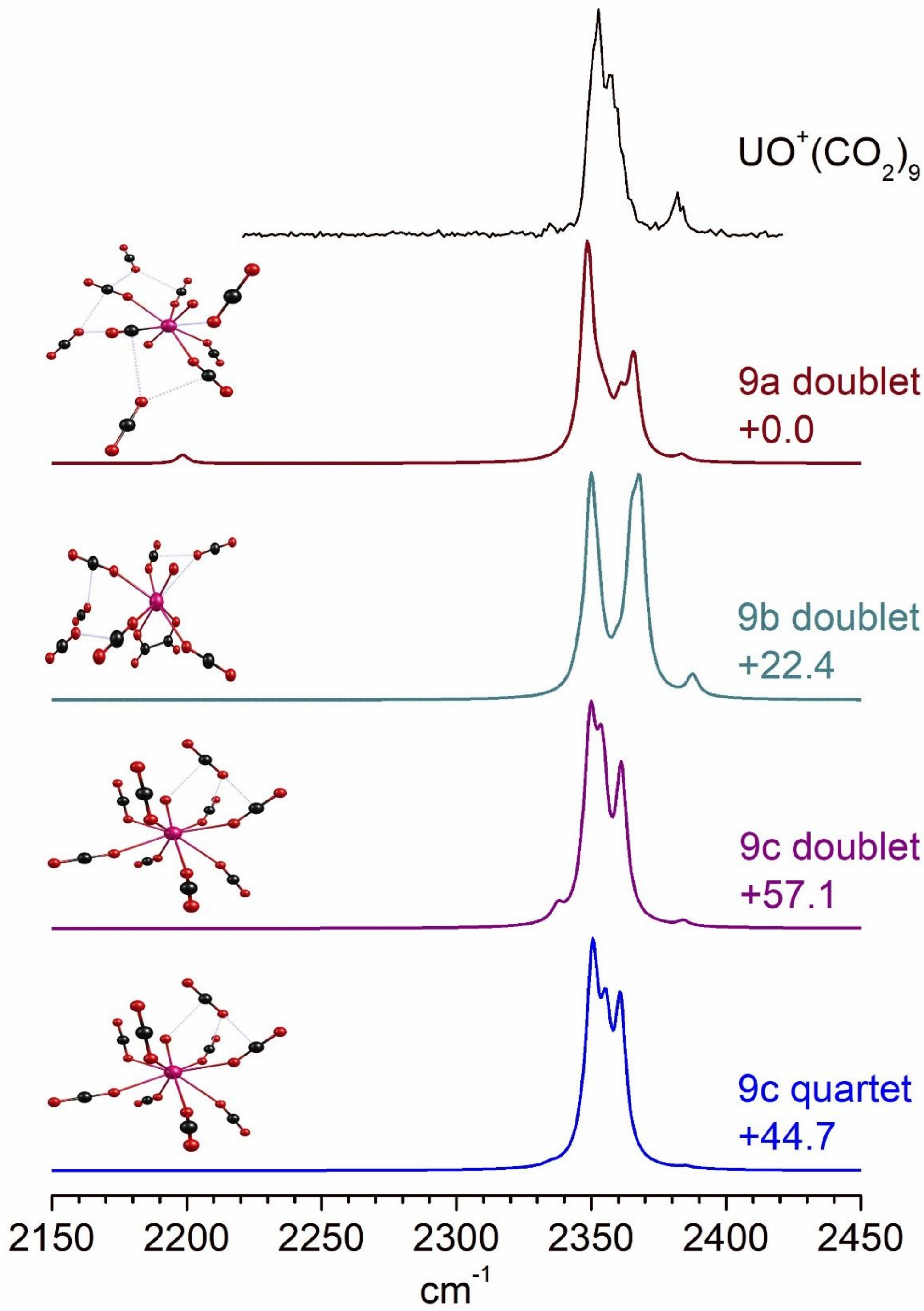

Figure S100. Experimental IR spectrum of $UO^{+}(CO_2)_9$ compared with simulated spectra for isomers 9a, 9b and 9c. Relative energies (kcal/mol) are shown next to each spectrum.

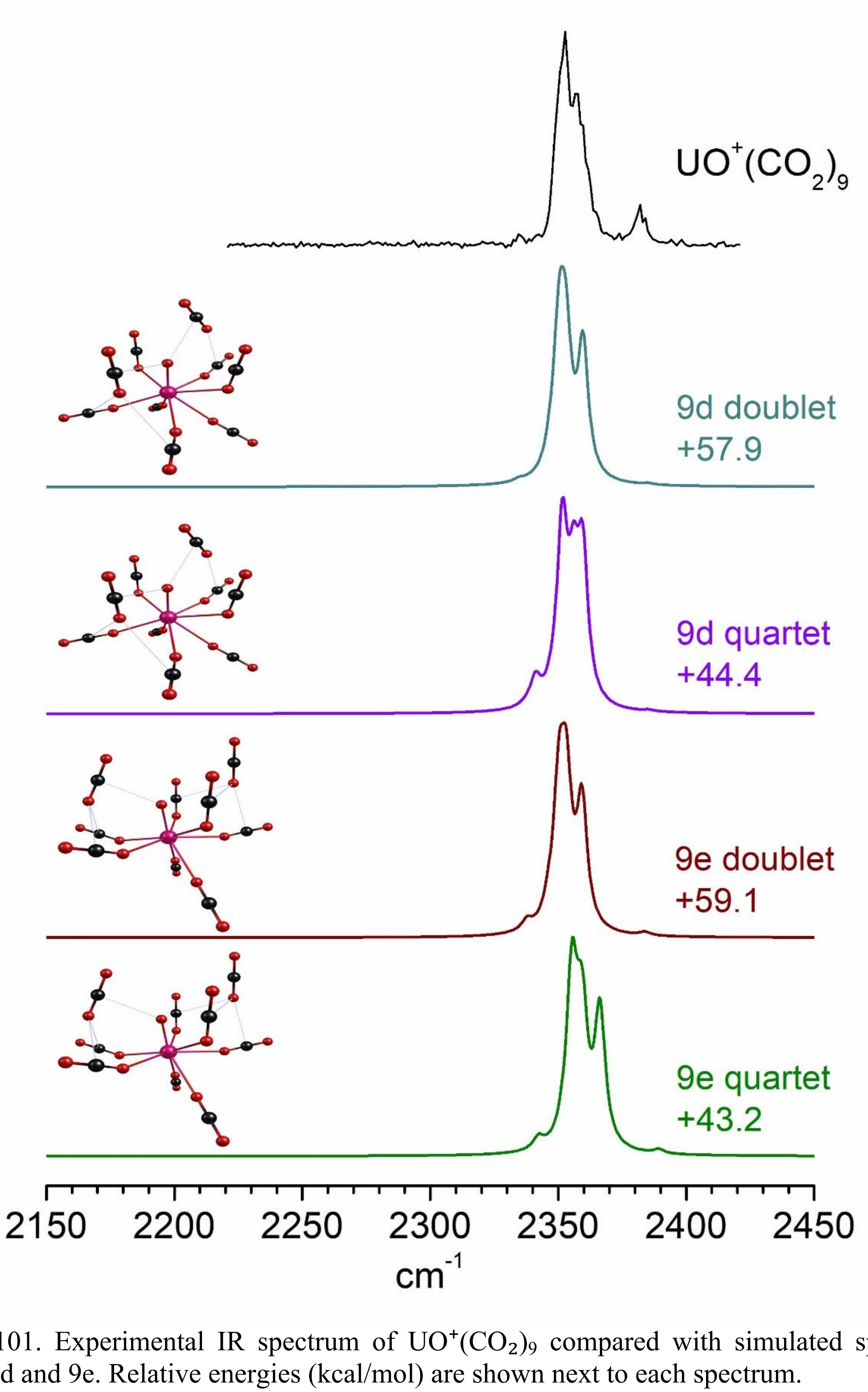

Figure S101. Experimental IR spectrum of $UO^{+}(CO_2)_9$ compared with simulated spectra for isomers 9d and 9e. Relative energies (kcal/mol) are shown next to each spectrum.

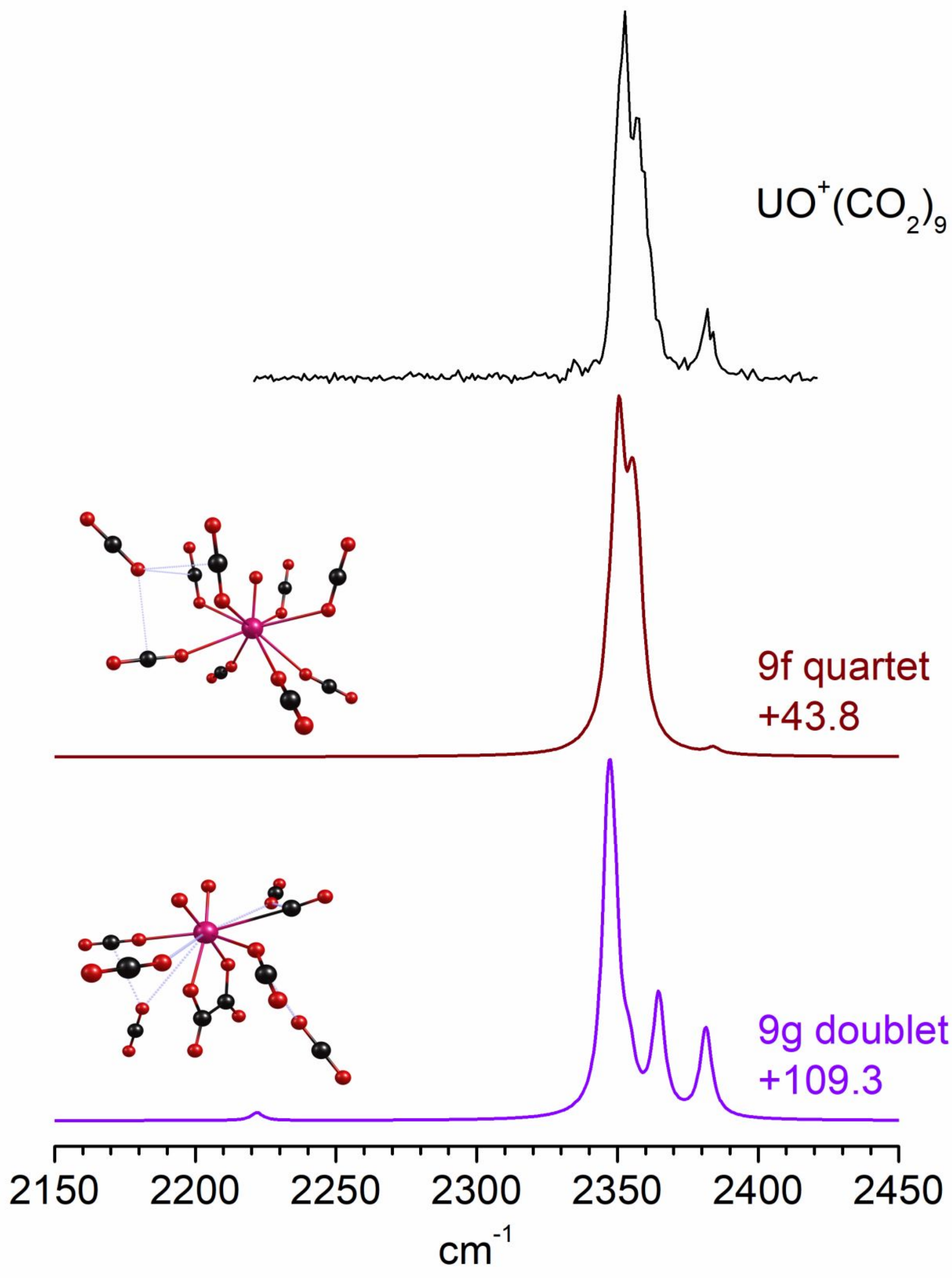

Figure S102. Experimental IR spectrum of $UO^{+}(CO_2)_9$ compared with simulated spectra for isomers 9f and 9g. Relative energies (kcal/mol) are shown next to each spectrum.

Table S131. UO^{2+} electronic energy calculated at the B3LYP/cc-pVTZ(-pp) level with Stuttgart/Koeln pseudopotential.

2s + 1	E (hartree)	Relative E (kcal/mol)
1	-549.323961	+9.8
3	-549.339592	+0.0
5	-549.241075	+61.8

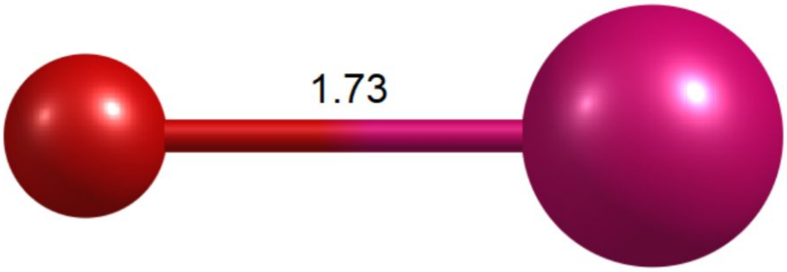

Figure S103.The optimized geometry of UO^{2+} singlet followed by its predicted frequencies(cm^{-1}) and IR intensities (km/mol).

Frequency (cm^{-1})	Intensity (km/mol)
1033.2712	97.6427

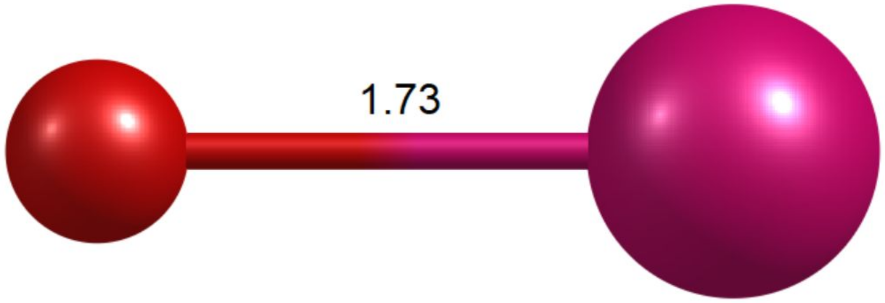

Figure S104. The optimized geometry of UO^{2+} triplet followed by its predicted frequencies (cm^{-1}) and IR intensities (km/mol).

Frequency (cm^{-1})	Intensity (km/mol)
1027.1672	94.2322

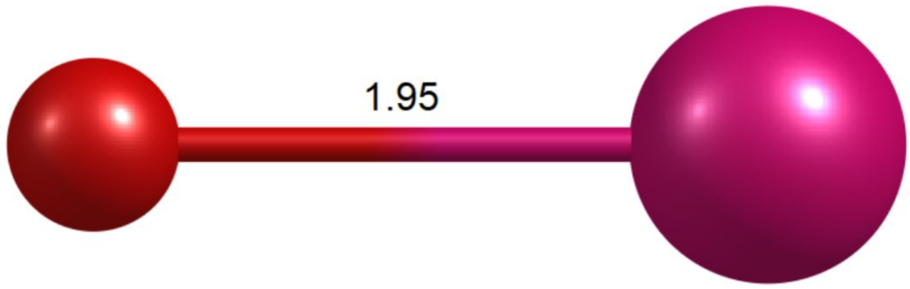

Figure S105.The optimized geometry of UO^{2+} quintet followed by its predicted frequencies(cm^{-1}) and IR intensities (km/mol).

Frequency (cm^{-1})	Intensity (km/mol)
712.8528	79.7006

Table S132. $UO^{2+}(CO_2)$ electronic energy calculated at the B3LYP/cc-pVTZ(-pp) level with Stuttgart/Koeln pseudopotential.

Isomer	2s + 1	Energy (hartree)	Rel. E (kcal/mol)	BDE (CO_2) (kcal/mol	BDE (CO) (kcal/mol)
1a	1	-738.036588	+9.9	40.0	
1a	3	-738.052286	+0.0	40.0	
1b	1	-738.032576	+12.4		68.3
1b	3	-737.949973	+64.2		68.7
1c	1	-737.891572	+100.8		
1c	3	-737.918839	+83.7		

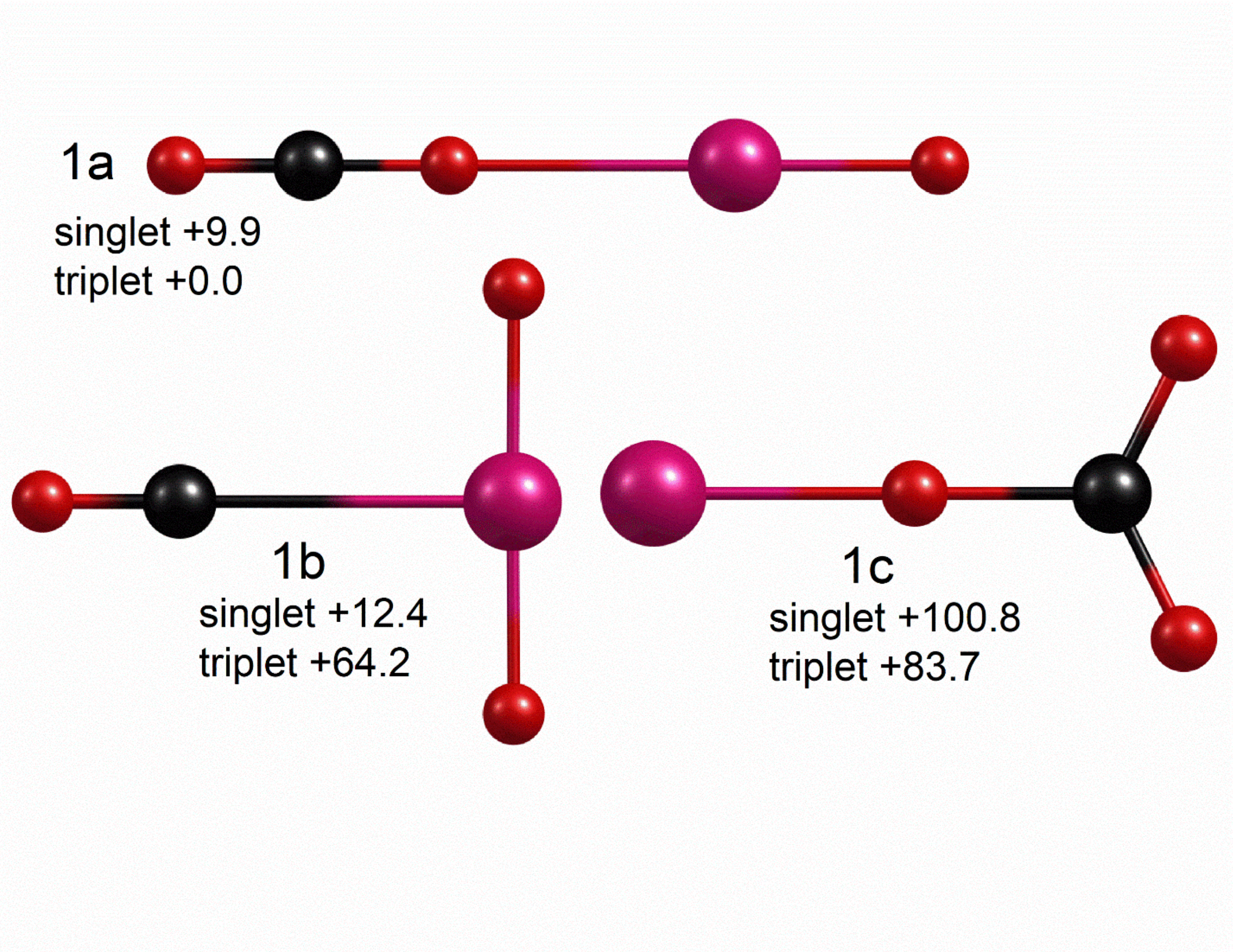

Figure S106. Predicted minimum energy structures of $UO^{2+}(CO_2)$ with energy of each spin state in kcal/mol. The lowest energy spin state of each isomer is shown.

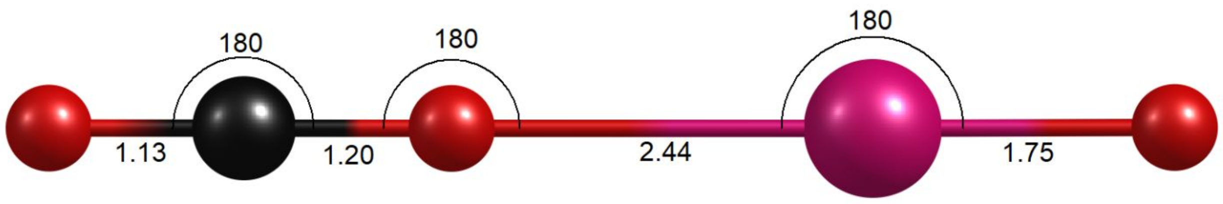

Figure S107. The optimized geometry of isomer 1a-singlet $UO^{2+}(CO_2)$ followed by its predicted frequencies (cm^{-1}) and IR intensities (km/mol).

Frequency (cm^{-1})	Intensity (km/mol)
20.6981	15.2053
101.0183	2.5767
105.4706	1.1559
212.5383	32.1154
631.1928	40.9552
631.6954	42.1084
1001.6891	177.0086
1327.9971	244.5982
2437.6345	907.2784

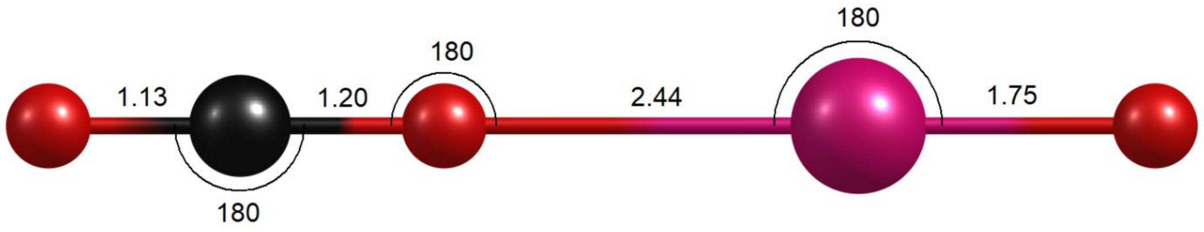

Figure S108. The optimized geometry of isomer 1a-triplet $UO^{2+}(CO_2)$ followed by its predicted frequencies (cm^{-1}) and IR intensities (km/mol).

Frequency (cm^{-1})	Intensity (km/mol)
28.7519	15.1759
94.5505	0.664
102.4332	1.7926
212.3708	32.4779
597.1921	53.9727
631.4452	41.1857
996.0323	170.4973
1327.5394	245.1839
2437.6808	896.6537

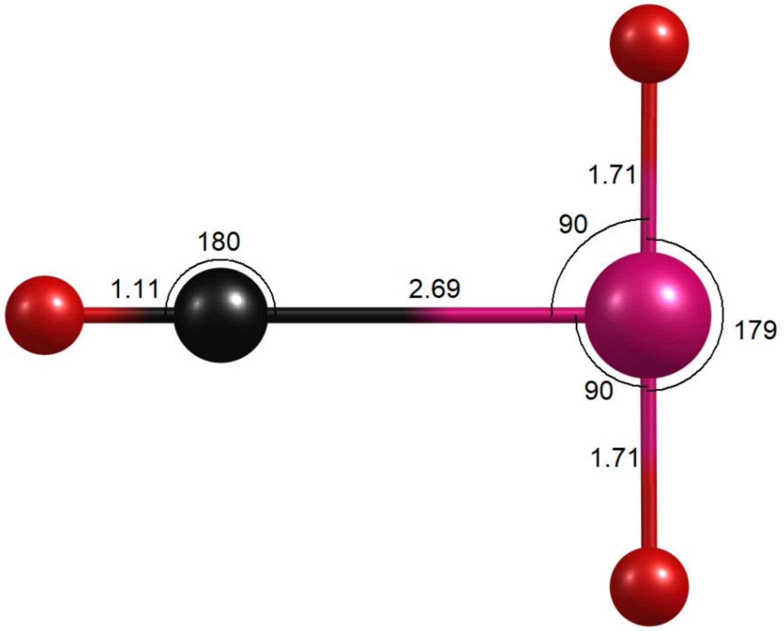

Figure S109. The optimized geometry of isomer 1b-singlet $UO^{2+}(CO_2)$ followed by its predicted frequencies (cm^{-1}) and IR intensities (km/mol).

Frequency (cm^{-1})	Intensity (km/mol)
80.4817	0.0551
163.6093	18.9817
185.4291	7.0513
223.637	0.3215
231.793	18.5508
269.3575	2.4039
1017.5076	1.6727
1100.5099	194.1774
2342.2428	3.874

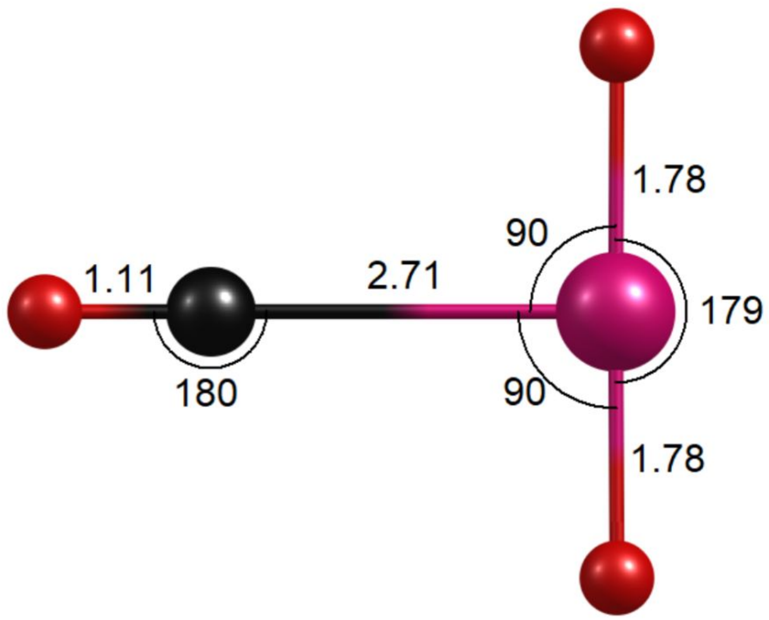

Figure S110. The optimized geometry of isomer 1b-triplet $UO^{2+}(CO_2)$ followed by its predicted frequencies (cm^{-1}) and IR intensities (km/mol).

Frequency (cm^{-1})	Intensity (km/mol)
46.206	0.7718
123.977	17.9394
152.5838	11.5898
221.1566	12.7981
222.0848	0.4076
244.103	6.4195
583.0271	153.0305
864.9891	0.8554
2338.4421	5.6569

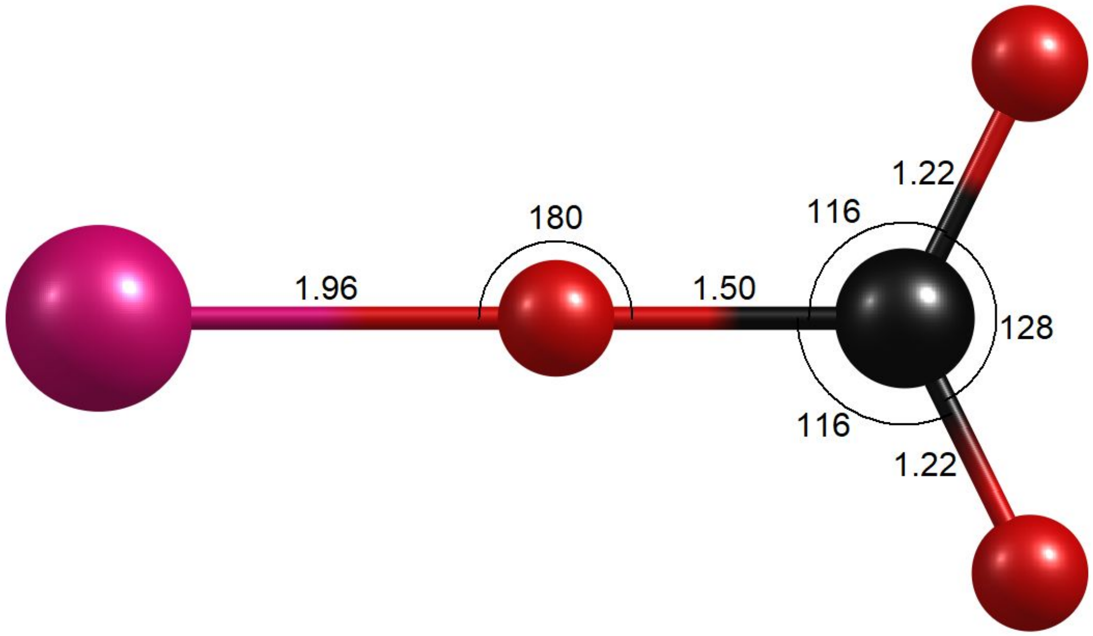

Figure S111. The optimized geometry of isomer 1c-singlet $UO^{2+}(CO_2)$ followed by its predicted frequencies (cm^{-1}) and IR intensities (km/mol).

Frequency (cm^{-1})	Intensity (km/mol)
95.6386	0.3699
135.2457	4.3438
279.4606	4.8343
382.6831	30.8276
549.552	486.3898
680.6751	43.4683
788.4948	850.2576
995.863	348.7316
1393.6748	250.9199

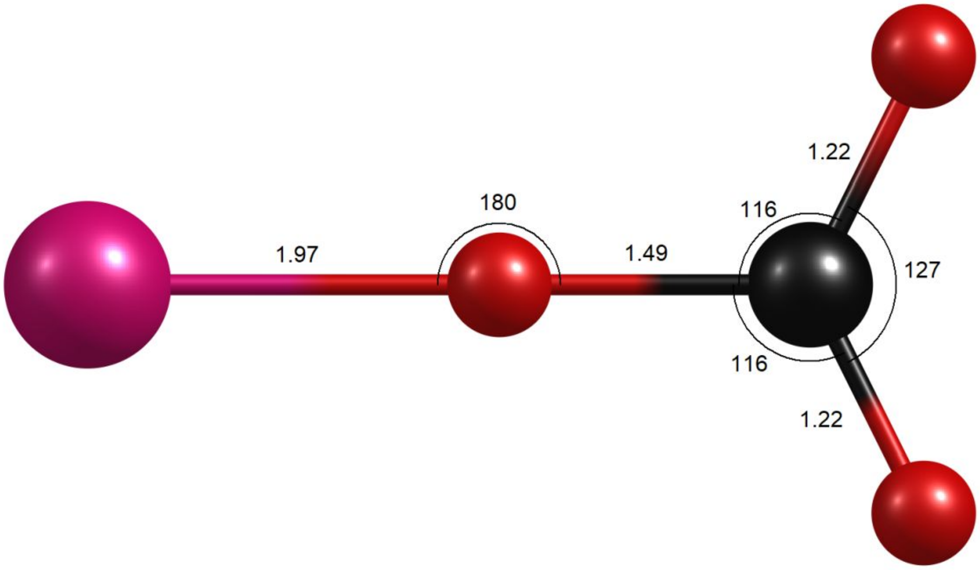

Figure S112. The optimized geometry of isomer 1c-triplet $UO^{2+}(CO_2)$ followed by its predicted frequencies (cm^{-1}) and IR intensities (km/mol).

Frequency (cm^{-1})	Intensity (km/mol)
89.095	0.0922
132.7027	4.199
282.4975	1.7508
389.0366	34.3815
562.9092	413.9985
682.2724	43.8602
797.8255	906.8177
989.6494	368.4492
1395.6472	262.751

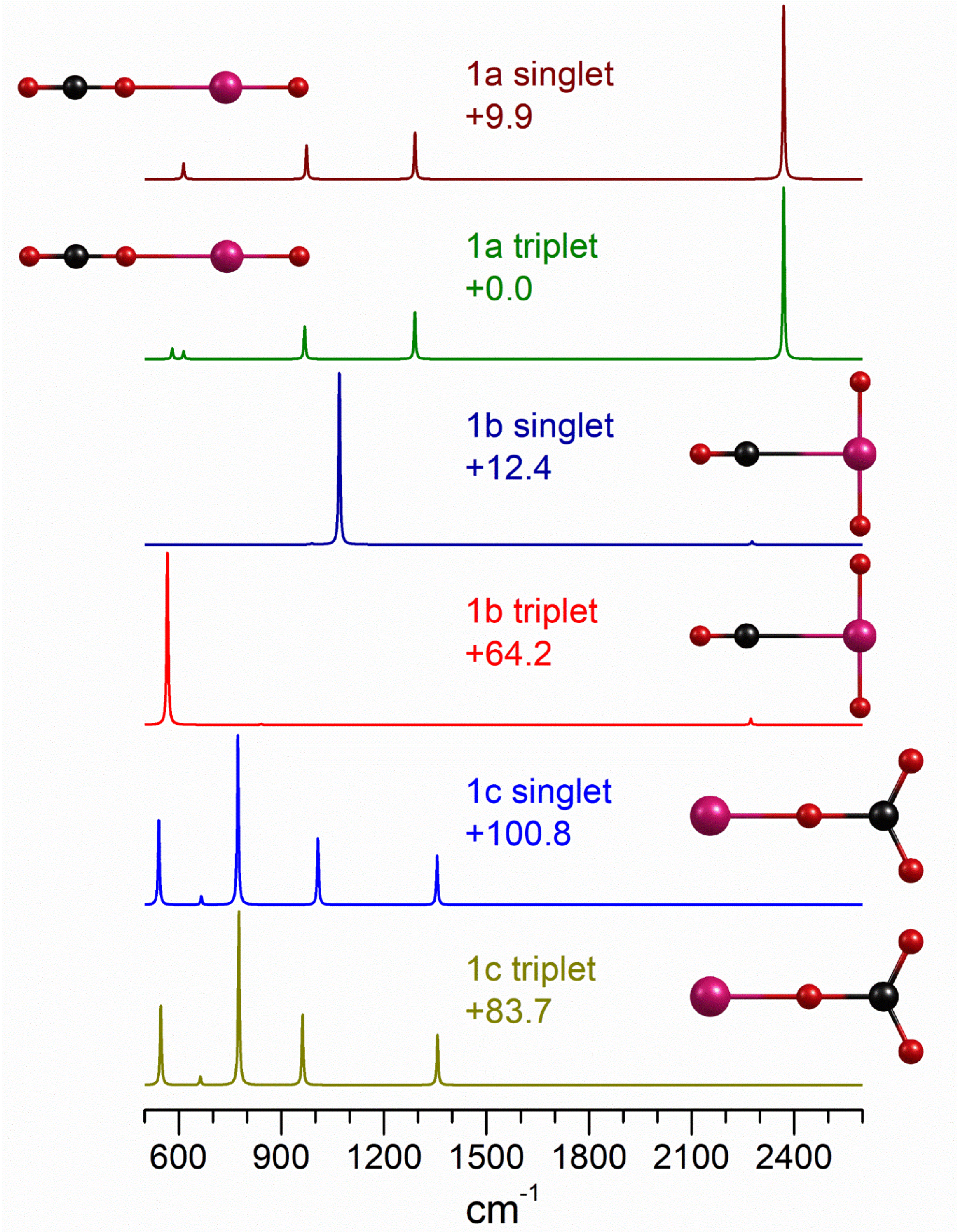

Figure S113. Simulated spectra of $UO^{2+}(CO_2)$ for isomer 1a-1c in their singlet and triplet spin states. Relative energies (kcal/mol) are shown next to each spectrum.

Table S133. $UO^{2+}(CO_2)_2$ electronic energy calculated at the B3LYP/cc-pVTZ(-pp) level with Stuttgart/Koeln pseudopotential.

Isomer	2s + 1	Energy (hartree)	Rel. E (kcal/mol)	BDE (CO_2) (kcal/mol)	BDE (CO) (kcal/mol)
2a	1	-926.744772	+8.9	37.2	
2a	3	-926.758963	+0.0	36.3	
2b	1	-926.745858	+8.2	40.4	28.9
2b	3	-926.661573	+61.1	39.4	27.3

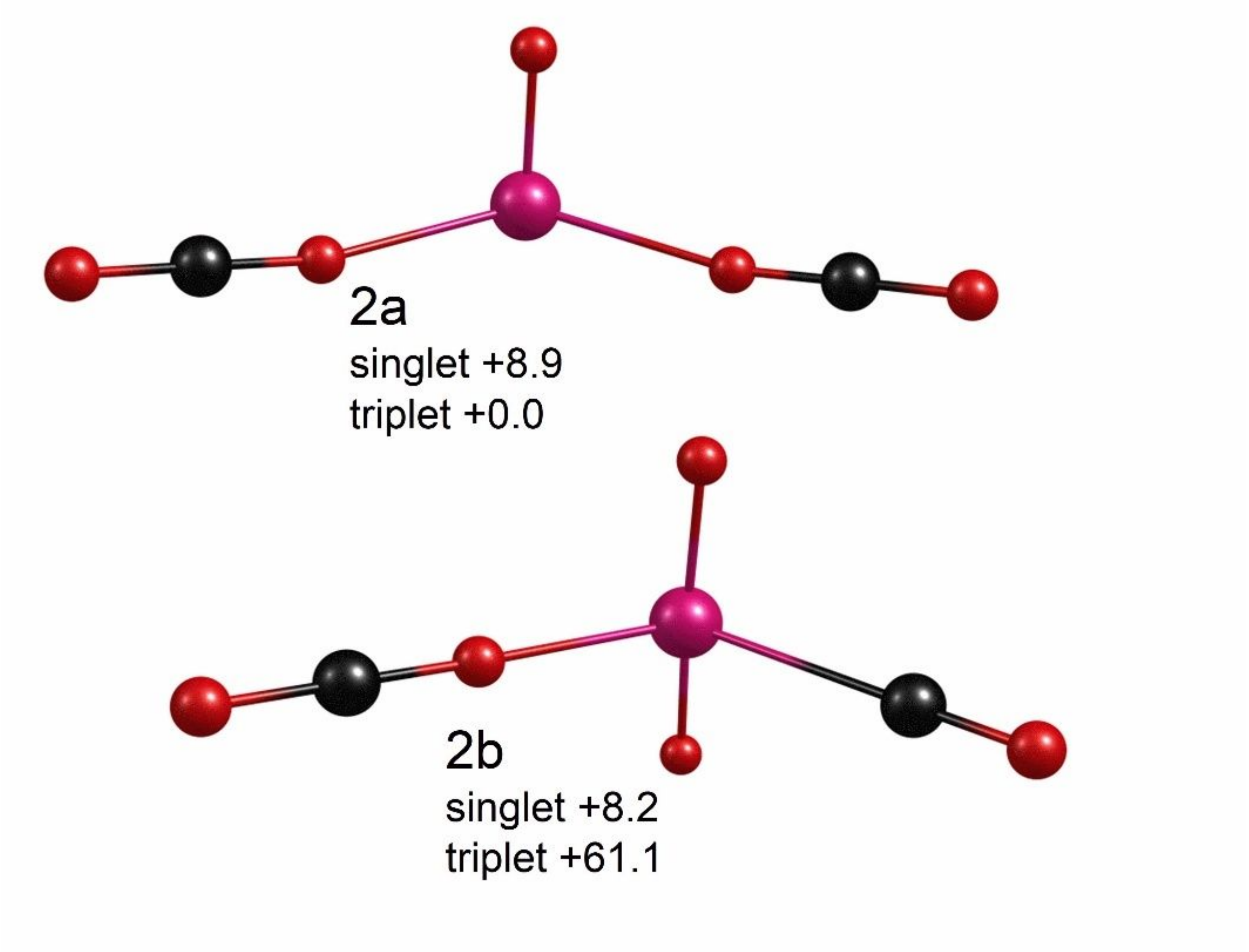

Figure S114. Predicted minimum energy structures of $UO^{2+}(CO_2)_2$ with energy of each spin state in kcal/mol. The lowest energy spin state of each isomer is shown

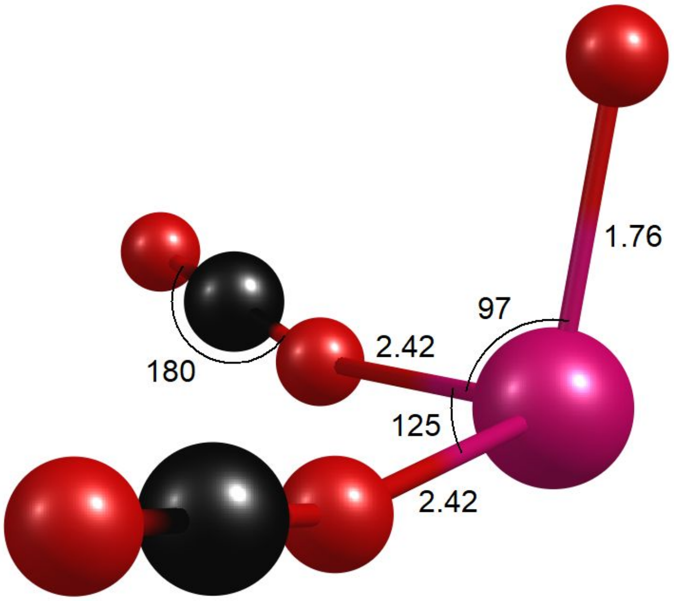

Figure S115. The optimized geometry of isomer 2a-singlet $UO^{2+}(CO_2)_2$ followed by its predicted frequencies (cm^{-1}) and IR intensities (km/mol).

Frequency (cm^{-1})	Intensity (km/mol)
16.7114	0.1211
43.8124	11.0079
64.5496	7.1882
94.7038	0.4463
99.4753	1.8449
120.7993	5.2163
143.4902	8.8572
215.7647	10.8462
221.886	66.1998
633.5855	4.5445
634.4654	69.8483
634.9738	12.0746
636.5261	64.3298
983.6808	128.5841
1344.8205	346.9776
1352.4011	60.8036
2434.9377	1698.5482
2449.4492	324.963

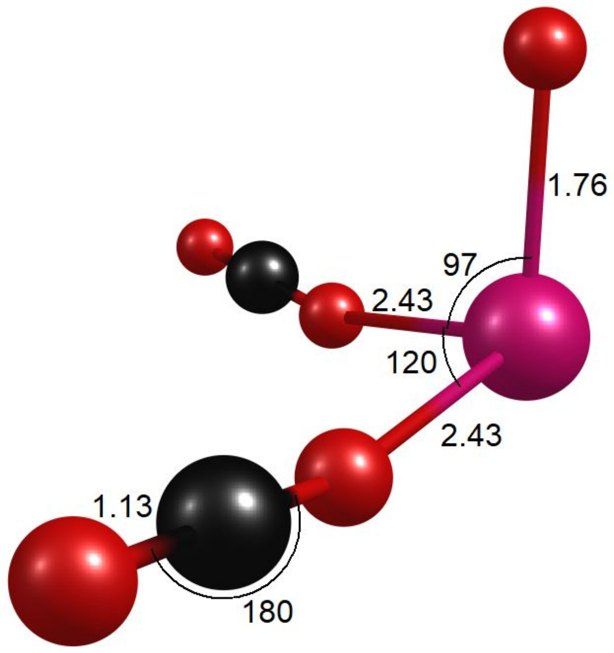

Figure S116. The optimized geometry of isomer 2a-triplet $UO^{2+}(CO_2)_2$ followed by its predicted frequencies (cm^{-1}) and IR intensities (km/mol).

Frequency (cm^{-1})	Intensity (km/mol)
18.1014	0.0253
44.7877	11.9635
64.5848	7.5658
94.2378	0.6459
99.4615	1.5111
119.9377	5.6422
140.477	9.8266
215.132	12.6861
220.074	60.23
633.9577	0.2439
634.838	71.9369
634.9815	19.2951
636.9736	60.773
980.5497	126.6152
1345.3587	335.9954
1352.5838	68.5713
2433.6157	1692.6132
2449.5682	367.1873

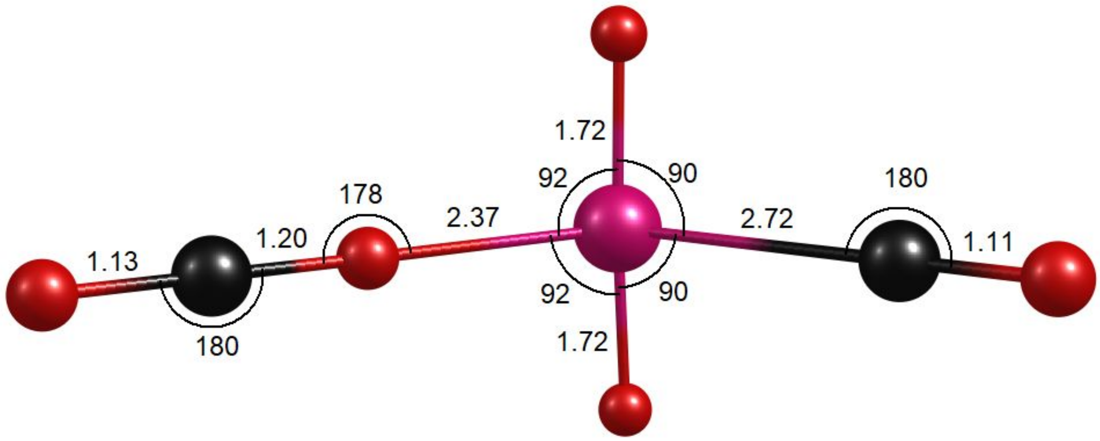

Figure S117. The optimized geometry of isomer 2b-singlet $UO^{2+}(CO_2)_2$ followed by its predicted frequencies (cm^{-1}) and IR intensities (km/mol).

Frequency (cm^{-1})	Intensity (km/mol)
17.6625	0.162
50.7851	0.6024
77.788	0.0118
98.8771	0.3137
155.2639	0.0309
181.5366	7.7724
193.7164	6.7238
217.9247	5.9748
224.4997	15.2392
243.2529	48.0987
265.3902	2.3134
619.1376	33.9701
631.5932	39.7136
995.181	7.106
1076.6464	216.9819
1349.0332	230.3055
2336.1514	9.9118
2443.9113	939.0229

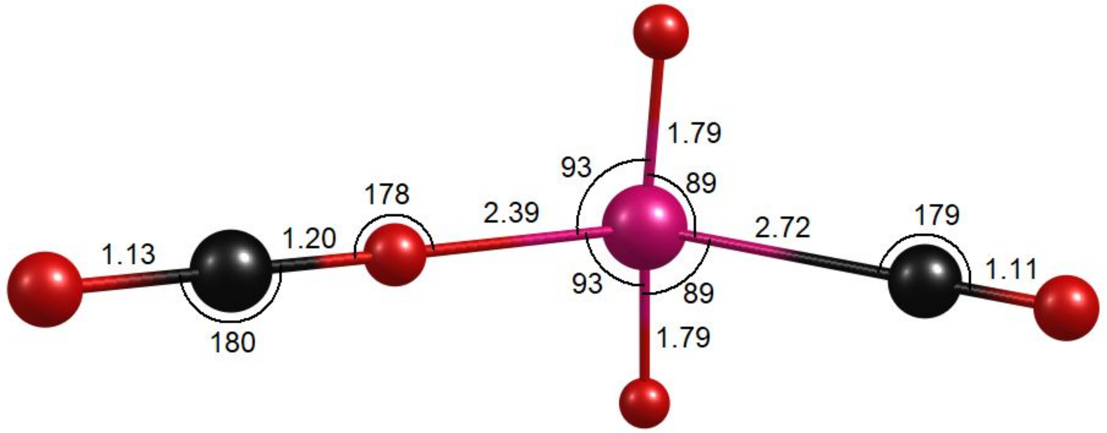

Figure S118. The optimized geometry of isomer 2b-triplet $UO^{2+}(CO_2)_2$ followed by its predicted frequencies (cm^{-1}) and IR intensities (km/mol).

Frequency (cm^{-1})	Intensity (km/mol)
25.4698	0.196
44.4925	0.5262
59.1558	0.0767
96.0982	0.0698
147.4555	0.0013
151.0535	14.3534
169.028	11.8434
209.3864	9.5964
219.5382	12.1595
230.1866	22.2249
246.7386	4.0693
626.9695	32.8195
629.6677	39.5019
639.5587	65.8879
847.1541	1.1173
1344.9881	201.878
2332.015	15.3538
2422.1871	720.7043

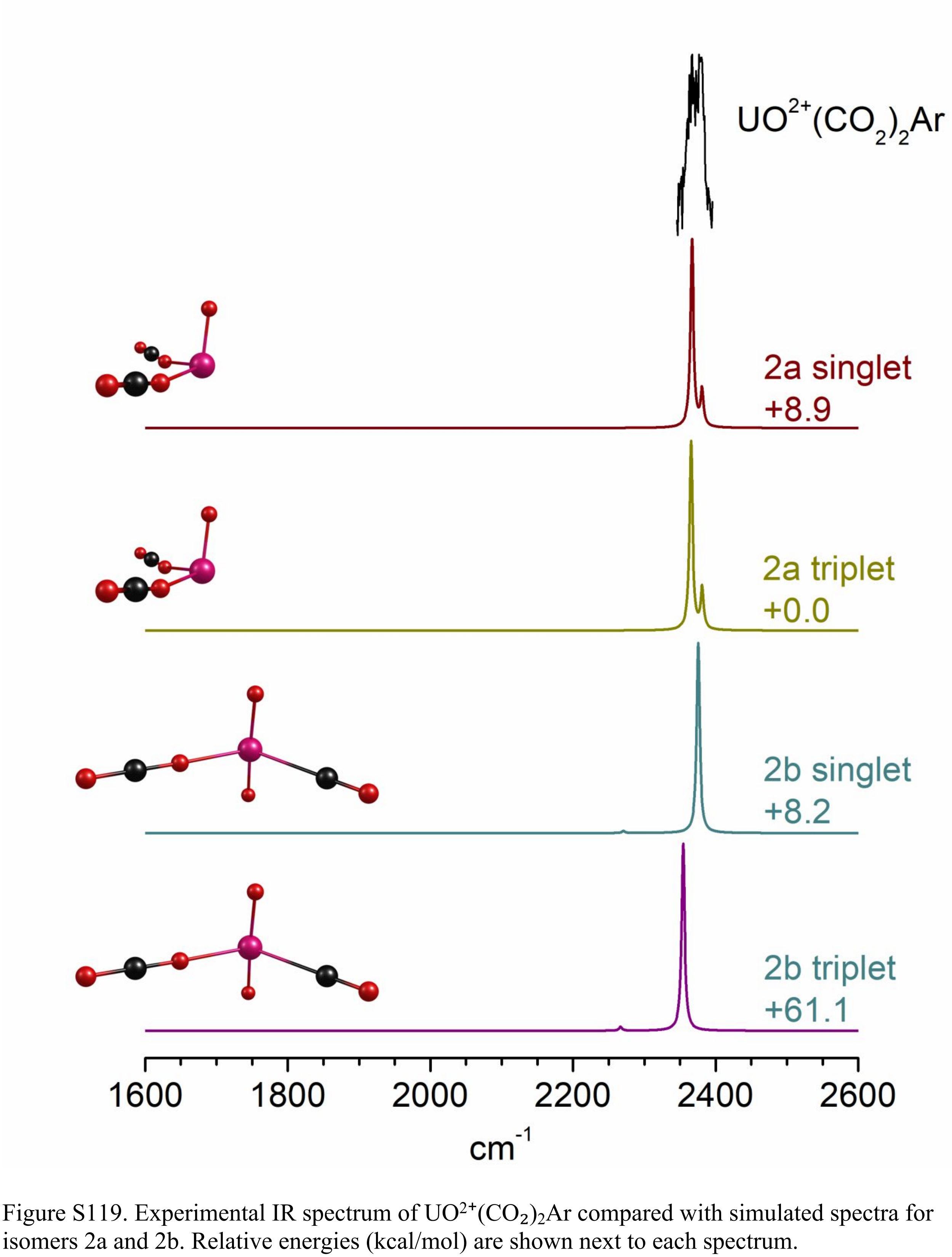

Figure S119. Experimental IR spectrum of $UO^{2+}(CO_2)_2Ar$ compared with simulated spectra for isomers 2a and 2b. Relative energies (kcal/mol) are shown next to each spectrum.

Table S134. $UO^{2+}(CO_2)_3$ electronic energy calculated at the B3LYP/cc-pVTZ(-pp) level with Stuttgart/Koeln pseudopotential.

Isomer	2s + 1	Energy (hartree)	Rel. E (kcal/mol)	BDE (CO_2) (kcal/mol)	BDE (CO) (kcal/mol)
3a	1	-1115.440572	+9.0	29.4	
3a	3	-1115.454846	+0.0	29.5	
3b	1	-1115.448309	+4.1	33.6	25.0
3b	3	-1115.361907	+58.3	32.3	22.8
3c	1	-1115.372725	+51.5		
3c	3	-1115.384813	+43.9		

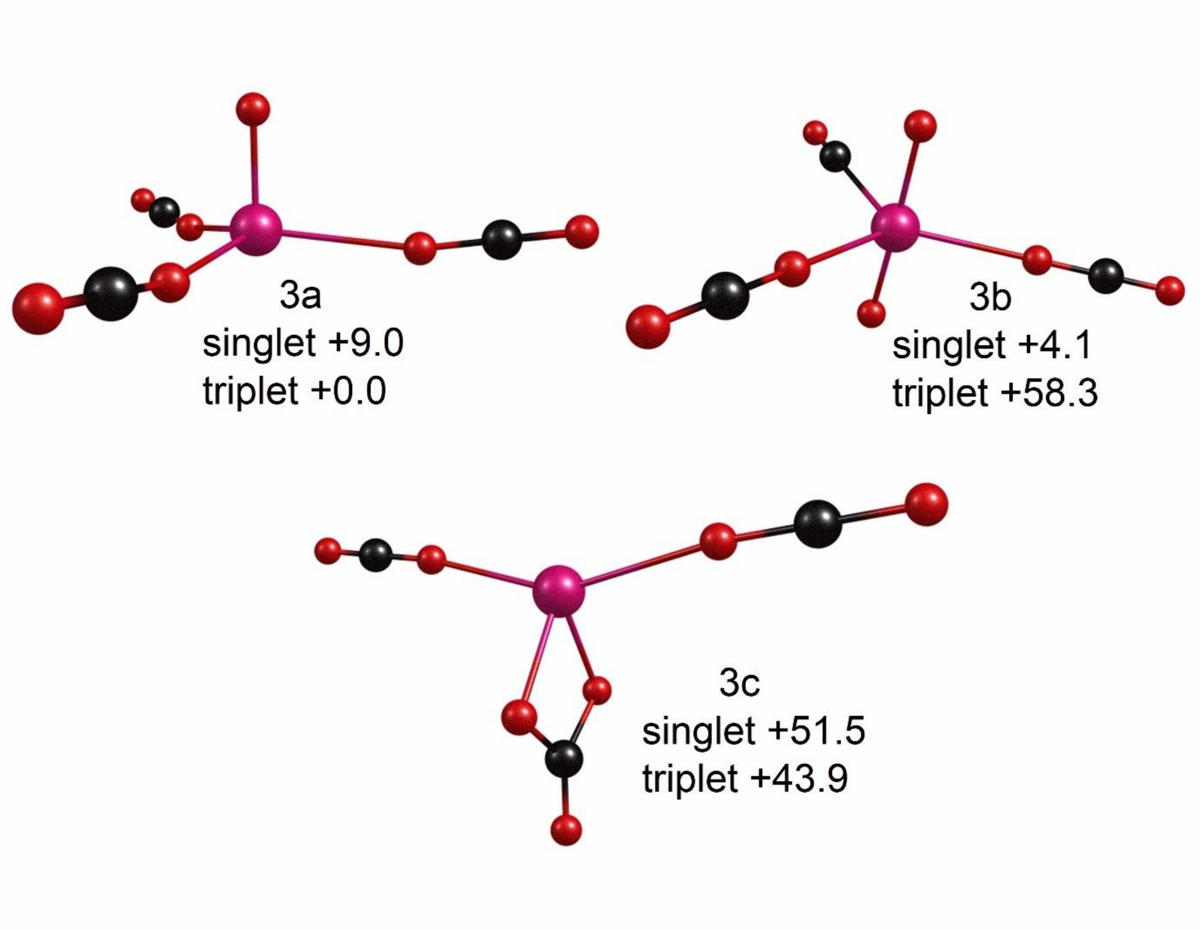

Figure S120. Predicted minimum energy structures of $UO^{2+}(CO_2)_3$ with energy of each spin state in kcal/mol. The lowest energy spin state of each isomer is shown

Table S135. Cartesian coordinates for the optimized geometry of isomer 3a-singlet $UO^{2+}(CO_2)_3$ followed by its predicted frequencies (cm^{-1}) and IR intensities (km/mol).

Z	x	y	z
92	-0.099625000	-0.000040000	-0.025565000
8	-0.175978000	-0.000071000	1.742146000
8	1.397527000	-1.937086000	-0.285392000
6	2.104725000	-2.886929000	-0.155614000
8	2.778256000	-3.792010000	-0.040536000
8	2.777306000	3.792653000	-0.040511000
6	2.103995000	2.887409000	-0.155598000
8	1.397029000	1.937395000	-0.285382000
8	-2.540410000	-0.000180000	-0.333956000
6	-3.723857000	-0.000284000	-0.198737000
8	-4.851694000	-0.000383000	-0.079908000

Frequency (cm^{-1})	Intensity (km/mol)	Frequency (cm^{-1})	Intensity (km/mol)
12.3995	0.553	638.0043	0.5138
19.0733	0.0991	638.2038	4.3177
20.9205	0.4862	639.0243	0.2793
49.1268	8.4343	639.4564	99.4095
62.6833	5.918	640.7528	65.3342
86.9944	0.0413	641.8139	47.6103
90.455	0.6479	965.8846	134.3955
95.1627	0.3667	1351.7906	226.5469
106.4872	0.8023	1352.4677	297.3848
123.0253	7.9878	1360.1587	0.1345
147.6892	10.4046	2432.3306	1361.7565
197.4764	1.3796	2435.7878	1775.0461
202.8741	43.0412	2453.916	14.538
211.2127	64.3892		

Table S136. Cartesian coordinates for the optimized geometry of isomer 3a-triplet $UO^{2+}(CO_2)_3$ followed by its predicted frequencies (cm^{-1}) and IR intensities (km/mol).

Z	x	y	z
92	-0.071509000	-0.000020000	-0.024657000
8	-0.097942000	-0.000055000	1.746049000
8	1.341613000	-1.999903000	-0.299312000
6	2.021182000	-2.968780000	-0.163321000
8	2.668057000	-3.892498000	-0.043054000
8	2.667380000	3.892944000	-0.043049000
6	2.020667000	2.969112000	-0.163305000
8	1.341266000	2.000115000	-0.299282000
8	-2.519858000	-0.000147000	-0.318719000
6	-3.703500000	-0.000227000	-0.188099000
8	-4.831929000	-0.000305000	-0.073031000

Frequency (cm^{-1})	Intensity (km/mol)	Frequency (cm^{-1})	Intensity (km/mol)
15.2228	0.0933	638.2049	4.2916
17.3304	0.1564	638.2371	0.8334
21.6239	0.4795	639.1989	11.3
55.9555	8.8022	639.602	98.1513
62.2688	5.8588	641.0684	53.3691
87.0661	0.0002	642.325	51.3193
92.2288	0.4817	963.1501	131.7053
94.4461	0.4288	1351.9952	235.4436
106.6189	0.7408	1353.0251	282.2361
130.82	10.7682	1360.284	0.5929
144.7093	10.0951	2432.081	1428.2226
196.3293	1.4207	2435.517	1734.1107
204.6137	43.9658	2453.674	8.341
210.1164	60.4748		

Table S137. Cartesian coordinates for the optimized geometry of isomer 3b-singlet $UO^{2+}(CO_2)_3$ followed by its predicted frequencies (cm^{-1}) and IR intensities (km/mol).

Z	x	y	z
92	0.000000000	0.149616000	0.000000000
6	-0.000020000	2.890630000	-0.000015000
8	-0.000028000	4.003285000	-0.000004000
8	0.000000000	0.188864000	-1.728164000
6	-3.200180000	-1.513668000	0.000004000
8	-2.143844000	-0.960547000	0.000025000
8	-4.203875000	-2.039030000	-0.000015000
8	2.143853000	-0.960532000	-0.000004000
6	3.200192000	-1.513645000	0.000002000
8	4.203891000	-2.039001000	0.000007000
8	0.000005000	0.188895000	1.728163000

Frequency (cm^{-1})	Intensity (km/mol)	Frequency (cm^{-1})	Intensity (km/mol)
14.6366	0.2637	241.0285	71.3231
19.3583	0.1832	263.0645	2.2672
30.2264	1.0864	625.1444	0
51.5146	0	625.7917	65.9623
74.781	0.0558	636.1823	16.1853
90.1147	0.0484	637.4869	60.1756
92.3839	0.3518	978.8149	1.431
139.0742	0.0671	1059.1521	230.042
165.6616	0	1354.9043	331.9596
186.6383	0.6192	1361.7411	81.7678
199.6614	0.3585	2330.359	15.9067
203.564	0.0503	2438.8284	1642.2719
211.6637	13.9591	2451.3805	428.632
233.016	41.0666		

Table S138. Cartesian coordinates for the optimized geometry of isomer 3b-triplet $UO^{2+}(CO_2)_3$ followed by its predicted frequencies (cm^{-1}) and IR intensities (km/mol).

Z	x	y	z
92	0.000001000	0.189620000	-0.000012000
6	-0.000060000	2.932046000	0.000018000
8	-0.000083000	4.045111000	0.000045000
8	-0.000007000	0.273292000	1.793375000
6	3.120664000	-1.641416000	0.000021000
8	2.086841000	-1.048311000	0.000033000
8	4.103432000	-2.206209000	0.000010000
8	-2.086806000	-1.048366000	0.000004000
6	-3.120617000	-1.641494000	0.000015000
8	-4.103373000	-2.206305000	0.000027000
8	-0.000004000	0.273305000	-1.793397000

Frequency (cm^{-1})	Intensity (km/mol)	Frequency (cm^{-1})	Intensity (km/mol)
21.9514	0.3774	228.3078	29.5376
27.6457	0.2479	250.9006	3.5134
29.4166	0.9063	630.7534	0
49.8892	0	631.9989	64.116
67.4183	0.0011	633.8058	18.8328
85.987	0.0005	635.795	56.6953
91.2127	0.0655	647.2578	44.7476
132.3551	0	832.2545	0.058
138.3954	0.0008	1353.8606	311.9321
169.7573	6.1435	1359.7766	87.1063
175.1952	11.1358	2325.7002	25.0462
194.2938	0.3023	2429.066	1506.9417
209.2564	37.2849	2440.5773	439.5123
219.1436	34.5824		

Table S139. Cartesian coordinates for the optimized geometry of isomer 3c-singlet $UO^{2+}(CO_2)_3$ followed by its predicted frequencies (cm^{-1}) and IR intensities (km/mol).

Z	x	y	z
92	0.000001000	-0.246058000	-0.000001000
6	-0.000008000	2.356199000	0.000001000
8	-0.163844000	1.462274000	-1.083604000
8	0.163836000	1.462272000	1.083604000
8	-0.000012000	3.514672000	0.000002000
6	-3.537772000	-0.978127000	0.020300000
8	-2.349726000	-0.840581000	0.033231000
8	-4.661072000	-1.114184000	0.009786000
8	4.661077000	-1.114173000	-0.009779000
6	3.537776000	-0.978119000	-0.020296000
8	2.349730000	-0.840577000	-0.033230000

Frequency (cm^{-1})	Intensity (km/mol)	Frequency (cm^{-1})	Intensity (km/mol)
11.8314	0.2858	633.5354	0.9171
17.0119	0.4904	634.1958	37.9093
35.1621	3.9083	634.7067	36.7304
58.1309	0.1221	635.275	67.1303
100.484	0.3285	729.9994	27.805
103.3771	0.355	803.6578	123.3908
104.2737	1.5573	854.7934	256.6272
108.6446	0.106	894.3036	160.38
148.5783	5.1644	1341.8435	459.7005
156.5659	4.2144	1350.171	25.2619
209.6435	8.0331	2005.7292	571.9922
224.3723	81.7038	2434.8367	2077.2155
380.975	16.1794	2447.4134	83.5362
518.8946	16.9369		

Table S140. Cartesian coordinates for the optimized geometry of isomer 3c-triplet $UO^{2+}(CO_2)_3$ followed by its predicted frequencies (cm^{-1}) and IR intensities (km/mol).

Z	x	y	z
92	-0.000001000	-0.238588000	-0.000003000
6	0.000006000	2.363646000	0.000004000
8	-0.178205000	1.470535000	-1.082340000
8	0.178216000	1.470535000	1.082341000
8	0.000005000	3.522161000	0.000003000
6	-3.532722000	-0.999378000	0.022176000
8	-2.345984000	-0.851060000	0.036590000
8	-4.654733000	-1.145500000	0.010385000
8	4.654729000	-1.145505000	-0.010372000
6	3.532718000	-0.999384000	-0.022167000
8	2.345980000	-0.851068000	-0.036585000

Frequency (cm^{-1})	Intensity (km/mol)	Frequency (cm^{-1})	Intensity (km/mol)
11.8204	0.3463	633.7843	1.3473
17.1024	0.494	634.3087	25.0076
35.2056	3.7879	634.8513	49.9449
60.5454	0.1384	635.3009	66.7109
100.3499	0.4229	728.3258	28.9145
103.4805	0.388	802.111	118.1828
104.1226	1.227	853.6434	259.8747
109.3127	0.072	891.6784	160.2367
130.1973	9.5061	1341.9637	457.2872
151.1722	1.2423	1350.207	27.0775
209.5837	8.288	2005.1105	571.6867
224.4128	81.2325	2435.13	2059.503
379.0724	16.665	2447.5559	90.231
518.1788	17.4594		

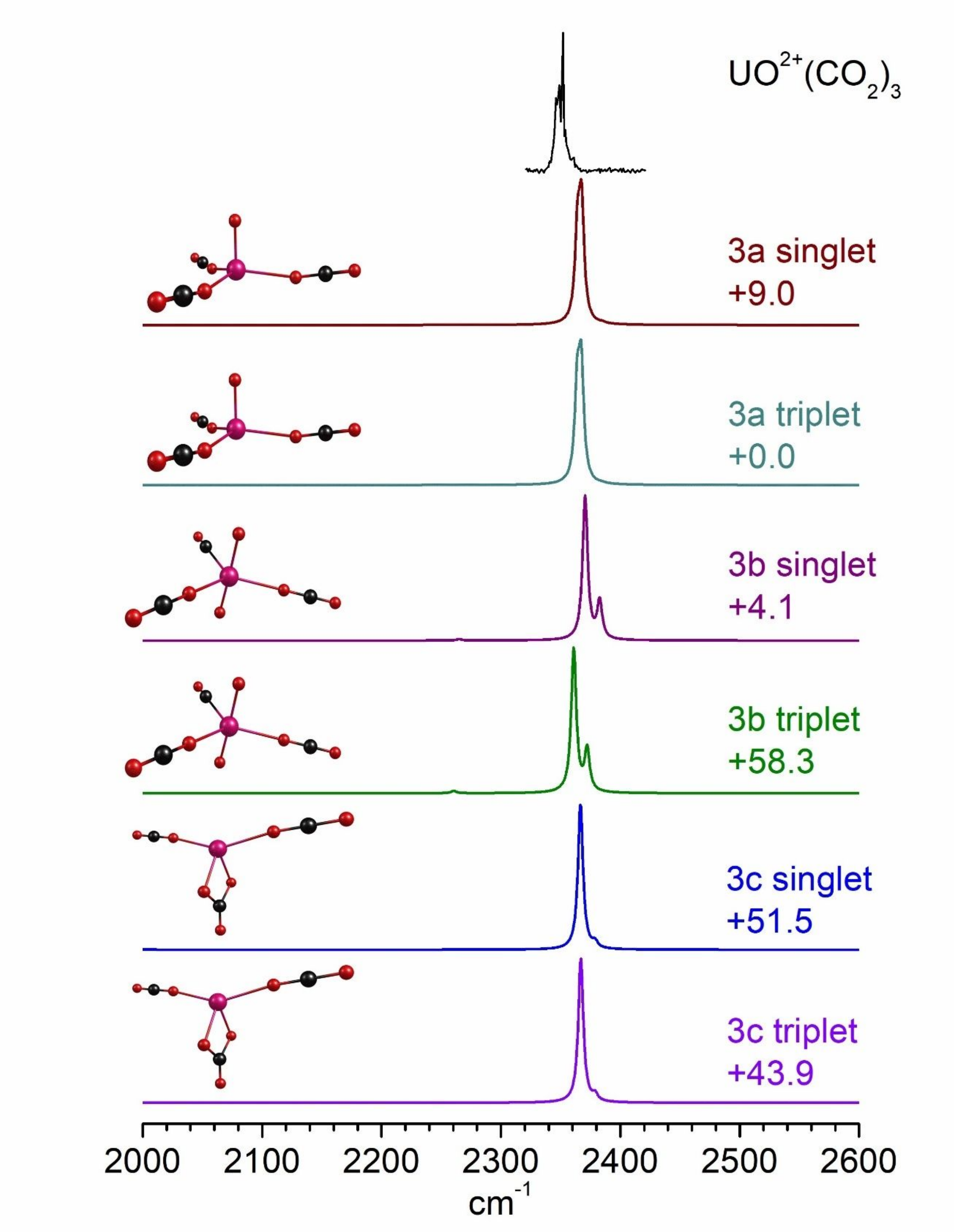

Figure S121. Experimental IR spectrum of $UO^{2+}(CO_2)_3$ compared with simulated spectra for isomers 3a, 3b and 3c. Relative energies (kcal/mol) are shown next to each spectrum.

Table S141. $UO^{2+}(CO_2)_4$ electronic energy calculated at the B3LYP/cc-pVTZ(-pp) level with Stuttgart/Koeln pseudopotential.

Isomer	2s + 1	Energy (hartree)	Rel. E (kcal/mol)	BDE (CO_2) (kcal/mol)	BDE (CO) (kcal/mol)
4a	1	-1304.139366	+0.0	26.5	19.5
4a	3	-1304.054237	+53.4	27.3	19.2
4b	1	-1304.12488	+9.1	22.2	
4b	3	-1304.13907	+0.2	22.2	
4c	1	-1304.124198	+9.5	21.8	
4c	3	-1304.138831	+0.3	22.0	
4d	1	-1304.067737	+44.9	28.9	
4d	3	-1304.080031	+37.2	29.1	

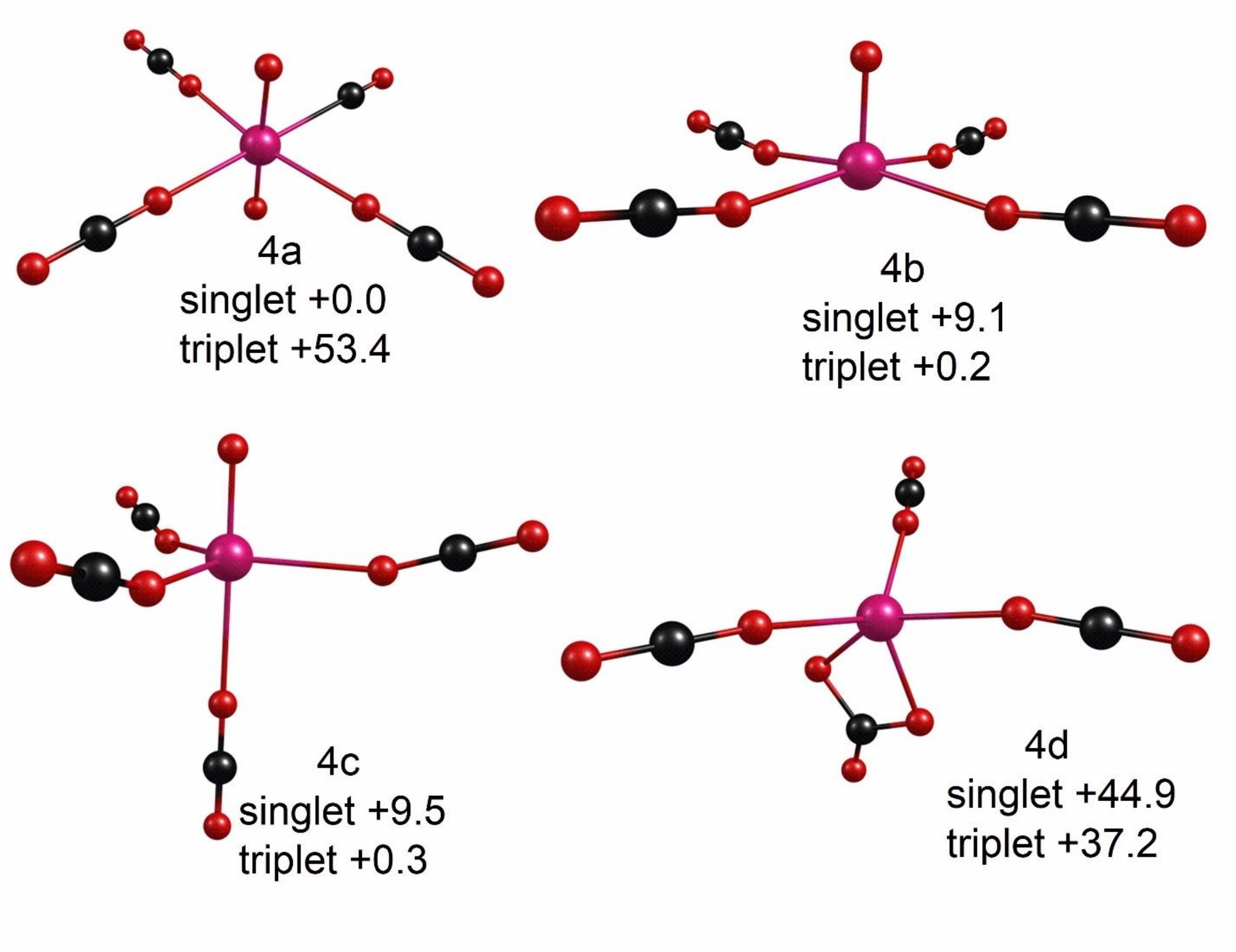

Figure S122. Predicted minimum energy structures of $UO^{2+}(CO_2)_4$ with energy of each spin state in kcal/mol. The lowest energy spin state of each isomer is shown.

Table S142. Cartesian coordinates for the optimized geometry of isomer 4a-singlet $UO^{2+}(CO_2)_4$ followed by its predicted frequencies (cm^{-1}) and IR intensities (km/mol).

Z	x	y	z
92	0.000043000	-0.096209000	-0.000010000
8	2.445593000	-0.318116000	0.000004000
6	3.630755000	-0.407373000	0.000016000
8	4.762615000	-0.492807000	0.000028000
8	-0.000324000	2.363010000	0.000011000
6	-0.000486000	3.551306000	0.000010000
8	-0.000640000	4.686509000	0.000010000
8	-2.445472000	-0.318534000	-0.000005000
6	-3.630618000	-0.407990000	0.000015000
8	-4.762464000	-0.493614000	0.000034000
8	0.000048000	-0.125854000	-1.734406000
6	0.000178000	-2.860907000	0.000006000
8	0.000224000	-3.974617000	0.000016000
8	0.000049000	-0.125852000	1.734386000

Frequency (cm^{-1})	Intensity (km/mol)	Frequency (cm^{-1})	Intensity (km/mol)
20.6035	0.1549	234.0843	58.6765
21.3391	0.1787	237.7487	73.2725
21.4938	0.0654	254.5297	2.0859
31.0691	1.0445	630.2343	6.8172
31.781	0.0006	630.6723	0
50.1468	0	632.2682	87.6712
67.1748	0.0028	640.0452	14.8182
82.1277	0.1114	642.7244	72.7307
92.4029	0.4225	643.9873	22.8393
97.4424	0.1128	966.3209	0.5464
118.4563	0.0949	1045.7906	238.139
144.7569	0.0028	1361.2157	338.8048
170.3207	0	1361.6061	135.1023
180.2535	0.0757	1369.0088	33.7547
185.9235	4.1907	2321.3706	21.0789
193.9474	1.9116	2435.1016	775.4328
194.0233	12.4526	2437.967	2098.255
218.0289	9.7688	2456.2964	281.9678

Table S143. Cartesian coordinates for the optimized geometry of isomer 4a-triplet $UO^{2+}(CO_2)_4$ followed by its predicted frequencies (cm^{-1}) and IR intensities (km/mol).

Z	x	y	z
92	-0.000012000	-0.104286000	-0.000005000
8	2.447096000	-0.284168000	0.000012000
6	3.632919000	-0.365802000	0.000025000
8	4.765367000	-0.443532000	0.000039000
8	0.000024000	2.350258000	-0.000001000
6	0.000030000	3.538989000	-0.000007000
8	0.000035000	4.674081000	-0.000013000
8	-2.447120000	-0.284163000	0.000014000
6	-3.632941000	-0.365819000	0.000027000
8	-4.765388000	-0.443570000	0.000040000
8	-0.000020000	-0.154453000	-1.789402000
6	0.000066000	-2.887020000	-0.000029000
8	0.000104000	-4.000919000	-0.000039000
8	-0.000013000	-0.154503000	1.789391000

Frequency (cm^{-1})	Intensity (km/mol)	Frequency (cm^{-1})	Intensity (km/mol)
20.7443	0.1074	224.6704	59.6091
21.6822	0.1741	224.8067	61.2805
22.7174	0.0758	243.9782	2.8042
30.2296	1.0683	632.8636	7.6091
30.5261	0.0014	633.0866	0
51.0739	0	634.9326	84.6202
59.9138	0.0015	639.9474	15.8859
81.6089	0.045	642.7624	71.5131
92.7636	0.3006	643.9554	20.6085
99.0703	0.0419	683.1758	16.5125
122.297	0.0003	827.8158	0.0616
138.5927	0.0036	1359.5637	150.8771
162.4305	0	1359.6457	359.8436
174.7606	0.2222	1367.3402	28.5242
184.4774	3.1717	2319.891	21.9379
191.5116	5.3405	2431.0866	788.8541
192.9809	1.4738	2434.3594	2126.9728
212.2976	37.6359	2452.2135	259.0301

Table S144. Cartesian coordinates for the optimized geometry of isomer 4b-singlet $UO^{2+}(CO_2)_4$ followed by its predicted frequencies (cm^{-1}) and IR intensities (km/mol).

Z	x	y	z
92	0.000268000	-0.055031000	0.010463000
8	0.000630000	-0.131589000	-1.765468000
8	0.013370000	-2.551615000	0.140276000
6	0.019386000	-3.699639000	-0.164333000
8	0.025159000	-4.801154000	-0.442714000
8	-4.788108000	0.241787000	0.516739000
6	-3.653588000	0.177632000	0.493530000
8	-2.468585000	0.109783000	0.475514000
8	2.467281000	0.135454000	0.475490000
6	3.651512000	0.215631000	0.493528000
8	4.785303000	0.291588000	0.516757000
8	-0.012702000	2.438953000	0.055158000
6	-0.018675000	3.587266000	-0.246202000
8	-0.024406000	4.688977000	-0.524466000

Frequency (cm^{-1})	Intensity (km/mol)	Frequency (cm^{-1})	Intensity (km/mol)
3.7919	0.0759	198.6782	70.0142
11.5112	0.0216	640.8534	0.5655
22.2103	0.0002	641.3918	9.8639
24.1438	0.5326	641.8967	0.0204
28.2666	0.0691	642.2731	2.016
52.0383	4.4817	644.1289	126.0498
60.454	4.2367	645.4686	75.9604
75.0571	0.1144	645.9917	46.0235
84.7142	1.2812	648.3373	7.6017
91.3865	0.251	951.6377	138.6826
92.0381	1.2133	1357.4876	212.3813
100.5949	0.1507	1358.3441	293.7208
108.184	0.3969	1359.677	63.0608
124.2111	8.1908	1366.7815	6.4915
169.1291	12.1975	2425.7551	2468.5006
171.9816	1.3676	2428.875	105.3105
187.4057	60.7041	2433.9184	1962.2488
189.575	0.7676	2458.7677	23.9307

Table S145. Cartesian coordinates for the optimized geometry of isomer 4b-triplet $UO^{2+}(CO_2)_4$ followed by its predicted frequencies (cm^{-1}) and IR intensities (km/mol).

Z	x	y	z
92	-0.000053000	-0.059646000	0.042556000
8	-0.000205000	-0.191989000	-1.731181000
8	-1.875035000	-1.708073000	0.305475000
6	-2.744467000	-2.495882000	0.122297000
8	-3.577355000	-3.250988000	-0.043134000
8	-3.250365000	3.484525000	-0.047064000
6	-2.482726000	2.657855000	0.091991000
8	-1.682083000	1.795304000	0.244975000
8	1.874303000	-1.708789000	0.305417000
6	2.743414000	-2.496962000	0.122278000
8	3.575993000	-3.252416000	-0.043123000
8	1.682985000	1.794397000	0.244926000
6	2.484080000	2.656531000	0.091961000
8	3.252150000	3.482803000	-0.047079000

Frequency (cm^{-1})	Intensity (km/mol)	Frequency (cm^{-1})	Intensity (km/mol)
6.4705	0.693	192.5867	69.7995
10.4588	0.0001	640.9121	5.4669
18.8539	0.0379	641.5439	7.8404
23.4696	0.5061	642.5629	4.6299
29.2995	0.0226	642.947	0.0024
50.8631	5.1675	644.5918	123.8868
60.0527	4.687	645.7995	67.1718
74.4891	0.0751	646.2862	55.4701
84.7576	0.602	648.7832	7.0845
90.205	1.0044	950.0967	133.711
92.2019	0.0184	1357.6675	182.97
103.8993	0.0324	1358.4624	260.089
105.0857	0.6052	1360.4118	95.0183
134.7743	13.3766	1367.157	10.7295
148.8739	9.7938	2428.7616	75.9204
170.6964	1.057	2432.2104	2095.6192
186.5454	1.6106	2433.3488	2043.9836
190.5724	58.3004	2458.7387	36.2825

Table S146. Cartesian coordinates for the optimized geometry of isomer 4c-singlet $UO^{2+}(CO_2)_4$ followed by its predicted frequencies (cm^{-1}) and IR intensities (km/mol).

Z	x	y	z
92	0.062322000	-0.000061000	-0.276302000
8	-0.015647000	-0.000535000	-2.053903000
8	-1.450339000	-1.968095000	-0.092419000
6	-2.152473000	-2.881699000	-0.382387000
8	-2.824883000	-3.758103000	-0.647144000
8	-2.825776000	3.757038000	-0.649451000
6	-2.153164000	2.880961000	-0.384123000
8	-1.450822000	1.967706000	-0.093559000
8	0.290731000	0.000793000	2.296951000
6	0.399227000	0.001204000	3.479529000
8	0.502917000	0.001598000	4.611467000
8	2.548305000	0.000049000	-0.370896000
6	3.647190000	-0.000021000	-0.822775000
8	4.703223000	-0.000084000	-1.241249000

Frequency (cm^{-1})	Intensity (km/mol)	Frequency (cm^{-1})	Intensity (km/mol)
10.3824	0.5191	203.4886	48.9099
17.3277	0.1807	641.3976	1.1522
21.3922	0.0003	641.6217	0.2343
23.8443	0.0213	642.9898	0.0065
25.5083	0.3014	643.3438	85.6511
48.8214	6.0176	644.2113	12.3011
57.4652	4.2272	644.9571	0.5231
79.8952	0.0214	646.1852	90.0524
82.6095	0.0172	646.3061	88.2113
85.9783	0.3734	947.5032	180.9932
90.1617	1.7539	1354.32	154.6053
91.9828	1.1091	1356.5833	178.8614
107.6404	0.1198	1356.995	228.5222
136.4786	12.2871	1364.0473	0.9511
158.9521	17.7823	2427.9103	1176.7448
162.737	18.3527	2429.2292	1327.5225
194.5207	33.4402	2434.1885	1674.4701
194.5252	3.6252	2456.9322	19.3984

Table S147. Cartesian coordinates for the optimized geometry of isomer 4c-triplet $UO^{2+}(CO_2)_4$ followed by its predicted frequencies (cm^{-1}) and IR intensities (km/mol).

Z	x	y	z
92	0.028099000	-0.000007000	-0.277461000
8	-0.070856000	0.001518000	-2.056029000
8	-1.366470000	-2.057124000	-0.103606000
6	-2.025407000	-2.997173000	-0.409936000
8	-2.656122000	-3.899536000	-0.689769000
8	-2.644962000	3.907574000	-0.685081000
6	-2.016883000	3.003081000	-0.406203000
8	-1.360693000	2.060788000	-0.100858000
8	0.225698000	-0.002038000	2.298664000
6	0.317558000	-0.002910000	3.482894000
8	0.405352000	-0.003743000	4.616015000
8	2.519978000	-0.002615000	-0.347530000
6	3.629786000	-0.003498000	-0.770875000
8	4.696145000	-0.004363000	-1.162910000

Frequency (cm^{-1})	Intensity (km/mol)	Frequency (cm^{-1})	Intensity (km/mol)
12.0752	0.1893	201.7191	45.3066
15.5595	0.2685	641.5299	1.5181
22.1443	0.0154	641.6322	0.985
23.5137	0.0093	643.083	7.337
25.7439	0.3145	643.4509	84.5563
53.7068	6.4979	644.6488	10.6851
56.4156	4.4408	645.0294	0.951
82.0615	0.2428	646.3086	83.4627
82.6653	0.0277	646.719	90.6614
84.7726	0.25	943.8432	176.9783
91.1281	1.4027	1353.9606	156.5953
92.1009	1.2722	1356.7835	187.4535
107.4378	0.0948	1357.6149	212.9916
143.9158	16.8858	1364.1283	0.5557
157.0886	15.8926	2428.2708	1153.8008
159.538	18.7865	2429.8088	1395.6124
193.5883	2.7091	2433.9782	1605.8343
197.6129	33.2878	2456.7606	14.32

Table S148. Cartesian coordinates for the optimized geometry of isomer 4d-singlet $UO^{2+}(CO_2)_4$ followed by its predicted frequencies (cm^{-1}) and IR intensities (km/mol).

Z	x	y	z
92	-0.000004000	-0.034330000	-0.083865000
6	-0.000085000	-1.217840000	2.245140000
8	-0.000101000	-1.790385000	0.946824000
8	-0.000125000	-1.768403000	3.268678000
8	-0.000011000	0.148526000	1.969413000
6	0.000211000	3.597349000	0.147097000
8	0.000139000	2.432406000	-0.108009000
8	0.000281000	4.706485000	0.380839000
8	-4.300278000	-1.338021000	-1.622663000
6	-3.280382000	-0.940866000	-1.328353000
8	-2.208648000	-0.515145000	-1.024840000
8	4.300151000	-1.338436000	-1.622636000
6	3.280296000	-0.941172000	-1.328330000
8	2.208607000	-0.515337000	-1.024820000

Frequency (cm^{-1})	Intensity (km/mol)	Frequency (cm^{-1})	Intensity (km/mol)
17.0656	0.5711	544.1998	21.9712
18.7158	0.3278	636.3338	0.1501
23.615	0.7702	637.0471	6.0044
27.1315	2.579	637.7178	0.409
30.3349	1.9785	638.1747	60.8458
51.1639	0.8941	639.2869	92.2252
88.0002	0.154	639.8629	50.4404
94.1424	0.3887	740.0992	25.7524
94.9222	0.4461	796.5229	169.7549
106.4517	0.004	859.4142	221.4995
107.7652	0.1678	922.7773	195.4794
107.9519	0.6858	1348.047	242.2881
153.4036	2.0138	1349.6205	313.994
199.1843	34.2998	1357.1065	32.3756
203.1474	12.3825	1986.5904	625.4579
211.1117	3.1061	2432.5041	1351.7608
213.5851	56.154	2434.8084	1721.1606
378.6331	22.7607	2452.7924	132.5073

Table S149. Cartesian coordinates for the optimized geometry of isomer 4d-triplet $UO^{2+}(CO_2)_4$ followed by its predicted frequencies (cm^{-1}) and IR intensities (km/mol).

Z	x	y	z
92	-0.000052000	0.043029000	-0.094572000
6	0.000775000	1.179592000	2.260294000
8	0.000328000	1.775058000	0.954656000
8	0.001145000	1.735978000	3.280481000
8	0.000648000	-0.170767000	1.971604000
6	-0.000615000	-3.586393000	0.142593000
8	-0.000505000	-2.422594000	-0.118061000
8	-0.000725000	-4.694404000	0.381288000
8	4.319798000	1.328047000	-1.595645000
6	3.295964000	0.934744000	-1.309848000
8	2.220216000	0.513096000	-1.015254000
8	-4.319879000	1.329268000	-1.594767000
6	-3.296111000	0.935689000	-1.309111000
8	-2.220435000	0.513756000	-1.014664000

Frequency (cm^{-1})	Intensity (km/mol)	Frequency (cm^{-1})	Intensity (km/mol)
17.0499	0.5557	533.9035	31.4983
19.2292	0.3565	636.5601	0.0147
23.7894	0.7522	637.0701	4.9409
27.8377	2.5267	637.9045	0.1383
30.3571	1.931	638.4102	65.6956
53.7413	0.7681	639.4602	88.845
87.9378	0.1756	639.7989	50.4367
94.2673	0.3164	738.8614	26.4676
95.086	0.443	794.0884	166.0193
106.7992	0.5311	838.9116	215.749
106.8164	0.0029	942.3758	189.022
108.4325	0.0701	1348.0015	242.7875
154.3719	1.9037	1349.9166	313.3065
180.0037	16.6061	1357.2574	31.4623
200.0341	28.2907	1988.309	623.3501
203.4042	11.7443	2432.5022	1343.2707
213.7648	56.0538	2435.2254	1718.666
375.0868	23.053	2452.8614	128.1852

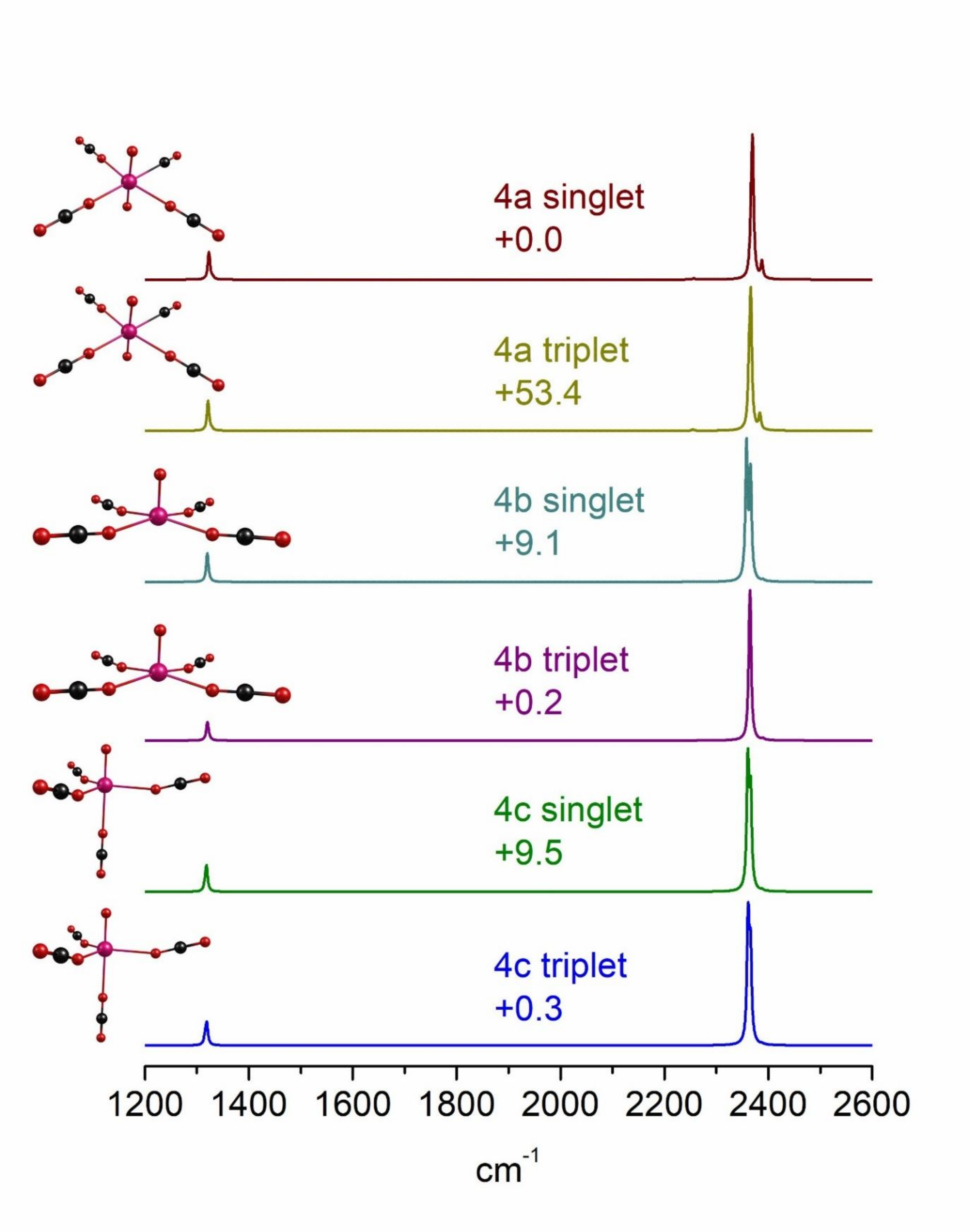

Figure S123. Simulated IR spectra of $UO^{2+}(CO_2)_3$ for isomers 4a, 4b and 4c. Relative energies (kcal/mol) are shown next to each spectrum.

Table S150. $UO^{2+}(CO_2)_5$ electronic energy calculated at the B3LYP/cc-pVTZ(-pp) level with Stuttgart/Koeln pseudopotential.

Isomer	2s + 1	Energy (hartree)	Rel. E (kcal/mol)	BDE (CO_2) (kcal/mol)	BDE (CO) (kcal/mol)
5a	1	-1492.804179	+9.2	19.1	
5a	3	-1492.818838	+0.0	18.9	
5b	1	-1492.814834	+2.5	16.7	11.5
5b	3	-1492.728804	+56.5	16.1	

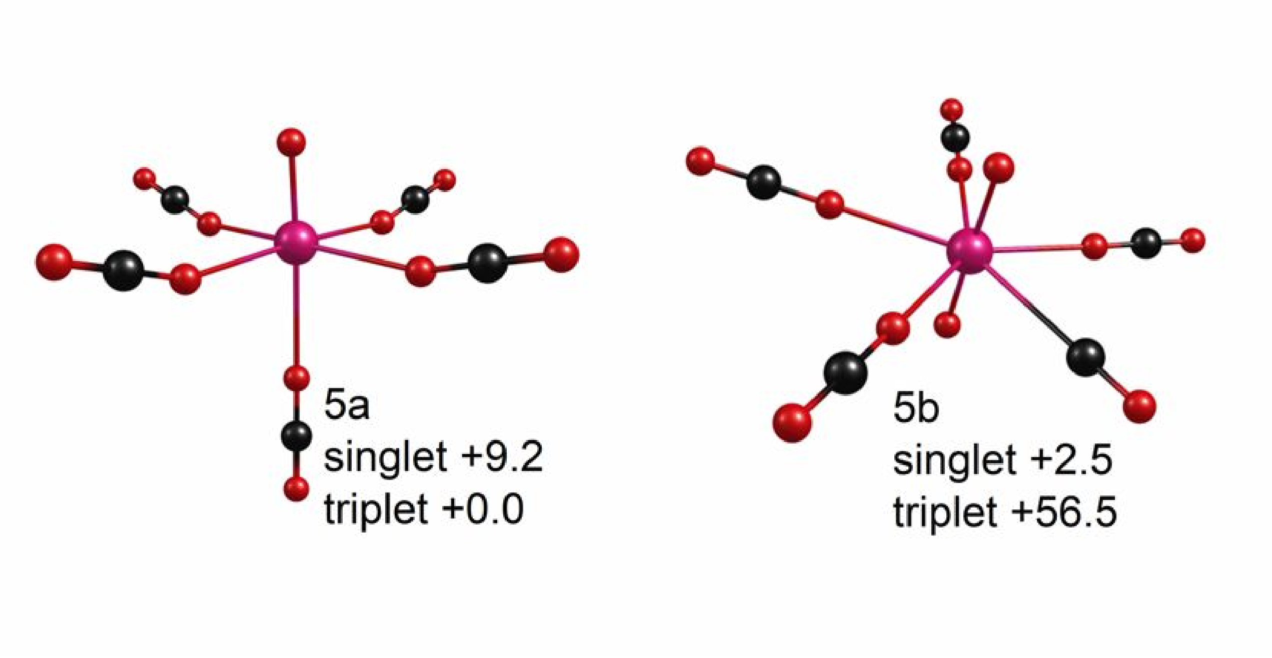

Figure S124. Predicted minimum energy structures of $UO^{2+}(CO_2)_5$ with energy of each spin state in kcal/mol. The lowest energy spin state of each isomer is shown.

Table S151. Cartesian coordinates for the optimized geometry of isomer 5a-singlet $UO^{2+}(CO_2)_5$.

Z	x	y	z
92	-0.040601000	-0.000234000	-0.216733000
8	-0.164339000	-0.000725000	-1.999093000
8	0.107497000	2.519725000	-0.197547000
6	0.144792000	3.638510000	-0.586734000
8	0.180845000	4.716971000	-0.947531000
8	4.760469000	0.013708000	-0.640226000
6	3.649363000	0.010500000	-0.392272000
8	2.495947000	0.007167000	-0.124443000
8	-0.019310000	-0.000385000	2.398886000
6	-0.019794000	-0.000496000	3.583933000
8	-0.019947000	-0.000601000	4.722114000
8	0.121587000	-2.519327000	-0.197697000
6	0.165074000	-3.637787000	-0.587186000
8	0.207098000	-4.715936000	-0.948265000
8	-2.573619000	-0.006581000	-0.064181000
8	-4.802325000	-0.012266000	-0.719329000
6	-3.708750000	-0.009475000	-0.404747000

Frequency (cm^{-1})	Intensity (km/mol)	Frequency (cm^{-1})	Intensity (km/mol)
9.6011	0.2685	188.9791	47.0697
17.6142	0.001	643.1606	1.6509
19.9012	0.0757	643.7422	5.5041
21.3021	0.0003	645.0547	3.3806
23.4892	0.0089	645.1016	11.5407
26.0837	0.0525	647.123	78.7025
26.2113	0.3487	647.4629	4.058
50.8326	3.3845	648.4987	19.4789
53.8829	3.8064	648.9871	89.0362
71.2731	0.0053	649.5493	108.3791
76.316	0.0135	651.5015	8.7799
80.5205	0.2702	935.3779	180.5272
85.2114	1.2131	1358.7005	136.4673
89.5391	1.1866	1360.6597	128.7969
94.2087	0.0275	1361.3843	220.8935
94.224	0.0076	1363.3331	77.0709
105.6455	0.0075	1369.1476	8.4089
149.6656	15.3315	2427.3406	547.8156
155.8366	15.6416	2428.0869	738.5481
156.5256	20.7389	2431.2747	1978.5862
169.3111	3.5676	2431.9112	1969.7927
184.341	47.721	2461.0582	10.1693
185.848	8.0667		

Table S152. Cartesian coordinates for the optimized geometry of isomer 5b-singlet $UO^{2+}(CO_2)_5$.

Z	x	y	z
92	0.000496000	-0.119849000	-0.000243000
8	-2.349096000	-1.013182000	-0.000450000
6	-3.467539000	-1.402397000	-0.002325000
8	-4.542374000	-1.775211000	-0.004060000
8	-1.511229000	1.886731000	-0.001664000
6	-2.221612000	2.833352000	0.002736000
8	-2.904689000	3.743542000	0.006790000
8	2.350403000	-1.011964000	0.000922000
6	3.469704000	-1.398695000	0.001218000
8	4.545349000	-1.769171000	0.001511000
8	0.000099000	-0.147001000	1.736803000
6	0.001423000	-2.893355000	0.002472000
8	0.001897000	-4.008178000	0.003614000
8	1.512000000	1.886778000	-0.002031000
6	2.215867000	2.838260000	-0.002371000
8	2.892800000	3.753038000	-0.002712000
8	0.000752000	-0.149993000	-1.737224000

Frequency (cm^{-1})	Intensity (km/mol)	Frequency (cm^{-1})	Intensity (km/mol)
11.6806	0	238.1235	63.6159
16.6505	0.08	246.9597	1.9104
30.5243	0.076	274.6324	0.0136
31.5562	0.0071	634.1402	0.0002
31.6175	0.9432	635.0838	9.4181
37.6406	0.014	636.6933	0.0003
46.6256	0.0001	638.9804	109.088
49.1694	0	639.5912	16.1767
61.2538	0	643.708	86.1554
68.0215	0.6145	646.9221	35.6732
92.1104	0	649.5081	0.0115
105.2164	2.2774	960.5051	0.4462
113.1016	0.0878	1039.2632	238.5319
122.5379	1.4547	1367.8479	42.1675
143.8112	0.4279	1368.6157	145.2891
148.7054	0.0049	1369.6433	267.8009
152.3296	0.0191	1376.8476	38.6147
159.1768	3.4283	2311.2995	23.5173
170.5436	0.0005	2428.5879	2.7328
175.0233	7.5315	2435.0135	1216.9015
179.0826	24.1732	2441.0859	2567.7105
180.9078	3.0899	2464.1478	313.2102
237.3741	77.8304		

Table S153. Cartesian coordinates for the optimized geometry of isomer 5b-triplet $UO^{2+}(CO_2)_5$.

Z	x	y	z
92	-0.000007000	-0.109352000	-0.000306000
8	-2.347409000	-1.017885000	-0.000933000
6	-3.461112000	-1.420133000	0.002168000
8	-4.531724000	-1.805430000	0.005037000
8	-1.517601000	1.886735000	-0.000742000
6	-2.234119000	2.829115000	-0.001411000
8	-2.922799000	3.734952000	-0.002035000
8	2.347234000	-1.018298000	0.000465000
6	3.460950000	-1.420519000	0.000932000
8	4.531574000	-1.805794000	0.001380000
8	-0.000748000	-0.135792000	1.792155000
6	-0.000235000	-2.894379000	-0.001984000
8	-0.000326000	-4.009505000	-0.002816000
8	1.517872000	1.886512000	0.000899000
6	2.234501000	2.828811000	0.001467000
8	2.923287000	3.734568000	0.002014000
8	0.000733000	-0.134682000	-1.792784000

Frequency (cm^{-1})	Intensity (km/mol)	Frequency (cm^{-1})	Intensity (km/mol)
11.5012	0	222.8785	65.6176
15.3707	0.0564	240.1417	2.3973
30.6472	0.0649	274.0233	0.037
31.3874	0.879	635.8601	0.0001
31.5402	0.0006	636.6648	8.8855
37.7051	0.0147	638.2041	0.0001
46.9194	0.0026	639.7401	16.1225
50.0015	0	640.5515	106.3658
59.5656	0.002	644.0065	83.6872
68.6625	0.5796	647.2736	33.8017
97.2038	0	649.6189	0.2843
104.2241	1.6681	684.0608	13.3724
114.907	0.0003	822.3794	0.0132
121.3615	1.6299	1366.8877	14.4022
141.6009	0.0353	1367.5449	161.1144
141.9885	0.7358	1368.0605	318.2682
151.882	0.0564	1375.6246	34.3378
156.3036	3.108	2308.0725	27.0821
160.7434	0.0001	2426.1072	0.5092
171.9149	5.8626	2432.2343	1259.5576
175.9995	25.2246	2437.3097	2658.7957
179.5389	3.2726	2460.8407	284.7943
221.4468	84.9336		

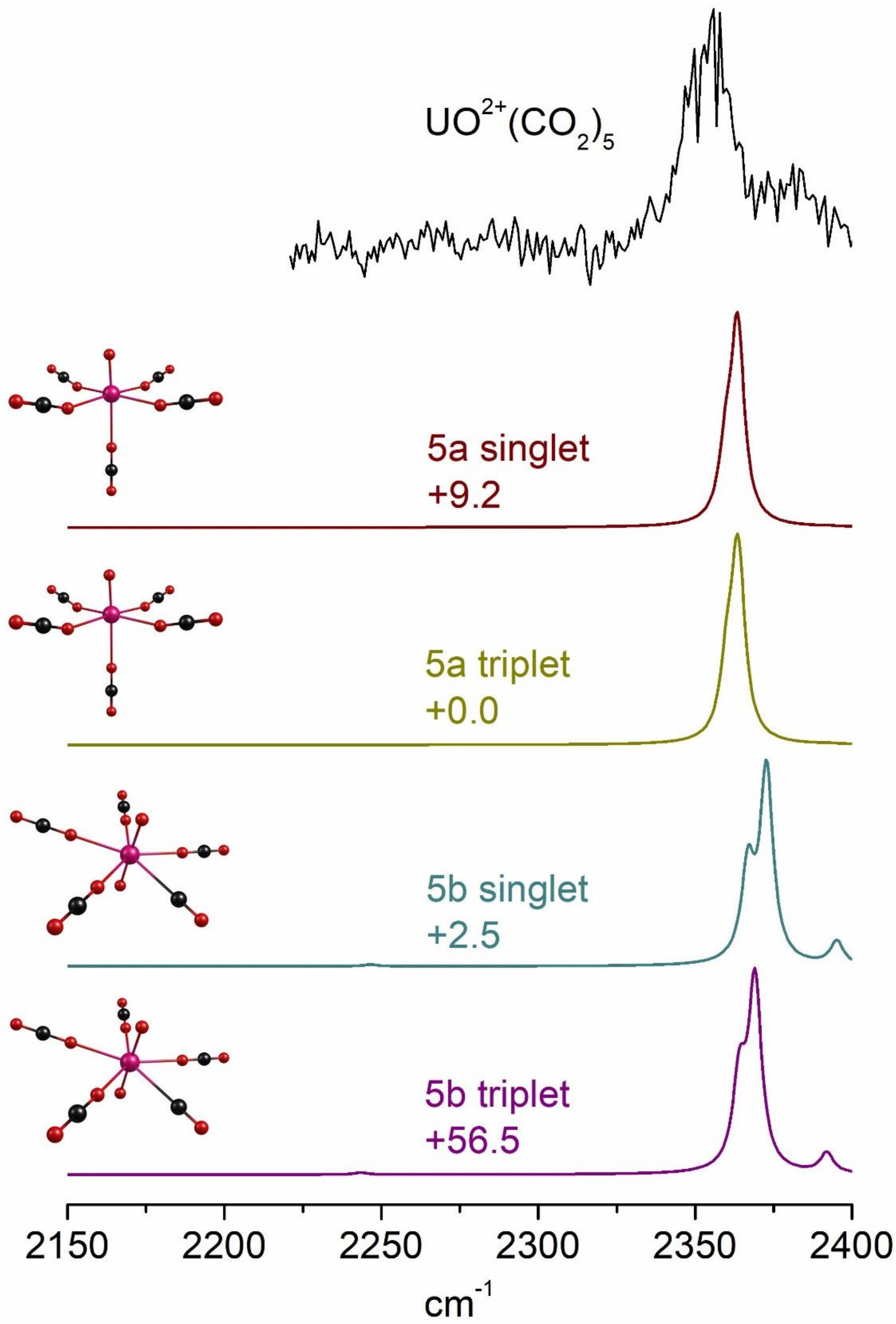

Figure S125. Experimental IR spectrum of $UO^{2+}(CO_2)_5$ compared with simulated spectra for 5a and 5b isomers. Relative energies (kcal/mol) are shown next to each spectrum.

Table S154. $UO^{2+}(CO_2)_6$ electronic energy calculated at the B3LYP/cc-pVTZ(-pp) level with Stuttgart/Koeln pseudopotential.

Isomer	2s + 1	Energy (hartree)	Rel. E (kcal/mol)	BDE (CO_2) (kcal/mol)
6a	1	-1681.473976	+9.1	13.1
6a	3	-1681.473976	+0.0	13.1
6b	1	-1681.466828	+13.6	2.0
6b	3	-1681.382349	+66.6	2.9

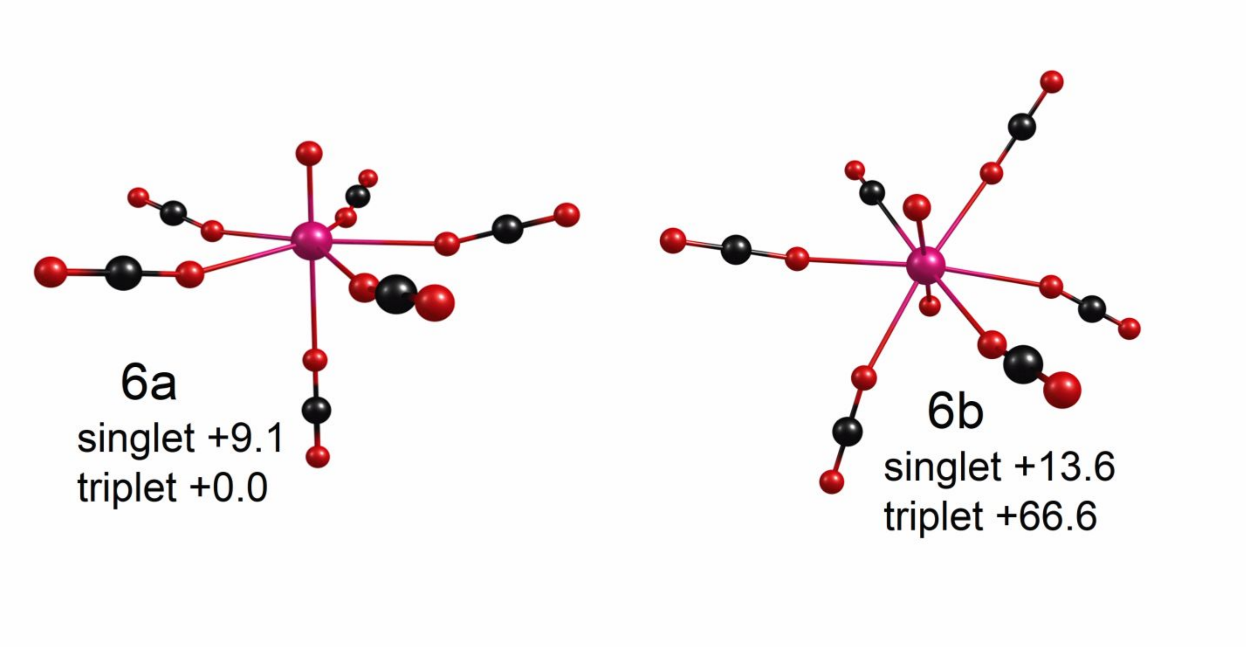

Figure S126. Predicted minimum energy structures of $UO^{2+}(CO_2)_6$ with energy of each spin state in kcal/mol. The lowest energy spin state of each isomer is shown.

Table S155. Cartesian coordinates for the optimized geometry of isomer 6a-singlet $UO^{2+}(CO_2)_6$ followed by its predicted frequencies (cm^{-1}) and IR intensities (km/mol).

Z	x	y	z
92	0.003149000	-0.000190000	-0.233319000
8	0.009752000	-0.000493000	-2.023563000
8	-2.089813000	-1.503039000	-0.042164000
6	-3.030451000	-2.178574000	-0.273616000
8	-3.940272000	-2.831878000	-0.488003000
8	-3.888286000	2.898509000	-0.508214000
6	-2.990887000	2.230287000	-0.287598000
8	-2.063129000	1.539362000	-0.049601000
8	0.824703000	2.436248000	-0.244241000
6	1.184506000	3.477856000	-0.670523000
8	1.533011000	4.487184000	-1.069898000
8	2.571193000	-0.023314000	0.115119000
6	3.747501000	-0.034100000	0.013830000
8	4.884482000	-0.044521000	-0.076519000
8	0.781240000	-2.450597000	-0.247244000
8	1.451597000	-4.511670000	-1.079334000
6	1.121761000	-3.497334000	-0.676826000
6	-0.049358000	0.002850000	3.586041000
8	-0.031475000	0.001790000	2.403080000
8	-0.066521000	0.003867000	4.725266000

Frequency	Intensity	Frequency	Intensity	Frequency	Intensity
11.1662	0.005	131.3856	0.276	650.522	82.5195
20.3025	0.0027	131.5472	0.054	650.9717	127.8738
23.3934	0.0048	139.2775	0.001	653.8233	0.2429
27.6853	0.237	141.0678	8.5699	653.9189	1.0643
28.518	0.0005	145.2783	5.0237	931.2867	183.1271
28.686	0.0033	151.8104	6.7689	1362.4703	112.4911
35.6391	0.0001	169.2032	22.3109	1366.8333	34.1965
35.786	0.0004	170.5405	65.3844	1367.2271	14.1355
44.7112	0.0016	174.412	3.1953	1368.615	164.1551
52.3933	2.4969	175.3315	54.464	1369.479	186.8317
52.6966	2.7979	642.4247	0.0299	1374.9389	0.7374
75.0111	0.0765	645.0262	3.3935	2420.9635	0.0075
77.8441	0.1375	645.1499	3.7198	2423.8905	5.7849
80.1439	0.1383	646.3438	2.2937	2428.3451	1224.2388
83.5809	0.3121	647.0319	8.0477	2436.0683	2482.0982
95.6882	1.8869	648.2884	34.1062	2436.0988	2316.5795
98.462	2.5548	648.5711	59.7603	2467.9966	0.6907
102.6459	0.014	650.4843	58.9817		

Table S156. Cartesian coordinates for the optimized geometry of isomer 6a-triplet $UO^{2+}(CO_2)_6$ followed by its predicted frequencies (cm^{-1}) and IR intensities (km/mol).

Z	x	y	z
92	0.000000000	0.000001000	-0.234682000
8	0.000026000	0.000015000	-2.026541000
8	0.298872000	2.563063000	-0.094690000
6	0.431667000	3.702034000	-0.377517000
8	0.560168000	4.804119000	-0.640826000
8	4.742253000	0.951884000	-0.639826000
6	3.654321000	0.733492000	-0.376874000
8	2.529973000	0.507791000	-0.094420000
8	1.264767000	-2.249227000	-0.094610000
6	1.826857000	-3.248737000	-0.377300000
8	2.370745000	-4.215876000	-0.640483000
8	-1.748323000	-1.897889000	-0.094627000
6	-2.525223000	-2.741341000	-0.377284000
8	-3.276962000	-3.557479000	-0.640426000
8	-2.345263000	1.076266000	-0.094481000
8	-4.396056000	2.017286000	-0.640010000
6	-3.387538000	1.554522000	-0.376995000
6	-0.000094000	0.000021000	3.584893000
8	-0.000074000	0.000010000	2.401530000
8	-0.000113000	0.000032000	4.724059000

Frequency	Intensity	Frequency	Intensity	Frequency	Intensity
9.3108	0	131.898	0	650.6418	62.7227
21.5059	0.0085	131.9048	0	651.3216	135.5995
21.5081	0.0084	142.5888	12.5752	653.9647	0
27.5173	0.2375	145.0324	0	653.966	0
28.445	0.0018	145.0479	0	928.9918	180.2328
28.4473	0.0018	156.3455	9.7052	1362.1098	113.8447
35.7432	0	156.3625	9.7256	1367.7972	0
35.7454	0	171.4736	65.1158	1367.7994	0.0003
43.4589	0	171.4806	65.1261	1369.1732	199.0281
52.5207	2.798	172.2782	3.9128	1369.1748	199.0379
52.5583	2.7965	642.4158	0	1375.1608	0.9859
78.9085	0	645.8152	0	2423.8714	0
78.9248	0.0001	645.8164	0	2423.8737	0.0001
79.8583	0.3151	646.4721	8.78	2428.6379	1164.6621
79.8712	0.3149	646.475	8.7688	2436.3159	2409.5706
96.6016	2.1881	648.5922	52.2949	2436.3178	2409.7166
96.628	2.1864	648.5946	52.345	2468.05	0.7263
101.4874	0.0071	650.6411	62.6372		

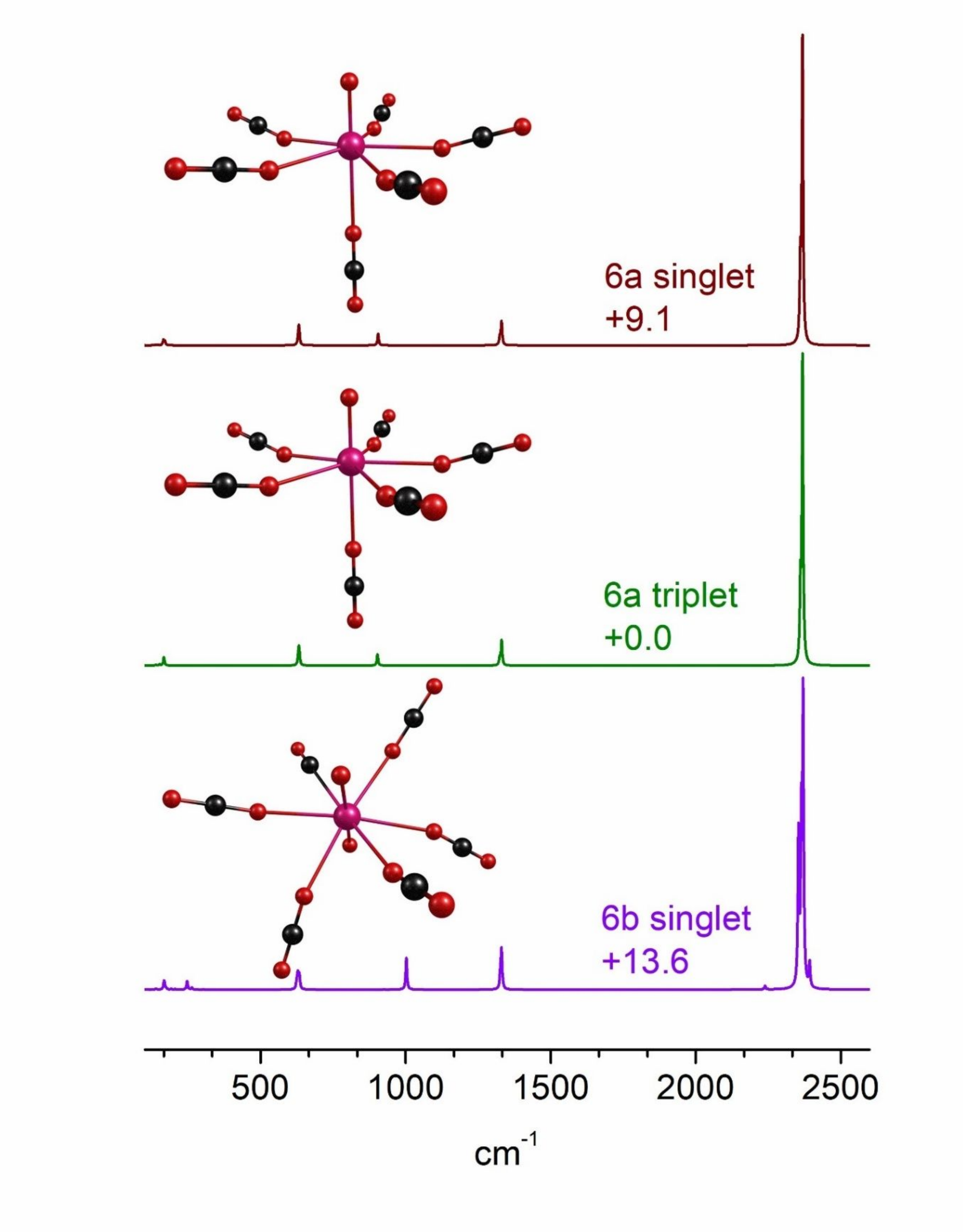

Figure S127.Simulated spectra of $UO^{2+}(CO_2)_6$ for 6a-singlet and 6a-triplet. Relative energies (kcal/mol) are shown next to each spectrum.

Table S157. $UO^{2+}(CO_2)_7$ electronic energy calculated at the B3LYP/cc-pVTZ(-pp) level with Stuttgart/Koeln pseudopotential.

Isomer	2s + 1	Energy (hartree)	Rel. E (kcal/mol)	BDE (CO_2) (kcal/mol)	BDE (CO) (kcal/mol)
7a	1	-1870.134698	+9.0	7.4	
7a	3	-1870.149120	+0.0	7.4	
7b	1	-1870.131452	+11.1	9.9	8.2
7b	3	-1870.045347	+65.1	8.9	

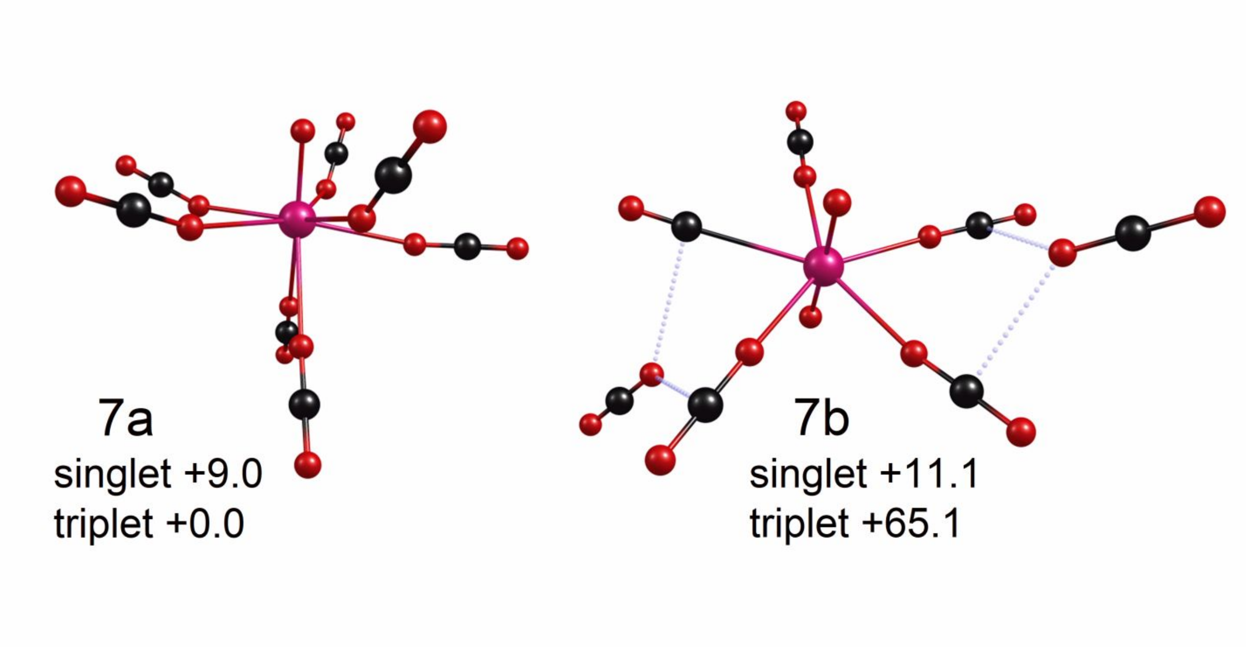

Figure S128. Predicted minimum energy structures of $UO^{2+}(CO_2)_7$ with energy of each spin state in kcal/mol. The lowest energy spin state of each isomer is shown.

Table S158. Cartesian coordinates for the optimized geometry of isomer 7a-singlet $UO^{2+}(CO_2)_7$ followed by its predicted frequencies (cm^{-1}) and IR intensities (km/mol).

Z	x	y	z
92	-0.000018000	0.077043000	-0.249761000
8	-0.000111000	0.337675000	-2.028357000
8	1.521856000	2.146881000	0.104875000
6	2.182782000	3.104446000	-0.091221000
8	2.824063000	4.031038000	-0.271184000
8	2.981386000	-0.774798000	3.712179000
6	2.240190000	-0.584406000	2.863564000
8	1.474281000	-0.387816000	1.990218000
8	2.427979000	-0.725429000	-0.831934000
6	3.189769000	-0.894811000	-1.718307000
8	3.938289000	-1.064234000	-2.563075000
8	-2.428002000	-0.725641000	-0.831674000
6	-3.189889000	-0.895113000	-1.717947000
8	-3.938496000	-1.064629000	-2.562619000
8	-1.474049000	-0.387833000	1.990387000
8	-2.980947000	-0.774817000	3.712529000
6	-2.239851000	-0.584422000	2.863828000
6	-2.183025000	3.104281000	-0.091173000
8	-1.522028000	2.146770000	0.104952000
8	-2.824369000	4.030825000	-0.271159000
8	0.000089000	-2.521838000	-0.194464000
6	0.000135000	-3.690363000	-0.354465000
8	0.000180000	-4.821863000	-0.504128000

Frequency	Intensity	Frequency	Intensity	Frequency	Intensity
8.3272	0.0396	86.7366	0.4654	644.4651	2.632
19.2855	0.0178	91.662	0.9426	646.0357	2.6448
19.4242	0.2051	95.4565	2.2643	646.1356	2.548
22.7161	0.0053	98.3227	2.4272	647.6908	10.4916
27.1432	0.1799	105.2413	1.4755	648.8687	43.6391
27.8307	0.0311	107.6547	2.9954	649.8576	53.5467
28.7342	0.0065	119.982	1.385	651.7558	48.1902
29.2621	0.0022	121.0298	0.7313	652.0209	1.542
30.4668	0.009	124.4611	13.2525	654.5532	61.6129
32.3512	0.0048	135.1403	2.8866	654.925	65.0598
34.8478	0.0098	137.6867	0.9659	656.2539	83.3894
43.9595	1.0136	138.731	2.2185	657.1097	44.546
46.6008	1.6532	160.4105	80.8284	657.7083	0.0031
47.9247	1.1743	164.0376	2.5111	658.1919	10.5043
61.0829	0.0019	166.7174	66.5109	917.7091	192.4823
71.1511	2.5229	185.1105	0.7256	1362.4364	57.2791
72.0344	0.0196	216.3274	5.8779	1363.0876	69.2867

1368.4885	18.1239	1375.6583	3.0714	2430.2913	1459.6691
1369.7781	101.4854	2419.6852	0.0014	2432.3692	2344.7435
1370.3018	89.4015	2420.6764	536.0773	2435.8226	2469.9331
1371.4354	141.3326	2421.1885	98.4663	2468.9542	0.5737

Table S159. Cartesian coordinates for the optimized geometry of isomer 7a-triplet $UO^{2+}(CO_2)_7$.

Z	x	y	z
92	0.000004000	0.061268000	-0.249054000
8	-0.000017000	0.279052000	-2.034658000
8	1.517976000	2.149690000	0.030159000
6	2.174767000	3.098908000	-0.214258000
8	2.812233000	4.017896000	-0.440863000
8	2.977780000	-0.607539000	3.750809000
6	2.235250000	-0.457107000	2.895520000
8	1.467899000	-0.301818000	2.014975000
8	2.443157000	-0.768813000	-0.786672000
6	3.231327000	-0.968130000	-1.642637000
8	4.004671000	-1.165769000	-2.458854000
8	-2.443148000	-0.768854000	-0.786646000
6	-3.231305000	-0.968140000	-1.642631000
8	-4.004637000	-1.165749000	-2.458867000
8	-1.467891000	-0.301800000	2.014983000
8	-2.977914000	-0.607440000	3.750708000
6	-2.235314000	-0.457048000	2.895473000
6	-2.174734000	3.098915000	-0.214317000
8	-1.517989000	2.149677000	0.030144000
8	-2.812156000	4.017923000	-0.440966000
8	0.000028000	-2.532114000	-0.167670000
6	0.000001000	-3.701474000	-0.323181000
8	-0.000025000	-4.833369000	-0.467936000

Frequency	Intensity	Frequency	Intensity	Frequency	Intensity
4.9684	0.0306	84.1008	0.7068	644.8551	0.122
18.3845	0.0237	91.9822	1.1796	646.0475	5.9994
19.3341	0.2082	94.8616	2.2758	646.8268	2.6286
22.8301	0.0008	97.3097	2.1171	647.48	7.4837
26.7447	0.1758	103.9135	1.726	649.2857	35.9867
27.83	0.033	107.9324	2.8877	649.8366	60.3101
28.7329	0.0052	119.9283	1.724	652.1661	50.929
28.9012	0.0041	121.2804	0.492	652.345	0.0071
30.2714	0.0059	125.0903	13.9491	654.3773	87.5548
32.107	0.0057	136.1594	3.4897	655.4043	38.688
34.7587	0.0047	138.2543	0.4113	656.0241	77.5225
43.3146	0.9806	140.6022	4.1314	656.913	47.4696
46.5109	1.782	156.8398	78.4787	657.8456	1.3974
47.0162	1.0941	165.036	6.8148	658.3664	15.9668
58.7313	0.0014	167.0193	62.3968	916.1029	189.2065
70.6505	2.4454	179.9302	1.1549	1363.3725	57.9753
72.0388	0.0032	209.0668	4.746	1363.8101	63.744

1368.3479	24.9056	1375.8132	2.5293	2430.7713	1468.7794
1369.8525	94.4684	2420.2615	0.1388	2432.9979	2324.5297
1370.2875	93.8052	2420.6784	492.7286	2436.2592	2419.0575
1371.5534	144.052	2421.2779	117.2561	2468.9777	0.3204

Table S160. Cartesian coordinates for the optimized geometry of isomer 7b-singlet $UO^{2+}(CO_2)_7$ followed by its predicted frequencies (cm^{-1}) and IR intensities (km/mol).

Z	x	y	z
92	0.128886000	0.242560000	-0.100236000
8	0.284862000	2.761920000	-0.154813000
6	0.310254000	3.943846000	-0.114786000
8	0.333992000	5.081413000	-0.077468000
8	-4.150997000	-0.901863000	-0.233977000
6	-5.230632000	-1.140843000	-0.629304000
8	-6.285083000	-1.373646000	-1.018619000
8	1.495100000	-1.744542000	-0.740155000
6	2.290010000	-2.618690000	-0.801086000
8	3.035203000	-3.475480000	-0.877656000
8	-0.573220000	0.257525000	-1.690717000
6	2.482381000	1.068118000	-1.300855000
8	3.405865000	1.418562000	-1.818348000
8	-1.214620000	-1.775780000	0.512920000
6	-1.972958000	-2.671323000	0.653622000
8	-2.685294000	-3.547547000	0.799826000
8	0.870478000	0.256949000	1.471215000
8	-4.118284000	1.683731000	1.645373000
8	-1.998014000	1.120499000	0.889350000
6	-3.082651000	1.397632000	1.268197000
8	6.109530000	-1.000716000	1.699181000
8	4.080857000	-0.841661000	0.581056000
6	5.106844000	-0.923805000	1.144947000

Frequency	Intensity	Frequency	Intensity	Frequency	Intensity
7.7211	0.1814	80.5118	0.2492	250.7633	4.6406
12.3105	0.155	85.7853	0.9726	274.0978	0.3326
14.3253	0.1433	91.1335	0.6996	629.2415	23.5421
15.3824	0.0359	101.006	0.0433	630.6772	47.2215
26.2005	0.1413	108.8722	0.8759	634.5855	43.7493
29.4819	0.261	120.985	0.7603	637.5286	63.1594
34.1787	0.1899	133.3047	1.0899	640.0993	14.4608
37.9963	0.2011	147.8164	0.3748	644.2406	79.7489
43.9443	0.0796	154.3421	0.9673	645.783	18.3432
44.8979	0.4893	156.9801	0.4428	648.01	8.2091
46.3373	0.2698	164.1105	3.164	665.5368	7.895
54.0139	1.5748	172.7365	0.4229	665.7369	58.1543
55.0728	0.2332	177.0237	7.9105	669.5463	27.31
57.0473	0.6315	183.3453	19.8656	671.2246	33.9618
64.9205	0.4665	189.3935	8.246	958.5807	0.5954
71.8918	0.9198	238.3282	73.7192	1037.3093	249.5978
74.951	0.4233	239.7359	74.8066	1364.2758	30.3598

1365.6982	28.5661	1379.1922	50.6514	2428.7454	3.2581
1369.6746	110.3066	2309.572	24.227	2436.0266	1018.1615
1370.9165	120.0729	2415.9746	2030.0385	2440.7826	2279.3872
1372.2735	165.5876	2418.1788	116.2049	2464.9898	346.8506

Table S161. Cartesian coordinates for the optimized geometry of isomer 7b-triplet $UO^{2+}(CO_2)_7$ followed by its predicted frequencies (cm^{-1}) and IR intensities (km/mol).

Z	x	y	z
92	0.133126000	0.243056000	-0.125000000
8	0.333844000	2.764400000	-0.147528000
6	0.380476000	3.945466000	-0.107631000
8	0.424768000	5.082641000	-0.069851000
8	-4.216807000	-0.892041000	-0.150354000
6	-5.316556000	-1.129002000	-0.486833000
8	-6.391015000	-1.359928000	-0.818504000
8	1.507942000	-1.744595000	-0.765153000
6	2.299704000	-2.619437000	-0.846709000
8	3.044285000	-3.475297000	-0.941595000
8	-0.568583000	0.288964000	-1.774897000
6	2.521756000	1.058631000	-1.291192000
8	3.458824000	1.399493000	-1.791103000
8	-1.236296000	-1.770877000	0.422309000
6	-1.987886000	-2.671199000	0.569923000
8	-2.693357000	-3.552186000	0.720134000
8	0.870535000	0.226831000	1.509784000
8	-4.089182000	1.750687000	1.626448000
8	-1.994563000	1.139843000	0.836326000
6	-3.066344000	1.441004000	1.233445000
8	6.043244000	-1.057930000	1.857833000
8	4.056289000	-0.884044000	0.669042000
6	5.061009000	-0.973598000	1.268480000

Frequency	Intensity	Frequency	Intensity	Frequency	Intensity
7.3217	0.1918	74.2443	0.5642	221.9521	75.2997
11.4462	0.1763	78.5974	0.1861	223.9272	69.8017
13.0905	0.0437	81.2466	0.4219	244.2072	2.9348
14.5337	0.1549	92.5202	0.4371	273.0138	0.1753
25.9206	0.195	102.8229	0.0477	629.7501	18.228
29.3739	0.2599	108.644	0.6287	631.5997	42.7672
33.7527	0.1447	119.9745	0.5674	635.4726	45.1292
37.1642	0.0613	131.3464	1.4738	638.8187	46.6078
43.7908	0.2552	141.7657	0.1641	640.6704	10.3522
44.6836	0.3481	149.8627	0.7049	644.6731	74.5247
46.2826	0.105	155.2646	1.0089	646.3066	18.4701
52.2225	0.1258	160.5237	1.8728	648.1937	9.8957
55.4017	0.3228	162.9648	0.0966	664.6006	25.857
56.3899	0.469	173.2917	6.223	665.6558	37.7051
61.8791	1.0108	180.1096	22.9769	668.5808	28.7331
68.3414	0.5829	184.6264	10.0164	671.3822	31.2472

689.3466	20.2446	1370.2908	110.5274	2416.6935	1017.9897
821.4174	0.0455	1371.166	179.3205	2426.5848	1.1876
1364.304	35.9675	1377.5876	97.0994	2433.5791	844.4613
1365.4015	33.4954	2305.921	24.2383	2437.4261	2333.915
1369.3106	99.164	2408.6959	943.9229	2458.7738	692.2563

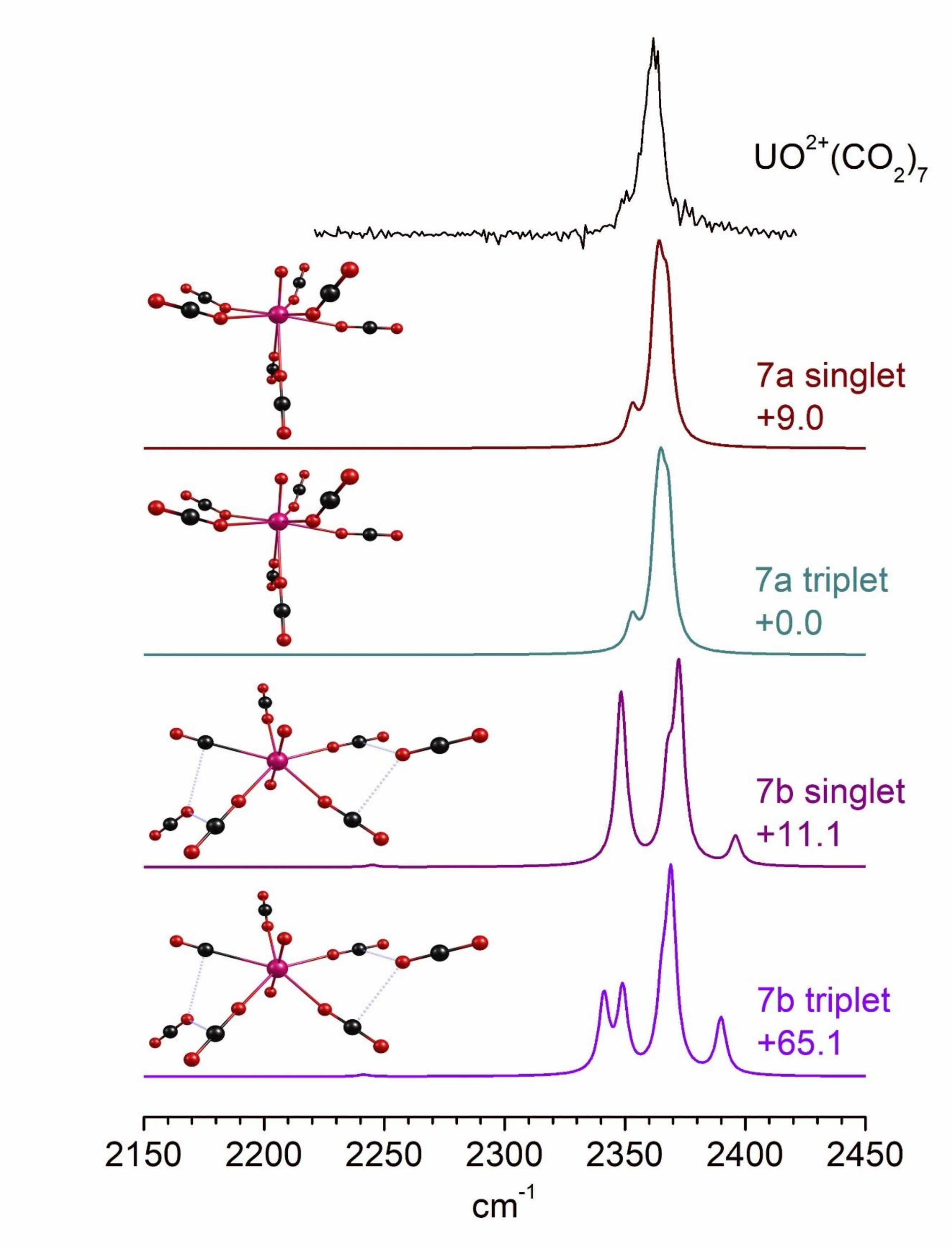

Figure S129. Experimental IR spectrum of $UO^{2+}(CO_2)_7$ compared with simulated spectra for 7a and 7b isomers. Relative energies (kcal/mol) are shown next to each spectrum.

Table S162. $UO^{2+}(CO_2)_8$ electronic energy calculated at the B3LYP/cc-pVTZ(-pp) level with Stuttgart/Koeln pseudopotential.

Isomer	2s + 1	Energy (hartree)	Rel. E (kcal/mol)	BDE (CO_2) (kcal/mol)	BDE (CO) (kcal/mol)
8a	1	-2058.791714	+9.1	5.1	
8a	3	-2058.806164	+0.0	4.4	
8b	1	-2058.790619	+9.8	5.2	
8b	3	-2058.804757	+0.9	4.2	
8c	1	-2058.78964	+10.4	5.8	3.1
8d	1	-2058.753786	+32.9		
8d	1	-2058.765646	+25.4		

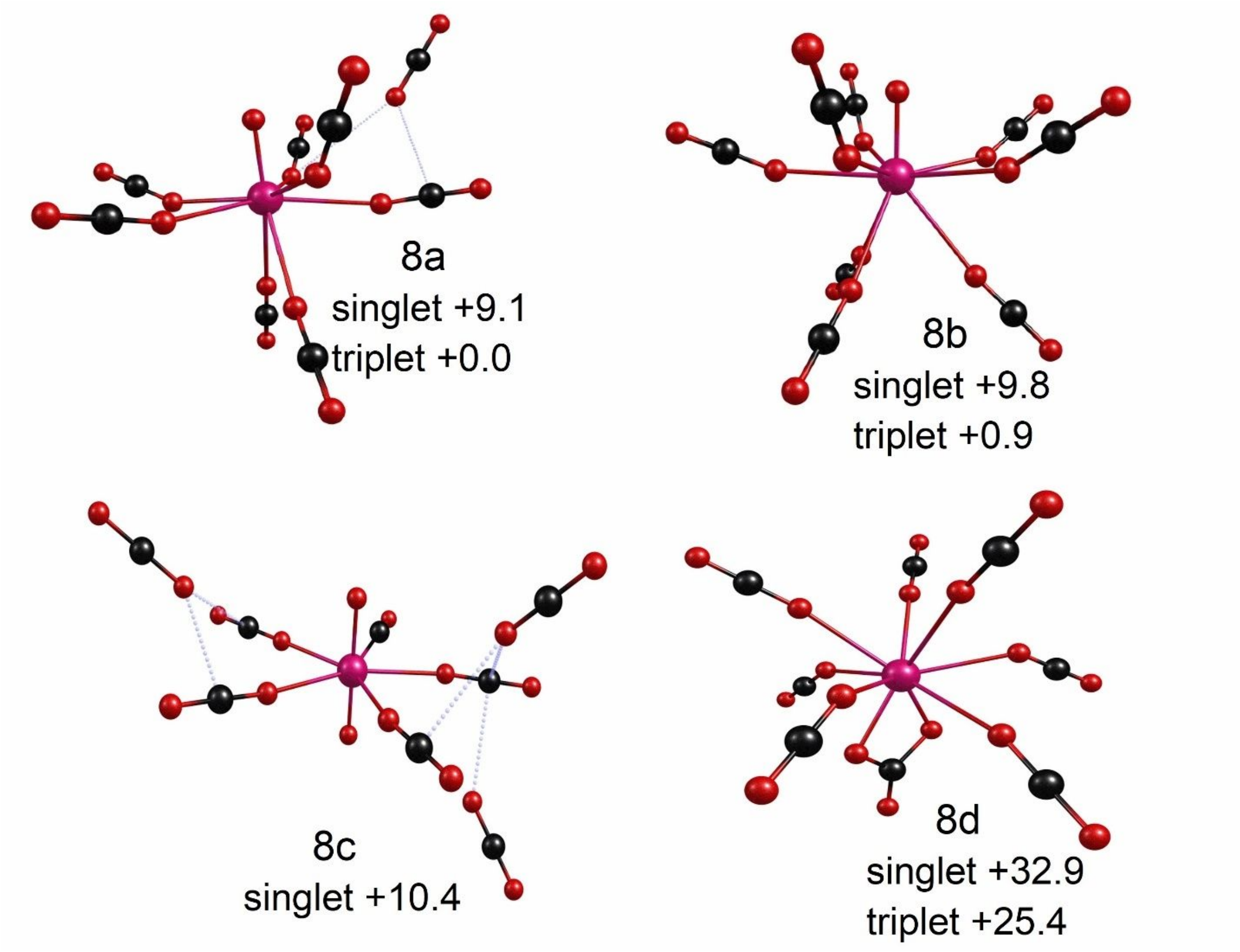

Figure S130. Predicted minimum energy structures of $UO^{2+}(CO_2)_8$ with energy of each spin state in kcal/mol. The lowest energy spin state of each isomer is shown.

Table S163. Cartesian coordinates for the optimized geometry of isomer 8a-singlet $UO^{2+}(CO_2)_8$ followed by its predicted frequencies (cm^{-1}) and IR intensities (km/mol).

Z	x	y	z
92	0.358087000	0.042683000	-0.181548000
8	-0.354723000	-0.028878000	-1.830669000
8	-1.526011000	1.806218000	0.138538000
6	-2.463003000	2.319873000	-0.361967000
8	-3.362540000	2.842800000	-0.831582000
8	-3.408068000	-2.000033000	2.148690000
6	-2.469710000	-1.535332000	1.694223000
8	-1.484744000	-1.067430000	1.246627000
8	0.666137000	1.400573000	2.162824000
6	0.701027000	2.090286000	3.116884000
8	0.736452000	2.758507000	4.043443000
8	1.440220000	2.339895000	-0.739657000
6	1.747526000	3.313931000	-1.329599000
8	2.050004000	4.260042000	-1.892018000
8	1.787447000	-1.289561000	1.726863000
8	2.969031000	-2.698583000	3.142481000
6	2.386668000	-2.005547000	2.444456000
6	3.636281000	-0.585744000	-1.872391000
8	2.736267000	-0.386466000	-1.136578000
8	4.513224000	-0.779214000	-2.576935000
8	0.469483000	-2.542947000	-0.641696000
6	0.176195000	-3.367764000	-1.433654000
8	-0.100962000	-4.178067000	-2.188652000
6	-5.112027000	-0.400725000	-0.867079000
8	-4.049244000	-0.237298000	-0.399158000
8	-6.152191000	-0.562147000	-1.327877000

Frequency	Intensity	Frequency	Intensity	Frequency	Intensity
7.8898	0.0567	43.4718	1.3842	107.5978	1.8109
8.5511	0.0031	50.9148	0.5158	112.1502	2.8103
16.7509	0.0447	52.1148	2.1067	120.8154	1.934
19.2466	0.1601	55.5993	0.9069	123.1141	11.647
22.6287	0.0004	61.1243	0.0306	126.6055	2.7615
25.6172	0.1677	69.3222	2.0679	135.4631	2.8859
27.4058	0.0594	70.7025	0.3681	139.4319	0.9953
28.2135	0.0237	78.2223	0.9994	140.91	2.1139
29.1106	0.0076	91.9787	0.9939	161.2427	80.0753
30.6932	0.0223	92.9782	1.347	163.4647	9.6974
33.3229	0.0117	95.9449	1.6977	170.9637	62.9702
35.8484	0.0793	96.8562	2.5011	185.3683	0.5524
40.6571	0.1215	103.1017	0.3417	214.1195	5.1052

638.4609	29.4625	657.358	47.9684	1370.8614	91.3719
644.9039	11.4739	657.6008	30.6574	1372.4249	126.2566
646.2968	10.1045	658.4122	9.4307	1376.4974	3.6426
648.3359	24.4317	666.6468	39.8668	2415.7107	826.4909
648.6557	20.2882	671.2375	31.8406	2420.5684	293.7059
649.6531	27.7398	917.2974	192.9904	2421.0645	721.2892
650.9857	80.4818	1363.0424	56.8664	2421.1303	41.3596
652.3971	0.4142	1365.5198	66.861	2430.4211	1349.8849
653.1307	39.9543	1366.1629	14.5961	2432.5969	2259.0558
655.1317	39.6162	1369.0651	13.2119	2436.4014	2220.5332
655.5355	74.1669	1370.2891	106.5552	2469.472	2.836

Table S164. Cartesian coordinates for the optimized geometry of isomer 8a-triplet $UO^{2+}(CO_2)_8$ followed by its predicted frequencies (cm^{-1}) and IR intensities (km/mol).

Z	x	y	z
92	0.339341000	0.046854000	-0.173080000
8	-0.406402000	0.013327000	-1.810083000
8	-1.549360000	1.773550000	0.323813000
6	-2.520155000	2.330085000	-0.048777000
8	-3.451486000	2.893111000	-0.394288000
8	-3.386002000	-2.147099000	2.072693000
6	-2.455005000	-1.649582000	1.638987000
8	-1.476534000	-1.148975000	1.212259000
8	0.857760000	1.396876000	2.133640000
6	1.001104000	2.084304000	3.079490000
8	1.141176000	2.750054000	3.997593000
8	1.338007000	2.376141000	-0.788322000
6	1.552753000	3.328685000	-1.450008000
8	1.766785000	4.255383000	-2.081347000
8	1.777984000	-1.351585000	1.689182000
8	2.938883000	-2.851901000	3.025925000
6	2.366708000	-2.113899000	2.366640000
6	3.649235000	-0.464413000	-1.859340000
8	2.726010000	-0.302510000	-1.143480000
8	4.547858000	-0.621738000	-2.545271000
8	0.501673000	-2.536975000	-0.724938000
6	0.220268000	-3.341673000	-1.540991000
8	-0.045517000	-4.132420000	-2.320922000
6	-5.115314000	-0.376720000	-0.837344000
8	-4.056118000	-0.234740000	-0.354525000
8	-6.151836000	-0.516914000	-1.313008000

Frequency	Intensity	Frequency	Intensity	Frequency	Intensity
3.2859	0.0195	43.2735	1.3545	106.2674	1.8974
8.6256	0.011	49.8182	0.5145	111.908	2.9767
15.8467	0.0936	52.3129	1.9009	121.089	0.9082
19.3869	0.1056	55.9368	1.2842	123.8754	13.7594
22.4084	0.0132	58.5644	0.0227	128.0188	1.9958
25.3268	0.156	69.3235	1.8236	136.9273	4.8192
27.4698	0.0659	72.0762	0.2748	139.3807	0.9932
27.9482	0.0167	77.4043	1.0897	142.3863	2.4293
28.9623	0.0049	90.3168	1.2966	157.7002	77.4871
30.6563	0.0224	91.7082	0.9763	165.2534	13.7354
32.8999	0.0061	95.0516	2.5006	170.6919	57.0506
35.9597	0.0878	95.5747	2.0648	178.9701	2.4195
40.6793	0.0806	103.4258	0.4493	207.6784	4.4357

638.513	29.6559	656.8131	34.3474	1371.0341	74.3625
645.5569	17.8774	657.8295	28.9378	1372.4619	133.7648
646.6099	5.3583	658.7765	23.054	1376.6275	3.8739
648.1213	34.0543	666.488	39.0438	2415.8535	808.3476
648.7527	7.8697	671.2261	31.8715	2420.5927	255.5506
649.8504	15.0051	915.7226	190.375	2421.1017	727.1125
650.7822	73.246	1363.2791	56.8074	2421.6964	47.6325
652.7685	21.2037	1366.2167	27.6519	2430.8756	1335.8459
653.6148	56.0392	1367.1854	42.1521	2433.1469	2290.042
655.4821	25.2101	1368.8003	14.7988	2436.848	2156.1125
655.6718	77.1365	1369.9257	127.9171	2469.4601	3.568

Table S165. Cartesian coordinates for the optimized geometry of isomer 8b-singlet $UO^{2+}(CO_2)_8$ followed by its predicted frequencies (cm^{-1}) and IR intensities (km/mol).

Z	x	y	z
92	-0.006168000	-0.001274000	-0.265318000
8	-0.135961000	0.000076000	-2.061601000
8	1.917424000	0.017688000	1.785742000
6	2.788129000	0.036776000	2.574652000
8	3.635790000	0.054777000	3.344085000
8	-1.300769000	3.194560000	3.402506000
6	-0.966340000	2.381096000	2.670998000
8	-0.621834000	1.541584000	1.922439000
8	-0.590267000	-1.557034000	1.921239000
6	-0.904261000	-2.394111000	2.685699000
8	-1.209258000	-3.205484000	3.432244000
8	2.025223000	-1.618191000	-0.657211000
6	2.784086000	-2.262784000	-1.287878000
8	3.524745000	-2.891002000	-1.889206000
8	-2.618712000	-0.028562000	0.033072000
8	-4.921606000	-0.063500000	-0.249313000
6	-3.787223000	-0.046359000	-0.113721000
6	-1.249123000	-3.139944000	-1.706610000
8	-0.926867000	-2.458860000	-0.799194000
8	-1.564675000	-3.811864000	-2.575222000
8	1.989968000	1.660561000	-0.654120000
6	2.727247000	2.323612000	-1.291315000
8	3.447068000	2.969825000	-1.898831000
6	-1.320501000	3.107354000	-1.706520000
8	-1.653294000	3.767164000	-2.577981000
8	-0.980048000	2.438688000	-0.796472000

Frequency	Intensity	Frequency	Intensity	Frequency	Intensity
15.1977	0.0284	37.1971	0.3267	116.5609	8.94
17.6846	0.2564	43.9795	1.4314	117.4062	1.1583
18.6915	0.0049	50.9972	0.1157	125.5938	5.0191
19.7251	0.3871	54.8373	0.8745	132.0632	0.3396
24.3872	0.3059	71.4424	0.0085	133.452	21.1447
25.5618	0.0434	76.3573	2.366	134.6765	0.1598
26.5613	0.0126	79.5316	0.0029	147.0058	5.3917
27.5899	0.0378	90.0682	0.2414	149.081	43.999
27.9158	0.0007	93.4744	0.1107	152.3737	63.5461
30.1576	0.0152	100.0697	4.2341	174.2258	1.4041
33.109	0	103.4982	5.8496	188.9403	7.1864
34.308	0.4602	108.0033	15.8189	231.4153	9.4402
36.6713	0.0031	108.3466	0.6194	646.9279	4.5787

647.0262	0.9387	659.075	102.0816	1373.2652	112.5434
647.9211	2.9883	660.2036	2.925	1376.9966	9.8628
649.4602	20.185	661.3564	0.6286	2414.0681	1.7942
650.1922	5.1832	661.5457	11.1535	2415.7641	163.6072
650.9764	2.4045	910.6648	198.9622	2420.6416	117.6043
652.8681	19.0263	1362.4344	34.8079	2423.218	98.6369
653.1772	64.3024	1363.2415	50.4083	2431.6035	2249.9904
655.0168	137.1496	1369.52	51.5726	2431.7843	2305.8686
656.1173	10.8914	1370.5556	81.8659	2435.3872	2647.8927
657.3899	3.7132	1371.4365	58.1386	2470.8084	0.0875
657.7788	90.876	1371.7487	39.5242		

Table S166. Cartesian coordinates for the optimized geometry of isomer 8b-triplet $UO^{2+}(CO_2)_8$ followed by its predicted frequencies (cm^{-1}) and IR intensities (km/mol).

Z	x	y	z
92	0.010662000	0.001339000	-0.207534000
8	-0.055108000	-0.001339000	-2.008859000
8	2.047888000	1.628244000	-0.600337000
6	2.774955000	2.263393000	-1.276475000
8	3.485602000	2.883194000	-1.921348000
8	-1.634764000	3.106279000	3.398663000
6	-1.226530000	2.313900000	2.682189000
8	-0.805916000	1.495810000	1.948205000
8	1.818256000	0.048809000	1.894943000
6	2.686009000	0.073771000	2.688267000
8	3.528780000	0.097966000	3.461720000
8	2.096472000	-1.564414000	-0.582268000
6	2.850274000	-2.185977000	-1.241523000
8	3.586752000	-2.792220000	-1.870053000
8	-0.728012000	-1.533740000	1.946014000
8	-1.480729000	-3.188024000	3.388664000
6	-1.109998000	-2.374089000	2.676020000
6	-1.084616000	-3.199846000	-1.688724000
8	-0.822953000	-2.501970000	-0.775461000
8	-1.341422000	-3.887528000	-2.564998000
8	-0.901707000	2.474687000	-0.770749000
6	-1.187745000	3.170476000	-1.678289000
8	-1.468487000	3.855952000	-2.548885000
6	-3.729090000	-0.066903000	-0.600340000
8	-4.813005000	-0.088385000	-0.960034000
8	-2.614202000	-0.044761000	-0.219416000

Frequency	Intensity	Frequency	Intensity	Frequency	Intensity
15.8763	0.016	37.2517	0.578	115.4315	0.2169
17.022	0.0122	42.8027	1.1374	120.2168	13.1614
19.194	0.3309	47.0562	0.1891	128.006	3.0591
20.5604	0.2311	59.989	1.55	130.9669	7.6396
24.8086	0.153	72.7183	0.0073	135.2926	0.5434
26.0358	0.0165	73.9714	0.4836	139.1869	23.2369
26.842	0.0216	86.2263	2.8437	146.8879	5.0679
27.6796	0.0233	86.6804	0.0042	148.7739	38.2006
27.8025	0.0776	94.4427	2.7383	155.5091	64.9219
30.0737	0.0143	98.6202	2.1885	173.6936	1.0921
31.2581	0.0001	102.0653	5.6301	195.219	21.9422
35.0964	0.6962	109.9138	0.4237	229.7023	15.9759
37.0081	0.0022	110.6406	4.0776	647.8304	0.1506

648.7132	3.2194	659.7625	82.5351	1372.0878	124.4519
649.6306	0.1939	660.9951	14.4935	1376.3374	10.4885
650.2653	0.0375	662.5192	6.5506	2414.289	0.4195
650.3324	9.5813	662.5849	4.5639	2414.9365	122.7842
651.6729	0.0183	905.5031	188.9041	2421.2685	51.9764
653.9493	95.7814	1363.566	32.0599	2422.5984	62.4379
654.8071	47.8085	1363.9574	38.2819	2430.5622	2240.5726
656.0984	70.8631	1368.6372	47.9582	2431.9246	2460.1134
656.77	98.5984	1369.7761	91.2144	2434.1391	2586.9198
657.0089	4.8694	1370.4603	65.4437	2469.9024	6.0672
657.5345	42.4409	1370.9081	42.6922		

Table S167. Cartesian coordinates for the optimized geometry of isomer 8b-triplet $UO^{2+}(CO_2)_8$ followed by its predicted frequencies (cm^{-1}) and IR intensities (km/mol).

Z	x	y	z
92	-0.298875000	-0.459938000	0.069068000
8	-2.632207000	-1.380232000	0.162775000
6	-3.775417000	-1.677687000	0.203423000
8	-4.870626000	-1.986140000	0.246376000
8	-1.708719000	1.340455000	1.083636000
6	-2.426658000	2.207992000	1.440228000
8	-3.102897000	3.051277000	1.799128000
8	3.673074000	-0.162218000	1.736626000
6	4.538268000	-0.288470000	2.519471000
8	5.382880000	-0.413247000	3.287437000
8	-0.003663000	-1.136655000	1.643316000
6	-0.393463000	-3.039310000	-0.957778000
8	-0.426498000	-4.075137000	-1.369470000
8	1.228602000	1.442772000	0.553510000
6	2.136230000	2.179403000	0.721618000
8	2.992532000	2.910254000	0.893975000
8	-0.612682000	0.153841000	-1.527509000
8	-6.497950000	2.064927000	-1.107065000
8	-4.528640000	1.126327000	-0.311439000
6	-5.524925000	1.600586000	-0.712341000
8	4.200024000	-1.519904000	-1.168151000
8	1.952949000	-1.222352000	-0.673786000
6	3.100344000	-1.360339000	-0.920064000
6	3.696642000	1.960033000	-2.829379000
8	3.088868000	1.408474000	-1.990625000
8	4.288750000	2.500188000	-3.651894000

Frequency	Intensity	Frequency	Intensity	Frequency	Intensity
6.5825	0.0678	48.2987	0.6171	94.7563	0.4226
10.416	0.0622	51.399	0.9192	103.8918	0.071
12.7423	0.184	55.3426	0.5511	117.1133	1.9389
14.7712	0.0592	55.9317	1.1344	120.9028	0.402
20.3622	0.0421	66.0228	0.1522	137.3896	0.2015
22.3953	0.2303	67.9007	0.6111	152.1566	0.0642
33.0526	0.2755	72.1958	1.6622	154.283	0.8043
36.5898	0.0506	74.1503	0.5875	161.6975	0.2328
41.4101	0.0154	74.5113	0.438	164.5857	0.712
43.9642	0.3508	74.8579	0.0923	175.0817	0.2254
44.8309	0.0851	79.9306	0.3611	178.2475	6.8063
45.9749	0.6968	84.7277	0.4621	184.9332	22.7039

193.004	12.3078	645.8899	14.8046	1372.457	55.9296
239.3306	59.9595	665.2927	14.4013	1373.4469	71.4048
240.3789	97.2631	665.8317	51.8568	1374.3461	272.2142
246.0659	1.887	666.6395	28.7222	1381.519	36.8737
274.693	0.0099	670.3496	0.1891	2309.0025	30.2374
627.4226	1.9153	671.0809	21.371	2413.2549	952.6405
629.3726	20.7129	671.4725	73.374	2416.3197	1731.473
632.9744	33.9249	956.8746	0.7637	2421.4619	476.8312
634.3075	101.1691	1035.4936	250.159	2429.2726	28.7925
635.8604	85.7458	1364.6388	30.82	2435.148	869.7092
640.5257	47.8208	1365.1923	21.8464	2444.298	2195.7324
643.2844	27.9198	1365.5402	22.4217	2466.7673	325.1882

Table S168. Cartesian coordinates for the optimized geometry of isomer 8d-singlet $UO^{2+}(CO_2)_8$ followed by its predicted frequencies (cm^{-1}) and IR intensities (km/mol).

Z	x	y	z
92	0.024698000	-0.000069000	-0.113630000
6	-0.552121000	-0.003061000	-2.697407000
8	-0.386965000	-1.093360000	-1.863250000
8	-0.774830000	-0.004267000	-3.849867000
8	-0.386615000	1.089163000	-1.865678000
6	2.585103000	2.305739000	1.484799000
8	1.798060000	1.526501000	1.078834000
8	3.347011000	3.056801000	1.881622000
8	3.348082000	-3.047414000	1.895079000
6	2.586067000	-2.298284000	1.494809000
8	1.798922000	-1.521029000	1.085274000
8	-4.772949000	-0.003690000	-0.773308000
6	-3.695065000	-0.002395000	-0.399399000
8	-2.584841000	-0.001039000	0.002149000
8	-1.666098000	0.005930000	4.542022000
6	-1.194126000	0.004393000	3.502772000
8	-0.702253000	0.002801000	2.430777000
8	-0.884372000	-2.387316000	0.541252000
6	-1.329922000	-3.386108000	0.093685000
8	-1.763536000	-4.356517000	-0.320726000
8	-1.767856000	4.354284000	-0.330150000
6	-1.333467000	3.385118000	0.086362000
8	-0.887132000	2.387646000	0.536073000
8	3.638838000	-0.006056000	-3.262558000
6	2.984680000	-0.004303000	-2.328627000
8	2.324143000	-0.002476000	-1.348548000

Frequency	Intensity	Frequency	Intensity	Frequency	Intensity
5.3113	0.5734	42.4645	2.9618	134.8163	15.5585
20.0612	0.0014	48.8099	0.0066	141.7628	8.2011
21.6593	0.0064	64.9088	1.4529	149.7089	0.0272
22.7753	0.2496	81.0501	0.9027	151.394	5.5471
24.529	0.8667	84.1406	0.3803	155.4946	5.6119
27.5531	0.7417	90.1451	0.4029	157.7661	14.1944
28.5683	0.0017	94.3661	1.8854	163.7605	57.3256
29.7069	0.0906	103.0713	2.5006	178.5241	7.4484
33.4029	0.3261	115.4442	0.3626	185.9771	15.4449
34.3046	0.6001	115.6607	5.5108	204.7264	1.545
34.6958	0.004	116.741	2.9648	277.0541	1.8317
38.0138	1.8628	120.538	4.134	377.5276	44.5621
40.9843	0.577	124.4606	5.2812	614.8859	7.195

643.0778	0.0012	655.7416	8.3283	1370.1903	126.676
643.7549	3.5388	658.0329	51.5734	1370.5384	83.6755
645.6694	11.0817	658.3433	23.7369	1375.7075	45.8486
646.4585	13.1601	777.4251	163.6945	1927.1741	577.8294
647.7094	21.1644	779.2839	54.1419	2417.7945	156.4006
648.9893	18.8122	917.4028	134.1293	2420.2715	228.4713
649.6137	33.0801	981.2292	237.1071	2424.7356	35.6018
650.0688	64.4413	1360.6236	94.6425	2429.2819	1673.6997
652.4211	49.4287	1361.0449	22.3071	2432.8277	2048.4063
654.2471	92.1836	1364.6382	74.8521	2437.4418	2542.6396
655.5543	31.8634	1368.3479	83.5843	2468.6179	63.4648

Table S169. Cartesian coordinates for the optimized geometry of isomer 8d-triplet $UO^{2+}(CO_2)_8$ followed by its predicted frequencies (cm^{-1}) and IR intensities (km/mol).

Z	x	y	z
92	-0.026238000	0.000074000	-0.111218000
6	0.531512000	0.003579000	-2.699502000
8	0.369283000	1.094413000	-1.865422000
8	0.750595000	0.004930000	-3.852760000
8	0.368901000	-1.089507000	-1.868183000
6	-2.578200000	-2.311974000	1.490730000
8	-1.791447000	-1.532511000	1.084569000
8	-3.339725000	-3.063245000	1.887823000
8	-3.341360000	3.051385000	1.904426000
6	-2.579659000	2.302572000	1.503027000
8	-1.792734000	1.525641000	1.092399000
8	4.769690000	0.004849000	-0.809881000
6	3.694377000	0.003224000	-0.428608000
8	2.586923000	0.001516000	-0.019573000
8	1.694997000	-0.006965000	4.535047000
6	1.217612000	-0.005118000	3.498286000
8	0.720210000	-0.003203000	2.428838000
8	0.892640000	2.385532000	0.539287000
6	1.335900000	3.385190000	0.091441000
8	1.767443000	4.356381000	-0.323300000
8	1.773407000	-4.353425000	-0.334241000
6	1.340800000	-3.383752000	0.082951000
8	0.896458000	-2.385711000	0.533307000
8	-3.658501000	0.007157000	-3.241615000
6	-3.000659000	0.005042000	-2.310234000
8	-2.336310000	0.002835000	-1.332783000

Frequency	Intensity	Frequency	Intensity	Frequency	Intensity
2.6774	0.5892	42.5142	2.9579	135.0743	15.3266
20.1528	0.0028	47.9238	0.0037	141.6156	8.1899
21.592	0.0065	64.6849	1.4185	149.8508	0.048
22.5447	0.2689	79.7622	0.7347	150.8246	5.0223
24.4218	0.88	83.7049	0.364	155.1046	6.5288
27.4334	0.7721	89.6555	0.5679	157.6779	14.9686
28.5441	0.0018	94.6816	1.7419	163.0827	57.8817
29.5974	0.0937	102.6361	2.4063	177.8065	7.2765
33.2727	0.3385	114.9929	0.7382	185.8278	15.5052
34.2836	0.6335	115.4543	6.1403	203.6807	1.4145
34.6728	0.0041	116.2483	2.8346	271.7469	1.4597
37.8871	1.8364	120.8032	3.3189	375.9326	44.2329
40.7535	0.5348	124.3541	5.5053	613.7571	7.977

643.1295	0.001	655.7539	8.9832	1370.2344	129.0048
643.8487	3.5653	657.9837	51.8776	1370.5908	84.1305
645.7517	11.2798	658.328	22.8356	1375.7314	45.8881
646.5597	13.2699	776.5268	182.4322	1926.1688	575.5452
647.7463	21.6737	778.1475	32.0327	2417.8963	159.4948
649.0096	18.6351	916.3029	133.3427	2420.4013	223.92
649.748	23.9429	977.5446	239.8242	2424.7105	39.4882
650.1009	73.6077	1360.7502	94.5803	2429.3202	1663.0085
652.4813	49.2995	1361.1782	22.4172	2432.9021	2053.7407
654.4288	95.5285	1364.6344	73.8177	2437.5242	2540.7985
655.6337	28.2822	1368.4437	82.6726	2468.6015	62.7183

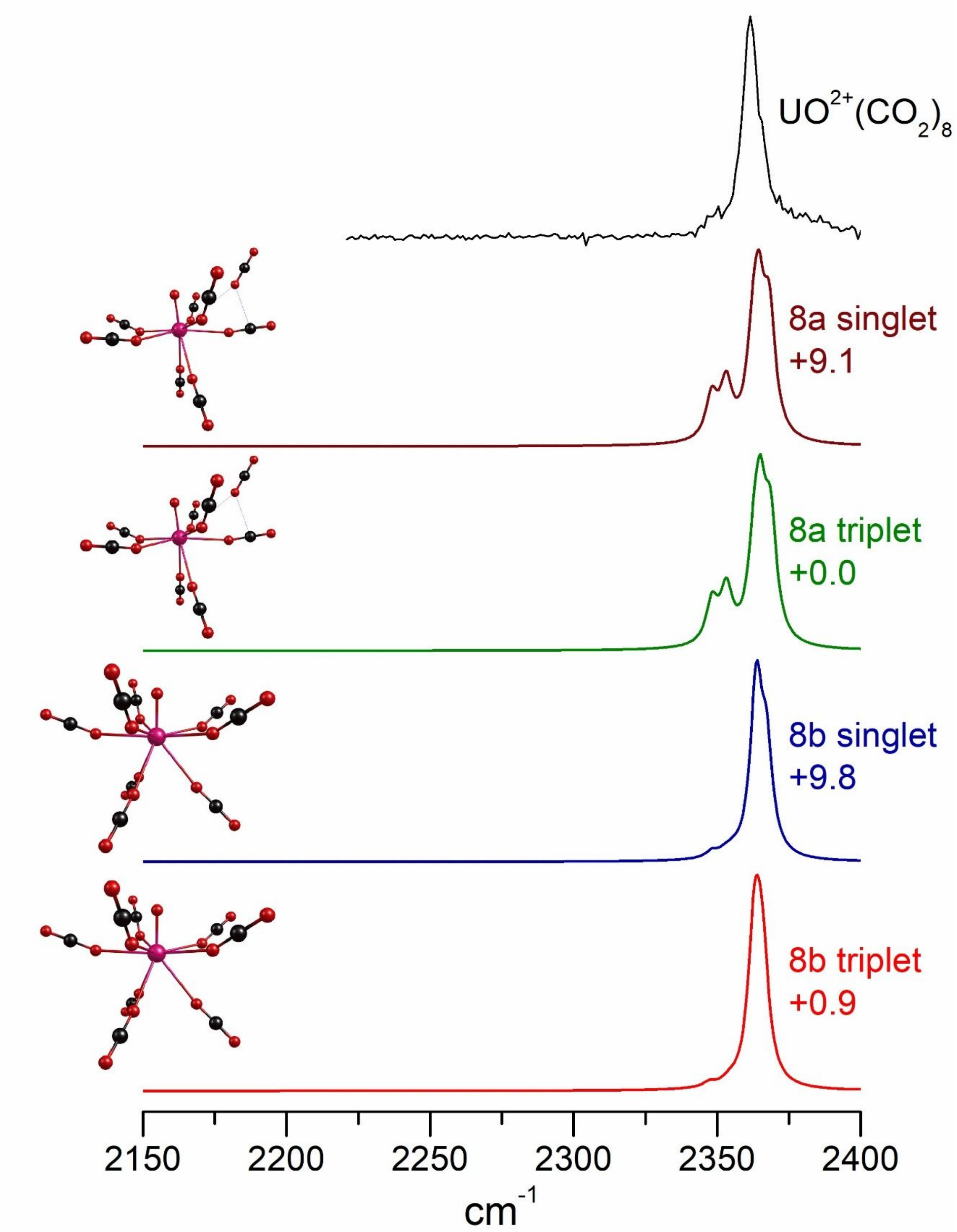

Figure S131. Experimental IR spectrum of $UO^{2+}(CO_2)_8$ compared with simulated spectra for isomers 8a and 8b. Relative energies (kcal/mol) are shown next to each spectrum.

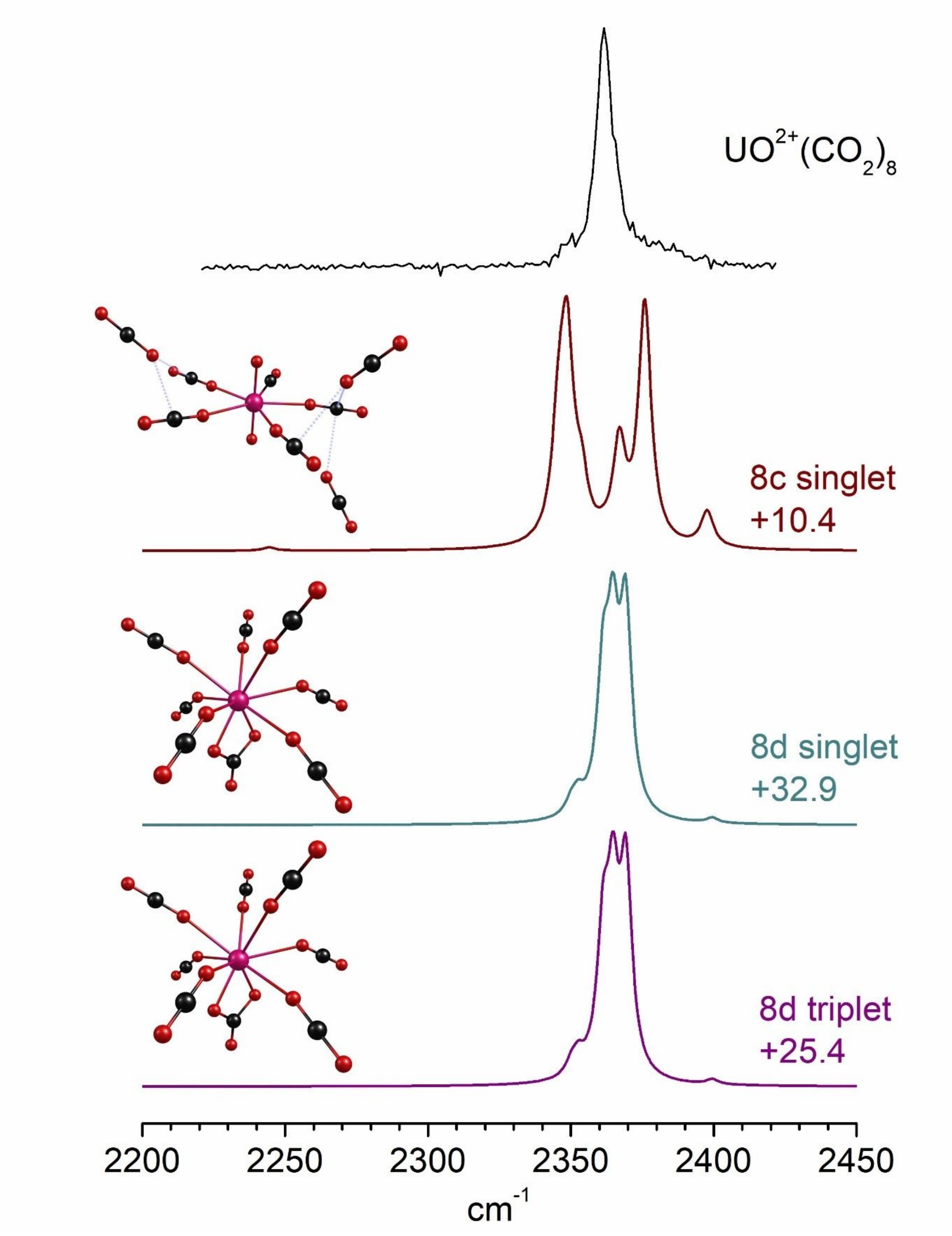

Figure S132. Experimental IR spectrum of $UO^{2+}(CO_2)_8$ compared with simulated spectra for isomers 8c and 8d. Relative energies (kcal/mol) are shown next to each spectrum.

Table S170. $UO^{2+}(CO_2)_9$ electronic energy calculated at the B3LYP/cc-pVTZ(-pp) level with Stuttgart/Koeln pseudopotential.

Isomer	2s + 1	Energy (hartree)	Rel. E (kcal/mol)	BDE (CO_2) (kcal/mol)	BDE (CO) (kcal/mol)
9a	1	-2247.448644	+9.0	5.1	
9a	3	-2247.46304	+0.0	5.0	
9b	1	-2247.448139	+9.4	4.7	
9b	3	-2247.462598	+0.3	4.7	
9c	1	-2247.447715	+9.6	4.5	
9c	3	-2247.46204	+0.6	4.3	
9d	3	-2247.460956	+1.3	3.7	
9f	1	-2247.445408	+11.6	4.3	
9f	3	-2247.359044	+65.3		

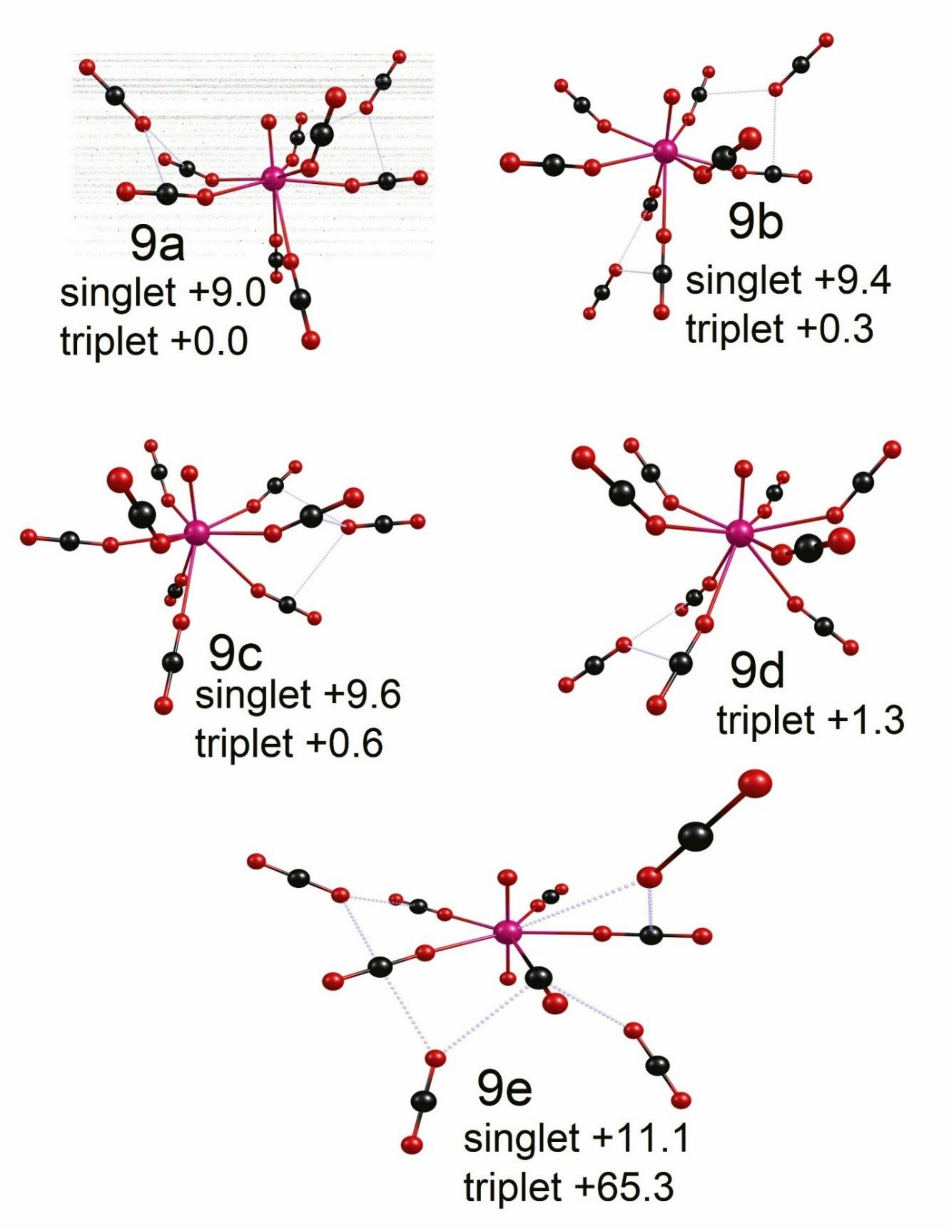

Figure S133. Predicted minimum energy structures of $UO^{2+}(CO_2)_9$ with energy of each spin state in kcal/mol. The lowest energy spin state of each isomer is shown.

Table S171. Cartesian coordinates for the optimized geometry of isomer 9a-singlet $UO^{2+}(CO_2)_9$.

Z	x	y	z
92	-0.035796000	0.221188000	0.032221000
8	-0.111488000	-1.112778000	-1.171423000
8	1.339308000	-1.566125000	1.336887000
6	1.794945000	-2.652649000	1.280460000
8	2.230050000	-3.708056000	1.255542000
8	4.731400000	1.120147000	-0.531246000
6	3.611913000	0.996714000	-0.343685000
8	2.454862000	0.898137000	-0.143966000
8	0.475081000	1.025604000	2.593343000
6	0.722415000	1.210826000	3.729383000
8	0.961991000	1.392786000	4.832328000
8	-1.720037000	-0.962870000	1.592224000
6	-2.506206000	-1.744918000	1.991928000
8	-3.257001000	-2.498257000	2.406755000
8	-0.082460000	2.930619000	0.421669000
8	-0.159064000	5.249574000	0.384388000
6	-0.121288000	4.106919000	0.401418000
6	-3.477823000	1.156478000	-1.029625000
8	-2.397118000	1.076838000	-0.565746000
8	-4.521796000	1.263469000	-1.478899000
8	0.250514000	1.568716000	-2.212422000
6	0.328860000	1.419005000	-3.380349000
8	0.406604000	1.291722000	-4.512529000
6	4.579082000	-2.590833000	-1.353748000
8	3.809014000	-1.886576000	-0.819386000
8	5.332211000	-3.281392000	-1.879798000
8	-5.499553000	-3.203500000	-1.495470000
6	-4.744402000	-2.467281000	-1.039045000
8	-3.971489000	-1.717418000	-0.575343000

Frequency	Intensity	Frequency	Intensity	Frequency	Intensity
7.2077	0.0462	35.1029	0.0011	71.4784	2.3067
7.7003	0.0701	39.3962	0.1952	73.9178	0.4226
8.6785	0.0031	39.9718	0.4429	79.8335	1.14
17.3785	0.0593	41.3393	0.0736	91.0437	0.0509
19.4496	0.0567	46.9153	1.7303	93.6435	0.8526
19.9054	0.0734	52.5496	0.8281	95.9153	1.4863
24.6739	0.1931	53.3188	0.8797	97.5294	4.3683
27.3855	0.0153	53.644	0.1378	102.9219	0.3038
27.8034	0.0315	61.0402	1.23	107.6642	1.8074
28.9347	0.0134	63.0214	0.4473	109.5416	0.0808
31.293	0.0108	69.7439	0.6137	114.9103	3.9368

121.563	13.4185	649.4586	12.1159	1366.4504	10.184
125.3251	1.3592	650.9728	62.8304	1366.5243	30.4172
127.7258	1.4847	652.7195	0.4781	1369.8196	32.675
139.1835	2.42	652.8622	17.9397	1371.7413	74.8159
140.4066	3.1901	655.1384	42.4286	1371.9168	67.6283
141.5933	1.2195	655.9791	78.4172	1373.4997	148.4467
162.9237	72.6766	657.8634	52.303	1377.479	1.1996
164.8311	2.2082	658.177	32.9329	2415.1091	1272.9078
171.747	78.52	658.5574	4.04	2416.4197	439.342
185.5737	1.3777	666.0861	36.3986	2420.5818	174.3152
214.2179	5.1331	666.8245	43.4512	2421.2224	112.7569
637.0217	12.9937	671.2496	11.7916	2421.9566	1306.867
639.0787	46.0082	671.5545	51.4559	2430.4682	1099.9425
643.4378	62.3101	916.6771	193.8257	2432.7965	2132.0093
647.1355	23.957	1363.4195	54.507	2437.4622	1985.1444
648.8646	14.3181	1365.9415	60.5067	2470.055	3.7096

Table S172. Cartesian coordinates for the optimized geometry of isomer 9a-triplet $UO^{2+}(CO_2)_9$ followed by its predicted frequencies (cm^{-1}) and IR intensities (km/mol).

Z	x	y	z
92	-0.026069000	0.222421000	0.043607000
8	-0.091548000	-1.118976000	-1.154144000
8	1.407297000	-1.503266000	1.386608000
6	1.906889000	-2.571260000	1.390480000
8	2.384557000	-3.608185000	1.423335000
8	4.720210000	1.144551000	-0.614274000
6	3.605542000	1.016382000	-0.404131000
8	2.452779000	0.913627000	-0.181478000
8	0.363330000	1.051372000	2.615752000
6	0.554128000	1.252855000	3.760104000
8	0.738493000	1.450427000	4.870751000
8	-1.695295000	-1.034728000	1.571147000
6	-2.449708000	-1.868965000	1.923665000
8	-3.170304000	-2.673623000	2.293432000
8	-0.087940000	2.937919000	0.419839000
8	-0.170826000	5.255390000	0.334237000
6	-0.129838000	4.113493000	0.375021000
6	-3.496642000	1.137958000	-0.960115000
8	-2.406815000	1.053593000	-0.519049000
8	-4.549331000	1.249624000	-1.387295000
8	0.218204000	1.581919000	-2.217640000
6	0.279972000	1.443000000	-3.387510000
8	0.341155000	1.324507000	-4.521984000
6	4.563861000	-2.584139000	-1.373304000
8	3.809589000	-1.870222000	-0.829318000
8	5.301190000	-3.284337000	-1.908885000
8	-5.433105000	-3.245768000	-1.599882000
6	-4.693718000	-2.507618000	-1.121207000
8	-3.937215000	-1.755451000	-0.634883000

Frequency	Intensity	Frequency	Intensity	Frequency	Intensity
4.6648	0.0465	30.9018	0.0061	62.3153	1.084
7.4179	0.0259	34.994	0.0009	69.9958	1.7821
8.7584	0.0073	39.3509	0.2065	71.4181	1.0228
16.44	0.1075	39.9032	0.4392	74.1236	0.4328
19.4472	0.0635	41.174	0.0981	79.2775	1.15
19.936	0.0311	46.5646	1.5691	90.9738	0.6709
24.1744	0.1992	51.8584	0.3041	92.0435	1.1129
27.2164	0.0108	52.834	0.9752	94.5734	1.371
27.7419	0.0399	53.4379	0.6001	96.774	3.8445
28.8416	0.0149	60.6421	0.6296	103.1712	0.136

106.5429	2.2184	647.3698	25.8761	1366.4479	29.1825
109.6118	0.1206	648.9609	21.2122	1366.584	18.9222
114.954	4.1212	649.1742	4.5705	1367.1548	48.0834
122.3389	13.9587	651.1721	67.5851	1369.6779	37.5716
125.938	0.8672	652.3602	17.8776	1371.6247	84.109
128.4481	1.985	653.1045	3.9691	1372.0668	57.7635
139.4448	2.5236	655.3979	38.4668	1373.5775	152.7452
140.7089	2.9292	656.0915	74.1342	1377.6285	0.8212
142.3422	4.4623	657.5508	41.9902	2415.2522	1256.1024
159.6802	70.0185	658.1794	33.199	2416.4614	440.7069
165.5131	1.0811	658.7969	17.0611	2420.6906	123.2662
171.8426	78.138	666.079	36.7986	2421.8183	391.1901
180.1518	1.8438	666.748	43.1782	2421.9742	1056.9201
207.5517	4.2406	671.2344	11.6011	2430.9097	1064.7968
637.6062	6.272	671.5517	51.7764	2433.4274	2146.6638
639.4373	56.6812	915.145	191.5003	2437.8267	1959.7367
644.259	56.0526	1363.8403	53.3358	2470.0543	4.1248

Table S173. Cartesian coordinates for the optimized geometry of isomer 9c-singlet $UO^{2+}(CO_2)_9$ followed by its predicted frequencies (cm^{-1}) and IR intensities (km/mol).

Z	x	y	z
92	-0.480162000	-0.000146000	-0.264780000
8	-0.524990000	0.000147000	-2.065709000
8	1.354562000	-0.001164000	1.890680000
6	2.346418000	-0.002973000	2.519333000
8	3.308158000	-0.004375000	3.141869000
8	-1.763573000	3.140808000	3.471685000
6	-1.473415000	2.353664000	2.693613000
8	-1.175359000	1.541922000	1.896878000
8	-1.176103000	-1.542562000	1.896239000
6	-1.476173000	-2.354946000	2.691565000
8	-1.768374000	-3.142521000	3.468433000
8	1.541746000	-1.634286000	-0.551136000
6	2.430528000	-2.173764000	-1.103528000
8	3.289633000	-2.709861000	-1.633123000
8	-3.100828000	0.000033000	-0.088056000
8	-5.389244000	0.004767000	-0.470921000
6	-4.261617000	0.002513000	-0.285779000
6	-1.639132000	-3.133968000	-1.758557000
8	-1.392610000	-2.447247000	-0.832302000
8	-1.881455000	-3.812067000	-2.645906000
8	1.541396000	1.634779000	-0.549053000
6	2.430555000	2.175206000	-1.099905000
8	3.290177000	2.712809000	-1.627131000
6	-1.636943000	3.134776000	-1.757969000
8	-1.877074000	3.813633000	-2.645335000
8	-1.392940000	2.447084000	-0.831765000
6	5.499535000	-0.000260000	-0.074977000
8	4.325853000	-0.000223000	-0.070947000
8	6.648076000	-0.000187000	-0.077281000

Frequency	Intensity	Frequency	Intensity	Frequency	Intensity
7.2764	0.0004	29.921	0.0954	60.5701	2.4445
14.6976	0.079	30.4842	0.0073	70.3767	0.0918
16.0958	0.0525	35.8572	0.61	75.2443	0.1034
17.3309	0.1639	37.0164	0.0734	76.0264	2.0546
20.1483	0.2523	38.5402	0.1451	78.3725	0.1848
21.6011	0.2676	42.1421	0.67	80.7223	0.01
22.2727	0.1156	42.8262	0.8653	94.3388	0.392
26.9393	0.0086	46.288	0.574	95.6781	0.0094
27.7466	0.0588	52.346	0.0511	100.716	2.6502
29.3535	0.0775	54.2044	0.0161	104.3798	6.0517

109.964	13.1335	648.3848	15.711	1363.9384	52.2288
112.6477	1.1795	648.8763	1.5496	1364.1839	20.1446
117.2877	10.3182	651.2284	1.0491	1371.7659	6.3272
118.6261	4.3222	652.3931	49.6802	1372.3644	27.9785
130.7938	3.424	653.0096	89.8491	1372.7078	62.2423
133.4667	0.3342	654.1296	23.1751	1373.2929	72.2162
133.8252	0.2795	656.139	16.4486	1373.8639	152.5558
137.4927	20.3062	657.4265	88.8873	1377.9723	4.7558
150.6753	2.6192	658.0347	8.5303	2414.6985	1161.2348
151.5333	42.9121	659.541	84.0413	2414.9921	16.4865
155.0496	73.3417	659.8867	12.7982	2415.9111	155.126
175.3255	0.9774	660.6404	27.415	2420.7796	195.2995
189.339	6.9997	660.869	3.9076	2423.9093	115.0339
231.8861	8.6758	668.4855	33.2084	2431.4769	2053.1716
642.3765	17.9538	670.6467	40.6776	2431.9409	2023.8079
644.9031	22.5686	909.972	197.5626	2437.326	2634.6562
647.9303	32.0538	1363.2672	39.7456	2471.9198	29.9176

Table S174. Cartesian coordinates for the optimized geometry of isomer 9c-triplet $UO^{2+}(CO_2)_9$ followed by its predicted frequencies (cm^{-1}) and IR intensities (km/mol).

Z	x	y	z
92	-0.484283000	-0.001637000	-0.264045000
8	-0.556969000	-0.001050000	-2.065485000
8	1.366038000	-0.006669000	1.886404000
6	2.355135000	-0.007252000	2.519381000
8	3.314737000	-0.007827000	3.145217000
8	-1.757785000	3.158421000	3.461439000
6	-1.461970000	2.362594000	2.694473000
8	-1.157836000	1.541901000	1.909153000
8	-1.171444000	-1.532580000	1.914079000
6	-1.480603000	-2.347258000	2.703686000
8	-1.781309000	-3.137143000	3.474876000
8	1.547247000	-1.620848000	-0.578597000
6	2.436945000	-2.157381000	-1.132234000
8	3.297031000	-2.690769000	-1.662989000
8	-3.100614000	-0.001524000	-0.092074000
8	-5.378229000	0.005593000	-0.534772000
6	-4.255934000	0.002122000	-0.320612000
6	-1.638729000	-3.173477000	-1.719156000
8	-1.391646000	-2.467959000	-0.807578000
8	-1.881752000	-3.869165000	-2.592852000
8	1.542936000	1.622956000	-0.573584000
6	2.432705000	2.161403000	-1.125346000
8	3.292789000	2.696779000	-1.654035000
6	-1.646797000	3.164621000	-1.728563000
8	-1.889610000	3.856059000	-2.605737000
8	-1.399978000	2.463412000	-0.813630000
6	5.507351000	0.001931000	-0.070366000
8	4.333667000	0.000953000	-0.069454000
8	6.655902000	0.002812000	-0.069812000

Frequency	Intensity	Frequency	Intensity	Frequency	Intensity
-52.127	19.3732	29.4665	0.0518	58.7992	2.4563
7.1177	0.001	29.9538	0.0849	60.5404	2.3415
14.7797	0.0829	35.3951	0.9169	70.4721	0.2038
16.3643	0.129	35.8686	0.6586	75.5161	0.1334
17.594	0.1674	38.5257	0.5918	75.744	2.4736
21.2203	0.1172	39.0731	0.49	77.3011	0.0021
22.3864	0.1303	43.0353	0.9196	89.6987	1.2887
24.5883	0.0234	45.0041	0.423	95.4071	0.2644
26.7455	0.0089	46.4938	0.1116	99.3845	8.8313
28.9783	0.0209	52.0875	0.0344	101.4591	17.3927

104.1307	7.3336	648.411	27.0627	1364.029	35.9846
111.8509	1.2108	649.0021	8.5622	1364.2862	33.1191
112.4591	0.2741	651.6573	2.9798	1371.7854	3.992
117.6013	10.4108	652.5096	47.3752	1372.3556	43.2374
131.5206	3.4036	653.1617	96.3619	1372.7969	46.7902
132.8334	12.4298	654.4696	12.7019	1373.2762	69.9168
134.0008	0.1514	656.5654	15.576	1373.8825	162.6173
135.0047	15.6553	657.3543	93.8729	1378.0316	4.3117
150.207	14.7766	658.224	11.0384	2414.1069	59.718
150.4994	6.1712	659.7805	81.2353	2414.7201	1103.8256
155.6077	73.7539	660.0644	11.6595	2415.8643	177.9928
173.7584	0.515	660.8026	32.9374	2420.7021	181.5734
187.1713	7.4657	661.1208	1.887	2423.4637	116.9554
221.5962	5.1274	668.5615	33.3623	2431.6011	2023.9598
641.7057	15.8791	670.6083	41.5481	2431.7376	2035.0376
644.9615	25.9249	908.0683	192.3836	2437.8107	2592.3812
647.4854	11.8013	1363.5475	43.2424	2471.7542	32.8127

Table S175. Cartesian coordinates for the optimized geometry of isomer 9d-triplet $UO^{2+}(CO_2)_9$ followed by its predicted frequencies (cm^{-1}) and IR intensities (km/mol).

Z	x	y	z
92	0.542791000	0.000087000	0.220428000
8	1.659160000	0.000026000	1.637152000
8	-4.319948000	0.000008000	-0.614159000
6	-5.491902000	-0.000030000	-0.636756000
8	-6.641331000	-0.000066000	-0.655917000
8	-3.219548000	2.847693000	-1.533933000
6	-2.344729000	2.173379000	-1.235542000
8	-1.433138000	1.494398000	-0.936170000
8	-1.433126000	-1.494344000	-0.936124000
6	-2.344728000	-2.173531000	-1.234994000
8	-3.219561000	-2.848043000	-1.532896000
8	-0.819258000	-1.546562000	1.909848000
6	-0.853843000	-2.038243000	2.980135000
8	-0.900715000	-2.520888000	4.015176000
8	3.027163000	0.001136000	-0.895919000
8	5.253536000	0.001317000	-0.236743000
6	4.154950000	0.001226000	-0.554321000
6	1.954367000	-3.472358000	-0.067028000
8	1.353006000	-2.467534000	-0.194458000
8	2.532537000	-4.451035000	0.048410000
8	-0.820453000	1.544855000	1.910292000
6	-0.855791000	2.036309000	2.980660000
8	-0.903402000	2.518721000	4.015773000
6	1.952997000	3.473215000	-0.065555000
8	2.531049000	4.451928000	0.050165000
8	1.351750000	2.468357000	-0.193265000
6	0.961747000	-0.000344000	-3.653656000
8	1.263441000	-0.000446000	-4.756884000
8	0.646943000	-0.000240000	-2.519978000

Frequency	Intensity	Frequency	Intensity	Frequency	Intensity
4.1766	0.0169	29.1637	0.0067	59.7639	1.9424
7.1028	0.1624	31.076	0.0061	66.975	0.4595
15.4658	0.0273	35.0128	1.1839	72.7225	1.0176
17.0159	0.0083	35.2122	0.0359	73.6002	0.0469
19.9158	0.2038	37.0844	0.2326	87.9826	0.4036
19.9356	0.3758	38.4326	0.2472	89.2431	2.1306
23.6869	0.384	41.2733	0.3029	93.5082	0.7407
24.7467	0.0478	44.8887	2.4914	96.1454	1.5315
25.6818	0.0321	48.3229	0.1769	102.8989	4.9509
27.0235	0.0328	54.779	0.3475	110.1007	2.2442

110.6383	0.6576	649.7689	9.8774	1366.0286	15.2627
115.1268	0.3471	650.3803	1.8887	1366.1637	16.827
115.9675	4.0182	651.2544	0.4005	1367.744	90.2583
124.4395	15.3143	651.9348	43.7961	1371.3701	47.0461
129.1168	3.0424	654.2952	55.7674	1371.7583	62.5641
132.4487	14.657	655.8842	125.1096	1372.6089	88.339
134.5046	1.1381	656.3083	4.1213	1373.7221	79.5763
141.2682	2.8108	657.1384	8.6312	1377.9689	19.0421
145.1502	46.5385	657.1525	83.1419	2414.2676	193.1565
148.4399	11.7577	659.12	58.5351	2414.8207	13.1676
153.4806	65.1456	661.6064	30.6643	2415.2057	1205.7177
174.1307	1.9738	662.2539	0.4137	2422.1288	9.2607
194.6812	20.552	663.2506	10.4455	2423.8994	74.8094
230.6991	16.4954	668.3079	46.7084	2430.6106	2339.3752
642.9396	13.1257	670.8154	33.7285	2433.6646	2036.1032
648.0111	40.0533	903.6196	196.1152	2434.5161	2508.7671
648.591	0.7937	1363.2823	36.498	2470.8324	2.4031

Table S176. Cartesian coordinates for the optimized geometry of isomer 9e-singlet $UO^{2+}(CO_2)_9$ followed by its predicted frequencies (cm^{-1}) and IR intensities (km/mol).

Z	x	y	z
92	0.134383000	-0.571307000	-0.208673000
8	-2.251633000	-1.243112000	-0.125424000
6	-3.405226000	-1.392713000	0.081118000
8	-4.515352000	-1.566025000	0.265047000
8	-0.036689000	-2.873693000	-1.251292000
6	-0.183991000	-3.924928000	-1.768983000
8	-0.324416000	-4.940047000	-2.267165000
8	1.643625000	1.288873000	0.439587000
6	2.364802000	2.208257000	0.610335000
8	3.061356000	3.091508000	0.787431000
8	-0.114890000	0.154728000	-1.768803000
6	-1.312916000	1.471390000	0.936069000
8	-1.874702000	2.308351000	1.412792000
8	2.454731000	-1.264293000	-0.891716000
6	3.573272000	-1.572141000	-1.110871000
8	4.644540000	-1.884467000	-1.341153000
8	0.376805000	-1.276388000	1.362141000
8	-5.386336000	2.388731000	-2.296027000
8	-3.755439000	1.171835000	-1.178607000
6	-4.580767000	1.784545000	-1.741838000
8	0.340333000	3.784953000	-0.689510000
8	0.088654000	5.896669000	-1.620501000
6	0.215494000	4.852026000	-1.158228000
6	5.635206000	0.379394000	1.460566000
8	4.582114000	0.287456000	0.951347000
8	6.665151000	0.468592000	1.961065000
8	-3.664628000	-0.875633000	4.944486000
6	-3.231019000	-0.756729000	3.887022000
8	-2.784777000	-0.634837000	2.809647000

Frequency	Intensity	Frequency	Intensity	Frequency	Intensity
5.7521	0.0371	34.2831	0.0912	58.1536	0.5717
6.6183	0.0363	37.7647	0.4048	61.5637	0.5591
11.9817	0.1003	43.7371	0.0699	67.4151	0.1629
12.1915	0.0857	45.0039	0.2779	69.3604	0.1893
13.0282	0.0832	46.4116	1.4484	72.783	0.1931
14.6973	0.1448	47.9193	0.8368	79.008	0.0262
20.0719	0.365	50.0347	0.7835	80.5972	1.1093
22.1875	0.0489	53.5455	0.1191	83.4099	0.9103
27.3519	0.0034	54.9336	0.6253	89.0417	2.0856
31.5917	0.1455	56.9083	0.6184	96.1611	1.2856

99.0536	0.2205	625.2247	52.4182	1365.3749	25.2863
120.1784	1.1469	634.3296	48.5634	1366.9942	24.4256
128.5128	0.56	638.8189	14.0666	1367.2516	10.3896
138.5627	1.4441	639.986	50.6113	1367.5148	28.6528
150.1787	0.2103	641.4835	54.4612	1372.9519	33.139
153.1399	1.7018	645.1828	42.4733	1374.4362	134.6711
155.1452	1.5334	648.1821	9.6478	1375.3173	246.8619
169.6794	8.0036	665.4828	6.487	1382.0575	18.0894
177.2671	2.958	665.8382	11.0725	2310.6563	22.2472
177.9746	3.7927	666.2991	42.3124	2413.6049	108.7625
190.1934	2.2333	667.0189	60.9359	2415.6752	1646.424
191.1757	22.6316	668.74	31.722	2416.687	1588.2258
238.8271	66.4475	669.6072	46.2539	2423.5114	928.8451
241.3185	92.4678	671.1349	20.9742	2430.1829	26.9609
251.0727	5.2444	671.5041	31.346	2436.6895	1060.0831
273.2046	1.3319	957.3361	0.0843	2443.7417	2030.001
620.9265	60.7144	1036.1022	258.0644	2466.69	158.3738

Table S177. Cartesian coordinates for the optimized geometry of isomer 9e-triplet $UO^{2+}(CO_2)_9$ followed by its predicted frequencies (cm^{-1}) and IR intensities (km/mol).

Z	x	y	z
92	0.129975000	-0.580553000	-0.214785000
8	-2.267591000	-1.223318000	-0.111652000
6	-3.421089000	-1.363089000	0.101298000
8	-4.531671000	-1.526331000	0.292925000
8	-0.071126000	-2.879538000	-1.250650000
6	-0.230098000	-3.933851000	-1.758989000
8	-0.381826000	-4.951562000	-2.248362000
8	1.640770000	1.283706000	0.431746000
6	2.365286000	2.194164000	0.632438000
8	3.064913000	3.069188000	0.837364000
8	-0.122944000	0.161523000	-1.828451000
6	-1.310158000	1.485869000	0.925266000
8	-1.869436000	2.327868000	1.396857000
8	2.444691000	-1.279899000	-0.896275000
6	3.561959000	-1.576819000	-1.136986000
8	4.631614000	-1.878967000	-1.387187000
8	0.381729000	-1.311001000	1.404489000
8	-5.386999000	2.410483000	-2.317810000
8	-3.762721000	1.185789000	-1.199262000
6	-4.584652000	1.802399000	-1.762953000
8	0.378760000	3.812246000	-0.681658000
8	0.147143000	5.938173000	-1.585089000
6	0.264051000	4.886324000	-1.136359000
6	5.639841000	0.351574000	1.439410000
8	4.584865000	0.261093000	0.933933000
8	6.671687000	0.439367000	1.936310000
8	-3.611571000	-0.858060000	4.985146000
6	-3.190107000	-0.745500000	3.921905000
8	-2.756274000	-0.630210000	2.838879000

Frequency	Intensity	Frequency	Intensity	Frequency	Intensity
5.7582	0.039	37.7033	0.3231	66.7639	0.0738
6.295	0.0412	43.3684	0.1717	68.7137	0.0362
11.3621	0.0155	44.7579	0.2174	70.3012	0.2506
11.6005	0.2485	46.1415	1.2599	77.8445	0.4485
12.3832	0.1236	47.745	0.794	79.0498	0.1102
14.1129	0.2158	49.4935	0.7113	82.5631	0.5491
19.8022	0.3475	52.9203	0.133	88.3097	1.4096
21.8223	0.0822	54.4964	0.5189	96.3743	0.8716
27.3037	0.0102	55.7872	0.4858	100.0916	0.0601
31.6456	0.0901	57.2625	0.25	118.8456	0.9115
34.0445	0.0754	60.4123	0.5653	128.2191	0.7447

136.8702	1.3637	639.9478	8.534	1366.7881	28.9882
142.6037	0.5051	640.9983	49.4698	1367.2673	12.5417
152.1906	2.3938	642.3965	44.5575	1367.5118	33.3936
152.7225	1.4872	645.8651	42.3429	1372.0955	35.9205
167.0701	4.9389	648.4294	9.1423	1373.3848	176.225
168.109	0.2206	665.3606	9.8229	1374.1873	210.784
174.2447	9.6928	665.8068	8.2579	1380.4477	38.1796
186.1182	1.8781	666.1801	36.1471	2306.9022	21.6323
186.7959	22.38	666.8989	58.3141	2411.6806	607.6658
222.7491	63.0867	668.5941	23.4142	2413.3116	853.4548
223.5748	93.6391	669.2159	53.4218	2415.9327	1961.4651
244.3192	4.3968	670.9241	17.5152	2421.5553	870.2345
272.868	0.9807	671.5137	30.9048	2428.1185	18.3117
621.9975	55.3454	690.2741	17.1534	2434.1696	977.0242
626.4776	43.1691	820.1075	0.0294	2439.963	1997.0049
634.8363	51.4608	1365.3817	28.0569	2461.1646	335.6202

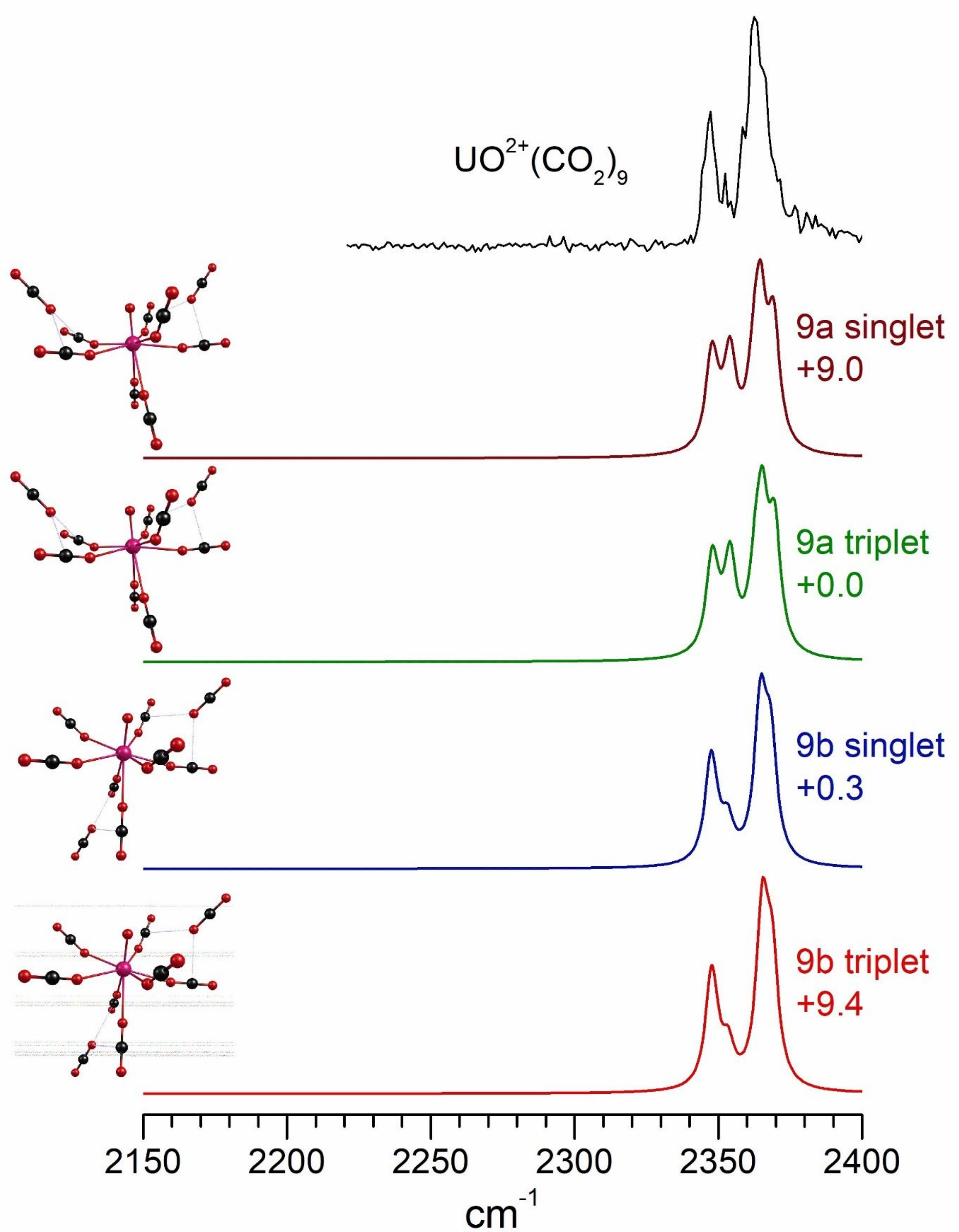

Figure S134. Experimental IR spectrum of $UO^{2+}(CO_2)_9$ compared with simulated spectra for isomers 9a and 9b. Relative energies (kcal/mol) are shown next to each spectrum.

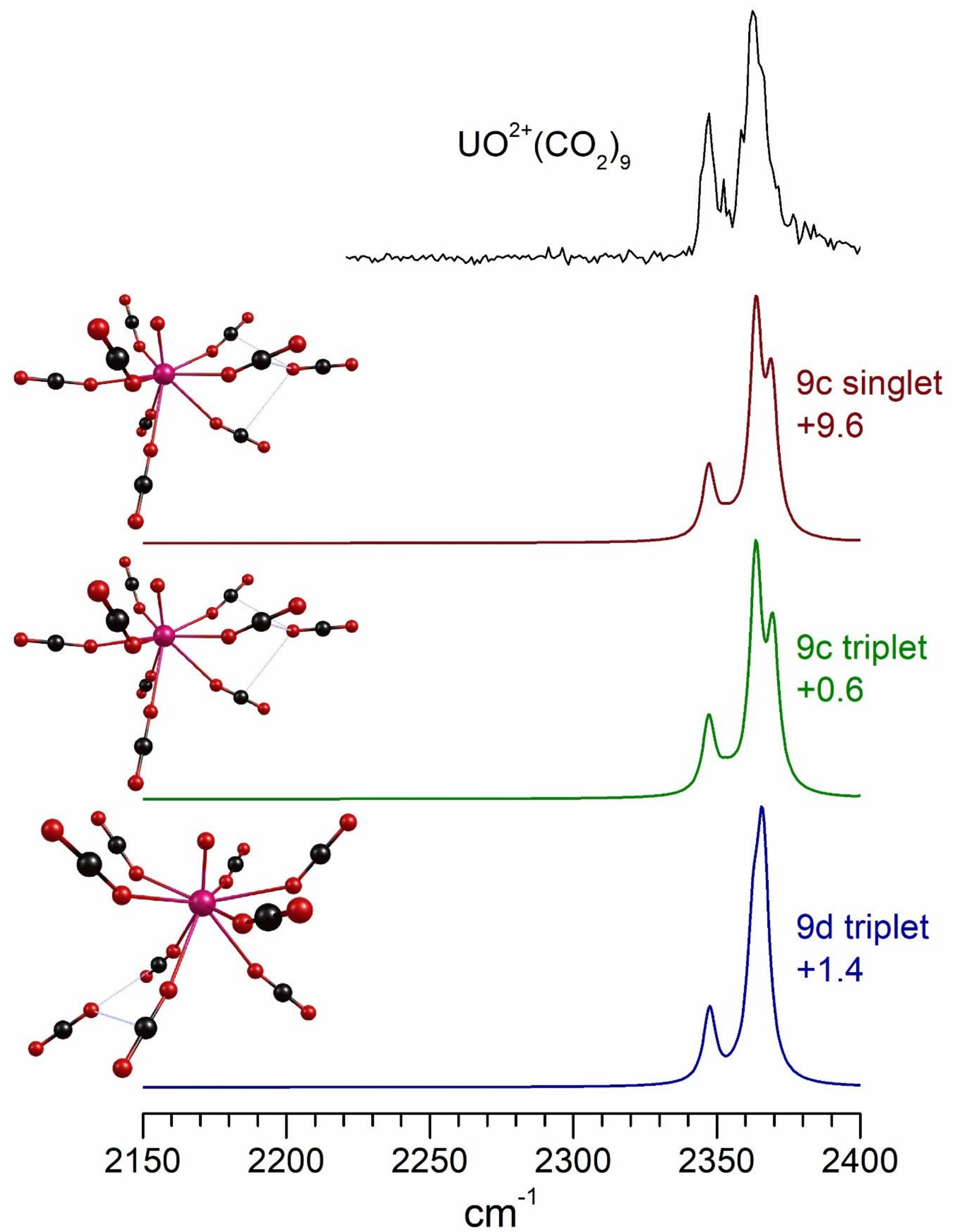

Figure S135. Experimental IR spectrum of $UO^{2+}(CO_2)_9$ compared with simulated spectra for isomers 9c and 9d. Relative energies (kcal/mol) are shown next to each spectrum.
